# Case management interventions seeking to counter radicalisation to violence and related forms of violence: A systematic review

**DOI:** 10.1002/cl2.1386

**Published:** 2024-04-12

**Authors:** James Lewis, Sarah Marsden, Adrian Cherney, Martine Zeuthen, Lotta Rahlf, Chloe Squires, Anne Peterscheck

**Affiliations:** ^1^ Handa Centre for the Study of Terrorism and Political Violence (CSTPV), School of International Relations University of St Andrews St Andrews, Fife Scotland UK; ^2^ School of Social Science University of Queensland Brisbane Queensland Australia; ^3^ Royal United Services Institute (RUSI) Mombasa Kenya; ^4^ Peace Research Institute Frankfurt (PRIF) Frankfurt Germany

## Abstract

**Background:**

Increasingly, counter‐radicalisation interventions are using case management approaches to structure the delivery of tailored services to those at risk of engaging in, or engaged in, violent extremism. This review sets out the evidence on case management tools and approaches and is made up of two parts with the following objectives.

**Objectives:**

*Part I*: (1) Synthesise evidence on the effectiveness of case management tools and approaches in interventions seeking to counter radicalisation to violence. (2) Qualitatively synthesise research examining whether case management tools and approaches are implemented as intended, and the factors that explain how they are implemented. *Part II*: (3) Synthesise systematic reviews to understand whether case management tools and approaches are effective at countering non‐terrorism related interpersonal or collective forms of violence. (4) Qualitatively synthesise research analysing whether case management tools and approaches are implemented as intended, and what influences how they are implemented. (5) Assess the transferability of tools and approaches used in wider violence prevention work to counter‐radicalisation interventions.

**Search Methods:**

Search terms tailored for Part I and Part II were used to search research repositories, grey literature sources and academic journals for studies published between 2000 and 2022. Searches were conducted in August and September 2022. Forward and backward citation searches and consultations with experts took place between September 2022 and February 2023. Studies in English, French, German, Russian, Swedish, Norwegian and Danish were eligible.

**Selection Criteria:**

*Part I*: Studies had to report on a case management intervention, tool or approach, or on specific stages of the case management process. Only experimental and stronger quasi‐experimental studies were eligible for inclusion in the analysis of effectiveness. The inclusion criteria for the analysis of implementation allowed for other quantitative designs and qualitative research. *Part II*: Systematic reviews examining a case management intervention, tool or approach, or stage(s) of the case management process focused on countering violence were eligible for inclusion.

**Data Collection and Analysis:**

*Part I*: 47 studies were eligible for Part I. No studies met the inclusion criteria for Objective 1; all eligible studies related to Objective 2. Data from these studies was synthesised using a framework synthesis approach and presented narratively. Risk of bias was assessed using the CASP (for qualitative research) and EPHPP (for quantitative research) checklists. *Part I*: Eight reviews were eligible for Part II. Five reviews met the inclusion criteria for Objective 3, and seven for Objective 4. Data from the studies was synthesised using a framework synthesis approach and presented narratively. Risk of bias was assessed using the AMSTAR II tool.

**Findings:**

*Part I*: No eligible studies examined effectiveness of tools and approaches. Seven studies examined the implementation of different approaches, or the assumptions underpinning interventions. Clearly defined theories of change were absent, however these interventions were assessed as being implemented in line with their own underlying logic. Forty‐three studies analysed the implementation of tools during individual stages of the case management process, and forty‐one examined the implementation of this process as‐a‐whole. Factors which influenced how individual stages and the case management process as a whole were implemented included strong multi‐agency working arrangements; the inclusion of relevant knowledge and expertise, and associated training; and the availability of resources. The absence of these facilitators inhibited implementation. Additional implementation barriers included overly risk‐oriented logics; public and political pressure; and broader legislation. Twenty‐eight studies identified moderators that shaped how interventions were delivered, including delivery context; local context; standalone interventions; and client challenges. *Part II*: The effectiveness of two interventions – mentoring and multi‐systemic therapy – in reducing violent outcomes were each assessed by one systematic review, whilst three reviews analysed the impact that the use of risk assessment tools (*n* = 2) and polygraphs (*n* = 1) had on outcomes. All these reviews reported mixed results. Comparable factors to those identified in Part I, such as staff training and expertise and delivery context, were found to shape implementation. On the basis of this modest sample, the research on interventions to counter non‐terrorism related violence was assessed to be transferable to counter‐radicalisation interventions.

**Authors' Conclusions:**

The effectiveness of existing case management tools and approaches is poorly understood, and research examining the factors that influence how different approaches are implemented is limited. However, there is a growing body of research on the factors which facilitate or generate barriers to the implementation of case management interventions. Many of the factors and moderators relevant to countering radicalisation to violence also impact how case management tools and approaches used to counter other forms of violence are implemented. Research in this wider field seems to have transferable insights for efforts to counter radicalisation to violence. This review provides a platform for further research to test the impact of different tools, and the mechanisms by which they inform outcomes. This work will benefit from using the case management framework as a way of rationalising and analysing the range of tools, approaches and processes that make up case managed interventions to counter radicalisation to violence.

## 1 PLAIN LANGUAGE SUMMARY

### 1.1 Case management tools and approaches are widely used in countering radicalisation to violence programmes, but their effectiveness is unclear

Case management tools and approaches were found to support counter‐radicalisation work when implemented appropriately. No eligible evaluations of effectiveness were identified. Research on tools and approaches used to counter non‐terrorism related violence is more developed, however robust evaluations of effectiveness are largely absent.

### 1.2 What is this review about?

This review has two parts. Part I is a systematic review of case management tools and approaches used in counter‐radicalisation interventions and has three objectives: (1) assess the effectiveness of tools and approaches; (2a) examine whether they are implemented as intended; and (2b) identify factors that explain this implementation.

Part II is an overview of systematic reviews examining tools and approaches used to counter other forms of violence and has the following objectives: (3) examine the effectiveness of tools and approaches; (4a) assess their implementation; (4b) identify factors that explain their implementation; and (5) analyse whether these tools and approaches are transferable to counter‐radicalisation work.

### 1.3 What studies are included?

#### 1.3.1 Part I – Countering radicalisation to violence

No eligible studies spoke to Objective 1.

Forty‐seven studies related to Objective 2. Research on Objective 2a (*n* = 7 studies) focused on approaches. Research on Objective 2b focused on implementation factors pertaining to stages of the case management process (*n* = 43); and implementation factors (*n* = 41) and moderators (*n* = 28) relevant to the full process.

#### 1.3.2 Part II – Countering other forms of violence

Eight reviews were included. Five examined the effectiveness of case management approaches (*n* = 2) and tools (*n* = 3) (Objective 3); two examined how tools were implemented (Objective 4a); and seven considered implementation factors and moderators (Objective 4b).

### 1.4 What are the findings of this review?

#### 1.4.1 Are case management tools and approaches effective in countering radicalisation to violence?

It is not possible to draw conclusions about effectiveness as no eligible studies were identified.

#### 1.4.2 Are case management tools and approaches implemented as intended?

Four studies concluded that the assumptions underpinning three interventions were sound and aligned with academic research. Four studies reported mixed results as to whether three interventions were implemented in line with their internal logic. Two studies highlighted weaknesses in programme logic, including misalignment between activities and intended outcomes.

#### 1.4.3 What explains how tools and approaches are implemented?

##### Different stages of case management

Two studies examined the *identification* stage, highlighting how working arrangements with external partners can create challenges when engaging potential clients. Research on the *client assessment* stage (*n* = 26 studies) examined multi‐agency assessment (*n* = 14); risk and needs assessment (RNA) tools (*n* = 12); and screening tools (*n* = 3). Themes included inconsistency in tool use; subjectivity in interpreting risk; differing opinions on the utility of tools; and the importance of expertise and experience, and organisational and operational support for assessors. Effective multi‐agency collaboration was important.

Evidence on *case planning* was limited (*n* = 5), and it remains unclear whether case planning is informed by client identification and assessment or informs delivery. Research on the use of case planning tools and case conferences identified similar themes to that on client assessment.

Research on the *delivery* stage (*n* = 28) highlighted the benefits of tailoring support to client needs, and skilled and committed practitioners who were well matched to clients and able to build trust.

*Monitoring and evaluation* tools (*n* = 16) included client assessment tools (*n* = 9); case file and case note data (*n* = 7); case conferences (*n* = 5); and less structured qualitative data (*n* = 5). Assessment tools were considered able to monitor change, inform evaluations, and support delivery, but were used inconsistently. Case notes and files help capture relevant data, whilst case conferences enable plausibility checking. However, there is limited consensus over how to interpret client change.

Studies examining *transition and exit* (*n* = 10) highlighted the importance of inter‐agency coordination and continuity in client support. Potential challenges included reticence to close cases; ending relationships smoothly; and difficulties monitoring clients post‐exit.

##### Implementation factors and moderators affecting the case management process

Implementation factors and moderators relevant to the whole case management process included effective multi‐agency working (*n* = 34), potential challenges to which included information sharing and relationships between partners. Staff expertise was a facilitator (*n* = 23), whilst an over emphasis on risk‐oriented logics (*n* = 17); political and public pressure (*n* = 10); and resourcing challenges (*n* = 17) were identified as implementation barriers. Eight studies highlighted how broader counter‐terrorism legislation might undermine rehabilitative aims.

The benefits of mandated versus voluntary interventions remain unclear (*n* = 11). Practitioners appear to prefer voluntary approaches, but discussed challenges engaging clients unwilling to participate voluntarily.

Moderators included features of the local context (*n* = 10) and the delivery context (*n* = 11); the distinction between standalone counter‐radicalisation work and interventions or practitioners that deliver this alongside other work (*n* = 4); and the impact of broader challenges in a client's life (*n* = 4).

#### 1.4.4 Are case management tools and approaches effective at countering interpersonal and collective forms of violence?

The effectiveness of case management in countering other forms of violence remains unclear. Two reviews examining the effectiveness of interventions did not find conclusive evidence that they effectively countered violence. Three reviews on risk assessment tools (*n* = 2) and polygraphs (*n* = 1) reported mixed results. However, use of these tools alone would not be expected to directly reduce violence as any positive impact would be indirect.

#### 1.4.5 How are case management tools and approaches implemented in the context of countering collective and interpersonal forms of violence?

Evidence focused almost entirely on risk assessment tools (*n* = 5). Two reviews found that risk management is not always directly informed by structured risk assessment. The extent to which practitioners use risk assessment tools to inform case planning is informed by their willingness to take risk assessments into account when making decisions, and their ability to offer services that can effectively target needs or risks. Feedback on the perceived utility of these tools was therefore found to be mixed. Whilst feedback on the use of polygraphs was positive, this feedback was drawn from a limited evidence base (*n* = 1).

Facilitators of risk assessment include the ability to adapt tools to local needs; training and guidance; opportunities to pilot tools; professional ownership; positive relationships with clients; and multi‐disciplinary working. Barriers included uncertainty about the utility of tools; insufficient room for clinical judgement; the perceived complexity and resource intensity of assessment; lack of experience and perceived self‐efficacy; subjective interpretations of risk; and uncertainty around translating assessments into practical action. Expertise, training, and time spent with clients facilitated the implementation of mentoring programmes.

#### 1.4.6 Are case management tools and approaches used to counter other forms of violence transferable to counter‐radicalisation work?

The research in Part II was considered transferable to counter‐radicalisation interventions. Risk assessment tools and mentoring approaches are already widely used within counter‐radicalisation interventions. The utility of using polygraphs has also been considered, however evidence for their effectiveness is insufficient to recommend their implementation. Evidence relating to the use of Multi‐Systemic Therapy (MST) for countering radicalisation to violence  was not identified in the literature included in Part I, however its adherence to socio‐ecological models of violence prevention suggests it is potentially transferrable.

### 1.5 What do the findings of this review mean?

Limited evidence exists for the effectiveness of case management. A body of research (47 studies) has identified factors which can facilitate or generate barriers to the implementation of interventions. The quality of this evidence is uneven. The case management framework provides a useful means of organising research on the different tools. The field will now benefit from research to test the impact of these tools and underlying approaches, and the mechanisms by which they shape intervention outcomes. More detailed analysis of case management in other fields may also strengthen counter‐radicalisation research and practice.

### 1.6 How up‐to‐date is this review?

Literature searches were completed in January 2023, and include studies first published between 2000 and 2023.

## 2 BACKGROUND

### 2.1 The problem, condition or issue

The concept of radicalisation is contested and complex. Although it can be used in different ways by policymakers and academics, it is often described as having attitudinal and behavioural features that refer to the adoption of radical or extreme beliefs and the justification and use of violence (Neumann, 2013). This has led to a distinction being drawn between cognitive and behavioural radicalisation (Wolfowicz et al., 2021); the former typically describes a process through which an individual comes to adopt extremist beliefs, and the latter framing the end point of radicalisation as involvement in violent behaviour (Neumann, 2013).

A range of models of radicalisation have been developed (e.g., see Borum, 2012; Kruglanski et al., 2018; McCauley & Moskalenko, 2017), however it is widely accepted that there is no uniform radicalisation process, nor is there a common profile of those who become radicalised (Horgan, 2008). Research instead describes pathways into violent extremism as a function of complex, individualised interactions between push and pull factors operating at different levels of analysis (Lewis & Marsden, 2021). There remains some debate over the precise nature of this radicalisation process, with research highlighting how some push and pull factors may be present across multiple cases of radicalisation (Vergani et al., 2020; Wolfowicz et al., 2021), particularly when radicalised individuals emerge from similar, or the same, contexts (e.g., Neve et al., 2020). However, whilst similar factors may be implicated in multiple cases of radicalisation, it is now widely accepted that no single factor causes radicalisation (Lewis & Marsden, 2021). This means that even when similar factors are relevant across multiple cases, individual journeys into violent extremism are driven by interactions between these factors that are specific to each individual, and the specific context(s) in which they are situated.

Despite the contention surrounding the concept of radicalisation (Githens‐Mazer & Lambert, 2010; Kundnani, 2012), it remains a dominant feature of counter‐terrorism policy and practice. A range of interventions have been developed to engage with those considered ‘at risk’ of radicalisation, and those who have become involved in violent extremism and/or been convicted of a terrorist offence (Pistone et al., 2019). Interventions can take different forms, from one‐to‐one mentoring; education, training or vocational provision; ideological guidance; family‐based programmes; mental health support; or help with practical issues such as housing (Koehler, 2017). Research on the process and impact of these interventions is in its infancy, and there is only limited understanding of what works to reduce the risk of involvement or re‐engagement in violent extremism (Hassan et al., 2021a, 2021b; Zeuthen, 2021).

There is also a lack of clarity over what the appropriate aims of interventions to counter radicalisation to violence should be. A distinction is commonly made between deradicalisation and disengagement; the former is typically used to refer to the process of rejecting extreme, violent supportive ideas and attitudes, whilst the latter refers to behavioural changes that see an individual move away from an extremist group (Horgan, 2009). Historically, state efforts focused on enforcing or encouraging disengagement, often through arrest or incentives (Silke, 2011). Over time, attention shifted to the role of ideology, and the potential benefits of trying to change the attitudes and beliefs believed to support violence (Koehler, 2016). More recently, research and practice has come to recognise that, similarly to radicalisation processes, deradicalisation and disengagement are driven by complex, individualised push and pull factors, that demand multi‐dimensional approaches to supporting change (Ellis et al., 2022).

A recent feature of interventions seeking to counter radicalisation to violence is the use of case management tools and approaches (Cherney & Belton, 2021a). Case management involves a tailored approach to working with individuals that structures the process and type of support they receive, from initial assessment through to case planning and exit (Cherney et al., 2020). These types of programmes have been used in a range of other contexts, including social work, corrections, and healthcare (Lukersmith et al., 2016). They have also seemingly been effective in programmes that seek to counter involvement in violence (e.g., Brantingham et al., 2021; Engel et al., 2013), including violence motivated by political or religious ideologies (Weine et al., 2021).

Case management interventions are considered potentially useful in the context of countering radicalisation to violence because the tailored approach they take can accommodate the individualised nature of radicalisation processes (Cherney et al., 2020). However, research on the nature and impact of case management in this context remains limited (Bellasio et al., 2018; Feddes & Gallucci, 2015; Pistone et al., 2019). A modest amount of attention has been directed at the implementation of case management interventions (e.g., Cherney & Belton, 2021a; Harris‐Hogan, 2020) and some research has been carried out on specific stages of the case management process, such as risk assessment (e.g., Scarcella et al., 2016). However, there has been no attempt to systematically assess the tools and approaches used in case management interventions seeking to counter radicalisation to violence.

There are a number of reasons for the limited empirical research on the process and impact of case management interventions in counter‐radicalisation work. Impact evaluations are hampered by methodological and analytical challenges such as identifying appropriate outcome indicators; establishing base rates against which intervention outcomes might be measured; ethical and security challenges associated with using control groups; and difficulties accessing data (Baruch et al., 2018; Lewis et al., 2020). Whereas process evaluations face challenges due to the complexity of interventions that often involve multiple stakeholders and processes operating at different stages of an individual's involvement with case management processes (Lewis et al., 2020).

A broader challenge is the exceptionalism with which interventions that counter radicalisation to violence are often treated, as this can mean insights from different types of intervention or policy context may be missed (Lewis et al., 2020). Together this means that the process by which case management interventions are delivered is rarely considered holistically, and insights from other areas of policy and practice are not adequately recognised.

In response, there have been calls to look to comparable policy areas such as gang‐related violence or larger‐scale militancy to derive insights relevant to countering radicalisation to violence (e.g., Baruch et al., 2018; Davies et al., 2017; Ris & Ernstorfer, 2017). This is because the processes by which people become involved in ideologically motivated and other forms of violence are considered similarly complex, and because efforts to address collective (e.g., Brantingham et al., 2021; Engel et al., 2013) and interpersonal (e.g., Gondolf, 2008) forms of violence use case management approaches.

Thus far, the insights from research on countering a broader range of violence for counter‐radicalisation interventions have not been fully exploited. Although some work has sought to derive lessons from other areas of practice (e.g., Davies et al., 2017), no research has yet systematically identified and applied the insights from research on case management interventions in the broader field of violence reduction to efforts to counter radicalisation.

This systematic review is therefore split into two parts that speak to two important gaps in the literature: first the need to systematically identify and assess the research on case management interventions seeking to counter radicalisation to violence; and second, to identify the insights from research on the broader field of violence prevention for counter‐radicalisation work.

Part I of the review examines the implementation and effectiveness of case management tools and approaches working to counter radicalisation to violence.
Part II of the review examines the implementation and effectiveness of case management tools and approaches working to counter other, non‐terrorism forms of violence.

Whilst focused on different phenomena, both parts of the review are underpinned by a specific conceptualisation of case management that is discussed in detail in Sections 2.2 and 2.3.

### 2.2 The intervention

Interventions to counter radicalisation to violence have developed across the world (Ucko, 2018). Often described as preventing or countering violent extremism (P/CVE) interventions, they are characterised by a diversity of methods that target different stages of the radicalisation process. These stages are often described in relation to the public health model of prevention which involves primary, secondary and tertiary intervention points (Bhui & Jones, 2017).

Primary interventions aim to address the root causes of extremism, seeking to develop societal and individual resilience to radicalisation through interventions targeted at the general population who are at the ‘pre‐risk stage’ (Elshimi, 2020, p. 229). Secondary interventions work with those considered ‘at risk’ of radicalisation aiming to reduce the risk of an individual becoming actively engaged in violent extremism and terrorism, whereas tertiary interventions work with those involved in terrorism and violent extremism, often once they have been convicted of an offence, and aim to support their disengagement from this activity (Elshimi, 2020).

Case management approaches are increasingly being used in secondary and tertiary interventions aiming to counter radicalisation to violence. For example, the UK's main government‐led secondary intervention, Channel, takes a case‐managed approach (HMG, 2020), whilst the Countering Violent Extremism Early Intervention Program (CVE‐EIP) (Cherney, 2022; Cherney & Belton, 2021a; Harris‐Hogan, 2020) and Proactive Integrated Support Model (PRISM) (Cherney & Belton, 2021b) in Australia use case management to coordinate efforts to support those considered at risk and those convicted of terrorism offences.

The case management approach is significant because it goes beyond specific kinds of intervention methods to structure the process through which services are delivered and monitored. Rather than focusing on, for example, mentoring, educational support, or ideological advice, looking across the case management process offers a wider perspective that pays attention to the different stages at which an individual is supported, from first being identified as in need of assistance through to their exit from the programme.

**Figure 1:**
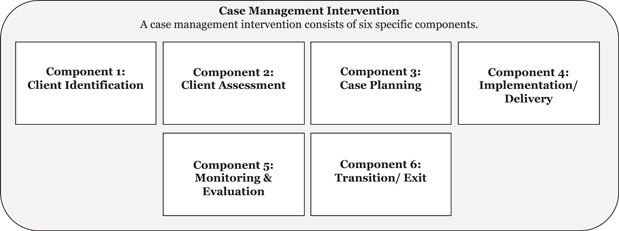
The intensive case management process (based on NCMN, 2009).

Case management interventions can take different forms, ranging from short‐term, less intensive ‘brokerage’ models where clients are connected to different kinds of support, through to more ‘assertive’ approaches which see case managers work with clients over the longer‐term (Lukersmith et al., 2016). Interventions targeting radicalisation to violence typically adopt more intensive models of case management which is typically understood to involve six stages (see Figure 1).

Although the specific features of case managed interventions can vary, these six stages are commonly described in guidance provided by professional organisations such as the Case Management Society UK (CMSUK) and Canada's National Case Management Network (NCMN) (CMSUK, 2009; NCMN, 2009). These stages structure the process by which an individual is identified, their needs are assessed, and an intervention to address those needs is planned and implemented. Case management also involves monitoring the client's progress until the point the intervention is assessed to have met their needs or achieved particular outcomes, before the final stage of transition out of the programme (NCMN, 2009; Ross et al., 2011). A key characteristic of case management is that it is client‐centred, as the following definition suggests:

[Case management is] a collaborative process which assesses, plans, implements, coordinates, monitors and evaluates the options and services required to meet an individual's health, care, educational and employment needs, using communication and available resources to promote quality cost effective outcomes. (CMSUK, 2009, p. 8)

Case management interventions involve the use of different tools relevant to each stage of the process, and can be delivered in ways which reflect different approaches. Specifying these tools and approaches helps to organise the knowledge of different aspects of the case management process and understand what influences the process and outcome of interventions. This is particularly helpful as, although some interventions are explicitly organised around the different stages of the case management process set out in Figure 1, many others use aspects of the case management process without organising or labelling it as ‘case management’. Nevertheless, insights are possible by looking at research on the tools and approaches that are used at each stage, which this review defines as follows:

– Case management tools: the processes or methods employed at each stage of the intervention. These include tools used to assess an individual's risk and needs, develop and deliver intervention plans, monitor their progress, and assess and support exit from the programme.
– Case management approaches: theories of change or intervention logics that inform how interventions are delivered. These can be implicit or explicit (White et al., 2021).

### 2.3 How the intervention might work

Most straightforwardly, case management aims to support positive outcomes by structuring the process of identifying suitable individuals, assessing and delivering support to address their needs, and managing their exit from the programme (Cherney & Belton, 2021a, 2021b). In this way, case management interventions try to interrupt pathways into radicalisation or divert people who are already involved in violent extremism and terrorism by structuring the process of identifying those at greater risk or need and providing tailored support to meet those needs in ways which reduce the risk of engaging in violent extremism and terrorism (Cherney & Belton, 2021a, 2021b).

A central feature of case management interventions is that they are tailored to the individual (CMSUK, 2009). This is why they are considered well suited to take account of the complex, individualised nature of radicalisation and deradicalisation processes (Cherney et al., 2020), and are applicable to a range of kinds of clients, who are engaged in secondary (e.g., Harris‐Hogan, 2020; Pettinger, 2020a, 2020b; Thompson & Leroux, 2022) or tertiary interventions (e.g., AEF, 2018; Schuurman & Bakker, 2016; van der Heide & Schuurman, 2018), or some combination of the two (e.g., Cherney & Belton, 2021a, 2021b). Interventions can focus solely on Islamist radicalisation (AEF, 2018; Schuurman & Bakker, 2016; van der Heide & Schuurman, 2018), be more oriented towards other ideologies such as the far‐right (e.g., Christensen, 2015), or may have a broader or unspecified ideological focus (e.g., Harris‐Hogan, 2020; Thompson & Leroux, 2022).

The delivery agents and contexts for case management interventions vary according to which sectors lead or are engaged in the intervention, and whether they are delivered in community (e.g., Harris‐Hogan, 2020; Pettinger, 2020a, 2020b; Thompson & Leroux, 2022) or correctional contexts (e.g., Cherney, 2018; Schuurman & Bakker, 2016; van der Heide & Schuurman, 2018). Most interventions are standalone programmes specifically designed for countering radicalisation to violence, however some providers integrate CVE into existing, broader, prevention work or other forms of psychosocial support (e.g., Raets, 2022; Thompson & Leroux, 2022). Geographically, interventions can have a national (e.g., AEF, 2018; Harris‐Hogan, 2020; Schuurman & Bakker, 2016; van der Heide & Schuurman, 2018) or a regional focus (e.g., Thompson & Leroux, 2022).

In trying to interpret how case management interventions are supposed to work, this review focuses on the process by which the tools and approaches are implemented, rather than the outcome of the specific services that are delivered through the intervention – for example, training or educational support. The way the intervention might work can therefore be broken down across the different stages of the case management process, as each stage plays a role in identifying, managing and reducing risks, and developing strengths so the individual is less likely to see terrorism as a route to meeting their needs.

#### 2.3.1 Identification

The case management process begins once potential clients in need of support have been identified. In secondary interventions, this identification process typically involves identifying particular patterns of risks and needs considered likely to indicate an elevated risk of involvement in terrorism. In tertiary interventions, an individual's involvement in terrorism or violent extremism has typically already been recognised, for example through a terrorism conviction.

A range of methods may be used to refer clients into interventions. Costa et al. (2021) set out a typology of three different mechanisms: (1) Active; (2) Passive; and (3) Mediated. The active approach involves clients being referred to interventions by front‐line professionals from different sectors (e.g., police, education, healthcare, etc.). This is seen in the UK's approach to secondary interventions (Pettinger, 2020a, 2020b; Weeks, 2018), although similar methods are used in secondary and tertiary interventions in Australia (e.g., Cherney & Belton, 2021a, 2021b; Harris‐Hogan, 2020) and the Netherlands (AEF, 2018; Eijkman & Roodnat, 2017). This type of active approach can include mechanisms through which family members, friends, or community members refer individuals they are concerned about into programmes, or raise concerns about potential radicalisation to relevant front‐line professionals or agencies (e.g., Thomas et al., 2020). Secondary and tertiary interventions may also use a more passive approach, whereby individuals self‐refer into interventions (e.g., Christensen, 2015). And finally, whilst less common, secondary interventions might also incorporate a mediated approach, where families can provide individuals with information about a relevant programme, or even physically take them to an intervention provider (e.g., Costa et al., 2021).

A significant proportion of individuals identified as being potentially in need of counter‐radicalisation support through the active or mediated approach never formally enter the case management process. For example, statistics from the UK's Channel programme indicate that 23% of the 6406 referrals made in the year ending 31st March 2022 were formally assessed by a multi‐agency ‘Channel panel’ (HM Government, 2023). As a result, research that focused on the methods by which members of the public and frontline professionals identify perceived indicators of risk, and decide when to refer into interventions was excluded from this review. Instead, we define the client identification stage as the period during which counter‐radicalisation practitioners make preliminary assessments relating to the potential eligibility of individual clients, and determine whether cases should progress to the client assessment stage. In secondary interventions these assessments may relate to screening out obviously inappropriate, misguided, or ‘spurious’ referrals that do not warrant further action (Lewis, 2021), as well as identifying referrals relating to individuals who are already the focus of a criminal investigation or other, harder forms of intervention (Cherney & Belton, 2021b).

#### 2.3.2 Client assessment

The identification process is typically followed by a formal assessment process. In secondary interventions, the assessment aims to differentiate between those who pose a radicalisation risk, those who do not, and others who might have needs unconnected to terrorism and who could benefit from signposting to alternative forms of support, such as mental health provision. Those ineligible for secondary interventions may fall below the threshold for radicalisation risk (Pettinger, 2020a, 2020b), and may be referred to other agencies or forms of support to address other needs identified through the assessment, or be considered too high risk and in need of harder forms of intervention (Cherney & Belton, 2021b). In tertiary interventions, the assessment process is primarily used to identify client‐specific intervention goals and the relevant forms of support needed to support disengagement from violent extremism, and reduce risk (Cherney & Belton, 2021b). This aspect of case management is examined in detail below.

Eligibility screening can be conducted in different ways and involve different actors. This may include those who are involved in delivering the intervention – on the basis this has the potential to enable them to ‘build rapport and assess their clients’ (van der Heide & Schuurman, 2018, p. 205) – as well as those who will not go on to work with the individual (AEF, 2018; Christensen, 2015). Assessments can be conducted in person, by one or two individuals (AEF, 2018; Christensen, 2015), or a specific team to assess individual cases before their discussion at a multi‐agency case conference (Inspectorate of Justice & Security, 2017, p. 24; also Eijkman & Roodnat, 2017; Vandaele et al., 2022a). However, it is more common for eligibility to be determined by multi‐agency case conferences before intervention staff engage with the client.

Case conferences draw on information from different partners and assess this information to determine whether clients are eligible for counter‐radicalisation support; identify their specific needs; and tailor intervention plans. They are a common feature of interventions across the world, including in the UK (Pettinger, 2020a, 2020b; Weeks, 2018); the Netherlands (AEF, 2018; Hardyns et al., 2022); Canada (Thompson & Leroux, 2022); and Australia (Cherney, 2022). The way case conferences operate varies across different programmes (Vandaele et al., 2022a). For example, some interventions may discuss deidentified cases when assessing individuals (Thompson & Leroux, 2022), in contrast to many interventions, which do not appear to de‐identify potential clients. In some contexts, individuals may be aware they are being discussed at conferences, but in others, they may not (Vandaele et al., 2022a).

Multi‐agency interventions often appoint a dedicated case manager, or in some interventions, multiple case managers (Cherney et al., 2022; van der Heide & Schuurman, 2018). Case managers are typically assigned overall responsibility for managing clients throughout the case management process. They help develop the intervention plan and identify relevant services and organisations able to meet the client's needs, as well as monitoring their progress (AEF, 2018). Some interventions will appoint a dedicated mentor (or mentors) who deliver support to clients alongside facilitating other aspects of the case management process, such as client assessment and case planning (e.g., Christensen, 2015; Fisher et al., 2020).

Risk and needs assessment tools can help inform the assessment process (Lloyd & Dean, 2015), although these tools are not always used in this way (Barracosa & March, 2022; Costa et al., 2021). These tools can be general or specialised for use in terrorism cases. Specialised tools include the Vulnerability Assessment Framework (VAF) (Pettinger, 2020a, 2020b); the Violent Extremism Risk Assessment, Version 2 Revised (VERA‐2R) (Raets, 2022; van der Heide & Schuurman, 2018); and the Terrorist Radicalization Assessment Protocol (TRAP‐18) (Corner & Pyszora, 2022; Raets, 2022). The majority of specialist tools use a Structured Professional Judgement (SPJ) approach, which ‘relies on the discretion of the assessor whilst providing a basic, empirically informed structure to help guide their decision‐making’ (Copeland & Marsden, 2020, p. 7). Specialised tools that use a more structured, actuarial approach are also in use internationally (e.g., Raets, 2022), as are general, non‐specialist tools, such as the Level of Service Inventory Revised (LSI‐R) (Cherney, 2021; Inspector of Custodial Services NSW, 2018). A range of bespoke specialist tools may also be used. This includes tools that are specific to individual interventions, or that are used only in specific regions such as the ‘Radix’ tool developed by one Belgian municipality (Costa et al., 2021; Raets, 2022), and tools that are used to assess specific cohorts, such as youth (Barracosa & March, 2022). All of these tools seek to accurately assess the risk an individual poses and inform decisions about whether and what kind of support they should receive.

#### 2.3.3 Case planning

Case management interventions are operationalised through the delivery of a case plan. The development of the case plan is informed by the assessment process. Some interventions will use risk and needs assessment tools to inform intervention planning (Lloyd, 2019). The basic approach to developing client‐specific case plans typically involves multi‐agency partners discussing the support needs of each client and designing a tailored intervention plan that is designed to target each client's needs, and deliver specific intervention goals (Cherney & Belton, 2021b), through provision of a tailored set of services (Ellis et al., 2022).

Case managers or dedicated mentors often play an important role in developing tailored intervention plans (e.g., AEF, 2018; Inspectorate of Justice & Security, 2017) and monitoring the individual's progress to determine if the case plan is adequately meeting their needs (Harris‐Hogan, 2020). However, the specific approach used may vary across interventions. For example, in some settings, the client and coach/mentor will co‐design an action plan, which will then be presented to, and assessed by, a multi‐agency case conference (AEF, 2018).

In many cases, the case plan structures the process by which services are delivered, for example, sequencing activities in ways which take account of the individual's learning style or needs (Cherney & Belton, 2021a). Case plans developed at the outset of the case management process are unlikely to remain static and may be reviewed and updated regularly to account for an individual's changing circumstances and needs (Disley et al., 2016; Thompson & Leroux, 2022), and respond to any emerging challenges (Cherney, 2022; Vandaele et al., 2022a).

#### 2.3.4 Delivery and implementation

Case management interventions involve the delivery of tailored intervention plans which deploy services considered likely to meet the individual's needs and reduce their risks (Cherney & Belton, 2021a). A diversity of services are typically available including education; employment; lifestyle; psychological help; family provision; and religious or ideological advice, as well as more specific types of support such as music programmes; childcare services; speech therapy; or referral to mental health services (Cherney & Belton, 2021a; Raets, 2022). In some settings, such as the Netherlands, the range of services is much broader, with up to 50 different types of intervention available to clients (AEF, 2018), which are under constant review and expansion (Eijkman & Roodnat, 2017).

The quality and delivery of interventions are supported in a variety of ways. Some intervention programmes – such as secondary and tertiary interventions in the UK – have developed mentor selection and accreditation processes for intervention providers (Pettinger, 2020a, 2020b; Thornton & Bouhana, 2019; Weeks, 2018). In other settings, competency is developed through an ongoing process. For example, in EXIT Sweden, former clients may become ‘client‐coaches’, who support clients whilst continuing to work on their own rehabilitation (Christensen, 2015). Whereas the Team TER reintegration intervention specifically set out to develop a group of specialists from the Dutch Probation Service who would apply their knowledge of working with other types of offenders to this programme, whilst gaining specialist expertise working with terrorist and violent extremist offenders (Schuurman & Bakker, 2016). A similar emphasis on ‘learning by doing’ is used in other interventions in the Netherlands (AEF, 2018; Eijkman & Roodnat, 2017). The aim of these methods is to develop a body of skilled professionals able to deliver and support the case management process.

#### 2.3.5 Monitoring and evaluation

A range of approaches are available to monitor and evaluate an individual's progress through the case management process. These include the use of multi‐agency case conferences which meet to review ongoing cases (e.g., Thompson & Leroux, 2022) and formalised assessment tools. For example, several interventions in Australia use information drawn from the Radar tool and from other sources such as case reviews to assess and track client progress (Cherney, 2022; Cherney & Belton, 2021b; Harris‐Hogan, 2020). Assessments are typically made against their original intervention plan and can be undertaken independently of the client, or as is the case in the Netherlands, collaboratively, so the coach and client assess progress against their action plan, and identify areas that might need additional attention (e.g., AEF, 2018).

As part of a range of different kinds of qualitative data, case files and case notes are used to assess progress and can be used to record a mix of factual information, such as recording that a client attended a session, as well as more subjective client feedback and assessments (Cherney & Belton, 2021a, p. 15; 2021b, p. 630). This process can involve the collation of a range of data collected at different points during the case management process, from different sources, and using different methods, including:

[…] qualitative inputs relating to client background information, risk and needs assessments, client intervention goals, dated observations about intervention staff/service provider engagements with clients, service provider and family members correspondence relating to client appointments, activities and participation, psychologist/counsellor feedback, nature and reason for police contact, court documents and forms of open‐source data.

(Cherney & Belton, 2021a, p. 4)

#### 2.3.6 Transition and exit

The decision of when an individual exits a case management intervention is largely determined by their circumstances and differs between secondary and tertiary interventions. There are few specific timeframes for secondary interventions (AEF, 2018; Costa et al., 2021), whereas when someone has been convicted, their involvement in an intervention is likely to be shaped by the length of their sentence or conditions of their parole. For example, Cherney (2020) notes that PRISM staff may begin the process of engaging clients 2 years before their earliest possible release date. When the individual's sentence has been served, they will typically exit the programme, although in some cases there are opportunities for individuals to continue to receive support after this point should they wish to, or the parole service can request assistance from the original intervention provider when managing a client in the community (Cherney, 2018; Marsden, 2017).

Where the intervention ends before a prisoner's sentence has been completed, or when someone is engaged in a secondary intervention, they are typically assessed to determine if their risk has reduced, and their needs have been met in a way which is consistent with their individualised intervention goals. For example, Khalil et al. (2019) note that exit from the Serendi rehabilitation centre in Somalia is dependent on the individual meeting ‘agreed and personalised rehabilitation objectives relating to family connections, education, vocational training, security issues in the locations of reintegration, and so on’ (p. 4). However, some criteria are more generic. For example, Vandaele et al. (2022a) note that cases in Germany, the Netherlands, and Belgium are closed ‘if no new events of concern occurred’ (p. 71).

The level of aftercare differs across contexts (Costa et al., 2021). Some interventions, such as Forsa in the Netherlands, Serendi in Somalia, and PRISM in Australia, provide ongoing support (AEF, 2018; Cherney, 2020; Khalil et al., 2019). Others including Team TER (van der Heide & Schuurman, 2018), and EXIT Sweden (Christensen, 2015) do not, although intervention staff may choose to stay in contact with former clients when no formal aftercare is offered. The approach to aftercare also varies, some contact former clients periodically or when information on them needs to be updated; others refer clients to other organisations/partners; and some have a structured follow‐up strategy (Costa et al., 2021). These processes aim to provide ongoing support for the individual's reintegration, and to monitor their progress outside the formal case management process.

### 2.4 Why it is important to do the review

The complexity of radicalisation and deradicalisation processes has led researchers, and policymakers and practitioners, to seek ever more comprehensive routes to countering radicalisation to violence. Increasingly, this has drawn on the principles of case management to structure the process of supporting individuals at risk, or already involved in violent extremism (Cherney & Belton, 2021a). At the same time, scrutiny of counter‐radicalisation interventions has increased, in particular in the wake of apparent failures of case management systems, when individuals enroled in these programmes have gone on to carry out terrorist attacks (Cherney et al., 2022; Clubb et al., 2021).

Inquiries following high‐profile attacks, such as by Usman Khan in London, have raised questions regarding the appropriateness of the tools and approaches used to manage terrorism offenders (Cherney et al., 2022; Lucraft, 2021). However, although the research in this area is growing, it has not yet been systematically synthesised. This is partly because the field is relatively new and the evidence base is still developing (Hassan et al., 2021a, 2021b). It is also because research is dispersed across multiple disciplines; typically focuses on specific aspects of the case management process (e.g., risk assessment or case planning); and with some exceptions (e.g., Cherney & Belton, 2021a, 2021b), rarely explicitly uses the term ‘case management’. In addition, the assumptions that underpin counter‐radicalisation interventions, which are typically understood in relation to logic models or theories of change, are rarely made explicit and/or assessed empirically (Lewis et al., 2020). This hampers evidence synthesis and makes it harder to develop an overall picture of what informs the process and outcome of interventions.

It is also important to learn what influences the implementation of case management interventions. Thus far, there have been no efforts to systematically synthesise research on how case management tools and approaches are used in practice. Without a better understanding of what influences whether, for example, risk assessment tools feed into case planning processes, or if monitoring of individual cases is informed by intervention plans, it is hard to determine what facilitates or creates barriers to implementation, or what contextual conditions, or moderators, shape how interventions are delivered.

Because of the limitations of the research on case management interventions in this field, which has yet to develop a robust evaluation culture (Baruch et al., 2018) or agree a set of progress and outcome measures (Pistone et al., 2019), there have been calls to look to fields with a better developed evidence base (Lewis et al., 2020). Research in the wider field of non‐terrorism related violence prevention has important insights for counter‐radicalisation policy and practice. Both because it has a longer history of evaluation (Davies et al., 2017), and because it has drawn on intensive case management models to structure interventions (Brantingham et al., 2021). However, the implications of broader violence reduction or prevention interventions for counter‐radicalisation work have yet to be fully systematised or exploited.

Given the high cost of failure, there is an unmet need to understand whether the tools and approaches used in case management interventions help counter radicalisation to violence; understand what informs how interventions are implemented in practice; and identify relevant learning from comparable fields. These issues are relevant not only for researchers, but also for policymakers and practitioners who will benefit from a synthesis of what is a rapidly expanding and increasingly dispersed evidence base. By understanding the current state of the research on whether case management interventions help counter radicalisation to violence, what informs how they are implemented, and what learning is possible from other fields, the review will support decision making and provide a foundation to inform the design and delivery of case management interventions. It will do this by first assessing the research to determine the strength of the evidence relating to the effectiveness of case management interventions; second, qualitatively synthesising the research on what facilitates or generates barriers to programme implementation, and how different contexts, or moderators shape these processes; third, synthesising the findings of existing systematic reviews of research on related fields of violence prevention; and finally, examining the transferability of evidence from comparable fields to interventions seeking to counter radicalisation to violence.

## 3 OBJECTIVES

### 3.1 Part I: Countering radicalisation to violence

The first part of the review aims to examine the research on case management tools and approaches to determine if they are effective in countering radicalisation to violence, either by supporting primary outcomes indicating diversion or disengagement from violent extremism, desistance or deradicalisation, or enabling secondary outcomes such as measures of client engagement or motivation (Objective 1: effectiveness). The review further aims to assess whether case management tools and approaches are implemented as intended (Objective 2a: implementation), and understand what explains how different case management tools and approaches are implemented, by examining what facilitates, or creates barriers to implementation, and learning whether contextual conditions, or moderators, influence how case management interventions are implemented in practice (Objective 2b: implementation).

### 3.2 Part II: Countering other forms of violence

The second part of the review aims to examine existing systematic reviews of research on case management tools and approaches seeking to counter other forms of violence to assess whether they are effective at countering interpersonal or collective forms of violence, either by supporting primary outcomes including desistance from violence or reducing the risk of violence or violent recidivism, or secondary outcomes, such as attitudinal or behavioural changes which support desistance (Objective 3). The review will also examine reviews seeking to understand whether case management tools and approaches are implemented as intended (Objective 4a), and what influences how they are implemented, including facilitators, barriers, and moderators (Objective 4b); and assess the transferability of tools and approaches used in wider violence prevention work to counter‐radicalisation interventions (Objective 5).

## 4 REVIEW PART I – COUNTERING RADICALISATION TO VIOLENCE

### 4.1 Methods

#### 4.1.1 Criteria for considering studies for Part I

##### Types of studies

The two objectives for Part I of the review focus on the same question of the role of case management interventions in responding to the problem of radicalisation to violence. However, the inclusion criteria for Objective 1 and Objective 2 rely on different criteria relating to research design and outcome measures. These are considered separately below.

###### Types of study designs for review of effectiveness (Objective 1)

Only quantitative studies were eligible for inclusion in the review of effectiveness of case management tools and approaches (Objective 1). These studies had to employ a randomised experimental (i.e., Randomised Control Trials) or stronger quasi‐experimental research design. Across both types of research design, comparator or control group conditions could include treatment‐as‐usual; no treatment; and alternative treatment. Robust quasi‐experimental designs had to be in line with the criteria set out by the UK government's Magenta Book (HM Treasury, 2020) and previous Campbell reviews (e.g., Mazerolle et al., 2020), and included the following designs:

‐ Cross‐over designs.
‐ Regression discontinuity designs.
‐ Designs using multivariate controls (e.g., multiple regression).
‐ Matched control group designs.
‐ Unmatched control group designs where the control group has face validity.
‐ Unmatched control group designs allowing for difference‐in‐difference analysis.
‐ Time‐series designs.

###### Types of study designs for review of implementation (Objective 2)

Both quantitative and qualitative studies were eligible for inclusion in the assessment of implementation (Objective 2). Eligible quantitative studies included research designs using randomised experimental and strong quasi‐experimental designs in line with the criteria for Objective 1 (set out above). Studies employing other quasi‐experimental or non‐experimental designs were also eligible for inclusion. These were analysed alongside the qualitative and mixed methods research that was incorporated in this aspect of the review.

To be included, qualitative and mixed methods research had to report on empirical findings on tools or approaches used in case management interventions which were informed by primary data, such as interviews or focus groups, or the secondary analysis of primary data. Opinion pieces, purely theoretical studies, and literature reviews were excluded.

Although qualitative research cannot support causal claims about effectiveness or implementation, it holds important insights into what facilitates and creates barriers to implementing case management interventions. Empirical research that uses interviews, focus groups, or observational research methods provide crucial insights into the factors that shape implementation processes and the inclusion of such research provides the opportunity to capture ‘the broadest range of evidence that assesses the reasons for [an intervention's] implementation success or failure’ (Higginson et al., 2015, p. 22). For these reasons, qualitative research was eligible for inclusion to address Objective 2.

##### Types of participants

There were no geographical exclusion criteria for either the review of effectiveness (Objective 1) or the review of implementation (Objective 2). There were also no demographic exclusion criteria. Studies drawing on data from participants of any age, gender, ethnicity, religion, or ideological perspective (e.g., right‐wing; Islamist, left‐wing, etc.) were eligible for inclusion. Empirical research which used data drawn from practitioners, stakeholders in any of the multiple agencies that are involved in implementing case management interventions, and clients of those interventions were included in the review.

##### Types of interventions

Studies for both Objective 1 (effectiveness) and Objective 2 (implementation) had to report on tools or approaches used in case management interventions aiming to counter radicalisation to violence by working directly with those at risk of engaging in, or who have been engaged in violent extremism as described in Section 2.2. Although there is no single model of case management, these interventions are typically understood as being made up of a series of stages. Each stage makes use of a range of tools to support client identification, assessment, case planning, implementation, monitoring and evaluation, and transition and exit. These interventions are also informed by different approaches, or theories of change, which inform how interventions are delivered.

To be eligible for inclusion, studies had to report on tools that were used at one or more stages of the intervention process or examine the approaches or intervention logics (see Section 2.2 for the definition of approaches used in the review) that underpinned the intervention. To capture the range of tools and approaches that are used in interventions seeking to counter radicalisation to violence, the review did not limit itself to studies that explicitly used the case management framework. To be included, studies had to analyse a tool or approach which:

(1) Focused on individuals rather than communities or collectives.
(2) Aimed to counter radicalisation to violence amongst those who had been identified as at risk of involvement in violent extremism and/or those who had been involved with or convicted for engagement in violent extremism (i.e., secondary or tertiary interventions).
(3) Was employed during one or more stages of the case management process described in Section 2.2.
(4) Involved a tailored approach which informed or enabled an individualised intervention seeking to support the move away from violent extremism.

##### Types of outcome measures

###### Outcomes relevant to effectiveness of interventions (Objective 1)

Two types of outcome measure were used for the review of effectiveness (Objective 1): primary outcomes that reflected measures of risk reduction, disengagement, or deradicalisation; and secondary outcomes which demonstrated the impact of specific tools or approaches on progress towards primary outcomes.

Primary outcomes relate to the overarching aims of counter‐radicalisation interventions and provide insights into whether the goal of preventing engagement (secondary interventions), or supporting the disengagement and deradicalisation of an individual from violent extremism (tertiary interventions) has been met. Although definitions are contested, deradicalisation is typically understood as attitudinal change that reflects a rejection of extremist ideas and the legitimacy of violence (Horgan, 2009). Disengagement on the other hand, is generally understood as behavioural change that sees someone cease involvement in violent extremism whilst not necessarily rejecting the ideas that support it (Horgan, 2009).

The metrics by which these outcomes can be measured are a source of debate (Lewis, Copeland & Marsden, 2020) and there are no agreed metrics of success for counter‐radicalisation interventions (Baruch et al., 2018). For this review of effectiveness (Objective 1), the first type of outcome measure focused on higher order outcomes that indicate that an individual's risk of engagement has reduced (secondary interventions), or that an individual has either deradicalised or disengaged according to assessments of recidivism or re‐engagement (tertiary interventions). The data underpinning these assessments could be derived from, for example, arrest, prosecution, sentencing and other relevant criminal justice data; interviews or official reporting derived from those involved in the case management process; risk assessments; and/or case notes.

Secondary outcomes are a broader category of measure and reflect lower‐order objectives which can help interpret progress towards the ultimate aim of preventing engagement, or promoting deradicalisation and disengagement. These outcomes relate to the impact of specific tools and approaches that are used across the case management process and their role in enabling or undermining progress towards these goals. Importantly, these assessments are not focused on the impact of specific interventions, such as theological mentoring or the provision of educational support, as these are covered in existing reviews (e.g., Hassan et al., 2021a, 2021b). The focus for this review is on the impact of the tools and approaches that support the delivery of the case management intervention.

To assess whether case management interventions help people move towards these goals, studies which reported the outcome of risk assessments which interpret – and sometimes track – whether risk and/or protective factors have changed in line with intervention goals were eligible for inclusion. A range of risk assessment measures have been developed which seek to assess change across risk and protective factors and are often used to inform intervention planning (Lloyd, 2019). Some of these include:

‐ Extremism Risk Guidance (ERG22+): Assesses risk through 22 indicators that are linked to three domains: engagement with a group, cause or ideology; intent to cause harm; and capability to cause harm (Lloyd & Dean, 2015).
‐ Violent Extremism Risk Assessment Version 2 Revised (VERA‐2R): Measures risks against a series of indicators which cover attitudes and ideology; history and capacity; commitment and motivation; protective factors; and individual criminal, personal and psychiatric history (Pressman, 2009).
‐ Terrorist Radicalisation Assessment Protocol (TRAP‐18): Assesses proximate and distal factors that indicate risk and threat with a focus on lone‐actor terrorism (Meloy, 2018).

To understand the impact of case management tools and approaches, studies were eligible if they reported quantitative evidence which evaluated the effect of one or more tools or approaches. Although none were identified, this would have included studies which assessed both the effectiveness of overall approaches including risk and strengths‐based approaches, and the impact of specific tools on different stages of the case management process.

Eligible studies reporting on specific tools could record the outcome of identification and referral processes, for example by assessing how many individuals were accurately identified and referred; risk assessment tools, by determining the impact of effective risk assessment processes; case planning, by assessing whether certain tools used to support case planning were more or less effective than others; the outcome of case planning processes and whether, for example, they identified the most appropriate interventions in individual cases; delivery processes, assessed by the extent to which they helped to support engagement and participation with interventions, or reduced levels of drop out; the relative impact of different monitoring and evaluation regimes; and tools to support transition and exit, for example, by assessing the relative impact of different ways of assessing needs and referring on to additional forms of support at the end of the case management process.

###### Outcomes relevant to implementation of interventions (Objective 2)

In line with Mazerolle et al. (2021), no specific outcome measures were necessary for studies to be eligible for inclusion in the assessment of implementation (Objective 2). Instead, all qualitative, quantitative and mixed methods empirical research which addressed implementation factors were eligible. Implementation factors were defined as ‘actions or actors necessary to successfully install and maintain an intervention’ (Cherney et al., 2020, p. 16) and were understood in relation to facilitators, which supported the implementation of case management intervention tools and approaches, and barriers which had the potential to undermine implementation.

A wide range of factors have the potential to act as facilitators and barriers to implementation. To give some examples in relation to tools, these might include factors which influence the identification and referral process such as the capacity of the organisations tasked with identifying relevant individuals (e.g., Becker et al., 2014). Factors relevant to implementing assessment processes could include the suitability of the criteria used to inform risk assessment tools (e.g., Fisher et al., 2020). In regard to case planning, implementation might be impacted by the ways in which case conferences are managed (Vandaele et al., 2022b), whilst practitioner characteristics might shape how interventions are delivered (e.g., Orban, 2019), and the quality of data capture and management processes may influence the implementation of monitoring and evaluation processes (e.g., Cherney & Belton, 2021a). Finally, transition and exit might be facilitated by good inter‐agency working (Cherney, 2020), or undermined due to difficulties monitoring individuals on release (Stern et al., 2023).

As well as studies which analysed facilitators and barriers, the review also included research which reported data relevant to moderators, or the ‘contextual conditions’ or ‘features of the people or places that are the target for intervention’ (Cherney et al., 2020, p. 15), and research that analysed whether interventions were being implemented as intended. Moderators can include the features of the delivery context, for example, whether an intervention is run in prisons or in the community; the characteristics of individual clients, informed by their demographics, or social and cultural context; the nature of the delivery agents, for instance whether they are civil society organisations or correctional staff; different organisational mandates; and the philosophy of different intervention providers and funders.

Although qualitative research does not treat outcomes in the same way as quantitative research, and may not refer to outcomes in its analysis, it remains valid for interpreting what shapes implementation processes (Mazerolle et al., 2021). Rather than focusing on outcomes, qualitative research typically discusses thematic features of data drawn from a range of sources able to inform broader insights into the process of implementing interventions. To capture these insights, this review included empirical research that analysed factors which facilitated or generated barriers to the implementation of tools and approaches, and the contextual conditions that shaped how interventions were implemented across all stages of the case management process.

##### Duration of follow‐up

No restrictions were placed on the length of follow up for either the review of effectiveness (Objective 1) or the review of implementation (Objective 2).

##### Types of settings

No geographic or setting‐based restrictions were used to exclude studies. Research conducted in any country, and reported on in the languages covered by the review (English, French, German, Swedish, Danish, Norwegian, and Russian) was eligible for inclusion.

#### 4.1.2 Search methods for identification of studies

The search process for English language material involved six stages that sought to identify relevant academic and grey literature research.

1. Identification and piloting of search terms.
2. Targeted search term searches of academic databases.
3. Hand searches of key journals, research outputs of relevant research institutions/professional agencies, and clinical trial repositories.
4. A review of studies cited in key evidence synthesis papers.
5. Consulting members of the research team and advisory board to identify studies.
6. Forward and backward citation searching of studies identified at Stages 1–5.

##### Identification and piloting of search terms

Search terms were developed by the research team and piloted in May 2021 using PsycNet as a test database to determine the scale of the literature and the sensitivity of the search terms. Search terms were differentiated according to two search domains:

‐ Problem (Any Field: extremis* OR Any Field: terror* OR Any Field: radical*) AND
‐ Intervention (Any Field: prevent* OR Any Field: treat* OR Any Field: interven*)

This process led to the team refining the search strategy searches to reduce the number of irrelevant and/or non‐empirical studies. Informed by feedback from the Campbell Crime and Justice Editorial Group, a further piloting process in May 2022 led to a search strategy informed by three domains:

‐ Problem: Search terms relevant to violent extremism and its synonyms (radicali*, extremis*, terroris*); or specific ideologies (e.g., ‘far‐right’; ‘white supremacis*’).
*AND*
‐ Intervention: Search terms describing synonyms for *interventions* (e.g., ‘interven*’; ‘program*’, etc.) and *tools* (e.g., ‘tool*’; ‘instrument*’); and the different *stages* of the case management process (e.g., ‘refer*’; ‘assess*’);
*AND*
‐ Outcomes: Search terms relating to primary *outcomes* of prevention (e.g., ‘prevent*’); and desistance (e.g., ‘disengage*’).

The full list of search terms is available in Supporting Information: Appendix I.

##### Electronic searches

A search of electronic databases was carried out by The Campbell Collaboration Crime and Justice Coordinating Editor and Information Specialist (Elizabeth Eggins) in August 2022. The databases are detailed in Table 1. The databases were categorised as either principal of supplementary sources according to the functionality and replicability of their search functions. This approach was informed by the findings of Gusenbauer and Haddaway's (2020) analysis of 28 academic search systems for systematic reviews. Principal resources are characterised by a more comprehensive search capability which supports the use of different combinations of search terms across multiple search fields. Supplementary resources have more limited search functionality and typically do not allow for fully comprehensive search term searching, or the easy replication of searches. The search syntax, tailored for each database, is available in Supporting Information: Appendix I. The timeframe for the searches began in January 2000, as this marks the point at which radicalisation, and subsequently, deradicalisation, began to emerge as a feature of policy discourse (Neumann, 2013).

**Table 1:** Platforms/providers included in review.

Platform/provider	Specific databases searched (if applicable)	Search fields^a^	Resource type
Ovid	PsycInfo	ab,hw,id,mh,ot,ti.	Principal
Elsevier	Scopus	TITLE‐ABS‐KEY	Principal
Web of Science	Book Citation Index (Social Sciences & Humanities); Social Sciences Citation Index; Arts & Humanities Citation Index; Emerging Sources Citation Index; Conference Proceedings Citation Index (Social Sciences & Humanities); Medline	Topic	Principal
EBSCO*host*	Criminal Justice Abstracts	Title Abstract Keywords Subject	Principal
ProQuest	International Bibliography of the Social Sciences	ti, ab, mainsubject	Principal
ProQuest	Sociological Abstracts	ti, ab, if	Principal
Informit	CINCH: Australian Criminology Database	All Fields	Supplementary
ProQuest	Dissertations and Theses Global	ti, ab, mainsubject, diskw	Principal
EThOS (Dissertations)	N/A	All fields	Supplementary
Directory of Open Access Journals (DOAJ)	N/A	All fields	Supplementary

^a^ Although preferable to search across all search fields in every database, the number of results returned from larger databases becomes too large to sift. These search fields are therefore tailored to the size of each database.

##### Searching other resources

In addition to searching electronic academic databases, we carried out searches of relevant websites and research repositories to identify grey literature. The search process for these sources was tailored to the functionality of the website. For example, for websites that were specific to the field of countering radicalisation to violence (e.g., Hedayah), we searched all publications listed on the website. For others with a broader focus (e.g., RAND), we searched all publications listed under relevant sections/themes (e.g., countering violent extremism).

The list of websites used to identify grey literature sources is in Table 2.

**Table 2:** Grey literature sources.

Source	Description
*Institute for Strategic Dialogue (ISD)* https://www.isdglobal.org/	Research centre
*RAND* https://www.rand.org/	Research centre
*Royal United Services Institute (RUSI):* https://rusi.org/	Research centre
*Hedayah* https://www.hedayahcenter.org	Research centre
*International Centre for Counter‐Terrorism (ICCT)* https://icct.nl/	Research centre
*Resolve Network* https://www.resolvenet.org/	Research centre
*Global Center on Cooperative Security* https://www.globalcenter.org/	Research centre
*International Centre for the Study of Radicalisation (ICSR):* https://icsr.info/	Research centre
*Centre for Research and Evidence on Security Threats (CREST)* https://crestresearch.ac.uk	Research centre
*National Consortium for the Study of Terrorism and Responses to Terrorism (START)* https://www.start.umd.edu/	Research centre
*IMPACT Europe* http://impacteurope.eu/	Research repository
*CT‐MORSE* https://ct-morse.eu/	Research repository
*National Criminal Justice Reference Service (NCJRS)* https://www.ojp.gov	Research repository
*Radicalisation Research* https://www.radicalisationresearch.org	Research repository
*VOX‐Pol* https://www.voxpol.eu	Research repository
*Crime Solutions* https://crimesolutions.ojp.gov	Research repository
*College of Policing Crime Reduction Toolkit* https://www.college.police.uk/research/crime-reduction-toolkit	Research repository
*Global Policing Database* https://gpd.uq.edu.au/s/gpd/page/about	Research repository
*Europol* https://www.europol.europa.eu/	Government agency
*Public Safety Canada* https://www.publicsafety.gc.ca	Government agency
*Department for International Development:* *Research for Development* https://www.gov.uk/research-for-development-outputs	Government agency
*Radicalisation Awareness Network* https://ec.europa.eu/home-affairs/networks/radicalisation-awareness-network-ran_en	Government agency

Hand searches of key journals were undertaken to identify recently published material that may not have been catalogued in the academic databases, and to ensure no relevant studies had been missed in the main search. This process involved searching all volumes and issues of each journal published since 2000, and, where relevant, any online first articles that had yet to be included in a published issue. The journals covered by these searches are listed in Table 3.

**Table 3:** Key journals.

Journal name
*Terrorism and Political Violence*
*Studies in Conflict & Terrorism*
*Behavioral Sciences of Terrorism and Political Aggression*
*Critical Studies on Terrorism*
*Journal for Deradicalization*
*Perspectives on Terrorism*
*International Journal of Conflict & Violence*
*Dynamics of Asymmetric Conflict*
*Journal of Policing, Intelligence & Counter Terrorism*
*Journal of Threat Assessment and Management*

In addition, we ran a separate search of clinical trial registries identified by Fay and Eggins (2019) using search terms relating to the problem of countering radicalisation to violence (e.g., extremis*; terroris*, etc). These registries searched are listed in Table 4. We also contacted our advisory board and experts in the field of radicalisation and counter‐radicalisation with a request to identify research relevant to our review. Hand searches of the bibliographies of existing synthesis studies were carried out to determine if they contained relevant research.

**Table 4:** Clinical trial registries.

Source
Australian and New Zealand Clinical Trials Registry
ClinicalTrials.gov
Clinical Trials Results
Cochrane Central Register of Controlled Trials (CENTRAL)
ISRCTN Registry (controlled-trials.com)
NIH RePORTER
Trials Register of Promoting Health Interventions (TRoPHI)
Unreported Trials Register
UK Clinical Research Network (UKCRN Study Portfolio)
WHO International Clinical Trials Registry Platform

These studies are detailed in Table 5 and were identified by the research team as the most comprehensive reviews of literature on countering radicalisation to violence. Finally, forward and backward citation searches of studies identified through the search process were carried out using Google Scholar and by searching bibliographies of included studies.

**Table 5:** Evidence synthesis studies.

Source
Davies, M., Warnes, R. & Hofman, J. (2017). *Exploring the transferability and applicability of gang evaluation methodologies to counter‐violent radicalisation*. Cambridge: RAND Europe.
Feddes, A. & Gallucci, M. (2015). A literature review on methodology used in evaluating effects of preventive and de‐radicalisation interventions. *Journal for Deradicalization*, 5, 1–27.
Hassan, G., Brouillette‐Alarie, S., Ousman, S., Kilinc, D., Savard, É. L., Varela, W., Lavoie, L., Fetiu, A., Harris‐Hogan, S., Borokhovski, E., Pickup, D., Madriaza, P., Rousseau, C., Thompson, S. K., McCoy, J., Venkatesh, V., Boivin, M., Srimathi Narayana, M., Morin, D., & the CPN‐PREV team. (2021a). *A systematic review on the outcomes of primary and secondary prevention programs in the field of violent radicalization.* Canadian Practitioners Network for the Prevention of Radicalization and Extremist Violence.
Hassan, G., Brouillette‐Alarie, S., Ousman, S., Savard, É. L., Kilinc, D., Madriaza, P., Varela, W., Pickup, D., Danis, E., & the CPN‐PREV team. (2021b). *A systematic review on the outcomes of tertiary prevention programs in the field of violent radicalization*. Canadian Practitioners Network for the Prevention of Radicalization and Extremist Violence.
Lewis, J. & Marsden, S. (2021). *Countering Violent Extremism Interventions: Contemporary Research*. Lancaster University, Lancaster: Centre for Research and Evidence on Security Threats (CREST).
Lewis, J., Marsden, S. & Copeland, S. (2020). *Evaluating Programmes To Prevent And Counter Extremism*. Lancaster University, Lancaster: Centre for Research and Evidence on Security Threats (CREST).
Mastroe, C. & Szmania, S. (2016). *Surveying CVE Metrics in Prevention, Disengagement and Deradicalization Programs*. University of Maryland: START.
Pistone, I., Eriksson, E., Beckman, U., Mattsson, C. & Sager, M. (2019). A scoping review of interventions for preventing and countering violent extremism: Current status and implications for future research. *Journal for Deradicalization*, 19, 1–84.
Morrison, J. F., Silke, A., Maiberg, H., Slay, C., & Stewart, R. (2021). *A Systematic Review Of Post‐2017 Research On Disengagement And Deradicalisation*, Lancaster University, Lancaster: Centre for Research and Evidence on Security Threats (CREST).
van Hemert, D., van den Berg, H., van Vliet, T., Roelofs, M., Huis in 't Veld, M., Marret, J., Gallucci, M. & Feddes, A. (2014). *Innovative Method and Procedure to Assess Counter‐violent‐radicalisation Techniques in Europe: Synthesis report on the state‐of‐the‐art in evaluating the effectiveness of counter‐violent extremism interventions*. IMPACT Europe Report.
Zeuthen, M. (2021). *Reintegration – Disengaging violent extremists: A systematic literature review of effectiveness of counter‐terrorism and preventing and countering violent extremism activities*. Report commissioned and financed by the Policy and Operations Evaluation Department (IOB) of the Netherlands Ministry of Foreign Affairs.

##### Search methods for languages other than English

As well as English, the review sought to identify research in German, French, Swedish, Danish, Norwegian, and Russian. Members of the research team are fluent or native speakers of these languages and used a comparable approach to that set out above to identify eligible studies. Because there are no codified guidelines for searching languages other than English (Walpole, 2019), the language specialists used a search strategy adapted for their linguistic context. The process of searching languages other than English is likely to be less comprehensive than the English language search process, because translating and analysing research in languages other than English is more resource intensive (Walpole, 2019) and due to the variety of databases and sources available in different country and linguistic contexts.

With the addition of an initial translation process, the search strategy for languages other than English followed the same approach used for English language searches:

1. Translating the English search terms and identifying alternative terms when direct translations did not exist.
2. Piloting the search terms using the approach set out above.
3. Searching the same platforms outlined in Table 1 using both (a) the English language search terms, with restrictions applied on language; and (b) the translated search terms.
4. Searching additional language‐specific academic and grey literature sources identified by each language specialist based on their knowledge of the relevant literature in specific languages. See Table 6 for a list of these sources.
5. Consulting members of the research team and advisory board to identify studies in languages other than English.
6. Forward and backward citation searching of studies identified through steps 1–5.

**Table 6:** Sources & databases used to identify research in languages other than English.

Source	Relevant language
CAIRN *Academic Research Repository*	French
Gallica *Academic Research Repository*	French
Pascal and Francis *Academic Research Repository*	French
Erudit *Academic Research Repository*	French
Persée *Academic Research Repository*	French
Center for the Prevention of Radicalization Leading to Violence *Intervention Provider and Research Institution*	French
Fonds de recherche du Québec *Public Agency*	French
Journal Exit‐Deutschland *Academic Journal*	German
Kriminologie *Academic Journal*	German
Degruyter *Academic Research Repository*	German
SpringerLink *Academic Research Repository*	German
Social Science Open Access Repository *Academic Research Repository*	German
Countering Extremism Project *Research Repository*	German
Vielfalt Mediathek *Research Repository*	German
Bundesarbeitsgemeinschaft religiös begründeter Extremismus (BAG RelEx) *Research Repository*	German
Forum Kriminalprävention (DFK) *Research Repository*	German
GESIS Leibniz‐ Institutfür Sozialwissenschaften *Research Institution*	German
Deutsches Jugendinstitut *Research Institution*	German
German Institute on Radicalization and De‐radicalization Studies *Research Institution*	German
Institut für Rechts‐und Kriminalsoziologie *Research Institution*	German
Monitoringssystem und Transferplattform Radikalisierung (MOTRA) *Research Institution*	German
Kriminologisches Forschungsinstitut Niedersachsen E.V. *Research Institution*	German
Hessische Stiftung Friedens‐ und Konfliktforschung (HSFK) *Research Institution*	German
Violence Prevention Network *Intervention Provider and Research Institution*	German
Nationale Zentrum Kriminalprävention (NZK) *Public Agency*	German
Bundeszentrale für politische Bildung (BPB) *Public Agency*	German
Bundesministeriums für Familie, Senioren, Frauen und Jugend (BMFSJ) *Public Agency*	German
Other university repositories *Academic Research Repositories*	German
Conflict Analysis & Prevention Center *Research Institution*	Russian
Institute of World Economy and International Relations (IMEMO) *Research Institution*	Russian
Organization for Security and Co‐operation in Europe (OSCE) *Research Repository*	Russian
The Program on New Approaches to Research and Security in Eurasia (PONARS) *Research Institution*	Russian
Center for Religious Studies of Kyrgyzstan at the Kyrgyz‐Russian Slavic University *Research Institution*	Russian
Indicator *Research Repository*	Russian
Psyjournals *Academic Research Repository*	Russian
Russian State Library *Research Repository*	Russian
Elibrary.ru *Academic Research Repository*	Russian
The Intellectual Center – Scientific Library Named After E.I. Ovsyankin *Research Institution*	Russian
Institute of Sociology of the Russian Academy of Sciences (ISRAS) *Research Institution*	Russian
CyberLeninka *Research Repository*	Russian
Russian National Library *Academic Research Repository*	Russian
Bibliographic resources of the Branch of the State Public Library for Science and Technology SB RAS *Research Repository*	Russian
Danish Institute for International Studies – DIIS *Research Institution*	Danish, Swedish, or Norwegian
Danish Centre for Extremism Prevention *Public Agency*	Danish, Swedish, or Norwegian
Danish Probation Service Resources *Public Agency*	Danish, Swedish, or Norwegian
Fryshuset *Intervention*	Danish, Swedish, or Norwegian
Anna Lindth Bibliotek Database *Academic Research Repository*	Danish, Swedish, or Norwegian
Stockholm International Peace Research Institute (SIPRI) *Research Institution*	Danish, Swedish, or Norwegian
University of Oslo Centre for Research on Extremism (C‐REX) *Research Institution*	Danish, Swedish, or Norwegian
Danish Social Ministry *Public Agency*	Danish, Swedish, or Norwegian
University College of Norwegian Correctional Service (KRUS) *Research Institution*	Danish, Swedish, or Norwegian
Other university repositories *Academic Research Repositories*	Danish, Swedish, or Norwegian

Adaptations were made to the searches to accommodate the limited search functionality of the Ovid and ProQuest platforms when undertaking the searches in languages other than English. These platforms did not support searches using some of the non‐English language characters. It was therefore only possible to search for research in languages other than English through these platforms using English search terms filtered to identify non‐English language studies.

#### 4.1.3 Data collection and analysis

##### Selection of studies

###### Title and abstract screening

The search results were imported into Endnote, duplicate records were removed and the final list was uploaded to Covidence.^1^ These results went through an initial phase of screening using the titles of records to remove all those that were obviously irrelevant and unrelated to radicalisation. Two reviewers (JL and CS) then assessed all titles and abstracts using the screening tool available in Supporting Information: Appendix II. Conflicts were discussed and where no agreement was reached, the final decision was made by a third reviewer (SM). A similar process was used to screen the research in languages other than English, where the relevant language specialists first reviewed the title and abstract removing obviously irrelevant studies not relating to radicalisation before assessing the abstracts using the screening tool in Supporting Information: Appendix II.

###### Full text screening

Studies retained following title and abstract screening went forward for full text review. Two reviewers (JL and CS) independently read the full texts of the English language studies using the same screening tool used for the title and abstract screening (see Supporting Information: Appendix II). Conflicts were discussed by the two screeners and any disagreements adjudicated by a third reviewer (SM). The full text review for languages other than English followed a similar strategy, with the relevant language specialists reviewing the full texts. Where there were conflicts, a final decision was reached through discussion between language specialists and the lead reviewer (JL). Any remaining conflicts were adjudicated by a final reviewer (SM).

##### Data extraction and management

A data extraction and coding tool (see Supporting Information: Appendix IV) was used to inform the full text coding process. This tool was used by the lead reviewer (JL) and the language specialists to capture information about the study (authors, title, source type, language, etc.); methods and research design; information about the intervention, and the tools or approaches that were used, alongside information relating to effectiveness and implementation; and details of the intervention context (country, population, delivery agents, etc.).

##### Assessment of risk of bias in included studies

The eligibility of qualitative, quantitative and mixed method research designs meant we used different risk of bias measures which were suitable for differing research designs. For Objective 1: on the effectiveness of case management interventions tools and approaches, only studies using a randomised experimental (i.e., Randomised Control Trials) or stronger quasi‐experimental research design were eligible. No studies were identified that addressed Objective 1 using these methods. Had eligible studies been identified, we intended to use the Cochrane Risk of Bias tool for randomised trials (RoB 2) or the Cochrane Risk of Bias in Non‐Randomised Studies – of Interventions (ROBINS‐I) tool (Sterne et al., 2016, 2019).

For Objective 2 on implementation, a wider range of research designs were eligible. The search process only identified studies using weaker quantitative research designs and qualitative research.^2^ To assess risk of bias for weaker quantitative studies, we used the Effective Public Health Practice Project (EPHPP) Quality Assessment Tool for Quantitative Studies, a tool used to determine the quality of studies in relation to selection bias, study design, confounders, blinding, data collection methods, and withdrawals and dropouts (see Supporting Information: Appendix III).

Qualitative studies addressing Objective 2 were assessed using the Critical Appraisal Skills Programme (CASP) checklist (see Supporting Information: Appendix III). This uses ten questions to assess a range of research designs including qualitative research, with each question answered as either ‘Yes’, ‘No’, or ‘Can't Tell’. Questions focus on the clarity of the research aims; appropriateness of methodology, research design, and recruitment strategy; data collection processes; relationships between researchers and participants; consideration of ethical issues; rigour of data analysis; clarity of research findings; and value of the research. Following a previous review by Mazerolle et al. (2021), studies were eligible for inclusion even when one or more question was answered as ‘Can't Tell’ or ‘No’, provided that the study did not have a critical weakness in relation to research design and sampling. To ensure that only studies of sufficient quality were included, where responses to the following two questions were ‘No’ or ‘Can't tell’, they were excluded:

‐ Is the research design appropriate to answer the question?
‐ Was the sampling strategy appropriate to the aims of the research?

To further ensure the quality of included studies, only studies where at least seven of the ten questions were answered as ‘Yes’ were included. Whilst the CASP tool is not designed to produce cumulative scores, including this step provided a further level of quality assurance.

Mixed method research designs were assessed using both tools: quantitative aspects of the studies were analysed using the EPHPP tool, and qualitative elements using CASP.

##### Measures of treatment effect

No eligible studies were identified that required an assessment of effect sizes. Had this been possible, or should an update of this review be undertaken, the process described in the protocol to calculate effect sizes would be used (Lewis et al., 2023).

##### Unit of analysis issues

Because of the research designs that were represented in the final selection of studies, there were no unit of analysis issues. That is, no studies assessing the effectiveness of case management interventions which reported several similar outcomes in a single study; used clustering in their research design; reported data from different points in time; and/or involved several studies reporting on one piece of research were eligible for inclusion (Mazerolle et al., 2021). If an update to this review is carried out, the means of addressing unit of analysis issues set out in the protocol will be used (Lewis et al., 2023).

##### Dealing with missing data

Missing data is a more readily identifiable and significant challenge in quantitative as opposed to qualitative research. The research designs reflected in the studies that went forward for inclusion in the review did not allow for additional statistical analyses such as effect sizes or meta‐analyses. There was therefore no need to contact authors for additional information to support this type of analysis.

##### Assessment of heterogeneity

The nature of the studies included in the review did not allow meta‐analyses to be conducted, which meant no assessment of heterogeneity was possible. Should an update to the review be undertaken, the approach described in the protocol will be used (Lewis et al., 2023).

##### Assessment of reporting biases

As there were no studies that went forward for the assessment of effectiveness (Objective 1) aspect of the review, it was not possible to carry out meta‐analyses and therefore assess publication or reporting biases. However, when reporting on the included studies, we quantify the number of published and unpublished studies, and comment on any identified differences in the results reported by published and unpublished studies. In any updates of this review, the approach set out in the protocol will be used (Lewis et al., 2023).

##### Data synthesis

###### Treatment of quantitative evaluation research (Objective 1)

No studies that would allow us to carry out a meta‐analysis to synthesise the findings of quantitative studies seeking to assess the effectiveness of case management interventions were eligible for inclusion. Should an update to the review be undertaken, quantitative data will be synthesised in line with the approach described in the protocol (Lewis et al., 2023).

###### Treatment of qualitative and weaker quantitative research (Objective 2)

To address Objective 2 on the implementation of case management interventions, qualitative research and weaker quantitative research designs that weren't eligible for more robust quantitative analysis were synthesised using the framework synthesis approach (Booth & Carroll, 2015) also used by Mazerolle et al. (2020) to analyse similar study designs in a comparable Campbell systematic review. This uses a framework to categorise data which is then synthesised using, in our case, narrative summaries.

Following Pollock et al. (2020) the synthesis of qualitative data involved an initial stage of deductive coding which was informed by the coding framework used to extract the data from the studies (see Supporting Information: Appendix IV) and then categorising the research according to the tools and approaches that were applied, either at different stages of the case management process, or which were used across the whole case management intervention. The findings were then coded inductively using thematic analysis to identify the themes not captured by the coding framework. These themes were used to synthesise the evidence relating to different case management tools and approaches, focusing on factors which facilitated, generated barriers, or acted as moderators to implementation.

##### Subgroup analysis and investigation of heterogeneity

No studies that made meta‐analysis, and hence subgroup analysis, possible were eligible for inclusion in the review. Should this be possible in future updates to the review, the approach outlined in the protocol will be used (Lewis et al., 2023).

##### Sensitivity analysis

It was not possible to carry out sensitivity analyses to understand the impact of the Risk of Bias results because of the nature of the studies that were included the review. If possible in future updates to this review, we will use the approach set out in the protocol (Lewis et al., 2023).

#### 4.1.4 Deviations from the protocol

The review made four deviations from the protocol. Two related to searching for and screening the literature in languages other than English, and two related to the methods of analysis used.

The strategy used to search for studies in languages other than English had to be adapted. We had intended to use the translated search terms to search each of the platforms listed in the protocol (reproduced in Table 1). However, a number of these platforms did not have the functionality to search non‐English characters (see Supporting Information: Appendix I for specific databases). We were therefore only able to search these databases using the English search terms, applying filters to filter out English studies. For consistency, we conducted two sets of searches in those databases that did allow us to use the translated search terms: (1) a search of the translated search terms in line with the approach outlined in the protocol; and (2) a separate search of the English search terms, with language filters applied.

A large number of the records returned by the searches in languages other than English had an English title and/or abstract. To remain consistent with the English language screening process, rather than ask the language experts to review these in the first instance, the primary reviewer (JL) conducted the initial relevancy screening of any English titles/abstracts returned by these searches. All titles and abstracts that were in languages other than English were then screened by the relevant language specialists.

Data analysis relating to Question 2b (on implementation) was structured around two elements of an intervention's theory of change as outlined in Section 1.2.6 of the protocol (Lewis et al., 2023); this covered implementation factors, and moderators. The structure used in the final review aligned with the protocol, however we refined the approach to interpreting implementation factors by examining two aspects of implementation: facilitators and barriers. There were three reasons for this: (1) it allowed for a more consistent, fine‐grained approach to synthesising the findings; (2) it is a distinction informed by our data extraction tool (see Supporting Information: Appendix IV); and (3) it was informed by the combination of deductive and inductive approaches to analysing the data we proposed to take in the protocol (see Section 4.8 of the protocol), which saw facilitators and barriers emerge as important codes from our analysis and which were then used to structure the results.

Finally, we did not review the transferability of findings to other contexts as originally outlined in Section 3.9 of the protocol, for two reasons. First, as we were unable to identify any eligible studies relating to Objective 1 (on effectiveness), it was not possible to assess whether effective interventions were transferable to different contexts. Second, the findings included in Part I were drawn from a wide range of countries and settings, which meant that the findings of Part I themselves reflected how different tools and approaches might be used in different contexts and served to highlight how contextual factors might influence the implementation (and thus the potential transferability) of tools.

### 4.2 Results

#### 4.2.1 Description of studies

##### Results of the search

**Figure 2:**
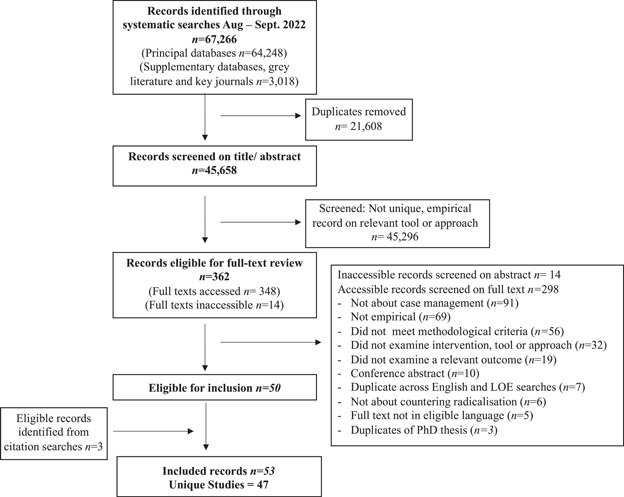
PRISMA flow diagram.

The results of the search and screening process are set out in Figure 2. The initial English database searches identified 49,410 records. An additional 14,838 records were identified from the searches in Languages other than English, which included research in French, German, Norwegian, Swedish and Danish, and Russian. These were combined with material identified through the search of grey literature sources, hand searches of relevant journals and consultation with experts (*n* = 3,018). A total of 67,266 records made up the initial corpus. After de‐duplication, 45,658 records went forward for title and abstract screening using the Covidence platform.

The title and abstract screening process led to the removal of 45,296 records, leaving 362 which went forward for full text review in Covidence. The full text of 14 records were unavailable in institutional repositories. We assessed the title and abstract of these records using the full‐text screening criteria and these were subsequently excluded. The remaining records were reviewed by two members of the research team and any conflicts adjudicated through discussion with a third team member.

From a total of 348 records that underwent full‐text screening, 50 went forward for inclusion in the review. No records met the inclusion criteria for Objective 1 on the effectiveness of case management interventions. All of the records therefore related to whether case management interventions were implemented as intended (Objective 2a), and/or discussed what influenced how interventions were implemented, focusing on factors which facilitated, generated barriers, or acted as moderators to implementation processes (Objective 2b). A further three eligible records were identified through forward and backward citation searches of the included records, leaving a total of 53 eligible records that were included in Part I. These records contained data relating to 47 different empirical studies. In total, seven studies contained data relevant to Objective 2a, and 47 contained data relevant to Objective 2b.

##### Included studies

A total of 53 unique records reporting on 47 studies met the inclusion criteria for the review. No eligible records or studies relating to intervention effectiveness (Objective 1) were identified, with all studies therefore included in the examination of implementation (Objective 2). A brief description of each of the studies is included in Table 7.

**Table 7:** Overview of included studies.

Study	Document type (I)	Document type (II)	Focus of analysis^a^	Intervention/tool name	Location	Relevant dates	Language
1. AEF (2018)	Government/other public agency report	Grey literature	Examination of Intervention(s)	Forsa	Netherlands	Established in 2015. Evaluation focuses on period 2015–2018.	English
2. Becker et al. (2014)	Government/other public agency report	Grey literature	Examination of Intervention(s)	XENOS	Germany	Established in 2009, and was initially due to operate until 2014. The evaluation was commissioned in 2010.	German
3. Cherney (2018)	Journal article	Academic literature	Examination of Intervention(s)	PRISM	Australia	Established in 2016. Research conducted in 2018.	English
4. Cherney (2020)	Journal article	Academic literature	Examination of Intervention(s)	PRISM	Australia	Established in 2016. Research conducted in 2017.	English
5. Cherney (2021)	Journal article	Academic literature	Discussion of Broader Practice and Challenges	Australian National Diversion Program	Australia	Established in 2014. Research conducted in 2017.	English
6. Cherney (2022)	Journal article	Academic literature	Examination of Intervention(s)	Intervention 1 (Part of Australian National Diversion Program)	Australia	Established in 2014. Evaluation conducted in 2020.	English
7. Cherney and Belton(2021a)	Journal article	Academic literature	Examination of Intervention(s)	Intervention 1 & Intervention 2 (Part of Australian National Diversion Program)	Australia	Established in 2014. Evaluation conducted in 2020.	English
8. Cherney and Belton (2021b)	Journal article	Academic literature	Examination of Intervention(s)	PRISM	Australia	Established in 2016. Research conducted in 2018.	English
9. Cherney et al. (2022)	Other academic output	Grey literature	Discussion of Broader Practice and Challenges	Examines issue of disguised compliance	Australia, Germany, UK and Indonesia	Not stated in paper.	English
10. Christensen (2015)	Dissertation/thesis	Academic literature	Examination of Intervention(s)	EXIT Sweden	Sweden	Established in 1998. Research conducted in 2012.	English
11. Corner and Pyszora (2022)	Journal article	Academic literature	Evaluation of Case Management Tool	TRAP‐18	Australia, UK, Canada	Not stated in paper.	English
12. Costa et al. (2021)	Journal article	Academic literature	Examination of Intervention(s)	Not named. Examines multiple interventions	Belgium, Denmark, Finland, France, Norway, The Netherlands, Germany	Research conducted 2020–2021.	English
13. Disley et al. (2016)	Research agency report	Grey literature	Evaluation of Case Management Tool	Multi‐Agency Public Protection Arrangements (MAPPA)	UK	Introduced in 2000, extended to terrorist offenders in 2010. Research conducted in 2010.	English
14. Eijkman and Roodnat (2017)	Journal article	Academic literature	Examination of Intervention(s)	‘Person‐specific’ interventions	Netherlands	Not stated in paper. Research conducted in 2016.	English
15. Fisher et al. (2020)	Think tank report	Grey literature	Examination of Intervention(s)	STRIVE‐II	Kenya	Established in 2017, due to end in 2000. Research conducted 2019–2020.	English
16. Førde and Andersen (2021)	Journal article	Academic literature	Evaluation of Case Management Tool	Conversations of Concern	Norway	Established in 2014. Research dates not stated.	English (via Norwegian search)
17. Harris‐Hogan (2020)	Journal article	Academic literature	Examination of Intervention(s)	Countering Violent Extremism Early Intervention Program (CVE‐EIP)	Australia	Established in 2015. Research dates not stated.	English
18a. Haugstvedt (2019) 18b. Haugstvedt (2022) 18c. Haugstvedt and Gunnarsdottir (2023) 18d. Haugstvedt and Tuastad (2021)	Journal article	Academic literature	Discussion of Broader Practice and Challenges	N/A. Examines work of social workers	Norway	Research conducted in 2018.	English
19. Hofinger and Schmidinger (2017)	Other academic output	Grey literature	Examination of Intervention(s)	DERAD deradicalisation programme	Austria	Established in 2016. Research dates not stated.	German
20. Inspector of Custodial Services NSW (2018)	Government/other public agency report	Grey literature	Discussion of Broader Practice and Challenges	N/A. Discusses broader management of radicalised mates, with some relevant lessons relating to case management	Australia	Research conducted 2016–2017.	English
21. Jukschat et al. (2020)	Government/other public agency report	Grey literature	Examination of Intervention(s)	N/A. Study examines deradicalisation projects delivered across all states in Germany	Germany	Operational 2017–2019. Study conducted 2017–2019.	German
22. Khalil et al. (2019)	Think tank report	Grey literature	Examination of Intervention(s)	Serendi Rehabilitation Centre	Somalia	Established in 2012. Research conducted 2015–2017.	English
23a. Kotzur et al. (2022) 23b. Vandaele et al. (2022a) 23c. Vandaele et al. (2022b)	Book chapter	Academic literature	Evaluation of Case Management Tool	N/A. Examines multi‐agency working across three European countries	Belgium, Netherlands, Germany	Research conducted 2020–2021.	English
24. Lukas (2006)	Think tank report	Grey literature	Examination of Intervention(s)	Democratic and social action as a key qualification for work, training and social integration	Germany	Operational 2001–2005. Study conducted 2001–2005.	German
25. Marsden (2015)	Journal article	Academic literature	Examination of Intervention(s)	London Probation Trust Central Extremism Unit (CEU)	UK	Established in 2009. Research conducted 2009–2010.	English
26. Mattsson (2021)	Journal article	Academic literature	Evaluation of Case Management Tool	N/A. Examines experiences of youth workers engaging young people	Sweden	Research conducted 2013–2016.	English
27. Möller and Neuscheler (2018)	Other academic output	Grey literature	Examination of Intervention(s)	Counselling Centre Hesse	Germany	Established 2014. Study conducted 2016–2017.	German
28. Möller et al. (2015)	Government/other public agency report	Grey literature	Examination of Intervention(s)	Exit Programme for Right‐Wing Extremists of the State of North Rhine‐Westphalia (APR NRW)	Germany	Established 2001 Research dates not stated.	German
29. Hellevik et al. (2022)	Journal article	Academic literature	Discussion of Broader Practice and Challenges	N/A. Examines how police officers engage with mental health issues and mental health practitioners in the context of countering radicalisation to violence	Norway	Research conducted 2016–2017.	English
30. Orban (2019)^b^	Government/other public agency report	Grey literature	Examination of Intervention(s)	Norwegian Mentoring System	Norway	Established in 2016. Research conducted 2017–2018.	Norwegian
31a. Pettinger (2020a) 31b. Pettinger (2020b)	Journal article	Academic literature	Examination of Intervention(s)	Channel	UK	First piloted in 2007, before rolled out nationally in 2012. In 2015, Channel was placed on a statutory footing. Research conducted 2017–2018.	English
32. Piltch‐Loeb et al. (2021)	Journal article	Academic literature	Evaluation of Case Management Tool	N/A. Examines case conferences	USA, North Macedonia, Sweden	Research conducted 2019–2020.	English
33. Raets (2022)	Journal article	Academic literature	Discussion of Broader Practice and Challenges	N/A. Examines local CVE work across Belgium, with relevant lessons for case management	Belgium	Research conducted 2019–2021.	English
34. Schroer‐Hippel (2019)	Government/other public agency output	Grey literature	Examination of Intervention(s)	KOMPASS Counselling Centre	Germany	Established 2015. Study conducted 2015–2017.	German
35. Schuhmacher (2018)	Other academic output	Grey literature	Examination of Intervention(s)	Legato Counselling Centre for Religiously Based Radicalisation	Germany	Established 2015. Study conducted 2017‐2018	German
36. Schuurman and Bakker (2016)	Journal article	Academic literature	Examination of Intervention(s)	Team TER Reintegration Programme	Netherlands	Established in 2012. Research conducted 2013–2014.	English
37. Sizoo et al. (2022)	Journal article	Academic literature	Discussion of Broader Practice and Challenges	N/A. Examines cross‐sectoral collaboration	Netherlands	Not stated in paper.	English
38. Solhjell et al. (2022)	Journal article	Academic literature	Evaluation of Case Management Tool	N/A. Examines case conferences	Norway, Denmark, Finland and Sweden	Research conducted 2018–2020.	English
39. Spalek et al. (2010)	Think tank report	Grey literature	Evaluation of Case Management Tool	West Midlands 1‐2‐1 Mentoring Programme	UK	Established in 2009, end date unclear. Research conducted in 2010.	English
40. Stern et al. (2023)	Journal article	Academic literature	Discussion of Broader Practice and Challenges	N/A. Examines practices of probation officers	USA	Research conducted 2020–2021.	English
41. Sträter and Stuppert (2019)	Think tank report	Grey literature	Examination of Intervention(s)	Clearing Procedure and Case Management – Prevention of Violent Neo‐Salafism and Right‐Wing Extremism'	Germany	Operational 2016–2019 Study conducted 2017–2019.	German
42. Thompson and Leroux (2022)	Journal article	Academic literature	Examination of Intervention(s)	FOCUS Toronto and ReDirect (Calgary)	Canada	Research conducted in 2018.	English
43. van de Weert and Eijkman (2020)	Journal article	Academic literature	Discussion of Broader Practice and Challenges	N/A. Examines delivery of local prevention efforts	Netherlands	Research conducted 2017–2018.	English
44. van der Heide and Schuurman (2018)	Journal article	Academic literature	Examination of Intervention(s)	Team TER Reintegration Programme	Netherlands	Established in 2012. Research conducted 2015–2016.	English
45. Webster et al. (2017)	Government/other public agency report	Grey literature	Evaluation of Case Management Tool	Structured Risk Guidance (SRG)	UK	Piloted in 2009. Research conducted in 2010.	English
46. Weeks (2018)	Journal article	Academic literature	Discussion of Broader Practice and Challenges	Experiences of Home Office‐accredited Intervention Providers	UK	Home Office funding of mentoring began in 2007. Research dates not stated.	English
47. Weggemans and de Graaf (2017)	Book	Academic literature	Discussion of Broader Practice and Challenges	N/A Experiences of former extremist detainees and practitioners working with them during and after release	Netherlands	Research began in 2011. End date unclear.	English

^a^ Examination of Intervention(s): Study examines or evaluates the implementation of specific intervention(s) in their entirety; Evaluation of Case Management Tool: Study examines a specific case management tool, or only certain stage(s) of the case management process; Discussion of Broader Practice and Challenges: Study offers broader reflections on the case management process, but does not examine an intervention or tool in detail.

^b^ Supplementary information relating to this evaluation is also drawn from an English paper published by the same author that was excluded (Orban, 2022).

Data relating to these 47 studies is drawn from 34 published, peer‐reviewed records and 19 non‐published records. Published records included peer reviewed journal articles (*n* = 30), books (*n* = 1) and book chapters (*n* = 3). Non‐published records included PhD theses (*n* = 1), government/public agency outputs (*n* = 8), think tank or research agency reports (*n* = 6), and other academic outputs (*n* = 4). All were published between 2006 and 2023, with over half (*n* = 29) published since 2020. The following describes the main characteristics of the 47 unique studies covering the participants; settings; study designs; intervention types; and outcomes. An overview of these key characteristics is provided in Table 8.

**Table 8:** Characteristics of included studies.

Study	Research design	Description of intervention, tool, or topic	Sample
1. AEF (2018)	Qualitative evaluation of the effectiveness and structural continuity of the Forsa and Family Support interventions delivered by LSE in the Netherlands. Some descriptive statistics drawn from programme documentation are presented. Only data relating to Forsa used in the review.	**Forsa** is a tertiary counselling intervention that works with individuals who have been convicted of extremist offences or who are/have been involved in extremist networks. Clients are provided with a range of services tailored to their individual needs on a voluntary basis. Programme is coordinated by, and delivered through the National Support Centre for Extremism (LSE).	Interviews with practitioners and stakeholders from a range of different agencies (number not stated), and three clients of Forsa.
2. Becker et al. (2014)	Mixed methods process and impact evaluation that included quantitative analysis of programme metrics and case data, and multiple forms of primary research (e.g., analysis of document, surveys of programme staff and participants, observation, interviews).	The **XENOS** programme funded fifteen projects between 2010‐2013 spanning secondary and tertiary prevention. Thirteen of these projects used case management, often in conjunction with other services.	Quantitative analysis of project application documents, change requests, financial data and case data for 566 clients supported through the different projects. Two online surveys completed by 14 and 13 of the projects respectively; observations of projects; semi‐structured interviews with team leaders, practitioners, multi‐agency partners, and other stakeholders; telephone interviews with those responsible for individual projects; and client survey (*n* = 50).
3. Cherney (2018)	Second evaluation of PRISM that was conducted in 2018, based on interviews with current or former staff and clients.	**The Proactive Integrated Support Model (PRISM)** intervention is a custody‐based, multi‐agency case management intervention that spans secondary and tertiary prevention. PRISM delivers individually tailored intervention plans to inmates who have been convicted for terrorism offences or who have been identified as being at risk of radicalisation. The intervention is voluntary and delivered by team of psychologists working with other partners.	Semi‐structured interviews with 10 current or former staff, and 12 current or former clients. This sample is part of a large sample of 38 respondents.
4. Cherney (2020)	First evaluation of PRISM that was conducted in 2017, based on interviews with current or former staff and clients.	**The Proactive Integrated Support Model (PRISM)** intervention is a custody‐based, multi‐agency case management intervention that spans secondary and tertiary prevention. PRISM delivers individually tailored intervention plans to inmates who have been convicted for terrorism offences or who have been identified as being at risk of radicalisation. The intervention is voluntary and delivered by team of psychologists working with other partners.	Semi‐structured interviews with 28 respondents drawn from larger sample of 55 staff, stakeholders, clients and families. Sample includes community corrections personnel (*n* = 16); Correctional Intelligence Group; (*n* = 1); PRISM psychologists (*n* = 2); prison chaplain (*n* = 2); offenders (*n* = 6); and family member (*n* = 1).
5. Cherney (2021)	Qualitative research study exploring specific challenges relating to the management and release of radicalised inmates in Australia.	No specific intervention examined, although sample includes staff from PRISM. Study is a broader review of how radicalised inmates are managed during and after release from custody, and the challenges faced by inmates and by practitioners.	Semi‐structured interviews (*n* = 55) with community corrections personnel (*n* = 28); state and federal police (*n* = 4); Correctional Intelligence Group (*n* = 2); PRISM team (*n* = 2); psychologist (*n* = 1); NGO/community organisation (*n* = 5); Imam (*n* = 2); Inspectorate of Custodial Services (*n* = 1); prison chaplain (*n* = 2); offenders (*n* = 7); and family member of offender (*n* = 1). Insights from offenders and family not included in analysis.
6. Cherney (2022)	Mixed methods evaluation of Intervention 1 drawing on quantitative analysis of case note data and qualitative analysis of case note information and interview data to examine client progress. *This paper draws on the same sample of Intervention 1 clients examined in* Cherney and Belton (2021a).	**Intervention 1** is a multi‐agency case management intervention that spans secondary and tertiary prevention. It originally began as a community‐based secondary intervention, before being expanded to include custody‐based work with terrorist offenders. Clients are provided with tailored intervention plans on a voluntary basis.	Analysis of case notes for 15 intervention 1 clients, and semi‐structured interviews with staff (*n* = 3); client (*n* = 2).
7. Cherney and Belton (2021a)	Content analysis of risk assessment and case notes completed for clients of Intervention 1 and 2 to identify intervention goals and track client progress against goals over time. *This paper draws on the same sample of Intervention 1 clients in* Cherney (2022).	**Intervention 1 and 2** are comparable multi‐agency, case management interventions that operate in separate Australian states/territories. Clients are provided with tailored intervention plans on a voluntary basis.	Analysis of case note data for 15 Intervention 1 clients and 5 Intervention 2 clients. Interviews with intervention staff and clients. Intervention 1: three staff members and two clients; Intervention 2: five staff members and two clients.
8. Cherney and Belton (2021b)	Second evaluation of PRISM that was conducted in 2018. Content analysis of risk assessments and case notes completed for clients of PRISM to identify specific intervention goals, and to track client progress against these goals over time.	**The Proactive Integrated Support Model (PRISM)** intervention is a custody‐based, multi‐agency case management intervention that spans secondary and tertiary prevention. PRISM delivers individually tailored intervention plans to inmates who have been convicted for terrorism offences or who have been identified as being at risk of radicalisation. The intervention is voluntary and delivered by team of psychologists working with other partners.	Analysis of case notes for 14 clients, and analysis of risk assessments completed for 11 of these clients.
9. Cherney et al. (2022)	Qualitative exploration of practitioners' perspectives on the issue of disguised compliance when working with radicalised clients.	Research examines practitioner perceptions on the issue of **disguised compliance,** and approaches for overcoming this issue. The study does not focus on one specific intervention programme, and draws on interviews with experts in different countries.	Semi‐structured interviews with 24 ‘subject matter experts’ who are directly involved in delivering CVE interventions in community and/or correctional contexts.
10. Christensen (2015)	Qualitative anthropological design that uses fieldwork, interviews, and participant observation to examine the lived experiences of EXIT‐Sweden clients and coaches.	**EXIT‐Sweden** is a ‘self‐help’ mentoring and counselling programme that works to support individuals from leaving the White Power Movement. It is based in the Fryshuset centre in Sweden. EXIT works closely with a range of multi‐agency partners, including therapists, social workers, teachers and police.	21 semi‐structured interviews with 15 respondents (both staff and clients), fieldwork in Fryshuset, and participant observation over the course of three fieldwork periods.
11. Corner and Pyszora (2022)	Qualitative exploratory study examining the potential applicability of the TRAP‐18 assessment tool to the Australian context.	**The Terrorist Radicalization Assessment Protocol (TRAP‐18)** is a risk assessment tool specifically designed for assessing lone actors. It consists of 18 factors: eight proximal warning behaviours; and ten distal characteristics.	Focus groups and interviews with 58 experts and users. Ten user focus groups (total *n* = 48); two user interviews and three expert focus groups (*n* = 8).
12. Costa et al. (2021)	Qualitative exploratory study examining the characteristics of exit programmes across different countries in mainland Europe.	Does not examine or name specific intervention, but looks to codify the characteristics of **exit programmes across Europe**, including the implementation of different elements of the case management process, and relevant tools.	Semi‐structured interviews with practitioners from 14 exit programmes, in 7 countries across mainland Europe (*n* = 17).
13. Disley et al. (2016)	Exploratory qualitative study that examines the challenges of extending MAPPA to include the management of terrorist offenders upon their release into the community, with a focus on two criminal justice areas in England.	**Multi‐Agency Public Protection Arrangements (MAPPA)** provide a framework for multi‐agency partners to identify, assess, ad manage certain types of offenders – including terrorist offenders – upon their release into the community.	Semi‐structured interviews with 10 practitioners and stakeholders, including police and probation practitioners from two urban criminal justice areas (*n* = 8) and stakeholders from the national headquarters of the National Offender Management Service (*n* = 2).
14. Eijkman and Roodnat (2017)	Qualitative exploratory study that examines the implementation and the effects of ‘person‐specific’ interventions that are delivered to individuals identified as being at risk of radicalisation.	No specific measure, but study focuses on the implementation and effects of **‘person‐specific’ interventions** that are tailored to individuals, and which are delivered at the local municipality level in the Netherlands.	10 practitioners with experience of implementation across different municipalities in the Netherlands. Sample included seven municipal officers who are in charge of co‐ordinating local activity (including two in so‐called ‘priority’ regions), and three representatives of partner organisations).
15. Fisher et al. (2020)	Programme evaluation of the STRIVE‐II programme drawing on secondary and primary qualitative and quantitative data.	The **Strengthening Resilience to Violent Extremism (STRIVE II**) project is organised around four strands, but only one is examined in the review. This mentorship strand involves offering counselling and mentoring to young people who are identified as being at risk of radicalisation in different regions across Kenya. Potential clients are identified using strict eligibility criteria, and program documentation suggests that mentorship is tailored to individual needs.	Primary data relating to mentorship from semi‐structured qualitative interviews with mentors (*n* = 26); observation of two mentor training sessions; two focus groups with mentees (total *n* = 18); and telephone interviews with other stakeholders; baseline, midline and end‐line questionnaires completed by STRIVE II mentors, mentees and community stakeholders.
16. Førde and Andersen (2021)	Qualitative evaluation of the ‘conversation of concern’ approach drawing on interviews and observation data.	Examines the **‘conversation of concern’** approach that is used by police to reach out to young people in the community who are identified as being at risk of different forms of criminality. This study specifically focuses on the use of this tactic in the context of countering radicalisation to violence.	Semi‐structured interviews with 12 police officers of different rank (*n* = 12) and additional observations.
17. Harris‐Hogan (2020)	Exploratory qualitative study examining the implementation of the Countering Violent Extremism Early Intervention Program (CVE‐EIP).	The **Countering Violent Extremism Early Intervention Program (CVE‐EIP)** is an early intervention, multi‐agency, case management programme that was introduced in 2015, and operating in all eight of the Australian states and territories. The CVE‐EIP is a national programme that is tailored to the individual needs of each state or territory.	18 semi‐structured interviews with policymakers and practitioners involved in the CVE‐EIP.
18a. Haugstvedt (2019) 18b. Haugstvedt (2022) 18c. Haugstvedt and Gunnarsdottir (2023) 18d. Haugstvedt and Tuastad (2021)	Qualitative exploratory study examining the experiences of social workers in Norway who are involved in CVE work.	This study examines the experiences of **social workers in Norway who engage in CVE work**. Whilst not explicitly stated, the broader context suggests that this engagement occurs as part of broader case management processes in some cases.	17 interviews and two focus groups with social workers engaged in CVE work in Norway.
19. Hofinger and Schmidinger (2017)	Qualitative process and impact evaluation drawing on primary and secondary data.	Clearing session and intervention sessions (deradicalisation) with prisoners and those released from prison (transition management and aftercare) from Austrian prisons, carried out by the external organisation **DERAD.**	114 individual problem‐centred interviews with clients accused of supporting a terrorist organisation (*n* = 39); prison staff working in different fields (*n* = 49); and experts (*n* = 26). Secondary analysis of documentation (indictments and sentences for 41 persons accused of supporting a terrorist organisation that were accessible to external users; reports from the DERAD association).
20. Inspector of Custodial Services NSW (2018)	Qualitative inspection of five maximum security institutions in New South Wales (NSW) to examine the management of radicalised inmates.	Does not discuss a specific intervention. Discussion centred **around physical management within the prison setting**. Whilst not solely focused on case management, identifies relevant lessons relating to the case management process.	Qualitative interviews with over 200 staff and 18 inmates. Not all data is relevant to case management, or to this review. Additional quantitative analysis of programme data.
21. Jukschat et al. (2020)	Qualitative process evaluation drawing on interviews and participant observation.	Study examines **model deradicalisation projects** delivered across all states in Germany. A total of 15 (sub‐) projects use a case management approach during detention and/or probation, and/or the transition from detention. A total of 180 cases managed across these projects, most of which were still being managed at the time of the evaluation.	Narrative semi‐structured interviews (*n* = 65) with various representatives of all evaluated projects (*n* = 37), including interviews specifically on case management (*n* = 4); representatives of the probation service (*n* = 6); representatives of prisons where model projects are active (*n* = 7); responsible officials in the ministries of justice (*n* = 9); and inmates who participated in group activities of the model projects (*n* = 6). Participant observations of group work with inmates (*n* = 6), further training measures (*n* = 3), and team meetings (*n* = 2). E‐mail follow up to query the current work status of all model projects with regard to individual case work, group measures and further training courses.
22. Khalil et al. (2019)	Qualitative exploration of the experiences of current and former residents of the Serendi Rehabilitation Centre.	The **Serendi Rehabilitation Centre** is a residential centre that offers rehabilitation programming for ‘low‐risk’ former members of Al‐Shabaab. Residents are provided with a range of services that are tailored to their individual needs, with residency intended to last around 6–7 months, up to a maximum of around 1 year (p. 3).	Qualitative interviews with 129 current or former residents of the Centre.
23a. Kotzur et al. (2022). 23b. Vandaele et al. (2022a) 23c. Vandaele et al. (2022b)	Process evaluation of multi‐agency working structures in Germany, the Netherlands, and Belgium, drawing on participatory observation and interviews.	This study examines local **multi‐agency working arrangement**s in three European countries, including a focus on case management. Sample includes representatives of Belgian LISC‐R (Local Integrated Security Cells Radicalisation), Dutch CSHs (Care and Safety Houses), and some German multi‐agency working approaches.	47 interviews with 51 practitioners working in local multi‐agency structures across three cities in Germany, two cities in the Netherlands, and five in Belgium. Participatory observations of 14 multi‐agency meetings. Includes eight observations in Belgium (four cities), three in the Netherlands (two cities) and three in Germany (two cities).
24. Lukas (2006)	Qualitative process evaluation drawing on interviews and programme data.	Study examines a **voluntary individual support programme for post‐release offenders** lasting over a year that is delivered as a follow‐up to a prison‐based group training programme for young offenders. Support can take the form of telephone support, coaching or intensive individual support.	Interviews with clients (*n* = 39) in a group setting and with trainers of the 14 groups after 1–2 years of experience with the programme (*n* unstated), and analysis of reports and case history forms completed by trainers.
25. Marsden (2015)	Qualitative exploratory study examining management of terrorist offenders upon their release into the community.	**MAPPA** supported process of multi‐agency working; community organisations worked on a 1‐2‐1 basis with offenders to support their reintegration in a mentor‐like relationship that was a prototype for the current intervention model used in England.	Semi‐structured interviews with probation officers (*n* = 9) and observation of their work.
26. Mattsson (2021)	Qualitative case study of how youth workers engaged in CVE work during a specific period marked by the rise of the Islamic State (IS).	Examines the experience of **‘lock pickers’,** youth workers who would be tasked with engaging young people in the local community who were considered to be at risk of radicalisation.	Qualitative semi‐structured interviews with 11 youth workers and three managers working in a specific neighbourhood of Gothenburg marked by radicalisation.
27. Möller and Neuscheler (2018)	Qualitative process and formative evaluation drawing on a variety of primary research methods. Also presents some descriptive statistics drawn from case data.	The **Counselling Centre Hesse** is based in the German state of Hesse, and is a local partner of the Federal Office for Migration and Refugees (BAMF) network of local advice centres. The services span secondary and tertiary prevention and include case‐managed support for young people.	Analysis of programme documentation; participant observation of team meetings (*n* = 5); case consultations (*n* = 5); and safety conferences/situation meetings (*n* = 5); open interviews with centre staff (*n* = 9) and partner organisation (*n* = 1); semi‐structured interviews with clients (*n* = 10); 4 workshops, interviews with individual staff members to discuss specific cases (*n* unstated).
28. Möller et al. (2015)	Mixed methods outcome (quantitative) and process (qualitative) evaluation.	The **Exit Programme for Right‐Wing Extremists of the State of North Rhine‐Westphalia (APR NRW**) is a tertiary prevention programme that uses a case management model. An individualised help plan is developed for each client to target specific issues (e.g., addiction, violence, etc.), and support is offered to work through these issues, and to develop a ‘new vision of a liveable life’. Programme can usually be discontinued after 2–3 years.	Quantitative outcome evaluation involved analysis of case note data (descriptive analysis; comparison of admitted and non‐admitted, and successful and unsuccessful cases). Data available for 145 people who received at least one in‐depth contact, 99 of whom were accepted onto the programme (including 46 who have not yet completed the programme). Qualitative process evaluation examined the appropriateness, effectiveness and efficiency of the exit programme through document analysis; semi‐structured interviews with practitioners (*n* = 6), the head of the unit (*n* = 1), current and former clients (*n* = 8, supplemented with data from case files), and parents (*n* = 2).
29. Hellevik et al. (2022)	Qualitative examination of how police officers engage with mental health issues and mental health practitioners in the context of countering radicalisation to violence.	Study examines **how police officers engage with mental health issues and mental health practitioners** in the context of countering radicalisation to violence.	Qualitative semi‐structured interviews with police officers (*n* = 12) across four police districts in Norway.
30. Orban (2019)	Qualitative process evaluation based on interviews with practitioners, stakeholders and clients.	**The Norwegian Mentoring System (NMS**) is delivered in correctional contexts in Norway. The scheme spans secondary and tertiary prevention, and is designed for inmates accused of engagement in violent extremism, as well as those considered to be at risk of being radicalised.	Interviews with mentors (*n* = 9), mentees (*n* = 8) and other stakeholders (*n* = 20), including prison staff working with participants in the program, prison wardens and designers of the Norwegian Mentoring System (NMS).
31a. Pettinger (2020a) 31b Pettinger (2020b)	Qualitative exploratory study examining the implementation of Channel.	**Channel** is a multi‐agency case management intervention for individuals identified as being at risk of radicalisation. Individuals who are assessed as being in need of support by a Channel panel receive a tailored package of support.	Qualitative semi‐structured interviews with 18 practitioners, including 6 Channel mentors and other local actors engaged in countering radicalisation to violence.
32. Piltch‐Loeb et al. (2021)	Qualitative study using Nominal Group Technique (NGT) sessions in three countries to examine multi‐agency collaboration in practice.	An assessment of how **multi‐agency collaboration** works in practice using the Nominal Group Technique (NGT) to examine simulated case conferences in Denver (US), Gothenburg (Sweden), Skopje (North Macedonia).	Simulated case conferences involving practitioners in Denver (*n* = 78), Gothenburg (*n* = 30), and Skopje (*n* = 27).
33. Raets (2022)	Qualitative exploration of local CVE practice across Belgium.	This study does not examine a specific intervention, but examines the **broader working practices of CVE practitioners** working in secondary and tertiary prevention, identifying areas of good practice, as well as implementation challenges and issues.	Semi‐structured interviews with local practitioners and officials, and policymakers (*n* = 50).
34. Schroer‐Hippel (2019)	Mixed methods process and impact evaluation consisting of quantitative analysis of case data, and primary research using qualitative and quantitative methods.	The **KOMPASS Counselling Centre** provides a voluntary intervention programme (counselling) to prevent radicalisation and work on deradicalisation of young people in the context of religiously based extremism; the primary target group is young people who are in the process of radicalisation or already want to distance themselves from the extremist scene, the secondary target group is relatives and supporters of the clients.	Quantitative analysis of case data for 67 clients; one group discussion; case‐related surveys at two points in time (interval of six months) with case managers regarding 22 ongoing cases; interviews with counsellors (*n* = 5); analysis of programme documentation.
35. Schuhmacher (2018)	Mixed methods concept, structure and process evaluation that included quantitative analysis of programme metrics and case data; secondary analysis of programme documentation; and multiple forms of primary data collection (e.g., surveys, focus groups, participatory observation, and interviews).	The **Legato** counselling centre is based in Hamburg, and is a local partner of the Federal Office for Migration and Refugees (BAMF) network of local advice centres. Services provided by Legato span secondary and tertiary prevention, and include counselling for the social environment of at‐risk and (potentially) radicalised Islamist individuals; and disengagement and exit counselling (see BAMF, 2020, p. 41).	One pre‐evaluation discussion group and one thematic guideline‐based group interview with the entire team at the end of the evaluation (*n* not stated); individual interviews with programme staff (*n* = 3); expert interviews with external stakeholders (*n* = 6). Analysis of intake forms and case lists of the counselling centre, case reports, concept and framework papers of the centre, published texts by staff members, and official sources. Survey of staff. 13 participatory observations of network meetings, social space meetings, training events and team meetings as well as further observations of the day‐to‐day life of the centre.
36. Schuurman and Bakker (2016)	Initial process and impact evaluation of Team TER reintegration programme.	The **Team TER reintegration programme** offers tailored support to inmates convicted of extremist offences or those suspected of engaging in such activities who are about to be released on parole, and clients on parole. Intervention is delivered by a specialist team within the Dutch probation service in partnership with other agencies.	Three rounds of interviews with practitioners in Team TER (*n* = 6), and one interview with liaison within Dutch National Coordinator for Security and Counterterrorism (NCTV).
37. Sizoo et al. (2022)	Qualitative exploratory study of multi‐agency collaboration between mental health and security professionals.	No specific intervention. Examines perceptions of, and challenges to, **intersectoral collaboration between security and mental health professionals** in the context of countering radicalisation to violence.	Focus groups (total *n* = 22) and semi‐structured interviews (total *n* = 29) with security and mental health professionals and trainers.
38. Solhjell et al. (2022)	Series of simulated case discussions and follow‐up interviews to examine operation of multi‐agency working in practice.	Examines **multi‐agency collaboration** during simulated case conferences in three cities in Nordic countries.	13 simulated case discussions in four cities that involved 78 participants. Group interviews and follow‐up interviews conducted with all participants to examine reflections on simulated case discussion, and on own work.
39. Spalek et al. (2010)	Qualitative process evaluation.	The **West Midlands (WM) 1‐2‐1 Mentoring Scheme** was introduced to produce a pool of mentors that could be used as a common resource for different agencies and interventions, spanning secondary and tertiary prevention. It is therefore not a specific intervention, but an example of tool for the selection and quality assurance of mentors.	Semi‐structured interviews (*n* = 16) with mentors and other stakeholders in the programme including steering group members, mentor selection panel members, project board members, and steering group members.
40. Stern et al. (2023)	Mixed methods exploratory study of the experiences and needs of probation officers working in the United States.	Study examines the **practices and needs of probation officers** in the context of working with radicalised offenders or offenders considered to be at risk of radicalisation.	Qualitative interviews with 39 federal probation officers across 27 districts and survey responses from a sample of 206 officers, 73% (150) of whom had experience overseeing violent extremists.
41. Sträter and Stuppert (2019)	Qualitative process evaluation.	This evaluation examines the delivery of the **‘Clearing Procedure and Case Management – Prevention of Violent Neo‐Salafism and Right‐Wing Extremism’** pilot project in six German high schools. This project used a seven stage, case management process to identify and offer school‐based measures to students at risk of radicalisation. A pedagogical specialist coordinates the clearing procedure and is supported in the planning and implementation of measures by a clearing team consisting of the head teacher, class teacher, clearing officer and school social worker.	Three one‐day workshops, each involving one clearing professional; interviews with one representative of school social work in each of the six participating schools; focus group discussions at all participating schools with 4 to 8 teachers in each group; interviews with 4 experts in radicalisation prevention; telephone interviews with the head masters of the six participating schools.
42. Thompson and Leroux (2022)	Formative evaluation of two interventions.	The study reflects on the learnings of evaluations of two case managed interventions in Canada: **Focus Toronto, and ReDirect**. Both programmes are multi‐agency interventions that design and deliver tailored packages of support to individuals identified as being at risk of radicalisation using a situation table model.	Observed 64 FOCUS meetings and three ReDirect meetings. Anonymous survey of all FOCUS table members (*n* = 83) Semi‐structured interviews with FOCUS Toronto (*n* = 34) and ReDirect (*n* = unstated) situation table members.
43. van de Weert and Eijkman (2020)	Qualitative exploratory case study.	Examines the delivery of **early intervention programming** at the local municipality level in the Netherlands.	Open interviews (using probes) with local municipality officials in priority areas in the Netherlands (*n* = 15).
44. van der Heide and Schuurman (2018)	Second process and impact evaluation of Team TER reintegration programme, following earlier evaluation (Schuurman & Bakker, 2016).	The **Team TER reintegration programme** offers tailored support to inmates convicted of extremist offences or those suspected of engaging in such activities who are about to be released on parole, and clients on parole. Intervention is delivered by a specialist team within the Dutch probation service in partnership with other agencies.	Three rounds of semi‐structured interviews: (1) May 2016: TER team's 11 staff members, 2 managers, 1 policy officer, the RN manager overseeing at national level, 3 public prosecutors, 1 NCTV policy advisor. (2) Nov 2016–Jan 2017: 13 Team TER staff (2 new members had joined), 5 clients, three employees of partner agencies. (3) May–June 2017: Same respondents as Round 1. A smaller fourth round of interviewing was conducted in 2018 to examine relationships between Team TER and key stakeholders: three local municipalities, and the National Support Centre for Extremism (LSE).
45. Webster et al. (2017)	Process evaluation of the piloting of the Structured Risk Guidance.	The **Structured Risk Guidance (SRG)** was a new, specialist risk assessment tool for violent extremist offenders that was piloted in 2009. The SRG was revised in 2012 and became the ERG 22+.	Qualitative interviews with strategic and operational staff (*n* = 15) and offenders who had been assessed using the SRG (*n* = 3) across four case study sites.
46. Weeks (2018)	Qualitative exploratory study examining the experiences of Home Office‐accredited intervention providers in the UK.	Study examines the practices of **Home Office‐accredited intervention providers** working across the secondary and tertiary prevention space in the UK.	23 semi‐structured interviews with intervention providers and six post‐release offenders.
47. Weggemans and de Graaf (2017)	Qualitative, exploratory study into the reintegration of jihadist extremist detainees in the Netherlands.	Examines the practice of **reintegrating jihadist detainees** based on capturing the experiences of former extremist detainees during and after their incarceration, and practitioners involved in working with detainees before and after their release.	Semi‐structured interviews with former extremist detainees (*n* = 10), and case workers and professionals (*n* = 37). Also interviewed small number of social scientists and journalists with relevant expertise (*n* = unstated).

###### Settings

Forty‐one studies focused on a single country, whilst six examined multiple countries. In total, data was collected from samples in 17 countries, with Germany (*n* = 11), the Netherlands (*n* = 9) and Australia (*n* = 10) the countries that were examined most frequently. The other countries examined were the United Kingdom (*n* = 8), Norway (*n* = 6), Belgium (*n* = 3), Sweden (*n* = 4), Denmark (*n* = 2), Canada (*n* = 2), Finland (*n* = 2), the United States (*n* = 2), France (*n* = 1), Indonesia (*n* = 1), Austria (*n* = 1), Kenya (*n* = 1), Somalia (*n* = 1) and North Macedonia (*n* = 1).

Thirty‐seven were published in English, nine in German, and one in Norwegian. No eligible studies were identified that were published in Russian, French, Danish or Swedish.

###### Research designs

The vast majority of studies examined projects using primary qualitative and/or quantitative research methods (*n* = 46), including thirteen studies that used both primary and secondary research data. One of the included studies only used secondary research data.

Every study used qualitative data. This included 37 studies reporting on qualitative research designs (including five studies that provided basic descriptive statistics alongside a qualitative analysis, but which did not report on quantitative methods or a detailed quantitative analysis); two studies that only presented a quantitative analysis of data collected using qualitative methods; and eight mixed methods studies.

A range of methods of primary data collection were used including group and/or individual qualitative interviews (*n* = 45), participant observation (*n* = 9), quantitative surveys (*n* = 5) and simulated case conferences (*n* = 2). Secondary data from case records and other programme documentation was also commonly examined (*n* = 14).

The quantitative data used included survey data (*n* = 5); quantitative analyses of data collected using qualitative interviewing (*n* = 3) and simulated case conferences (*n* = 1); and data from programme documentation and records (*n* = 8).

Almost all studies presented qualitative analysis (*n* = 45). The exceptions were two studies that presented a quantitative analysis of qualitative data. In total 32 studies only presented data in qualitative form, whilst 15 presented quantitative data: five qualitative studies that did not conduct any detailed quantitative analysis, but provided some basic statistics; two studies that only presented a quantitative analysis of qualitative data; and the eight mixed methods studies.

###### Qualitative research participants

Most of the studies that used interviews or focus groups interviewed practitioners (*n* = 44). Practitioners were drawn from a range of agencies and contexts, including the police, probation, independent intervention service providers, and social workers. Other programme stakeholders – including programme managers, policymakers, local and national government officials, and representatives of agencies partnered with interventions – were interviewed in 19 studies. Clients were interviewed in 21 studies, with three of these studies also interviewing family members of clients. Academic and other experts were interviewed for 6 studies. The samples of qualitative participants ranged from 5 to 218 participants, although the exact size was not always stated.

###### Quantitative research participants

All five studies that used quantitative surveys engaged with practitioners, including counsellors, mentors and other programme staff, and probation officers. In addition, three surveyed clients, and one surveyed other programme stakeholders. Whilst the survey size was not always stated, the largest sample size reported in the included studies was 206 participants.

###### Interventions

Twenty‐seven studies examined an intervention or multiple interventions, and nine studies focused on a specific case management tool. Eleven studies examined practices and challenges related to case management based on interviews with practitioners and/or clients.

Twelve studies examined secondary prevention; fourteen focused on tertiary prevention; and twenty‐one analysed data relating to work spanning both secondary and tertiary interventions. The studies examined a range of delivery settings including prisons and correctional settings (*n* = 4); community contexts (*n* = 11); and work that spanned both correctional and community contexts (*n* = 18), including correctional‐based interventions that extended into the post‐release and probation context. One study examined a school‐based case management intervention in Germany. Six studies analysed interventions delivered through specialist counselling centres: Forsa and the Family Support Centre (the Netherlands), Fryshuset youth centre (Sweden), the Serendi Rehabilitation Centre (Somalia), The Legato counselling centre (Germany), The KOMPASS Counselling Centre (Germany), and the Counselling Centre Hesse (Germany). The specifics of the delivery context were not discussed in the remaining seven studies.

It was not possible to develop a comprehensive typology of different case management approaches based on the data available in the included studies. Whilst we had hoped to develop such a typology by examining the explicit or implicit theories of change underpinning different interventions, the relevant information was largely absent. However, seven eligible studies examined the assumptions underpinning case management interventions and/or the implementation of clearly articulated programme logics that informed individual case management interventions. These assumptions were examined individually.

Forty‐three studies examined the use of tools and related implementation factors during a specific stage or multiple stages of the case management process: client identification (*n* = 2); client assessment (*n* = 26); case planning (*n* = 5); delivery (*n* = 28); monitoring and evaluation (*n* = 16); and transition/exit (*n* = 10). A typology of the different tools identified across these different stages of the case management process is presented in Table 9 below.

**Table 9:** Typology of tools examined within included studies.

Stage	Tools and themes examined
Client identification (*n* = 2)	Outreach work with potential clients (*n* = 2)
Client assessment (*n* = 26)	Screening tools (*n* = 3) Multi‐agency client assessment (*n* = 14) RNA tools (*n* = 12)
Case planning (*n* = 5)	Intervention planning tools (*n* = 3) Multi‐agency case conferences (*n* = 2)
Delivery and implementation (*n* = 28)	Tailoring intervention services & goals (*n* = 19) Practitioner characteristics & approaches (*n* = 20) Practitioner supervision & quality assurance (*n* = 13)
Monitoring and evaluation (*n* = 16)	Client assessment tools (*n* = 9) Case file and case note data (*n* = 7) Case conferences (*n* = 5) Less structured qualitative data (*n* = 5)
Transition and exit (*n* = 10)	Exit and aftercare approaches (*n* = 3) Post‐exit and post‐release processes (*n* = 7)

Forty‐one studies provided empirical evidence relating to implementation factors relevant to the case management process as a whole (i.e., that were not specific to individual stages), and 28 studies described findings relating to different moderators of the case management process.

Data relating to implementation factors focused on the use, importance, and challenges associated with multi‐agency working (*n* = 34); the impact of risk‐oriented logics (*n* = 17); public and political pressure (*n* = 10); resourcing (*n* = 17); staff expertise (*n* = 23) and training (*n* = 16); voluntary and mandatory interventions (*n* = 11); and the impact of broader legislation (*n* = 8). The moderators examined in the included studies were delivery context (*n* = 11); local context (*n* = 10); standalone interventions (*n* = 4); and client challenges (*n* = 4).

##### Excluded records

In total, 298 records were excluded at the full text screening stage. The main reasons for exclusion were the intervention, tool, or approach examined was not relevant to case management (*n* = 91); the record was not empirical (*n* = 69) or did not meet the methodological inclusion criteria (*n* = 56); and/or the record did not examine an intervention, tool, or approach (*n* = 32). Other reasons for exclusion were the record did not examine a relevant outcome (*n* = 19); the record was a conference abstract only (*n* = 10); duplicate references across the English and searches in languages other than English (*n* = 7); the record was not about countering radicalisation to violence (*n* = 6); and the full text was not in an eligible language (*n* = 5). Three were excluded as they were duplicates of chapters in an included PhD thesis.

#### 4.2.2 Risk of bias in included studies

The CASP tool described in Section 4.1.3 was used to assess elements of the 45 qualitative or mixed methods studies. The quality appraisals for each study, and each domain, are shown in Table 10. Whilst the authors of the CASP tool do not recommend a scoring system, it is worth noting that only three of the included studies were assessed as having no limitations across the ten different domains covered in CASP (Cherney, 2018; Jukschat et al., 2020; Orban, 2019). We only included studies that did not have a critical weaknesses across seven of the ten domains, and which therefore scored positively on a majority of the domains assessed by the tool. Only studies meeting the below criteria were included:

- Well‐defined research question
- Appropriate research design
- Appropriate recruitment strategy
- Appropriate data collection strategy
- Clear statement of findings
- Qualitative data considered appropriate
- Valuable research that discussed contribution made to the literature

**Table 10:** CASP ratings of included studies (*n* = 45).

Study	Well defined question?	Qualitative method appropriate?	Research design appropriate for research aims?	Appropriate recruitment strategy?	Data collection appropriate for research?	Relationship between researcher and participants adequately considered?	Ethical issues taken into consideration?	Rigorous data analysis?	Clear statement of findings?	Valuable research?
1. AEF (2018)	Yes	Yes	Yes	Yes	Yes	Can't Tell	Can't Tell	Can't Tell	Yes	Yes
2. Becker et al. (2014)	Yes	Yes	Yes	Yes	Yes	Can't Tell	Can't Tell	Yes	Yes	Yes
3. Cherney (2018)	Yes	Yes	Yes	Yes	Yes	Yes	Yes	Yes	Yes	Yes
4. Cherney (2020)	Yes	Yes	Yes	Yes	Yes	Can't Tell	Can't Tell	Yes	Yes	Yes
5. Cherney (2021)	Yes	Yes	Yes	Yes	Yes	Can't Tell	Can't Tell	Yes	Yes	Yes
6. Cherney (2022)	Yes	Yes*	Yes	Yes	Yes	Can't Tell	Yes	Yes	Yes	Yes
7. Cherney and Belton (2021a)	Yes	Yes*	Yes	Yes	Yes	Can't Tell	Yes	Yes	Yes	Yes
9. Cherney et al. (2022)	Yes	Yes	Yes	Yes	Yes	Can't Tell	Can't Tell	Can't Tell	Yes	Yes
10. Christensen (2015)	Yes	Yes	Yes	Yes	Yes	Yes	Yes	Can't Tell	Yes	Yes
11. Corner and Pyszora (2022)	Yes	Yes	Yes	Yes	Yes	Can't Tell	Yes	Can't Tell	Yes	Yes
12. Costa et al. (2021)	Yes	Yes	Yes	Yes	Yes	Can't Tell	Yes	Yes	Yes	Yes
13. Disley et al. (2016)	Yes	Yes	Yes	Yes	Yes	Can't Tell	Can't Tell	Can't Tell	Yes	Yes
14. Eijkman and Roodnat (2017)	Yes	Yes	Yes	Yes	Yes	Can't Tell	Can't Tell	Yes	Yes	Yes
15. Fisher et al. (2020)	Yes	Yes	Yes	Yes	Yes	Yes	Can't Tell	Yes	Yes	Yes
16. Førde and Andersen (2021)	Yes	Yes	Yes	Yes	Yes	Yes	Yes	Can't Tell	Yes	Yes
17. Harris‐Hogan (2020)	Yes	Yes	Yes	Yes	Yes	Can't Tell	Can't Tell	Can't Tell	Yes	Yes
18a. Haugstvedt (2019) 18b Haugstvedt (2022) 18c Haugstvedt and Gunnarsdottir (2023) 18d Haugstvedt and Tuastad (2021)*	Yes	Yes	Yes	Yes	Yes	Can't Tell	Yes	Yes	Yes	Yes
19. Hofinger and Schmidinger (2017)	Yes	Yes	Yes	Yes	Yes	No	Yes	Yes	Yes	Yes
20. Inspector of Custodial Services NSW (2018)	Yes	Yes	Yes	Yes	Yes	Can't Tell	Can't Tell	Can't Tell	Yes	Yes
21. Jukschat et al. (2020)	Yes	Yes	Yes	Yes	Yes	Yes	Yes	Yes	Yes	Yes
22. Khalil et al. (2019)	Yes	Yes	Yes	Yes	Yes	Yes	Yes	Can't Tell	Yes	Yes
23a. Kotzur et al. (2022) 23b. Vandaele et al. (2022a) 23c. Vandaele et al. (2022b)	Yes	Yes	Yes	Yes	Yes	Can't Tell	Can't Tell	Yes	Yes	Yes
24. Lukas (2006)	Yes	Yes	Yes	Yes	Yes	No	Can't Tell	Yes	Yes	Yes
25. Marsden (2015)	Yes	Yes	Yes	Yes	Yes	Can't Tell	Can't Tell	Yes	Yes	Yes
26. Mattsson (2021)	Yes	Yes	Yes	Yes	Yes	Can't Tell	Yes	Yes	Yes	Yes
27. Möller and Neuscheler (2018)	Yes	Yes	Yes	Yes	Yes	No	Can't Tell	Yes	Yes	Yes
28. Möller et al. (2015)	Yes	Yes	Yes	Yes	Yes	No	Can't Tell	Yes	Yes	Yes
29. Hellevik et al. (2022)	Yes	Yes	Yes	Yes	Yes	Can't Tell	Yes	Yes	Yes	Yes
30. Orban (2019)	Yes	Yes	Yes	Yes	Yes	Yes	Yes	Yes	Yes	Yes
31a. Pettinger (2020a) 31b, Pettinger (2020b)	Yes	Yes	Yes	Yes	Yes	Can't Tell	Can't Tell	Can't Tell	Yes	Yes
33. Raets (2022)	Yes	Yes	Yes	Yes	Yes	Can't Tell	Yes	Yes	Yes	Yes
34. Schroer‐Hippel (2019)	Yes	Yes	Yes	Yes	Yes	No	Yes	Yes	Yes	Yes
35. Schuhmacher (2018)	Yes	Yes	Yes	Yes	Yes	No	Yes	Yes	Yes	Yes
36. Schuurman and Bakker (2016)	Yes	Yes	Yes	Yes	Yes	Can't Tell	Can't Tell	Can't Tell	Yes	Yes
37. Sizoo et al. (2022)	Yes	Yes	Yes	Yes	Yes	Can't Tell	Yes	Yes	Yes	Yes
38. Solhjell et al. (2022)	Yes	Yes	Yes	Yes	Yes	Can't Tell	Yes	Yes	Yes	Yes
39. Spalek et al. (2010)	Yes	Yes	Yes	Yes	Yes	Can't Tell	Can't Tell	Can't Tell	Yes	Yes
40. Stern et al. (2023)	Yes	Yes	Yes	Yes	Yes	Yes	Can't Tell	Can't Tell	Yes	Yes
41. Sträter & Stuppert (2019)	Yes	Yes	Yes	Yes	Yes	No	Can't Tell	Can't tell	Yes	Yes
42. Thompson and Leroux (2022)	Yes	Yes	Yes	Yes	Yes	Can't Tell	Yes	Yes	Yes	Yes
43. van de Weert and Eijkman (2020)	Yes	Yes	Yes	Yes	Yes	Yes	Can't Tell	Yes	Yes	Yes
44, van der Heide and Schuurman (2018)	Yes	Yes	Yes	Yes	Yes	Yes	Yes	Can't Tell	Yes	Yes
45. Webster et al. (2017)	Yes	Yes	Yes	Yes	Yes	Can't Tell	Yes	Yes	Yes	Yes
46. Weeks (2018)	Yes	Yes	Yes	Yes	Yes	Can't Tell	Can't Tell	Can't Tell	Yes	Yes
47. Weggemans and de Graaf (2017)	Yes	Yes	Yes	Yes	Yes	Yes	Yes	Can't Tell	Yes	Yes

* Answers relate to the individual record with the lowest risk of bias reporting on specified study.

Table 10 highlights that the answers to the questions relating to the other three domains were ‘No’ or ‘Can't Tell’ for several studies. Whilst these domains are no less important than the seven listed above, we did not exclude studies based on these domains in isolation as such answers did not necessarily highlight a critical weakness. It is not unusual for published and non‐published studies to fail to specifically reference ethical issues or the relationship between researcher and participants, and so we did not exclude studies based on these domains. Studies that had obviously failed to consider these issues would have been excluded, including studies that were clearly conducted in an unethical way, or which failed to discuss an obvious power imbalance between researchers and participants. However, these issues did not appear to be present in the included studies. Similarly, whilst studies that clearly lacked a rigorous approach to data analysis would have been excluded, studies that did not specify a specific approach to data analysis were included where the authors presented evidence in support of their findings and conclusions.

The quantitative components of the eight mixed methods studies and the two studies that only presented a quantitative analysis of qualitative data were assessed using the EPHPP tool described in Section 4.1.3. However, as shown in Table 11, domains relating to confounders, blinding, and withdrawals/dropouts were not relevant to assessing the specific quantitative research designs included in the review (i.e., one‐time surveys and quantitative analysis of programme documentation), and so were not used to assess study quality.

**Table 11:** EPHPP assessment of included studies (*n* = 10).

Study	Selection bias	Study design	Confounders	Blinding	Data collection	Withdrawals and dropouts	Overall rating
2. Becker et al. (2014)	Moderate	Moderate	N/A	N/A	Moderate	N/A	Moderate
6. Cherney (2022)	Moderate	Moderate	N/A	N/A	Moderate	N/A	Moderate
7. Cherney and Belton (2021a)	Moderate	Moderate	N/A	N/A	Moderate	N/A	Moderate
8. Cherney and Belton (2021b)	Moderate	Moderate	N/A	N/A	Moderate	N/A	Moderate
15. Fisher et al. (2020)	Moderate	Moderate	N/A	N/A	Moderate	N/A	Moderate
28. Möller et al. (2015)	Moderate	Moderate	N/A	N/A	Moderate	N/A	Moderate
32. Piltch‐Loeb et al. (2021)	Moderate	Moderate	N/A	N/A	Moderate	N/A	Moderate
34. Schroer‐Hippel (2019)	Moderate	Moderate	N/A	N/A	Moderate	N/A	Moderate
35. Schuhmacher (2018)	Moderate	Moderate	N/A	N/A	Moderate	N/A	Moderate
40. Stern et al. (2023)	Moderate	Moderate	N/A	N/A	Moderate	N/A	Moderate

Whilst it was not possible to assess for publication bias, a comparison of the themes identified in published and non‐published studies highlighted that the results of both were comparable.

#### 4.2.3 Synthesis of results

The following discussion of the results is made up of three parts: the first reviews the research on case management approaches, addressing Objectives 1 (on effectiveness) and 2 (on implementation); the second covers the same objectives for case management tools split according to the different stages of the case management process; whilst the third discusses the research relating to both objectives as it relates to case management as an overarching process.

#### 4.2.4 Case management approaches

The analysis of approaches is split into four sections: Identifying case management approaches; Assessing the effectiveness of case management approaches; Examining the implementation of case management approaches; and Identifying implementation factors and moderators that influence how approaches are delivered.

##### Identifying case management approaches

To categorise different case management approaches, we attempted to identify the constituent parts of the programme logic or theory of change (i.e., the approach) underpinning each tool or intervention. This involved coding each study according to different elements of an implicit or explicit theory of change: drivers; domains; levels of analysis; mechanisms; and progress/outcome measures (see Supporting Information: Appendix IV for the extraction tool that informed this coding process).

Using this process, we identified a small number of interventions and tools that were explicitly or implicitly underpinned by particular approaches. These broadly fell into two approaches identified in the protocol (Lewis et al., 2023): the Risk Needs Responsivity (RNR) model (e.g., Cherney, 2018, 2021), and strengths‐based approaches (e.g., Marsden, 2015; Raets, 2022). Notably, the boundaries between these differing programme logics were typically not explicit, and many interventions and tools reflected aspects of both models (e.g., Marsden, 2015).

An intervention or tool was categorised as being informed by the RNR model when risk reduction was identified as a primary, and explicit, goal of the case management process. The use of the RNR model was evidenced by references to risk‐oriented intervention logics (e.g., Cherney, 2021), intervention goals (e.g., van der Heide & Schuurman, 2018), or the use of risk‐oriented case management tools (e.g., Corner & Pyszora, 2022). A defining feature of this approach was that the goals that were set for individual clients focused on tackling risk factors to reduce their risk of radicalisation (in secondary interventions), or of terrorist or violent extremist recidivism (in tertiary interventions) (e.g., Cherney & Belton, 2021a, 2021b).

An intervention or tool was categorised as being informed by a strengths‐based approach based on its adherence to the basic principles of this approach as outlined in the literature (Marsden, 2017). This approach was evidenced by an emphasis on building strengths and skills considered to be important for long‐term rehabilitation (e.g., AEF, 2018; Christensen, 2015; Eijkman & Roodnat, 2017; Khalil et al., 2019; Raets, 2022), and/or supporting clients in pursuing pro‐social alternatives to violent extremism (e.g., Eijkman & Roodnat, 2017; Möller et al., 2015; Orban, 2019; Schuhmacher, 2018; van der Heide & Schuurman, 2018). For example, the stated objective of **Forsa** in the Netherlands was ‘to reinforce protective factors to facilitate individuals to renounce extremist violence and/or to distance themselves from extremist networks (disengagement)’ (AEF, 2018, p. 22). Similarly, case management delivered as part of the **XENOS** project in Germany was underpinned by the assumption that providing education, training and work played a role in facilitating young people's exit from right‐wing extremism (Becker et al., 2014).

Unfortunately, as noted above, limitations in the data meant we were not able to develop a comprehensive typology of different case management approaches. As a result, we were not able to categorise every intervention or tool as being either primarily risk‐oriented or strengths‐based, or to develop a more nuanced categorisation. Even those studies that explicitly examined a specific theory of change or programme logic (i.e., an approach) did not provide sufficient detail for us to develop categories of comparable approaches. Taken together then, this meant it was not possible to assess the effectiveness or implementation of different types of case management approach as conceptualised above, and in the protocol (Lewis et al., 2023).

However, a small number of studies examined individual interventions' implicit or explicit theory of change (i.e., an approach). For example, Schuurman and Bakker (2016) analysed whether the underlying ‘cognitive’ and ‘operational’ logics of the **Team TER reintegration programme** were evidence informed. Because these assessments and theories of change were specific to each programme they could not be easily categorised into a typology. Thus, whilst it was not possible to examine categories of case management approaches in the way set out in the protocol, this section examines the internal logics of individual interventions.

##### The effectiveness of case management approaches in countering radicalisation to violence (Objective 1)

No studies were identified which assessed the effectiveness of case management approaches which sought to counter radicalisation to violence. A small number of studies provided qualitative (e.g., Cherney & Belton, 2021a, 2021b) and/or quantitative (e.g., Becker et al., 2014; Möller et al., 2015; Cherney & Belton, 2021a, 2021b) data to suggest that specific interventions had been effective in supporting clients. However, these studies did not meet the methodological criteria to be included in the analysis of effectiveness. An important lesson from studies that did provide some outcome data is that no intervention – even those assessed as working well – is likely to be 100% successful.

Whilst this is true of interventions operating in a range of different fields, it is particularly important in the context of countering radicalisation to violence given the impact that terrorist recidivism can have on the social and political discourse and the scrutiny that interventions face for what are perceived as failures of public protection (e.g., Goldberg & Clifton, 2020). Whilst calls for typically more punitive changes to interventions are common in the aftermath of such events, this does not mean that singular cases of recidivism should be taken as evidence of an intervention being fundamentally flawed without proper evaluation and investigation of the case and the programme (Cherney, 2022).

##### The implementation of case management approaches (Objective 2a)

Seven eligible studies examined the assumptions underpinning case management interventions and/or the implementation of clearly articulated programme logics that informed individual case management interventions (Becker et al., 2014; Schuurman & Bakker, 2016; Möller & Neuscheler, 2018; van der Heide & Schuurman, 2018; AEF, 2018; Thompson & Leroux, 2022; Harris‐Hogan, 2020). These studies examined secondary interventions in Canada (FOCUS Toronto and ReDirect) and Australia (CVE‐EIP); tertiary interventions in the Netherlands (the Team Terrorism, Extremism & Radicalisation (TER) Reintegration Programme, and Forsa); and programmes in Germany focusing on both secondary and tertiary forms of prevention (XENOS and the Counselling Centre Hesse). Whilst seven studies represents a relatively small body of evidence, the evidence relating to Objective 2a was assessed as having a low risk of bias based on the results of assessments completed using the CASP tool, with over half of these studies scoring positively on eight (*n* = 2) or nine (*n* = 2) of the ten critical domains in this tool.

###### Assessing the assumptions underpinning case management approaches

Four studies assessed a case management intervention's programme logic by examining its underlying assumptions against current research relating to countering radicalisation to violence (Schuurman & Bakker, 2016; van der Heide & Schuurman, 2018; AEF, 2018; Becker et al., 2014). These studies analysed two interventions in the Netherlands: The Team TER reintegration programme, and the Forsa programme; and case management interventions delivered as part of the XENOS project in Germany.

The assumptions underpinning these interventions were considered sound. Two separate evaluations of the ‘program theory’ underpinning the **Team TER reintegration programme** reported that its underlying ‘cognitive’ and ‘operational’ logics were evidence informed, and appropriate (Schuurman & Bakker, 2016; van der Heide & Schuurman, 2018). The first evaluation, conducted in 2013‐2014 concluded that the cognitive logic – ‘the mechanisms thought to make it an effective means for achieving the desired ends’ – was realistic and in line with the evidence base (Schuurman & Bakker, 2016, p. 72). The intervention's dual focus on deradicalisation and disengagement was considered appropriate given the lack of clarity in research over which is likely to produce better outcomes. The operational logic of the programme, or the ‘assumptions being made about the capacity of the mandated organization to actually implement the measures successfully’ was also considered sound (Schuurman & Bakker, 2016, p. 73). The assumption that the Dutch Probation Service (in which Team TER was based) were best placed to deliver this intervention due to their experience reintegrating non‐terrorism related offenders was considered reasonable. The second evaluation, conducted in 2016‐2017, confirmed these conclusions (van der Heide & Schuurman, 2018).

An evaluation of **Forsa** similarly concluded its methods were supported by academic research (AEF, 2018). This assessment was based on determining that the methods Forsa used reflected twelve of thirteen key elements that the authors had identified from their review of the research on what works to counter radicalisation (AEF, 2018. P. 23).^3^ However, the evaluators acknowledged that the state of knowledge of this topic was limited. The evaluation of **XENOS** (Germany) similarly concluded that it was informed by scientific research (Becker et al., 2014).

###### Examining the implementation of case management approaches

Four studies examined whether interventions in the Netherlands and in Germany were implemented in ways that aligned with their underlying programme logics (Schuurman & Bakker, 2016; van der Heide & Schuurman, 2018; AEF, 2018; Möller & Neuscheler, 2018). Two further studies identified how weaknesses in underlying theories of change can create implementation challenges (Harris‐Hogan, 2020; Thompson & Leroux, 2022).

In the Netherlands, **Forsa** and the **Team TER** programme were found to be implemented in ways consistent with their programme logic. However, several challenges were identified. Both evaluations of the **Team TER** programme were positive about how the intervention was being implemented but pointed to some divergence from its cognitive and operational logic (Schuurman & Bakker, 2016; van der Heide & Schuurman, 2018). The first evaluation noted that the initial implementation of the programme diverged from the expectations of the Dutch National Coordinator for Security and Counterterrorism (NCTV) in two ways: the Dutch Probation Service did not oversee clients’ whole reintegration process in the way the NCTV had initially envisioned (Schuurman & Bakker, 2016, p. 73); and the NCTV felt that practitioners focused too heavily on addressing practical reintegration issues at the expense of trying to bring about cognitive, attitudinal change. Whilst the first issue was overcome during the evaluation period, the second remained ‘a point of friction’, with staff seen to primarily work towards promoting disengagement rather than deradicalisation. Although the evaluators considered this to be appropriate, this was not in line with the expectations of the NCTV. In those cases where some form of ideological intervention was deemed important, external consultants were used, but they were identified as a ‘potential weakness’ of the programme because it was not possible to assess their efficacy (Schuurman & Bakker, 2016, p. 72).

The second evaluation of **Team TER** was positive that staff were ‘avoiding a one‐sided focus on either disengagement or deradicalization as the only suitable way to minimize recidivism risk’ (van der Heide & Schuurman, 2018, p. 213), and would focus on promoting deradicalisation when it was deemed appropriate or feasible. However, they also highlighted variation in how these goals were implemented. These differences were shaped by assessments of how amenable clients were to deradicalisation or disengagement, and by practitioners' analysis of whether these goals were realistic. The majority of interviewees still felt as though work to tackle beliefs ‘remained underemphasized’ (van der Heide & Schuurman, 2018, p. 217). The evaluation called for a more systematised approach to deradicalisation, but also noted that over time, ideological factors had become a less significant explanation for radicalisation.

The second evaluation of the **Team TER reintegration intervention** also concluded that ‘assumptions about disengagement and deradicalization appear to have been translated into a theoretically effective set of tools’ (van der Heide & Schuurman, 2018, p. 215). However, implementation challenges were noted, including difficulties providing pro‐social alternatives to violent extremism, such as finding employment or participating in education. This was challenging as many of the alternatives available to clients, such as specific jobs, were ‘mundane’ when considered against the sense of purpose that membership of an extremist group might have provided (van der Heide & Schuurman, 2018, pp. 215‐216).

The AEF (2018) evaluation of **Forsa** found that the programme ‘operate[s] in accordance with a self‐developed methodology’ (p. 27). The evaluators were positive that these methodologies continued to evolve as practitioners developed greater experience. However, the evaluation suggested that this practice based approach should be validated through a more systematic examination of the methods used in the context of individual cases.

In Germany, an evaluation of the **Counselling Centre Hesse** similarly concluded that staff had implemented counselling in a way that was consistent with underlying ‘theoretical‐conceptual considerations’ (Möller & Neuscheler, 2018, p. 157), and that the Centre had achieved results that reflected both the objectives of both the Centre and the authority which contracted the work.

Two studies illustrated how weaknesses in underlying theories of change can create implementation challenges. An examination of Australia's **Countering Violent Extremism Early Intervention Program (CVE‐EIP)** noted that its underlying logic was unclear to practitioners who had been tasked with implementing it (Harris‐Hogan, 2020). Interviewees suggested that the programme had been ‘launched without any clear understanding of what was being proposed, nor agreement among key stakeholders regarding the overarching goal of the program’ (Harris‐Hogan, 2020, p. 107). This lack of a broader vision led to a disconnect between policy and practice. None of the practitioners interviewed for this study viewed the programme goal of prevention as set by policymakers to be a feasible or appropriate outcome.

An evaluation of the programme assumptions and logic models underpinning two interventions in Canada – **FOCUS Toronto** and **ReDirect (Calgary)** – identified two issues (Thompson & Leroux, 2022). Stakeholders had been hesitant to refer individuals into both programmes because they did not have clear expectations about the outcomes they were trying to achieve. This meant that stakeholders found it difficult to describe the outcomes they expected the interventions to deliver. Second, in the case of ReDirect, the ‘underlying theory of change for the program was non‐existent’ (p. 10) as it failed to link intended outcomes to activities. Whilst the programme had initially aimed to decrease ‘violent ideology’, the original logic model did not contain any activities that targeted ideological factors. A key output from the evaluation was therefore the development of a logic model with refined programme assumptions, redefined outcomes, and activities that were adapted so they were better linked to outcomes.^4^

##### Influences on the implementation of case management approaches (Objective 2b)

No eligible studies assessed the factors that facilitated, generated barriers or which related to moderators relevant to the implementation of case management approaches.

#### 4.2.5 Case management tools

The analysis of tools is split into three sections: Assessing the effectiveness of case management tools; Examining the implementation of case management tools; and Identifying implementation factors and moderators that influence how approaches are delivered.

##### The effectiveness of case management tools (Objective 1)

No eligible studies were identified which examined whether case management tools were effective in supporting efforts to counter radicalisation to violence.

##### The implementation of case management tools (Objective 2a)

No eligible studies were identified which examined whether case management tools were being implemented as expected.

##### Influences on the implementation of case management tools (Objective 2b)

This section examines 47 eligible studies which speak to research objective 2b. The analysis that follows explores the factors that influence the implementation of tools used to support the different stages of the case management process (see Figure 1 for a graphic representation of the stages). This analysis focuses on factors that facilitate and support implementation, as well as barriers which inhibit it. Whilst our analysis also identified a number of moderators, or ‘contextual conditions’ that impacted various stages of the case management process, the discussion of moderators is reserved for the examination of the case management process as a whole that follows in Section 4.2.6 because moderators were typically relevant to multiple stages.

The discussion is organised according to the stage of the case management process that the research relates to, ranging from client identification; client assessment, covering sub‐themes on screening tools, multi‐agency client assessment (incorporating discussion of multi‐agency collaboration, and using multi‐agency case conferences to assess clients), risk and needs assessment tools (covering inconsistency in use, perceived utility of tools, subjectivity, role of expertise, political climate, and organisational support for assessors); case planning, with sub‐themes on intervention planning tools and case conferences; delivery and implementation, with separate discussions on tailoring intervention goals and services, practitioner characteristics and approaches, and practitioner supervision and quality assurance; monitoring and evaluation, covering client assess‐ ment tools, case files and case notes, case conferences, and less structured forms of qualitative data; and transition and exit.

The evidence relating to Objective 2b was assessed as having a low risk of bias based on the results of assessments conducted using the CASP and/or EPHPP assessment tools. All ten studies assessed using the EPHPP tool were assessed as being of ‘moderate’ quality, whilst the risk of bias identified using the CASP tool varied across the 45 assessed studies. Ten of these studies met the minimum threshold for inclusion by scoring positively on seven of the ten domains contained within the CASP tool. The remaining 35 studies scored above this threshold by scoring positively on eight (*n* = 11), nine (*n* = 21), or all ten (*n* = 3) domains.

###### Stage 1: Client identification

Two studies presented data relating to tools that were used to identify and engage with potential clients (Mattsson, 2021; Førde & Andersen, 2021). Both studies described implementation factors relevant to this identification stage. Whilst the strength of evidence relating to this stage of the case management process was very limited, the risk of bias identified within these studies was low, with both studies scoring positively on nine of the ten domains in the CASP tool.

Implementation barriers were identified in research examining the use of **‘lock pickers’** in Sweden; these are youth workers tasked with identifying and engaging young people at risk of radicalisation, and if needed, passing on relevant information to other agencies. Mattsson (2021) noted that between 2013 and 2015, the practice of only hiring lock pickers on short‐term contracts, and institutional mistrust towards them meant that, at times, ‘information was not passed beyond the local management level because those with the most vital knowledge had weak positions in the organization and seldom were part of staff meetings’ (p. 10).

Further challenges were identified in an empirical study examining the **‘Conversation of Concern’** approach used in Norway and Denmark (Førde & Andersen, 2021). This involves police officers reaching out to individuals considered at risk of engaging in criminality, including engagement in violent extremism. In some instances, individuals were referred to a case management intervention following a conversation. This study raised concerns relating to the police's role in these conversations, due to potentially blurring commitments to care and control (Førde & Andersen, 2021). Related issues linked to police involvement identified in the context of multi‐agency working are discussed in more detail in Section 4.2.6.

###### Stage 2: Client assessment

Data relating to client assessment tools were identified across twenty‐six eligible studies. These studies presented data relating to the implementation of three tools: screening tools; multi‐agency assessment forums; and risk and needs assessment (RNA) tools.

####### Screening tools

Three eligible studies presented evidence relating to the implementation of different types of eligibility screening tools (Christensen, 2015; Fisher et al., 2020; Khalil et al., 2019). The risk of bias within these studies was assessed as very low, with all three studies scoring positively on nine out of the ten critical domains within the CASP qualitative assessment framework. Thus, whilst the overall strength of evidence relating to this specific tool was limited, the results of individual studies cited here can be considered robust based on the assessments conducted.

Coaches working for **EXIT Sweden** (Christensen, 2015) felt the presence of two people during the initial assessment of potential clients helped ensure that key issues were not overlooked, thereby addressing a central cause of a ‘failed client case’ (p. 251) by reducing the chances that coaches were not aware of relevant information. This process was also considered helpful in capturing information relevant to future intervention planning.

Clearly defined eligibility criteria, which is understood by all stakeholders, help to support the decision of who to accept onto a programme (Khalil et al., 2019; Fisher et al., 2020). The **Serendi Rehabilitation Centre** in Somalia for former members of Al‐Shabaab is only open to those assessed as being ‘low risk’, who are defined as those who have voluntarily left Al‐Shabaab; rejected their ideology; and who are believed not to present a public safety risk (Khalil et al., 2019). Following earlier research that had identified flaws in how potential clients were being screened, a standardised client assessment tool was developed to better support this process.

Fisher et al. (2020) describe a fixed set of ‘primary’ and ‘secondary’ criteria used by the **STRIVE intervention** in Kenya, with only those meeting at least one of the primary and two of the secondary criteria eligible to participate in the programme.^5^ These criteria were described as helping reduce the risk that decisions are taken subjectively; limit the number of ‘inappropriate referrals’; identify those most at risk of radicalisation; and ensure that the programme's aims are prioritised (Fisher et al., 2020). Although, this research also noted that some of the criteria had ‘subjective wording’ (Fisher et al., 2020, p. 27).

####### Multi‐agency client assessment

Fourteen studies examined how multi‐agency working might facilitate or inhibit client assessment. Eight studies examined how actual or simulated multi‐agency case conferences or other meetings functioned (Eijkman & Roodnat, 2017; Hofinger & Schmidinger, 2017; Sträter & Stuppert, 2019; van de Weert & Eijkman, 2020; Pettinger, 2020a; Piltch‐Loeb et al., 2021; Thompson & Leroux, 2022; Solhjell et al., 2022), and eight examined how multi‐agency working arrangements more broadly might serve as a facilitator or a barrier to assessment (Eijkman & Roodnat, 2017; Webster et al., 2017; Disley et al., 2016; Pettinger, 2020b; Førde & Andersen, 2021; Sizoo et al., 2022; Mattsson, 2021; Hellevik et al., 2022).^6^ Overall, the risk of bias within these studies was low, with most of the 13 studies (*n* = 9) assessed using the CASP qualitative assessment tool scoring positively on nine of the ten domains. The sole study assessed using the EPHPP tool was assessed as being of moderate quality. Overall, the strength of evidence relating to multi‐agency client assessment was relatively strong.

######## Multi‐agency collaboration

Eight studies highlighted the importance of strong multi‐agency collaboration when assessing clients (Eijkman & Roodnat, 2017; Webster et al., 2017; Disley et al., 2016; Pettinger, 2020b; Førde & Andersen, 2021; Mattsson, 2021; Sizoo et al., 2022; Hellevik et al., 2022). These studies identified effective collaboration between partners as helping practitioners make accurate assessments of clients and/or for reducing potential errors in assessment. Working arrangements such as the UK's **MAPPA** framework were seen as potentially facilitating this type of multi‐agency partnership (Webster et al., 2017; Disley et al., 2016). However, a key barrier related to difficulties in obtaining information from partners from security or policing (Eijkman & Roodnat, 2017; Disley et al., 2016), or ensuring an efficient flow of information between multi‐agency partners (Mattsson, 2021).

######## Using multi‐agency case conferences to assess clients

Eight studies presented data relating to the factors that influence how case conferences and other types of multi‐agency meetings are implemented in the context of client assessment, and how multi‐agency assessments are used (Eijkman & Roodnat, 2017; Hofinger & Schmidinger, 2017; Sträter & Stuppert, 2019; van de Weert & Eijkman, 2020; Pettinger, 2020a; Piltch‐Loeb et al., 2021; Thompson & Leroux, 2022; Solhjell et al., 2022).^7^

Case conferences are facilitated by systems that support communication between stakeholders; enable information sharing and service mapping; facilitate coordination between partners; work within appropriate legal frameworks; and ensure the availability of risk assessment tools (Piltch‐Loeb et al., 2021). Piltch‐Loeb et al. (2021) identify seven factors believed to facilitate client assessment: ‘allied professionals, system/protocols, coordination, interagency, communication, resources/services, and education’ (p. 131).

Trust and strong relationships between partners are key facilitators of case conferences (Thompson & Leroux, 2022; Solhjell et al., 2022). Trust‐building and relationship building can be supported by having an established team with a clear mandate, and strong leadership or the presence of an effective coordinator; working together over time and having regular meetings; and establishing personal relationships characterised by familiarity and reciprocity (Thompson & Leroux, 2022; Solhjell et al., 2022). The commitment and personalities of practitioners are also important in building trust (Thompson & Leroux, 2022). When such facilitators are present, case conferences can be an appropriate forum for discussing and sharing competing perspectives on the risks posed by individual clients (Hofinger & Schmidinger, 2017, p. 147).

Barriers to implementing case conferences are inconsistent implementation, subjectivity, bias, time, and power imbalances between stakeholders. Conferences have been found to operate differently according to the size of the municipality in the Netherlands (Eijkman & Roodnat, 2017), and can reflect variations in local practice in the UK (Pettinger, 2020a). Studies in both countries (Pettinger, 2020a; van de Weert & Eijkman, 2020) have identified concerns that individuals who are not at risk of radicalisation are wrongly adopted as clients based on low and subjectively applied thresholds which are used to guide case adoption decisions. Research in the UK also highlights that adoption thresholds can vary across regions (Pettinger, 2020a).^8^

A lack of faith in the ability of individual stakeholders and case conferences to accurately assess threat was identified as a barrier to implementation (van de Weert & Eijkman, 2020). This was partly informed by concerns over how information provided by partners was used. Although client assessments were based on information derived from multiple sources, including schools and youth workers, where assessments were completed without further consultation with professionals with direct experience of the potential client, there were concerns that not all the relevant information was assessed adequately or objectively, and that practitioners may overestimate their own expertise when assessing risk (van de Weert & Eijkman, 2020).

Unconscious bias may be a barrier to decision‐making (Pettinger, 2020a; van de Weert & Eijkman, 2020). Research in the UK highlighted how broader discourses that wrongly associate Islam with extremism can influence how practitioners interpret the views and behaviours of Muslims in ways that may lead to Muslims being disproportionately viewed as at risk of radicalisation (Pettinger, 2020a). Research in the Netherlands noted how practitioners may be impacted by ‘confirmation bias’, focusing on evidence that confirms pre‐existing assumptions, and overlooking that which does not (van de Weert & Eijkman, 2020). The risk of such bias may be influenced by the ‘one‐sided focus on religious (Islamic) extremism and Jihadism that so far has dominated the courses on detecting radicalization’ (van de Weert & Eijkman, 2020, p. 503) used to train practitioners in the Netherlands at the time of this research.

Excessive bureaucracy, and hierarchical struggles can generate barriers to building relationships that support case conferences (Solhjell et al., 2022; Thompson & Leroux, 2022).^9^

Power differentials between stakeholders can influence decision making processes when those in a higher position of authority (i.e., the police or the justice department) are ‘assumed to be correct and are, thus, insufficiently challenged (if at all) by the people around them’ (van de Weert & Eijkman, 2020, p. 501), something known as ‘authority bias’. It can also be challenging to obtain relevant information from the police and other security actors that might be useful in informing client assessments (Thompson & Leroux, 2022; Solhjell et al., 2022), and engage relevant agencies, particularly in countries where CVE work is less well developed (Piltch‐Loeb et al., 2021); points which are discussed in more detail in Section 4.2.6. Finally, there may be scheduling challenges, as it can be difficult to find a time and date that works for every actor involved in case management (Sträter & Stuppert, 2019, p. 20).

####### Risk and needs assessment (RNA) tools

Twelve studies discussed factors which facilitated or acted as barriers to the implementation of risk and needs assessment tools during the assessment stage of the case management process. The eligible studies were concerned with a variety of themes, covering the inconsistency in the use of RNA tools (Costa et al., 2021; Vandaele et al., 2022b; van der Heide & Schuurman, 2018); the actual and perceived utility of RNA tools (Webster et al., 2017; Inspector of Custodial Services NSW, 2018; Cherney, 2021; Disley et al., 2016; Costa et al., 2021; Stern et al., 2023; Corner & Pyszora, 2022; Cherney et al., 2022); the subjectivity of assessments completed with, and without RNA tools (Pettinger, 2020a, 2020b; Cherney, 2021; Corner & Pyszora, 2022; van de Weert & Eijkman, 2020); the role of expertise and experience in assessment (Webster et al., 2017; Disley et al., 2016; Cherney et al., 2022; Corner & Pyszora, 2022); and providing support to professionals involved in assessing clients (Webster et al., 2017; van der Heide & Schuurman, 2018; Corner & Pyszora, 2022). The risk of bias in relevant studies was assessed as being low based on the results of assessments conducted using the CASP qualitative assessment tool, with the majority of these studies scoring positively on eight (*n* = 4) or nine (*n* = 4) of the domains contained within this tool. Taken together, the strength of evidence relating to RNA tools can be considered robust based on both the quality and the quantity of eligible research identified through the literature searches.

######## Inconsistency in use

Three studies highlighted the inconsistent use of RNA tools. Costa et al.'s (2021) analysis of 14 exit programmes across Europe reported ‘57% of the programmes either do not implement RNA or lack structure’ (p. 19): six of these programmes used a named RNA tool, four used no RNA tool, and four used their own approach. Vandaele et al. (2022b) found that specific tools were rarely used when discussing individual cases across 14 multi‐agency meetings observed in different regions across Belgium, the Netherlands, and Germany.

Focusing on the **VERA‐2R** risk assessment tool, one study found that inconsistency in use was in part explained by practitioners' uncertainty about its ‘day‐to‐day’ applicability, and a perception that it required too much information, making it time consuming to use (van der Heide & Schuurman, 2018). Relatedly, another study concluded that practitioners would benefit from being provided with more information about the importance of using RNA tools during the client assessment stage (Costa et al., 2021).

######## Perceived utility of tools

Eight studies examined the actual and perceived utility of RNA tools (Webster et al., 2017; Inspector of Custodial Services NSW, 2018; Disley et al., 2016; Costa et al., 2021; Cherney, 2021; Cherney et al., 2022; Stern et al., 2023; Corner & Pyszora, 2022). These studies examined specialist tools that were specifically developed for assessing extremist cohorts, and the applicability of less specialised tools used with other cohorts to counter‐radicalisation work.

The benefits of specialist RNA tools in facilitating risk assessments were emphasised by two studies that examined **TRAP‐18**, a tool developed to assist in assessing the risk of lone actor terrorism (Corner & Pyszora, 2022); and the **Structured Risk Guidance (SRG)** for extremist offenders that was previously piloted with offenders in England before being revised to become the Extremism Risk Guidance 22+ (ERG 22+) in 2012 (Webster et al., 2017). Users interviewed in both studies were generally positive about the tools, but identified several improvements.

Focusing on the Australian context, Corner and Pyszora (2022) found that experts and users believed that the **TRAP‐18** represented the risks associated with lone actor terrorism and facilitated risk assessment through defined risk factors which practitioners felt able to operationalise. There was ‘collective agreement’ that the tool did not overwhelm users with a large number of factors and was more manageable than other assessment tools used in Australia. However, some barriers were identified, including concerns that the tool incorporated some factors that were potentially problematic or irrelevant, and that some risk, and many protective factors, were missing. A further barrier related to the limited information provided to interpret patterns of risk factors, and to explain how the tool could be used to inform case management. Recommendations to extend the tool's applicability included expanding the risk factor definitions; simplifying the language; improving the explanations of why different factors are relevant; and including prompt questions and ‘data gathering avenues’ (p. 12).

In England, Webster et al. (2017) concluded that the piloting of the **SRG** in English prisons had provided a ‘robust’ method of assessing extremist offenders and those at risk. It facilitated risk assessments by providing clarity over procedural assessment processes for staff; legitimising decisions relating to risk management; increasing the efficacy of assessors; and improving partnership working. Offenders interviewed for this study also suggested that their relationships with staff had improved, and that they were increasingly willing to engage in positive change since the pilot started, although the extent to which this was specifically linked to the use of the SRG is difficult to determine.

A number of potential improvements to the SRG were suggested. Recommendations included making offender eligibility criteria clearer; refining assessor eligibility criteria; extending training eligibility; and raising the profile of the SRG. Recommendations relating to delivery included revisiting the time and resources required to complete an assessment using the SRG; reviewing overlapping items; providing support for assessors; and enhancing partnership working (Webster et al., 2017, p. 4).

Five studies highlighted the barriers to risk assessment caused by the use of non‐specialist tools when working with terrorist or radicalised offenders, primarily because they are less able to identify the specific risks and criminogenic needs relevant to this cohort (Webster et al., 2017; Inspector of Custodial Services NSW, 2018; Cherney, 2021; Disley et al., 2016; Stern et al., 2023). For example, the **LSI‐R** risk assessment tool was considered likely to assess radicalised offenders as low risk because they did not reflect the pattern of risk factors, such as drug use or an offending history, more common in non‐terrorism offenders (Cherney, 2021). However, research also highlighted how practitioners might override this tool if necessary, such as when intelligence that was not accounted for within the LSI‐R became available (Cherney, 2021).^10^

Probation officers in the US similarly reported regularly overriding the results of assessments completed using a generic, non‐specialised RNA tool – **The Post‐Conviction Risk Assessment (PCRA)** – that was used to assess the recidivism risk of extremist offenders. Overriding these assessments aimed to ‘compensate for their perception that the PCRA, designed to evaluate the risk of recidivism for common criminals, would underestimate the risks posed by extremist offenders’ (Stern et al., 2023, p. 10). Respondents to this study suggested that the tool may be less accurate for some forms of extremism, such as white supremacists and anti‐government extremists, and called for the integration of extremism‐specific indicators into the PCRA, or the introduction of assessment tools that were specifically designed for extremist cohorts.

One study emphasised the benefits of triangulating the results of assessments from RNA tools with other data to facilitate decision making around risk (Cherney et al., 2022). The importance of triangulating data is also discussed in the discussion of monitoring and evaluation tools.

######## Subjectivity

Four studies examined how subjectivity influenced client assessment in practice: three of which looked at how specific RNA tools were used (Pettinger, 2020a, 2020b; Cherney, 2021; Corner & Pyszora, 2022); and one that discussed subjectivity in relation to client assessment without reference to any specific tool or process (van de Weert & Eijkman, 2020).^11^

Subjectivity in the way assessments were carried out was typically considered a barrier to effective client assessment. Two studies emphasised that the use of structured risk assessment tools is not sufficient to overcome such subjectivity (Pettinger, 2020a, 2020b; Cherney, 2021). Studies in the UK and the Netherlands point to the subjectivity inherent in how individual practitioners assess clients (Pettinger, 2020a, 2020b; van de Weert & Eijkman, 2020). For example, in the UK, the lack of consistency in assessments meant that the threshold for adopting an individual case varied across different areas, and that ‘a large number’ of cases did not meet the official threshold for case adoption as outlined in official UK guidance (Pettinger, 2020a, p. 5). This inconsistency and an inability to differentiate those at greatest risk of engaging in extremist violence from those expressing intolerant but not extremist views has led authors to raise concerns that a proportion of those being offered support through case management interventions may not actually be at risk of radicalisation, or in need of support (Pettinger, 2020a; van de Weert & Eijkman, 2020).

However, a significant proportion of secondary intervention clients are, by definition, likely to be at earlier stages of radicalisation, and may not pose an immediate security risk. This is reflected by Schroer‐Hippel's (2019) analysis, which highlighted how the vast majority of clients supported by **KOMPASS** ‘do not involve manifest radicalisation, but rather the first signs of such a development’, with only 3% presenting with ‘threatening signs of radicalisation’ (p. 35). The ability to use risk assessment tools more subjectively was not therefore always viewed negatively, with some practitioners emphasising the importance of drawing on professional judgement when using these tools and assessing clients (Pettinger, 2020a; Corner & Pyszora, 2022). However, others suggested this approach was contra to the way they typically approached risk assessment processes and had the potential to result in lower confidence in the process and outcome of the assessment (Corner & Pyszora, 2022).

######## Role of expertise and experience

Four studies discussed how risk assessment is facilitated by practitioners with specific expertise and/or highlighted issues that can emerge when such expertise is lacking (Webster et al., 2017; Disley et al., 2016; Cherney et al., 2022; Corner & Pyszora, 2022).^12^ Professional judgement and experience with terrorist cases was considered an important facilitator when assessing the risk of recidivism (Webster et al., 2017; Disley et al., 2016). Experience was also considered relevant to enhancing practitioners' ability to identify and assess disguised compliance (Cherney et al., 2022) and interpret the presence and relevance of risk factors (Corner & Pyszora, 2022).

The implementation of risk assessment tools is facilitated when it takes account of differences in the knowledge and experience of assessors from different disciplines or agencies, for example police or mental health professionals (Corner & Pyszora, 2022). Training is therefore important in developing skills and confidence in ways which support risk assessment processes (Webster et al., 2017).

######## Organisational support for assessors

Three studies identified organisational practices believed to improve the ability of practitioners to accurately assess clients, including the use of multiple assessors (Webster et al., 2017; van der Heide & Schuurman, 2018; Corner & Pyszora, 2022); and providing formal and/or informal support to practitioners involved in assessing clients (Webster et al., 2017). Partnership working, including effective and efficient information‐sharing, was also identified as facilitating assessment (Webster et al., 2017).

###### Stage 3: Case planning

Data relating to the case planning stage was identified in five studies. Two tools related to case planning were identified: intervention planning tools and case conferences. Whilst these tools were also used to inform the client assessment and monitoring stages of the case management process, this section examines research relating to their use in case and intervention planning. The evidence‐base relating to case planning cannot be considered robust given that it is based on only five studies. However, the risk of bias within these five studies was very low, with four scoring positively on eight (*n* = 3) or nine (*n* = 1) of the domains in the CASP assessment tool.

####### Intervention planning tools

Three studies presented evidence related to the use of case planning tools (Cherney, 2021; Inspector of Custodial Services NSW, 2018; Corner & Pyszora, 2022). This research analysed the use of the LSI‐R, a non‐terrorism specific RNA tool used in Australia (Cherney, 2021; Inspector of Custodial Services NSW, 2018)^13^ and TRAP‐18 (Corner & Pyszora, 2022). Where the assessment process informs case planning, these tools can help facilitate appropriate levels of monitoring and reporting (Cherney, 2021). However, case plans developed using these tools were not always considered ‘meaningful’ to clients or to practitioners (Inspector of Custodial Services NSW, 2018). A barrier to the implementation of case planning is the lack of consistency between case plans and the results of assessments conducted using RNA tools (Cherney, 2021). The lack of guidance in some RNA tools to support case formulation and inform ongoing case management creates a further barrier to implementing case planning (Corner & Pyszora, 2022).

####### Case conferences

Two studies examined how case planning was conducted during case conferences based on observations of actual (Vandaele et al., 2022b) or simulated (Solhjell et al., 2022) meetings. These meetings served multiple functions that spanned assessment, planning, and monitoring, and so the data discussed here was not always specific to planning. However, the analysis presented in these studies has specific relevance to this stage of case management.

Case conferences have a number of roles beyond case planning which influenced the amount of time spent on this aspect of the case management process. Vandaele et al. (2022b) found that the time spent on case management in 14 multi‐agency meetings in Belgium, the Netherlands and Germany varied from 10% to 88%. There was also much variation in how these meetings were delivered, including whether meetings were formally chaired (and by whom); whether they were organised around a formal agenda; whether they adopted a formal or informal approach; and whether any tools or thinking frameworks were used (Vandaele et al., 2022b). Further regional variations in implementation are discussed in Section 4.2.6.

A SWOT (strengths, weaknesses, opportunities, and threats) analysis of these case conferences pointed to a number of strengths that were seen as facilitating effective multi‐agency meetings, and barriers that might limit their effectiveness (Vandaele et al., 2022b). Facilitators included trust between partners; high motivation; sufficient expertise being present; the use of structured meetings guided by an agenda and a neutral meeting chair; and appropriate information sharing between partners (i.e., a good balance between ‘nice to know and need to know’ information) (Vandaele et al., 2022b). In a separate chapter based on the same research, Vandaele et al. (2022a) highlighted a number of additional facilitators of efficient meetings, including a positive working environment, and horizontal relationships that were marked by equality.

Additional facilitators included having an established team, with a clear mandate and leadership; working together over time and having regular meetings; and establishing personal relationships marked by familiarity and reciprocity (Solhjell et al., 2022).

Barriers to effective case conferences included the absence of clear, common goals; a shortage of resources including time, finances, and people; and partners dominating discussions and/or acting in their own, or their organisation's self‐interest (Vandaele et al., 2022b). High levels of bureaucracy; information‐sharing challenges; and hierarchical struggles were identified as potential barriers to trust building (Solhjell et al., 2022). Other facilitators and barriers to multi‐agency working more broadly are discussed in detail in Section 4.2.6.

###### Stage 4: Delivery and implementation

Twenty‐eight studies presented empirical evidence relating to the delivery of intervention plans, representing a larger number of studies than for any other stage of the case management process. The risk of bias within these studies was also assessed as being low based on the CASP assessments, with 21 of the 27 relevant studies assessed using this tool scoring positively on eight (*n* = 7), nine (*n* = 13), or all ten (*n* = 1) of the domains in this tool. Overall, the strength of evidence relating to this stage was high based on both the quality and quantity of the research.

These studies focused on three central themes: tailoring intervention goals and services; practitioner characteristics and support; and practitioner supervision and quality assurance.

####### Tailoring intervention goals and services

Reflecting its centrality to case management process, nineteen studies provided empirical data relating to the tailoring of intervention plans and goals to the specific needs of individual clients (Lukas, 2006; Spalek et al., 2010; Becker et al., 2014; Christensen, 2015; Schuurman & Bakker, 2016; Eijkman & Roodnat, 2017; Costa et al., 2021; AEF, 2018; van der Heide & Schuurman, 2018; Haugstvedt, 2019, 2022; Orban, 2019; Fisher et al., 2020; Cherney, 2020, 2022; Cherney & Belton, 2021a, 2021b; Raets, 2022; Vandaele et al., 2022a; Stern et al., 2023). In line with studies relating to this stage more broadly, the risk of bias in these studies was low, with three‐quarters of relevant studies (*n* = 15) assessed using the CASP qualitative assessment tool scoring positively on eight (*n* = 6), nine (*n* = 8), or ten (*n* = 1) of the included domains.

Four studies specifically identified the tailoring of intervention plans as a key facilitator of the case management process (Lukas, 2006; Becker et al., 2014; Schuurman & Bakker, 2016; Raets, 2022). For example, Schuurman and Bakker (2016) reported that tailoring was found to both work towards addressing the needs of individual clients of the **Team TER reintegration programme**, and to support the process of trust building between the individual and the practitioner working with them. This process of trust building was also considered important in helping to identify deceptive behaviour, a point discussed in more detail in the examination of false and disguised compliance in Section 4.2.6.

A combination of formal services and informal forms of support are described as helpful in developing the trust and motivation of clients in ways which facilitate the delivery of case management interventions, particularly in early interactions between clients and practitioners (Spalek et al., 2010; Christensen, 2015; Orban, 2019; Haugstvedt, 2019; Cherney & Belton, 2021a; Cherney, 2022; Raets, 2022). These services speak to particular needs which can be shared across clients, or be specific to the individual (Cherney & Belton, 2021a, 2021b).

The process of tailoring goals is enabled by ensuring aims are realistic and/or motivational. To support this process, some interventions and practitioners specifically work towards client‐directed goals (AEF, 2018; Haugstvedt, 2019, 2022; Raets, 2022), whilst others set different goals for different clients based on what is deemed realistic given the client's unique circumstances (van der Heide & Schuurman, 2018; Eijkman & Roodnat, 2017). This approach is believed to prevent disappointment by ensuring goals are achievable (Eijkman & Roodnat, 2017). Efforts to tailor services are further supported by considering services in relation to intervention goals and the causes of individual radicalisation (Cherney & Belton, 2021b).

Efforts to tailor interventions can be facilitated by identifying services that work across different levels of a client's social ecology. Alongside individual, or micro‐level, goals such as tackling mental health needs or finding employment, intervention plans can also work towards goals at the meso level, such as repairing family relationships; at the exo‐ or community/social level, for example developing prosocial relationships and supports; and at the macro‐level, for example by seeking to challenge risk factors existing at the societal or structural level. A range of interventions adopted a multi‐level approach when identifying, and working towards, client‐specific goals (e.g., Christensen, 2015; Schuurman & Bakker, 2016; Fisher et al., 2020; Vandaele et al., 2022a). Examples included **STRIVE** in Kenya, which seeks to address factors across different levels of analysis by tackling structural factors; addressing group‐based dynamics; countering enabling factors; and reducing individual incentives (Fisher et al., 2020).

Work to address multiple layers of someone's social ecology can be facilitated through both one‐to‐one activities, such as working with a mentor, and group work (Costa et al., 2021). Although sometimes challenging, engagement or reconciliation with the client's family can also be important (e.g., Schuurman & Bakker, 2016; AEF, 2018; Cherney, 2020, 2022; Costa et al., 2021; Raets, 2022; Vandaele et al., 2022a; Disley et al., 2016).^14^ However, interventions may struggle to engage with families and peers of clients, even when such engagement is seen as crucial (Cherney, 2020).

Appropriate sequencing of different components of intervention plans can be an important aspect of tailoring. If too many services are delivered at the same time there is a risk that the client is overloaded, and the plan becomes counter‐productive (Eijkman & Roodnat, 2017).

####### Practitioner characteristics and approaches

Twenty studies considered the characteristics of practitioners who are typically mentors or other staff working on a one‐to‐one basis with clients as part of broader case management processes (Lukas, 2006; Spalek et al., 2010; Christensen, 2015; Schuurman & Bakker, 2016; Hofinger & Schmidinger, 2017; AEF, 2018; van der Heide & Schuurman, 2018; Möller & Neuscheler, 2018; Weeks, 2018; Schuhmacher, 2018; Orban, 2019; Schroer‐Hippel, 2019; Haugstvedt, 2019; Fisher et al., 2020; Pettinger, 2020a; Mattsson, 2021; Costa et al., 2021; Cherney & Belton, 2021a; Stern et al., 2023; Cherney, 2022). Again, the strength of evidence relating to this theme was assessed as being high based on the quality and quantity of research, with fifteen of these twenty studies scoring positively on eight or more domains in the CASP.

Ten studies discussed the process of matching practitioners with clients, or of employing practitioners with similar backgrounds to clients, a process which was considered an important facilitator of engaging and building relationships and delivering support to them (Lukas, 2006; Spalek et al., 2010; Christensen, 2015; Costa et al., 2021; Möller & Neuscheler, 2018; AEF, 2018; Orban, 2019; Schroer‐Hippel, 2019; Fisher et al., 2020; Mattsson, 2021). A prominent example of this approach is **EXIT Sweden**, which employs former right‐wing extremists as coaches on the assumption that they will be best placed to support young people in exiting an extremist scene that they themselves were once part of (Christensen, 2015).^15^

A process evaluation of the **Norwegian Mentoring System (NMS)** in the custodial system elaborates on the importance of matching of mentors and mentees (Orban, 2019). The factors that were considered important included language skills (so that conversations could be held in the client's mother tongue); subject knowledge (in particular regarding religion, politics and society); and the mentors adopting an approach that was different to that of prison officers, social workers, psychologists and family members and relatives. Mentees explained that a good mentor was an effective listener and discussant who was dedicated and believed in them and their potential to contribute to society in the future. Less effective matching was informed by a perception the mentor lacked sufficient knowledge or did not use the right approach(es).

The **STRIVE** counselling programme in Kenya selected mentors from local communities who were older and who had some shared experiences with mentees (Fisher et al., 2020). This helped facilitate the implementation of the intervention by providing more relatable mentors able to act as role models helping to motivate and inspire clients (Fisher et al., 2020). In the UK, a study on a mentoring programme found matching the identities of clients and mentors was often beneficial, although this was not always considered necessary or appropriate, for example if there were differences in age, ‘self‐understanding [and] self‐positioning in relation to wider social, political and other processes and structures’ (Spalek et al., 2010, p. 28). The implementation of exit programmes across Europe was facilitated by using case workers who were able to engage effectively with the individual, and demonstrate empathy and authenticity (Costa et al., 2021). Whilst a good fit between clients and practitioners working for the **Counselling Centre Hesse** in Germany was seen in comparable personalities, language, and religion, and helped to build trust and relationships (Möller & Neuscheler, 2018).

Maintaining continuity in the relationship between practitioners and clients helped facilitate the delivery of interventions (Lukas, 2006; van der Heide & Schuurman, 2018). An individualised aftercare programme for post‐release offenders in Germany used the same staff as a group intervention offered to the same offenders whilst in prison (Lukas, 2006). An evaluation found that ‘almost all participants’ who took part in this aftercare programme explicitly stated that they only agreed to participate because they had already worked with, and trusted, the staff assigned to them (Lukas, 2006, p. 52). Similarly, van der Heide and Schuurman (2018) note ‘the traditional distinction between assessment and supervision work is suspended’ in the Dutch **Team TER** programme, with Team TER staff doing both as this was believed to develop rapport and support the assessment of clients (p. 205).^16^ Overall, these studies emphasise the importance of developing something akin to a ‘therapeutic relationship’ (Stern et al., 2023) to motivate clients to participate in interventions, and to change.

However, asking practitioners to perform this dual function can create challenges. Hofinger & Schmidinger's (2017) evaluation of the **DERAD** intervention in Austria concluded that practitioners' dual role as both an ‘assessor’ for prisons and as a ‘religious social worker’ for inmates created challenges for both functions. Assessments were not always clear, and often diverged from the perceptions of prison management, whilst there was no ‘protected, confidential framework for the actual deradicalisation work’ (Hofinger & Schmidinger, 2017, p. 147). These findings draw attention to broader challenges relating to multi‐agency working across different cultures which are discussed in more detail in Section 4.2.6.

Nine studies found that practitioners' flexibility and commitment to their client, for example through their willingness to make themselves available outside of standard working hours, invest large amounts of time, and respond promptly to any requests, helped facilitate case management interventions (Christensen, 2015; Schuurman & Bakker, 2016; van der Heide & Schuurman, 2018; Schuhmacher, 2018; Möller & Neuscheler, 2018; AEF, 2018; Haugstvedt, 2019; Orban, 2019; Cherney, 2022). Two further studies emphasised the importance of practitioners spending time with clients to foster rapport or a therapeutic relationship as being an important facilitator in intervention delivery (Weeks, 2018; Cherney & Belton, 2021a). The importance of this implementation factor is most clearly illustrated by Cherney and Belton (2021a)'s analysis of client progress measures. This study found ‘a positive relationship between program intensity and the level of progress clients made during their participation’ in Intervention 1 and 2 in Australia, suggesting that ‘more frequent contact … with intervention staff/service providers seemed to make a difference’ (Cherney and Belton (2021a, p. 14).

However, the amount of time and flexibility required to deliver counter‐radicalisation work can create barriers to implementation. Three studies highlighted how the intensive nature of this work can be challenging due to the lack of a ‘definitive indicator of when you have done enough’ (AEF, 2018, p. 36); potential overwork and stress (Schuurman & Bakker, 2016, p. 75; van der Heide & Schuurman, 2018, p. 214); and tensions with managers who may not see the value of spending more time with terrorist offenders than would normally be spent with other clients (Schuurman & Bakker, 2016, p. 75).

There were mixed findings regarding the utility of practitioners delivering their work in different ways. In some cases, a flexible approach, responsive to the client, was seen as a strength (Christensen, 2015). In other research which assessed interventions without set processes of engaging with clients, and where mentors played a greater role in determining the shape of sessions, inconsistency in how individual mentors approached this work had the potential to raise issues related to quality assurance (Pettinger, 2020a).^17^ A potential outcome of such inconsistency is disagreement between practitioners over how to engage with the client (Spalek et al., 2010; Christensen, 2015). This point is discussed in the next section.

####### Practitioner supervision and quality assurance

Thirteen studies presented data that illustrated how effective supervision, oversight and quality assurance facilitated implementation (Spalek et al., 2010; Becker et al., 2014; Christensen, 2015; Schuurman & Bakker, 2016; Hofinger & Schmidinger, 2017; van der Heide & Schuurman, 2018; Schuhmacher, 2018; AEF, 2018; Orban, 2019; Haugstvedt, 2022; Pettinger, 2020a; Haugstvedt & Gunnarsdottir, 2023; Cherney et al., 2022; Sizoo et al., 2022). This finding was most clearly emphasised by Haugstvedt (2022), who reported that social workers had an ‘outspoken need for supervision and professional guidance’ (p. 172). Whilst the evidence base for this theme was slightly less developed than other themes relating to the delivery stage, the quality of research remained high, with eight of the 13 relevant studies scoring positively on eight or more of the critical domains contained within the CASP tool.

Only one study specifically examined the use of a formal quality assurance process (Spalek et al., 2010). A **Mentor Selection Panel** involving a structured application and recruitment process sought to mitigate the risks believed to accompany less structured and systematised approaches to engaging with those at risk of radicalisation (Spalek et al., 2010). This type of formalised supervision and quality assurance processes can make it possible to identify those unsuited to working with clients, something that might be particularly important when former clients of interventions become coaches, as is the case in **EXIT Sweden** (Christensen, 2015).

Factors that helped support those delivering interventions included the use of two case managers, which provided self‐reported benefits for staff including a greater sense of safety; oversight of one another's work and the provision of alternative perspectives and input; and helping to assess the authenticity of clients (van der Heide & Schuurman, 2018; Schuhmacher, 2018; Cherney et al., 2022). Organisational, managerial and peer support can help practitioners work through the emotional impacts of ‘highly challenging meetings and sessions with clients who may have strong views on society that may contradict their own’ (Haugstvedt, 2022, p. 171), and cope with the emotional demands of working with radicalised clients or clients at risk of radicalisation (Orban, 2019; Haugstvedt & Gunnarsdottir, 2023).

Formal debriefing sessions, supervision of practice, group discussions and peer support, and engaging with psychologists were other mechanisms that may be used to support practitioners in delivering support to clients, and to introduce different perspectives on cases (Haugstvedt, 2022; Spalek et al., 2010 Christensen, 2015; Cherney et al., 2022; van der Heide & Schuurman, 2018; Schuurman & Bakker, 2016). Peer support may be important ‘where there are no comprehensive guidelines or professional history of what to do and how to collaborate with other agencies, such as the police and security service’ (Haugstvedt, 2022, p. 172).

Experience and expertise accumulated over a period of time working in the field of countering radicalisation to violence can facilitate the development of independently functioning teams that are well placed to run interventions (van der Heide & Schuurman, 2018; AEF, 2018).

By contrast poor working relationships with managers (Schuurman & Bakker, 2016) or ineffective management structures (Sizoo et al., 2022); a lack of communication (and miscommunication) between different actors involved in the case management process (Hofinger & Schmidinger, 2017); and the absence of a clearly defined ‘competency framework’ to assess the suitability of practitioners (Pettinger, 2020a) were identified as potential barriers to implementation.

###### Stage 5: Monitoring and evaluation

Sixteen studies presented empirical data relating to monitoring and evaluating progress. These studies included research on specific client assessment tools; the use of case files and case notes to track change; case conferences; and less structured forms of qualitative data.

####### Client assessment tools

Nine eligible studies examined the use (or lack thereof) of client assessment tools in monitoring client change and/or progress towards intervention goals (Inspector of Custodial Services NSW, 2018; van der Heide & Schuurman, 2018; Weeks, 2018; Costa et al., 2021; Cherney & Belton, 2021a, 2021b; Cherney et al., 2022; Raets, 2022; Stern et al., 2023). Whilst this represented a smaller number of studies than those examining the use of this tool during client assessment (*n* = 12), these studies were considered similarly robust, with half of the relevant studies (*n* = 4) that were assessed using the CASP tool scoring positively on nine domains.

Structured assessment tools can monitor client progress in ways that can help facilitate case management interventions (Costa et al., 2021; Cherney & Belton, 2021a, 2021b; Stern et al., 2023). When triangulated with other sources of data, formalised risk assessment tools have the potential to support the detection of false compliance (Cherney et al., 2022).

Barriers to effective client assessment include similar issues of subjectivity and bias to those identified in the earlier discussion of client assessment tools (Cherney & Belton, 2021b). Inconsistency in how practitioners monitor the progress of clients can create an implementation barrier. It is harder to develop a consistent assessment of progress when government agencies use, for example, ‘a risk assessment model based on recidivism and psychometric tools’, and non‐government mentors or intervention providers use ‘more subjective assessments based on positive reintegration in society’ (Weeks, 2018, p. 524).

Where assessment tools are not used to inform longitudinal efforts to interpret risk, the opportunities for practitioners to track recidivism risk or other progress measures may be reduced (van der Heide & Schuurman, 2018; Raets, 2022). The failure to complete risk assessments before the commencement of an intervention plan can also lead to an absence of baseline data against which to assess change (Inspector of Custodial Services NSW, 2018).

####### Case files and case notes

Seven studies provided data relating to the use of case notes or other documentation when working with, and assessing the progress made by, clients (Spalek et al., 2010; Schuhmacher, 2018; Cherney & Belton, 2021a, 2021b; Cherney, 2022; Raets, 2022; Cherney et al., 2022). Whilst this represents a small number of relevant studies, two‐thirds of those studies assessed using the CASP tool (*n* = 4) scored positively on nine of the ten domains. Several important findings emerged from these studies which warrant discussion.

Case notes have the potential to provide ‘a rich source of data’ (Cherney & Belton, 2021b, p. 630) that help interventions track and monitor client progress over time (Cherney & Belton, 2021a, 2021b; Cherney, 2022). They can capture factual information relating to an individual's engagement in the intervention as well as practitioner feedback on the quality of client engagement and on client behaviour and attitudes with the potential to help detect false compliance (Cherney & Belton, 2021a; Cherney et al., 2022).

Inconsistency in the quality and content of case notes can reduce their utility (Cherney & Belton, 2021a, 2021b). Where they do not capture sufficient information, it can be harder to assess progress and evaluate the effectiveness of interventions (Raets, 2022). Systems and processes able to record specific types of information in case notes can help address this issue, whilst allowing multiple practitioners to contribute to case files can reduce the possibility of biased assessments from single members of staff (Cherney & Belton, 2021a, 2021b).^18^

A potential barrier to the use of case notes is the risk they might negatively impact one‐to‐one interactions between a client and their mentor (Spalek et al., 2010). Taking notes to inform case files in meetings may have the potential to decrease trust (Spalek et al., 2010). Ethical and legal restrictions in some countries may also prevent the recording of some information that might be desired by some stakeholders, such as religious orientation (Schuhmacher, 2018).^19^

####### Case conferences

Five studies examined the use of case conferences and other meetings to monitor clients (Cherney et al., 2022; Thompson & Leroux, 2022; Möller & Neuscheler, 2018; Haugstvedt, 2022; Pettinger, 2020b). This means that a smaller number of studies have examined the use of case conferences for monitoring and evaluation than for client assessment, which limits the conclusions that can be drawn. However, a number of useful lessons can be identified from this relatively small body of research. This research found that case conferences enabled a process of ‘plausibility checking’, bringing together multi‐disciplinary teams with different perspectives to interpret and monitor client progress through information sharing, and discussion of any observed indicators of change (or lack thereof) (Möller & Neuscheler, 2018; Haugstvedt, 2022; Cherney et al., 2022; Pettinger, 2020b), although this remained a subjective process (Pettinger, 2020b). Conferences also provide a forum to carry out case reviews and light‐touch, internal audits through which ‘the demographics and needs of program [sic] participants are reviewed on a semi‐regular basis by the case planning team to assess whether additional program partners are needed’ (Thompson & Leroux, 2022, p. 12).^20^

####### Less structured qualitative data

Five studies highlighted barriers and facilitators in relation to the use of less structured or more subjective forms of qualitative data. These studies highlighted how partners might disagree over which metrics are indicative of genuine change, or use different measures to monitor and evaluate client progress and outcomes (Weeks, 2018; van der Heide & Schuurman, 2018; Pettinger, 2020a; Cherney et al., 2022; Raets, 2022). Whilst this evidence base cannot be considered robust, the relevant studies were assessed as having a low risk of bias using the CASP tool, and can therefore be considered to have produced some relevant findings.

A lack of systematic approaches to monitoring and evaluating clients, and the use of subjective, qualitative criteria, such as ‘body language, tone, and facial expressions’ (Pettinger, 2020a, p. 10), can lead to disagreements between practitioners over levels of risk and progress (Pettinger, 2020a). Without formal metrics of progress, it is harder to resolve disagreements over, for example, whether a lack of non‐terrorism related recidivism is a relevant metric for counter‐radicalisation work  (van der Heide & Schuurman, 2018). However, practitioners have been found to follow ‘general principles’ (van der Heide & Schuurman, 2018), and even where formal metrics were not employed, considerable levels of consistency have been found in how mentors assess change (Weeks, 2018).

###### Stage 6: Transition and exit

The transition and exit stage of case management intervention was rarely discussed, and so the evidence base relating to this stage of case management remains underdeveloped. However, the risk of bias in the relevant studies was very low, with all ten studies included in this stage scoring positively on eight (*n* = 7) or nine (*n* = 3) of the ten domains in the CASP assessment tool. The findings relating to this stage can therefore be considered to be reasonably robust. Three eligible studies presented data relating to this element of the case management process (Lukas, 2006; Cherney, 2020; Vandaele et al., 2022a), whilst seven studies discussed challenges relating to monitoring clients after they had exited interventions or been released from prison on probation or parole (Möller et al., 2015; Weggemans & de Graaf, 2017; Schuhmacher, 2018; van der Heide & Schuurman, 2018; Cherney, 2021; Costa et al., 2021; Stern et al., 2023). One study reported that cases largely closed when expected, and in general, ‘were followed up well’ once case management had ended (Vandaele et al., 2022a, p. 70). Cherney (2020) emphasised the importance of maintaining continuity in support during the prerelease and release periods.

Barriers associated with the exit process include the difficulties of ending the relationship between practitioners and clients smoothly (Lukas, 2006) and fear over closing cases due to the potential consequences of making a wrong decision (Vandaele et al., 2022a).^21^ Weggemans and de Graaf (2017), van der Heide and Schuurman (2018), Cherney (2021), and Stern et al. (2023) also reported that there can be practical challenges in trying to monitor former offenders upon their release from prison due to their contacts, activities and movements being less tightly controlled than they were during their incarceration. The absence of post‐intervention monitoring or data on former clients can also be a barrier to effective case management working (Costa et al., 2021; Schuhmacher, 2018; Möller et al., 2015).

#### 4.2.6 The case management process

##### Implementation factors and moderators across the case management process (Objective 2b)

As well as research specific to the implementation of different stages of case management, a body of evidence spoke to those factors which influenced the implementation of the full case management process. Of the 47 studies eligible for this review, 41 provided empirical evidence relating to implementation factors, including facilitators and barriers, and 28 provided evidence relating to different moderators.

###### Implementation factors (Objective 2b)

This section describes the different factors that influenced the implementation of the case management process, that were not specific to individual stages or tools. The discussion examines the following facilitators and barriers: multi‐agency working, with sub‐themes on satisfaction with multi‐agency working covering information sharing; clarity of goals; and relationship; risk‐oriented logics covering approaches to tertiary and secondary interventions and issues linked to false compliance; public and policy pressure; the intensity of CVE work; resourcing; staff expertise; voluntary and mandatory approaches; and broader legislation.

####### Multi‐agency working

Every intervention identified across the eligible studies used some form of multi‐agency or multi‐disciplinary working. For some interventions, multi‐agency working was formally embedded into every stage of the case management process, and helped to inform intake, assessment, case planning, delivery, monitoring, and exit. Examples included Channel in the UK (Pettinger, 2020a, 2020b); in Australia, PRISM and Interventions 1 and 2 (part of the Australian National Diversion Program) (Cherney, 2018; Cherney, 2020; Cherney & Belton, 2021a, 2021b; Cherney, 2022); and ReDirect and FOCUS Toronto in Canada (Thompson & Leroux, 2022). For other interventions, multi‐agency working was more relevant to individual stages of the case management process, as evidenced by studies which specifically examined how multi‐agency networks undertook case planning and monitoring (e.g., Vandaele et al., 2022a), or how expert practitioners would be tasked with delivering specific components of intervention plans when required (e.g., Weeks, 2018; van der Heide & Schuurman, 2018).

A range of approaches to multi‐agency working supported these interventions. For example, whilst **FOCUS Toronto** and **ReDirect** were underpinned by the same ‘situation table model’, Thompson and Leroux (2022) highlighted key differences in how they operated. FOCUS Toronto did not deliver support itself, and instead used a ‘brokerage system between multi‐sectoral partners’, through which they would link clients to different forms of support. ReDirect hired a multi‐disciplinary team to deliver this support themselves. Several interventions used a hybrid of these two approaches, where support was primarily delivered by staff employed by the intervention – or, in the case of one German intervention, by individual schools (Sträter & Stuppert, 2019) – and external partners were tasked with delivering more specialised support when required (e.g., Christensen, 2015; Schuurman & Bakker, 2016).

A novel approach to multi‐disciplinary working is the *‘clearing team’* model used in German schools (Sträter & Stuppert, 2019) which aims to identify and support school pupils at risk of radicalisation. This process is coordinated by a ‘pedagogical specialist’, who is supported in the planning and implementation of tailored packages of support by a clearing team consisting of the head of the school, the head of the class, the clearing officer, and a school social worker.

Several studies examined broader issues relating to multi‐agency working. This included exploring how actors from different sectors collaborated when delivering CVE work (e.g., Haugstvedt & Tuastad, 2021; Sizoo et al., 2022), as well as examining how multi‐agency meetings operated in practice (e.g., Piltch‐Loeb et al., 2021; Vandaele et al., 2022b).

In total, 34 studies provided empirical evidence relating to how multi‐agency working operated in practice. Taken together, this research illustrated how multi‐agency working might be a facilitator, but in some cases a barrier to the effective implementation of case management. The strength of evidence relating to multi‐agency working can be considered robust based on both the quantity, and the quality of the relevant research. Over two‐thirds (*n* = 24) of these studies scored positively on eight or more of the ten domains within the CASP qualitative assessment tool, and almost half (*n* = 16) scored positively on nine or more of these domains.

######## The importance of multi‐agency working

Twenty‐three studies suggested that effective multi‐agency working supported implementation (Becker et al., 2014; Schuurman & Bakker, 2016; Eijkman & Roodnat, 2017; Webster et al., 2017; van der Heide & Schuurman, 2018; AEF, 2018; Disley et al., 2016; Möller & Neuscheler, 2018; Sträter & Stuppert, 2019; Fisher et al., 2020; Pettinger, 2020b; Hellevik et al., 2022; Vandaele et al., 2022a, 2022b) and/or identified multi‐agency working as important to case management delivery (Hofinger & Schmidinger, 2017; Weggemans & de Graaf, 2017; Schuhmacher, 2018; Haugstvedt, 2022; Piltch‐Loeb et al., 2021; Cherney, 2020; Sizoo et al., 2022; Stern et al., 2023; Raets, 2022; Thompson & Leroux, 2022). Whilst studies mainly focused on formal relationships, informal relationships and networks were also identified as being relevant (Weggemans & de Graaf, 2017; Schuhmacher, 2018; Vandaele et al., 2022a).

A number of implementation factors relating to multi‐agency working were identified: visibility; rules for information sharing; the clarity of intervention goals; ensuring the right partners are involved in partnerships; and the relationships between different partners. As noted in the previous discussion of client assessment and case planning, collaboration during multi‐agency case conferences and other stakeholder meetings was a further factor that influenced implementation.

######## Visibility to partners

Four studies highlighted the importance of interventions being visible and accessible to multi‐agency partners who may wish to refer into them (Schuurman & Bakker, 2016; van der Heide & Schuurman, 2018; AEF, 2018; Cherney, 2020). For example, the second evaluation of the **Team TER intervention** highlighted that the programme had become more visible internally and to external partners and was ‘widely recognized as having relevant expertise and has thus been able to occupy the central role in the Dutch reintegration framework that was originally envisioned’ (van der Heide & Schuurman, 2018, p. 214). Cherney (2020) reported that an increasing awareness of **PRISM** amongst community corrections staff led to an increased number of enquiries, whilst AEF (2018) reported that ‘practically all municipal professionals’ in the Netherlands interviewed for their evaluation of **Forsa** found the over‐arching National Support Centre for Extremism (LSE) to be ‘highly accessible and easy to contact’ (p. 19).

######## Information sharing

Four studies explicitly identified effective and efficient information sharing as a key facilitator of multi‐agency working (Webster et al., 2017; Thompson & Leroux, 2022; Solhjell et al., 2022; Vandaele et al., 2022a). However, fifteen studies also identified information sharing as a challenge within multi‐agency partnerships, most commonly in the context of collaborations between security actors and other actors, particularly those working in psychosocial or healthcare contexts. Two specific challenges resulted from disparities in how different partners might approach sharing information.

First, the less transparent working practices of the police and security agencies were found to inhibit them from sharing information on occasion (Weggemans & de Graaf, 2017; Eijkman & Roodnat, 2017; Disley et al., 2016; Cherney, 2021; Haugstvedt & Tuastad, 2021; Raets, 2022; Sizoo et al., 2022; Vandaele et al., 2022a; Kotzur et al., 2022; Stern et al., 2023). Second, some partners – particularly healthcare and social work – had strict confidentiality rules that inhibited sharing only to situations where there is imminent danger or risk and/or when informed consent is provided by the client (Spalek et al., 2010; Haugstvedt & Tuastad, 2021; Sizoo et al., 2022; Hellevik et al., 2022; Solhjell et al., 2022; Vandaele et al., 2022a). One further study reported on a programme in Norway's custodial system that limited the amount of information given to mentors about mentees to increase the mentor's safety and enhance levels of trust (Orban, 2019); mentors disagreed as to whether this was the best approach.

One mechanism with the potential to overcome barriers associated with information sharing between partners was the development of codified rules (Hofinger & Schmidinger, 2017; Disley et al., 2016; Inspector of Custodial Services NSW, 2018; Sizoo et al., 2022; Piltch‐Loeb et al., 2021; Vandaele et al., 2022a; Kotzur et al., 2022). However, information sharing still rested on the willingness and trust of partners (Kotzur et al., 2022; Sizoo et al., 2022), as even when information sharing rules are codified, they may only provide basic guidance as to what can be shared (Inspector of Custodial Services NSW, 2018). Partners can also be unaware what the formal rules on information sharing are (Vandaele et al., 2022a), and there may be laws that inhibit information sharing (Kotzur et al., 2022). Secure data transfer systems between agencies may be able to address some of these barriers (Hofinger & Schmidinger, 2017).

Seven studies identified potential conflicts that can emerge when different partners are rooted in differing organisational contexts and cultures and/or lack an understanding of partners' organisational culture(s) (Weggemans & de Graaf, 2017; Hellevik et al., 2022; Raets, 2022; Haugstvedt & Gunnarsdottir, 2023; Sizoo et al., 2022; Kotzur et al., 2022; Stern et al., 2023). A common issue was the discomfort that practitioners working in non‐security sectors might experience when engaging with security actors, which may require them to share information about individuals to whom they have a duty of care (Haugstvedt & Gunnarsdottir, 2023; Raets, 2022). This issue was discussed in the context of mental healthcare, with practitioners from other sectors noting how engaging with mental health practitioners can be challenging due to a lack of training, or willingness to work with violent extremists (Hellevik et al., 2022; Sizoo et al., 2022; Stern et al., 2023). However, this issue was not universal, and in some cases, multi‐agency working with mental health professionals was unproblematic (Sizoo et al., 2022).

Having a large number of people involved in multi‐agency meetings may potentially inhibit attendees from sharing pertinent information, whilst reducing the number of attendees at meetings may facilitate information sharing (Disley et al., 2016). Multi‐agency structures with fewer actors may also be better at taking responsibility for cases, thereby reducing the chances of cases being passed onto other actors in ways that may be unhelpful (Vandaele et al., 2022a).

######## The clarity of intervention goals

Twelve studies highlighted the importance of intervention goals being clear and understood to all partners. Ten of these studies identified a lack of clarity around goals as a collaboration challenge (Marsden, 2015; Schuurman & Bakker, 2016; Weggemans & de Graaf, 2017; Weeks, 2018; Orban, 2019; Harris‐Hogan, 2020; Pettinger, 2020a; Førde & Andersen, 2021; Kotzur et al., 2022; Thompson & Leroux, 2022; Vandaele et al., 2022a). The goals of multi‐agency partners may diverge from each other, and/or from policymakers (Marsden, 2015; Schuurman & Bakker, 2016; Weeks, 2018; Orban, 2019; Pettinger, 2020a; Harris‐Hogan, 2020; Førde & Andersen, 2021; Kotzur et al., 2022). These differences may be reflected in contrasting ways of assessing success between the government and intervention providers (Weeks, 2018) and between national and local practitioners (Harris‐Hogan, 2020).

Barriers can be shaped by a lack of clarity over the intended outcomes of multi‐agency working (Weggemans & de Graaf, 2017; Orban, 2019; Thompson & Leroux, 2022; Vandaele et al., 2022a). For example, prison staff in Norway were not aware of the purpose of the mentorship programme offered to inmates, and did not appreciate that mentors had conversations with offenders in confidence (Orban, 2019). Differing goals may also reflect differences in organisational culture (Weggemans & de Graaf, 2017). When practitioners, such as the police, are more focused on public protection, and probation on the resettlement and rehabilitation of offenders, this has the potential to create barriers (Marsden, 2015).

Where common goals are understood, this can facilitate multi‐agency working (Sträter & Stuppert, 2019). Regularly reviewing and clarifying outcomes and goals through the development of logic models (Thompson & Leroux, 2022), or through other documentation accessible to all stakeholders can support interventions (Vandaele et al., 2022a; Kotzur et al., 2022). Interventions that lack clearly articulated goals may not include all relevant partners (Thompson & Leroux, 2022), or may end up with multi‐agency structures that are ‘too bulky and complex’ due to the inclusion of agencies who are less important to the case management process (Weggemans & de Graaf, 2017, p. 117).

######## Relationships between different partners

Nine studies highlighted how relationships between specific multi‐agency partners can be challenging (AEF, 2018; van der Heide & Schuurman, 2018; Schuhmacher, 2018; van de Weert & Eijkman, 2020; Stern et al., 2023; Thompson & Leroux, 2022; Solhjell et al., 2022; Hellevik et al., 2022; Sizoo et al., 2022). This included three studies which highlighted how working with specific agencies, actors and/or municipalities can be challenging, even when overall broader multi‐agency or multi‐disciplinary collaborations are found to be working well (AEF, 2018; van der Heide & Schuurman, 2018; Stern et al., 2023).^22^

Relationships between the police and other actors can sometimes be characterised by power differentials and hierarchical struggles which can create barriers to implementation (Thompson & Leroux, 2022; Solhjell et al., 2022; van de Weert & Eijkman, 2020). Related challenges include a lack of clarity over the jurisdictional boundaries of different partners, and where their specific mandate begins and ends (AEF, 2018; Schuhmacher, 2018; Hellevik et al., 2022), and disagreements between partners about their own responsibilities (Sizoo et al., 2022).

Six studies discussed factors that facilitated positive relationships between partners (Disley et al., 2016; Sträter & Stuppert, 2019; Vandaele et al., 2022a, 2022b; Kotzur et al., 2022; Thompson & Leroux, 2022; Solhjell et al., 2022; Sizoo et al., 2022). Approaches to facilitating relationship building included training practitioners about the mandates and working practices of the other organisations involved in multi‐agency working structures and focusing attention on nurturing effective collaborations, a process supported by the commitment, and personalities of those involved (Sträter & Stuppert, 2019; Thompson & Leroux, 2022).

Trust between individual practitioners and between agencies was the most commonly cited facilitator of effective multi‐agency working (Vandaele et al., 2022a, 2022b; Kotzur et al., 2022; Disley et al., 2016; Thompson & Leroux, 2022; Solhjell et al., 2022; Sizoo et al., 2022). Three forms of trust have been described as important: structural, reflecting a general trust in the authorities, and the multi‐agency process; professional, reflecting trust in specific professions, and/or their representatives within the multi‐agency structure; and personal, reflecting trust in individuals (Solhjell et al., 2022, pp. 173‐175). Whilst it can be a time‐consuming process, trust building is therefore likely to be important (Sträter & Stuppert, 2019).

######## Ensuring the right partners are involved in multi‐agency working structures

Eight studies highlighted the importance of ensuring that the right partners were involved in multi‐agency partnerships. This included ensuring that all partners relevant to achieving stated goals or performing specific case management functions such as client assessment are included (van de Weert & Eijkman, 2020; Cherney, 2020; Mattsson, 2021; Thompson & Leroux, 2022; Piltch‐Loeb et al., 2021; Vandaele et al., 2022b), and ensuring that all partners add value, contribute, and have relevant expertise (Weggemans & de Graaf, 2017; Solhjell et al., 2022; Vandaele et al., 2022b). One study which asked probation officers to assess the utility of working with different agencies found that collaboration with mental health professionals was most useful, followed by client's family members, and law enforcement (Stern et al., 2023).

####### Risk‐oriented logics

The use of risk‐oriented logics was examined in research relating to both secondary and tertiary prevention. This discussion also touched on the potential issue of false or disguised compliance.

######## Risk‐oriented approaches to tertiary prevention

Fifteen studies examining tertiary interventions highlighted the use of risk‐oriented approaches. A focus on risk was highlighted by explicit reference to the RNR model (Marsden, 2015; Cherney, 2018, 2021), or by the language practitioners used to describe their work (Schuurman & Bakker, 2016; Eijkman & Roodnat, 2017, ^23^; Weggemans & de Graaf, 2017; Webster et al., 2017; AEF, 2018; van der Heide & Schuurman, 2018; Inspector of Custodial Services NSW, 2018; Disley et al., 2016; Weeks, 2018; Raets, 2022, ^24^; Corner & Pyszora, 2022; Stern et al., 2023). This focus on risk reduction aligns with the central logic of tertiary CVE interventions, in that they are typically considered ‘terrorism risk reduction initiatives’ (Williams & Kleinman, 2014) in the academic literature. However, five of these studies discussed how a preoccupation with risk might create implementation challenges (Marsden, 2015; Schuurman & Bakker, 2016; Disley et al., 2016; Weeks, 2018; Cherney, 2021). Whilst results from five studies cannot be considered to represent a strong evidence base – particularly as these studies were assessed as having a moderate risk of bias – the consistency of their findings suggests that these specific challenges are emerging in a variety of different contexts.

Three studies identified challenges in trying to integrate the goals of risk reduction and rehabilitation, highlighting how a focus on the former might undermine the latter (Marsden, 2015; Weeks, 2018; Cherney, 2021). In noting how the **London Probation Trust** primarily focused on risk reduction when working with post‐release terrorist offenders, Marsden (2015) points to a tension between the rehabilitative goals expressed by practitioners, and the risk‐oriented logics that underpinned the strict post‐release license conditions offenders were typically subject to. Whilst this study concluded that an integration of risk‐oriented and rehabilitative logics was ‘tenable’, it also recognised that this can be challenging in practice.

Similarly, an analysis of the **UK mentoring system** highlighted the challenge of ‘conceptualising where the balance point is’ between public protection and rehabilitative goals (Weeks, 2018, p. 537). This study also pointed to a ‘potential disparity’ (p. 535) between the short‐term goal of protecting the public, and the long‐term goal of facilitating desistance by highlighting how terrorist offenders themselves might view the restrictions placed on them as factors which created barriers to their rehabilitation and reintegration into society. A similar tension is identified by Cherney (2021) and is discussed further in the section on public and political pressure below.

A related challenge identified by Disley et al. (2016) was that of balancing ‘overt’ and ‘covert’ risk management (p. 25) when offenders are placed under more covert forms of surveillance whilst being case managed through **MAPPA**. Two studies highlighted how a broader political focus on reducing risk might come into tension with rehabilitative goals (Cherney, 2021; Schuurman & Bakker, 2016), a point discussed in more detail in the section on public and political pressure.^25^

######## Risk‐oriented approaches to secondary prevention

Risk management was often an important function of secondary interventions. Four studies highlighted how risk‐oriented logics impacted how practitioners worked with clients assessed as being at risk of radicalisation (Eijkman & Roodnat, 2017; Haugstvedt, 2019; Haugstvedt & Gunnarsdottir, 2023; Haugstvedt & Tuastad, 2021; Mattsson, 2021; Raets, 2022). Whilst the evidence‐base relating to these challenges remains limited, research on secondary interventions identified similar challenges to those discussed above in the context of tertiary interventions.

These studies highlighted how risk‐oriented logics can create barriers by contributing to risk aversion, which may conflict with rehabilitative goals. This can be informed by the security context which can entangle professionals from non‐security fields in a ‘security logic’ where risk aversion has the potential to overcome commitments to confidentiality or client privacy (Haugstvedt & Tuastad, 2021, p. 8; Haugstvedt & Gunnarsdottir, 2023). The dominance of risk‐oriented logics means that professionals have to ‘navigate [a] dual agenda of control and care at the micro‐level’ (Raets, 2022, p. 243), which can create a tension between security logics and the pursuit of client‐oriented goals (Haugstvedt & Gunnarsdottir, 2023).

These issues are not considered insurmountable, and goals related to care and control may complement one another over the long‐term (Raets, 2022). Approaches which may help to mitigate these issues and to resist risk‐oriented logics in ways that facilitate case management processes include using client‐oriented approaches which are more focused on helping the client achieve their own goals (Haugstvedt, 2019, p. 167; Haugstvedt & Tuastad, 2021), or using strengths‐based and desistance‐informed approaches, such as the Good Lives Model (Raets, 2022, pp. 246‐247). Practitioners may also be able to resist the language of risk by recognising the potentially long‐term impact of labelling someone as ‘high risk’, such as attracting future sanctions or media attention (Eijkman & Roodnat, 2017).^26^

######## False compliance

One study examined the topic of false compliance in detail (Cherney et al., 2022). Six further studies made passing reference to this issue but in insufficient depth to be included in the analysis of this theme (Weggemans & de Graaf, 2017; van der Heide & Schuurman, 2018; Haugstvedt, 2019; Cherney & Belton, 2021a; Raets, 2022; Stern et al., 2023). False compliance was not considered a significant issue by practitioners interviewed for this study, although none ruled out the potential for it to occur (Cherney et al., 2022). An overly suspicious, risk‐averse approach to clients was therefore not considered effective in identifying disguised compliance, although practitioners also cautioned against being too optimistic when assessing any observed client change (Cherney et al., 2022). However, the results of one small‐scale study cannot be considered representative, and more research examining the topic of false or disguised compliance would be useful at this time.

####### Public and political pressure

Ten studies highlighted how the public attention that interventions working to counter radicalisation to violence tend to attract might influence implementation (Schuurman & Bakker, 2016; Weggemans & de Graaf, 2017; Hofinger & Schmidinger, 2017; Disley et al., 2016; AEF, 2018; Schuhmacher, 2018; Cherney, 2021; Raets, 2022; Cherney et al., 2022; Stern et al., 2023). These studies identified barriers to implementation linked to such attention. The evidence base underpinning these barriers can be considered fairly strong given that the studies discussing this issue had a low risk of bias, as evidenced by over half (*n* = 6) scoring positively on eight (*n* = 2) or nine (*n* = 4) of the ten domains in the CASP tool.

Practitioners placed under public scrutiny might perceive they are under pressure to adopt a more risk averse attitude (Disley et al., 2016; Cherney, 2021; Cherney et al., 2022; Raets, 2022). Public debates around the risk of disguised compliance were described as potentially ‘disempowering’ for practitioners (Cherney et al., 2022, p. 40). The high‐profile nature of countering radicalisation to violence programmes might also negatively impact practitioners' confidence to deliver (Raets, 2022), or willingness to engage in (Stern et al., 2023), work of this nature.

Risk aversion shaped by public scrutiny can lead to interventions being delivered in ways that diverge from their underlying logic. Although the rationale of a risk‐oriented approach is that a reduction in risk should lead to lower levels of supervision, practitioners may not follow this principle for fear of being held responsible for a further offence, even in the face of evidence that this risk has reduced (Cherney, 2021, p. 133). A political focus on reducing risk and limiting opportunities for parole may also undermine the positive, rehabilitative goals enabled through community supervision (Cherney, 2021), and may reduce policymakers' willingness to support reintegration work with high‐profile offenders (Schuurman & Bakker, 2016).

Political and media scrutiny may make it harder for practitioners to build relationships with clients who are wary of attempts to engage them (Disley et al., 2016; Cherney, 2021). Ongoing stigmatisation linked to past offending can undermine reintegration and rehabilitation (Weggemans & de Graaf, 2017), and high levels of scrutiny may set parolees ‘up for failure’ (Cherney, 2021, p. 133). Under these circumstances, offenders might feel that ‘decisions about their classification, release, and supervision conditions are determined by “politics”’ (Cherney, 2021, p. 133; Weggemans & de Graaf, 2017) as opposed to their own behaviour. This might contribute to a sense of injustice (Hofinger & Schmidinger, 2017; Weggemans & de Graaf, 2017), and inhibit trust building (Hofinger & Schmidinger, 2017).

Public scrutiny is not inevitable, however. Attention directed at the **TER intervention** ebbed and flowed, and overall, it received less public scrutiny than might have been expected for a programme of its kind (Schuurman & Bakker, 2016). Whilst attention peaked around the release of two better known prisoners, it receded quickly, leading the researchers to suggest that this low level of attention was likely down to the fact that the programme had not been made public, suggesting that work with those who are not well known is of less interest.^27^

####### Resourcing

Seventeen studies highlighted how a shortage of time, staff, or other resources can create implementation challenges, and in turn highlighted the importance of providing adequate resources to support interventions (Becker et al., 2014; Schuurman & Bakker, 2016; Webster et al., 2017; Hofinger & Schmidinger, 2017; Weggemans & de Graaf, 2017; Möller & Neuscheler, 2018; van der Heide & Schuurman, 2018; AEF, 2018; Sträter & Stuppert, 2019; Orban, 2019; Harris‐Hogan, 2020; Mattsson, 2021; Haugstvedt & Gunnarsdottir, 2023; Piltch‐Loeb et al., 2021; Raets, 2022; Vandaele et al., 2022a, 2022b; Kotzur et al., 2022; Stern et al., 2023). Two further studies emphasised the importance of providing practitioners with formal or informal support and guidance (Haugstvedt, 2022) and with adequate resources (Schuhmacher, 2018) to cope with the specific pressures of working in this area. The importance of resourcing is supported by a robust evidence base, with a low risk of bias, with three quarters (*n* = 12) of the 16 studies assessed using the CASP tool scoring positively on at least eight of the ten domains, and eight scoring positively on nine (*n* = 7) or all ten (*n* = 1).

Barriers relating to resource issues may be more pronounced for newer interventions (Vandaele et al., 2022a; Sträter & Stuppert, 2019); in countries with less well‐developed CVE infrastructures (Piltch‐Loeb et al., 2021; Vandaele et al., 2022b); or where practitioners' roles in CVE are less institutionalised (Kotzur et al., 2022; Stern et al., 2023). The need to develop and implement interventions in a condensed timeframe can also lead to practitioners delivering work without clear guidance, or a robust understanding of intervention goals (Orban, 2019; Harris‐Hogan, 2020; Raets, 2022). Financial pressures can cause challenges, in particular where funding is needed for specialist external partners to support case work (van der Heide & Schuurman, 2018) or where there is a reduction in funding (Becker et al., 2014; Orban, 2019).

For example, Becker et al. (2014) reported that in 6% of cases, individual counselling delivered through the **XENOS** programme in Germany had to be terminated once funding for the programme had ended (p. 99), concluding that the absence of ongoing funding after the initial funding period had ended ‘was to the disadvantage of the participants’ (p. 132).

A number of studies suggested barriers might be generated by time‐limited funding arrangements that undermined programme sustainability (Möller & Neuscheler, 2018; AEF, 2018), or highlighted short‐term project funding as a potential challenge for practitioners (Kotzur et al., 2022). The nature of employment terms can also create challenges. The practice of offering ad hoc contracts can create practical challenges for practitioners who do not receive a fixed salary, lack clarity on their expected caseloads, are only offered limited travel expenses, and do not receive sufficient compensation given the demands of the work (Orban, 2019; Mattsson, 2021). This can mean that effective practitioners are underutilised (Mattsson, 2021).

The intensity of CVE work, and the perception that extremist clients require more supervision and support than other offending cohorts may generate barriers to implementation. It can contribute to resourcing issues (AEF, 2018; Schuurman & Bakker, 2016; van der Heide & Schuurman, 2018; Stern et al., 2023), or place strain on practitioners (Hofinger & Schmidinger, 2017; Haugstvedt & Gunnarsdottir, 2023; van der Heide & Schuurman, 2018; Sträter & Stuppert, 2019), particularly when they are required to take part in this work in addition to their normal responsibilities (Kotzur et al., 2022). Tension can be caused when more senior stakeholders do not see reintegration work with extremist clients as a high priority, which may lead them to challenge the amount of time that practitioners spend on this work in comparison to other clients (Schuurman & Bakker, 2016). The potential for overwork, and for ‘stress and dissatisfaction’ was also noted (van der Heide & Schuurman, 2018, p. 214). Overcoming these issues was identified as important in facilitating implementation, as it supports the client‐centred, intensive approach that informs programmes (van der Heide & Schuurman, 2018).

Practical considerations such as the length of waiting times are also important implementation factors (AEF, 2018; Orban, 2019). For example, whilst the AEF (2018) identified the short waiting time as a strength of **Forsa** in the Netherlands, Orban (2019) noted how long waiting times and other resource limitations can potentially create frustration for clients of other interventions.

####### Staff expertise

Twenty‐three studies emphasised the importance of staff expertise in facilitating delivery. These studies were assessed as having a low risk of bias using the CASP tool, with two thirds (*n* = 16) scoring positively on eight (*n* = 4), nine (*n* = 11), or all ten (*n* = 1) CASP domains. The expertise held in multidisciplinary teams, with diverse and ideally complementary knowledge and skills was considered an important facilitator of case management (Becker et al., 2014; AEF, 2018; Schroer‐Hippel, 2019; Haugstvedt, 2022; Costa et al., 2021). Whilst expertise and experience related to other types of offenders may be transferable to work with at risk or radicalised individuals, specialist expertise and/or (gaining) experience of working with these cohorts is valuable (Spalek et al., 2010; Christensen, 2015; Schuurman & Bakker, 2016; Weggemans & de Graaf, 2017; Eijkman & Roodnat, 2017; Webster et al., 2017; Disley et al., 2016; van der Heide & Schuurman, 2018; Weeks, 2018; AEF, 2018; Orban, 2019; Vandaele et al., 2022b). Other studies emphasised the importance of skills not specific to CVE that would support this work, such as broader counselling skills (Cherney et al., 2022).

Barriers to ensuring the appropriate staffing of case management interventions include difficulties recruiting and retaining staff with the requisite, specialist expertise (AEF, 2018; Vandaele et al., 2022a), and trying to engage external partners with specific expertise (Stern et al., 2023; Hellevik et al., 2022; Sizoo et al., 2022). Conversely, maintaining continuity within intervention teams, and in the membership of multi‐agency working structures, can be facilitators (Becker et al., 2014; Vandaele et al., 2022a).

Training was identified as an important facilitator by sixteen studies (Christensen, 2015; Schuurman & Bakker, 2016; Eijkman & Roodnat, 2017; Webster et al., 2017; Weggemans & de Graaf, 2017; Inspector of Custodial Services NSW, 2018; van der Heide & Schuurman, 2018; Disley et al., 2016; Haugstvedt, 2022; Pettinger, 2020a; Cherney, 2021; Piltch‐Loeb et al., 2021; Thompson & Leroux, 2022; Vandaele et al., 2022a; Sizoo et al., 2022; Stern et al., 2023), although some authors highlighted that there were likely limits to what specialised training could achieve (Weggemans & de Graaf, 2017; Pettinger, 2020a). This included studies describing the benefits of providing training on topics that, although not specific to countering radicalisation to violence, would support practitioners in delivering this work. This included training on techniques such as motivational interviewing (Christensen, 2015; Haugstvedt, 2022), and on multi‐agency collaboration (Sizoo et al., 2022; Thompson & Leroux, 2022). Studies emphasising the importance of training were again assessed as having low risk of bias.

The absence of specific expertise within intervention teams – most commonly related to ideological work – can be a potential barrier to implementation (Schuurman & Bakker, 2016; Weggemans & de Graaf, 2017; van der Heide & Schuurman, 2018; Orban, 2019; Thompson & Leroux, 2022; Vandaele et al., 2022a; Stern et al., 2023). However, interventions and practitioners may be able to draw on external partners when they lack the specific in‐house expertise needed to deliver specific forms of support as part of intervention plans (AEF, 2018; Schuurman & Bakker, 2016; van der Heide & Schuurman, 2018; Sträter & Stuppert, 2019; Fisher et al., 2020); when requiring advice on specific cases (Haugstvedt, 2022); and for training on specific topics relevant to countering radicalisation (Vandaele et al., 2022a, 2022b).

It can take time for staff to build relationships with external partners able to provide this additional expertise (van der Heide & Schuurman, 2018; Sträter & Stuppert, 2019). Barriers to building up a network of relevant actors include relatively small caseloads and the time it takes to build up a relationship of trust with external stakeholders (Sträter & Stuppert, 2019). Overcoming these challenges is possible, for example, **Team TER** staff who were initially sceptical of external theological consultants came to see them as ‘central to the initiative's overall efforts’ (van der Heide & Schuurman, 2018, p. 212).

Three studies discussed the importance of language skills. The ability to converse with clients in their mother tongue helped facilitate mentoring delivered in custodial settings in Norway (Orban, 2019), whilst a lack of relevant language skills can be a potential barrier to implementation (Hofinger & Schmidinger, 2017; Stern et al., 2023). This is both because it hampers the process of working with clients (Hofinger & Schmidinger, 2017) and because it can mean practitioners don't have full sight of a client's activities. For example, a probation officer working in the United States expressed ‘concern about not being able to tell if a client's foreign language writing contained extremist content’ (Stern et al., 2023, p. 13).

####### Voluntary and mandatory interventions

Eleven studies presented evidence relating to mandating interventions or making them voluntary (Becker et al., 2014; Christensen, 2015; Weggemans & de Graaf, 2017; Costa et al., 2021; AEF, 2018; Orban, 2019; Schroer‐Hippel, 2019; Cherney, 2018, 2020; van der Heide & Schuurman, 2018; Cherney et al., 2022). These studies were assessed as having a low risk of bias, with the majority (*n* = 7) scoring positively on nine (*n* = 5) or all ten (*n* = 2) of the CASP domains. Whilst evidence specifically related to the impact of mandating (or not mandating) interventions was limited within these studies, a number of important points were raised.

This research generally suggested that practitioners prefer voluntary approaches on the basis that clients must be motivated to change if an intervention is to be effective (Christensen, 2015; AEF, 2018; Orban, 2019; Costa et al., 2021). One evaluation reported that it was beneficial if clients entered an intervention already willing to disengage from violent extremism (Schroer‐Hippel, 2019), whilst another suggested it was not uncommon for clients to have begun disengaging before agreeing to participate in an intervention (Cherney, 2018). In contrast, there was some concern that mandated clients may not be motivated to disengage (Becker et al., 2014). Disguised compliance may also be less of an issue with voluntary programmes, the risk of which may increase where participation is mandated (Cherney et al., 2022).

However, some research suggested that the voluntary nature of some programmes might undermine attempts to engage potential intervention clients. Practitioners in the Netherlands reported concerns about the lack of mandatory programmes, explaining that they ‘feel powerless without some way to force former detainees to participate in these programs’ (Weggemans & de Graaf, 2017, p. 109). In turn, there was some suggestion that voluntary interventions might face challenges encouraging individuals to engage with programmes when they are not mandated to (Weggemans & de Graaf, 2017; Schroer‐Hippel, 2019).^28^ However, practitioners delivering both voluntary (Cherney, 2018, 2020) and mandatory (van der Heide & Schuurman, 2018) interventions highlighted how motivating clients can be challenging, and in some cases, almost impossible (van der Heide & Schuurman, 2018), suggesting that mandating an intervention will not always be sufficient to overcome this challenge.^29^

####### Broader legislation

Eight studies considered the impact of broader counter‐terrorism legislation and other related criminal justice measures on how interventions are implemented, with implications for their potential effectiveness (Schuurman & Bakker, 2016; Weggemans & de Graaf, 2017; Eijkman & Roodnat, 2017; van der Heide & Schuurman, 2018; Disley et al., 2016; Cherney, 2021; Cherney et al., 2022; Raets, 2022). Whilst this represents a small number of studies, these studies were assessed as being high quality, and of only having a low to moderate risk of bias given that over half (*n* = 5) scored positively on at least eight of the CASP domains.

The enactment of broader counter‐terrorism powers or other criminal justice measures can create barriers when engaging with, and seeking to rehabilitate, clients through case management interventions (Schuurman & Bakker, 2016; Weggemans & de Graaf, 2017; Eijkman & Roodnat, 2017; Disley et al., 2016; Raets, 2022). Individuals who are simultaneously subject to harder forms of counter‐terrorism intervention, such as being stripped of citizenship rights or being unable to open a bank account, experience barriers to their rehabilitation (Schuurman & Bakker, 2016; Weggemans & de Graaf, 2017; Raets, 2022) which can make it ‘very difficult to create a perspective for the future’ that they are motivated to work towards (Raets, 2022, p. 245). Weggemans and de Graaf (2017) reported that Dutch laws focused on the suppression of terrorism ‘are considered by several former prisoners to be a major, if not the biggest obstacle to successful reintegration’ for this reason (p. 98).

The imposition, but also the lifting of other sanctions, particularly without the opportunity to prepare clients, can create a barrier to rehabilitation efforts (Weggemans & de Graaf, 2017), whilst delivering interventions when clients are subject to other criminal justice interventions may be sub‐optimal (Eijkman & Roodnat, 2017). However, sanctions may, in some circumstances, facilitate case management processes, particularly when they are enacted in ways that do not undermine interventions, and when used selectively and as part of a broader, well‐coordinated, multi‐agency approach (Weggemans & de Graaf, 2017, p. 115).

An issue specific to the Dutch context was the potential overlap between programmes run by the National Support Centre for Extremism (LSE) – including **Forsa** – and **Team TER** (van der Heide & Schuurman, 2018). By 2018, LSE had ‘become a competitor of sorts to team TER’, due partly to an unclear mandate determining which should be the lead organisation. Because the LSE interventions operated ‘outside of a criminal justice framework’, they had strict privacy controls, which created barriers to cooperating and sharing information with other organisations (van der Heide & Schuurman, 2018).

###### Moderators (Objective 2b)

Eligible studies referred to four moderators: delivery context; local context; whether an intervention was standalone; and client challenges that affected their ability to engage in interventions.

####### Delivery context

Eleven studies highlighted how the characteristics of delivery contexts might facilitate and/or inhibit implementation (Christensen, 2015; Webster et al., 2017; Hofinger & Schmidinger, 2017; Weggemans & de Graaf, 2017; Inspector of Custodial Services NSW, 2018; Khalil et al., 2019; Orban, 2019; Cherney, 2020, 2021; Raets, 2022; Jukschat et al., 2020). The findings of these studies can be considered to be particularly robust, with the vast majority (*n* = 8) scoring positively on nine (*n* = 6) or all ten (*n* = 2) of the domains within the CASP tool.

Interventions delivered in correctional contexts were seen to face specific barriers. Strict controls placed on offenders in prison can make it difficult for intervention providers to build therapeutic and trusting relationships with clients (Hofinger & Schmidinger, 2017; Orban, 2019; Cherney, 2020, 2021; Inspector of Custodial Services NSW, 2018; Jukschat et al., 2020), and may restrict the time that practitioners have to perform case management functions (Webster et al., 2017). Such restrictions may also inhibit inmates from participating in activities that might contribute to their rehabilitation (Inspector of Custodial Services NSW, 2018).

Prison conditions can negatively impact mental health (Raets, 2022; Weggemans & de Graaf, 2017), and contribute to feelings of discrimination (Weggemans & de Graaf, 2017). Limiting the use of harsher detention regimes and providing expedited access to support services were two of the ways practitioners in Belgium sought to mitigate these challenges (Raets, 2022). These issues may be less acute for individuals on probation who may have more ‘hope for the future compared with prisoners facing long sentences, or appealing their sentencing’ (Webster et al., 2017, p. 25). However, the post‐release context can also create specific challenges: greater freedom, and the presence of more external, uncontrollable factors than existed in the custodial environment may make the monitoring and assessment of offenders on probation or parole challenging (Cherney, 2021; Weggemans & de Graaf, 2017).

Institutional contexts seen to facilitate intervention goals are characterised by a clear communication of the ethos and values of the organisation (Christensen, 2015, p. 98), and good quality conditions that able to support positive change, and which have the potential to encourage clients to reassess negative attitudes towards the government (Khalil et al., 2019).

Operating in a conflict‐affected setting can create specific barriers. For example, the **Serendi Rehabilitation Centre** in Somalia faced the active presence of violent extremist groups (Khalil et al., 2019). This was seen to potentially undermine efforts to promote long‐term disengagement, and posed a security risk for staff and clients (Khalil et al., 2019). Although practitioners and former extremists engaging in interventions in non‐conflict settings have also expressed concerns about hostility from extremists (Weggemans & de Graaf, 2017).

####### Local context

Ten studies highlighted how the delivery of case management was shaped to and by features of specific local contexts (Harris‐Hogan, 2020; Pettinger, 2020a, 2020b; Eijkman & Roodnat, 2017; Weggemans & de Graaf, 2017; van der Heide & Schuurman, 2018; Schroer‐Hippel, 2019; Raets, 2022; Vandaele et al., 2022a; Solhjell et al., 2022; Stern et al., 2023; Kotzur et al., 2022). These studies were assessed as having a low risk of bias, with three‐quarters (*n* = 8) of these studies scoring positively on eight (*n* = 3) or nine (*n* = 5) of the critical domains in the CASP tool. Local context can therefore be considered an important moderator of implementation.

Typically, tailoring was a function of programme design, with a number of interventions providing space for practitioners to tailor interventions to the regions in which they worked (Harris‐Hogan, 2020; Pettinger, 2020a, 2020b; Eijkman & Roodnat, 2017; Raets, 2022; Vandaele et al., 2022a; Solhjell et al., 2022; Stern et al., 2023), or the features of the CVE infrastructure in specific countries (Kotzur et al., 2022). Although some level of national consistency can be valuable, providing space for tailoring can facilitate interventions by enabling practitioners to adapt programmes to an area's particular needs (Harris‐Hogan, 2020).

Tailoring interventions to local contexts also allows practitioners and policymakers to take account of varying local levels of resources, expertise and risk. For example, the Netherlands and the UK provide additional resources for ‘priority areas’, which are regions deemed to have a greater level of local radicalisation risk (Pettinger, 2020a, 2020b; Eijkman & Roodnat, 2017).^30^ Taking account of different levels of resources and experience can help to facilitate delivery, whilst expertise can cluster around larger or better resourced locations (Weggemans & de Graaf, 2017). Priority municipalities in the Netherlands with more experience of delivering individualised interventions and greater capacity attracted smaller, non‐priority municipalities who sought to develop cooperative relationships with them (Eijkman & Roodnat, 2017). However, interventions operating in smaller locales may not be transferable to larger regions. In noting how the **KOMPASS intervention** in Germany was largely based on outreach work, Schroer‐Hippel (2019) argued this programme had limited transferability to regions with ‘long distances between the counselling centre and the counselling seekers’ (p. 14).

The features of the local context, such as employment opportunities and the services available in neighbourhoods, were also found to influence how multi‐agency working structures operated in different regions in the Netherlands, Germany, and Belgium (Vandaele et al., 2022a). The presence of an effective local co‐ordinator (Eijkman & Roodnat, 2017) or local practitioner(s) (Weggemans & de Graaf, 2017) were also identified as key facilitators of interventions.

Two studies highlight how regional variations might create challenges. Variation in the quality of the relationships between different regional authorities in the Netherlands saw **Team TER's** engagement with Amsterdam identified as a particular challenge (van der Heide & Schuurman, 2018). This study also found that the quality of cooperation with the Public Prosecution Service varied across different regions. In the USA, ‘conditions imposed on [extremist] offenders are written by the district in which the crime takes place but are enforced where the person lives’ (Stern et al., 2023, p. 12), which means that cooperation and coordination between different regional authorities is important. However, this coordination may not always be effective, creating a barrier to effective case management working.

####### Standalone interventions

The differing role of standalone and non‐standalone interventions was discussed in four studies (Raets, 2022; Thompson & Leroux, 2022; Becker et al., 2014; Stern et al., 2023). Only one of these studies discussed how this moderator impacted implementation, highlighting the benefits of interventions being delivered by organisations who are already well‐established in the community before becoming engaged in CVE work (Thompson & Leroux, 2022). The strength of evidence underpinning this moderator is therefore limited, suggesting that more research into this topic is needed. However, several useful, preliminary insights can be drawn from this research.

Pre‐existing connections can enable organisations to avoid the challenges faced by newly introduced, standalone CVE interventions which may face resistance due in part to ‘pre‐existing scepticism and mistrust of the police and CVE more generally’ (Thompson & Leroux, 2022, p. 10). In the absence of pre‐existing connections, standalone interventions can overcome these barriers by becoming known in the local area, nurturing a good reputation, and employing a positive public relations approach (Becker et al., 2014).

More generally, a lack of available CVE‐specific support can act as a barrier. Almost 80% of a sample of American probation officers ‘indicated a lack of specific programming for extremist offenders’ (p. 23) and discussed how offenders might be supported through more generic rehabilitation services. The study explained that ‘the lack of extremist‐specific programming was a consistent complaint’ amongst their sample (Stern et al., 2023, p. 23).

####### Client challenges

Four studies highlighted how ongoing challenges in clients' lives can create barriers to their ability or willingness to engage in case management interventions (Lukas, 2006; Möller et al., 2015; Weggemans & de Graaf, 2017; Cherney, 2022). Whilst the evidence underpinning this moderator is underdeveloped, there was some consistency in the types of challenges identified across this small number of studies. These challenges include mental health and other psychological issues (Weggemans & de Graaf, 2017; Cherney, 2022); addiction and substance abuse (Lukas, 2006; Möller et al., 2015); and a breakdown or absence of supportive relationships (Lukas, 2006; Cherney, 2022). In turn, these studies highlight how a client's engagement with interventions, and their progress towards intervention goals, may fluctuate over time (Cherney, 2022). In some instances, interventions may need to be paused, or even cancelled, so that other issues can be dealt with (Lukas, 2006; Möller et al., 2015). One additional study also highlighted how support may end for more practical reasons, such as a client's relocation to a region not supported by an intervention (Möller & Neuscheler, 2018).

Four studies tracking client progress highlighted that fluctuations in engagement and progress, and even setbacks, did not prohibit interventions from producing positive outcomes over time (Lukas, 2006; Cherney, 2022; Cherney & Belton, 2021a, 2021b) – although, the strength of these conclusions is limited due to the fact that three of these studies were conducted by the same author, and two focused on the same intervention in Australia. Practitioners interviewed by Cherney et al. (2022) similarly emphasised the importance of having ‘an acceptance of, and level of comfort with, the idea that that rehabilitation of an extremist often includes setbacks and reversions to previous behaviours or thought processes’ (p. 32).

### 4.3 Discussion

#### 4.3.1 Summary of main results

Part I of the review had two objectives: to understand the effectiveness of tools and approaches used in case management interventions seeking to counter radicalisation to violence, and to examine those factors and moderators which impact their implementation. One of the main findings from Part I is that very little is known about the effectiveness of tools and approaches used in this context (Objective 1). Whilst a number of innovative methodologies for assessing the effectiveness of case management were identified (e.g., Cherney & Belton, 2021a, 2021b; Cherney, 2022), no quasi‐experimental or experimental evaluations of case management tools and/or approaches were identified. As a result, we are unable to offer any conclusions relating to Objective 1 on effectiveness.

However, we identified a relatively large, and growing, body of research (*n* = 47 studies) relating to Objective 2 on implementation. The key findings relating to the facilitators, barriers, and moderators impacting the implementation of tools and approaches are summarised below.

Another important finding is the utility of the case management model in supporting the analysis of secondary and tertiary interventions. Although the language of case management has only recently begun to be applied to counter‐radicalisation research and practice, it offers a valuable way of organising the array of tools and approaches that are used in this context.

*Implementing case management approaches*

We had hoped to understand whether particular approaches, such as the RNR model or strengths‐based approaches, were more or less effective at informing case management interventions. However, it was not possible to develop a typology of different approaches based on the information provided in the eligible studies, and in turn to assess the implementation of approaches in the way conceptualised in the background section above, or the original protocol.

However, it was possible to assess the specific theories of change or programme logics (i.e., the approach) underpinning individual interventions. Seven studies assessed the assumptions underpinning an intervention and/or examined whether a programme was being delivered in line with a clearly defined programme logic, theory of change, or approach (Objective 2a). In general, these studies reported positive, albeit somewhat mixed results. Four studies that used academic research as a benchmark concluded that the assumptions underpinning interventions were sound, but caveated this observation by noting that the academic literature was limited. Four studies that assessed whether interventions were being implemented in ways which aligned with their own underlying logic reported that programmes generally adhered to their underlying logic, but that specific, practical challenges might undermine their ability to do so.

Two additional studies highlighted the challenges created by weaknesses in an intervention's theory of change. These included a lack of clarity over goals informed by differences between the ambitions of policymakers and practitioners' perceptions about what was feasible; and a disconnect between an intervention's activities and its intended outcomes.

Caution is needed when seeking to transfer the findings of these studies to other contexts, as they focus on specific interventions delivered in specific settings. The findings cannot therefore be assumed to be relevant to all case management interventions. However, the broader finding pointing to the importance of interventions having a clearly defined theory of change is likely to be relevant to all interventions, regardless of the context in which they are delivered.

*Implementing case management tools*

The review identified a reasonable amount of research on those factors that shaped the implementation of case management programmes and associated tools (Objective 2b). Although the strength of the evidence may not be robust, a body of primarily qualitative research has developed that provides insights into what facilitates and inhibits the implementation of case management tools and approaches, and has begun to identify moderators or contextual factors that inform this process. Relevant facilitators and barriers are set out in Table 12 and are examined in more detail in the discussion below.

**Table 12:** Implementation facilitators and barriers.

Tool/implementation	Case management stage		
	Tool	Facilitators	Barriers
**Client identification**	Outreach work		1. External actors (youth workers): lack of trust from state actors; short‐term contracts limiting continuity & buy‐in 2. State actors (police): managing competing aims of care & control
**Client assessment**	Screening tools	1. Multiple assessors able to capture more information helpful for case planning 2. Clear, standardised & shared understanding of eligibility criteria	1. Subjective language
	Multi‐agency client assessment	1. Effective communication processes to enable information sharing & support coordination 2. Availability of risk assessment tools 3. Trusted relationships enabled by strong leadership; a clear mandate; regular meetings over time; positive interpersonal relationships	1. Inefficient information sharing between partners 2. Limits on information sharing by police/security agencies 3. Inconsistency & subjectivity in assessment processes 4. Time & scheduling pressures 5. Power differentials & hierarchies between stakeholders 6. Unconscious bias 7. Confirmation bias 8. Excessive bureaucracy
	Risk & needs analysis (RNA) tools	1. Use of tools tailored for violent extremism & terrorism 2. Supplementing assessments informed by RNA tools with professional judgement 3. Practitioners with relevant expertise & experience of terrorism cases supports assessment of recidivism risk, disguised compliance & relevance of risk factors 4. Acknowledging different levels of knowledge & experience of those from different disciplines/agencies 5. Benefits of training in developing skills & confidence 6. Using multiple assessors 7. Formal & informal support for assessors	1. Inconsistent use of RNA tools 2. Uncertainty around utility of RNA tools 3. Concerns over nature of risk & protective factors 4. Little guidance on interpreting patterns of risk factors 5. Limited guidance on how RNA assessments can be used to support case management/planning 6. Lack of clarity over definitions of risk factors 7. RNA tools unable to address challenge of subjectivity in client assessment
**Case planning**	Intervention planning tools	1. When informed by client assessments these can inform appropriate levels of monitoring and reporting	1. Lack of consistency between case plans and risk assessments
	Multi‐agency case conferences	1. Trust between stakeholders 2. High levels of motivation 3. Sufficient expertise in the group 4. Structured meetings overseen by a neutral chair 5. Appropriate levels of information sharing 6. Positive working environment 7. Equality amongst stakeholders 8. An established team which has worked together over time 9. Clear mandate and leadership 10. Good interpersonal relationships	1. Absence of clear, commonly agreed goals 2. Shortage of time, finances and people 3. Stakeholders prioritising their own/organisation's interest 4. Overly bureaucratic processes 5. Difficulties over information‐sharing 6. Hierarchical struggles that undermined trust
**Implementation/delivery**	Tailoring intervention services & goals	1. Combining formal & informal types of support 2. Agreeing realistic and/or motivational goals 3. Identifying services consonant with intervention goals & the causes of individual radicalisation 4. Identifying services relevant to different levels of a client's social ecology 5. Combining one‐to‐one activities with group work 6. Engaging with the client's family 7. Appropriate sequencing of intervention plans	
	Practitioner characteristics & approaches	1. Matching practitioners with clients who have some shared characteristics such as language, religion, subject matter knowledge and/or lived experience 2. Effective listening skills 3. Belief in the client & their capacity to pursue a positive future 4. Commitment & flexibility 5. Empathy & authenticity 6. Continuity in the relationship between practitioners & clients across the case management process 7. Spending and committing time to the client	1. Having a dual role, e.g., as an assessor & social worker 2. Difficulties understanding when practitioners had ‘done enough’ 3. Overwork & stress 4. Tensions caused by a lack of organisational understanding of the additional time needed to work with terrorism offenders
	Practitioner supervision & quality assurance	1. Structured methods of selecting & recruiting mentors 2. Intervention providers working in pairs to provide oversight, safety, sharing alternative perspectives, and assess the authenticity of clients 3. Organisational, managerial & peer support to help mitigate emotional toll of intervention delivery 4. Formal debriefing sessions, supervision of practice & engaging with psychologists 5. Experience & expertise support independently functioning teams	1. Poor working relationships with managers 2. Ineffective management structures 3. Lack of communication between stakeholders 4. Absence of a competency framework
**Monitoring & evaluation**	Client assessment tools	1. Structured assessment tools can help monitor change and progress towards programme goals & facilitate case management interventions 2. Triangulation with other data sources may help detect false compliance	1. Subjectivity in assessment processes 2. Unconscious & confirmation biases 3. Inconsistency in the tools used to monitor client progress across stakeholders involved in delivery 4. Lack of longitudinal monitoring limits assessments about long‐term outcomes such as recidivism 5. Failure to complete risk assessments before an intervention begins means there is no baseline against which to assess change
	Case file & case note data	1. Case notes provide a means of monitoring progress 2. Multiple types of data can be captured in case files including practitioner feedback, quality of client engagement, & their behaviour & attitudes 3. Triangulating different kinds of case file data may help detect false compliance 4. Processes to systematise the process of capturing case data can facilitate monitoring and evaluation 5. Input from multiple practitioners can reduce the possibility of bias from single practitioner reports	1. Inconsistency in the quality & content of case notes can limit their ability to assess progress & effectiveness 2. May negatively impact relationships between practitioners & clients when notes are taken in meetings 3. Some jurisdictions' ethical & legal restrictions may prevent some information, such as religious orientation, from being recorded
	Case conferences	1. Can enable ‘plausibility checking’ to interpret & monitor client progress 2. Enable case reviews & light‐touch internal audits	
	Less structured qualitative data		1. Lack of agreement between stakeholders over which measures are most appropriate indicators of client progress & outcomes 2. Lack of systematic approaches & subjective criteria to monitoring & evaluating clients can lead to disagreements between practitioners over levels of progress & risk
**Transition/exit**	Exit & aftercare approaches	1. Inter‐agency coordination supports ongoing case management of former intervention clients 2. Continuity of support during the pre‐release and post‐release period facilitated exit processes	1. Challenges associated with ending the relationship between practitioners & clients smoothly 2. Fear of closing cases too early in error
	Post‐exit & post‐release processes		1. Difficulties monitoring clients on release 2. An absence of post‐intervention monitoring

*Stage 1: Client identification*

Two barriers were identified in two studies that examined the processes by which individuals are identified and referred to case management interventions. Both barriers speak to the difficulties of finding appropriate actors to undertake this work. First, the difficulties posed by working with external actors on the basis of short‐term contracts, which limited their long‐term commitment to the intervention, and by institutional mistrust of those external to the state agencies that managed this work. Second, the challenges of using police officers to fulfil this function due to the differing commitments of care and control that the police are subject to.

*Stage 2: Client assessment*

Twenty‐six studies looked at client assessment processes, with an emphasis on multi‐agency client assessment (*n* = 14 studies) and risk and needs analysis tools (*n* = 12), alongside a smaller number of studies that looked at screening tools (*n* = 3). Factors which facilitated screening tools included a shared, clearly defined, often standardised set of criteria to determine who is eligible for an intervention, and the perceived benefits of having more than one person involved in the screening process. Overall, these processes were believed to reduce the potential of inappropriate and/or subjective referral decisions, and identify those most at risk of radicalisation or terrorist recidivism, and/or best able to benefit from the intervention.

Fourteen studies identified factors which facilitated or generated barriers to multi‐agency collaboration and client assessment. All of these studies considered multi‐agency working to be a valuable tool to support client assessment and reduce the potential for mistakes. Factors which supported multi‐agency collaboration included effective communication processes; the availability of risk assessment tools; and trusted relationships between those representing different agencies. These relationships were enabled by strong leadership, a clear mandate, and regular meetings which allowed positive, reciprocal relationships to develop over time.

Identified barriers to effective multi‐agency working included inefficient or limited information sharing between partners, particularly when working with the police or security agencies, and inconsistency and subjectivity in assessment processes. Power differentials between stakeholders were also considered able to undermine relationships between partners, and lead to authority bias where those with greatest power are assumed to be correct. More practical barriers included excessive bureaucracy, and time and scheduling difficulties. Finally, unconscious bias was considered to potentially lead to certain identity groups – particularly Muslims – being disproportionately considered at greater risk of radicalisation, whilst confirmation bias may see stakeholders foreground evidence that supports pre‐existing assumptions.

Risk and needs assessments (RNA) were examined by twelve studies. Factors which facilitated the use of RNA tools included the use of tools designed specifically for countering radicalisation to violence, as standard measures were considered less able to adequately capture factors relevant to this context. Having knowledgeable and experienced practitioners helped to facilitate assessments, as well as having the potential to identify disguised compliance. Training was therefore considered valuable, as was the use of multiple assessors, and the provision of formal and informal support for assessors to help mitigate some of the pressures associated with this role. Finally, an awareness of differing levels of knowledge and experience across stakeholders was considered important in facilitating assessments.

Barriers to client assessment processes included the inconsistent use of RNA tools, and an associated uncertainty as to how valuable they were. Conceptual barriers were also identified, such as a lack of clarity over how risk factors were defined, and uncertainty as to whether the most appropriate factors were covered in RNA tools, with particular concerns about the neglect of protective factors. A lack of guidance around how to interpret patterns of risk factors as well as limited support to help practitioners understand how RNA might be able to support case planning and case management were also considered potential implementation barriers.

There were some areas of debate in the literature on RNA tools. Several studies suggested that subjectivity in how clients were assessed acted as a barrier to effective risk assessment, whilst one study presented the views of some practitioners who suggested that it was useful to allow for more subjective processes that took greater account of professional judgement.

*Stage 3: Case planning*

Case planning received less attention in the literature: a total of five studies looked at tools used to develop individualised case plans for clients. Three of these examined intervention planning tools, and found that it was important to ensure the outcome of the client assessment stage fed into the case planning process. The second tool used to inform case planning was case conferences. Many of the same themes identified in the work on multi‐agency working in the assessment stage were identified in research on case planning, including the benefits of trust, motivation, expertise, appropriate information sharing arrangements, structured regular meetings informed by a clear mandate and overseen by a neutral chair, as well as equality amongst stakeholders, good interpersonal relationships developed over time, and a positive working environment. Conversely, the lack of commonly agreed goals, a shortage of resources, difficulties sharing information, hierarchical struggles and overly bureaucratic processes all had the potential to act as barriers to case conferences used to inform case planning.

*Stage 4: Delivery and implementation*

A total of twenty‐eight studies looked at the delivery stage, including research that examined the tailoring of intervention services and goals (*n* = 19 studies); practitioner characteristics and approaches (*n* = 20) and practitioner supervision and quality assurance processes (*n* = 13).

Factors facilitating the delivery and implementation of case management interventions include structured methods of recruiting mentors to ensure the most appropriate individuals are selected for this work, and experience and expertise of working with radicalised individuals. The importance of adequately supporting practitioners who deliver this work was identified across the different tools that helped deliver interventions. This support included organisational, managerial and, in particular, peer support to help manage the emotional toll of working with what can be a challenging population. In contrast, poor working relationships with management, ineffective management structures, poor communication between stakeholders, and the absence of a competency framework were identified as barriers to delivery.

*Stage 5: Monitoring and evaluation*

Sixteen studies considered monitoring and evaluation tools, of which nine looked at client assessment tools; seven focused on case file and case note data; five on case conferences; and five on other, less structured forms of qualitative data.

Among those factors with the potential to facilitate monitoring and evaluation were the availability and use of structured assessment tools able to help monitor change, inform evaluations, and support the delivery of interventions. The detection of false compliance may be aided by triangulating different sources of monitoring data. Case notes and case files able to capture multiple forms of data, including practitioner feedback, information on the quality of the client's engagement, and their behaviour and attitudes can also support delivery. As can input from multiple practitioners which helps reduce the potential of biased reports from individual stakeholders. In addition to those benefits associated with their use during the assessment and case planning stages as outlined above, case conferences can support monitoring and evaluation by enabling plausibility checking over client progress, and supporting case reviews and light‐touch internal audits.

Potential barriers to monitoring and evaluation include subjective assessment processes, biases, inconsistency in the quality and content of information recorded, uneven use of tools to monitor client progress, and failing to complete risk assessments before an intervention has started which means there is no baseline against which to assess change. The absence of longitudinal monitoring limits assessments about long‐term outcomes such as recidivism. A lack of agreed measures of client progress can create a barrier to monitoring and evaluation processes, as can the absence of systematic approaches and the use of subjective criteria to interpret progress which in turn can lead to disagreements between practitioners over levels of risk and progress.

*Stage 6: Transition and exit*

Ten studies examined the transition and exit aspect of interventions and identified two factors which facilitated this process: inter‐agency coordination, and continuity of support during the pre‐release and post‐release periods. Potential barriers included fears over closing cases too early and concern over the consequences of making the wrong decision, as well as the challenges associated with ending the relationship between practitioners and clients smoothly. Finally, in the post‐exit context, difficulties monitoring clients after their release from prison generated practical barriers, as did a lack of data on the position of former clients.

*Implementation factors across the case management process*

The review also identified factors that affected the full case management process. These factors included multi‐agency working; risk‐oriented logics; public and policy pressure; the intensity of intervention work; resourcing; staff expertise; voluntary and mandatory interventions; and broader legislation. This aspect of the review also considered the moderators, or contextual factors, that were relevant to case management interventions including delivery context; local context; standalone interventions; and client challenges.

*Multi‐agency working*. All of the interventions covered in the review incorporated some form of multi‐agency working, and 34 studies examined how this operated in practice. In some cases, multi‐agency working structured one or a number of stages of the case management process. In others, they were central to the entire process of managing interventions. Twenty‐three of the studies that looked at multi‐agency working suggested it helped to facilitate interventions.

Implementation factors considered to facilitate multi‐agency working included visibility, so that interventions were known to internal and external stakeholders and were considered an appropriate source of expertise and support; and efficient information sharing between partners, which was supported by the development of codified rules, secure data transfer systems, trust between stakeholders, and knowledge about the rules associated with sharing information.

Having a shared understanding of clearly defined intervention goals and of the intended outcomes of multi‐agency working were considered facilitators, and their absence a barrier. Reviewing and clarifying outcomes and goals through logic models was one means of addressing this issue. Differing organisational cultures, where practitioners are focused on different types of goals – for example public protection and rehabilitation – had the potential to create barriers to effective multi‐agency working. Positive working relationships facilitated case management processes and were supported by training around the working practices and mandates of other organisations, as well as efforts to develop trust between practitioners from different agencies.

Barriers to implementing multi‐agency working were comparable to those identified at individual stages of the case management process, most notably issues associated with information sharing, where the less transparent processes used by the police and security agencies inhibited information sharing, as well as confidentiality rules which had the same effect, but typically involved those from healthcare and social work. Having large numbers of actors involved in multi‐agency meetings was identified as a possible barrier to sharing relevant information, whilst ensuring the right partners with the relevant expertise and capacity to address specific case management functions were involved in multi‐agency processes facilitated this process. Power differentials and hierarchical struggles, often between the police and other stakeholders, again had the potential to create barriers to implementation, as could a lack of clarity over the responsibilities and jurisdictional boundaries of different partners.

*Risk‐oriented logics*. Fifteen studies examining tertiary interventions discussed the use of risk‐oriented approaches. A third of these studies identified potential barriers generated by a preoccupation with short‐term efforts to manage and control risk, noting how this focus on risk can come into tension with longer‐term rehabilitative goals. Four studies discussed risk‐oriented approaches to secondary interventions, and again highlighted how risk logics can contribute to risk aversion which can bump up against rehabilitative goals, particularly where professionals from non‐security fields were concerned. Client‐centred approaches using strengths‐based and desistance‐informed approaches had the potential to mitigate these implementation barriers.

*Public and political pressure*. The particular public attention paid to counter‐radicalisation interventions was identified as a potential barrier to implementation by ten studies. Public scrutiny has the potential to make practitioners adopt a more risk averse attitude, whilst the high‐profile nature of these interventions could negatively impact practitioners' willingness and confidence to engage in this work. Public debates around the risk of disguised compliance were also considered potentially disempowering. Risk aversion may lead to interventions diverging from their underlying logic, for example, by not reducing supervision where levels of risk are seen to dip because of concerns over the repercussions should an individual reoffend.

Political and media scrutiny has the potential to create a barrier to constructive relationships between practitioners and clients who may be sceptical of efforts to work with them, particularly if the client feels that decisions are being made due to political considerations rather than because of a genuine assessment of their progress. The stigma associated with a terrorism conviction has the potential to undermine reintegration and rehabilitation options.

*Resourcing*. Seventeen studies drew attention to the barriers that a shortage of resources, time, staff or adequate support for staff can create. Resource‐related barriers were described as particularly challenging in contexts with less well‐developed CVE infrastructures; for newer interventions; and where practitioners' roles in interventions were less well embedded in existing systems. The potential for overwork and stress were also identified as barriers.

Time pressures can lead to inadequate preparation and guidance, and a poor understanding of intervention goals. A reduction or lack of sustainable funding was also identified as a challenge, particularly where this meant practitioners were employed on short‐term contracts and did not have clarity over their expected caseloads or salary. These issues were described as a particular challenge given the perception that radicalised clients need more supervision and support.

*Staff expertise*. Staff expertise was considered an important facilitator by twenty‐three studies. The diversity of knowledge held in multi‐disciplinary teams was considered helpful, as was transferable expertise gained from working with other kinds of clients, alongside experience of engaging with at risk or radicalised individuals. Relevant language skills were identified as a facilitator (and their absence a barrier), in enabling practitioners to make informed risk assessments and develop a positive relationship with the client.

Sixteen studies identified training as an important facilitator, including specialised training on countering radicalisation to violence and knowledge of other relevant techniques such as motivational interviewing, multi‐agency collaboration, or broader skills such as counselling. Barriers associated with staffing included challenges recruiting and retaining staff with the necessary expertise, and a lack of specialist knowledge, in particular in relation to ideology. Drawing on external experts has the potential to address this barrier.

*Voluntary and mandatory interventions*. The barriers and opportunities of voluntary and mandated interventions were considered by eleven studies. This research suggested practitioners had a preference for voluntary approaches as these were considered better able to elicit the motivation needed to change. However, practitioners delivering voluntary interventions also described barriers, including an inability to compel individuals to participate in programmes, and difficulties encouraging people to engage with interventions once enroled.

*Broader legislation*. Eight studies considered the impact of broader counter‐terrorism legislation. This research drew attention to the barriers counter‐terrorism powers can generate when trying to pursue rehabilitative aims, including where clients are subject to harder forms of counter‐terrorism intervention which may negatively impact their motivation to engage with efforts to support their reintegration. Where clients are not adequately prepared, the imposition, but also the lifting of sanctions can create a barrier to rehabilitation efforts. Although in some cases these sanctions were considered able to facilitate case management processes, particularly if used selectively and as part of a well‐coordinated multi‐agency approach.

*Moderators across the case management process*

Moderators, or those contextual conditions that influence how case management interventions are implemented, included the delivery context, local context, whether an intervention was standalone, and client challenges that impacted their ability to engage with the intervention.

*Delivery context*. The role delivery contexts played in case management interventions was considered by eleven studies. The specific features of correctional settings had the potential to generate barriers to delivering interventions in this context, including the impact controls placed on individuals can have on the ability of practitioners to develop constructive relationships with clients, and the ability of prisoners to take part in rehabilitative activities.

The negative impact that prison conditions can have on prisoners can also act as a potential barrier. Providing expedited access to support services can help mitigate this challenge. Whilst those on probation do not face these challenges, the greater freedom individuals have can make monitoring and assessment harder. Institutional contexts able to facilitate interventions are characterised by good conditions and clearly communicated values and principles which guide decision making. Finally, operating in conflict affected contexts where violent extremist groups were still active has the potential to undermine efforts to promote long‐term disengagement.

*Local context*. The influence of local contexts was considered by ten studies which described the benefits of tailoring interventions to local conditions or the CVE infrastructure in a given country in ways which take account of differing levels of resources, expertise and risk. Being sensitive to the relative availability of employment opportunities or the services available in local neighbourhoods was considered helpful, as was the presence of an effective local coordinator. Tensions between different regional authorities and the quality of cooperation between agencies had the potential to create barriers to implementation.

*Standalone interventions*. Four studies considered the role of standalone interventions, as distinct from those integrated into existing organisational structures offering broader support. Delivering an intervention through an organisation that was already well‐established in a local area was considered able to facilitate delivery. In the absence of this sort of organisation, standalone interventions may overcome this barrier by becoming known in the area, nurturing a good reputation, and employing a positive public relations approach. However, a lack of CVE‐specific support can act as a barrier to supporting those convicted of terrorism offences.

*Client challenges*. Challenges in clients' lives can create barriers through the impact they have on an individuals' ability or willingness to engage with an intervention. Challenges described in the research include mental health problems; addiction and substance abuse; and a breakdown or absence of supportive relationships. The dynamic nature of these challenges means interventions benefit from taking a responsive approach that aims to accommodate and address challenges as they emerge rather than seeing them as permanent setbacks.

#### 4.3.2 Overall completeness and applicability of evidence

The studies identified through the review only allowed us to respond to Objective 2 on the factors that shaped the implementation of case management interventions. No studies were identified that enabled us to speak to the effectiveness of different tools and approaches (Objective 1). A modest number of studies (*n* = 7) allowed us to draw some insights into the assumptions underpinning the implementation of case management approaches (Objective 2a), but no research was identified that enabled the review to draw conclusions as to what influenced the implementation of different approaches (Objective 2b). Research relating to Objective 2b instead examined the implementation of specific case management tools and specific stages of the case management process (*n* = 43) and those factors which facilitated, generated barriers, or acted as moderators across the full case management process (*n* = 41).

The research included in the review was international in nature. Studies reported on interventions in seventeen countries, and six examined more than one country context. Most of these studies were based in the Global North so whilst the review is not representative of CVE initiatives internationally, it has gone some way to develop a broader evidence base on secondary and tertiary interventions operating across the world. The inclusion of languages other than English supported this ambition. Secondary and tertiary interventions were almost equally represented in the review (*n* = 12 and *n* = 14 respectively), whilst the remaining 21 studies examined the use of tools and/or approaches that spanned secondary or tertiary prevention.

This review has demonstrated that much more attention has been paid to client assessment (specifically risk assessment tools and methods); aspects of the delivery and implementation process; and to a lesser extent, tools to support monitoring and evaluation. Client identification, case planning, and exit and transition processes have received less attention. An additional finding is the limited efforts to develop and deploy theories of change or logic models, and the often implicit and hybrid nature of underpinning frameworks such as RNR or strengths‐based models. Together these issues speak to the organic way the CVE field has evolved, and the relative absence of agreed systems, processes, or measures of success, all of which present challenges when trying to integrate a wide body of work into a structure that may not have been considered when the tools and approaches discussed in this review were developed.

One notable limitation is the lack of research examining the potential unintended consequences of case management tools and approaches. Broader research examining policy and practice has pointed to the potentially negative impacts that counter‐radicalisation work might have on individuals and communities (e.g., Heath‐Kelly, 2013; Abbas, 2019). Although these studies provide initial data pointing to the potential for case management interventions to generate these effects, robust empirical research examining whether case management tools and approaches are producing these effects is lacking. None of the eligible studies examined these potential impacts in any detail, which means we are unable to comment on whether and how these unintended consequences might play out. It is crucial not to overlook the concerns that have been raised, making more empirical research necessary to better understand the scope of these issues, and how they might be addressed.

#### 4.3.3 Quality of the evidence

The quality of evidence in the review was uneven. In comparison to more typical Campbell systematic reviews, this review included a broader range of research designs, including quantitative and, predominantly, qualitative designs that are traditionally considered unsuitable for inclusion in systematic reviews of intervention effectiveness, and we did not identify any eligible studies that used experimental or stronger quasi‐experimental designs. In the context of a traditional review of intervention effectiveness, these types of designs can be understood as having important methodological weaknesses, and/or elevated risk of bias. The results of our analysis should therefore be read with these potential weaknesses in mind, however these research designs provide strong evidence in relation to implementation, and are therefore not considered ‘weak’ in the context of evaluating implementation, or of our specific, objectives.

Overall, the analysis of implementation (Objective 2) was based on a robust body of qualitative and mixed methods research. As discussed in Section 4.2.2, all of the studies included in this analysis were assessed as being of good quality by the review team, and as having no critical weakness when assessed using the relevant quality assessment tool (i.e., the CASP or EPHPP tools). However, the evidence relating to Objective 2b was more robust than for Objective 2a, with the evidence‐base for the former comprising of 47 studies, compared to only seven for the latter. More research that examines the programme logics underpinning case management interventions, and which assesses the implementation of these logics is therefore badly needed. More research including ‘stronger’ quantitative methods is also needed to explore those themes identified in qualitative studies.

Future research exploring effectiveness will also be important, as the research designs identified through this review are not able to assess whether case management tools and approaches are effective at reducing the risk of radicalisation, recidivism, or terrorism. Similarly, the number of different tools, approaches, and stages of the case management process makes it difficult, on the basis of the current evidence, to determine which aspects might be more or less helpful. Further, although the case management framework seems able to support the process of structuring and delivering interventions, there is little evidence to determine if this is a reasonable assumption to make.

These challenges with the evidence base are due to the lack of an evaluation culture in this field (Baruch et al., 2018), and the significant methodological and conceptual challenges facing efforts to evaluate interventions (Lewis et al., 2020). An additional challenge in trying to understand the wide range of tools, approaches, activities and actors operating in this field is the relatively recent incorporation of the case management framework. Although the language of case management is beginning to be integrated into policy and research, it has not reached a stage where this is the dominant framework used in this space. This means that research is unevenly distributed across different aspects of the case management process and, with some exceptions (e.g., Cherney & Belton, 2021a, 2021b; Cherney, 2022), little effort has been made to use the case management framework to look holistically at CVE programmes, or to integrate policy or research across the different processes that make up interventions.

#### 4.3.4 Limitations and potential biases in the evidence

Part I was designed to capture the broadest body of evidence possible. To do so, it included research in languages other than English and incorporated qualitative and quantitative research designs. Given the aim of looking across the case management process, and the wide number of search terms that sought to capture the range of tools and approaches used in this area of practice, we feel we reduced the chances of missing relevant evidence as far as possible.

Including a broader body of languages did however introduce the potential for bias. There are no agreed guidelines on how to carry out searches in languages other than English (Walpole, 2019). The nature and visibility of databases, grey literature sources and search functionality in non‐English language contexts may vary in ways which are harder to control for. Similarly, there are challenges identifying the most appropriate translations of search terms which may mean some relevant research in the searches in languages other than English could have been missed. Collaborating with subject matter experts with relevant language skills sought to mitigate some of these challenges, however the additional resources needed to enable the identification, translation and analysis of material in languages other than English has the potential to impact how comprehensive these searches might be (Walpole, 2019).

The inclusion of qualitative research sought to overcome some of the limitations of the evidence base. The outcome of the search demonstrated the benefits of this strategy as no methodologically robust quantitative studies were identified. However, identifying, screening, and synthesising qualitative and weaker quantitative research designs carries a number of challenges (see Soilemezi & Linceviciute, 2018) including an increased risk of subjectivity at different stages of the process. The use of double, and sometimes triple coding; working with a team of subject matter experts; and employing a widely used quality assessment tool sought to mitigate some of these challenges, however it is possible that other research teams may have reached different conclusions as to what to include and exclude.

A broader challenge was that that the term ‘case management’ was rarely used in the identified literature, which meant that the research team had to determine what was (and what was not) a case management tool or approach based on the descriptions of the tool or approach provided by the original authors in conjunction with the definition of case management used in this review. This may have led to some bias in our inclusion or exclusion decisions. Whilst we used double, and sometimes triple coding to minimise this risk, the identification of case management tools and approaches remains somewhat subjective. To this end, we hope that the conceptual framework that we presented in the protocol (Lewis et al., 2023), and in Section Objectives, 2 of this review, provides a foundation for future synthesis.

#### 4.3.5 Agreements and disagreements with other reviews

This is the first review to examine tools and approaches used in case management interventions for countering radicalisation to violence. There are therefore no reviews to assess levels of agreement or disagreement against. However, two systematic reviews examined related questions that are helpful to consider in relation to the findings of our review. Hassan et al. (2021b) carried out a systematic review of tertiary intervention programmes. This primarily focused on the overall impact of programmes seeking to reduce violent radicalisation risk, as opposed to ours which looked at the tools and approaches used to support the delivery of these programmes. However, Hassan et al. (2021b) did identify facilitators and challenges to the implementation of tertiary interventions. Our review largely agrees with their findings.

Challenges identified in Hassan et al.'s (2021b) review included inadequate training; uncertainty and a lack of clarity over programme objectives; insufficient human and financial resources; expensive external experts; tensions between staff members; competition between stakeholders in multi‐agency partnerships; short‐term interventions; overwork; concerns over safety; and challenges supporting the reintegration of clients due to the stigma of the offence. Facilitators included the benefits of trust between practitioners and clients; strong working relationships; tailoring interventions to client needs; and engaging with family members. All of these factors were identified in our review of the case management process.

Conclusions from a systematic review of research on multi‐agency programmes with police as a partner for reducing radicalisation to violence (Mazerolle et al., 2021) also aligned with the findings of our analysis around the importance and role of multi‐agency working. Factors found to facilitate multi‐agency working in this context included trusting relationships between partners; a shared understanding of goals; reducing the bureaucratic load practitioners are required to carry; appropriate means of dealing with information and intelligence sharing; and the availability of adequate support and training for practitioners. Our review also found all of these factors relevant to the implementation of multi‐agency working.

## 5 REVIEW PART II – COUNTERING OTHER FORMS OF VIOLENCE

### 5.1 METHODS

#### 5.1.1 Criteria for considering reviews for inclusion in Part II

##### Types of review

Part II aims to identify systematic reviews in the wider field of violence prevention to: assess whether case management tools and approaches are effective at countering interpersonal or collective forms of violence (Objective 3); learn whether these tools and approaches are implemented as intended (Objective 4a); and identify the factors which influence how they are implemented, considering facilitators, barriers and moderators (Objective 4b). This part of the review further aims to assess the transferability of insights from this broader body of literature to interventions seeking to counter radicalisation to violence (Objective 5).

To meet these aims, we carried out an ‘overview of reviews’ (Pollock et al., 2021) which focused exclusively on systematic reviews. Reviews were eligible if they aligned with the Campbell collaboration definition^31^ of a systematic review and met the following criteria:

‐ Reviews use clear inclusion and exclusion criteria, and offer justification
‐ Reviews use an explicit search strategy, specifying the:
  o Stages used to identify research
  o Sources used to identify literature
  o Process for screening studies
  o Number of records identified through the initial searches
  o Number of unique records included in the review
‐ Reviews employ a systematic coding and analysis of included studies which are:
  o Clearly outlined and justified
  o Methods used to carry out meta‐analyses must also be specified

Systematic reviews including randomised and non‐randomised research designs were eligible for inclusion in Part II.

##### Types of participants

Mirroring Part I, there were no demographic or geographic exclusion criteria for a review to be included. Reviews that covered studies focused on participants of all ages, genders, ethnicities and religions were eligible, as were those drawing on data from practitioners, stakeholders and intervention clients or service users.

#### 5.1.2 Types of interventions

Reviews examining case management interventions or tools and approaches used at different stages of the case management process that are designed to prevent engagement in, or promote disengagement from, collective or interpersonal violence were eligible for inclusion.

As set out in more detail in the protocol (Lewis et al., 2023), violence is understood as ‘[t]he intentional use of physical force or power, threatened or actual’ that ‘either results in or has a high likelihood of resulting in injury, death, psychological harm, mal‐development, or deprivation’ (Dahlberg & Krug, 2002, p. 5). It is common to distinguish between collective, interpersonal, or self‐directed forms of violence (Dahlberg & Krug, 2002). This review focuses on collective or interpersonal violence as these are the most relevant to counter‐radicalisation interventions. These are defined as:

a. Collective Violence: Physical, psychological or sexual violence perpetrated by those acting as part of a collective such as gang‐related violence (e.g., Randhawa‐Horne et al., 2019) or larger‐scale militancy (e.g., USAID, 2021).
b. Interpersonal Violence: Physical, psychological or sexual violence perpetrated by individuals (or small groups of individuals) against other people (Mercy et al., 2017), including family members or partners (e.g., Gondolf, 2008).

Interventions designed to address these kinds of violence are increasingly seen as holding lessons for efforts to counter radicalisation to violence. Research has begun to explicitly draw the lessons from, for example, interventions to address larger‐scale militancy or gang violence (Ris & Ernstorfer, 2017; Davies et al., 2017), as well as sexual offending (Cherney et al., 2021) for CVE work. Recognising these potential synergies, reviews in Part II were eligible if they focused on interventions that:

1. Were designed for individuals rather than communities or collectives.
2. Aimed to prevent engagement or re‐engagement in violence.
3. Address interpersonal and/or collective violence.
4. Focus on case management interventions and/or their constituent stages as defined in Section 2.2, including standalone case managed interventions and larger‐scale programmes containing a case management component.

##### Types of outcome measures

Relevant outcomes reported in the reviews echo those used for Part I (see Section 4.1.1), and include those assessing the effectiveness of case management interventions seeking to prevent interpersonal and collective violence (Objective 3), and their implementation (Objective 4).

###### Outcomes relevant to countering violence (Objective 3)

Following Part I, two kinds of outcomes relevant to countering violence were used (Objective 3): primary outcomes designed to understand whether interventions prevented individual engagement in violence, and/or supported individual desistance or disengagement from violence; and secondary outcomes which described the impact of tools or approaches used in case management interventions to support progress towards primary outcomes (see Section 4.1.1 for more on interpreting primary and secondary outcomes).

###### Outcomes relevant to implementation

Implementation was defined broadly, and in the same way as for Part I (see Section 4.1.1). There were no specific outcome measures that determined a review's eligibility. All systematic reviews that reported on implementation factors relevant to the delivery of case management interventions were suitable for inclusion. Similarly to Part I, tools and approaches used to support the delivery of interventions were interpreted in relation to those factors which facilitated, represented barriers, or acted as moderators to implementation.

#### 5.1.3 Search methods for identification of reviews

The search strategy for reviews in English and languages other than English followed a similar process to that set out for Part I and involved the following stages:

1. Identification of search terms.
2. Translation of English search terms into languages other than English.
3. Piloting and revision of English and translated search terms.
4. Targeted search term searches of academic databases.
5. Hand searches of key journals, research outputs of relevant research institutions/professional agencies, and clinical trial repositories.
6. Consulting members of the research team and advisory board to identify studies.
7. Forward and backward citation searching of studies identified at Stages 1–6.

##### Identification and piloting of search terms

An initial set of search terms were identified by the research team and piloted in May 2021 on APA PsycNet. This led to the following approach to identifying appropriate search terms for Part II using four domains which were applied to both the English and languages other than English search processes:

‐ The **Problem** domain sought to capture search terms related to collective and interpersonal forms of violence.
‐ The **Intervention** domain included search terms with synonyms for interventions, tools and different stages of the case management process.
‐ The **Outcome** domain included terms relevant to countering and preventing interpersonal and collective forms of violence.
‐ A **Data** domain was used to ensure that the searches only captured studies referencing systematic reviews.

A full list of English and translated search terms for Part II is available in Supporting Information: Appendix I.

##### Targeted searches of search terms

Search terms were used to search the same databases and parameters as set out in Section 4.1.2. These were supplemented with the Cochrane, Campbell, and PROSPERO databases as these are the most comprehensive databases of systematic reviews.

The same adaptations outlined in Section 4.1.4 were made to the Part II search process to accommodate the limited search functionality of the Ovid and ProQuest platforms. It was only possible to search for research in languages other than English through these platforms using English search terms filtered to identify non‐English language studies. To ensure consistency, two sets of searches were undertaken for the languages other than English: one using English search terms restricted to non‐English language studies, and one using the translated search terms.

##### Searching other resources

In addition to database searches, we used a number of other routes to identify eligible studies. This included carrying out hand searches of the top ten journals with the highest impact factor according to the Web of Science Journal Citation Report 2021 in the category of ‘criminology and penology’ (set out in Table 13); forward and background citation searches of eligible studies; and asking experts in the field to recommend reviews for inclusion.

**Table 13:** Key journals: Criminology and penology.

Journal name
*Trauma Violence & Abuse*
*Annual Review of Criminology*
*Criminology*
*Journal of Interpersonal Violence*
*Youth Violence and Juvenile Justice*
*Justice Quarterly*
*Crime and Justice – A Review of Research*
*Aggression and Violent Behavior*
*Criminology & Public Policy*
*Journal of Quantitative Criminology*

We also drew on the expertise of the research team who were knowledgeable on different linguistic and geographical contexts to identify appropriate grey literature sources. As far as possible, these were searched using the search terms applied to the core database search. However, the variable search functionality of these databases meant that it was not possible to be as comprehensive as the approach used for the search of academic databases.

#### 5.1.4 Data collection and analysis

##### Selection of reviews

The screening process reflected a similar process to Part I (see Section 4.1.3). Search results were imported into Endnote, de‐duplicated and uploaded to Covidence. An initial screening process removed obviously irrelevant reviews and studies that were not systematic reviews, and produced a shortened list of reviews that were screened on title/abstract using the screening tool in Supporting Information: Appendix II. Where there were disagreements between screeners over the eligibility of reviews, these were resolved through discussion and consensus and a final list of reviews went forward for full text review. A similar process was followed for the reviews in languages other than English which was carried out by the relevant language specialists in consultation with the lead reviewer (JL).

##### Data extraction and management

An adapted data extraction and coding tool was used to code the full text of the reviews (see Supporting Information: Appendix IV). This used a more flexible framework than the extraction tool used for Part I on the basis that the assumptions underpinning counter‐radicalisation interventions which guided the development of the coding framework for Part I may not map directly onto the wider field of violence prevention. The lead reviewer (JL) used this adapted tool to capture the main findings of the reviews. As no eligible reviews in languages other than English were identified, this tool was only used for the English language reviews.

A citation matrix was completed to assess whether there was any overlap between the primary studies cited in the reviews included in the overview of reviews. The question of overlap is discussed further in Section 5.2.1 in the description of reviews.

##### Quality assessment of included reviews

Reviews were assessed using the AMSTAR 2 (A MeaSurement Tool to Assess systematic Reviews) quality assessment tool (Shea et al., 2017) which is appropriate for reviews which include randomised and nonrandomised studies. The domains included in AMSTAR 2 were incorporated into the data extraction tool described earlier. All domains were assessed, however, following guidance from AMSTAR 2's developers, greatest weight was placed on the following seven ‘critical domains’ (Shea et al., 2017). Although, inclusion and exclusion decisions were not based solely on the answers to these critical domains:

‐ Protocol registered before commencement of the review.
‐ Adequacy of the literature search.
‐ Justification for excluding individual studies.
‐ Risk of bias from individual studies being included in the review.
‐ Appropriateness of meta‐analytical methods (if review includes meta‐analysis).
‐ Consideration of risk of bias when interpreting the results of the review.
‐ Assessment of presence and likely impact of publication bias.

##### Data synthesis

Data synthesis proceeded in two stages. First considering the findings of the reviews in relation to effectiveness and implementation, and then assessing the transferability of the findings from Part II to Part I.

###### Synthesising evidence for effectiveness and implementation

In line with guidance for Cochrane overviews of reviews, the review presented narrative summaries of the findings from the reviews (Pollock et al., 2021). This did not involve reanalysing the outcome data presented in the original studies but instead set out the evidence relating to the effectiveness of tools and approaches to counter violence (Objective 3), and the process of implementing them (Objective 4).

This approach to synthesising the evidence was organised according to the different tools and approaches reflected in the reviews. The initial intention set out in the protocol (Lewis et al., 2023) had been to develop a typology of tools and approaches against which primary and secondary outcome measures could be mapped and which would help organise the findings from the reviews. However, because so few reviews were identified this was not necessary. Instead we developed narrative summaries of each of the two interventions (mentoring and multi‐systemic therapy), and each of the two tools (risk assessment and polygraph) covered by the reviews.

The narrative reviews include an overview of how the tools, approaches and interventions were intended to work; a summary of data from the review regarding effectiveness and/or implementation factors; a discussion of the strength of evidence for each tool, approach or intervention; and a discussion of the insights relevant to countering radicalisation to violence interventions organised according to the different objectives of the review. This covered insights regarding the effectiveness of case management tools, approaches and interventions, and their implementation focusing on the primary comparable tool – risk assessment tools – and the similarities and differences between the facilitators and barriers to implementation identified across the two parts of the review.

###### Transferability to counter‐radicalisation interventions

To assess whether the findings from Part II were transferable to counter‐radicalisation interventions, we went through two stages. First, we assessed the overall transferability of the research covered in the reviews from Part II to counter‐radicalisation work; and second, we assessed the transferability of the only intervention covered in Part II that was not examined in Part I: multi‐systemic therapy (MST) (van der Stouwe et al., 2014).

To determine whether the findings from the reviews in Part II were transferable we used Munthe‐Kaas et al.'s (2020) framework of transferability. This framework was informed by research which identified a series of themes that were common in work seeking to assess whether findings from primary and secondary research, including systematic reviews, were transferable from one context to another (Munthe‐Kaas et al., 2019). These themes, adapted to counter‐radicalisation work, are set out in Table 14.

**Table 14:** Transferability themes (from Munthe‐Kaas et al., 2019).

Theme	Sub‐themes and description
Population	*Population of interest* At risk of engagement or already engaged in violent extremism *Population characteristics* Information about population of interest, such as their demographic characteristics; type of extremism; whether they have engaged with the intervention voluntarily, etc.
Intervention	*Intervention characteristics* Information about intervention design, stage(s) of case management included, specific tools or approaches used, etc. *Intervention delivery* Information on how intervention is intended to be delivered, such as the settings in which it is delivered, and whether the intervention can be tailored to other types of setting; intensity or duration of the intervention, etc.
Implementation context	*Providers* Number and type of providers delivering an intervention. *Organisations* Information about implementing organisation(s) such as the resources available, size and structure; culture, etc.
Comparison intervention (*if relevant*)	Information about the comparison condition against which an intervention is evaluated, including an assessment of whether the support provided through a control condition is of sufficient quality to provide a robust comparison of effectiveness.
Outcomes	Information about the specific outcomes an intervention is seeking to deliver, and how they are being measured.
Environmental context	Relevant information about, for example the temporal context (e.g., whether there have been any relevant changes in how an intervention operates/or the broader context since a study was conducted); the political, social or regulatory context; or other interventions that might influence the intervention in question.
Researcher conduct	Relevant information about how the research was conducted/how data was analysed which might influence results.

Drawing on this framework, and the research that underpinned it, we assessed the transferability of each review by considering the comparability of the population; intervention, tool or approach; implementation context; outcomes; and environmental context to counter‐radicalisation. Following the protocol, we also considered whether processes of engagement and disengagement for the form of violence studied were comparable to radicalisation to violence, and whether the tool or approach could feasibly be used in counter‐radicalisation work. This same framework was used to assess the transferability of the only type of intervention not examined in Part I: MST. A narrative discussion of the potential applicability of MST to countering radicalisation to violence is presented in the analysis, and is structured around a discussion of the same themes listed above: population, intervention, implementation context, outcomes, and environmental context.

#### 5.1.5 Deviations from the protocol

Four of the deviations identified in Section 4.1.4 in relation to Part I also applied to Part II. First, the search strategy for literature in languages other than English was adapted to include searches for the translated search terms, and searches for the English language search terms filtered on languages other than English. Second, it was not possible to conduct searches using the translated search terms in the Ovid and ProQuest databases, which meant that only English language search term searches, filtered on languages other than English, were conducted on these platforms. Third, studies returned by the searches in languages other than English that had English language titles and abstracts were initially screened by the primary reviewer (JL), and not one of the language specialists. Finally, the discussion of evidence relating to implementation (i.e., relating to Objectives 4a and 4b) was structured around a discussion of facilitators, barriers and moderators in the same way as was done in Part I of the review.

One further deviation from the protocol was specific to the Part II analysis. It was not possible to construct a typology of different tools and approaches as outlined in the original protocol due to the small number of eligible reviews identified, and the fact that over half of the reviews identified (*n* = 5) focused on a single type of tool: risk assessment tools.

### 5.2 Results

#### 5.2.1 Description of included reviews

##### Results of the search

**Figure 3:**
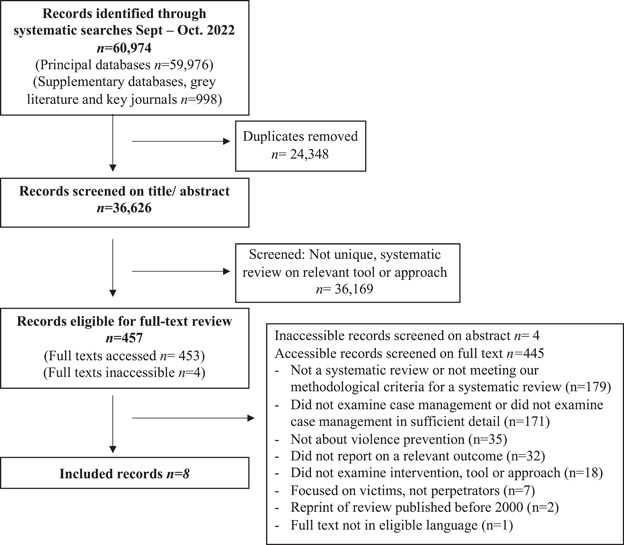
PRISMA diagram of included reviews.

The results of the Part II search and screening process are set out in Figure 3. The initial English database searches identified 55,872 studies. An additional 4104 studies were identified from the languages other than English searches, which included research in French, German, Norwegian, Swedish and Danish, and Russian. These were combined with material identified through the search of grey literature sources, hand searches of relevant journals and consultation with experts (*n* = 998). A total of 60,974 references made up the initial corpus. After de‐duplication, a total of 36,626 references went forward for title and abstract screening using Covidence.

The titles and abstracts of English language material were screened by two members of the research team, conflicts were assessed by a third team member and a decision reached through discussion. Non‐English language material was similarly reviewed by team members with relevant language expertise. This initial screening process led to the removal of 36,169 reviews, leaving 457 which went forward for full text review in Covidence. The full text of four reviews were unavailable in institutional repositories. After assessing the title and abstract of these reviews using the full‐text screening criteria, these were subsequently excluded. The remaining 453 reviews were reviewed by two members of the research team and conflicts adjudicated through discussion with a third team member.

From a total of 453 reviews that underwent full text screening, eight went forward for inclusion in the review, all of which were published in English. No eligible reviews were identified in German, Norwegian, Danish, Swedish, or Russian. Five reviews met the inclusion criteria for Objective 3 on the effectiveness of case management tools (*n* = 3) and approaches (*n* = 2). Seven of the reviews presented data related to whether case management interventions were being implemented as intended (Objective 4a), and/or discussed what influenced how interventions were implemented, focusing on factors which facilitated, generated barriers, or acted as moderators to implementation processes (Objective 4b).

##### Included reviews

A total of eight reviews met the inclusion criteria for Part II. They included five published peer‐reviewed journal articles, and three non‐published studies, including two dissertations and one other academic output. All reviews were published between 2014 and 2022. The number of studies included in each eligible review ranged from 10 to 73, and a total of 172 unique studies were included in these reviews. Eight studies were cited in two separate reviews, and four reviews included at least one study that was cited in another included review. Relevant overlaps between reviews are identified in the analysis to ensure that individual studies are not double counted. The following describes the main characteristics of the reviews covering participants; settings; study designs; intervention types; and outcomes. An overview of the key characteristics of the included reviews is provided in Table 15.

**Table 15:** Characteristics of included reviews (*n* = 8).

Author	Objective	Population	Focus	Type of analysis	Study designs	No. of named databases searched	No. of included studies	Sample	Outcomes
O'Dowd et al. (2022)	Examine the experiences of practitioners engaged in risk assessment and management	Practitioners working in forensic mental healthcare settings	Violence risk assessment and management in forensic mental health settings	Qualitative/narrative	Quantitative and qualitative	5	16	Forensic mental health settings in the UK (*n* = 9), Sweden (*n* = 3), Norway (*n* = 1), Finland (*n* = 1), the Netherlands (*n* = 1) and Scotland/Ireland (*n* = 1).	Implementation factors relating to themes developed using framework synthesis.
Edwards et al. (2015)	Review violence prevention programmes for young people involved in, or at risk of violence that include a mentoring, mediation or peer‐support component	Participants included perpetrators of violence and those at risk of violence who were aged less than 25 years	Interventions including one or more components of mediation, mentoring, or peer support *Only mentoring interventions examined in this review*	Qualitative/narrative	Quantitative: Randomised controlled trials, cluster randomised trials, controlled before‐after studies, cohort studies, case‐control studies.	15	16 (incl. 9 relating to mentoring	Evaluations in the USA (*n* = 13), Netherlands (*n* = 1), Australia (*n* = 1), UK (*n* = 1). Delivered in the community ( = 7), schools (*n* = 5), hospital emergency department and community post‐discharge from hospital (*n* = 2), and juvenile detention facility and in the community post‐release (*n* = 2).	Carrying a weapon, violence, offending, and health service use due to injury. Crime and self‐reported outcomes were included.
Collins (2019)	Examination of the effectiveness of polygraphs in the treatment and management of male sexual offenders	Adult or juvenile male sex offenders who had undertaken a polygraph test	Studies examining polygraph as part of assessment, treatment or management of sexual offence related behaviour or risks	Qualitative/narrative	Quantitative and qualitative	6	10	Conducted in USA (*n* = 6) and he UK (*n* = 4). Average total sample size of 229.1, number of participants ranging from 32 to 635. Average polygraph sample size of 159.9, number of participants ranged from 15 to 342.	Outcomes of polygraph use were disclosure and recidivism. Qualitative studies also explored perceptions of polygraphs.
van der Stouwe et al. (2014)	Examine the effectiveness of MST with antisocial or delinquent juveniles	Antisocial, conduct disordered and/or delinquent juveniles	Multi‐systemic therapy (MST)	Meta‐analysis	Specific quantitative designs: participants had to be assigned into MST and one or more control groups; pre‐ and post‐treatment assessment measures and/or follow‐up assessment measures provided; and statistics suitable for meta‐analysis presented.	3	22	*N* = 4066 juveniles of whom *n* = 1890 received MST treatment and *n* = 1835 constituted the control group. Studies conducted in USA (*n* = 17); UK (*n* = 1); Canada (*n* = 1); Norway (*n* = 1); Netherlands (*n* = 1); Sweden (*n* = 1).	Delinquency. Seven studies used violent delinquency as outcome.
O'Shea and Dickens (2014)	Examine the psychometric properties of the START tool	Mentally and personally disordered offenders	The Short Term Assessment of Risk and Treatability (START) is a risk assessment tool that is not specific to violence, but which captures factures relating to risk of violence	Qualitative/narrative	Quantitative and qualitative	5	Narrative overview *n* = 23: 7 examine feasibility/usability; 2 examine change over time.	Data only provided for meta‐analysis which did not report on relevant outcomes.	Relevant outcomes were (a) user evaluations of feasibility and utility of the START; and (b) change over time.
Levin et al. (2016)	Exploration of the factors that influence the implementation of structured risk assessment instruments in psychiatric, correctional, and community in‐patient settings	Psychiatric, correctional, or community in‐patient settings	Structured Risk Assessment Instruments	Qualitative/narrative	Quantitative and qualitative	3	11	USA (*n* = 3), Canada (*n* = 3), UK (*n* = 3), Australia (*n* = 1), and Norway (*n* = 1). Settings were psychiatric care (*n* = 8); juvenile services (*n* = 1), juvenile probation services (*n* = 1), and correctional probation (*n* = 1).	Implementation facilitators and barriers.
Viljoen et al. (2018)	Examines whether professionals perceive risk assessment tools to be useful for risk management; whether risk assessments guide risk management efforts; whether the use of risk assessment tools facilitate match to the risk and to the need principle of the RNR model; whether the use of risk assessment tools reduces violence or offending; and identifying the strategies are available to improve the utility of risk assessment tools	Psychiatric patients and offenders, professionals	Structured risk assessment tools	Qualitative/narrative	Quantitative only	13	73	N = 31,551 psychiatric patients and offenders, *n* = 10,002 professionals. Countries not specified.	Examined either professionals' risk management efforts following the use of a tool, or rates of violence or offending following the implementation of a tool.
Tarpey (2021)	Examine the use & effects of risk formulation in forensic settings	Any forensic practice setting/service that works with adult service users who are at risk of offending/reoffending/harming others. Exclusions were non‐forensic settings and/or populations, and juvenile population	Use and impact of formulation	Qualitative/narrative	Quantitative & qualitative	10	10	Conducted in the UK (*n* = 8), New Zealand (*n* = 1), and Sweden (*n* = 1). Settings were forensic psychiatric hospital (*n* = 2), custodial settings (*n* = 2); forensic outpatient settings (*n* = 1); and criminal justice community settings (*n* = 5).	Narrative analysis of implementation and effects of risk formulations.

###### Research designs

Five of the reviews included qualitative and quantitative designs of any type (O'Shea & Dickens, 2014; Levin et al., 2016; Collins, 2019; Tarpey, 2021; O'Dowd et al., 2022). In contrast, Edwards et al. (2015), van der Stouwe et al. (2014), and Viljoen et al. (2018) only included quantitative research designs as shown in Table 15. The most rigorous inclusion criteria relating to research design were set by van der Stouwe et al. (2014): participants had to be assigned to a relevant intervention or one or more control groups; studies had to collect data relating to pre‐and posttest assessment measures and/or follow up assessment measures; and studies had to present statistics suitable for meta‐analysis.

###### Participants

Participants of studies included in each review varied, and included practitioners working in a range of settings including forensic and other mental healthcare settings; education and healthcare; and both community and correctional contexts (Levin et al., 2016; Collins, 2019; Viljoen et al., 2018; O'Dowd et al., 2022; Tarpey, 2021). Participants also included clients/service users across these various contexts and settings (van der Stouwe et al., 2014; Edwards et al., 2015; Tarpey, 2021), or those assessed with specific risk assessment tools (Viljoen et al., 2018; Collins, 2019), or with polygraphs (O'Shea & Dickens, 2014).

A range of types of client were examined across the reviews, including perpetrators of violence or related forms of delinquency, or adjudicated offenders (Edwards et al., 2015; O'Shea & Dickens, 2014; Collins, 2019; van der Stouwe et al., 2014; Viljoen et al., 2018; Tarpey, 2021); individuals at risk of violence (Edwards et al., 2015; Tarpey, 2021) or presenting with issues such as antisocial behaviour or conduct disorders (van der Stouwe et al., 2014); psychiatric patients (Viljoen et al., 2018); or some combination of the above (Levin et al., 2016).

A number of reviews set specific inclusion criteria based on age by focusing on adults (Tarpey, 2021) or juveniles only (van der Stouwe et al., 2014; Edwards et al., 2015). Interventions and tools were implemented in different settings including clinical settings such as forensic mental healthcare or psychiatric settings (Viljoen et al., 2018; O'Dowd et al., 2022; Levin et al., 2016; Tarpey, 2021) community settings (van der Stouwe et al., 2014; Edwards et al., 2015; Levin et al., 2016; Tarpey, 2021); or criminal justice and correctional contexts, including probation (Viljoen et al., 2018; Levin et al., 2016; Collins, 2019; Tarpey, 2021).

Countries examined across the included reviews included the USA (Edwards et al., 2015; Collins, 2019; van der Stouwe et al., 2014), Australia (Edwards et al., 2015; Levin et al., 2016), UK (Levin et al., 2016; Collins, 2019; Edwards et al., 2015; O'Dowd et al., 2022; van der Stouwe et al., 2014; Tarpey, 2021), Netherlands (Edwards et al., 2015; O'Dowd et al., 2022; van der Stouwe et al., 2014), Sweden (O'Dowd et al., 2022; van der Stouwe et al., 2014; Tarpey, 2021), Norway (Levin et al., 2016; O'Dowd et al., 2022; van der Stouwe et al., 2014), Finland (O'Dowd et al., 2022), Ireland (O'Dowd et al., 2022), Canada (van der Stouwe et al., 2014; Levin et al., 2016), and New Zealand (Tarpey, 2021). The specific countries examined by Viljoen et al. (2018) were not specified in the review.

###### Interventions

Two of the eight reviews examined different types of intervention: Multi‐Systemic Therapy (MST) (van der Stouwe et al., 2014), and mentoring (Edwards et al., 2015). The remaining six reviews examined specific case management tools. One review analysed the use and effectiveness of polygraphs in the context of monitoring and assessing sex offenders (Collins, 2019), and five reviews examined the use of structured risk assessment and risk formulation tools (O'Shea & Dickens, 2014; Levin et al., 2016; Viljoen et al., 2018; Tarpey, 2021; O'Dowd et al., 2022). This included one review which examined whether the use of risk assessment tools contributed to better intervention outcomes (Viljoen et al., 2018).

###### Analysis

Seven reviews presented narrative, qualitative forms of analysis of relevant measures. Only one review presented a meta‐analysis of relevant outcomes (van der Stouwe et al., 2014). One of the other included reviews also presented a meta‐analysis of the psychometric properties of a specific risk assessment tool (O'Shea & Dickens, 2014). The results of this meta‐analysis are not discussed here as analyses of psychometric properties are not within the scope of our systematic review. Instead, the discussion of this review is limited to its narrative assessment of other relevant outcomes.

###### Outcomes

Two reviews examined whether specific tools or interventions contributed to a reduction in violent delinquency (Viljoen et al., 2018; van der Stouwe et al., 2014). Four reviews presented other outcomes relating to the prevention or reduction of violence such as recidivism, other metrics of (re)offending (Edwards et al., 2015; Collins, 2019; Tarpey, 2021), or change over time (O'Shea & Dickens, 2014). Seven reviews presented data relating to the implementation of tools and approaches by exploring their feasibility (O'Shea & Dickens, 2014; Edwards et al., 2015; Viljoen et al., 2018; Collins, 2019; Tarpey, 2021) and/or examining facilitators or barriers (O'Dowd et al., 2022; Levin et al., 2016).

##### Excluded references

In total, 445 references were excluded at the full text screening stage. Due to the large number of reviews excluded at this stage, the details of the individual reviews are not included here. The reasons for exclusion were: not a systematic review or did not meet our inclusion or methodological criteria for a systematic review (*n* = 179); did not examine a case management tool or approach, or did not examine case management in requisite detail (*n* = 171); not about violence prevention (*n* = 35); did not report on a relevant outcome (*n* = 32); did not examine an intervention, tool or approach (*n* = 18); focused on victims, not perpetrators (*n* = 7); reprint of a review originally published before 2000 (*n* = 2); and full text not in eligible language (*n* = 1).

#### 5.2.2 Quality of included reviews

##### Quality of included reviews

Included reviews were assessed using the AMSTAR II tool described in the methodology. The individual assessments are reported in Table 16. Only two reviews were assessed as having high quality overall, four were assessed as being of medium quality, and two as low/medium quality.

**Table 16:** AMSTAR II ratings of included reviews.

	O'Dowd et al. (2022)	Edwards et al. (2015)	Collins (2019)	van der Stouwe et al. (2014)	O'Shea and Dickens (2014)	Viljoen et al. (2018)	Levin et al. (2016)	Tarpey (2021)
Research questions/inclusion criteria include PICO?	Yes	Yes	Yes	Yes	Yes	Yes	Yes	Yes
Methods established before conduct of review?	Yes	Yes	No	No	No	No	No	No
Selection of the study designs explained?	Yes	Yes	Yes	Yes	Yes	Yes	Yes	Yes
Comprehensive literature search strategy?	Yes	Yes	Yes	Yes	Yes	Yes	Yes	Yes
Study selection in duplicate?	Yes	Yes	Yes	Not stated	Yes	Yes	No	No
Data extraction in duplicate?	Unclear	Yes	No	No	No	Yes	No	No
List of excluded studies exclusions justified?	No	No	Yes	No	No	No	No	Yes
Included studies described in adequate detail?	Yes	Yes	Yes	Yes	Yes	Yes	Yes	Yes
Satisfactory technique for assessing the risk of bias?	Yes	Yes	Yes	Yes	Yes	Yes	Yes	Yes
Report on the sources of funding for the studies included in the review?	No	No	No	No	No	No	No	No
Appropriate methods for statistical analysis?	No meta‐analysis	No meta‐analysis	No meta‐analysis	Yes	Not relevant to review	No meta‐analysis	No meta‐analysis	No meta‐analysis
Potential impact of RoB in individual studies on the results of meta‐analysis or other synthesis assessed?	No meta‐analysis	No meta‐analysis	No meta‐analysis	Yes	Not relevant to review	No meta‐analysis	No meta‐analysis	No meta‐analysis
Did authors account for RoB in individual studies when interpreting/discussing the results?	No	Yes	No	Yes	No	Yes	No	Yes
Discussion and explanation of heterogeneity?	Yes	Yes	Yes	Yes	Yes	Yes	No	No
Investigation of publication bias (small study bias)?	No relevant quantitative synthesis	No relevant quantitative synthesis	No relevant quantitative synthesis	Yes	No relevant quantitative synthesis	No relevant quantitative synthesis	No relevant quantitative synthesis	No relevant quantitative synthesis
Report any potential sources of conflict of interest?	Yes	Yes	No	No	No	No	No	No
Overall assessment	Medium quality	Medium quality	Medium quality	High Quality	Medium quality	High quality	Low/medium quality	Low/medium quality

##### Quality of primary studies included in reviews

Table 17 provides an overview of the different methods and tools that each review used to assess the quality of individual studies. As noted in the original protocol, we did not reassess the quality of studies included in each review. However, where relevant, the analysis sections discuss the limitations or biases of specific studies as identified by the original review.

**Table 17:** Approaches to assessing study quality.

Study	Quality assessment tool	Data	Scoring system	Quality rating	Studies excluded on quality
O'Dowd et al., (2022)	The Mixed Methods Appraisal Tool (MMAT; Hong et al., 2018).	Qualitative & quantitative	Five questions: Can't Tell/No/Yes. Score as percentage of ‘Yes’ answers.	Range: 40%–100%.	None.
Edwards et al. (2015)	Used ‘a modified framework’ based on the EPHPP tools.	Qualitative & quantitative	Assessed on following domains: Allocation to intervention/control; Confounders; Blinding; Data collection methods; Attrition; Fidelity; Follow up.	The authors summarised their assessment of each domain for every included studies, but did not provide an overall quality assessment of each study. Two of the nine mentoring studies rated as high quality. For other study designs, ‘study quality was such that some qualitative inferences about effects of mentoring may be drawn’ (p. 24).	Not stated.
Collins (2019)	Specific tool developed for this research containing similar domains to EPHPP tools.	Qualitative & quantitative	10 measures: No/Can't Tell (0); Partial (1) or Yes (2). Converted into percentage.	Range: 70%–90%.	24 excluded for score <70%.
van der Stouwe et al. (2014)	EPHPP Quality Assessment Tool for Quantitative Studies (EPHPP, undated)	Quantitative	Six domains (selection bias, study design, confounders, blinding, data collection method and withdrawals and dropouts) assessed as weak (1), moderate (2), strong (3). Total score (min = 5, max = 18) reported.	Range: Score of 10–16 out of 18.	Not stated.
O'Shea and Dickens (2014)	Assessed independently by both authors using a procedure adopted by Haney et al. (2012).	Qualitative & quantitative	Only discusses quality assessment for inclusion in meta‐analysis in methods.	Information not available online.	11 studies excluded from narrative review at full‐text stage but all related to quality.
Viljoen et al. (2018)	Cochrane Risk of Bias tool for RCTs (Higgins et al., 2011)	RCT	Low, moderate, high limitations.	Quality ratings of different types of study design is not presented. Overall 18 studies assessed as having high limitations, 30 moderate limitations, and 15 low limitations.	22 excluded for not being empirical, but not specifically on quality.
	National Institute for Health and Care Excellence (2012) (adapted)	Observational and non‐controlled studies	Low, moderate, high limitations.		
	Center for Evidence‐Based Management Critical Appraisal of a Survey tool (n.d.)	Surveys	Low, moderate, high limitations.		
	Evidence‐Based Practice Center of the Agency for Healthcare Research and Quality (AHRQ; Berkman et al., 2015)	Assessment of strength of evidence for each research question	Qualitative assessment.		
Levin et al. (2016)	Used the four elements of trustworthiness identified by Patton (2002); credibility, confirmability, transferability, and dependability.	Qualitative & quantitative	Measures: unsatisfactory, fair, or good.	No overall rating. 10 of 11 studies rated as ‘unsatisfactory/fair’ or ‘unsatisfactory’ on one measure. One study rated as ‘unsatisfactory’ or ‘unsatisfactory/fair’ on all.	Not stated.
Tarpey (2021)	Effective Public Health Practice Project Quality Assessment Tool for Quantitative Studies (EPHPP, undated) (adapted)	Quantitative	13 measures: No/Can't Tell (0); Partial (1) or Yes (2) Converted into percentage.	Range: 15%–73%.	None.
	Critical Appraisal Skills Programme qualitative checklist (CASP, undated)	Qualitative	13 measures: No/Can't Tell (0); Partial (1) or Yes (2) Converted into percentage.	Range: 46%–65%.	

#### 5.2.3 Synthesis of results

The following discussion of the Part II results is made up of three parts: the first part reviews the research on case management approaches, addressing Objectives 3 (effectiveness) and 4 (implementation); the second covers the same objectives for different case management tools; and the third discusses the transferability of these tools and approaches to the field of countering radicalisation to violence, addressing Objective 5 (transferability).

Following Cochrane guidelines (Pollock et al., 2021), the analysis that follows summarises relevant data reported within the included systematic reviews. Where possible, we summarise results drawn from meta‐analyses of outcomes. However, only one review presented a relevant meta‐analysis (van der Stouwe et al., 2014). This meant that data from the remaining seven reviews could only be extracted narratively. When presenting findings in narrative form, we quantify the number of studies reporting on a specific theme, and where relevant, cite the original study or studies that the review refers to in their findings. However, we did not undertake a separate analysis of the individual studies themselves.

##### Case management approaches

The analysis of approaches is split into four sections: Identifying case management approaches; Assessing the effectiveness of case management approaches; Examining the implementation of case management approaches; and Identifying implementation factors and moderators that influence how approaches are delivered.

###### Identifying case management approaches

Two of the eight reviews examined specific types of intervention: Multi‐Systemic Therapy (MST) (van der Stouwe et al., 2014), and mentoring (Edwards et al., 2015). It was not possible to determine whether these interventions were informed by a specific approach as defined in Part I. The analysis of approaches therefore considers evidence relating to the effectiveness and implementation of these two forms of intervention.

####### Multi‐systemic therapy

Whilst van der Stouwe et al. (2014) do not explicitly identify MST as a form of case management, they note that ‘the implementation of MST is highly flexible and designed to address specific individual risk factors’ in a way that adheres with the RNR model of rehabilitation (p. 469). We therefore consider MST to be an example of a tailored intervention that aligns with the core assumptions of case management, and which might be delivered either as standalone intervention or as a component of broader intervention plans that are tailored to individual clients. MST uses a socio‐ecological approach, and is underpinned by the assumption that improving family functioning contributes to better outcomes for juveniles, including those at risk of, or already engaged in, violent behaviour. It is delivered as follows:

Therapists visit the juveniles and their families at home and/or in their community to reduce drop‐out rates, to provide treatment exactly where and when it is needed, and to increase generalizability of newly acquired skills. Moreover, the therapist is available twenty‐four hours a day, seven days a week, and therapeutic sessions may take place up to everyday. MST uses well‐established treatment strategies derived from strategic family therapy, structural family therapy, behavioral parent training and cognitive‐behavioral therapy […] Finally, MST is accompanied by training and supervision, organizational support and adherence measures to monitor treatment integrity.

(van der Stouwe et al., 2014, p. 469)

####### Mentoring

Edwards et al. (2015) examined nine papers which evaluated the effects of mentoring to prevent youth violence. Whilst only one of these papers explicitly described ‘case management’, the mentoring programmes examined in this review were tailored to the needs of clients, and often formed parts of more holistic packages of support, thereby aligning with the basic principles of case management. This review therefore met our inclusion criteria.

###### The effectiveness of approaches in countering violence (Objective 3)

####### Multi‐systemic therapy

The effectiveness of MST was examined through a meta‐analysis of delinquency outcomes across 22 studies, seven of which used violent delinquency as the primary outcome measure, and three of which focused on sex offenders (van der Stouwe et al., 2014). MST was found to have a limited impact on violent delinquency. The authors reported that ‘only significant effects were found if general delinquency was measured and not if specifically violent or non‐violent delinquency was assessed’ (p. 472). However, MST was found to produce ‘uniquely large effects’ on delinquency when used with sex offenders (p. 474). Moderator analysis was only conducted for overall delinquency, which meant that it is not possible to comment on any moderators that might influence violent delinquency. However, it is worth noting that larger effect sizes for overall delinquency were found in better quality studies (p. 474).

####### Mentoring

Edwards et al. (2015) examined the effectiveness and implementation of mentoring interventions using the EMMIE framework (Effect size, Mechanism, Moderators, Implementation, and Economics). Based on data drawn from eight studies that reported on relevant outcomes, they conclude that mentoring may be effective in reducing violence. However, they were unable to quantify an effect size and suggested larger scale evaluations able to control for the effects of different components of support offered as part of holistic interventions were needed. Any overall evidence of effectiveness cannot be attributed to case management due to the inclusion of different approaches in the review. None of the suggested moderators of implementation effectiveness were empirically tested.

###### The implementation of case management approaches (Objective 4a)

Neither the review of MST (van der Stouwe et al., 2014) or mentoring (Edwards et al. (2015) examined whether the interventions were implemented as intended, nor whether the assumptions underpinning the interventions were empirically supported and sound.

###### Influences on the implementation of approaches (Objective 4b)

####### Multi‐systemic therapy

The review by van der Stouwe et al. (2014) did not examine implementation.

####### Mentoring

There was ‘good evidence’ of the following inputs being important for facilitating the implementation of mentoring schemes: the availability of specialist mentoring staff; staff training and supervision; and time spent with youth (Edwards et al., 2015).

##### Case management tools

The analysis of tools is split into two sections: Assessing the effectiveness of case management tools; and Examining the implementation of case management tools. Both sections are structured around the two different case management tools that were examined in the eligible reviews: risk assessment tools (four reviews), and polygraphs (one review).

###### The effectiveness of tools in countering violence (Objective 3)

This section examines three reviews that analysed the impact that risk assessment tools (two reviews) and polygraphs (one review) had on violent offending and recidivism.

####### Risk assessment tools

Two overlapping reviews examined whether the use of risk assessment tools contributed to reductions in violent and/or general offending (O'Shea & Dickens, 2014; Viljoen et al., 2018). O'Shea and Dickens' (2014) analysis of this relationship was based on one study that was included in a larger meta‐analysis of outcome data relating to violent (*k* = 11) and/or general offending (*k* = 4) conducted by Viljoen et al. (2018), and is therefore not examined below.

Viljoen et al. (2018) reported inconclusive results based on an analysis of outcome data drawn from 7350 patients or offenders across 12 studies. These studies were assessed as having high (*n* = 2), medium (*n* = 8), and low (*n* = 2) limitations. The review concluded that the mixed findings meant the evidence was insufficient to argue that risk assessment tools played a direct role in reducing violence or recidivism across both RCTs and nonrandomised trials. Of the 12 studies, seven reported that the use of risk assessment tools had no impact; one reported mixed results; and four reported that use led to a decrease in violence and/or offending.

The authors identified different aspects of delivery context as potential moderators, although not all of them were empirically tested. There was some evidence to suggest that risk assessment tools have a larger impact in settings that have a high base level of violence. The impact of tools may also be affected by differences in how offending is measured. The authors discuss how intervention outcomes might be affected by policy changes: if stricter policies are introduced, individuals may be more likely to be convicted of a new offence, thereby increasing the base rate of recidivism (Viljoen et al., 2018).^32^

####### Polygraph

Collins' (2019) systematic review of the use of polygraphs with sexual offenders reported mixed results when examining the relationship between polygraph usage and recidivism. Three of the ten studies in this review analysed recidivism; two reported data relating to effectiveness (total number of participants = 374). One reported that rates of (a) combined violent and sexual recidivism; and (b) violent recidivism were significantly lower amongst offenders who had received a polygraph than those who had not, but that the effect on sexual‐only recidivism was not significant. The other study found no significant difference in overall recidivism, but offenders who had received a polygraph were significantly less likely to be charged with a subsequent non‐sexual offence. Both studies received a quality score of 78%.

###### The implementation of case management tools (Objective 4a)

This section examines two reviews that analysed whether risk assessments were being implemented in ways that align with their underlying logic by assessing whether they were being used to inform risk management (Viljoen et al., 2018; Tarpey, 2021). The single review on polygraphs did present comparable data, but as this was only drawn from a single study, it is not discussed (Collins, 2019).

####### Risk assessment tools

Analysis of fourteen studies by Viljoen et al. (2018) suggested that risk assessment tools do not always inform decision‐making around risk management. The authors concluded that ‘although tools guide decisions in some contexts, “slippage” often occurs between assessments and risk management’ (p. 191), whereby risk management is not aligned with the results of risk assessments, and/or with the risk level or needs of the individual. Two studies reported high use of risk assessment tools for risk management where over 70% of the sample used the tool for this purpose; and four reported low use where less than half of the sample used the tool. The studies were assessed as having low (*n* = 6), moderate (*n* = 5) and high (*n* = 3) limitations.

The use of tools for risk management varied across different tasks (Viljoen et al., 2018). For example, one of the included studies reported that whilst 80% of professionals used the *LSI‐R* to guide service referrals, only 42% used it to develop re‐entry plans (Haas & DeTardo‐Bora, 2009). There was also some suggestion that specific tools such as the *Historical, Clinical, Risk Management‐20 (HCR‐20)*, a widely used tool that captures 20 risk factors, may be used more frequently than others to inform risk management. The review suggested this was because practitioners may consider tools such as the HCR‐20 as being more pertinent to risk management (Viljoen et al., 2018).

When analysing whether the use of risk assessment tools was associated with a good fit to the ‘risk’ (*n* = 36) and ‘need’ (*n* = 17) principles of the RNR model, Viljoen et al. (2018) concluded that ‘following the use of a tool, match to the risk principle is moderate and match to the needs principle is limited, as many needs remained unaddressed’ (p. 181). However, they also reported that, based on the AHRQ system for scoring the strength of evidence identified in Table 17 above, there was insufficient evidence to conclude whether the use of a risk assessment tool improved the extent to which either principle was met in practice.

Three studies not cited in the Viljoen et al. (2018) review were included in a review of ten studies which included weaker designs (Tarpey, 2021). This review reported that risk formulations were being used to inform risk management in practice, although the quality scores for two of these studies were low (31% and 38%). The higher quality study (73%) also reported that users were positive about the individualised focus of risk assessment tools, and felt that the risk assessment process increased knowledge of, and facilitated access to a wider range of potential treatment options when developing management plans (Judge et al., 2014).

####### Polygraph

As noted above, the single review on polygraphs did not present relevant analysis.

###### Influences on the implementation of tools (Objective 4b)

This section focuses on six eligible studies which examine case management tools which speak to objective 4b. The analysis explores the implementation factors that influence how tools used to support different stages of the case management process are delivered. Discussion focuses on factors that facilitate and support implementation, as well as barriers which undermine it. It also examines moderators that impact the use of different tools.

####### Risk assessment tools

The analysis of risk assessment tools is organised around three themes: the perceived utility of risk assessment and formulation tools; the perceived impact of using risk assessment tools; and implementation factors (facilitators and barriers) and moderators.

######## The perceived utility of risk assessment tools

Three reviews captured feedback from practitioners relating to the overall utility of risk assessment tools (O'Shea & Dickens, 2014; Viljoen et al., 2018; Tarpey, 2021). This included two reviews that examined a range of risk assessment tools, and one that focused on the *Short‐Term Assessment of Risk and Treatability (START)* tool that was originally designed for use in forensic mental healthcare settings, and which assesses twenty items in terms of both ‘vulnerabilities’ and ‘strengths’ (O'Shea & Dickens, 2014).

Findings from these reviews were mixed. This was most clearly demonstrated by Viljoen et al. (2018) who found that eight of the twelve studies (total number of participants = 6,664) that examined the perceived utility of risk assessment tools in supporting risk management reported mixed results. These studies indicated that practitioners assessed the utility of risk assessment tools as a little over the midpoint, somewhere between 5 and 7 out of 10 (p. 187). Only two studies reported that the utility of the assessed tool(s) was high – indicated by most professionals viewing the tool as useful – whilst two reported low utility, where ratings of usefulness for the full sample fell below the midpoint. The review also found that perceptions of utility varied across different risk assessment tools, and across different professions. These studies were assessed as having low (*n* = 5), moderate (*n* = 5), or high (*n* = 2) limitations.

The highest quality relevant study included in Tarpey's (2021) review reported users were positive about the use of structured risk formulations (Judge et al., 2014).

Seven of the twenty‐three studies included in the systematic review of the *START tool* examined feasibility and utility (O'Shea & Dickens, 2014). Ratings for feasibility and utility were largely positive across these studies. The review concluded that users felt additional training would be beneficial, and that it was hard to ‘make fine distinctions between scores on items and specific risk estimates’ (p. 996). Perceived utility and levels of confidence in using the START varied across users in two of the included studies. There were also variations in how different elements of the START were scored.

Findings from the studies in O'Shea & Dickens' (2014) review reported that agreement with different statements relating to the START's clinical utility ranged from 62% to 92.5% (Desmarais et al., 2011), whilst sections of the START tool relating to ‘T.H.R.E.A.T’ (distinguishing between Threats of Harm that are Real, Enactable, Acute and Targeted) and ‘Health Concerns/Medical Tests’ were not considered useful by users (Crocker et al., 2008). Three papers (Crocker et al., 2008, 2011; Doyle et al., 2008) highlighted how users were less confident in using certain aspects of START, such as completing risk estimates.

######## The perceived impacts of risk assessment tools

Two reviews presented data on the broader perceived impacts of using risk assessment tools, both positive and negative, covering a range of effects such as the impact on collaboration, and on relationships between practitioners and service users (Tarpey, 2021; O'Dowd et al., 2022).

Risk assessments and related documentation were reported to increase transparency, support multi‐agency working, and support and add weight to decision‐making (O'Dowd et al., 2022). Criminal justice professionals felt their recommendations were taken more seriously by senior personnel responsible for offender management when supported by a risk assessment and reported that formulations helped them understand the personalities of sex offenders, which in turn informed how they communicated and engaged with them (Tarpey, 2021).

Five of the ten studies examined by Tarpey (2021) discussed outcomes relating to relationships with clients. Four of these studies received a quality score of 35% or lower and are therefore not discussed here. The remaining study found that engaging in collaborative case planning had a positive impact on offender‐practitioner relationships (Shaw et al., 2017 in Tarpey, 2021). This study only received a quality score of 46%, and Tarpey (2021) urged caution in interpreting these results as the study authors collaborated with offender managers in developing these case formulations, and delivered training to them, thereby introducing potential bias to the outcome of the process.

Risk assessment tools were identified as being ‘both an opportunity and a barrier when considering service users' individual needs and resources’ in a review of 16 papers by O'Dowd et al. (2022, p. 39). On the positive side, professionals across six studies saw benefits from the structure and objectivity that risk assessment tools afforded. However, five studies identified potential issues, including suggesting that practitioners can feel constrained by the structure imposed by tools, and pointing to concerns these tools were overly focused on risks, and neglected resources and protective factors. Some level of reluctance in using risk assessment tools was identified across six out of the 16 studies, with six studies also discussing how some practitioners instead relied on ‘clinical intuition’ because they believed their judgement was more valuable.

######## Implementation factors and moderators

Three reviews examined implementation factors that facilitated or acted as barriers to the use of risk assessment tools (Levin et al., 2016; Viljoen et al., 2018; O'Dowd et al., 2022). Two reviews also examined moderators relating to delivery context and the specific cohort being assessed and managed (Levin et al., 2016; O'Dowd et al., 2022).

A range of strategies for improving the ability of risk assessment tools to facilitate adherence to the risk and/or need principle, and/or to support the reduction of violence were identified in 8 of the 73 studies examined by (Viljoen et al., 2018). Training and guidelines were found to contribute to improvements in two of the three studies discussing these topics, and quality implementation contributed to improvements in both studies on this issue. Four studies were assessed as having low limitations, and four medium limitations (Viljoen et al., 2018).

Four main types of ‘implementation determinants’ were found to impact the implementation of Structured Risk Assessment Instruments (SRAI) in psychiatric, correctional, and community in‐patient settings in a review based on eleven studies (Levin et al., 2016).

(1) *Characteristics of the intervention object*, which refers to the features of the tool being implemented. Potential facilitators of implementation captured by this theme included involving staff in the process of producing or selecting the SRAI (*n* = 5); the ability to adapt tools to local needs and practice (*n* = 9); the ability to trial the SRAI through, for example, a piloting process (*n* = 5). Potential barriers included a perceived lack of clinical usefulness (*n* = 2), and the complexity of using SRAIs (*n* = 10) in terms of, for example, tools being time consuming or creating increased workloads due to changing practices.
(2) *Characteristics of individuals* using SRAIs. The most widely discussed barrier related to this theme were knowledge and beliefs about SRAIs (*n* = 10). Previous negative experiences of using SRAIs might reduce perceptions of clinical utility, or a lack of previous experience with SRAIs may make implementation harder. A perceived lack of self‐efficacy was identified as a barrier in two studies. Perceptions about the need for change also had the potential to impact implementation; those who did not perceive any issues with their current practice saw no need to change, and vice‐versa. Some professionals may be sceptical about change due to past experiences. A perceived sense of professional ownership was identified as a facilitator, whereas a perceived loss of professional discretion was identified as a barrier (*n* = 2).
(3) *Characteristics of the inner setting*. Structural factors such as the size and complexity of an organisation, and high staff turnover were seen as potential barriers in two studies, although another study discussed how dedicated members of staff might be appointed to identify and respond to issues as they arise. Staff culture was an important determinant, whereby staff who did not see new routines (i.e., the use of new tools) as part of their job description may be less likely to implement new processes and tools as directed (*n* = 2). Inadequate networks and communication were also identified as potential barriers to implementation (*n* = 4).
  This theme included a number of factors related to the implementation climate including perceptions about the need for change within an organisation (*n* = 5); the compatibility of the SRAI with organisational practices, and with users' values and needs (*n* = 2); clearly communicating and specifying responsibilities, goals and tasks, and providing opportunities for feedback (*n* = 7); leaders' willingness to provide time and space for staff to learn, and to take an active part in implementation (*n* = 6); leadership engagement (*n* = 4); providing sufficient resourcing, including manpower, funding, education, and time (*n* = 4); and providing sufficient access to information and knowledge (*n* = 11).
(4) *Process of implementation*. Facilitators included having a predetermined implementation plan that is sufficiently flexible and adaptable (*n* = 5); involving different stakeholders (*n* = 11), opinion leaders (*n* = 1), and external change agents (*n* = 2); appointing internal implementation leaders (*n* = 10); monitoring the implementation process to increase fidelity (*n* = 10); and reflecting on and discussing the progress of implementation with staff, managers, and other stakeholders (*n* = 6).

Developing a caring or therapeutic relationship between practitioners and service users was a facilitator of risk assessment and management (O'Dowd et al., 2022). In practice, this meant creating a trusting, lasting relationship (*n* = 5) by ensuring that users were well informed and prepared before attending meetings, and involving the service user in risk assessment and management discussions. O'Dowd et al.'s (2022) review also pointed to the challenges in balancing treatment and care against enforcing restrictions (*n* = 5). This included the concern that talking about risk assessment and management with clients might damage the relationship, and frustration from professionals about being asked to deliver assessments for cases they were not involved in. This linked to a broader theme relating to the level of patient involvement in risk assessment (*n* = 2), potential benefits of which included increased transparency, enhanced understanding of risk, and facilitating collaboration.

Multi‐disciplinary working can be both a potential facilitator and barrier to effective risk assessment and management (O'Dowd et al., 2022). Effective multi‐disciplinary working was identified as an important facilitator (*n* = 5) of information sharing, assessing service users' needs in a structured way, capturing different opinions, and developing management plans. Barriers linked to multi‐disciplinary working included individuals interpreting risks differently; communication and information sharing issues; a lack of consistency in how risk assessments were completed; and differing opinions on the validity and utility of various tools.

Barriers to implementing risk assessment tools included difficulties in gaining support from colleagues when raising the importance of protective factors (O'Dowd et al., 2022). The need to complete structured risk assessments was considered ‘another burdensome task’ in a context of high workloads, challenges in changing ingrained practices, a lack of support from colleagues, and time pressures (*n* = 3). One possible outcome of these barriers was less accurate assessments (*n* = 1) (O'Dowd et al., 2022).

Two moderators that were considered barriers to the implementation of risk assessment and risk management were (O'Dowd et al., 2022): challenges in knowing about an individual's past, particularly when they had a history of violence (*n* = 2), and the sheltered environment of forensic mental healthcare settings as potentially inhibiting efforts to support individual needs (*n* = 4), whilst also reducing certainty in risk assessments due to uncertainty in how individuals might act outside of a secure setting (*n* = 1).

####### Polygraph

The analysis of polygraph tools is organised around two themes: the perceived utility of polygraphs as reported by professionals; and by the offenders who are assessed using polygraphs.

######## The perceived utility of polygraphs as reported by professionals

Polygraphs were found by a ‘large proportion’ of case and offender managers to support the process of managing sexual offenders in three out of four studies included in a review by Collins (2019). Other related benefits identified by individual studies included giving practitioners more confidence that offenders were complying with license conditions, and a perception that the use of polygraphs makes it ‘significantly more likely’ that offenders will disclose relevant information (p. 63).

######## The perceived utility of polygraphs as reported by offenders

Offenders saw the polygraph as helping them avoid reoffending or focus on their license conditions in two studies included in Collins' (2019) review. One of these studies also reported negative responses from offenders who believed it to be a mechanism to recall them to prison, or that it was a ‘paper exercise’ (p. 65).

#### 5.2.4 Transferability to interventions seeking to counter radicalisation

The discussion of transferability is split into two sections. The first section examines the extent to which the research covered in the reviews cited in Part II are transferable to case management interventions seeking to counter radicalisation to violence. Transferability is interpreted using the domains set out by Munthe‐Kaas et al. (2019; 2020). The second discusses the implications of the insights set out in Part II for counter‐radicalisation interventions. It describes a series of themes identified in Part II which relate to the objectives of the review, considering effectiveness and implementation of case management interventions in the context of radicalisation to violence. It uses the same transferability framework to look at the one intervention that was eligible for inclusion in Part II that is not already a feature of CVE practice according to the literature reviewed in Part I.

##### Assessing transferability

The transferability of Part II findings to counter‐radicalisation work was assessed across five domains that were identified by Munthe‐Kaas et al. (2019) in the research that informed their framework, and which were integrated into the final framework in some way (Munthe‐Kaas et al., 2020): population; intervention; implementation context; outcomes; and environmental context (Munthe‐Kaas et al., 2019). Two of the domains set out in Munthe‐Kaas et al. (2019) were not assessed: comparator intervention, because none of the reviews assessed relevant comparators; and researcher conduct, both because the reviews did not report sufficient information relating to the conduct of researchers involved in the studies, and because this was not captured in their final framework (Munthe‐Kaas et al., 2020).

The assessment of these five domains lends confidence that the findings from Part II are transferable to counter‐radicalisation interventions. The populations are considered comparable; the tools and approaches discussed in the reviews largely map onto similar methods being used to counter radicalisation to violence; and the implementation contexts and outcomes are similar. However, the environmental contexts examined in Part I and Part II were not always directly comparable, and so caution is needed when considering transferability.

###### Population

Population was understood in terms of the participants who were taking part in the intervention or who were subject to the case management tool or approach (Munthe‐Kaas et al., 2019). All eight systematic reviews examined broadly relevant populations. Reviews presented data relating to work with young people and/or adults who were identified as being at risk of engagement in violence (i.e., secondary prevention) or who had already engaged in violent behaviour (i.e., tertiary prevention) (*n* = 3); sex offenders (*n* = 1); those in forensic mental health settings (*n* = 2); and those across multiple forensic/criminal justice contexts including community, psychiatric and correctional settings (*n* = 2). These are broadly comparable to those populations considered in Part 1 which were differentiated according to whether they engaged with at risk populations (*n* = 12) or those convicted of offences (*n* = 14), or both (*n* = 21).

A number of the interventions examined in Part I spanned multiple forms of violence (e.g., Thompson & Leroux, 2022) or were informed by broader violence prevention work (e.g., Christensen, 2015). Studies in Part I – particularly those working in correctional contexts – reported on interventions that involved working with a range of different clients, including those involved in non‐terrorism related forms of violence (e.g., Stern et al., 2023). This would suggest that lessons emerging from efforts to counter other forms of violence are transferable to counter‐radicalisation work. However, it is important not to generalise about this transferability, as radicalisation to violence is a distinct phenomenon which is likely to require a specialist, tailored response (Davies et al., 2017), albeit a response that might feasibly incorporate approaches developed in other fields of violence prevention.

Given the complexity of the links between mental health problems and terrorism (see Gill et al., 2021) those in mental health settings may be considered a somewhat different population to those involved in counter‐radicalisation interventions, as might sexual offenders. However, as the protocol for this review sets out in more detail, we opted for an inclusive definition of violence which did not preclude the inclusion of tools and approaches for those with mental health problems (Lewis et al., 2023). Nevertheless, we are careful in setting out the limits of the parallels between these different populations and contexts in the analysis.

The definitions used to guide the identification of studies set out in the protocol, and the comparability of the populations from Part I and Part II, which span those at risk and those involved in violence, and those based in different institutional and community settings, support the transferability of insights from the broader field of violence prevention to the counter‐radicalisation context.

###### Intervention

The comparability of the interventions across the two parts of the review was determined in relation to intervention characteristics and intervention delivery (Munthe‐Kaas et al., 2019). The two interventions examined in Part II – MST and mentoring – were both assessed to be transferable to counter‐radicalisation work across both measures.

Mentoring (Edwards et al., 2015) is a common component of many interventions examined in Part I (e.g., Christensen, 2015; Orban, 2019; Fisher et al., 2020). The characteristics and forms of delivery described in the systematic review in Part II (Edwards et al., 2015) are comparable to the mentoring programmes that are often used to counter radicalisation to violence.

Although none of the Part I studies discussed the use of MST as examined by van der Stouwe et al. (2014), there were sufficient parallels with efforts to work with families to counter radicalisation to consider these to be comparable. Not least as working with families to support those at risk, or engaged in violent extremism was an important component of many case management intervention plans (e.g., Cherney & Belton, 2021a, 2021b). Whilst it is not possible to comment on whether MST is likely to be effective in countering radicalisation to violence based on the data available, its underlying assumptions as outlined above appear to align with the socio‐ecological models of counter‐radicalisation discussed in Part I.

The transferability of the six systematic reviews that looked at tools rather than interventions can be assessed by understanding them as part of the materials used in the context of intervention delivery (Munthe‐Kaas et al., 2019). The examination of risk assessment and risk formulation tools presented in Part II has clear transferability to counter‐radicalisation work due to the widespread use of risk assessment tools in counter‐radicalisation interventions discussed in Part I. Whilst this discussion suggested that specialist tools were valuable when working with violent extremists or potential violent extremists, practitioners across different fields are likely to face similar challenges. Furthermore, as the reviews included in Part II provide data relating to the implementation and effectiveness of these tools that was not identified in the counter‐radicalisation space, the analysis of risk assessment tools can help illuminate how and why risk assessment tools may not be used in line with expectations, and whether and how they might help to improve case planning and outcomes.

The transferability of polygraphs to counter‐radicalisation interventions is less immediately obvious. However, the question of disguised compliance was discussed as a challenge across a number of stages in the case management process (e.g., see Section 4.2.6). One of the studies included in Part I highlighted how practitioners working in correctional contexts in some countries may use polygraphs ‘as part of a suite of tactics’ (Cherney et al., 2022, p. 38) to monitor and assess terrorist offenders. Although the same study acknowledged that this tool can generate anxiety in offenders, and may lack predictive accuracy, which are both challenges that have been made of polygraphs in the broader literature (Elvin et al., 2021). Research on polygraphs therefore seems to offer transferable insights for some of the barriers that counter‐radicalisation interventions face.

###### Implementation context

Implementation context is understood in terms of service providers and implementing organisations (Munthe‐Kaas et al., 2019). The studies in Part II involve a range of different settings and practitioners, most of which are also represented in the studies covered in Part I.

Data in Part II is largely drawn from three delivery contexts: community settings, correctional settings, and clinical settings involving correctional, clinical and community‐based service providers. Tools and approaches delivered in community and correctional settings are likely to be particularly relevant to counter‐radicalisation work. The vast majority of studies included in Part I examined one or both of these contexts, and examined the work of a comparable range of practitioners including police, probation, community‐based intervention service providers, mental health professionals and social workers.

Although the insights derived from studies looking at non‐CVE correctional and community contexts have obvious comparability with counter‐radicalisation work, determining the transferability of research drawn from clinical settings is more challenging. Four of the reviews included in Part II focus entirely on, or include studies concerned with forensic or other clinical settings (O'Shea & Dickens, 2014; Levin et al., 2016; Viljoen et al., 2018; Tarpey, 2021).

A number of Part I studies examined collaboration between mental healthcare professionals and other actors (e.g., Hellevik et al., 2022; Sizoo et al., 2022). However, as discussed above, caution is needed when trying to apply the results from these settings to counter‐radicalisation work as clients and practitioners in forensic mental health settings will face distinct challenges that may not be immediately transferable. Given the inclusive definition of violence we adopted in the protocol (Lewis et al., 2023) and the potential for some relevant insights to be derived from forensic settings we have included these reviews in Part II to draw out broader lessons relating to the implementation of risk assessment tools whilst including caveats to this transferability where appropriate.

###### Outcomes

The inclusion criteria for studies to be eligible for both Parts I and II specified that the outcomes had to be directly comparable. This measure therefore meets the criteria for transferability (Munthe‐Kaas et al., 2019).

###### Environmental context

Environmental context covers a range of contextual factors including temporal, regulatory, political and systems contexts (Munthe‐Kaas et al., 2019). The reviews that make up Part II provide less information relating to these contexts than the studies included in Part I. However two of the means of interpreting context – geographic/physical, and systems contexts (e.g., in relation to the type of organisation that delivers the intervention) are described in aspects of the review and indicate that there are grounds for the findings to be transferable.

There is significant overlap in the countries examined in both parts of the review. Although Part I highlighted that there can be variation in how interventions operate across different regions of individual countries (Section 4.2.6.), this type of overlap would suggest that there is some comparability in the environmental contexts. There is also overlap in relation to the organisational/systems contexts within which the interventions discussed in the two parts of the review operated. Both cover correctional and community contexts, whilst Part II also looks at forensic mental health settings.

##### Insights relevant to countering radicalisation to violence

The following draws out the findings from the systematic reviews included in Part II and applies them to the tools and approaches discussed in Part I. It considers the implications and insights regarding effectiveness (Objective 3) and implementation (Objective 4) to meet Objective 5 of the review. This seeks to understand whether tools and approaches used in fields other than CVE might be relevant for counter‐radicalisation work and considers the implications of the findings from Part II for the field of CVE.

Six of the eight reviews in Part II discuss tools and interventions that are already in use in counter‐radicalisation work. In terms of interventions, mentoring is widely used. With respect to tools: risk assessment, risk formulation, and risk management are common aspects of case management interventions. Because of these pre‐existing synergies, rather than assess their applicability, the discussion below draws attention to the similarities and differences between the findings of the two parts of the review to understand whether research from the wider field of violence prevention supports or undermines the use of these tools in counter‐radicalisation work. The two reviews that considered tools and approaches not already in widespread use in CVE focused on the use of the polygraph, and MST, and are considered in more detail below.

###### The effectiveness of case management approaches

The effectiveness of case management tools and approaches remains poorly understood across all fields of violence prevention. No studies were identified that assessed this outcome for Part I, and whilst the evidence base appears to be more developed within the broader field of violence prevention, systematic reviews relating to the use of case management remain limited. Only two relevant reviews were identified (van der Stouwe et al., 2014; Edwards et al., 2015), one of which conducted a meta‐analysis of relevant outcomes (van der Stouwe et al., 2014). When considered alongside the results of Part I, these reviews suggest that counter‐radicalisation interventions are using approaches that are common in other fields of violence prevention. However, it is not yet possible to determine whether these approaches are effective.

With respect to the means of interpreting outcomes, it is notable that the reviews in Part II provide better quality data relating to key outcome measures. In particular, the extent to which risk assessment tools facilitate better implementation and intervention outcomes (Viljoen et al., 2018); the perceived feasibility and usability of different case management tools; and the implementation factors that facilitate or act as barriers to the use of risk assessment tools (Levin et al., 2016; Viljoen et al., 2018; O'Dowd et al., 2022).

The description of Multi‐Systemic Therapy (MST) outlined above suggests that this approach could potentially be applied to secondary and/or tertiary counter‐radicalisation work with juveniles. Using the same framework for assessing transferability as outlined earlier, this approach has been used to engage similar populations (i.e., juveniles and adolescents) as many of those counter‐radicalisation interventions examined in Part I; sees engagement with family members at crucial to the delivery of an intervention, in much the same way as those socio‐ecological forms of intervention examined in Part I (e.g., Cherney & Belton, 2021a, 2021b), and has been delivered in comparable intervention contexts (i.e., settings) and environmental contexts (i.e., countries) to these interventions; and seeks to deliver comparable outcomes relating to a reduction in violence. However, uncertainty around its effectiveness means that caution is needed when considering its potential use for counter‐radicalisation, particularly as some of those studies included in Part I highlighted how attempts to similarly transfer methods that are commonly used with other offending populations to counter‐radicalisation work had proved unsuccessful (e.g., van der Heide & Schuurman, 2018).

###### The effectiveness of case management tools

Twelve studies included in Part I presented data relating to the use of RNA tools for client assessment. However, none of these studies examined whether the use of such tools were associated with more positive intervention outcomes. The review by Viljoen et al. (2018) described in Part II illustrates that evidence of a direct link between the use of these tools and a reduction in violence is mixed. Importantly, this review recognises that ‘it may be unrealistic to expect risk assessment tools to directly reduce violence or offending’, on the basis that ‘tools might be effective only if they enhance the likelihood that individuals receive appropriate, empirically supported interventions’ (Viljoen et al., 2018, p. 204). In turn, they highlight how the quality of implementation is likely to be crucial as to whether risk assessment tools are likely to have positive effects, a point that is discussed in more detail below.

The impact of polygraph usage on recidivism outcomes was similarly inconsistent in the review conducted by Collins (2019). As a result, there is no conclusive evidence to suggest that polygraphs are an effective tool for monitoring and assessing violent offenders of any kind, including violent extremist and terrorist offenders. This is in line with the opinions of practitioners interviewed by Cherney et al. (2022), who stressed that polygraphs ‘are not the deciding factor’ (pp. 38–39) in assessment. Despite this, there was evidence to suggest that both practitioners and service users were largely positive about the use of polygraphs in the context of work with sex offenders (Collins, 2019).

###### The implementation of case management tools

The only tools examined in Part II that were also reflected in Part I were risk assessment tools. Comparing the findings across both parts of the review, there are a number of similar themes. These are illustrated in Table 18 and include facilitators and barriers linked to tailoring implementation; practitioner perspectives; delivery; and the tools themselves.

**Table 18:** Risk assessment tools: Facilitators and barriers across different fields.

	Part I	Part II
**Facilitators**	**Tailoring implementation** 1. Use of tools tailored for violent extremism & terrorism **Practitioners** 2. Benefits of training in developing skills & confidence 3. Supplementing assessments informed by RNA tools with professional judgement 4. Practitioners with relevant expertise & experience of terrorism cases supports assessment of recidivism risk, disguised compliance & relevance of risk factors 5. Formal & informal support for assessors **Delivery** 6. Acknowledging different levels of knowledge & experience of those from different disciplines/agencies 7. Using multiple assessors	**Tailoring implementation** 1. Ability to adapt tools to local needs **Practitioners** 2. Training & guidance considered beneficial 3. Opportunity to trial & pilot 4. Sense of professional ownership **Delivery** 5. Multi‐disciplinary working supporting risk assessment; assessing needs in a structured way & developing management plans 6. Positive relationship between practitioners & service users 7. Involving the service user in risk assessment
**Barriers**	**Practitioners** 1. Uncertainty around utility of RNA tools 2. RNA tools unable to address challenge of subjectivity in client assessment **Delivery** 3. Inconsistent use of RNA tools **Tools** 4. Concerns over nature of risk & protective factors 5. Little guidance on interpreting patterns of risk factors 6. Limited guidance on how RNA assessments can be used to support case management/planning 7. Lack of clarity over definitions of risk factors	**Practitioners** 1. Mixed assessment of utility & validity of risk assessment tools 2. Overly structured approach allows insufficient room for clinical judgement **Delivery** 3. Inconsistent use of risk assessment tools to inform risk management 4. Complexity of using the tools 5. Concerns over length of time needed to complete them & increase in workload 6. Lack of experience creating challenges for implementation 7. Lack of self‐efficacy in practitioners 8. Multi‐disciplinary working leading to individuals interpreting risk differently **Tools** 9. Neglect of protective factors 10. Challenges making fine distinctions between risk assessment scores and estimates of risk

####### Factors facilitating implementation

*Tailoring implementation*: Although most of the tools included in Part II were better established and used standardised approaches to assessing risk of violence amongst different populations, the reviews indicated that the ability to adapt tools to local needs and practice facilitated risk assessment. The research reviewed for Part I found that having tools that were tailored to the assessment of terrorism and violent extremism was beneficial, due to the different patterns of risk and protective factors believed to be relevant for terrorism cases. The benefits of tailoring risk assessment tools to the local context, practice, and type of violence is a theme across both parts of the review.

*Practitioners*: Both parts of the review presented evidence that spoke to the benefits of providing training and support for practitioners to develop skills and confidence, and the ways in which experienced practitioners facilitated risk assessment processes. In addition, Part II described how enabling practitioners to trial and pilot risk assessment tools was beneficial, as well as explaining the positive impact that a sense of professional ownership over the risk assessment process brought. Together this evidence points to the benefits of developing well‐trained, knowledgeable and confident practitioners who have a professional stake in the development and delivery of risk assessment tools.

*Delivery*: Multi‐agency working was found to facilitate risk assessment in both general violence and violent extremist cases (although, see discussion below for some of the ways that multi‐agency working can also generate barriers). Acknowledging the different levels of knowledge and experience across stakeholders was considered facilitative in the CVE space, whilst broader multi‐disciplinary working and its capacity to support risk assessment, structured needs assessments, and the development of management plans was found to be beneficial in Part II's assessment of wider forensic settings. Part II therefore provides more detailed evidence of whether and how risk assessment tools inform (or do not inform) intervention plans that was not identified in Part I, and also provides a more detailed overview of potential explanations for any identified ‘slippage’ (Viljoen et al., 2018).

Part II emphasised the advantages that come with developing positive relationships with service users, and the benefits of involving them in the risk assessment process. Although this was not as strong a theme in the risk assessment stage of the case management process in Part I, the benefits of nurturing trusting and positive interpersonal relationships was a feature of the practitioner characteristics and approaches discussed in Section 4.2.5. The parallels across the two parts of the review suggest there is some evidence that multi‐disciplinary working and positive relationships between clients and practitioners support risk assessment processes.

####### Barriers to implementation

*Practitioners*: Both Part I and II presented mixed evidence as to whether practitioners perceived risk assessment tools as useful. Whilst feedback was generally positive, a number of common challenges were identified across both parts of the review in relation to the perceived utility and validity of risk assessment tools. Similarly both aspects of the review pointed to the challenges associated with the subjectivity that can be part of risk assessment processes. For Part II this focused on the way structured tools precluded clinicians using their own judgement, whilst Part I spoke more to the subjectivity that can be a feature of risk assessment tools used by different practitioners. These findings point to differences in opinion regarding the relative benefits of subjectivity when carrying out risk assessments which are features of both the research on general violence and counter‐radicalisation work.

*Delivery*: Both parts of the review highlighted that the use of risk assessment tools varies, and that such tools are not always implemented as might be anticipated. Part I included studies which touched on this point in passing, however the reviews in Part II provided a more comprehensive examination of this barrier and in particular the inconsistency around when risk assessments inform case planning.

The reviews discussed in Part II drew attention to a number of other issues with the potential to generate barriers to risk assessment processes including the time and resources required to complete assessments; the complexity of the tools; and multi‐disciplinary teams interpreting risk differently. These were not discussed in as much detail in the risk assessment aspect of Part I, but were referenced in the context of the stage of case management within which risk assessment was nested; that of client assessment. These findings suggest there are comparable barriers to risk assessment across the different populations covered in the research in Part I and Part II relating to the challenges associated with inconsistency in use; the resources needed to effectively deliver risk assessments; and some of the issues around multi‐disciplinary working.

*Tools*: The neglect of protective factors was considered a barrier to risk assessment across both parts of the review, as were the difficulties associated with making fine‐grained assessment about the findings of risk assessments and estimate of actual risk. In Part I these issues were a little more extensive, with research drawing attention to the difficulties associated with defining risk and protective factors; the challenges interpreting patterns of risk; and a lack of certainty in how to translate risk assessments into case management plans.

### 5.3 Discussion

#### 5.3.1 Summary of main results

Part II of the review had three objectives: to understand the effectiveness of tools, approaches and interventions used in case management interventions seeking to counter violence; to examine those factors and moderators which impact how they are implemented; and to consider the transferability of these tools and approaches to counter‐radicalisation work.

The analysis in Part II illustrates that case management tools and approaches are being used in a variety of different settings (i.e., community, correctional, clinical), and to prevent different forms of interpersonal violence. However, their effectiveness remains unclear: only five systematic reviews examined the impact of different tools, approaches or interventions on outcomes relating to the prevention of violence and they reported mixed findings.

The presence of these reviews highlights that research examining the use of case management tools and approaches to counter other forms of violence is more mature than research relating to countering radicalisation to violence where no studies, and therefore no systematic reviews of research on the effectiveness of these measures, were identified. The pattern of research identified in Part II was comparable to that of Part I; both illustrate a heavy emphasis on risk assessment tools, and offer more comprehensive analyses of implementation facilitators and barriers than effectiveness and outcomes.

##### The effectiveness of case management tools and approaches

The impact of case management tools, approaches and interventions remains unclear (Objective 3). The two reviews that examined the effectiveness of case management interventions – MST and mentoring – did not find conclusive evidence to suggest they are effective in countering violence. Whilst studies cited within these reviews do present some evidence of efficacy, this was insufficient to draw conclusions about overall effectiveness. Only one review was identified that had conducted a meta‐analysis (van der Stouwe et al., 2014), thereby limiting the conclusions that can be drawn about overall effectiveness.

The three reviews that examined whether the use of risk assessment tools (*n* = 2) and polygraphs (*n* = 1) impacted rates of violent offending reported mixed results. However, the use of these tools alone would not be expected to directly contribute to a reduction of violence. This relationship is more likely to be indirect and would rely on these tools being implemented in ways that supported improved assessment, planning, and monitoring decisions.

##### The implementation of case management tools and approaches

Evidence relating to the implementation of case management tools and approaches was limited. Two reviews examined whether risk assessment tools were being implemented in ways that aligned with their underlying logic (Objective 4a); both assessed whether they were being used to inform risk management (i.e., case planning and delivery). No findings relevant to this objective were identified for other case management tools, or for case management approaches.

These reviews found that risk management is not always directly informed by structured risk assessment. This misalignment may occur when practitioners do not use structured tools, or when there is a mismatch between the results of structured risk assessments and decisions around risk management. There was also some evidence that specific risk assessment tools may be more appropriate for informing risk management decisions, and that risk assessment tools may be used more frequently for some case management tasks (e.g., referrals) than others (e.g., case planning).

One review found that the use of risk assessment tools did not necessarily improve the extent to which the ‘risk’ or ‘need’ principles of the RNR model were met (Viljoen et al., 2018). Implementation factors proposed to explain this included practitioners not taking the results of risk assessments into account when making decisions; deciding only to focus on a small number of ‘high impact’ needs at a time (due to it not being feasible to target all needs at once); or not being able to offer specific services to clients because they are not available to them.

The evidence presented in these reviews suggests that the extent to which practitioners use risk assessment tools to inform case planning will be shaped by their willingness and ability to consider the results of risk assessments when making decisions, and their ability to offer those services that can most effectively target any identified needs or risks.

##### Influences on the implementation of case management tools and approaches

Reviews which captured practitioner perspectives on the perceived utility of risk assessment tools in supporting different elements of the case management process (*n* = 3) and on the impacts of these tools (*n* = 2) reported mixed findings, particularly in relation to whether practitioners saw these tools as being useful to their work. Several reviews identified positive impacts from using these tools, including practitioner perceptions that the use of structured tools helped to support collaboration between different staff and different agencies; enhanced the objectivity of risk assessments; and helped to inform how staff worked with service users. The reviews also reported that practitioners might feel constrained by the structure these tools imposed on their work, and concerns that tools were overly focused on risk factors to the neglect of protective factors (O'Dowd et al., 2022). The review on the polygraph suggested practitioners were generally positive about its capacity to support assessment and monitoring.

Whilst the total number of reviews examining the implementation factors and/or moderators affecting the implementation of case management tools (*n* = 6) and mentoring approaches (*n* = 1) was relatively small, it is notable that the findings of these reviews largely aligned with Part I. The analysis highlighted that mentoring interventions were understood as being facilitated by the availability of specialist mentoring staff; staff training and supervision; and time spent with youth (Edwards et al., 2015). As discussed earlier, many of the implementation factors and moderators identified in reviews related to risk assessment tools aligned with those identified in Part I. Factors that facilitated the implementation of these tools included the ability to adapt tools to local needs; the benefits of training and guidance; providing opportunities to trial and pilot tools; developing a sense of professional ownership; positive relationships between practitioners and service users – including potentially engaging service users in assessments and formulations; and multi‐disciplinary working. Barriers included uncertainty about the utility and validity of risk assessment tools, including concerns about the lack of focus on protective factors, and of tools leaving insufficient room for clinical judgement; the perceived complexity and resource intensity of risk assessments; a lack of experience and perceived self‐efficacy; different interpretations of risk across multi‐disciplinary teams; and uncertainty about how to translate the results of risk assessments into practical risk management actions.

##### The transferability of tools and approaches to counter‐radicalisation

The evidence examined in Part II was assessed as having transferrable lessons for efforts to counter radicalisation to violence, due to synergies in the populations, interventions, contexts, and outcomes that were examined in both parts of the review. Six of the eight reviews included in Part II examined tools (i.e., risk assessment tools) and interventions (i.e., mentoring) that are already widely used in counter‐radicalisation interventions. The remaining two reviews examined a tool (i.e., polygraph) that has been discussed in the context of counter‐radicalisation, but which is not yet widely used and an intervention (multi‐systemic therapy) that had potential transferability to counter‐radicalisation work. However, there is insufficient evidence to argue that polygraphs or MST are effective and should be adopted in this context.

When considered together, both Part I and Part II highlighted that the overall effectiveness of case management interventions and approaches is poorly understood in the context of preventing violence, including radicalisation to violence. However, reviews included in Part II provided better quality data relating to key outcome measures, such as the extent to which risk assessment tools facilitate better quality implementation and better intervention outcomes, as well as additional data relating to the perceived utility of different tools. Whilst the specific risk assessment tools used by counter‐radicalisation practitioners are likely to vary from those used by other practitioners, data drawn from these reviews helped to identify relevant lessons, particularly by highlighting that the quality of implementation is important.

An important finding from Part II was that risk assessment tools are not always implemented in the way that might be expected, and that risk assessment does not always inform risk management. The reviews included in Part II provided further evidence of those factors that might facilitate or inhibit the use of risk assessment tools. Many of these factors overlapped with Part I. The benefits of being able to adapt tools to local needs; provide training and guidance; use multi‐disciplinary teams; and build positive relationships with service users were identified across both parts of the review, providing support for their relevance across the wider field of violence prevention and counter‐radicalisation work.

Similarly, evidence relating to barriers including an uncertainty about the utility and validity of tools; time and resources required to complete risk assessments; subjectivity in how risk was assessed by different professionals; and concerns about the neglect of protective factors was identified in both parts of the review.

#### 5.3.2 Overall completeness and applicability of evidence

The reviews identified through the literature searches enabled us to respond to all of the research objectives for Part II. However, the amount and quality of evidence identified varied across the research questions. In total, five reviews presented evidence relating to Objective 3 on the effectiveness of case management tools and approaches. Two reviews examined the effectiveness of different interventions, which limits the conclusions that can be drawn about the effectiveness of case management more broadly; particularly as neither of the interventions were specifically defined as ‘case management’ in the reviews. It was also not possible to examine the effectiveness of different approaches to case management as outlined in the protocol (Lewis et al., 2023) due to the limited number of reviews identified, and the lack of detail provided about their underlying assumptions.

Three reviews allowed us to draw some conclusions about the potential effectiveness of risk assessment tools and the polygraph in contributing to violence reduction. However, because any relationship between the use of such tools and intervention outcomes is likely to be indirect, it was not possible to comment on how these tools might contribute to a reduction in violence. No eligible reviews examined the effectiveness of other case management tools identified in Part I, which limits the conclusions that can be drawn about the effectiveness of relevant tools.

Whilst the number of reviews that examined their implementation remained small, it was possible to explore whether risk assessment tools were being delivered in the ways originally outlined in Section Objectives, 2 (Objective 4a) using data from two studies. However, no relevant evidence for other case management tools was identified. Moreover, all but one of the six reviews that pointed to relevant implementation factors and moderators affecting the use of case management tools (Objective 4b) focused on risk assessment tools and their use in informing risk management. The evidence relating to implementation was therefore heavily weighted towards one case management tool, and one stage of the case management process.

The reviews in Part II were international in nature, and included studies conducted in ten countries. Similarly to Part I, the vast majority of the evidence was drawn from the Global North, which limits the representativeness of our findings. Whilst we searched for relevant research in languages other than English, no eligible reviews were identified in French, Russian, German, Norwegian, Danish, or Swedish. The included reviews spanned secondary and tertiary prevention work delivered in a range of settings (i.e., clinical, community, and correctional), and included research relating to violent and sexual offending. Taken together, Part II captured evidence relating to a diverse range of populations and problems, albeit with some limitations.

Part II attempted to cover a huge body of research that was not restricted to any one type of violence, or any one field of violence prevention. Whilst our search strategy was comprehensive and systematic, there are inevitably challenges in trying to identify research from across multiple fields that may use different terminology to describe relevant tools and approaches. This is particularly the case given that Part II of the review was designed to identify transferable lessons for counter‐radicalisation work. As a result, the search terms developed to search this wider literature were based on those search terms used for Part I, which was informed by the literature on countering radicalisation to violence. Whilst we took steps to minimise any potential challenges created by this approach – such as piloting our search terms and benefiting from the input of an information retrieval specialist with experience in conducting Campbell systematic reviews relating to violence prevention (EE) – it is important to recognise this issue.

#### 5.3.3 Quality of the evidence

Only two of the reviews were assessed as being high quality using the AMSTAR II tool. Every review had at least one methodological weakness, and only one review included a meta‐analysis of relevant outcomes. In addition, the methodological inclusion criteria used in most of the reviews was less stringent than for Campbell systematic reviews; only two set strict restrictions on the types of quantitative designs that could be included. However, the original methodology as outlined in the protocol (Lewis et al., 2023) did not set any restrictions on the types of studies that could be included in Part II, and every review was assessed as being of sufficient quality to be included using the AMSTAR II tool. Moreover, as noted in Section 4.3.3, data drawn from weaker quantitative and qualitative studies (as cited in included reviews) provided relevant insights relating to implementation.

#### 5.3.4 Limitations and potential biases in the evidence

As discussed in Section 4.3.4 in relation to Part I, the inclusion of multiple languages, and the use of search terms that related to different stages of the case management process aimed to reduce the chances of missing relevant evidence. In addition, feedback from the Campbell Crime and Justice editorial board on our original search terms led us to include a larger set of search terms in the ‘problem’ domain of our search strategy for Part II (see Supporting Information: Appendix I) at the protocol stage so as to capture a broader range of synonyms for different types of violence. However, although some members of the team (particularly AC ad EE), and the advisory board also had experience in conducting research on topics relating to Part II, the research team had specific expertise in countering radicalisation to violence, and therefore had a stronger understanding of that literature than the wider field of violence prevention.

Whilst we used an appropriate tool for assessing the quality of included reviews (AMSTAR II), such assessments are subject to potential bias. The use of double coding, and in some instances detailed discussions between team members on individual reviews, helped to mitigate this issue. However, as noted in Section 4.3.4, it is possible that other research teams may have reached different conclusions as to what to include and exclude based on quality. Furthermore, the decision not to exclude studies based on the quality of the studies that they cited potentially introduced an increased risk of bias into our analysis of Part II. To mitigate this, as far as possible, we considered the potential biases that might have impacted the analysis presented in each review and reported on this where relevant.

No eligible reviews focused specifically on ‘case management’ interventions. Instead, the reviews included in Part II used a range of different terms to describe tools and approaches that the research team assessed as being relevant to this review. This may have led to some bias in our inclusion or exclusion decisions, as this process relied on the research team assessing whether the tools and approaches adhered to the core assumptions of our conceptual framework outlined in Section Objectives, 2. Whilst we used double, and sometimes triple coding to minimise this risk, the identification of case management tools and approaches remains subject to bias. Although we are confident in the methods used to screen studies, we recognise that other research teams may have made different inclusion and exclusion decisions.

A related challenge is that a number of systematic reviews which examined studies relating to relevant case management interventions were identified, but were not included in the review on the basis that they only presented outcome data at an aggregate level, or did not conduct sub‐group analysis of case management specifically. Whilst these studies would be captured by a systematic review of primary research studies, they are not covered here.

#### 5.3.5 Agreements and disagreements with other reviews

Due to the broad focus of Part II, no comparable overviews of reviews were identified.

## 6 AUTHORS' CONCLUSIONS

Very little robust evidence exists regarding the effectiveness of tools and approaches used in case management interventions designed to counter radicalisation to violence. Although research is better developed in the wider field of non‐terrorism related violence prevention, research regarding effectiveness is still limited.

Research has begun to develop a body of findings about factors which act to facilitate or create barriers to implementing case management interventions. The quality of this research is not strong, and much of the evidence is subject to significant risk of bias. However, research on non‐terrorism related violence prevention appears to be transferable to counter‐radicalisation. This offers promise that more systematic comparative work across these fields will be able to identify responses to the challenges facing research and practice in counter‐radicalisation work.

The findings set out in this review provide a platform for further research and practice. The review has identified important gaps in the literature and has laid out a nascent but growing body of work on processes that seem to carry the potential to support and undermine counter‐radicalisation interventions. It has demonstrated the benefits of the case management framework to organise research on the wide array of tools, approaches, actors and systems involved in this work. It has also illustrated the insights that can be derived from analysing the processes by which interventions are delivered, rather than the outcome of specific types of intervention such as ideological guidance or mentoring which, although still limited in scope, has been a more concerted area of research in this field over recent years.

### 6.1 Implications for policy and practice

Interventions explicitly informed by case management frameworks remain uncommon in counter‐radicalisation work, and there is insufficient evidence to say whether the tools and approaches currently in use are effective. This points to the need for ensuring that monitoring and evaluation processes are built into programme design. Notwithstanding the lack of robust evaluations, the case management framework provides a useful way of consolidating research and practice in an area that is only just beginning to develop more systematic approaches to structuring and quality assuring counter‐radicalisation interventions (Koehler, 2017).

Organising the evidence base in this way helps to identify areas of practice that warrant greater attention. The research suggests that policymakers and practitioners should place more explicit focus on the case planning and evaluation stages of the case management process when designing and delivering interventions, and consider which tools might be best utilised to supporting these stages of the process. It will also be important to consider whether and how the different stages of the case management process intersect, and the extent to which the process as a whole is operating as expected.

Although unable to speak to questions of effectiveness, this review did identify a body of research on what seems to facilitate or create barriers to the implementation of counter‐radicalisation interventions with insights for policy and practice. The quality of this research is not yet robust, however the evidence in this review points to three clusters of factors that offer preliminary insights into emerging good practice covering the role of systems and structures; relational processes; and staff training and support.

The importance of well‐conceived systems and structures able to support interventions was a feature of research on most aspects of the case management process. This highlighted the need for adequate resources and financing to enable sustainable programme development and delivery. An awareness of the barriers multi‐agency working arrangements may face and what supports them was also important. The research pointed to the benefits of developing effective communication processes; protocols and secure data transfer systems to support information sharing; a clear mandate and shared understanding of goals; and effective administrative processes that reduce the bureaucratic burden as far as possible.

Further insights from the review pointed to the need to identify more structured ways of reducing subjectivity and bias across different stages of the case management process. For example, during risk assessments through developing clearer definitions and means of identifying risk and protective factors, and at the monitoring and evaluation stage, through applying clearly conceptualised measures of change, as well as addressing issues that might lead to inconsistent use of risk assessment, and monitoring and evaluation tools. However, there was some evidence that some practitioners perceived benefits from being able to draw upon clinical judgements less reliant on structured risk assessment tools. Finally, developing more systematic ways of ensuring different stages of the case management process inform one another seems important, so that assessment processes support case planning and delivery.

As well as refining the systems and structures that enable case management interventions, the review pointed to the relational processes that seem to help facilitate or generate barriers to implementation. Multi‐agency arrangements were an important site for these relational dynamics. The importance of opportunities to develop trust between representatives from different agencies, and between statutory and external actors who might be involved in delivering interventions were identified as relevant. As were the benefits of reciprocal relationships that develop over time, and of being alert to potentially counter‐productive power differentials and hierarchies.

Related to this, providing opportunities to address the tensions that can emerge when different organisational cultures and priorities collide, for example when rehabilitative and public protection goals come into conflict, may help reduce barriers to inter‐agency working and support better outcomes. The relationships between clients and those delivering interventions were also highlighted as important, which suggests that intervention designers would benefit from more explicitly focusing on opportunities to nurture pro‐social relationships able to support the change process, and of identifying certain moments – such as the period at the end of an intervention – where this relationship might come under strain.

The third theme that emerged from the review related to staff support and training. The benefits of having a body of knowledgeable and experienced staff were emphasised repeatedly. This draws attention to the need for effective training programmes able to support the delivery of case management tools such as risk assessment, or monitoring and evaluation instruments, but which is also able to provide the skills needed to work effectively with clients, and with an awareness of the different types of knowledge and perspectives multi‐agency partners bring. Training may also be a route to addressing some of the biases that were identified in the research, including confirmation bias and unconscious bias that might shape decision making in ways which unfairly disadvantage certain identity groups.

As well as formal training, the provision of ongoing support and supervision for practitioners to help manage the demands of this work is important. A variety of types of support were identified that might be more or less appropriate depending on the context and the individual's role. Some of these included peer support; working in pairs; formal debriefing sessions; engaging with psychologists; and formal supervision. As well as supporting practitioner well‐being, this may provide ways of navigating the tensions practitioners face when working in a context characterised by high levels of public and political scrutiny.

A broader implication for policy and practice relates to the need to account for differing levels of resources, expertise and risk. Much of the research discussed in this report is rooted in the Global North. Conflict affected contexts, and those characterised by lower levels of CVE‐relevant infrastructure may attract lower levels of investment in case management interventions and limit opportunities for the kinds of research that might help understand how contextual factors shape implementation dynamics. Those responsible for enabling programmes in these contexts would benefit from recognising that having robust policies and related evidence requires investment in counter‐radicalisation interventions; case management structures and processes; and in research to understand the process and impact of these programmes.

### 6.2 Implications for research

The review points to some exciting avenues for future research. The most obvious of these is the need to conduct more rigorous evaluations. Although there are significant challenges to evaluating both the process and outcome of interventions seeking to counter radicalisation to violence (Lewis et al., 2020), there is an urgent need to understand the impact of different aspects of the case management process. Part of this involves addressing conceptual challenges associated with understanding the mechanisms by which interventions deliver their effects (Thompson & Leroux, 2022). Many of the tools and approaches described in the review are unlikely to directly contribute to a reduction in violence. Instead, their effects are likely to be indirect, mediated by the ways in which tools are implemented and the mechanisms through which they influence case management outcomes. As Mazerolle et al. (2021) argue, it is therefore important to theorise, identify and analyse these mechanisms to understand how and under what conditions they work.

The use of the case management framework has illustrated the unevenness of research across the case management process, and in turn identified a number of evidence gaps. Most attention has been paid to delivery and implementation, followed by client assessment, with an emphasis on risk assessment processes, whilst a reasonable amount of work has examined monitoring and evaluation tools. However, comparatively little attention has been paid to the first and last stages of the case management process. Although there is a modest but growing body of work on the processes by which members of the public and frontline non‐security related practitioners might identify and refer individuals they believe to be at risk into interventions (e.g., Thomas et al., 2020), relatively little research has been carried out on the means by which counter‐radicalisation practitioners make initial assessment regarding the potential eligibility of clients. Similarly, very little attention has focused on the exit and transition process. Both of these represent important areas for future work, not least as the process of exiting interventions has been identified as a period of particular vulnerability for clients (Marsden, 2017).

A wide range of potential facilitators and barriers to implementing case management interventions were identified in the review. There is now a need to understand more about the impact of these factors. Both to understand whether and how they influence intervention outcomes, and to learn if some are more important than others in supporting case management work. There is also some interesting work to be done to understand which factors are more important for different kinds of interventions, in what contexts. Comparative, systematic research able to map and test different facilitators and barriers will therefore be beneficial.

As well as looking at individual stages of the case management process, there is a need to develop a more holistic understanding of these types of intervention. First, to better understand how working practices across case management structures relate to one another, and how they might be better integrated, and second to understand the nature and impact of the different approaches, or theories of change, which inform interventions. To this end, the case management framework outlined in this review could be used by researchers to inform more comprehensive, and holistic evaluations of interventions by providing a foundation for understanding how the full case management process might be expected to unfold in practice, and how the implementation of this process might best be evaluated; the different stages of case management that require evaluation; and the different types of data relevant to each stage.

For example, findings from this review suggest that case planning and risk assessment does not always inform delivery and implementation, and that interventions do not always align with their underlying theory of change. Understanding what impact these inconsistencies have and learning how to develop more integrated approaches to case management working are therefore important areas for future research. To inform this, more work on the characteristics and outcomes of interventions informed by different types of logic model or theories of change would be beneficial. The review found that most interventions reflect a hybrid approach which combines aspects of risk‐oriented and strengths‐based perspectives. Understanding more about how these relate to one another – particularly given the review's finding that rehabilitative aims can sometimes be in tension with heavily risk‐oriented approaches to delivery – and how outcomes might be impacted by different underlying approaches, has the potential to inform important practical and conceptual developments.

Finally, although the assessment of transferability between research on countering radicalisation to violence and non‐terrorism related violence was limited by the quality and quantity of the research, there is some promise that more fine‐grained comparative analysis might yield insights for counter‐radicalisation work. Whilst Part II of this review was limited to examining systematic reviews, it provided empirical evidence of the potential synergies that might exist between criminological research and research on radicalisation that have long been touted by researchers writing from more theoretical perspectives (e.g., LaFree & Miller, 2008). To interrogate these processes more carefully, research that looked at primary studies across different stages of the case management process in non‐terrorism related violence prevention work will be helpful. This may include research on how to overcome the barriers to implementation described in this review, and to understand whether there are additional facilitators that could be relevant, as well as identifying additional tools, approaches, or interventions that might be relevant to efforts to counter radicalisation to violence.

## INFORMATION ABOUT THIS REVIEW

**Roles and responsibilities**

- Content: James Lewis, Sarah Marsden, Adrian Cherney, Martine, Zeuthen, Lotta Rahlf, Chloe Squires.
- Systematic review methods: James Lewis, Sarah Marsden, Adrian Cherney, Martine Zeuthen.
- Data analysis: James Lewis, Sarah Marsden, Adrian Cherney, Martine Zeuthen, Lotta Rahlf, Anne Peterscheck, Chloe Squires.
- Information retrieval: James Lewis, Sarah Marsden, Martine Zeuthen, Lotta Rahlf, Anne Peterscheck, Chloe Squires.

**Sources of support**

This review was supported by a Campbell collaboration grant awarded to Sarah Marsden via Public Safety Canada.

We would like to acknowledge the support of Elizabeth Eggins in designing and conducting the searches of the core academic research platforms, and of Farangiz Atamuradova and Julien Domergue in supporting the searching of literature in languages other than English.

## DECLARATIONS OF INTEREST

Adrian Cherney has authored or co‐authored several studies on case management interventions seeking to counter radicalisation to violence. However, he was not involved in the search and screening process.

## PLANS FOR UPDATING THE REVIEW

Sarah Marsden and James Lewis will be responsible for updating the review, which is anticipated to occur to in 5 years.

## Supporting information

Supporting information.

## ENDNOTES

1 Covidence is an online systematic review platform used for screening studies and for extracting data.

2 The term ‘weaker’ is used here to refer to research designs that are traditionally considered too weak to be able to infer evidence of intervention effectiveness (i.e., those designs that are not eligible for inclusion in a traditional Campbell systematic review). However, these designs are only considered weak in this context. That is, whilst they provide only weak evidence of intervention effectiveness, they can provide more robust evidence of implementation. Moreover, whilst they may be considered ‘weak’ within research fields with a more developed impact evaluation culture, they are considered more robust within other fields, including research on countering radicalisation to violence (e.g., Hassan, 2021a, 2021b). These designs are therefore not considered to be weak in relation to our research objectives.

3 These 13 factors were: Maintaining intensive and regular contact; Organising sufficient support within the family and close social circle; Cultivating theological reflection; Offering alternatives with regard to networks and participation; Enhancing self‐esteem; Bringing together different groups of people to combat stereotyping; Giving guidance to promote reflection and self‐examination with regard to the client's identity and decisions made in the past; Providing grief counselling and helping clients deal with uncertainty surrounding death; A structured approach in accordance with standard criteria to ensure a successful programme; Research into methods and exiting; Improving connection with society; Increasing resilience to discrimination; and Voluntary participation (AEF, 2018, p. 24).

4 The practice, and importance, of adapting and refining interventions in response to emerging lessons was similarly emphasised by Orban (2019) in research on prison mentoring in Norway.

5 The primary criteria were having a close peer or relative who has been recruited into a violent extremist group or who is engaged in violent extremist activity; being associated with violent criminals or gang members; holding radical or extremist views and tendencies; and being affiliated with holders of extremist views and tendencies. The secondary criteria were being a school dropout; having a dysfunctional family background; suddenly becoming socially withdrawn; being a former convict; being a new convert to a religion; and being idle (Fisher et al., 2020, p. 23).

6 Multiple individual records reporting on the same study are only counted once in each section of the analysis. The number of in‐text citations does not, therefore, always match the count of studies.

7 Whilst Vandaele et al. (2022b) also present some data relevant to client assessment, it is not discussed here as this particular record examines data related to client assessment as part of a broader analysis of how multi‐agency conferences operate in practice.

8 The journal article from van de Weert and Eijkman (2020) was one of five articles based on the same research project that were identified through the literature searches. Whilst all five articles were primarily focused on the identification of radicalisation in the community, and raised similar issues related to client assessment, this article was the only one examined in the final review as it was solely focused on local security professionals who played a specific role in CVE work. The other papers were excluded because they focused on broader samples (van de Weert, 2022), or on frontline professionals tasked with identifying indicators of radicalisation risk (van de Weert & Eijkman, 2019, 2021a, 2021b).

9 Similar facilitators and barriers of multi‐agency working were identified by Vandaele et al., (2022a, 2022b), although not always in reference to client assessment.

10 Similar issues with the LSI‐R are also identified by the Inspector of Custodial Services (2018).

11 A number of additional studies mentioned the subjective nature of client assessment (e.g., Raets, 2022). However, only studies analysing the practical implications of subjectivity are included here.

12 A number of studies also pointed to how a lack of knowledge or confidence around ideology and religion might inhibit client assessment, although these studies are not discussed here as their analysis was not specifically linked to implementation (e.g., Cherney, 2021; Schuurman & Bakker, 2016).

13 In addition, five studies pointed to the use of some form of intervention planning tool, although limited information about these tools and/or how they were used was provided (AEF, 2018; Disley et al., 2016; Harris‐Hogan, 2020; Vandaele et al., 2022a; van der Heide & Schuurman, 2018).

14 Information collected from family members can also support the client assessment and case planning (Costa et al., 2021) and monitoring and evaluation (Cherney et al., 2022) stages.

15 The use of formers as providers is noted in other studies (e.g., Costa et al., 2021; Weeks, 2018). The organisation Life After Hate (2023) has also developed guidance for employing formers in counter radicalisation work which outlines the key knowledge, skills, and attitudes required for this work.

16 The importance of continuity is also implicitly referenced by other studies when discussing how practitioners focus a lot of their effort and time on building relationships and trust with their clients (e.g., Cherney, 2022; Christensen, 2015; Marsden, 2015; Weeks, 2018), or emphasising the importance of regular contact between practitioners and clients (e.g., Hofinger & Schmidinger, 2017; Raets, 2022).

17 Although, some organisations have developed handbooks (BAMF, 2020; Fisher et al., 2020).

18 Whilst not specifically related to interventions, the Inspector of Custodial Services (2018) identified four barriers to the effective completion of case notes by practitioners engaging with inmates in New South Wales: not all staff would complete case notes, as this wasn't a requirement; information in case notes was perceived by staff as being unverified, and thus potentially unreliable; there was a lack of training in how to complete case notes, which had led to some case notes being completed in an ‘unprofessional’ way; and concerns that inmates would be shown what was written about them meant that some staff were discouraged from writing anything down (p. 38). Variations in how different multi‐agency structures used (or did not use) documentation is also noted by Vandaele et al. (2022a).

19 The ethical implications related to information sharing have been widely discussed in the Scandinavian context (e.g., Kessing & Andersen, 2019; Bråthen, 2021). Whilst these studies did not meet our inclusion criteria, they raise important ethical, legal, and privacy considerations around the sharing of information in the context of countering radicalisation to violence. For example, a study conducted by the Danish Institute for Human Rights (Kessing & Andersen, 2019) examined the legality of information sharing regarding prisoners in Denmark from a Human Rights Convention perspective around the right to privacy, religion and equal treatment.

20 A number of the broader lessons relating to the use of case conferences during the assessment and case planning stages are likely to also be relevant to this stage, but are not discussed in this section to avoid double counting.

21 Similar concerns have also been raised by practitioners interviewed for excluded studies, such as Johansen's (2018) research examining case work conducted by the Infohouse in Aarhus, Denmark.

22 Whilst the earlier evaluation of the Team TER intervention identified specific challenges related to multi‐agency working (Schuurman & Bakker, 2016), this study is not listed here as the specific issue identified in this evaluation appeared to have been resolved by the time of the second evaluation.

23 Practitioners interviewed by Eijkman and Roodnat (2017) spanned secondary and tertiary prevention.

24 Practitioners interviewed by Raets (2022) spanned secondary and tertiary prevention.

25 Former detainees interviewed by Weggemans and de Graaf (2017) in the Netherlands also expressed frustration at the strict conditions placed on them after their release (p. 82).

26 It was unclear whether the participants quoted by Eijkman and Roodnat (2017) in reference to this point worked in secondary or tertiary prevention. However, this issue appeared to be more relevant to the secondary space, as the authors spoke of the importance of avoiding ‘anticipatory justice’.

27 A separate point relating to the broader public discourse around terrorism identified in one eligible study (Schuhmacher, 2018), and reflected in broader research relating to countering radicalisation to violence (e.g., Lewis, 2021), is that increasing focus on terrorism within public discourse can lead to an increase in the number of referrals/requests made to case management interventions.

28 Similarly, clients of voluntary interventions may choose to terminate their engagement even after they have initially agreed to participate (Möller & Neuscheler, 2018).

29 Whilst not always stated, the majority of identified interventions were voluntary (e.g., AEF, 2018; Cherney, 2018, 2020, 2022; Christensen, 2015; Costa et al., 2021; Harris‐Hogan, 2020; Pettinger, 2020a, 2020b). However, a small number of tertiary interventions were mandated (e.g., Costa et al., 2021; van der Heide & Schuurman, 2018; Weeks, 2018). It is worth noting that a recent systematic review found limited empirical evidence relating to the effectiveness of mandated versus voluntary CVE interventions more broadly (Cherney et al., 2021).

30 This localised approach to identifying risk has proved controversial due to the potential stigmatisation this might cause for local communities who are identified as especially ‘risky’ (e.g., Abbas, 2019; Heath‐Kelly, 2013). Whilst none of the eligible studies discussed this issue in detail, it is important to recognise the potential unintended consequences that such an approach might produce at the local level.

31
https://www.campbellcollaboration.org/what-is-a-systematic-review.html

32 One review also identified three studies using illustrative case examples involving the use of structured risk formulation which reported successful outcomes for the individual (Tarpey, 2021). However, the quality scores for these studies were low (ranging from 31% to 35%), meaning that there was insufficient evidence that the risk formulation contributed to these outcomes.
