# Supplementary material for: Case management interventions seeking to counter radicalisation to violence and related forms of violence: A systematic review
Source: Campbell Syst Rev. 2024 Apr 12;20(2):e1386. doi: 10.1002/cl2.1386 (PMC11015087; doi:10.1002/cl2.1386)
Supplement: Supplementary file 1 — Supporting information. [file CL2-20-e1386-s001.docx]

**Appendix I (A) Part I Search Record (English Language)**

**Table A1.1(a) Academic Platform Search Record – Part I**

| **Search Source** | **Source** | **Search Date)** | **Date Coverage for Search** | **Results** |
| --- | --- | --- | --- | --- |
| Criminal Justice Abstracts | EBSCO | 07/08/2022 | 01/01/2000 - 07/08/2022 | 3829 |
| Scopus | Elsevier | 07/08/2022 | 01/01/2000 - 07/08/2022 | 15777 |
| CINCH: Australian Criminology Database | Informit | 07/08/2022 | 01/01/2000 - 07/08/2022 | 549 |
| Medline | Ovid | 07/08/2022 | 01/01/2000 - 07/08/2022 | 2922 |
| PsycInfo | Ovid | 07/08/2022 | 01/01/2000 - 07/08/2022 | 3168 |
| Dissertations and Theses Global | ProQuest | 07/08/2022 | 01/01/2000 - 07/08/2022 | 3618 |
| International Bibliography of the Social Sciences | ProQuest | 07/08/2022 | 01/01/2000 - 07/08/2022 | 7940 |
| Sociological Abstracts | ProQuest | 07/08/2022 | 01/01/2000 - 07/08/2022 | 3482 |
| Book Citation Index – Social Sciences & Humanities (BKCI-SSH)  Social Sciences Citation Index (SSCI)  Arts & Humanities Citation Index (A&HCI)  Emerging Sources Citation Index (ESCI)  Conference Proceedings Citation Index – Social Science & Humanities (CPCI-SSH) | Web of Science | 07/08/2022 | 01/01/2000 - 07/08/2022 | 8125 |

**Table A1.2(a). Criminal Justice Abstracts Search Record – Part I**

| **#** | **Query** | **Limiters/Expanders** | **Last Run Via** | **Results** |
| --- | --- | --- | --- | --- |
| S4 | S1 AND S2 AND S3 | Limiters - Publication Date: 20000101-20221231 | Interface - EBSCOhost Research Databases | 3,829 |
|  |  | Search modes - Boolean/Phrase | Search Screen - Advanced Search |  |
|  |  |  | Database - Criminal Justice Abstracts |  |
| S3 | TI ( prevent* OR reduc* OR counter* OR disengage* OR rehab* OR reintegrat* OR re-integrat* OR re-entry OR reentry OR desist* OR recidivism OR deradical* OR de-radical* OR exit* ) OR AB ( prevent* OR reduc* OR counter* OR disengage* OR rehab* OR reintegrat* OR re-integrat* OR re-entry OR reentry OR desist* OR recidivism OR deradical* OR de-radical* OR exit* ) OR KW ( prevent* OR reduc* OR counter* OR disengage* OR rehab* OR reintegrat* OR re-integrat* OR re-entry OR reentry OR desist* OR recidivism OR deradical* OR de-radical* OR exit* ) OR SU ( prevent* OR reduc* OR counter* OR disengage* OR rehab* OR reintegrat* OR re-integrat* OR re-entry OR reentry OR desist* OR recidivism OR deradical* OR de-radical* OR exit* ) | Limiters - Publication Date: 20000101-20221231 | Interface - EBSCOhost Research Databases | 111,265 |
|  |  | Search modes - Boolean/Phrase | Search Screen - Advanced Search |  |
|  |  |  | Database - Criminal Justice Abstracts |  |
| S2 | TI ( initiative* OR interven* OR program* OR policy OR policies OR scheme* OR treat* OR approach* OR model* OR strateg* OR method* OR project* OR practice* OR instrument* OR tool* OR framework* OR protocol* OR guid* OR scale* OR system* OR inventor* OR metric* OR template* OR profile* OR criteria OR questionnaire* OR refer* OR assess* OR "case plan*" OR "case formulat*" OR "management plan*" OR "treatment plan*" OR "support plan*" OR "case manage*" OR "risk manage*" OR progress* OR monitor* OR supervis* OR measur* ) OR AB ( initiative* OR interven* OR program* OR policy OR policies OR scheme* OR treat* OR approach* OR model* OR strateg* OR method* OR project* OR practice* OR instrument* OR tool* OR framework* OR protocol* OR guid* OR scale* OR system* OR inventor* OR metric* OR template* OR profile* OR criteria OR questionnaire* OR refer* OR assess* OR "case plan*" OR "case formulat*" OR "management plan*" OR "treatment plan*" OR "support plan*" OR "case manage*" OR "risk manage*" OR progress* OR monitor* OR supervis* OR measur* ) OR KW ( initiative* OR interven* OR program* OR policy OR policies OR scheme* OR treat* OR approach* OR model* OR strateg* OR method* OR project* OR practice* OR instrument* OR tool* OR framework* OR protocol* OR guid* OR scale* OR system* OR inventor* OR metric* OR template* OR profile* OR criteria OR questionnaire* OR refer* OR assess* OR "case plan*" OR "case formulat*" OR "management plan*" OR "treatment plan*" OR "support plan*" OR "case manage*" OR "risk manage*" OR progress* OR monitor* OR supervis* OR measur* ) OR SU ( initiative* OR interven* OR program* OR policy OR policies OR scheme* OR treat* OR approach* OR model* OR strateg* OR method* OR project* OR practice* OR instrument* OR tool* OR framework* OR protocol* OR guid* OR scale* OR system* OR inventor* OR metric* OR template* OR profile* OR criteria OR questionnaire* OR refer* OR assess* OR "case plan*" OR "case formulat*" OR "management plan*" OR "treatment plan*" OR "support plan*" OR "case manage*" OR "risk manage*" OR progress* OR monitor* OR supervis* OR measur* ) | Limiters - Publication Date: 20000101-20221231 | Interface - EBSCOhost Research Databases | 360,906 |
|  |  | Search modes - Boolean/Phrase | Search Screen - Advanced Search |  |
|  |  |  | Database - Criminal Justice Abstracts |  |
| S1 | TI ( radicali* OR extremis* OR terroris* OR jihadi* OR islamis* OR salafi* OR right-wing OR "right wing" OR extreme-right OR "extreme right" OR neo-nazi OR far-right OR "far right" OR Nationalist* OR "white supremacis*" OR left-wing OR "left wing" OR extreme-left OR "extreme left" OR anarch* OR "single issue" OR single-issue ) OR AB ( radicali* OR extremis* OR terroris* OR jihadi* OR islamis* OR salafi* OR right-wing OR "right wing" OR extreme-right OR "extreme right" OR neo-nazi OR far-right OR "far right" OR Nationalist* OR "white supremacis*" OR left-wing OR "left wing" OR extreme-left OR "extreme left" OR anarch* OR "single issue" OR single-issue ) OR KW ( radicali* OR extremis* OR terroris* OR jihadi* OR islamis* OR salafi* OR right-wing OR "right wing" OR extreme-right OR "extreme right" OR neo-nazi OR far-right OR "far right" OR Nationalist* OR "white supremacis*" OR left-wing OR "left wing" OR extreme-left OR "extreme left" OR anarch* OR "single issue" OR single-issue ) OR SU ( radicali* OR extremis* OR terroris* OR jihadi* OR islamis* OR salafi* OR right-wing OR "right wing" OR extreme-right OR "extreme right" OR neo-nazi OR far-right OR "far right" OR Nationalist* OR "white supremacis*" OR left-wing OR "left wing" OR extreme-left OR "extreme left" OR anarch* OR "single issue" OR single-issue ) | Limiters - Publication Date: 20000101-20221231 | Interface - EBSCOhost Research Databases | 14,412 |
|  |  | Search modes - Boolean/Phrase | Search Screen - Advanced Search |  |
|  |  |  | Database - Criminal Justice Abstracts |  |

**Table A1.3(a). Scopus Search Records – Part I**

| **Query** | **Records** |
| --- | --- |
| ( TITLE-ABS-KEY ( prevent* OR reduc* OR counter* OR disengage* OR rehab* OR reintegrat* OR re-integrat* OR re-entry OR reentry OR desist* OR recidivism OR deradical* OR de-radical* OR exit* ) ) AND ( TITLE-ABS-KEY ( initiative* OR interven* OR program* OR policy OR policies OR scheme* OR treat* OR approach* OR model* OR strateg* OR method* OR project* OR practice* OR instrument* OR tool* OR framework* OR protocol* OR guid* OR scale* OR system* OR inventor* OR metric* OR template* OR profile* OR criteria OR questionnaire* OR refer* OR assess* OR "case plan*"OR "case formulat*" OR "management plan*" OR "treatment plan*" OR "support plan*" OR "case manage*" OR "risk manage*" OR progress* ORmonitor* OR supervis* OR measur* ) ) AND ( TITLE-ABS-KEY ( radicali* OR extremis* OR terroris* OR jihadi* OR islamis* OR salafi* OR right-wingOR "right wing" OR extreme-right OR "extreme right" OR neo-nazi OR far-right OR "far right" OR nationalist* OR "white supremacis*" OR left-wing OR"left wing" OR extreme-left OR "extreme left" OR anarch* OR "single issue" OR single-issue ) ) AND ( LIMIT-TO ( PUBYEAR , 2004 ) OR LIMIT-TO (PUBYEAR , 2003 ) OR LIMIT-TO ( PUBYEAR , 2002 ) OR LIMIT-TO ( PUBYEAR , 2001 ) OR LIMIT-TO ( PUBYEAR , 2000 ) ) AND ( EXCLUDE ( SUBJAREA ,"engi" ) OR EXCLUDE ( SUBJAREA , "envi" ) OR EXCLUDE ( SUBJAREA , "phys" ) OR EXCLUDE ( SUBJAREA , "bioc" ) OR EXCLUDE ( SUBJAREA , "ceng" )OR EXCLUDE ( SUBJAREA , "eart" ) OR EXCLUDE ( SUBJAREA , "phar" ) OR EXCLUDE ( SUBJAREA , "ener" ) OR EXCLUDE ( SUBJAREA , "chem" ) OREXCLUDE ( SUBJAREA , "immu" ) ) AND ( EXCLUDE ( DOCTYPE , "no" ) OR EXCLUDE ( DOCTYPE , "ed" ) OR EXCLUDE ( DOCTYPE , "le" ) OREXCLUDE ( DOCTYPE , "cr" ) ) | 970 |
| ( TITLE-ABS-KEY ( prevent* OR reduc* OR counter* OR disengage* OR rehab* OR reintegrat* OR re-integrat* OR re-entry OR reentry OR desist*OR recidivism OR deradical* OR de-radical* OR exit* ) ) AND ( TITLE-ABS-KEY ( initiative* OR interven* OR program* OR policy OR policies ORscheme* OR treat* OR approach* OR model* OR strateg* OR method* OR project* OR practice* OR instrument* OR tool* OR framework* ORprotocol* OR guid* OR scale* OR system* OR inventor* OR metric* OR template* OR profile* OR criteria OR questionnaire* OR refer* OR assess*OR "case plan*" OR "case formulat*" OR "management plan*" OR "treatment plan*" OR "support plan*" OR "case manage*" OR "risk manage*" ORprogress* OR monitor* OR supervis* OR measur* ) ) AND ( TITLE-ABS-KEY ( radicali* OR extremis* OR terroris* OR jihadi* OR islamis* OR salafi*OR right-wing OR "right wing" OR extreme-right OR "extreme right" OR neo-nazi OR far-right OR "far right" OR nationalist* OR "white supremacis*"OR left-wing OR "left wing" OR extreme-left OR "extreme left" OR anarch* OR "single issue" OR single-issue ) ) AND ( LIMIT-TO ( PUBYEAR , 2004 )OR LIMIT-TO ( PUBYEAR , 2003 ) OR LIMIT-TO ( PUBYEAR , 2002 ) OR LIMIT-TO ( PUBYEAR , 2001 ) OR LIMIT-TO ( PUBYEAR , 2000 ) ) AND (EXCLUDE ( SUBJAREA , "engi" ) OR EXCLUDE ( SUBJAREA , "envi" ) OR EXCLUDE ( SUBJAREA , "phys" ) OR EXCLUDE ( SUBJAREA , "bioc" ) OREXCLUDE ( SUBJAREA , "ceng" ) OR EXCLUDE ( SUBJAREA , "eart" ) OR EXCLUDE ( SUBJAREA , "phar" ) OR EXCLUDE ( SUBJAREA , "ener" ) OREXCLUDE ( SUBJAREA , "chem" ) OR EXCLUDE ( SUBJAREA , "immu" ) ) | 1012 |
| ( TITLE-ABS-KEY ( prevent* OR reduc* OR counter* OR disengage* OR rehab* OR reintegrat* OR re-integrat* OR re-entry OR reentry OR desist*OR recidivism OR deradical* OR de-radical* OR exit* ) ) AND ( TITLE-ABS-KEY ( initiative* OR interven* OR program* OR policy OR policies ORscheme* OR treat* OR approach* OR model* OR strateg* OR method* OR project* OR practice* OR instrument* OR tool* OR framework* ORprotocol* OR guid* OR scale* OR system* OR inventor* OR metric* OR template* OR profile* OR criteria OR questionnaire* OR refer* OR assess*OR "case plan*" OR "case formulat*" OR "management plan*" OR "treatment plan*" OR "support plan*" OR "case manage*" OR "risk manage*" ORprogress* OR monitor* OR supervis* OR measur* ) ) AND ( TITLE-ABS-KEY ( radicali* OR extremis* OR terroris* OR jihadi* OR islamis* OR salafi*OR right-wing OR "right wing" OR extreme-right OR "extreme right" OR neo-nazi OR far-right OR "far right" OR nationalist* OR "white supremacis*"OR left-wing OR "left wing" OR extreme-left OR "extreme left" OR anarch* OR "single issue" OR single-issue ) ) AND ( LIMIT-TO ( PUBYEAR , 2004 )OR LIMIT-TO ( PUBYEAR , 2003 ) OR LIMIT-TO ( PUBYEAR , 2002 ) OR LIMIT-TO ( PUBYEAR , 2001 ) OR LIMIT-TO ( PUBYEAR , 2000 ) ) | 1752 |
| ( TITLE-ABS-KEY ( prevent* OR reduc* OR counter* OR disengage* OR rehab* OR reintegrat* OR re-integrat* OR re-entry OR reentry OR desist*OR recidivism OR deradical* OR de-radical* OR exit* ) ) AND ( TITLE-ABS-KEY ( initiative* OR interven* OR program* OR policy OR policies ORscheme* OR treat* OR approach* OR model* OR strateg* OR method* OR project* OR practice* OR instrument* OR tool* OR framework* ORprotocol* OR guid* OR scale* OR system* OR inventor* OR metric* OR template* OR profile* OR criteria OR questionnaire* OR refer* OR assess*OR "case plan*" OR "case formulat*" OR "management plan*" OR "treatment plan*" OR "support plan*" OR "case manage*" OR "risk manage*" ORprogress* OR monitor* OR supervis* OR measur* ) ) AND ( TITLE-ABS-KEY ( radicali* OR extremis* OR terroris* OR jihadi* OR islamis* OR salafi*OR right-wing OR "right wing" OR extreme-right OR "extreme right" OR neo-nazi OR far-right OR "far right" OR nationalist* OR "white supremacis*"OR left-wing OR "left wing" OR extreme-left OR "extreme left" OR anarch* OR "single issue" OR single-issue ) ) AND ( LIMIT-TO ( PUBYEAR , 2007 )OR LIMIT-TO ( PUBYEAR , 2006 ) OR LIMIT-TO ( PUBYEAR , 2005 ) ) AND ( EXCLUDE ( SUBJAREA , "engi" ) OR EXCLUDE ( SUBJAREA , "envi" ) OREXCLUDE ( SUBJAREA , "phys" ) OR EXCLUDE ( SUBJAREA , "mate" ) OR EXCLUDE ( SUBJAREA , "bioc" ) OR EXCLUDE ( SUBJAREA , "ceng" ) OREXCLUDE ( SUBJAREA , "ener" ) OR EXCLUDE ( SUBJAREA , "eart" ) OR EXCLUDE ( SUBJAREA , "chem" ) OR EXCLUDE ( SUBJAREA , "phar" ) OREXCLUDE ( SUBJAREA , "immu" ) ) AND ( EXCLUDE ( DOCTYPE , "ed" ) OR EXCLUDE ( DOCTYPE , "no" ) OR EXCLUDE ( DOCTYPE , "cr" ) OREXCLUDE ( DOCTYPE , "le" ) ) | 1427 |
| ( TITLE-ABS-KEY ( prevent* OR reduc* OR counter* OR disengage* OR rehab* OR reintegrat* OR re-integrat* OR re-entry OR reentry OR desist* ORrecidivism OR deradical* OR de-radical* OR exit* ) ) AND ( TITLE-ABS-KEY ( initiative* OR interven* OR program* OR policy OR policies OR scheme*OR treat* OR approach* OR model* OR strateg* OR method* OR project* OR practice* OR instrument* OR tool* OR framework* OR protocol* ORguid* OR scale* OR system* OR inventor* OR metric* OR template* OR profile* OR criteria OR questionnaire* OR refer* OR assess* OR "case plan*" OR "case formulat*" OR "management plan*" OR "treatment plan*" OR "support plan*" OR "case manage*" OR "risk manage*" OR progress*OR monitor* OR supervis* OR measur* ) ) AND ( TITLE-ABS-KEY ( radicali* OR extremis* OR terroris* OR jihadi* OR islamis* OR salafi* OR right-wing OR "right wing" OR extreme-right OR "extreme right" OR neo-nazi OR far-right OR "far right" OR nationalist* OR "white supremacis*" OR left-wing OR "left wing" OR extreme-left OR "extreme left" OR anarch* OR "single issue" OR single-issue ) ) AND ( LIMIT-TO ( PUBYEAR , 2007 ) OR LIMIT-TO ( PUBYEAR , 2006 ) OR LIMIT-TO ( PUBYEAR , 2005 ) ) AND ( EXCLUDE ( SUBJAREA , "engi" ) OR EXCLUDE ( SUBJAREA , "envi" ) OR EXCLUDE (SUBJAREA , "phys" ) OR EXCLUDE ( SUBJAREA , "mate" ) OR EXCLUDE ( SUBJAREA , "bioc" ) OR EXCLUDE ( SUBJAREA , "ceng" ) OR EXCLUDE (SUBJAREA , "ener" ) OR EXCLUDE ( SUBJAREA , "eart" ) OR EXCLUDE ( SUBJAREA , "chem" ) OR EXCLUDE ( SUBJAREA , "phar" ) OR EXCLUDE (SUBJAREA , "immu" ) ) | 1448 |
| ( TITLE-ABS-KEY ( prevent* OR reduc* OR counter* OR disengage* OR rehab* OR reintegrat* OR re-integrat* OR re-entry OR reentry OR desist*OR recidivism OR deradical* OR de-radical* OR exit* ) ) AND ( TITLE-ABS-KEY ( initiative* OR interven* OR program* OR policy OR policies ORscheme* OR treat* OR approach* OR model* OR strateg* OR method* OR project* OR practice* OR instrument* OR tool* OR framework* ORprotocol* OR guid* OR scale* OR system* OR inventor* OR metric* OR template* OR profile* OR criteria OR questionnaire* OR refer* OR assess*OR "case plan*" OR "case formulat*" OR "management plan*" OR "treatment plan*" OR "support plan*" OR "case manage*" OR "risk manage*" ORprogress* OR monitor* OR supervis* OR measur* ) ) AND ( TITLE-ABS-KEY ( radicali* OR extremis* OR terroris* OR jihadi* OR islamis* OR salafi*OR right-wing OR "right wing" OR extreme-right OR "extreme right" OR neo-nazi OR far-right OR "far right" OR nationalist* OR "white supremacis*"OR left-wing OR "left wing" OR extreme-left OR "extreme left" OR anarch* OR "single issue" OR single-issue ) ) AND ( LIMIT-TO ( PUBYEAR , 2007 )OR LIMIT-TO ( PUBYEAR , 2006 ) OR LIMIT-TO ( PUBYEAR , 2005 ) ) | 2428 |
| ( TITLE-ABS-KEY ( prevent* OR reduc* OR counter* OR disengage* OR rehab* OR reintegrat* OR re-integrat* OR re-entry OR reentry OR desist*OR recidivism OR deradical* OR de-radical* OR exit* ) ) AND ( TITLE-ABS-KEY ( initiative* OR interven* OR program* OR policy OR policies ORscheme* OR treat* OR approach* OR model* OR strateg* OR method* OR project* OR practice* OR instrument* OR tool* OR framework* ORprotocol* OR guid* OR scale* OR system* OR inventor* OR metric* OR template* OR profile* OR criteria OR questionnaire* OR refer* OR assess*OR "case plan*" OR "case formulat*" OR "management plan*" OR "treatment plan*" OR "support plan*" OR "case manage*" OR "risk manage*" ORprogress* OR monitor* OR supervis* OR measur* ) ) AND ( TITLE-ABS-KEY ( radicali* OR extremis* OR terroris* OR jihadi* OR islamis* OR salafi*OR right-wing OR "right wing" OR extreme-right OR "extreme right" OR neo-nazi OR far-right OR "far right" OR nationalist* OR "white supremacis*"OR left-wing OR "left wing" OR extreme-left OR "extreme left" OR anarch* OR "single issue" OR single-issue ) ) AND ( LIMIT-TO ( PUBYEAR , 2010 )OR LIMIT-TO ( PUBYEAR , 2009 ) OR LIMIT-TO ( PUBYEAR , 2008 ) ) AND ( EXCLUDE ( SUBJAREA , "engi" ) OR EXCLUDE ( SUBJAREA , "envi" ) OREXCLUDE ( SUBJAREA , "phys" ) OR EXCLUDE ( SUBJAREA , "bioc" ) OR EXCLUDE ( SUBJAREA , "mate" ) OR EXCLUDE ( SUBJAREA , "phar" ) OREXCLUDE ( SUBJAREA , "eart" ) OR EXCLUDE ( SUBJAREA , "ener" ) OR EXCLUDE ( SUBJAREA , "chem" ) OR EXCLUDE ( SUBJAREA , "ceng" ) OREXCLUDE ( SUBJAREA , "immu" ) ) AND ( EXCLUDE ( DOCTYPE , "ed" ) OR EXCLUDE ( DOCTYPE , "no" ) OR EXCLUDE ( DOCTYPE , "cr" ) ) | 1925 |
| ( TITLE-ABS-KEY ( prevent* OR reduc* OR counter* OR disengage* OR rehab* OR reintegrat* OR re-integrat* OR re-entry OR reentry OR desist*OR recidivism OR deradical* OR de-radical* OR exit* ) ) AND ( TITLE-ABS-KEY ( initiative* OR interven* OR program* OR policy OR policies ORscheme* OR treat* OR approach* OR model* OR strateg* OR method* OR project* OR practice* OR instrument* OR tool* OR framework* ORprotocol* OR guid* OR scale* OR system* OR inventor* OR metric* OR template* OR profile* OR criteria OR questionnaire* OR refer* OR assess*OR "case plan*" OR "case formulat*" OR "management plan*" OR "treatment plan*" OR "support plan*" OR "case manage*" OR "risk manage*" ORprogress* OR monitor* OR supervis* OR measur* ) ) AND ( TITLE-ABS-KEY ( radicali* OR extremis* OR terroris* OR jihadi* OR islamis* OR salafi*OR right-wing OR "right wing" OR extreme-right OR "extreme right" OR neo-nazi OR far-right OR "far right" OR nationalist* OR "white supremacis*"OR left-wing OR "left wing" OR extreme-left OR "extreme left" OR anarch* OR "single issue" OR single-issue ) ) AND ( LIMIT-TO ( PUBYEAR , 2010 )OR LIMIT-TO ( PUBYEAR , 2009 ) OR LIMIT-TO ( PUBYEAR , 2008 ) ) AND ( EXCLUDE ( SUBJAREA , "engi" ) OR EXCLUDE ( SUBJAREA , "envi" ) OREXCLUDE ( SUBJAREA , "phys" ) OR EXCLUDE ( SUBJAREA , "bioc" ) OR EXCLUDE ( SUBJAREA , "mate" ) OR EXCLUDE ( SUBJAREA , "phar" ) OREXCLUDE ( SUBJAREA , "eart" ) OR EXCLUDE ( SUBJAREA , "ener" ) OR EXCLUDE ( SUBJAREA , "chem" ) OR EXCLUDE ( SUBJAREA , "ceng" ) OREXCLUDE ( SUBJAREA , "immu" ) ) | 1963 |
| ( TITLE-ABS-KEY ( prevent* OR reduc* OR counter* OR disengage* OR rehab* OR reintegrat* OR re-integrat* OR re-entry OR reentry OR desist*OR recidivism OR deradical* OR de-radical* OR exit* ) ) AND ( TITLE-ABS-KEY ( initiative* OR interven* OR program* OR policy OR policies ORscheme* OR treat* OR approach* OR model* OR strateg* OR method* OR project* OR practice* OR instrument* OR tool* OR framework* ORprotocol* OR guid* OR scale* OR system* OR inventor* OR metric* OR template* OR profile* OR criteria OR questionnaire* OR refer* OR assess*OR "case plan*" OR "case formulat*" OR "management plan*" OR "treatment plan*" OR "support plan*" OR "case manage*" OR "risk manage*" ORprogress* OR monitor* OR supervis* OR measur* ) ) AND ( TITLE-ABS-KEY ( radicali* OR extremis* OR terroris* OR jihadi* OR islamis* OR salafi*OR right-wing OR "right wing" OR extreme-right OR "extreme right" OR neo-nazi OR far-right OR "far right" OR nationalist* OR "white supremacis*"OR left-wing OR "left wing" OR extreme-left OR "extreme left" OR anarch* OR "single issue" OR single-issue ) ) AND ( LIMIT-TO ( PUBYEAR , 2010 )OR LIMIT-TO ( PUBYEAR , 2009 ) OR LIMIT-TO ( PUBYEAR , 2008 ) ) | 2653 |
| ( TITLE-ABS-KEY ( prevent* OR reduc* OR counter* OR disengage* OR rehab* OR reintegrat* OR re-integrat* OR re-entry OR reentry OR desist*OR recidivism OR deradical* OR de-radical* OR exit* ) ) AND ( TITLE-ABS-KEY ( initiative* OR interven* OR program* OR policy OR policies ORscheme* OR treat* OR approach* OR model* OR strateg* OR method* OR project* OR practice* OR instrument* OR tool* OR framework* ORprotocol* OR guid* OR scale* OR system* OR inventor* OR metric* OR template* OR profile* OR criteria OR questionnaire* OR refer* OR assess*OR "case plan*" OR "case formulat*" OR "management plan*" OR "treatment plan*" OR "support plan*" OR "case manage*" OR "risk manage*" ORprogress* OR monitor* OR supervis* OR measur* ) ) AND ( TITLE-ABS-KEY ( radicali* OR extremis* OR terroris* OR jihadi* OR islamis* OR salafi*OR right-wing OR "right wing" OR extreme-right OR "extreme right" OR neo-nazi OR far-right OR "far right" OR nationalist* OR "white supremacis*"OR left-wing OR "left wing" OR extreme-left OR "extreme left" OR anarch* OR "single issue" OR single-issue ) ) AND ( LIMIT-TO ( PUBYEAR , 2012 )OR LIMIT-TO ( PUBYEAR , 2011 ) ) AND ( EXCLUDE ( SUBJAREA , "engi" ) OR EXCLUDE ( SUBJAREA , "envi" ) OR EXCLUDE ( SUBJAREA , "phys" ) OREXCLUDE ( SUBJAREA , "bioc" ) OR EXCLUDE ( SUBJAREA , "eart" ) OR EXCLUDE ( SUBJAREA , "mate" ) OR EXCLUDE ( SUBJAREA , "ener" ) OREXCLUDE ( SUBJAREA , "phar" ) OR EXCLUDE ( SUBJAREA , "chem" ) OR EXCLUDE ( SUBJAREA , "ceng" ) OR EXCLUDE ( SUBJAREA , "immu" ) ) AND( EXCLUDE ( DOCTYPE , "ed" ) OR EXCLUDE ( DOCTYPE , "no" ) OR EXCLUDE ( DOCTYPE , "cr" ) OR EXCLUDE ( DOCTYPE , "le" ) ) | 1706 |
| ( TITLE-ABS-KEY ( prevent* OR reduc* OR counter* OR disengage* OR rehab* OR reintegrat* OR re-integrat* OR re-entry OR reentry OR desist*OR recidivism OR deradical* OR de-radical* OR exit* ) ) AND ( TITLE-ABS-KEY ( initiative* OR interven* OR program* OR policy OR policies ORscheme* OR treat* OR approach* OR model* OR strateg* OR method* OR project* OR practice* OR instrument* OR tool* OR framework* ORprotocol* OR guid* OR scale* OR system* OR inventor* OR metric* OR template* OR profile* OR criteria OR questionnaire* OR refer* OR assess*OR "case plan*" OR "case formulat*" OR "management plan*" OR "treatment plan*" OR "support plan*" OR "case manage*" OR "risk manage*" ORprogress* OR monitor* OR supervis* OR measur* ) ) AND ( TITLE-ABS-KEY ( radicali* OR extremis* OR terroris* OR jihadi* OR islamis* OR salafi*OR right-wing OR "right wing" OR extreme-right OR "extreme right" OR neo-nazi OR far-right OR "far right" OR nationalist* OR "white supremacis*"OR left-wing OR "left wing" OR extreme-left OR "extreme left" OR anarch* OR "single issue" OR single-issue ) ) AND ( LIMIT-TO ( PUBYEAR , 2012 )OR LIMIT-TO ( PUBYEAR , 2011 ) ) AND ( EXCLUDE ( SUBJAREA , "engi" ) OR EXCLUDE ( SUBJAREA , "envi" ) OR EXCLUDE ( SUBJAREA , "phys" ) OREXCLUDE ( SUBJAREA , "bioc" ) OR EXCLUDE ( SUBJAREA , "eart" ) OR EXCLUDE ( SUBJAREA , "mate" ) OR EXCLUDE ( SUBJAREA , "ener" ) OREXCLUDE ( SUBJAREA , "phar" ) OR EXCLUDE ( SUBJAREA , "chem" ) OR EXCLUDE ( SUBJAREA , "ceng" ) OR EXCLUDE ( SUBJAREA , "immu" ) ) | 1728 |
| ( TITLE-ABS-KEY ( prevent* OR reduc* OR counter* OR disengage* OR rehab* OR reintegrat* OR re-integrat* OR re-entry OR reentry OR desist*OR recidivism OR deradical* OR de-radical* OR exit* ) ) AND ( TITLE-ABS-KEY ( initiative* OR interven* OR program* OR policy OR policies ORscheme* OR treat* OR approach* OR model* OR strateg* OR method* OR project* OR practice* OR instrument* OR tool* OR framework* ORprotocol* OR guid* OR scale* OR system* OR inventor* OR metric* OR template* OR profile* OR criteria OR questionnaire* OR refer* OR assess*OR "case plan*" OR "case formulat*" OR "management plan*" OR "treatment plan*" OR "support plan*" OR "case manage*" OR "risk manage*" ORprogress* OR monitor* OR supervis* OR measur* ) ) AND ( TITLE-ABS-KEY ( radicali* OR extremis* OR terroris* OR jihadi* OR islamis* OR salafi*OR right-wing OR "right wing" OR extreme-right OR "extreme right" OR neo-nazi OR far-right OR "far right" OR nationalist* OR "white supremacis*"OR left-wing OR "left wing" OR extreme-left OR "extreme left" OR anarch* OR "single issue" OR single-issue ) ) AND ( LIMIT-TO ( PUBYEAR , 2012 )OR LIMIT-TO ( PUBYEAR , 2011 ) ) | 2198 |
| ( TITLE-ABS-KEY ( prevent* OR reduc* OR counter* OR disengage* OR rehab* OR reintegrat* OR re-integrat* OR re-entry OR reentry OR desist*OR recidivism OR deradical* OR de-radical* OR exit* ) ) AND ( TITLE-ABS-KEY ( initiative* OR interven* OR program* OR policy OR policies ORscheme* OR treat* OR approach* OR model* OR strateg* OR method* OR project* OR practice* OR instrument* OR tool* OR framework* ORprotocol* OR guid* OR scale* OR system* OR inventor* OR metric* OR template* OR profile* OR criteria OR questionnaire* OR refer* OR assess*OR "case plan*" OR "case formulat*" OR "management plan*" OR "treatment plan*" OR "support plan*" OR "case manage*" OR "risk manage*" ORprogress* OR monitor* OR supervis* OR measur* ) ) AND ( TITLE-ABS-KEY ( radicali* OR extremis* OR terroris* OR jihadi* OR islamis* OR salafi*OR right-wing OR "right wing" OR extreme-right OR "extreme right" OR neo-nazi OR far-right OR "far right" OR nationalist* OR "white supremacis*"OR left-wing OR "left wing" OR extreme-left OR "extreme left" OR anarch* OR "single issue" OR single-issue ) ) AND ( LIMIT-TO ( PUBYEAR , 2014 )OR LIMIT-TO ( PUBYEAR , 2013 ) ) AND ( EXCLUDE ( SUBJAREA , "engi" ) OR EXCLUDE ( SUBJAREA , "envi" ) OR EXCLUDE ( SUBJAREA , "phys" ) OREXCLUDE ( SUBJAREA , "bioc" ) OR EXCLUDE ( SUBJAREA , "mate" ) OR EXCLUDE ( SUBJAREA , "chem" ) OR EXCLUDE ( SUBJAREA , "phar" ) OREXCLUDE ( SUBJAREA , "ener" ) OR EXCLUDE ( SUBJAREA , "ceng" ) OR EXCLUDE ( SUBJAREA , "eart" ) OR EXCLUDE ( SUBJAREA , "immu" ) ) AND (EXCLUDE ( DOCTYPE , "ed" ) OR EXCLUDE ( DOCTYPE , "no" ) OR EXCLUDE ( DOCTYPE , "le" ) ) | 1486 |
| ( TITLE-ABS-KEY ( prevent* OR reduc* OR counter* OR disengage* OR rehab* OR reintegrat* OR re-integrat* OR re-entry OR reentry OR desist*OR recidivism OR deradical* OR de-radical* OR exit* ) ) AND ( TITLE-ABS-KEY ( initiative* OR interven* OR program* OR policy OR policies ORscheme* OR treat* OR approach* OR model* OR strateg* OR method* OR project* OR practice* OR instrument* OR tool* OR framework* ORprotocol* OR guid* OR scale* OR system* OR inventor* OR metric* OR template* OR profile* OR criteria OR questionnaire* OR refer* OR assess*OR "case plan*" OR "case formulat*" OR "management plan*" OR "treatment plan*" OR "support plan*" OR "case manage*" OR "risk manage*" ORprogress* OR monitor* OR supervis* OR measur* ) ) AND ( TITLE-ABS-KEY ( radicali* OR extremis* OR terroris* OR jihadi* OR islamis* OR salafi*OR right-wing OR "right wing" OR extreme-right OR "extreme right" OR neo-nazi OR far-right OR "far right" OR nationalist* OR "white supremacis*"OR left-wing OR "left wing" OR extreme-left OR "extreme left" OR anarch* OR "single issue" OR single-issue ) ) AND ( LIMIT-TO ( PUBYEAR , 2014 )OR LIMIT-TO ( PUBYEAR , 2013 ) ) AND ( EXCLUDE ( SUBJAREA , "engi" ) OR EXCLUDE ( SUBJAREA , "envi" ) OR EXCLUDE ( SUBJAREA , "phys" ) OREXCLUDE ( SUBJAREA , "bioc" ) OR EXCLUDE ( SUBJAREA , "mate" ) OR EXCLUDE ( SUBJAREA , "chem" ) OR EXCLUDE ( SUBJAREA , "phar" ) OREXCLUDE ( SUBJAREA , "ener" ) OR EXCLUDE ( SUBJAREA , "ceng" ) OR EXCLUDE ( SUBJAREA , "eart" ) OR EXCLUDE ( SUBJAREA , "immu" ) ) | 1509 |
| ( TITLE-ABS-KEY ( prevent* OR reduc* OR counter* OR disengage* OR rehab* OR reintegrat* OR re-integrat* OR re-entry OR reentry OR desist*OR recidivism OR deradical* OR de-radical* OR exit* ) ) AND ( TITLE-ABS-KEY ( initiative* OR interven* OR program* OR policy OR policies ORscheme* OR treat* OR approach* OR model* OR strateg* OR method* OR project* OR practice* OR instrument* OR tool* OR framework* ORprotocol* OR guid* OR scale* OR system* OR inventor* OR metric* OR template* OR profile* OR criteria OR questionnaire* OR refer* OR assess*OR "case plan*" OR "case formulat*" OR "management plan*" OR "treatment plan*" OR "support plan*" OR "case manage*" OR "risk manage*" ORprogress* OR monitor* OR supervis* OR measur* ) ) AND ( TITLE-ABS-KEY ( radicali* OR extremis* OR terroris* OR jihadi* OR islamis* OR salafi*OR right-wing OR "right wing" OR extreme-right OR "extreme right" OR neo-nazi OR far-right OR "far right" OR nationalist* OR "white supremacis*"OR left-wing OR "left wing" OR extreme-left OR "extreme left" OR anarch* OR "single issue" OR single-issue ) ) AND ( LIMIT-TO ( PUBYEAR , 2014 ) ORLIMIT-TO ( PUBYEAR , 2013 ) ) | 1964 |
| ( TITLE-ABS-KEY ( prevent* OR reduc* OR counter* OR disengage* OR rehab* OR reintegrat* OR re-integrat* OR re-entry OR reentry OR desist*OR recidivism OR deradical* OR de-radical* OR exit* ) ) AND ( TITLE-ABS-KEY ( initiative* OR interven* OR program* OR policy OR policies ORscheme* OR treat* OR approach* OR model* OR strateg* OR method* OR project* OR practice* OR instrument* OR tool* OR framework* ORprotocol* OR guid* OR scale* OR system* OR inventor* OR metric* OR template* OR profile* OR criteria OR questionnaire* OR refer* OR assess*OR "case plan*" OR "case formulat*" OR "management plan*" OR "treatment plan*" OR "support plan*" OR "case manage*" OR "risk manage*" ORprogress* OR monitor* OR supervis* OR measur* ) ) AND ( TITLE-ABS-KEY ( radicali* OR extremis* OR terroris* OR jihadi* OR islamis* OR salafi*OR right-wing OR "right wing" OR extreme-right OR "extreme right" OR neo-nazi OR far-right OR "far right" OR nationalist* OR "white supremacis*"OR left-wing OR "left wing" OR extreme-left OR "extreme left" OR anarch* OR "single issue" OR single-issue ) ) AND ( LIMIT-TO ( PUBYEAR , 2016 )OR LIMIT-TO ( PUBYEAR , 2015 ) ) AND ( EXCLUDE ( SUBJAREA , "engi" ) OR EXCLUDE ( SUBJAREA , "phys" ) OR EXCLUDE ( SUBJAREA , "envi" ) OREXCLUDE ( SUBJAREA , "bioc" ) OR EXCLUDE ( SUBJAREA , "mate" ) OR EXCLUDE ( SUBJAREA , "eart" ) OR EXCLUDE ( SUBJAREA , "phar" ) OREXCLUDE ( SUBJAREA , "chem" ) OR EXCLUDE ( SUBJAREA , "ener" ) OR EXCLUDE ( SUBJAREA , "ceng" ) OR EXCLUDE ( SUBJAREA , "immu" ) ) AND( EXCLUDE ( DOCTYPE , "no" ) OR EXCLUDE ( DOCTYPE , "ed" ) OR EXCLUDE ( DOCTYPE , "le" ) OR EXCLUDE ( DOCTYPE , "cr" ) ) | 1634 |
| ( TITLE-ABS-KEY ( prevent* OR reduc* OR counter* OR disengage* OR rehab* OR reintegrat* OR re-integrat* OR re-entry OR reentry OR desist*OR recidivism OR deradical* OR de-radical* OR exit* ) ) AND ( TITLE-ABS-KEY ( initiative* OR interven* OR program* OR policy OR policies ORscheme* OR treat* OR approach* OR model* OR strateg* OR method* OR project* OR practice* OR instrument* OR tool* OR framework* ORprotocol* OR guid* OR scale* OR system* OR inventor* OR metric* OR template* OR profile* OR criteria OR questionnaire* OR refer* OR assess*OR "case plan*" OR "case formulat*" OR "management plan*" OR "treatment plan*" OR "support plan*" OR "case manage*" OR "risk manage*" ORprogress* OR monitor* OR supervis* OR measur* ) ) AND ( TITLE-ABS-KEY ( radicali* OR extremis* OR terroris* OR jihadi* OR islamis* OR salafi*OR right-wing OR "right wing" OR extreme-right OR "extreme right" OR neo-nazi OR far-right OR "far right" OR nationalist* OR "white supremacis*"OR left-wing OR "left wing" OR extreme-left OR "extreme left" OR anarch* OR "single issue" OR single-issue ) ) AND ( LIMIT-TO ( PUBYEAR , 2016 )OR LIMIT-TO ( PUBYEAR , 2015 ) ) AND ( EXCLUDE ( SUBJAREA , "engi" ) OR EXCLUDE ( SUBJAREA , "phys" ) OR EXCLUDE ( SUBJAREA , "envi" ) OREXCLUDE ( SUBJAREA , "bioc" ) OR EXCLUDE ( SUBJAREA , "mate" ) OR EXCLUDE ( SUBJAREA , "eart" ) OR EXCLUDE ( SUBJAREA , "phar" ) OREXCLUDE ( SUBJAREA , "chem" ) OR EXCLUDE ( SUBJAREA , "ener" ) OR EXCLUDE ( SUBJAREA , "ceng" ) OR EXCLUDE ( SUBJAREA , "immu" ) ) | 1659 |
| ( TITLE-ABS-KEY ( prevent* OR reduc* OR counter* OR disengage* OR rehab* OR reintegrat* OR re-integrat* OR re-entry OR reentry OR desist*OR recidivism OR deradical* OR de-radical* OR exit* ) ) AND ( TITLE-ABS-KEY ( initiative* OR interven* OR program* OR policy OR policies ORscheme* OR treat* OR approach* OR model* OR strateg* OR method* OR project* OR practice* OR instrument* OR tool* OR framework* ORprotocol* OR guid* OR scale* OR system* OR inventor* OR metric* OR template* OR profile* OR criteria OR questionnaire* OR refer* OR assess*OR "case plan*" OR "case formulat*" OR "management plan*" OR "treatment plan*" OR "support plan*" OR "case manage*" OR "risk manage*" ORprogress* OR monitor* OR supervis* OR measur* ) ) AND ( TITLE-ABS-KEY ( radicali* OR extremis* OR terroris* OR jihadi* OR islamis* OR salafi*OR right-wing OR "right wing" OR extreme-right OR "extreme right" OR neo-nazi OR far-right OR "far right" OR nationalist* OR "white supremacis*"OR left-wing OR "left wing" OR extreme-left OR "extreme left" OR anarch* OR "single issue" OR single-issue ) ) AND ( LIMIT-TO ( PUBYEAR , 2016 )OR LIMIT-TO ( PUBYEAR , 2015 ) ) | 2163 |
| ( TITLE-ABS-KEY ( prevent* OR reduc* OR counter* OR disengage* OR rehab* OR reintegrat* OR re-integrat* OR re-entry OR reentry OR desist*OR recidivism OR deradical* OR de-radical* OR exit* ) ) AND ( TITLE-ABS-KEY ( initiative* OR interven* OR program* OR policy OR policies ORscheme* OR treat* OR approach* OR model* OR strateg* OR method* OR project* OR practice* OR instrument* OR tool* OR framework* ORprotocol* OR guid* OR scale* OR system* OR inventor* OR metric* OR template* OR profile* OR criteria OR questionnaire* OR refer* OR assess*OR "case plan*" OR "case formulat*" OR "management plan*" OR "treatment plan*" OR "support plan*" OR "case manage*" OR "risk manage*" ORprogress* OR monitor* OR supervis* OR measur* ) ) AND ( TITLE-ABS-KEY ( radicali* OR extremis* OR terroris* OR jihadi* OR islamis* OR salafi*OR right-wing OR "right wing" OR extreme-right OR "extreme right" OR neo-nazi OR far-right OR "far right" OR nationalist* OR "white supremacis*"OR left-wing OR "left wing" OR extreme-left OR "extreme left" OR anarch* OR "single issue" OR single-issue ) ) AND ( LIMIT-TO ( PUBYEAR , 2017 ) )AND ( EXCLUDE ( SUBJAREA , "engi" ) OR EXCLUDE ( SUBJAREA , "phys" ) OR EXCLUDE ( SUBJAREA , "envi" ) OR EXCLUDE ( SUBJAREA , "ener" ) OREXCLUDE ( SUBJAREA , "bioc" ) OR EXCLUDE ( SUBJAREA , "eart" ) OR EXCLUDE ( SUBJAREA , "mate" ) OR EXCLUDE ( SUBJAREA , "chem" ) OREXCLUDE ( SUBJAREA , "ceng" ) OR EXCLUDE ( SUBJAREA , "phar" ) OR EXCLUDE ( SUBJAREA , "immu" ) ) AND ( EXCLUDE ( DOCTYPE , "no" ) OREXCLUDE ( DOCTYPE , "ed" ) OR EXCLUDE ( DOCTYPE , "cr" ) OR EXCLUDE ( DOCTYPE , "le" ) ) | 1106 |
| ( TITLE-ABS-KEY ( prevent* OR reduc* OR counter* OR disengage* OR rehab* OR reintegrat* OR re-integrat* OR re-entry OR reentry OR desist*OR recidivism OR deradical* OR de-radical* OR exit* ) ) AND ( TITLE-ABS-KEY ( initiative* OR interven* OR program* OR policy OR policies ORscheme* OR treat* OR approach* OR model* OR strateg* OR method* OR project* OR practice* OR instrument* OR tool* OR framework* ORprotocol* OR guid* OR scale* OR system* OR inventor* OR metric* OR template* OR profile* OR criteria OR questionnaire* OR refer* OR assess*OR "case plan*" OR "case formulat*" OR "management plan*" OR "treatment plan*" OR "support plan*" OR "case manage*" OR "risk manage*" ORprogress* OR monitor* OR supervis* OR measur* ) ) AND ( TITLE-ABS-KEY ( radicali* OR extremis* OR terroris* OR jihadi* OR islamis* OR salafi*OR right-wing OR "right wing" OR extreme-right OR "extreme right" OR neo-nazi OR far-right OR "far right" OR nationalist* OR "white supremacis*"OR left-wing OR "left wing" OR extreme-left OR "extreme left" OR anarch* OR "single issue" OR single-issue ) ) AND ( LIMIT-TO ( PUBYEAR , 2017 ) )AND ( EXCLUDE ( SUBJAREA , "engi" ) OR EXCLUDE ( SUBJAREA , "phys" ) OR EXCLUDE ( SUBJAREA , "envi" ) OR EXCLUDE ( SUBJAREA , "ener" ) OREXCLUDE ( SUBJAREA , "bioc" ) OR EXCLUDE ( SUBJAREA , "eart" ) OR EXCLUDE ( SUBJAREA , "mate" ) OR EXCLUDE ( SUBJAREA , "chem" ) OREXCLUDE ( SUBJAREA , "ceng" ) OR EXCLUDE ( SUBJAREA , "phar" ) OR EXCLUDE ( SUBJAREA , "immu" ) ) | 1140 |
| ( TITLE-ABS-KEY ( prevent* OR reduc* OR counter* OR disengage* OR rehab* OR reintegrat* OR re-integrat* OR re-entry OR reentry OR desist*OR recidivism OR deradical* OR de-radical* OR exit* ) ) AND ( TITLE-ABS-KEY ( initiative* OR interven* OR program* OR policy OR policies ORscheme* OR treat* OR approach* OR model* OR strateg* OR method* OR project* OR practice* OR instrument* OR tool* OR framework* ORprotocol* OR guid* OR scale* OR system* OR inventor* OR metric* OR template* OR profile* OR criteria OR questionnaire* OR refer* OR assess*OR "case plan*" OR "case formulat*" OR "management plan*" OR "treatment plan*" OR "support plan*" OR "case manage*" OR "risk manage*" ORprogress* OR monitor* OR supervis* OR measur* ) ) AND ( TITLE-ABS-KEY ( radicali* OR extremis* OR terroris* OR jihadi* OR islamis* OR salafi*OR right-wing OR "right wing" OR extreme-right OR "extreme right" OR neo-nazi OR far-right OR "far right" OR nationalist* OR "white supremacis*"OR left-wing OR "left wing" OR extreme-left OR "extreme left" OR anarch* OR "single issue" OR single-issue ) ) AND ( LIMIT-TO ( PUBYEAR , 2017 ) ) | 1429 |
| ( TITLE-ABS-KEY ( prevent* OR reduc* OR counter* OR disengage* OR rehab* OR reintegrat* OR re-integrat* OR re-entry OR reentry OR desist*OR recidivism OR deradical* OR de-radical* OR exit* ) ) AND ( TITLE-ABS-KEY ( initiative* OR interven* OR program* OR policy OR policies ORscheme* OR treat* OR approach* OR model* OR strateg* OR method* OR project* OR practice* OR instrument* OR tool* OR framework* ORprotocol* OR guid* OR scale* OR system* OR inventor* OR metric* OR template* OR profile* OR criteria OR questionnaire* OR refer* OR assess*OR "case plan*" OR "case formulat*" OR "management plan*" OR "treatment plan*" OR "support plan*" OR "case manage*" OR "risk manage*" ORprogress* OR monitor* OR supervis* OR measur* ) ) AND ( TITLE-ABS-KEY ( radicali* OR extremis* OR terroris* OR jihadi* OR islamis* OR salafi*OR right-wing OR "right wing" OR extreme-right OR "extreme right" OR neo-nazi OR far-right OR "far right" OR nationalist* OR "white supremacis*"OR left-wing OR "left wing" OR extreme-left OR "extreme left" OR anarch* OR "single issue" OR single-issue ) ) AND ( LIMIT-TO ( PUBYEAR , 2018 ) )AND ( EXCLUDE ( SUBJAREA , "engi" ) OR EXCLUDE ( SUBJAREA , "envi" ) OR EXCLUDE ( SUBJAREA , "phys" ) OR EXCLUDE ( SUBJAREA , "mate" )OR EXCLUDE ( SUBJAREA , "bioc" ) OR EXCLUDE ( SUBJAREA , "ener" ) OR EXCLUDE ( SUBJAREA , "chem" ) OR EXCLUDE ( SUBJAREA , "eart" ) OREXCLUDE ( SUBJAREA , "ceng" ) OR EXCLUDE ( SUBJAREA , "phar" ) OR EXCLUDE ( SUBJAREA , "immu" ) ) AND ( EXCLUDE ( DOCTYPE , "ed" ) OREXCLUDE ( DOCTYPE , "no" ) OR EXCLUDE ( DOCTYPE , "le" ) ) | 1052 |
| ( TITLE-ABS-KEY ( prevent* OR reduc* OR counter* OR disengage* OR rehab* OR reintegrat* OR re-integrat* OR re-entry OR reentry OR desist*OR recidivism OR deradical* OR de-radical* OR exit* ) ) AND ( TITLE-ABS-KEY ( initiative* OR interven* OR program* OR policy OR policies ORscheme* OR treat* OR approach* OR model* OR strateg* OR method* OR project* OR practice* OR instrument* OR tool* OR framework* ORprotocol* OR guid* OR scale* OR system* OR inventor* OR metric* OR template* OR profile* OR criteria OR questionnaire* OR refer* OR assess*OR "case plan*" OR "case formulat*" OR "management plan*" OR "treatment plan*" OR "support plan*" OR "case manage*" OR "risk manage*" ORprogress* OR monitor* OR supervis* OR measur* ) ) AND ( TITLE-ABS-KEY ( radicali* OR extremis* OR terroris* OR jihadi* OR islamis* OR salafi*OR right-wing OR "right wing" OR extreme-right OR "extreme right" OR neo-nazi OR far-right OR "far right" OR nationalist* OR "white supremacis*"OR left-wing OR "left wing" OR extreme-left OR "extreme left" OR anarch* OR "single issue" OR single-issue ) ) AND ( LIMIT-TO ( PUBYEAR , 2018 ) )AND ( EXCLUDE ( SUBJAREA , "engi" ) OR EXCLUDE ( SUBJAREA , "envi" ) OR EXCLUDE ( SUBJAREA , "phys" ) OR EXCLUDE ( SUBJAREA , "mate" )OR EXCLUDE ( SUBJAREA , "bioc" ) OR EXCLUDE ( SUBJAREA , "ener" ) OR EXCLUDE ( SUBJAREA , "chem" ) OR EXCLUDE ( SUBJAREA , "eart" ) OREXCLUDE ( SUBJAREA , "ceng" ) OR EXCLUDE ( SUBJAREA , "phar" ) OR EXCLUDE ( SUBJAREA , "immu" ) ) | 1066 |
| ( TITLE-ABS-KEY ( prevent* OR reduc* OR counter* OR disengage* OR rehab* OR reintegrat* OR re-integrat* OR re-entry OR reentry OR desist*OR recidivism OR deradical* OR de-radical* OR exit* ) ) AND ( TITLE-ABS-KEY ( initiative* OR interven* OR program* OR policy OR policies ORscheme* OR treat* OR approach* OR model* OR strateg* OR method* OR project* OR practice* OR instrument* OR tool* OR framework* ORprotocol* OR guid* OR scale* OR system* OR inventor* OR metric* OR template* OR profile* OR criteria OR questionnaire* OR refer* OR assess*OR "case plan*" OR "case formulat*" OR "management plan*" OR "treatment plan*" OR "support plan*" OR "case manage*" OR "risk manage*" ORprogress* OR monitor* OR supervis* OR measur* ) ) AND ( TITLE-ABS-KEY ( radicali* OR extremis* OR terroris* OR jihadi* OR islamis* OR salafi*OR right-wing OR "right wing" OR extreme-right OR "extreme right" OR neo-nazi OR far-right OR "far right" OR nationalist* OR "white supremacis*"OR left-wing OR "left wing" OR extreme-left OR "extreme left" OR anarch* OR "single issue" OR single-issue ) ) AND ( LIMIT-TO ( PUBYEAR , 2018 ) ) | 1394 |
| ( TITLE-ABS-KEY ( prevent* OR reduc* OR counter* OR disengage* OR rehab* OR reintegrat* OR re-integrat* OR re-entry OR reentry OR desist*OR recidivism OR deradical* OR de-radical* OR exit* ) ) AND ( TITLE-ABS-KEY ( initiative* OR interven* OR program* OR policy OR policies ORscheme* OR treat* OR approach* OR model* OR strateg* OR method* OR project* OR practice* OR instrument* OR tool* OR framework* ORprotocol* OR guid* OR scale* OR system* OR inventor* OR metric* OR template* OR profile* OR criteria OR questionnaire* OR refer* OR assess*OR "case plan*" OR "case formulat*" OR "management plan*" OR "treatment plan*" OR "support plan*" OR "case manage*" OR "risk manage*" ORprogress* OR monitor* OR supervis* OR measur* ) ) AND ( TITLE-ABS-KEY ( radicali* OR extremis* OR terroris* OR jihadi* OR islamis* OR salafi*OR right-wing OR "right wing" OR extreme-right OR "extreme right" OR neo-nazi OR far-right OR "far right" OR nationalist* OR "white supremacis*"OR left-wing OR "left wing" OR extreme-left OR "extreme left" OR anarch* OR "single issue" OR single-issue ) ) AND ( LIMIT-TO ( PUBYEAR , 2019 ) )AND ( EXCLUDE ( SUBJAREA , "engi" ) OR EXCLUDE ( SUBJAREA , "envi" ) OR EXCLUDE ( SUBJAREA , "phys" ) OR EXCLUDE ( SUBJAREA , "mate" ) OREXCLUDE ( SUBJAREA , "eart" ) OR EXCLUDE ( SUBJAREA , "ener" ) OR EXCLUDE ( SUBJAREA , "bioc" ) OR EXCLUDE ( SUBJAREA , "ceng" ) OREXCLUDE ( SUBJAREA , "chem" ) OR EXCLUDE ( SUBJAREA , "phar" ) OR EXCLUDE ( SUBJAREA , "immu" ) ) AND ( EXCLUDE ( DOCTYPE , "no" ) OREXCLUDE ( DOCTYPE , "ed" ) OR EXCLUDE ( DOCTYPE , "le" ) OR EXCLUDE ( DOCTYPE , "cr" ) ) | 1193 |
| ( TITLE-ABS-KEY ( prevent* OR reduc* OR counter* OR disengage* OR rehab* OR reintegrat* OR re-integrat* OR re-entry OR reentry OR desist*OR recidivism OR deradical* OR de-radical* OR exit* ) ) AND ( TITLE-ABS-KEY ( initiative* OR interven* OR program* OR policy OR policies ORscheme* OR treat* OR approach* OR model* OR strateg* OR method* OR project* OR practice* OR instrument* OR tool* OR framework* ORprotocol* OR guid* OR scale* OR system* OR inventor* OR metric* OR template* OR profile* OR criteria OR questionnaire* OR refer* OR assess*OR "case plan*" OR "case formulat*" OR "management plan*" OR "treatment plan*" OR "support plan*" OR "case manage*" OR "risk manage*" ORprogress* OR monitor* OR supervis* OR measur* ) ) AND ( TITLE-ABS-KEY ( radicali* OR extremis* OR terroris* OR jihadi* OR islamis* OR salafi*OR right-wing OR "right wing" OR extreme-right OR "extreme right" OR neo-nazi OR far-right OR "far right" OR nationalist* OR "white supremacis*"OR left-wing OR "left wing" OR extreme-left OR "extreme left" OR anarch* OR "single issue" OR single-issue ) ) AND ( LIMIT-TO ( PUBYEAR , 2019 ) )AND ( EXCLUDE ( SUBJAREA , "engi" ) OR EXCLUDE ( SUBJAREA , "envi" ) OR EXCLUDE ( SUBJAREA , "phys" ) OR EXCLUDE ( SUBJAREA , "mate" )OR EXCLUDE ( SUBJAREA , "eart" ) OR EXCLUDE ( SUBJAREA , "ener" ) OR EXCLUDE ( SUBJAREA , "bioc" ) OR EXCLUDE ( SUBJAREA , "ceng" ) OREXCLUDE ( SUBJAREA , "chem" ) OR EXCLUDE ( SUBJAREA , "phar" ) OR EXCLUDE ( SUBJAREA , "immu" ) ) | 1209 |
| ( TITLE-ABS-KEY ( prevent* OR reduc* OR counter* OR disengage* OR rehab* OR reintegrat* OR re-integrat* OR re-entry OR reentry OR desist*OR recidivism OR deradical* OR de-radical* OR exit* ) ) AND ( TITLE-ABS-KEY ( initiative* OR interven* OR program* OR policy OR policies ORscheme* OR treat* OR approach* OR model* OR strateg* OR method* OR project* OR practice* OR instrument* OR tool* OR framework* ORprotocol* OR guid* OR scale* OR system* OR inventor* OR metric* OR template* OR profile* OR criteria OR questionnaire* OR refer* OR assess*OR "case plan*" OR "case formulat*" OR "management plan*" OR "treatment plan*" OR "support plan*" OR "case manage*" OR "risk manage*" ORprogress* OR monitor* OR supervis* OR measur* ) ) AND ( TITLE-ABS-KEY ( radicali* OR extremis* OR terroris* OR jihadi* OR islamis* OR salafi*OR right-wing OR "right wing" OR extreme-right OR "extreme right" OR neo-nazi OR far-right OR "far right" OR nationalist* OR "white supremacis*"OR left-wing OR "left wing" OR extreme-left OR "extreme left" OR anarch* OR "single issue" OR single-issue ) ) AND ( LIMIT-TO ( PUBYEAR , 2019 ) ) | 1562 |
| ( TITLE-ABS-KEY ( prevent* OR reduc* OR counter* OR disengage* OR rehab* OR reintegrat* OR re-integrat* OR re-entry OR reentry OR desist*OR recidivism OR deradical* OR de-radical* OR exit* ) ) AND ( TITLE-ABS-KEY ( initiative* OR interven* OR program* OR policy OR policies ORscheme* OR treat* OR approach* OR model* OR strateg* OR method* OR project* OR practice* OR instrument* OR tool* OR framework* ORprotocol* OR guid* OR scale* OR system* OR inventor* OR metric* OR template* OR profile* OR criteria OR questionnaire* OR refer* OR assess*OR "case plan*" OR "case formulat*" OR "management plan*" OR "treatment plan*" OR "support plan*" OR "case manage*" OR "risk manage*" ORprogress* OR monitor* OR supervis* OR measur* ) ) AND ( TITLE-ABS-KEY ( radicali* OR extremis* OR terroris* OR jihadi* OR islamis* OR salafi*OR right-wing OR "right wing" OR extreme-right OR "extreme right" OR neo-nazi OR far-right OR "far right" OR nationalist* OR "white supremacis*"OR left-wing OR "left wing" OR extreme-left OR "extreme left" OR anarch* OR "single issue" OR single-issue ) ) AND ( LIMIT-TO ( PUBYEAR , 2020 ) )AND ( EXCLUDE ( SUBJAREA , "engi" ) OR EXCLUDE ( SUBJAREA , "envi" ) OR EXCLUDE ( SUBJAREA , "phys" ) OR EXCLUDE ( SUBJAREA , "ener" ) OREXCLUDE ( SUBJAREA , "mate" ) OR EXCLUDE ( SUBJAREA , "chem" ) OR EXCLUDE ( SUBJAREA , "bioc" ) OR EXCLUDE ( SUBJAREA , "eart" ) OREXCLUDE ( SUBJAREA , "phar" ) OR EXCLUDE ( SUBJAREA , "ceng" ) OR EXCLUDE ( SUBJAREA , "immu" ) ) AND ( EXCLUDE ( DOCTYPE , "no" ) OREXCLUDE ( DOCTYPE , "ed" ) OR EXCLUDE ( DOCTYPE , "cr" ) ) | 1162 |
| ( TITLE-ABS-KEY ( prevent* OR reduc* OR counter* OR disengage* OR rehab* OR reintegrat* OR re-integrat* OR re-entry OR reentry OR desist*OR recidivism OR deradical* OR de-radical* OR exit* ) ) AND ( TITLE-ABS-KEY ( initiative* OR interven* OR program* OR policy OR policies ORscheme* OR treat* OR approach* OR model* OR strateg* OR method* OR project* OR practice* OR instrument* OR tool* OR framework* ORprotocol* OR guid* OR scale* OR system* OR inventor* OR metric* OR template* OR profile* OR criteria OR questionnaire* OR refer* OR assess*OR "case plan*" OR "case formulat*" OR "management plan*" OR "treatment plan*" OR "support plan*" OR "case manage*" OR "risk manage*" ORprogress* OR monitor* OR supervis* OR measur* ) ) AND ( TITLE-ABS-KEY ( radicali* OR extremis* OR terroris* OR jihadi* OR islamis* OR salafi*OR right-wing OR "right wing" OR extreme-right OR "extreme right" OR neo-nazi OR far-right OR "far right" OR nationalist* OR "white supremacis*"OR left-wing OR "left wing" OR extreme-left OR "extreme left" OR anarch* OR "single issue" OR single-issue ) ) AND ( LIMIT-TO ( PUBYEAR , 2020 ) )AND ( EXCLUDE ( SUBJAREA , "engi" ) OR EXCLUDE ( SUBJAREA , "envi" ) OR EXCLUDE ( SUBJAREA , "phys" ) OR EXCLUDE ( SUBJAREA , "ener" ) OREXCLUDE ( SUBJAREA , "mate" ) OR EXCLUDE ( SUBJAREA , "chem" ) OR EXCLUDE ( SUBJAREA , "bioc" ) OR EXCLUDE ( SUBJAREA , "eart" ) OREXCLUDE ( SUBJAREA , "phar" ) OR EXCLUDE ( SUBJAREA , "ceng" ) OR EXCLUDE ( SUBJAREA , "immu" ) ) | 1175 |
| ( TITLE-ABS-KEY ( prevent* OR reduc* OR counter* OR disengage* OR rehab* OR reintegrat* OR re-integrat* OR re-entry OR reentry OR desist*OR recidivism OR deradical* OR de-radical* OR exit* ) ) AND ( TITLE-ABS-KEY ( initiative* OR interven* OR program* OR policy OR policies ORscheme* OR treat* OR approach* OR model* OR strateg* OR method* OR project* OR practice* OR instrument* OR tool* OR framework* ORprotocol* OR guid* OR scale* OR system* OR inventor* OR metric* OR template* OR profile* OR criteria OR questionnaire* OR refer* OR assess*OR "case plan*" OR "case formulat*" OR "management plan*" OR "treatment plan*" OR "support plan*" OR "case manage*" OR "risk manage*" ORprogress* OR monitor* OR supervis* OR measur* ) ) AND ( TITLE-ABS-KEY ( radicali* OR extremis* OR terroris* OR jihadi* OR islamis* OR salafi*OR right-wing OR "right wing" OR extreme-right OR "extreme right" OR neo-nazi OR far-right OR "far right" OR nationalist* OR "white supremacis*"OR left-wing OR "left wing" OR extreme-left OR "extreme left" OR anarch* OR "single issue" OR single-issue ) ) AND ( LIMIT-TO ( PUBYEAR , 2020 ) ) | 1620 |
| ( TITLE-ABS-KEY ( prevent* OR reduc* OR counter* OR disengage* OR rehab* OR reintegrat* OR re-integrat* OR re-entry OR reentry OR desist*OR recidivism OR deradical* OR de-radical* OR exit* ) ) AND ( TITLE-ABS-KEY ( initiative* OR interven* OR program* OR policy OR policies ORscheme* OR treat* OR approach* OR model* OR strateg* OR method* OR project* OR practice* OR instrument* OR tool* OR framework* ORprotocol* OR guid* OR scale* OR system* OR inventor* OR metric* OR template* OR profile* OR criteria OR questionnaire* OR refer* OR assess*OR "case plan*" OR "case formulat*" OR "management plan*" OR "treatment plan*" OR "support plan*" OR "case manage*" OR "risk manage*" ORprogress* OR monitor* OR supervis* OR measur* ) ) AND ( TITLE-ABS-KEY ( radicali* OR extremis* OR terroris* OR jihadi* OR islamis* OR salafi*OR right-wing OR "right wing" OR extreme-right OR "extreme right" OR neo-nazi OR far-right OR "far right" OR nationalist* OR "white supremacis*"OR left-wing OR "left wing" OR extreme-left OR "extreme left" OR anarch* OR "single issue" OR single-issue ) ) AND ( LIMIT-TO ( PUBYEAR , 2021 ) )AND ( EXCLUDE ( SUBJAREA , "engi" ) OR EXCLUDE ( SUBJAREA , "envi" ) OR EXCLUDE ( SUBJAREA , "phys" ) OR EXCLUDE ( SUBJAREA , "eart" ) OREXCLUDE ( SUBJAREA , "mate" ) OR EXCLUDE ( SUBJAREA , "ener" ) OR EXCLUDE ( SUBJAREA , "bioc" ) OR EXCLUDE ( SUBJAREA , "phar" ) OREXCLUDE ( SUBJAREA , "agri" ) OR EXCLUDE ( SUBJAREA , "chem" ) OR EXCLUDE ( SUBJAREA , "ceng" ) OR EXCLUDE ( SUBJAREA , "immu" ) ) AND (EXCLUDE ( DOCTYPE , "ed" ) OR EXCLUDE ( DOCTYPE , "le" ) OR EXCLUDE ( DOCTYPE , "no" ) OR EXCLUDE ( DOCTYPE , "cr" ) ) | 1320 |
| ( TITLE-ABS-KEY ( prevent* OR reduc* OR counter* OR disengage* OR rehab* OR reintegrat* OR re-integrat* OR re-entry OR reentry OR desist*OR recidivism OR deradical* OR de-radical* OR exit* ) ) AND ( TITLE-ABS-KEY ( initiative* OR interven* OR program* OR policy OR policies ORscheme* OR treat* OR approach* OR model* OR strateg* OR method* OR project* OR practice* OR instrument* OR tool* OR framework* ORprotocol* OR guid* OR scale* OR system* OR inventor* OR metric* OR template* OR profile* OR criteria OR questionnaire* OR refer* OR assess*OR "case plan*" OR "case formulat*" OR "management plan*" OR "treatment plan*" OR "support plan*" OR "case manage*" OR "risk manage*" ORprogress* OR monitor* OR supervis* OR measur* ) ) AND ( TITLE-ABS-KEY ( radicali* OR extremis* OR terroris* OR jihadi* OR islamis* OR salafi*OR right-wing OR "right wing" OR extreme-right OR "extreme right" OR neo-nazi OR far-right OR "far right" OR nationalist* OR "white supremacis*"OR left-wing OR "left wing" OR extreme-left OR "extreme left" OR anarch* OR "single issue" OR single-issue ) ) AND ( LIMIT-TO ( PUBYEAR , 2021 ) )AND ( EXCLUDE ( SUBJAREA , "engi" ) OR EXCLUDE ( SUBJAREA , "envi" ) OR EXCLUDE ( SUBJAREA , "phys" ) OR EXCLUDE ( SUBJAREA , "eart" ) OREXCLUDE ( SUBJAREA , "mate" ) OR EXCLUDE ( SUBJAREA , "ener" ) OR EXCLUDE ( SUBJAREA , "bioc" ) OR EXCLUDE ( SUBJAREA , "phar" ) OREXCLUDE ( SUBJAREA , "agri" ) OR EXCLUDE ( SUBJAREA , "chem" ) OR EXCLUDE ( SUBJAREA , "ceng" ) OR EXCLUDE ( SUBJAREA , "immu" ) ) | 1332 |
| ( TITLE-ABS-KEY ( prevent* OR reduc* OR counter* OR disengage* OR rehab* OR reintegrat* OR re-integrat* OR re-entry OR reentry OR desist*OR recidivism OR deradical* OR de-radical* OR exit* ) ) AND ( TITLE-ABS-KEY ( initiative* OR interven* OR program* OR policy OR policies ORscheme* OR treat* OR approach* OR model* OR strateg* OR method* OR project* OR practice* OR instrument* OR tool* OR framework* ORprotocol* OR guid* OR scale* OR system* OR inventor* OR metric* OR template* OR profile* OR criteria OR questionnaire* OR refer* OR assess*OR "case plan*" OR "case formulat*" OR "management plan*" OR "treatment plan*" OR "support plan*" OR "case manage*" OR "risk manage*" ORprogress* OR monitor* OR supervis* OR measur* ) ) AND ( TITLE-ABS-KEY ( radicali* OR extremis* OR terroris* OR jihadi* OR islamis* OR salafi*OR right-wing OR "right wing" OR extreme-right OR "extreme right" OR neo-nazi OR far-right OR "far right" OR nationalist* OR "white supremacis*"OR left-wing OR "left wing" OR extreme-left OR "extreme left" OR anarch* OR "single issue" OR single-issue ) ) AND ( LIMIT-TO ( PUBYEAR , 2021 ) ) | 1699 |
| ( TITLE-ABS-KEY ( prevent* OR reduc* OR counter* OR disengage* OR rehab* OR reintegrat* OR re-integrat* OR re-entry OR reentry OR desist* ORrecidivism OR deradical* OR de-radical* OR exit* ) ) AND ( TITLE-ABS-KEY ( initiative* OR interven* OR program* OR policy OR policies OR scheme*OR treat* OR approach* OR model* OR strateg* OR method* OR project* OR practice* OR instrument* OR tool* OR framework* OR protocol* ORguid* OR scale* OR system* OR inventor* OR metric* OR template* OR profile* OR criteria OR questionnaire* OR refer* OR assess* OR "case plan*"OR "case formulat*" OR "management plan*" OR "treatment plan*" OR "support plan*" OR "case manage*" OR "risk manage*" OR progress* ORmonitor* OR supervis* OR measur* ) ) AND ( TITLE-ABS-KEY ( radicali* OR extremis* OR terroris* OR jihadi* OR islamis* OR salafi* OR right-wingOR "right wing" OR extreme-right OR "extreme right" OR neo-nazi OR far-right OR "far right" OR nationalist* OR "white supremacis*" OR left-wing OR"left wing" OR extreme-left OR "extreme left" OR anarch* OR "single issue" OR single-issue ) ) AND ( LIMIT-TO ( PUBYEAR , 2022 ) ) AND ( EXCLUDE (SUBJAREA , "envi" ) OR EXCLUDE ( SUBJAREA , "bioc" ) OR EXCLUDE ( SUBJAREA , "phys" ) OR EXCLUDE ( SUBJAREA , "eart" ) OR EXCLUDE (SUBJAREA , "chem" ) OR EXCLUDE ( SUBJAREA , "ener" ) OR EXCLUDE ( SUBJAREA , "mate" ) OR EXCLUDE ( SUBJAREA , "agri" ) OR EXCLUDE (SUBJAREA , "ceng" ) OR EXCLUDE ( SUBJAREA , "phar" ) OR EXCLUDE ( SUBJAREA , "immu" ) ) AND ( EXCLUDE ( DOCTYPE , "no" ) OR EXCLUDE (DOCTYPE , "ed" ) OR EXCLUDE ( DOCTYPE , "cr" ) OR EXCLUDE ( DOCTYPE , "le" ) ) | 796 |
| ( TITLE-ABS-KEY ( prevent* OR reduc* OR counter* OR disengage* OR rehab* OR reintegrat* OR re-integrat* OR re-entry OR reentry OR desist* ORrecidivism OR deradical* OR de-radical* OR exit* ) ) AND ( TITLE-ABS-KEY ( initiative* OR interven* OR program* OR policy OR policies OR scheme*OR treat* OR approach* OR model* OR strateg* OR method* OR project* OR practice* OR instrument* OR tool* OR framework* OR protocol* ORguid* OR scale* OR system* OR inventor* OR metric* OR template* OR profile* OR criteria OR questionnaire* OR refer* OR assess* OR "case plan*"OR "case formulat*" OR "management plan*" OR "treatment plan*" OR "support plan*" OR "case manage*" OR "risk manage*" OR progress* ORmonitor* OR supervis* OR measur* ) ) AND ( TITLE-ABS-KEY ( radicali* OR extremis* OR terroris* OR jihadi* OR islamis* OR salafi* OR right-wingOR "right wing" OR extreme-right OR "extreme right" OR neo-nazi OR far-right OR "far right" OR nationalist* OR "white supremacis*" OR left-wing OR"left wing" OR extreme-left OR "extreme left" OR anarch* OR "single issue" OR single-issue ) ) AND ( LIMIT-TO ( PUBYEAR , 2022 ) ) AND ( EXCLUDE (SUBJAREA , "envi" ) OR EXCLUDE ( SUBJAREA , "bioc" ) OR EXCLUDE ( SUBJAREA , "phys" ) OR EXCLUDE ( SUBJAREA , "eart" ) OR EXCLUDE (SUBJAREA , "chem" ) OR EXCLUDE ( SUBJAREA , "ener" ) OR EXCLUDE ( SUBJAREA , "mate" ) OR EXCLUDE ( SUBJAREA , "agri" ) OR EXCLUDE (SUBJAREA , "ceng" ) OR EXCLUDE ( SUBJAREA , "phar" ) OR EXCLUDE ( SUBJAREA , "immu" ) ) | 807 |
| ( TITLE-ABS-KEY ( prevent* OR reduc* OR counter* OR disengage* OR rehab* OR reintegrat* OR re-integrat* OR re-entry OR reentry OR desist* ORrecidivism OR deradical* OR de-radical* OR exit* ) ) AND ( TITLE-ABS-KEY ( initiative* OR interven* OR program* OR policy OR policies OR scheme*OR treat* OR approach* OR model* OR strateg* OR method* OR project* OR practice* OR instrument* OR tool* OR framework* OR protocol* ORguid* OR scale* OR system* OR inventor* OR metric* OR template* OR profile* OR criteria OR questionnaire* OR refer* OR assess* OR "case plan*"OR "case formulat*" OR "management plan*" OR "treatment plan*" OR "support plan*" OR "case manage*" OR "risk manage*" OR progress* ORmonitor* OR supervis* OR measur* ) ) AND ( TITLE-ABS-KEY ( radicali* OR extremis* OR terroris* OR jihadi* OR islamis* OR salafi* OR right-wingOR "right wing" OR extreme-right OR "extreme right" OR neo-nazi OR far-right OR "far right" OR nationalist* OR "white supremacis*" OR left-wing OR"left wing" OR extreme-left OR "extreme left" OR anarch* OR "single issue" OR single-issue ) ) AND ( LIMIT-TO ( PUBYEAR , 2022 ) ) | 910 |

**Table A1.4(a). CINCH: Australian Criminology Database Search Records – Part I**

| [All Fields: radicali* OR All Fields: extremis* OR All Fields: terroris* OR All Fields: jihadi* OR All Fields: islamis* OR All Fields: salafi* OR All Fields: right-wing OR All Fields: 'right wing' OR All Fields: extreme-right OR All Fields: 'extreme right' OR All Fields: neo-nazi OR All Fields: far-right OR All Fields: 'far right' OR All Fields: nationalist* OR All Fields: 'white supremacis*' OR All Fields: left-wing OR All Fields: 'left wing' OR All Fields: extreme-left OR All Fields: 'extreme left' OR All Fields: anarch* OR All Fields: 'single issue' OR All Fields: single-issue] AND [All Fields: initiative* OR All Fields: interven* OR All Fields: program* OR All Fields: policy OR All Fields: policies OR All Fields: scheme* OR All Fields: treat* OR All Fields: approach* OR All Fields: model* OR All Fields: strateg* OR All Fields: method* OR All Fields: project* OR All Fields: practice* OR All Fields: instrument* OR All Fields: tool* OR All Fields: framework* OR All Fields: protocol* OR All Fields: guid* OR All Fields: scale* OR All Fields: system* OR All Fields: inventor* OR All Fields: metric* OR All Fields: template* OR All Fields: profile* OR All Fields: criteria OR All Fields: questionnaire* OR All Fields: refer* OR All Fields: assess* OR All Fields: 'case plan*' OR All Fields: 'case formulat*' OR All Fields: 'management plan*' OR All Fields: 'treatment plan*' OR All Fields: 'support plan*' OR All Fields: 'case manage*' OR All Fields: 'risk manage*' OR All Fields: progress* OR All Fields: monitor* OR All Fields: supervis* OR All Fields: measur*] AND [All Fields: prevent* OR All Fields: reduc* OR All Fields: counter* OR All Fields: disengage* OR All Fields: rehab* OR All Fields: reintegrat* OR All Fields: re-integrat* OR All Fields: re-entry OR All Fields: reentry OR All Fields: desist* OR All Fields: recidivism OR All Fields: deradical* OR All Fields: de-radical* OR All Fields: exit*] AND Publication Date: (01/01/2000 TO 31/12/2022) | 549 |
| --- | --- |

**Table A1.5(a). Medline Search Records – Part I**

| **1** | (radicali* or extremis* or terroris* or jihadi* or islamis* or salafi* or right-wing or "right wing" or extreme-right or "extreme right" or neo-nazi or far-right or "far right" or Nationalist* or "white supremacis*" or left-wing or "left wing" or extreme-left or "extreme left" or anarch* or "single issue" or single-issue).ab,hw,kf,kw,ot,sh,sy,ti,fx. | 16733 |
| --- | --- | --- |
| **2** | (initiative* or interven* or program* or policy or policies or scheme* or treat* or approach* or model* or strateg* or method* or project* or practice* or instrument* or tool* or framework* or protocol* or guid* or scale* or system* or inventor* or metric* or template* or profile* or criteria or questionnaire* or refer* or assess* or "case plan*" or "case formulat*" or "management plan*" or "treatment plan*" or "support plan*" or "case manage*" or "risk manage*" or progress* or monitor* or supervis* or measur*).ab,hw,kf,ot,sh,sy,ti,fx. | 22466030 |
| **3** | (prevent* or reduc* or counter* or disengage* or rehab* or reintegrat* or re-integrat* or re-entry or reentry or desist* or recidivism or deradical* or de-radical* or exit*).ab,hw,kf,ot,sh,sy,ti,fx. | 6645913 |
| **4** | 1 and 2 and 3 | 4114 |
| **5** | limit 4 to (humans and yr="2000 - 2022") | 2922 |

**Table A1.6(a). PsycInfo Search Records – Part I**

| **1** | (radicali* or extremis* or terroris* or jihadi* or islamis* or salafi* or right-wing or "right wing" or extreme-right or "extreme right" or neo-nazi or far-right or "far right" or Nationalist* or "white supremacis*" or left-wing or "left wing" or extreme-left or "extreme left" or anarch* or "single issue" or single-issue).ab,hw,id,mh,ot,ti. | 19229 |
| --- | --- | --- |
| **2** | (initiative* or interven* or program* or policy or policies or scheme* or treat* or approach* or model* or strateg* or method* or project* or practice* or instrument* or tool* or framework* or protocol* or guid* or scale* or system* or inventor* or metric* or template* or profile* or criteria or questionnaire* or refer* or assess* or "case plan*" or "case formulat*" or "management plan*" or "treatment plan*" or "support plan*" or "case manage*" or "risk manage*" or progress* or monitor* or supervis* or measur*).ab,hw,id,mh,ot,ti. | 4146675 |
| **3** | (prevent* or reduc* or counter* or disengage* or rehab* or reintegrat* or re-integrat* or re-entry or reentry or desist* or recidivism or deradical* or de-radical* or exit*).ab,hw,id,mh,ot,ti. | 855662 |
| **4** | 1 and 2 and 3 | 3324 |
| **5** | limit 4 to yr="2000 - 2022" | 3168 |

**Table A1.7(a). Dissertations and Theses Global – Part I**

| Set# | Searched for | Databases | Results |
| --- | --- | --- | --- |
| S1 | ti(radicali* OR extremis* OR terroris* OR jihadi* OR islamis* OR salafi* OR right-wing OR "right wing" OR extreme-right OR "extreme right" OR neo-nazi OR far-right OR "far right" OR Nationalist* OR "white supremacis*" OR left-wing OR "left wing" OR extreme-left OR "extreme left" OR anarch* OR "single issue" OR single-issue) OR ab(radicali* OR extremis* OR terroris* OR jihadi* OR islamis* OR salafi* OR right-wing OR "right wing" OR extreme-right OR "extreme right" OR neo-nazi OR far-right OR "far right" OR Nationalist* OR "white supremacis*" OR left-wing OR "left wing" OR extreme-left OR "extreme left" OR anarch* OR "single issue" OR single-issue) OR mainsubject(radicali* OR extremis* OR terroris* OR jihadi* OR islamis* OR salafi* OR right-wing OR "right wing" OR extreme-right OR "extreme right" OR neo-nazi OR far-right OR "far right" OR Nationalist* OR "white supremacis*" OR left-wing OR "left wing" OR extreme-left OR "extreme left" OR anarch* OR "single issue" OR single-issue) OR diskw(radicali* OR extremis* OR terroris* OR jihadi* OR islamis* OR salafi* OR right-wing OR "right wing" OR extreme-right OR "extreme right" OR neo-nazi OR far-right OR "far right" OR Nationalist* OR "white supremacis*" OR left-wing OR "left wing" OR extreme-left OR "extreme left" OR anarch* OR "single issue" OR single-issue) | ProQuest Dissertations & Theses Global | 28510 |
| S2 | ti(initiative* OR interven* OR program* OR policy OR policies OR scheme* OR treat* OR approach* OR model* OR strateg* OR method* OR project* OR practice* OR instrument* OR tool* OR framework* OR protocol* OR guid* OR scale* OR system* OR inventor* OR metric* OR template* OR profile* OR criteria OR questionnaire* OR refer* OR assess* OR "case plan*" OR "case formulat*" OR "management plan*" OR "treatment plan*" OR "support plan*" OR "case manage*" OR "risk manage*" OR progress* OR monitor* OR supervis* OR measur*) OR ab(initiative* OR interven* OR program* OR policy OR policies OR scheme* OR treat* OR approach* OR model* OR strateg* OR method* OR project* OR practice* OR instrument* OR tool* OR framework* OR protocol* OR guid* OR scale* OR system* OR inventor* OR metric* OR template* OR profile* OR criteria OR questionnaire* OR refer* OR assess* OR "case plan*" OR "case formulat*" OR "management plan*" OR "treatment plan*" OR "support plan*" OR "case manage*" OR "risk manage*" OR progress* OR monitor* OR supervis* OR measur*) OR mainsubject(initiative* OR interven* OR program* OR policy OR policies OR scheme* OR treat* OR approach* OR model* OR strateg* OR method* OR project* OR practice* OR instrument* OR tool* OR framework* OR protocol* OR guid* OR scale* OR system* OR inventor* OR metric* OR template* OR profile* OR criteria OR questionnaire* OR refer* OR assess* OR "case plan*" OR "case formulat*" OR "management plan*" OR "treatment plan*" OR "support plan*" OR "case manage*" OR "risk manage*" OR progress* OR monitor* OR supervis* OR measur*) OR diskw(initiative* OR interven* OR program* OR policy OR policies OR scheme* OR treat* OR approach* OR model* OR strateg* OR method* OR project* OR practice* OR instrument* OR tool* OR framework* OR protocol* OR guid* OR scale* OR system* OR inventor* OR metric* OR template* OR profile* OR criteria OR questionnaire* OR refer* OR assess* OR "case plan*" OR "case formulat*" OR "management plan*" OR "treatment plan*" OR "support plan*" OR "case manage*" OR "risk manage*" OR progress* OR monitor* OR supervis* OR measur*) | ProQuest Dissertations & Theses Global | 3769329 |
| S3 | ti(prevent* OR reduc* OR counter* OR disengage* OR rehab* OR reintegrat* OR re-integrat* OR re-entry OR reentry OR desist* OR recidivism OR deradical* OR de-radical* OR exit*) OR ab(prevent* OR reduc* OR counter* OR disengage* OR rehab* OR reintegrat* OR re-integrat* OR re-entry OR reentry OR desist* OR recidivism OR deradical* OR de-radical* OR exit*) OR mainsubject(prevent* OR reduc* OR counter* OR disengage* OR rehab* OR reintegrat* OR re-integrat* OR re-entry OR reentry OR desist* OR recidivism OR deradical* OR de-radical* OR exit*) OR diskw(prevent* OR reduc* OR counter* OR disengage* OR rehab* OR reintegrat* OR re-integrat* OR re-entry OR reentry OR desist* OR recidivism OR deradical* OR de-radical* OR exit*) | ProQuest Dissertations & Theses Global | 847069 |
| S4 | S1 AND S2 AND S3 | ProQuest Dissertations & Theses Global | 4473 |
| S5 | (S1 AND S2 AND S3) AND pd(20000101-20220807) | ProQuest Dissertations & Theses Global | 3618 |

**Table A1.8(a). International Bibliography of the Social Sciences – Part I**

| Set# | Searched for | Databases | Results |
| --- | --- | --- | --- |
| S1 | ti(radicali* OR extremis* OR terroris* OR jihadi* OR islamis* OR salafi* OR right-wing OR "right wing" OR extreme-right OR "extreme right" OR neo-nazi OR far-right OR "far right" OR Nationalist* OR "white supremacis*" OR left-wing OR "left wing" OR extreme-left OR "extreme left" OR anarch* OR "single issue" OR single-issue) OR ab(radicali* OR extremis* OR terroris* OR jihadi* OR islamis* OR salafi* OR right-wing OR "right wing" OR extreme-right OR "extreme right" OR neo-nazi OR far-right OR "far right" OR Nationalist* OR "white supremacis*" OR left-wing OR "left wing" OR extreme-left OR "extreme left" OR anarch* OR "single issue" OR single-issue) OR mainsubject(radicali* OR extremis* OR terroris* OR jihadi* OR islamis* OR salafi* OR right-wing OR "right wing" OR extreme-right OR "extreme right" OR neo-nazi OR far-right OR "far right" OR Nationalist* OR "white supremacis*" OR left-wing OR "left wing" OR extreme-left OR "extreme left" OR anarch* OR "single issue" OR single-issue) | International Bibliography of the Social Sciences (IBSS) | 101122 |
| S2 | ti(initiative* OR interven* OR program* OR policy OR policies OR scheme* OR treat* OR approach* OR model* OR strateg* OR method* OR project* OR practice* OR instrument* OR tool* OR framework* OR protocol* OR guid* OR scale* OR system* OR inventor* OR metric* OR template* OR profile* OR criteria OR questionnaire* OR refer* OR assess* OR "case plan*" OR "case formulat*" OR "management plan*" OR "treatment plan*" OR "support plan*" OR "case manage*" OR "risk manage*" OR progress* OR monitor* OR supervis* OR measur*) OR ab(initiative* OR interven* OR program* OR policy OR policies OR scheme* OR treat* OR approach* OR model* OR strateg* OR method* OR project* OR practice* OR instrument* OR tool* OR framework* OR protocol* OR guid* OR scale* OR system* OR inventor* OR metric* OR template* OR profile* OR criteria OR questionnaire* OR refer* OR assess* OR "case plan*" OR "case formulat*" OR "management plan*" OR "treatment plan*" OR "support plan*" OR "case manage*" OR "risk manage*" OR progress* OR monitor* OR supervis* OR measur*) OR mainsubject(initiative* OR interven* OR program* OR policy OR policies OR scheme* OR treat* OR approach* OR model* OR strateg* OR method* OR project* OR practice* OR instrument* OR tool* OR framework* OR protocol* OR guid* OR scale* OR system* OR inventor* OR metric* OR template* OR profile* OR criteria OR questionnaire* OR refer* OR assess* OR "case plan*" OR "case formulat*" OR "management plan*" OR "treatment plan*" OR "support plan*" OR "case manage*" OR "risk manage*" OR progress* OR monitor* OR supervis* OR measur*) | International Bibliography of the Social Sciences (IBSS) | 2709404 |
| S3 | ti(prevent* OR reduc* OR counter* OR disengage* OR rehab* OR reintegrat* OR re-integrat* OR re-entry OR reentry OR desist* OR recidivism OR deradical* OR de-radical* OR exit*) OR ab(prevent* OR reduc* OR counter* OR disengage* OR rehab* OR reintegrat* OR re-integrat* OR re-entry OR reentry OR desist* OR recidivism OR deradical* OR de-radical* OR exit*) OR mainsubject(prevent* OR reduc* OR counter* OR disengage* OR rehab* OR reintegrat* OR re-integrat* OR re-entry OR reentry OR desist* OR recidivism OR deradical* OR de-radical* OR exit*) | International Bibliography of the Social Sciences (IBSS) | 330404 |
| S4 | S1 AND S2 AND S3 | International Bibliography of the Social Sciences (IBSS) | 8395 |
| S5 | (S1 AND S2 AND S3) AND pd(20000101-20220807) | International Bibliography of the Social Sciences (IBSS) | 8163 |
| S6 | (S1 AND S2 AND S3) NOT (at.exact("General Information" OR "News" OR "Editorial" OR "Correspondence" OR "Credit/Acknowledgement" OR "Editorial Cartoon/Comic" OR "Letter to the Editor") AND pd(20000101-20220807)) | International Bibliography of the Social Sciences (IBSS) | 7967 |
| S7 | (S1 AND S2 AND S3) NOT (at.exact("General Information" OR "News" OR "Editorial" OR "Correspondence" OR "Credit/Acknowledgement" OR "Editorial Cartoon/Comic" OR "Letter to the Editor") NOT stype.exact("Newspapers") AND pd(20000101-20220807)) | International Bibliography of the Social Sciences (IBSS) | 7940 |

**Table A1.9(a). Sociological Abstracts – Part I**

| Set# | Searched for | Databases | Results |
| --- | --- | --- | --- |
| S1 | ti(radicali* OR extremis* OR terroris* OR jihadi* OR islamis* OR salafi* OR right-wing OR "right wing" OR extreme-right OR "extreme right" OR neo-nazi OR far-right OR "far right" OR Nationalist* OR "white supremacis*" OR left-wing OR "left wing" OR extreme-left OR "extreme left" OR anarch* OR "single issue" OR single-issue) OR ab(radicali* OR extremis* OR terroris* OR jihadi* OR islamis* OR salafi* OR right-wing OR "right wing" OR extreme-right OR "extreme right" OR neo-nazi OR far-right OR "far right" OR Nationalist* OR "white supremacis*" OR left-wing OR "left wing" OR extreme-left OR "extreme left" OR anarch* OR "single issue" OR single-issue) OR mainsubject(radicali* OR extremis* OR terroris* OR jihadi* OR islamis* OR salafi* OR right-wing OR "right wing" OR extreme-right OR "extreme right" OR neo-nazi OR far-right OR "far right" OR Nationalist* OR "white supremacis*" OR left-wing OR "left wing" OR extreme-left OR "extreme left" OR anarch* OR "single issue" OR single-issue) OR if(radicali* OR extremis* OR terroris* OR jihadi* OR islamis* OR salafi* OR right-wing OR "right wing" OR extreme-right OR "extreme right" OR neo-nazi OR far-right OR "far right" OR Nationalist* OR "white supremacis*" OR left-wing OR "left wing" OR extreme-left OR "extreme left" OR anarch* OR "single issue" OR single-issue) | Sociological Abstracts | 49523 |
| S2 | ti(initiative* OR interven* OR program* OR policy OR policies OR scheme* OR treat* OR approach* OR model* OR strateg* OR method* OR project* OR practice* OR instrument* OR tool* OR framework* OR protocol* OR guid* OR scale* OR system* OR inventor* OR metric* OR template* OR profile* OR criteria OR questionnaire* OR refer* OR assess* OR "case plan*" OR "case formulat*" OR "management plan*" OR "treatment plan*" OR "support plan*" OR "case manage*" OR "risk manage*" OR progress* OR monitor* OR supervis* OR measur*) OR ab(initiative* OR interven* OR program* OR policy OR policies OR scheme* OR treat* OR approach* OR model* OR strateg* OR method* OR project* OR practice* OR instrument* OR tool* OR framework* OR protocol* OR guid* OR scale* OR system* OR inventor* OR metric* OR template* OR profile* OR criteria OR questionnaire* OR refer* OR assess* OR "case plan*" OR "case formulat*" OR "management plan*" OR "treatment plan*" OR "support plan*" OR "case manage*" OR "risk manage*" OR progress* OR monitor* OR supervis* OR measur*) OR mainsubject(initiative* OR interven* OR program* OR policy OR policies OR scheme* OR treat* OR approach* OR model* OR strateg* OR method* OR project* OR practice* OR instrument* OR tool* OR framework* OR protocol* OR guid* OR scale* OR system* OR inventor* OR metric* OR template* OR profile* OR criteria OR questionnaire* OR refer* OR assess* OR "case plan*" OR "case formulat*" OR "management plan*" OR "treatment plan*" OR "support plan*" OR "case manage*" OR "risk manage*" OR progress* OR monitor* OR supervis* OR measur*) OR if(initiative* OR interven* OR program* OR policy OR policies OR scheme* OR treat* OR approach* OR model* OR strateg* OR method* OR project* OR practice* OR instrument* OR tool* OR framework* OR protocol* OR guid* OR scale* OR system* OR inventor* OR metric* OR template* OR profile* OR criteria OR questionnaire* OR refer* OR assess* OR "case plan*" OR "case formulat*" OR "management plan*" OR "treatment plan*" OR "support plan*" OR "case manage*" OR "risk manage*" OR progress* OR monitor* OR supervis* OR measur*) | Sociological Abstracts | 1549447 |
| S3 | ti(prevent* OR reduc* OR counter* OR disengage* OR rehab* OR reintegrat* OR re-integrat* OR re-entry OR reentry OR desist* OR recidivism OR deradical* OR de-radical* OR exit*) OR ab(prevent* OR reduc* OR counter* OR disengage* OR rehab* OR reintegrat* OR re-integrat* OR re-entry OR reentry OR desist* OR recidivism OR deradical* OR de-radical* OR exit*) OR mainsubject(prevent* OR reduc* OR counter* OR disengage* OR rehab* OR reintegrat* OR re-integrat* OR re-entry OR reentry OR desist* OR recidivism OR deradical* OR de-radical* OR exit*) OR if(prevent* OR reduc* OR counter* OR disengage* OR rehab* OR reintegrat* OR re-integrat* OR re-entry OR reentry OR desist* OR recidivism OR deradical* OR de-radical* OR exit*) | Sociological Abstracts | 228973 |
| S4 | S1 AND S2 AND S3 | Sociological Abstracts | 4423 |
| S5 | (S1 AND S2 AND S3) AND pd(20000101-20220807) | Sociological Abstracts | 3533 |
| S6 | (S1 AND S2 AND S3) NOT (at.exact("General Information" OR "Editorial" OR "News" OR "Correspondence") AND pd(20000101-20220807)) | Sociological Abstracts | 3482 |

**Table A1.10(a). Web of Science Searches – Part I**

| #4 | Search  #3 AND #2 AND #1 and Letter or Book Review or Editorial Material or Chronology (Exclude – Document Types) and Urology Nephrology or Thermodynamics or Physics Fluids Plasmas or Oceanography or Materials Science Ceramics or Marine Freshwater Biology or Forestry or Engineering Ocean or Engineering Marine or Biochemistry Molecular Biology or Biodiversity Conservation or Biochemical Research Methods or Immunology or Engineering Geological or Biophysics or Allergy or Agriculture Dairy Animal Science or Infectious Diseases or Dermatology or Peripheral Vascular Disease or Orthopedics or Optics or Ophthalmology or Chemistry Analytical or Cell Biology or Respiratory System or Physics Nuclear or Cardiac Cardiovascular Systems or Biotechnology Applied Microbiology or Oncology or Engineering Aerospace or Anesthesiology or Agriculture Multidisciplinary or Pharmacology Pharmacy or Engineering Electrical Electronic or Literature Romance or Energy Fuels or Water Resources or Obstetrics Gynecology or Meteorology Atmospheric Sciences or Nuclear Science Technology or Surgery or Engineering Environmental or Construction Building Technology or Radiology Nuclear Medicine Medical Imaging or Toxicology or Geography Physical or Agricultural Economics Policy or Ecology or Engineering Chemical or Food Science Technology or Green Sustainable Science Technology or Engineering Civil or Engineering Industrial or Geography or Environmental Studies (Exclude – Web of Science Categories) and Nutrition Dietetics or Pathology or Geology or Materials Science or Physiology or Chemistry or Gastroenterology Hepatology or Architecture or Engineering (Exclude – Research Areas)  5:03 PM | Exact search  Web of Science Core Collection Hide editions  Editions = A&HCI , BKCI-SSH , ESCI ,CPCI-SSH , SSCI | 8,125 |
| --- | --- | --- |
| #3 | Search TS=(prevent* OR reduc* OR counter* OR disengage* OR rehab* OR reintegrat* OR re-integrat* OR re-entry OR reentry OR desist* OR recidivism OR deradical* OR de-radical* OR exit*)  4:57 PM | Timespan: 2000-01-01 to 2022-12-31 (Publication Date) | Exact search  Web of Science Core Collection Hide editions  Editions = A&HCI , BKCI-SSH , ESCI ,CPCI-SSH , SSCI | 1,209,712 |
| #2 | Search  TS=(initiative* OR interven* OR program* OR policy OR policies OR scheme* OR treat* OR approach* OR model* OR strateg* OR method* OR project* OR practice* OR instrument* OR tool* OR framework* OR protocol* OR guid* OR scale* OR system* OR inventor* OR metric* OR template* OR profile* OR criteria OR questionnaire* OR refer* OR assess* OR "case plan*" OR "case formulat*" OR "management plan*" OR "treatment plan*" OR "support plan*" OR "case manage*" OR "risk manage*" OR progress* OR monitor* OR supervis* OR measur*)  4:56 PM | Timespan: 2000-01-01 to 2022-12-31 (Publication Date) | Exact search  Web of Science Core Collection Hide editions  Editions = A&HCI , BKCI-SSH , ESCI ,CPCI-SSH , SSCI | 5,962,903 |
| #1 | Search  TS=(radicali* OR extremis* OR terroris* OR jihadi* OR islamis* OR salafi* OR right-wing OR "right wing" OR extreme-right OR "extreme right" OR neo-nazi OR far-right OR "far right" OR Nationalist* OR "white supremacis*" OR left-wing OR "left wing" OR extreme-left OR "extreme left" OR anarch* OR "single issue" OR single-issue)  4:56 PM | Timespan: 2000-01-01 to 2022-12-31 (Publication Date) | Exact search  Web of Science Core Collection Hide editions  Editions = A&HCI , BKCI-SSH , ESCI ,CPCI-SSH , SSCI | 69,200 |

**Appendix I (B). Part I Search Record (LOE)[[1]](#footnote-1)**

**Table A1.1(b) Academic Platform Search Record – Part I (LOE)**

| **Search Source** | **Source** | **Search Date)** | **Date Coverage for Search** | **Results** |
| --- | --- | --- | --- | --- |
| Criminal Justice Abstracts | EBSCO | 27/09/2022 | 01/01/2000 - 27/09/2022 | 490 |
| Scopus | Elsevier | 27/09/2022 | 01/01/2000 - 27/09/2022 | 7790 |
| Medline | Ovid | 27/09/2022 | 01/01/2000 - 27/09/2022 | 283* |
| PsycInfo | Ovid | 27/09/2022 | 01/01/2000 - 27/09/2022 | 113* |
| Dissertations and Theses Global | ProQuest | 27/09/2022 | 01/01/2000 - 27/09/2022 | 426* |
| International Bibliography of the Social Sciences | ProQuest | 27/09/2022 | 01/01/2000 - 27/09/2022 | 569* |
| Sociological Abstracts | ProQuest | 27/09/2022 | 01/01/2000 - 27/09/2022 | 304* |
| Book Citation Index – Social Sciences & Humanities (BKCI-SSH)  Social Sciences Citation Index (SSCI)  Arts & Humanities Citation Index (A&HCI)  Emerging Sources Citation Index (ESCI)  Conference Proceedings Citation Index – Social Science & Humanities (CPCI-SSH) | Web of Science | 27/09/2022 | 01/01/2000 - 27/09/2022 | 4863 |

* Unable to search LOE keywords due to unsupported characters.

**Table A1.2(b). Criminal Justice Abstracts Search Records – Part I (LOE)**

English Keywords

| **#** | **Query** | **Limiters/Expanders** | **Last Run Via** | **Results** |
| --- | --- | --- | --- | --- |
| S5 | S1 AND S2 AND S3 | Narrow by Language: - Norwegian  Macedonian  Lithuanian  Bosnian  Danish  Italian  Croatian  Russian  Turkish  Dutch/Flemish  Spanish  Slovenian  French  Romanian  German  Search modes - Boolean/Phrase | Interface - EBSCOhost Research Databases  Search Screen - Advanced Search  Database - Criminal Justice Abstracts | 124 |
| S4 | S1 AND S2 AND S3 | Search modes - Boolean/Phrase | Interface - EBSCOhost Research Databases | 3,850 |
|  |  |  | Search Screen - Advanced Search |  |
|  |  |  | Database - Criminal Justice Abstracts |  |
| S3 | TI ( prevent* OR reduc* OR counter* OR disengage* OR rehab* OR reintegrat* OR re-integrat* OR re-entry OR reentry OR desist* OR recidivism OR deradical* OR de-radical* OR exit* ) OR AB ( prevent* OR reduc* OR counter* OR disengage* OR rehab* OR reintegrat* OR re-integrat* OR re-entry OR reentry OR desist* OR recidivism OR deradical* OR de-radical* OR exit* ) OR KW ( prevent* OR reduc* OR counter* OR disengage* OR rehab* OR reintegrat* OR re-integrat* OR re-entry OR reentry OR desist* OR recidivism OR deradical* OR de-radical* OR exit* ) OR SU ( prevent* OR reduc* OR counter* OR disengage* OR rehab* OR reintegrat* OR re-integrat* OR re-entry OR reentry OR desist* OR recidivism OR deradical* OR de-radical* OR exit* ) | Limiters - Publication Date: 20000101-20221231 | Interface - EBSCOhost Research Databases | 111,950 |
|  |  | Search modes - Boolean/Phrase | Search Screen - Advanced Search |  |
|  |  |  | Database - Criminal Justice Abstracts |  |
| S2 | TI ( initiative* OR interven* OR program* OR policy OR policies OR scheme* OR treat* OR approach* OR model* OR strateg* OR method* OR project* OR practice* OR instrument* OR tool* OR framework* OR protocol* OR guid* OR scale* OR system* OR inventor* OR metric* OR template* OR profile* OR criteria OR questionnaire* OR refer* OR assess* OR "case plan*" OR "case formulat*" OR "management plan*" OR "treatment plan*" OR "support plan*" OR "case manage*" OR "risk manage*" OR progress* OR monitor* OR supervis* OR measur* ) OR AB ( initiative* OR interven* OR program* OR policy OR policies OR scheme* OR treat* OR approach* OR model* OR strateg* OR method* OR project* OR practice* OR instrument* OR tool* OR framework* OR protocol* OR guid* OR scale* OR system* OR inventor* OR metric* OR template* OR profile* OR criteria OR questionnaire* OR refer* OR assess* OR "case plan*" OR "case formulat*" OR "management plan*" OR "treatment plan*" OR "support plan*" OR "case manage*" OR "risk manage*" OR progress* OR monitor* OR supervis* OR measur* ) OR KW ( initiative* OR interven* OR program* OR policy OR policies OR scheme* OR treat* OR approach* OR model* OR strateg* OR method* OR project* OR practice* OR instrument* OR tool* OR framework* OR protocol* OR guid* OR scale* OR system* OR inventor* OR metric* OR template* OR profile* OR criteria OR questionnaire* OR refer* OR assess* OR "case plan*" OR "case formulat*" OR "management plan*" OR "treatment plan*" OR "support plan*" OR "case manage*" OR "risk manage*" OR progress* OR monitor* OR supervis* OR measur* ) OR SU ( initiative* OR interven* OR program* OR policy OR policies OR scheme* OR treat* OR approach* OR model* OR strateg* OR method* OR project* OR practice* OR instrument* OR tool* OR framework* OR protocol* OR guid* OR scale* OR system* OR inventor* OR metric* OR template* OR profile* OR criteria OR questionnaire* OR refer* OR assess* OR "case plan*" OR "case formulat*" OR "management plan*" OR "treatment plan*" OR "support plan*" OR "case manage*" OR "risk manage*" OR progress* OR monitor* OR supervis* OR measur* ) | Limiters - Publication Date: 20000101-20221231 | Interface - EBSCOhost Research Databases | 363,031 |
|  |  | Search modes - Boolean/Phrase | Search Screen - Advanced Search |  |
|  |  |  | Database - Criminal Justice Abstracts |  |
| S1 | TI ( radicali* OR extremis* OR terroris* OR jihadi* OR islamis* OR salafi* OR right-wing OR "right wing" OR extreme-right OR "extreme right" OR neo-nazi OR far-right OR "far right" OR Nationalist* OR "white supremacis*" OR left-wing OR "left wing" OR extreme-left OR "extreme left" OR anarch* OR "single issue" OR single-issue ) OR AB ( radicali* OR extremis* OR terroris* OR jihadi* OR islamis* OR salafi* OR right-wing OR "right wing" OR extreme-right OR "extreme right" OR neo-nazi OR far-right OR "far right" OR Nationalist* OR "white supremacis*" OR left-wing OR "left wing" OR extreme-left OR "extreme left" OR anarch* OR "single issue" OR single-issue ) OR KW ( radicali* OR extremis* OR terroris* OR jihadi* OR islamis* OR salafi* OR right-wing OR "right wing" OR extreme-right OR "extreme right" OR neo-nazi OR far-right OR "far right" OR Nationalist* OR "white supremacis*" OR left-wing OR "left wing" OR extreme-left OR "extreme left" OR anarch* OR "single issue" OR single-issue ) OR SU ( radicali* OR extremis* OR terroris* OR jihadi* OR islamis* OR salafi* OR right-wing OR "right wing" OR extreme-right OR "extreme right" OR neo-nazi OR far-right OR "far right" OR Nationalist* OR "white supremacis*" OR left-wing OR "left wing" OR extreme-left OR "extreme left" OR anarch* OR "single issue" OR single-issue ) | Limiters - Publication Date: 20000101-20221231 | Interface - EBSCOhost Research Databases | 14,483 |
|  |  | Search modes - Boolean/Phrase | Search Screen - Advanced Search |  |
|  |  |  | Database - Criminal Justice Abstracts |  |

French Keywords

| **#** | **Query** | **Limiters/Expanders** | **Last Run Via** | **Results** |
| --- | --- | --- | --- | --- |
| S5 | S1 AND S2 AND S3 | Expanders - Apply equivalent subjects  Narrow by Language: - Ukrainian  Norwegian  Romanian  German  Italian  Spanish  French  Search modes - Boolean/Phrase | Interface - EBSCOhost Research Databases  Search Screen - Advanced Search  Database - Criminal Justice Abstracts | 48 |
| S4 | S1 AND S2 AND S3 | Limiters - Publication Date: 20000101-20221231  Search modes - Boolean/Phrase | Interface - EBSCOhost Research Databases  Search Screen - Advanced Search  Database - Criminal Justice Abstracts | 535 |
| S3 | TI ( prévent* OR réduct* OR anti* OR désembriga* OR désendoctrin* OR désengage* OR réinser* OR ré-inser* OR récidiv* OR déradicalis* OR dé-radicalis* OR contre* OR réintégr* OR ré-intégr* OR renonc* OR désist* OR réhab* ) OR AB ( prévent* OR réduct* OR anti* OR désembriga* OR désendoctrin* OR désengage* OR réinser* OR ré-inser* OR récidiv* OR déradicalis* OR dé-radicalis* OR contre* OR réintégr* OR ré-intégr* OR renonc* OR désist* OR réhab* ) OR KW ( prévent* OR réduct* OR anti* OR désembriga* OR désendoctrin* OR désengage* OR réinser* OR ré-inser* OR récidiv* OR déradicalis* OR dé-radicalis* OR contre* OR réintégr* OR ré-intégr* OR renonc* OR désist* OR réhab* ) OR SU ( prévent* OR réduct* OR anti* OR désembriga* OR désendoctrin* OR désengage* OR réinser* OR ré-inser* OR récidiv* OR déradicalis* OR dé-radicalis* OR contre* OR réintégr* OR ré-intégr* OR renonc* OR désist* OR réhab* ) | Search modes - Boolean/Phrase | Interface - EBSCOhost Research Databases  Search Screen - Advanced Search  Database - Criminal Justice Abstracts | 27,813 |
|  |  |  |  |  |
|  |  |  |  |  |
| S2 | TI ( politique OR initiative* OR interven* OR progra* OR plan* OR traitement* OR approche* OR modèl* OR stratégi* OR méthod* OR projet* OR pratique* OR instrument* OR outil* OR cadre* OR protocole* OR guide* OR échelle* OR système* OR inventaire* OR métrique* gabarit* OR profile* OR critère* OR questionnaire* OR réfer* OR évalu* OR "plan d’intervention" OR "plan de traitement" OR "plan de gestion" OR "programme de gestion" OR "plan d’encadrement" OR "plan d’action" OR "programme de traitement" OR "plan de soins" OR "programme de soins" OR "projet de traitement" OR "plan thérapeutique" OR "programme thérapeutique" OR "plan de soutien" OR "plan d’accompagnement" OR "gestion de cas" OR "gestion de dossier" OR "gestion de risque*" OR "gestion des risques" OR progrès* OR évolution* OR progression* OR supervision* OR observation* OR surveill* OR suivi* OR mesure* ) OR AB ( politique OR initiative* OR interven* OR progra* OR plan* OR traitement* OR approche* OR modèl* OR stratégi* OR méthod* OR projet* OR pratique* OR instrument* OR outil* OR cadre* OR protocole* OR guide* OR échelle* OR système* OR inventaire* OR métrique* gabarit* OR profile* OR critère* OR questionnaire* OR réfer* OR évalu* OR "plan d’intervention" OR "plan de traitement" OR "plan de gestion" OR "programme de gestion" OR "plan d’encadrement" OR "plan d’action" OR "programme de traitement" OR "plan de soins" OR "programme de soins" OR "projet de traitement" OR "plan thérapeutique" OR "programme thérapeutique" OR "plan de soutien" OR "plan d’accompagnement" OR "gestion de cas" OR "gestion de dossier" OR "gestion de risque*" OR "gestion des risques" OR progrès* OR évolution* OR progression* OR supervision* OR observation* OR surveill* OR suivi* OR mesure* ) OR KW ( politique OR initiative* OR interven* OR progra* OR plan* OR traitement* OR approche* OR modèl* OR stratégi* OR méthod* OR projet* OR pratique* OR instrument* OR outil* OR cadre* OR protocole* OR guide* OR échelle* OR système* OR inventaire* OR métrique* gabarit* OR profile* OR critère* OR questionnaire* OR réfer* OR évalu* OR "plan d’intervention" OR "plan de traitement" OR "plan de gestion" OR "programme de gestion" OR "plan d’encadrement" OR "plan d’action" OR "programme de traitement" OR "plan de soins" OR "programme de soins" OR "projet de traitement" OR "plan thérapeutique" OR "programme thérapeutique" OR "plan de soutien" OR "plan d’accompagnement" OR "gestion de cas" OR "gestion de dossier" OR "gestion de risque*" OR "gestion des risques" OR progrès* OR évolution* OR progression* OR supervision* OR observation* OR surveill* OR suivi* OR mesure* ) OR SU ( politique OR initiative* OR interven* OR progra* OR plan* OR traitement* OR approche* OR modèl* OR stratégi* OR méthod* OR projet* OR pratique* OR instrument* OR outil* OR cadre* OR protocole* OR guide* OR échelle* OR système* OR inventaire* OR métrique* gabarit* OR profile* OR critère* OR questionnaire* OR réfer* OR évalu* OR "plan d’intervention" OR "plan de traitement" OR "plan de gestion" OR "programme de gestion" OR "plan d’encadrement" OR "plan d’action" OR "programme de traitement" OR "plan de soins" OR "programme de soins" OR "projet de traitement" OR "plan thérapeutique" OR "programme thérapeutique" OR "plan de soutien" OR "plan d’accompagnement" OR "gestion de cas" OR "gestion de dossier" OR "gestion de risque*" OR "gestion des risques" OR progrès* OR évolution* OR progression* OR supervision* OR observation* OR surveill* OR suivi* OR mesure* ) |  | Interface - EBSCOhost Research Databases  Search Screen - Advanced Search  Database - Criminal Justice Abstracts | 186,968 |
|  |  | Search modes - Boolean/Phrase |  |  |
|  |  |  |  |  |
| S1 | TI ( terroris* OR radical* OR extremis* OR djihadi* OR islamis* OR salafi* OR "droite extrémiste" OR "droite extrême" OR néo-nazi OR néonazisme OR "extrême droite" OR nationalis* OR "suprématis* blanc" OR "gauche extrémiste" OR "gauche extrême" OR "extrême gauche" OR anarch* ) OR AB ( terroris* OR radical* OR extremis* OR djihadi* OR islamis* OR salafi* OR "droite extrémiste" OR "droite extrême" OR néo-nazi OR néonazisme OR "extrême droite" OR nationalis* OR "suprématis* blanc" OR "gauche extrémiste" OR "gauche extrême" OR "extrême gauche" OR anarch* ) OR KW ( terroris* OR radical* OR extremis* OR djihadi* OR islamis* OR salafi* OR "droite extrémiste" OR "droite extrême" OR néo-nazi OR néonazisme OR "extrême droite" OR nationalis* OR "suprématis* blanc" OR "gauche extrémiste" OR "gauche extrême" OR "extrême gauche" OR anarch* ) OR SU ( terroris* OR radical* OR extremis* OR djihadi* OR islamis* OR salafi* OR "droite extrémiste" OR "droite extrême" OR néo-nazi OR néonazisme OR "extrême droite" OR nationalis* OR "suprématis* blanc" OR "gauche extrémiste" OR "gauche extrême" OR "extrême gauche" OR anarch* ) |  | Interface - EBSCOhost Research Databases  Search Screen - Advanced Search  Database - Criminal Justice Abstracts | 18,865 |
|  |  | Search modes - Boolean/Phrase |  |  |
|  |  |  |  |  |

German Key Words

| **#** | **Query** | **Limiters/Expanders** | **Last Run Via** | **Results** |
| --- | --- | --- | --- | --- |
| S5 | S1 AND S2 AND S3 | Expanders - Apply equivalent subjects  Narrow by Language: -  Russian  Lithuanian  Dutch/ Flemish  Spanish  Slovenian  French  German  Search modes - Boolean/Phrase | Interface - EBSCOhost Research Databases  Search Screen - Advanced Search  Database - Criminal Justice Abstracts | 36 |
| S4 | S1 AND S2 AND S3 | Limiters - Publication Date: 20000101-20221231  Search modes - Boolean/Phrase | Interface - EBSCOhost Research Databases  Search Screen - Advanced Search  Database - Criminal Justice Abstracts | 106 |
| S3 | TI ( verhinder* OR reduzier* OR minder* OR bekämpf* OR loslösen* OR rehab* OR resozialisier* OR reintegrat* OR wiedereinglieder* OR Abstrandn* OR unterlass* OR rückfall* OR Rückfäll* OR deradikal* OR aussteige* OR Aussstieg* ) OR AB ( verhinder* OR reduzier* OR minder* OR bekämpf* OR loslösen* OR rehab* OR resozialisier* OR reintegrat* OR wiedereinglieder* OR Abstrandn* OR unterlass* OR rückfall* OR Rückfäll* OR deradikal* OR aussteige* OR Aussstieg* ) OR KW ( verhinder* OR reduzier* OR minder* OR bekämpf* OR loslösen* OR rehab* OR resozialisier* OR reintegrat* OR wiedereinglieder* OR Abstrandn* OR unterlass* OR rückfall* OR Rückfäll* OR deradikal* OR aussteige* OR Aussstieg* ) OR SU ( verhinder* OR reduzier* OR minder* OR bekämpf* OR loslösen* OR rehab* OR resozialisier* OR reintegrat* OR wiedereinglieder* OR Abstrandn* OR unterlass* OR rückfall* OR Rückfäll* OR deradikal* OR aussteige* OR Aussstieg* ) | Expanders - Apply equivalent subjects  Search modes - Boolean/Phrase | Interface - EBSCOhost Research Databases  Search Screen - Advanced Search  Database - Criminal Justice Abstracts | 12,518 |
|  |  |  |  |  |
|  |  |  |  |  |
| S2 | TI ( initiative* OR interven* OR programm* OR schema* OR behand* OR Ansatz* OR modell* OR strateg* OR method* OR projekt* OR praxis* OR instrument* OR Werkzeug* OR Rahmen* OR protokoll* OR anleit* OR Handlungsempfehlung* OR Ausmaß* OR Maßstab* OR system* OR Erfinder* OR metri* OR Vorlage* OR Profil* OR Kriteri* OR Umfrage* OR Einliefer* OR Einweis* OR Beurteil* OR Einschätz* OR "Fallplan*" OR "Fallgestaltung*" OR "Managementplan*" OR "Behandlungsplan*" OR "Unterstützungsplan*" OR "Förderplan*" OR "Fallmanagement*" OR "Fallbearbeitung*" OR "Risikomanagement*" OR "Risikobewältigung*" OR Forschritt* OR Entwicklung* OR beobacht* OR überwach* OR beaufsichtig* OR Aufsicht* OR Aufseher* OR Maßnahme* OR Messen* ) OR AB ( initiative* OR interven* OR programm* OR schema* OR behand* OR Ansatz* OR modell* OR strateg* OR method* OR projekt* OR praxis* OR instrument* OR Werkzeug* OR Rahmen* OR protokoll* OR anleit* OR Handlungsempfehlung* OR Ausmaß* OR Maßstab* OR system* OR Erfinder* OR metri* OR Vorlage* OR Profil* OR Kriteri* OR Umfrage* OR Einliefer* OR Einweis* OR Beurteil* OR Einschätz* OR "Fallplan*" OR "Fallgestaltung*" OR "Managementplan*" OR "Behandlungsplan*" OR "Unterstützungsplan*" OR "Förderplan*" OR "Fallmanagement*" OR "Fallbearbeitung*" OR "Risikomanagement*" OR "Risikobewältigung*" OR Forschritt* OR Entwicklung* OR beobacht* OR überwach* OR beaufsichtig* OR Aufsicht* OR Aufseher* OR Maßnahme* OR Messen* ) OR KW ( initiative* OR interven* OR programm* OR schema* OR behand* OR Ansatz* OR modell* OR strateg* OR method* OR projekt* OR praxis* OR instrument* OR Werkzeug* OR Rahmen* OR protokoll* OR anleit* OR Handlungsempfehlung* OR Ausmaß* OR Maßstab* OR system* OR Erfinder* OR metri* OR Vorlage* OR Profil* OR Kriteri* OR Umfrage* OR Einliefer* OR Einweis* OR Beurteil* OR Einschätz* OR "Fallplan*" OR "Fallgestaltung*" OR "Managementplan*" OR "Behandlungsplan*" OR "Unterstützungsplan*" OR "Förderplan*" OR "Fallmanagement*" OR "Fallbearbeitung*" OR "Risikomanagement*" OR "Risikobewältigung*" OR Forschritt* OR Entwicklung* OR beobacht* OR überwach* OR beaufsichtig* OR Aufsicht* OR Aufseher* OR Maßnahme* OR Messen* ) OR SU ( initiative* OR interven* OR programm* OR schema* OR behand* OR Ansatz* OR modell* OR strateg* OR method* OR projekt* OR praxis* OR instrument* OR Werkzeug* OR Rahmen* OR protokoll* OR anleit* OR Handlungsempfehlung* OR Ausmaß* OR Maßstab* OR system* OR Erfinder* OR metri* OR Vorlage* OR Profil* OR Kriteri* OR Umfrage* OR Einliefer* OR Einweis* OR Beurteil* OR Einschätz* OR "Fallplan*" OR "Fallgestaltung*" OR "Managementplan*" OR "Behandlungsplan*" OR "Unterstützungsplan*" OR "Förderplan*" OR "Fallmanagement*" OR "Fallbearbeitung*" OR "Risikomanagement*" OR "Risikobewältigung*" OR Forschritt* OR Entwicklung* OR beobacht* OR überwach* OR beaufsichtig* OR Aufsicht* OR Aufseher* OR Maßnahme* OR Messen* ) | Expanders - Apply equivalent subjects | Interface - EBSCOhost Research Databases  Search Screen - Advanced Search  Database - Criminal Justice Abstracts | 254,219 |
|  |  | Search modes - Boolean/Phrase |  |  |
|  |  |  |  |  |
| S1 | "TI ( radikalisier* OR extremis* OR terroris* OR jihadi* OR dschihad* OR Gotteskriegertum* OR islamis* OR salafi* OR rechts* OR ""rechter Flügel*"" OR rechtsterroris* OR rechtsextrem* OR ""extreme Rechte"" OR neonazi* OR rechtsaußen* OR Rechtsaußen* OR Nationalist* OR linksaußen* OR ""weiße Vorherrschaft"" OR ""Überlegenheit der Weißen"" OR ""linker Flügel*"" OR linksterroris* OR linksextrem * OR ""extreme Linke"" OR anarch* ) OR AB ( radikalisier* OR extremis* OR terroris* OR jihadi* OR dschihad* OR Gotteskriegertum* OR islamis* OR salafi* OR rechts* OR ""rechter Flügel*"" OR rechtsterroris* OR rechtsextrem* OR ""extreme Rechte"" OR neonazi* OR rechtsaußen* OR Rechtsaußen* OR Nationalist* OR linksaußen* OR ""weiße Vorherrschaft"" OR ""Überlegenheit der Weißen"" OR ""linker Flügel*"" OR linksterroris* OR linksextrem * OR ""extreme Linke"" OR anarch* ) OR KW ( radikalisier* OR extremis* OR terroris* OR jihadi* OR dschihad* OR Gotteskriegertum* OR islamis* OR salafi* OR rechts* OR ""rechter Flügel*"" OR rechtsterroris* OR rechtsextrem* OR ""extreme Rechte"" OR neonazi* OR rechtsaußen* OR Rechtsaußen* OR Nationalist* OR linksaußen* OR ""weiße Vorherrschaft"" OR ""Überlegenheit der Weißen"" OR ""linker Flügel*"" OR linksterroris* OR linksextrem * OR ""extreme Linke"" OR anarch* ) OR SU ( radikalisier* OR extremis* OR terroris* OR jihadi* OR dschihad* OR Gotteskriegertum* OR islamis* OR salafi* OR rechts* OR ""rechter Flügel*"" OR rechtsterroris* OR rechtsextrem* OR ""extreme Rechte"" OR neonazi* OR rechtsaußen* OR Rechtsaußen* OR Nationalist* OR linksaußen* OR ""weiße Vorherrschaft"" OR ""Überlegenheit der Weißen"" OR ""linker Flügel*"" OR linksterroris* OR linksextrem * OR ""extreme Linke"" OR anarch* ) | Expanders - Apply equivalent subjects | Interface - EBSCOhost Research Databases  Search Screen - Advanced Search  Database - Criminal Justice Abstracts | 15,317 |
|  |  | Search modes - Boolean/Phrase |  |  |
|  |  |  |  |  |

Russian Key Words

| **#** | **Query** | **Limiters/Expanders** | **Last Run Via** | **Results** |
| --- | --- | --- | --- | --- |
| S5 | S1 AND S2 AND S3 | Narrow by Language: -  Russian  Search modes - Boolean/Phrase | Interface - EBSCOhost Research Databases  Search Screen - Advanced Search  Database - Criminal Justice Abstracts | 1 |
| S4 | S1 AND S2 AND S3 | Limiters - Publication Date: 20000101-20221231  Search modes - Boolean/Phrase | Interface - EBSCOhost Research Databases  Search Screen - Advanced Search  Database - Criminal Justice Abstracts | 17 |
| S3 | TI ( предупре* OR профилактик* OR предотвра* OR снижени* OR снизи* OR уменьш* OR противодейств* OR противостоя* OR реабORт* OR реинтегр* OR рецидив* OR дерадикализ* ) OR AB ( предупре* OR профилактик* OR предотвра* OR снижени* OR снизи* OR уменьш* OR противодейств* OR противостоя* OR реабORт* OR реинтегр* OR рецидив* OR дерадикализ* ) OR KW ( предупре* OR профилактик* OR предотвра* OR снижени* OR снизи* OR уменьш* OR противодейств* OR противостоя* OR реабORт* OR реинтегр* OR рецидив* OR дерадикализ* ) OR SU ( предупре* OR профилактик* OR предотвра* OR снижени* OR снизи* OR уменьш* OR противодейств* OR противостоя* OR реабORт* OR реинтегр* OR рецидив* OR дерадикализ* ) | Expanders - Apply equivalent subjects  Search modes - Boolean/Phrase | Interface - EBSCOhost Research Databases  Search Screen - Advanced Search  Database - Criminal Justice Abstracts | 117 |
|  |  |  |  |  |
|  |  |  |  |  |
| S2 | TI ( Инициатив* OR Вмешательств* OR программ* OR политик* OR схема* OR терап* OR подход* OR модель OR стратеги* метод* OR проект* OR практи* OR инструмент* OR концепци* OR структур* OR протокол* OR инструк* OR принцип* OR разработк* OR масштаб* OR системн* OR пример* OR обзор* OR критери* OR опросник* OR направлен* OR консульта* OR оцен* OR диагности* OR план* OR "план развития" OR мониторинг* OR "план ведения" OR "план* поддерж*" OR "управлени* риск*" OR развитие OR контрол* OR наблюд* OR оцен* OR измер* ) OR AB ( Инициатив* OR Вмешательств* OR программ* OR политик* OR схема* OR терап* OR подход* OR модель OR стратеги* метод* OR проект* OR практи* OR инструмент* OR концепци* OR структур* OR протокол* OR инструк* OR принцип* OR разработк* OR масштаб* OR системн* OR пример* OR обзор* OR критери* OR опросник* OR направлен* OR консульта* OR оцен* OR диагности* OR план* OR "план развития" OR мониторинг* OR "план ведения" OR "план* поддерж*" OR "управлени* риск*" OR развитие OR контрол* OR наблюд* OR оцен* OR измер* ) OR KW ( Инициатив* OR Вмешательств* OR программ* OR политик* OR схема* OR терап* OR подход* OR модель OR стратеги* метод* OR проект* OR практи* OR инструмент* OR концепци* OR структур* OR протокол* OR инструк* OR принцип* OR разработк* OR масштаб* OR системн* OR пример* OR обзор* OR критери* OR опросник* OR направлен* OR консульта* OR оцен* OR диагности* OR план* OR "план развития" OR мониторинг* OR "план ведения" OR "план* поддерж*" OR "управлени* риск*" OR развитие OR контрол* OR наблюд* OR оцен* OR измер* ) OR SU ( Инициатив* OR Вмешательств* OR программ* OR политик* OR схема* OR терап* OR подход* OR модель OR стратеги* метод* OR проект* OR практи* OR инструмент* OR концепци* OR структур* OR протокол* OR инструк* OR принцип* OR разработк* OR масштаб* OR системн* OR пример* OR обзор* OR критери* OR опросник* OR направлен* OR консульта* OR оцен* OR диагности* OR план* OR "план развития" OR мониторинг* OR "план ведения" OR "план* поддерж*" OR "управлени* риск*" OR развитие OR контрол* OR наблюд* OR оцен* OR измер* ) | Expanders - Apply equivalent subjects | Interface - EBSCOhost Research Databases  Search Screen - Advanced Search  Database - Criminal Justice Abstracts | 506 |
|  |  | Search modes - Boolean/Phrase |  |  |
|  |  |  |  |  |
| S1 | TI ( радикализ* OR экстреми* OR террори* OR джихади* OR ислами* OR салафи* OR правое-крыло OR "правое крыло" OR крайне-правый OR "крайне правый" OR неонацист OR националист* OR превосходства бел* OR превосходства белой расы OR левое-крыло OR "левое крыло" OR крайне-левый OR "крайне левый" OR анарх* OR единичн* ) OR AB ( радикализ* OR экстреми* OR террори* OR джихади* OR ислами* OR салафи* OR правое-крыло OR "правое крыло" OR крайне-правый OR "крайне правый" OR неонацист OR националист* OR превосходства бел* OR превосходства белой расы OR левое-крыло OR "левое крыло" OR крайне-левый OR "крайне левый" OR анарх* OR единичн* ) OR KW ( радикализ* OR экстреми* OR террори* OR джихади* OR ислами* OR салафи* OR правое-крыло OR "правое крыло" OR крайне-правый OR "крайне правый" OR неонацист OR националист* OR превосходства бел* OR превосходства белой расы OR левое-крыло OR "левое крыло" OR крайне-левый OR "крайне левый" OR анарх* OR единичн* ) OR SU ( радикализ* OR экстреми* OR террори* OR джихади* OR ислами* OR салафи* OR правое-крыло OR "правое крыло" OR крайне-правый OR "крайне правый" OR неонацист OR националист* OR превосходства бел* OR превосходства белой расы OR левое-крыло OR "левое крыло" OR крайне-левый OR "крайне левый" OR анарх* OR единичн* ) | Expanders - Apply equivalent subjects | Interface - EBSCOhost Research Databases  Search Screen - Advanced Search  Database - Criminal Justice Abstracts | 46 |
|  |  | Search modes - Boolean/Phrase |  |  |
|  |  |  |  |  |

Danish Key Words

| **#** | **Query** | **Limiters/Expanders** | **Last Run Via** | **Results** |
| --- | --- | --- | --- | --- |
| S5 | S1 AND S2 AND S3 | Limiters/Expanders  Expanders - Apply equivalent subjects  Narrow by Language: - Norwegian  Macedonian  Lithuanian  Italian  Bosnian  Danish  Russian  Croatian  Dutch/Flemish  Turkish  Spanish  Slovenian  French  Romanian  German  Search modes - Boolean/Phrase | Interface - EBSCOhost Research Databases  Search Screen - Advanced Search  Database - Criminal Justice Abstracts | 101 |
| S4 | S1 AND S2 AND S3 | Limiters - Publication Date: 20000101-20221231  Search modes - Boolean/Phrase | Interface - EBSCOhost Research Databases  Search Screen - Advanced Search  Database - Criminal Justice Abstracts | 3,344 |
| S3 | TI ( prevent* OR forebyg* OR reducere* OR formindske OR counter* OR bekæmp* OR disengage* OR afhop* OR rehab* OR reintegrat* OR gen-integrat* OR re-entry OR genindtræde OR return* OR desist* OR afstå* OR "tage afstand fra" OR recidivism OR deradikal* OR tilbagfald OR de-radikal* OR afradikalisering OR exit* ) OR AB ( prevent* OR forebyg* OR reducere* OR formindske OR counter* OR bekæmp* OR disengage* OR afhop* OR rehab* OR reintegrat* OR gen-integrat* OR re-entry OR genindtræde OR return* OR desist* OR afstå* OR "tage afstand fra" OR recidivism OR deradikal* OR tilbagfald OR de-radikal* OR afradikalisering OR exit* ) OR KW ( prevent* OR forebyg* OR reducere* OR formindske OR counter* OR bekæmp* OR disengage* OR afhop* OR rehab* OR reintegrat* OR gen-integrat* OR re-entry OR genindtræde OR return* OR desist* OR afstå* OR "tage afstand fra" OR recidivism OR deradikal* OR tilbagfald OR de-radikal* OR afradikalisering OR exit* ) OR SU ( prevent* OR forebyg* OR reducere* OR formindske OR counter* OR bekæmp* OR disengage* OR afhop* OR rehab* OR reintegrat* OR gen-integrat* OR re-entry OR genindtræde OR return* OR desist* OR afstå* OR "tage afstand fra" OR recidivism OR deradikal* OR tilbagfald OR de-radikal* OR afradikalisering OR exit* ) | Limiters - Publication Date: 20000101-20221231  Search modes - Boolean/Phrase | Interface - EBSCOhost Research Databases  Search Screen - Advanced Search  Database - Criminal Justice Abstracts | 87,919 |
|  |
|  |
| S2 | TI ( initiativ* OR interven* OR program* OR tilgang OR policy OR policies OR behandling OR model* OR strateg* OR metod* OR projekt OR instrument* OR tool* OR redskab OR framework OR ramme OR protokol OR analy* OR guide* OR vejledning OR skala OR system* OR indeks OR beholdning OR matrix OR format* OR retningslin* OR template* OR skabelon OR profil OR kriterie* OR spørgeskema OR refer* OR henvisning OR assess* OR vurde* OR måling OR screening OR profil* OR "case plan*" OR "case formulering" OR "management plan*" OR "treatment plan*" OR "behandlingsplan" OR "behandlingsforløb" OR "behandlingsprogram"OR "support plan*" OR "støtteforløb" OR "case manage*" OR "Sagsbehandling*" OR "plan" OR "risikohåndtering*" OR "sikkerhedsvurdering" OR "sikkerhedsanalyse" OR progress OR fremskridt OR forbedringer OR monitor* OR supervis* OR måle* OR måling ) OR AB ( initiativ* OR interven* OR program* OR tilgang OR policy OR policies OR behandling OR model* OR strateg* OR metod* OR projekt OR instrument* OR tool* OR redskab OR framework OR ramme OR protokol OR analy* OR guide* OR vejledning OR skala OR system* OR indeks OR beholdning OR matrix OR format* OR retningslin* OR template* OR skabelon OR profil OR kriterie* OR spørgeskema OR refer* OR henvisning OR assess* OR vurde* OR måling OR screening OR profil* OR "case plan*" OR "case formulering" OR "management plan*" OR "treatment plan*" OR "behandlingsplan" OR "behandlingsforløb" OR "behandlingsprogram"OR "support plan*" OR "støtteforløb" OR "case manage*" OR "Sagsbehandling*" OR "plan" OR "risikohåndtering*" OR "sikkerhedsvurdering" OR "sikkerhedsanalyse" OR progress OR fremskridt OR forbedringer OR monitor* OR supervis* OR måle* OR måling ) OR KW ( initiativ* OR interven* OR program* OR tilgang OR policy OR policies OR behandling OR model* OR strateg* OR metod* OR projekt OR instrument* OR tool* OR redskab OR framework OR ramme OR protokol OR analy* OR guide* OR vejledning OR skala OR system* OR indeks OR beholdning OR matrix OR format* OR retningslin* OR template* OR skabelon OR profil OR kriterie* OR spørgeskema OR refer* OR henvisning OR assess* OR vurde* OR måling OR screening OR profil* OR "case plan*" OR "case formulering" OR "management plan*" OR "treatment plan*" OR "behandlingsplan" OR "behandlingsforløb" OR "behandlingsprogram"OR "support plan*" OR "støtteforløb" OR "case manage*" OR "Sagsbehandling*" OR "plan" OR "risikohåndtering*" OR "sikkerhedsvurdering" OR "sikkerhedsanalyse" OR progress OR fremskridt OR forbedringer OR monitor* OR supervis* OR måle* OR måling ) OR SU ( initiativ* OR interven* OR program* OR tilgang OR policy OR policies OR behandling OR model* OR strateg* OR metod* OR projekt OR instrument* OR tool* OR redskab OR framework OR ramme OR protokol OR analy* OR guide* OR vejledning OR skala OR system* OR indeks OR beholdning OR matrix OR format* OR retningslin* OR template* OR skabelon OR profil OR kriterie* OR spørgeskema OR refer* OR henvisning OR assess* OR vurde* OR måling OR screening OR profil* OR "case plan*" OR "case formulering" OR "management plan*" OR "treatment plan*" OR "behandlingsplan" OR "behandlingsforløb" OR "behandlingsprogram"OR "support plan*" OR "støtteforløb" OR "case manage*" OR "Sagsbehandling*" OR "plan" OR "risikohåndtering*" OR "sikkerhedsvurdering" OR "sikkerhedsanalyse" OR progress OR fremskridt OR forbedringer OR monitor* OR supervis* OR måle* OR måling ) | Limiters - Publication Date: 20000101-20221231  Search modes - Boolean/Phrase | Interface - EBSCOhost Research Databases  Search Screen - Advanced Search  Database - Criminal Justice Abstracts | 338,756 |
|  |
|  |
| S1 | TI ( radikali* OR ekstremis* OR terroris* OR jihadi* OR islamis* OR salafi* OR højreorienteret OR "højreorienteret" OR "ekstrem højreorienteret" OR højreekstrem OR nynazis* OR nationalis* OR "hvidt overherredømme" OR venstreorienteret OR "ekstrem venstreorienteret" OR anark* OR enkeltsag ) OR AB ( radikali* OR ekstremis* OR terroris* OR jihadi* OR islamis* OR salafi* OR højreorienteret OR "højreorienteret" OR "ekstrem højreorienteret" OR højreekstrem OR nynazis* OR nationalis* OR "hvidt overherredømme" OR venstreorienteret OR "ekstrem venstreorienteret" OR anark* OR enkeltsag ) OR KW ( radikali* OR ekstremis* OR terroris* OR jihadi* OR islamis* OR salafi* OR højreorienteret OR "højreorienteret" OR "ekstrem højreorienteret" OR højreekstrem OR nynazis* OR nationalis* OR "hvidt overherredømme" OR venstreorienteret OR "ekstrem venstreorienteret" OR anark* OR enkeltsag ) OR SU ( radikali* OR ekstremis* OR terroris* OR jihadi* OR islamis* OR salafi* OR højreorienteret OR "højreorienteret" OR "ekstrem højreorienteret" OR højreekstrem OR nynazis* OR nationalis* OR "hvidt overherredømme" OR venstreorienteret OR "ekstrem venstreorienteret" OR anark* OR enkeltsag ) | Limiters - Publication Date: 20000101-20221231  Search modes - Boolean/Phrase | Interface - EBSCOhost Research Databases  Search Screen - Advanced Search  Database - Criminal Justice Abstracts | 13,426 |
|  |
|  |

Norwegian Key Words

| **#** | **Query** | **Limiters/Expanders** | **Last Run Via** | **Results** |
| --- | --- | --- | --- | --- |
| S5 | S1 AND S2 AND S3 | Expanders - Apply equivalent subjects  Narrow by Language: - Macedonian  Italian  Danish  Bosnian  Russian  Croatian  Dutch/ Flemish  Turkish  Spanish  Slovenian  French  Romanian  German  Search modes - Boolean/Phrase | Interface - EBSCOhost Research Databases  Search Screen - Advanced Search  Database - Criminal Justice Abstracts | 88 |
| S4 | S1 AND S2 AND S3 | Limiters - Publication Date: 20000101-20221231  Search modes - Boolean/Phrase | Interface - EBSCOhost Research Databases  Search Screen - Advanced Search  Database - Criminal Justice Abstracts | 2,938 |
| S3 | TI ( prevent* OR forhindre* OR reducere* OR reduser* OR counter* OR kamp OR disengage* OR afhop* OR rehab* ELER reintegrat* OR genintegrer* OR re-entry OR retur* OR desist* OR avstå* OR "avstand fra" OR recidivism OR deradikal* OR tilbakefall OR de-radikal* OR avradikalisering OR exit* ) OR AB ( prevent* OR forhindre* OR reducere* OR reduser* OR counter* OR kamp OR disengage* OR afhop* OR rehab* ELER reintegrat* OR genintegrer* OR re-entry OR retur* OR desist* OR avstå* OR "avstand fra" OR recidivism OR deradikal* OR tilbakefall OR de-radikal* OR avradikalisering OR exit* ) OR KW ( prevent* OR forhindre* OR reducere* OR reduser* OR counter* OR kamp OR disengage* OR afhop* OR rehab* ELER reintegrat* OR genintegrer* OR re-entry OR retur* OR desist* OR avstå* OR "avstand fra" OR recidivism OR deradikal* OR tilbakefall OR de-radikal* OR avradikalisering OR exit* ) OR SU ( prevent* OR forhindre* OR reducere* OR reduser* OR counter* OR kamp OR disengage* OR afhop* OR rehab* ELER reintegrat* OR genintegrer* OR re-entry OR retur* OR desist* OR avstå* OR "avstand fra" OR recidivism OR deradikal* OR tilbakefall OR de-radikal* OR avradikalisering OR exit* ) | Expanders - Apply equivalent subjects  Search modes - Boolean/Phrase | Interface - EBSCOhost Research Databases  Search Screen - Advanced Search  Database - Criminal Justice Abstracts | 91,456 |
|  |
|  |
| S2 | TI ( initiativ* OR intervensjon OR program* OR adgang OR policy OR behandle OR modell OR strateg* OR metod* OR prosjekt OR instrument* OR tool* OR verktøy OR framework OR rammeverk OR protokoll OR analyse OR guide* OR veiledning OR skala OR system OR indeks OR inventar OR matrix OR metrisk OR format* OR retningslinjer OR template* OR mal OR profil* OR kriterie* OR spørreskjema OR refer* OR referanse OR assess* OR vurdere* OR måling OR screening OR profil OR "case plan*" OR "Saksplan*" OR "management plan*" OR "treatment plan*" OR "behandlingsplan*" OR "behandlingsforløp" OR "behandlingsprogram"OR "support plan*" OR "Støttekurs" OR "case manage*" OR "Saksbehandling *" OR "plan" OR "risikohåndtering*" OR "Sikkerhetsvurdering" OR "sikkerhetsanalyse" OR progress OR fremgang OR forbedringer OR monitor* OR tilsyn* OR måle* OR mål ) OR AB ( initiativ* OR intervensjon OR program* OR adgang OR policy OR behandle OR modell OR strateg* OR metod* OR prosjekt OR instrument* OR tool* OR verktøy OR framework OR rammeverk OR protokoll OR analyse OR guide* OR veiledning OR skala OR system OR indeks OR inventar OR matrix OR metrisk OR format* OR retningslinjer OR template* OR mal OR profil* OR kriterie* OR spørreskjema OR refer* OR referanse OR assess* OR vurdere* OR måling OR screening OR profil OR "case plan*" OR "Saksplan*" OR "management plan*" OR "treatment plan*" OR "behandlingsplan*" OR "behandlingsforløp" OR "behandlingsprogram"OR "support plan*" OR "Støttekurs" OR "case manage*" OR "Saksbehandling *" OR "plan" OR "risikohåndtering*" OR "Sikkerhetsvurdering" OR "sikkerhetsanalyse" OR progress OR fremgang OR forbedringer OR monitor* OR tilsyn* OR måle* OR mål ) OR KW ( initiativ* OR intervensjon OR program* OR adgang OR policy OR behandle OR modell OR strateg* OR metod* OR prosjekt OR instrument* OR tool* OR verktøy OR framework OR rammeverk OR protokoll OR analyse OR guide* OR veiledning OR skala OR system OR indeks OR inventar OR matrix OR metrisk OR format* OR retningslinjer OR template* OR mal OR profil* OR kriterie* OR spørreskjema OR refer* OR referanse OR assess* OR vurdere* OR måling OR screening OR profil OR "case plan*" OR "Saksplan*" OR "management plan*" OR "treatment plan*" OR "behandlingsplan*" OR "behandlingsforløp" OR "behandlingsprogram"OR "support plan*" OR "Støttekurs" OR "case manage*" OR "Saksbehandling *" OR "plan" OR "risikohåndtering*" OR "Sikkerhetsvurdering" OR "sikkerhetsanalyse" OR progress OR fremgang OR forbedringer OR monitor* OR tilsyn* OR måle* OR mål ) OR SU ( initiativ* OR intervensjon OR program* OR adgang OR policy OR behandle OR modell OR strateg* OR metod* OR prosjekt OR instrument* OR tool* OR verktøy OR framework OR rammeverk OR protokoll OR analyse OR guide* OR veiledning OR skala OR system OR indeks OR inventar OR matrix OR metrisk OR format* OR retningslinjer OR template* OR mal OR profil* OR kriterie* OR spørreskjema OR refer* OR referanse OR assess* OR vurdere* OR måling OR screening OR profil OR "case plan*" OR "Saksplan*" OR "management plan*" OR "treatment plan*" OR "behandlingsplan*" OR "behandlingsforløp" OR "behandlingsprogram"OR "support plan*" OR "Støttekurs" OR "case manage*" OR "Saksbehandling *" OR "plan" OR "risikohåndtering*" OR "Sikkerhetsvurdering" OR "sikkerhetsanalyse" OR progress OR fremgang OR forbedringer OR monitor* OR tilsyn* OR måle* OR mål ) | Expanders - Apply equivalent subjects  Search modes - Boolean/Phrase | Interface - EBSCOhost Research Databases  Search Screen - Advanced Search  Database - Criminal Justice Abstracts | 338,590 |
|  |
|  |
| S1 | TI ( radikali* OR ekstremis* OR terroris* OR jihadi* OR islamis* OR salafi* OR "høyre ving" OR høyreekstreme OR nynazis* OR nasjonalistis* OR "hvit overherredømme" OR venstreorientert OR "ekstrem venstreorientert" OR anark* OR enkelttilfelle ) OR AB ( radikali* OR ekstremis* OR terroris* OR jihadi* OR islamis* OR salafi* OR "høyre ving" OR høyreekstreme OR nynazis* OR nasjonalistis* OR "hvit overherredømme" OR venstreorientert OR "ekstrem venstreorientert" OR anark* OR enkelttilfelle ) OR KW ( radikali* OR ekstremis* OR terroris* OR jihadi* OR islamis* OR salafi* OR "høyre ving" OR høyreekstreme OR nynazis* OR nasjonalistis* OR "hvit overherredømme" OR venstreorientert OR "ekstrem venstreorientert" OR anark* OR enkelttilfelle ) OR SU ( radikali* OR ekstremis* OR terroris* OR jihadi* OR islamis* OR salafi* OR "høyre ving" OR høyreekstreme OR nynazis* OR nasjonalistis* OR "hvit overherredømme" OR venstreorientert OR "ekstrem venstreorientert" OR anark* OR enkelttilfelle ) | Expanders - Apply equivalent subjects  Search modes - Boolean/Phrase | Interface - EBSCOhost Research Databases  Search Screen - Advanced Search  Database - Criminal Justice Abstracts | 12,784 |
|  |
|  |

Swedish Key Words

| **#** | **Query** | **Limiters/Expanders** | **Last Run Via** | **Results** |
| --- | --- | --- | --- | --- |
| S5 | S1 AND S2 AND S3 | Expanders - Apply equivalent subjects  Narrow by Language: - Macedonian  Lithuanian  Italian  Bosnian  Danish  Russian  Croatian  Dutch/Flemish  Turkish  Spanish  Slovenian  French  Romanian  German  Search modes - Boolean/Phrase | Interface - EBSCOhost Research Databases  Search Screen - Advanced Search  Database - Criminal Justice Abstracts | 92 |
| S4 | S1 AND S2 AND S3 | Limiters - Publication Date: 20000101-20221231  Search modes - Boolean/Phrase | Interface - EBSCOhost Research Databases  Search Screen - Advanced Search  Database - Criminal Justice Abstracts | 3,198 |
| S3 | TI ( prevent* OR förhindra* OR reducere* OR minska* OR counter* OR bekämpa OR disengage* OR afhop* OR rehab* ELER reintegrat* OR återintegrera* OR re-entry OR återinträde OR returnera* OR desist* OR avstå* OR "avstånd från" OR recidivism OR deradikal* OR "falla tillbaka" OR de-radikal* OR avradikal* OR avradikalisering OR exit* OR utgång ) OR AB ( prevent* OR förhindra* OR reducere* OR minska* OR counter* OR bekämpa OR disengage* OR afhop* OR rehab* ELER reintegrat* OR återintegrera* OR re-entry OR återinträde OR returnera* OR desist* OR avstå* OR "avstånd från" OR recidivism OR deradikal* OR "falla tillbaka" OR de-radikal* OR avradikal* OR avradikalisering OR exit* OR utgång ) OR KW ( prevent* OR förhindra* OR reducere* OR minska* OR counter* OR bekämpa OR disengage* OR afhop* OR rehab* ELER reintegrat* OR återintegrera* OR re-entry OR återinträde OR returnera* OR desist* OR avstå* OR "avstånd från" OR recidivism OR deradikal* OR "falla tillbaka" OR de-radikal* OR avradikal* OR avradikalisering OR exit* OR utgång ) OR SU ( prevent* OR förhindra* OR reducere* OR minska* OR counter* OR bekämpa OR disengage* OR afhop* OR rehab* ELER reintegrat* OR återintegrera* OR re-entry OR återinträde OR returnera* OR desist* OR avstå* OR "avstånd från" OR recidivism OR deradikal* OR "falla tillbaka" OR de-radikal* OR avradikal* OR avradikalisering OR exit* OR utgång ) | Expanders - Apply equivalent subjects  Search modes - Boolean/Phrase | Interface - EBSCOhost Research Databases  Search Screen - Advanced Search  Database - Criminal Justice Abstracts | 85,909 |
|  |
|  |
| S2 | TI ( initiativ* OR intervensjon OR program* OR tillgång OR policy OR policies OR behandla* OR model* OR strateg* OR metod* OR projekt OR instrument* OR tool* OR verktyg OR framework* OR ram OR protokoll OR analy* OR guide* OR vägledning OR skala OR system* OR indeks OR inventering OR matrix OR metrisk OR format* OR riktlinj* OR template* OR mall OR profil* OR kriterie* OR frågeformulär OR refer* OR referens OR assess* OR bedöm* OR mätning OR screen* OR "case plan*" OR "Fallformulering" OR "management plan*" OR "treatment plan*" OR "behandlingsplan*" OR "behandlingsförlopp" OR "behandlingsprogram"OR "support plan*" OR "case manage*" OR "plan" OR "Planera" OR "Riskhantering*" OR "Säkerhetsbedömning" OR "Säkerhetsanalys" OR progress OR framsteg OR förbättringar OR monitor* OR övervaka* OR mäta* OR mått ) OR AB ( initiativ* OR intervensjon OR program* OR tillgång OR policy OR policies OR behandla* OR model* OR strateg* OR metod* OR projekt OR instrument* OR tool* OR verktyg OR framework* OR ram OR protokoll OR analy* OR guide* OR vägledning OR skala OR system* OR indeks OR inventering OR matrix OR metrisk OR format* OR riktlinj* OR template* OR mall OR profil* OR kriterie* OR frågeformulär OR refer* OR referens OR assess* OR bedöm* OR mätning OR screen* OR "case plan*" OR "Fallformulering" OR "management plan*" OR "treatment plan*" OR "behandlingsplan*" OR "behandlingsförlopp" OR "behandlingsprogram"OR "support plan*" OR "case manage*" OR "plan" OR "Planera" OR "Riskhantering*" OR "Säkerhetsbedömning" OR "Säkerhetsanalys" OR progress OR framsteg OR förbättringar OR monitor* OR övervaka* OR mäta* OR mått ) OR KW ( initiativ* OR intervensjon OR program* OR tillgång OR policy OR policies OR behandla* OR model* OR strateg* OR metod* OR projekt OR instrument* OR tool* OR verktyg OR framework* OR ram OR protokoll OR analy* OR guide* OR vägledning OR skala OR system* OR indeks OR inventering OR matrix OR metrisk OR format* OR riktlinj* OR template* OR mall OR profil* OR kriterie* OR frågeformulär OR refer* OR referens OR assess* OR bedöm* OR mätning OR screen* OR "case plan*" OR "Fallformulering" OR "management plan*" OR "treatment plan*" OR "behandlingsplan*" OR "behandlingsförlopp" OR "behandlingsprogram"OR "support plan*" OR "case manage*" OR "plan" OR "Planera" OR "Riskhantering*" OR "Säkerhetsbedömning" OR "Säkerhetsanalys" OR progress OR framsteg OR förbättringar OR monitor* OR övervaka* OR mäta* OR mått ) OR SU ( initiativ* OR intervensjon OR program* OR tillgång OR policy OR policies OR behandla* OR model* OR strateg* OR metod* OR projekt OR instrument* OR tool* OR verktyg OR framework* OR ram OR protokoll OR analy* OR guide* OR vägledning OR skala OR system* OR indeks OR inventering OR matrix OR metrisk OR format* OR riktlinj* OR template* OR mall OR profil* OR kriterie* OR frågeformulär OR refer* OR referens OR assess* OR bedöm* OR mätning OR screen* OR "case plan*" OR "Fallformulering" OR "management plan*" OR "treatment plan*" OR "behandlingsplan*" OR "behandlingsförlopp" OR "behandlingsprogram"OR "support plan*" OR "case manage*" OR "plan" OR "Planera" OR "Riskhantering*" OR "Säkerhetsbedömning" OR "Säkerhetsanalys" OR progress OR framsteg OR förbättringar OR monitor* OR övervaka* OR mäta* OR mått ) | Expanders - Apply equivalent subjects  Search modes - Boolean/Phrase | Interface - EBSCOhost Research Databases  Search Screen - Advanced Search  Database - Criminal Justice Abstracts | 399,236 |
|  |
|  |
| S1 | TI ( radikali* OR ekstremis* OR terroris* OR jihadi* OR islamis* OR salafi* OR "högra vingen" OR extremhöger OR nynazis* OR nationalis* OR "vit överlägsenhet" OR vänsterorienterad OR vänster OR extremvänster OR anark* OR "enskilda fall" ) OR AB ( radikali* OR ekstremis* OR terroris* OR jihadi* OR islamis* OR salafi* OR "högra vingen" OR extremhöger OR nynazis* OR nationalis* OR "vit överlägsenhet" OR vänsterorienterad OR vänster OR extremvänster OR anark* OR "enskilda fall" ) OR KW ( radikali* OR ekstremis* OR terroris* OR jihadi* OR islamis* OR salafi* OR "högra vingen" OR extremhöger OR nynazis* OR nationalis* OR "vit överlägsenhet" OR vänsterorienterad OR vänster OR extremvänster OR anark* OR "enskilda fall" ) OR SU ( radikali* OR ekstremis* OR terroris* OR jihadi* OR islamis* OR salafi* OR "högra vingen" OR extremhöger OR nynazis* OR nationalis* OR "vit överlägsenhet" OR vänsterorienterad OR vänster OR extremvänster OR anark* OR "enskilda fall" ) | Expanders - Apply equivalent subjects  Search modes - Boolean/Phrase | Interface - EBSCOhost Research Databases  Search Screen - Advanced Search  Database - Criminal Justice Abstracts | 14,701 |
|  |
|  |

**Table A1.3(b). Scopus Search Records – Part I (LOE)**

LOE with English Key Words

| 1 | TITLE-ABS-KEY ( radicali* OR extremis* OR terroris* OR jihadi* OR islamis* OR salafi* OR right-wing OR "right wing" OR extreme-right OR "extreme right" OR neo-nazi OR far-right OR "far right" OR nationalist* OR "white supremacis*" OR left-wing OR "left wing" OR extreme-left OR "extreme left" OR anarch* OR "single issue" OR single-issue ) | 134,656 results |
| --- | --- | --- |
| 2 | TITLE-ABS-KEY ( initiative* OR interven* OR program* OR policy OR policies OR scheme* OR treat* OR approach* OR model* OR strateg* OR method* OR project* OR practice* OR instrument* OR tool* OR framework* OR protocol* OR guid* OR scale* OR system* OR inventor* OR metric* OR template* OR profile* OR criteria OR questionnaire* OR refer* OR assess* OR "case plan*" OR "case formulat*" OR "management plan*" OR "treatment plan*" OR "support plan*" OR "case manage*" OR "risk manage*" OR progress* OR monitor* OR supervis* OR measur* ) | 59,648,382 results |
| 3 | TITLE-ABS-KEY ( prevent* OR reduc* OR counter* OR disengage* OR rehab* OR reintegrat* OR re-integrat* OR re-entry OR reentry OR desist* OR recidivism OR deradical* OR de-radical* OR exit* ) | 13,424,906 results |
| 4 | ( TITLE-ABS-KEY ( radicali* OR extremis* OR terroris* OR jihadi* OR islamis* OR salafi* OR right-wing OR "right wing" OR extreme-right OR "extreme right" OR neo-nazi OR far-right OR "far right" OR nationalist* OR "white supremacis*" OR left-wing OR "left wing" OR extreme-left OR "extreme left" OR anarch* OR "single issue" OR single-issue ) ) AND ( TITLE-ABS-KEY ( initiative* OR interven* OR program* OR policy OR policies OR scheme* OR treat* OR approach* OR model* OR strateg* OR method* OR project* OR practice* OR instrument* OR tool* OR framework* OR protocol* OR guid* OR scale* OR system* OR inventor* OR metric* OR template* OR profile* OR criteria OR questionnaire* OR refer* OR assess* OR "case plan*" OR "case formulat*" OR "management plan*" OR "treatment plan*" OR "support plan*" OR "case manage*" OR "risk manage*" OR progress* OR monitor* OR supervis* OR measur* ) ) AND ( TITLE-ABS-KEY ( prevent* OR reduc* OR counter* OR disengage* OR rehab* OR reintegrat* OR re-integrat* OR re-entry OR reentry OR desist* OR recidivism OR deradical* OR de-radical* OR exit* ) ) | 22,911 results |
| 5 | ( TITLE-ABS-KEY ( radicali* OR extremis* OR terroris* OR jihadi* OR islamis* OR salafi* OR right-wing OR "right wing" OR extreme-right OR "extreme right" OR neo-nazi OR far-right OR "far right" OR nationalist* OR "white supremacis*" OR left-wing OR "left wing" OR extreme-left OR "extreme left" OR anarch* OR "single issue" OR single-issue ) ) AND ( TITLE-ABS-KEY ( initiative* OR interven* OR program* OR policy OR policies OR scheme* OR treat* OR approach* OR model* OR strateg* OR method* OR project* OR practice* OR instrument* OR tool* OR framework* OR protocol* OR guid* OR scale* OR system* OR inventor* OR metric* OR template* OR profile* OR criteria OR questionnaire* OR refer* OR assess* OR "case plan*" OR "case formulat*" OR "management plan*" OR "treatment plan*" OR "support plan*" OR "case manage*" OR "risk manage*" OR progress* OR monitor* OR supervis* OR measur* ) ) AND ( TITLE-ABS-KEY ( prevent* OR reduc* OR counter* OR disengage* OR rehab* OR reintegrat* OR re-integrat* OR re-entry OR reentry OR desist* OR recidivism OR deradical* OR de-radical* OR exit* ) ) AND ( LIMIT-TO ( PUBYEAR , 2022 ) OR LIMIT-TO ( PUBYEAR , 2021 ) OR LIMIT-TO ( PUBYEAR , 2020 ) OR LIMIT-TO ( PUBYEAR , 2019 ) OR LIMIT-TO ( PUBYEAR , 2018 ) OR LIMIT-TO ( PUBYEAR , 2017 ) OR LIMIT-TO ( PUBYEAR , 2016 ) OR LIMIT-TO ( PUBYEAR , 2015 ) OR LIMIT-TO ( PUBYEAR , 2014 ) OR LIMIT-TO ( PUBYEAR , 2013 ) OR LIMIT-TO ( PUBYEAR , 2012 ) OR LIMIT-TO ( PUBYEAR , 2011 ) OR LIMIT-TO ( PUBYEAR , 2010 ) OR LIMIT-TO ( PUBYEAR , 2009 ) OR LIMIT-TO ( PUBYEAR , 2008 ) OR LIMIT-TO ( PUBYEAR , 2007 ) OR LIMIT-TO ( PUBYEAR , 2006 ) OR LIMIT-TO ( PUBYEAR , 2005 ) OR LIMIT-TO ( PUBYEAR , 2004 ) OR LIMIT-TO ( PUBYEAR , 2003 ) OR LIMIT-TO ( PUBYEAR , 2002 ) OR LIMIT-TO ( PUBYEAR , 2001 ) OR LIMIT-TO ( PUBYEAR , 2000 ) ) | 21,995 results |
| 6 | ( TITLE-ABS-KEY ( radicali* OR extremis* OR terroris* OR jihadi* OR islamis* OR salafi* OR right-wing OR "right wing" OR extreme-right OR "extreme right" OR neo-nazi OR far-right OR "far right" OR nationalist* OR "white supremacis*" OR left-wing OR "left wing" OR extreme-left OR "extreme left" OR anarch* OR "single issue" OR single-issue ) ) AND ( TITLE-ABS-KEY ( initiative* OR interven* OR program* OR policy OR policies OR scheme* OR treat* OR approach* OR model* OR strateg* OR method* OR project* OR practice* OR instrument* OR tool* OR framework* OR protocol* OR guid* OR scale* OR system* OR inventor* OR metric* OR template* OR profile* OR criteria OR questionnaire* OR refer* OR assess* OR "case plan*" OR "case formulat*" OR "management plan*" OR "treatment plan*" OR "support plan*" OR "case manage*" OR "risk manage*" OR progress* OR monitor* OR supervis* OR measur* ) ) AND ( TITLE-ABS-KEY ( prevent* OR reduc* OR counter* OR disengage* OR rehab* OR reintegrat* OR re-integrat* OR re-entry OR reentry OR desist* OR recidivism OR deradical* OR de-radical* OR exit* ) ) AND ( LIMIT-TO ( PUBYEAR , 2022 ) OR LIMIT-TO ( PUBYEAR , 2021 ) OR LIMIT-TO ( PUBYEAR , 2020 ) OR LIMIT-TO ( PUBYEAR , 2019 ) OR LIMIT-TO ( PUBYEAR , 2018 ) OR LIMIT-TO ( PUBYEAR , 2017 ) OR LIMIT-TO ( PUBYEAR , 2016 ) OR LIMIT-TO ( PUBYEAR , 2015 ) OR LIMIT-TO ( PUBYEAR , 2014 ) OR LIMIT-TO ( PUBYEAR , 2013 ) OR LIMIT-TO ( PUBYEAR , 2012 ) OR LIMIT-TO ( PUBYEAR , 2011 ) OR LIMIT-TO ( PUBYEAR , 2010 ) OR LIMIT-TO ( PUBYEAR , 2009 ) OR LIMIT-TO ( PUBYEAR , 2008 ) OR LIMIT-TO ( PUBYEAR , 2007 ) OR LIMIT-TO ( PUBYEAR , 2006 ) OR LIMIT-TO ( PUBYEAR , 2005 ) OR LIMIT-TO ( PUBYEAR , 2004 ) OR LIMIT-TO ( PUBYEAR , 2003 ) OR LIMIT-TO ( PUBYEAR , 2002 ) OR LIMIT-TO ( PUBYEAR , 2001 ) OR LIMIT-TO ( PUBYEAR , 2000 ) ) AND ( LIMIT-TO ( LANGUAGE , "russian" ) OR LIMIT-TO ( LANGUAGE , "french" ) OR LIMIT-TO ( LANGUAGE , "spanish" ) OR LIMIT-TO ( LANGUAGE , "german" ) OR LIMIT-TO ( LANGUAGE , "chinese" ) OR LIMIT-TO ( LANGUAGE , "italian" ) OR LIMIT-TO ( LANGUAGE , "portuguese" ) OR LIMIT-TO ( LANGUAGE , "czech" ) OR LIMIT-TO ( LANGUAGE , "polish" ) OR LIMIT-TO ( LANGUAGE , "turkish" ) OR LIMIT-TO ( LANGUAGE , "croatian" ) OR LIMIT-TO ( LANGUAGE , "japanese" ) OR LIMIT-TO ( LANGUAGE , "dutch" ) OR LIMIT-TO ( LANGUAGE , "persian" ) OR LIMIT-TO ( LANGUAGE , "ukrainian" ) OR LIMIT-TO ( LANGUAGE , "bosnian" ) OR LIMIT-TO ( LANGUAGE , "romanian" ) OR LIMIT-TO ( LANGUAGE , "slovenian" ) OR LIMIT-TO ( LANGUAGE , "hungarian" ) OR LIMIT-TO ( LANGUAGE , "afrikaans" ) OR LIMIT-TO ( LANGUAGE , "lithuanian" ) OR LIMIT-TO ( LANGUAGE , "moldavian" ) OR LIMIT-TO ( LANGUAGE , "moldovan" ) OR LIMIT-TO ( LANGUAGE , "norwegian" ) OR LIMIT-TO ( LANGUAGE , "slovak" ) OR LIMIT-TO ( LANGUAGE , "estonian" ) OR LIMIT-TO ( LANGUAGE , "hebrew" ) OR LIMIT-TO ( LANGUAGE , "swedish" ) OR LIMIT-TO ( LANGUAGE , "korean" ) OR LIMIT-TO ( LANGUAGE , "arabic" ) OR LIMIT-TO ( LANGUAGE , "serbian" ) OR LIMIT-TO ( LANGUAGE , "danish" ) OR LIMIT-TO ( LANGUAGE , "finnish" ) OR LIMIT-TO ( LANGUAGE , "catalan" ) OR LIMIT-TO ( LANGUAGE , "greek" ) OR LIMIT-TO ( LANGUAGE , "malay" ) OR LIMIT-TO ( LANGUAGE , "bulgarian" ) OR LIMIT-TO ( LANGUAGE , "undefined" ) ) | 1,524 results |

LOE Key Words

| **Search String** | **Results** | **Years** |
| --- | --- | --- |
| ( ( TITLE-ABS-KEY ( prévent* OR réduct* OR anti* OR désembriga* OR désendoctrin* OR désengage* OR réinser* OR ré-inser* OR récidiv* OR déradicalis* OR dé-radicalis* OR contre* OR réintégr* OR ré-intégr* OR renonc* OR désist* OR réhab* ) ) OR ( TITLE-ABS-KEY ( verhinder* OR reduzier* OR minder* OR bekämpf* OR loslösen* OR rehab* OR resozialisier* OR reintegrat* OR wiedereinglieder* OR abstrandn* OR unterlass* OR rückfall* OR rückfäll* OR deradikal* OR aussteige* OR aussstieg* ) ) OR ( TITLE-ABS-KEY ( предупре* OR профилактик* OR предотвра* OR снижени* OR снизи* OR уменьш* OR противодейств* OR противостоя* OR реабorт* OR реинтегр* OR рецидив* OR дерадикализ* ) ) OR ( TITLE-ABS-KEY ( prevent* OR forebyg* OR reducere* OR formindske OR counter* OR bekæmp* OR disengage* OR afhop* OR rehab* OR reintegrat* OR gen-integrat* OR re-entry OR genindtræde OR return* OR desist* OR afstå* OR "tage afstand fra" OR recidivism OR deradikal* OR tilbagfald OR de-radikal* OR afradikalisering OR exit* ) ) OR ( TITLE-ABS-KEY ( prevent* OR forhindre* OR reducere* OR reduser* OR counter* OR kamp OR disengage* OR afhop* OR rehab* AND eler AND reintegrat* OR genintegrer* OR re-entry OR retur* OR desist* OR avstå* OR "avstand fra" OR recidivism OR deradikal* OR tilbakefall OR de-radikal* OR avradikalisering OR exit* ) ) OR ( TITLE-ABS-KEY ( prevent* OR förhindra* OR reducere* OR minska* OR counter* OR bekämpa OR disengage* OR afhop* OR rehab* AND eler AND reintegrat* OR återintegrera* OR re-entry OR återinträde OR returnera* OR desist* OR avstå* OR "avstånd från" OR recidivism OR deradikal* OR "falla tillbaka" OR de-radikal* OR avradikal* OR avradikalisering OR exit* OR utgång ) ) ) AND ( ( TITLE-ABS-KEY ( politique OR initiative* OR interven* OR progra* OR plan* OR traitement* OR approche* OR modèl* OR stratégi* OR méthod* OR projet* OR pratique* OR instrument* OR outil* OR cadre* OR protocole* OR guide* OR échelle* OR système* OR inventaire* OR métrique* OR gabarit* OR profile* OR critère* OR questionnaire* OR réfer* OR évalu* OR "plan d’intervention" OR "plan de traitement" OR "plan de gestion" OR "programme de gestion" OR "plan d’encadrement" OR "plan d’action" OR "programme de traitement" OR "plan de soins" OR "programme de soins" OR "projet de traitement" OR "plan thérapeutique" OR "programme thérapeutique" OR "plan de soutien" OR "plan d’accompagnement" OR "gestion de cas" OR "gestion de dossier" OR "gestion de risque*" OR "gestion des risques" OR progrès* OR évolution* OR progression* OR supervision* OR observation* OR surveill* OR suivi* OR mesure* ) ) OR ( TITLE-ABS-KEY ( initiative* OR interven* OR programm* OR schema* OR behand* OR ansatz* OR modell* OR strateg* OR method* OR projekt* OR praxis* OR instrument* OR werkzeug* OR rahmen* OR protokoll* OR anleit* OR handlungsempfehlung* OR ausmaß* OR maßstab* OR system* OR erfinder* OR metri* OR vorlage* OR profil* OR kriteri* OR umfrage* OR einliefer* OR einweis* OR beurteil* OR einschätz* OR "Fallplan*" OR "Fallgestaltung*" OR "Managementplan*" OR "Behandlungsplan*" OR "Unterstützungsplan*" OR "Förderplan*" OR "Fallmanagement*" OR "Fallbearbeitung*" OR "Risikomanagement*" OR "Risikobewältigung*" OR forschritt* OR entwicklung* OR beobacht* OR überwach* OR beaufsichtig* OR aufsicht* OR aufseher* OR maßnahme* OR messen* ) ) OR ( TITLE-ABS-KEY ( initiativ* OR interven* OR program* OR tilgang OR policy OR policies OR behandling OR model* OR strateg* OR metod* OR projekt OR instrument* OR tool* OR redskab OR framework OR ramme OR protokol OR analy* OR guide* OR vejledning OR skala OR system* OR indeks OR beholdning OR matrix OR format* OR retningslin* OR template* OR skabelon OR profil OR kriterie* OR spørgeskema OR refer* OR henvisning OR assess* OR vurde* OR måling OR screening OR profil* OR "case plan*" OR "case formulering" OR "management plan*" OR "treatment plan*" OR "behandlingsplan" OR "behandlingsforløb" OR "behandlingsprogram" OR "support plan*" OR "støtteforløb" OR "case manage*" OR "Sagsbehandling*" OR "plan" OR "risikohåndtering*" OR "sikkerhedsvurdering" OR "sikkerhedsanalyse" OR progress OR fremskridt OR forbedringer OR monitor* OR supervis* OR måle* OR måling ) ) OR ( TITLE-ABS-KEY ( initiativ* OR intervensjon OR program* OR adgang OR policy OR behandle OR modell OR strateg* OR metod* OR prosjekt OR instrument* OR tool* OR verktøy OR framework OR rammeverk OR protokoll OR analyse OR guide* OR veiledning OR skala OR system OR indeks OR inventar OR matrix OR metrisk OR format* OR retningslinjer OR template* OR mal OR profil* OR kriterie* OR spørreskjema OR refer* OR referanse OR assess* OR vurdere* OR måling OR screening OR profil OR "case plan*" OR "Saksplan*" OR "management plan*" OR "treatment plan*" OR "behandlingsplan*" OR "behandlingsforløp" OR "behandlingsprogram" OR "support plan*" OR "Støttekurs" OR "case manage*" OR "Saksbehandling *" OR "plan" OR "risikohåndtering*" OR "Sikkerhetsvurdering" OR "sikkerhetsanalyse" OR progress OR fremgang OR forbedringer OR monitor* OR tilsyn* OR måle* OR mål ) ) OR ( TITLE-ABS-KEY ( initiativ* OR intervensjon OR program* OR tillgång OR policy OR policies OR behandla* OR model* OR strateg* OR metod* OR projekt OR instrument* OR tool* OR verktyg OR framework* OR ram OR protokoll OR analy* OR guide* OR vägledning OR skala OR system* OR indeks OR inventering OR matrix OR metrisk OR format* OR riktlinj* OR template* OR mall OR profil* OR kriterie* OR frågeformulär OR refer* OR referens OR assess* OR bedöm* OR mätning OR screen* OR "case plan*" OR "Fallformulering" OR "management plan*" OR "treatment plan*" OR "behandlingsplan*" OR "behandlingsförlopp" OR "behandlingsprogram" OR "support plan*" OR "case manage*" OR "plan" OR "Planera" OR "Riskhantering*" OR "Säkerhetsbedömning" OR "Säkerhetsanalys" OR progress OR framsteg OR förbättringar OR monitor* OR övervaka* OR mäta* OR mått ) ) ) AND ( ( TITLE-ABS-KEY ( terroris* OR radical* OR extremis* OR djihadi* OR islamis* OR salafi* OR "droite extrémiste" OR "droite extrême" OR néo-nazi OR néonazisme OR "extrême droite" OR nationalis* OR "suprématis* blanc" OR "gauche extrémiste" OR "gauche extrême" OR "extrême gauche" OR anarch* ) ) OR ( TITLE-ABS-KEY ( radikalisier* OR extremis* OR terroris* OR jihadi* OR dschihad* OR gotteskriegertum* OR islamis* OR salafi* OR rechts* OR "rechter Flügel*" OR rechtsterroris* OR rechtsextrem* OR "extreme Rechte" OR neonazi* OR rechtsaußen* OR rechtsaußen* OR nationalist* OR linksaußen* OR "weiße Vorherrschaft" OR "Überlegenheit der Weißen" OR "linker Flügel*" OR linksterroris* OR linksextrem AND * OR "extreme Linke" OR anarch* ) ) OR ( TITLE-ABS-KEY ( радикализ* OR экстреми* OR террори* OR джихади* OR ислами* OR салафи* OR правое-крыло OR "правое крыло" OR крайне-правый OR "крайне правый" OR неонацист OR националист* OR превосходства AND бел* OR превосходства AND белой AND расы OR левое-крыло OR "левое крыло" OR крайне-левый OR "крайне левый" OR анарх* OR единичн* ) ) OR ( TITLE-ABS-KEY ( radikali* OR ekstremis* OR terroris* OR jihadi* OR islamis* OR salafi* OR højreorienteret OR "højreorienteret" OR "ekstrem højreorienteret" OR højreekstrem OR nynazis* OR nationalis* OR "hvidt overherredømme" OR venstreorienteret OR "ekstrem venstreorienteret" OR anark* OR enkeltsag ) ) OR ( TITLE-ABS-KEY ( radikali* OR ekstremis* OR terroris* OR jihadi* OR islamis* OR salafi* OR "høyre ving" OR høyreekstreme OR nynazis* OR nasjonalistis* OR "hvit overherredømme" OR venstreorientert OR "ekstrem venstreorientert" OR anark* OR enkelttilfelle ) ) OR ( TITLE-ABS-KEY ( radikali* OR ekstremis* OR terroris* OR jihadi* OR islamis* OR salafi* OR "högra vingen" OR extremhöger OR nynazis* OR nationalis* OR "vit överlägsenhet" OR vänsterorienterad OR vänster OR extremvänster OR anark* OR "enskilda fall" ) ) ) AND ( LIMIT-TO ( LANGUAGE , "Chinese" ) OR LIMIT-TO ( LANGUAGE , "Russian" ) OR LIMIT-TO ( LANGUAGE , "German" ) OR LIMIT-TO ( LANGUAGE , "French" ) OR LIMIT-TO ( LANGUAGE , "Japanese" ) OR LIMIT-TO ( LANGUAGE , "Spanish" ) OR LIMIT-TO ( LANGUAGE , "Korean" ) OR LIMIT-TO ( LANGUAGE , "Italian" ) OR LIMIT-TO ( LANGUAGE , "Polish" ) OR LIMIT-TO ( LANGUAGE , "Portuguese" ) OR LIMIT-TO ( LANGUAGE , "Czech" ) OR LIMIT-TO ( LANGUAGE , "Turkish" ) OR LIMIT-TO ( LANGUAGE , "Persian" ) OR LIMIT-TO ( LANGUAGE , "Hungarian" ) OR LIMIT-TO ( LANGUAGE , "Ukrainian" ) OR LIMIT-TO ( LANGUAGE , "Dutch" ) OR LIMIT-TO ( LANGUAGE , "Croatian" ) OR LIMIT-TO ( LANGUAGE , "Slovak" ) OR LIMIT-TO ( LANGUAGE , "Serbian" ) OR LIMIT-TO ( LANGUAGE , "Romanian" ) OR LIMIT-TO ( LANGUAGE , "Bulgarian" ) OR LIMIT-TO ( LANGUAGE , "Moldavian" ) OR LIMIT-TO ( LANGUAGE , "Moldovan" ) OR LIMIT-TO ( LANGUAGE , "Slovenian" ) OR LIMIT-TO ( LANGUAGE , "Danish" ) OR LIMIT-TO ( LANGUAGE , "Bosnian" ) OR LIMIT-TO ( LANGUAGE , "Lithuanian" ) OR LIMIT-TO ( LANGUAGE , "Norwegian" ) OR LIMIT-TO ( LANGUAGE , "Greek" ) OR LIMIT-TO ( LANGUAGE , "Swedish" ) OR LIMIT-TO ( LANGUAGE , "Afrikaans" ) OR LIMIT-TO ( LANGUAGE , "Arabic" ) OR LIMIT-TO ( LANGUAGE , "Hebrew" ) OR LIMIT-TO ( LANGUAGE , "Estonian" ) OR LIMIT-TO ( LANGUAGE , "Finnish" ) OR LIMIT-TO ( LANGUAGE , "Malay" ) OR LIMIT-TO ( LANGUAGE , "catalan" ) OR LIMIT-TO ( LANGUAGE , "Azerbaijani" ) OR LIMIT-TO ( LANGUAGE , "Indonesian" ) OR LIMIT-TO ( LANGUAGE , "Latvian" ) OR LIMIT-TO ( LANGUAGE , "Albanian" ) OR LIMIT-TO ( LANGUAGE , "Icelandic" ) OR LIMIT-TO ( LANGUAGE , "Thai" ) OR LIMIT-TO ( LANGUAGE , "Belarusian" ) OR LIMIT-TO ( LANGUAGE , "Undefined" ) ) AND ( LIMIT-TO ( PUBYEAR , 2022 ) OR LIMIT-TO ( PUBYEAR , 2021 ) OR LIMIT-TO ( PUBYEAR , 2020 ) OR LIMIT-TO ( PUBYEAR , 2019 ) ) AND ( EXCLUDE ( SUBJAREA , "BIOC" ) OR EXCLUDE ( SUBJAREA , "ENVI" ) OR EXCLUDE ( SUBJAREA , "ENGI" ) OR EXCLUDE ( SUBJAREA , "CENG" ) OR EXCLUDE ( SUBJAREA , "MATE" ) OR EXCLUDE ( SUBJAREA , "PHYS" ) OR EXCLUDE ( SUBJAREA , "EART" ) OR EXCLUDE ( SUBJAREA , "ENER" ) ) AND ( EXCLUDE ( DOCTYPE , "ed" ) ) AND ( EXCLUDE ( EXACTKEYWORD , "Prostatectomy" ) OR EXCLUDE ( EXACTKEYWORD , "Nonhuman" ) OR EXCLUDE ( EXACTKEYWORD , "Prostate Cancer" ) OR EXCLUDE ( EXACTKEYWORD , "Prostatic Neoplasms" ) OR EXCLUDE ( EXACTKEYWORD , "Cancer Staging" ) OR EXCLUDE ( EXACTKEYWORD , "Prostate Specific Antigen" ) OR EXCLUDE ( EXACTKEYWORD , "Prostate Tumor" ) OR EXCLUDE ( EXACTKEYWORD , "Tumor Recurrence" ) OR EXCLUDE ( EXACTKEYWORD , "Cancer Patient" ) OR EXCLUDE ( EXACTKEYWORD , "Radical Prostatectomy" ) OR EXCLUDE ( EXACTKEYWORD , "Cancer Prognosis" ) OR EXCLUDE ( EXACTKEYWORD , "Prostate-Specific Antigen" ) OR EXCLUDE ( EXACTKEYWORD , "Antineoplastic Combined Chemotherapy Protocols" ) OR EXCLUDE ( EXACTKEYWORD , "Laparoscopy" ) OR EXCLUDE ( EXACTKEYWORD , "Lymph Node Dissection" ) OR EXCLUDE ( EXACTKEYWORD , "Cancer Recurrence" ) OR EXCLUDE ( EXACTKEYWORD , "Stomach Tumor" ) OR EXCLUDE ( EXACTKEYWORD , "Cancer Survival" ) OR EXCLUDE ( EXACTKEYWORD , "Gastrectomy" ) OR EXCLUDE ( EXACTKEYWORD , "Postoperative Period" ) OR EXCLUDE ( EXACTKEYWORD , "Cancer Radiotherapy" ) OR EXCLUDE ( EXACTKEYWORD , "Cancer Chemotherapy" ) OR EXCLUDE ( EXACTKEYWORD , "Lymph Node Metastasis" ) OR EXCLUDE ( EXACTKEYWORD , "Advanced Cancer" ) OR EXCLUDE ( EXACTKEYWORD , "Postoperative Complications" ) OR EXCLUDE ( EXACTKEYWORD , "Chemoradiotherapy" ) OR EXCLUDE ( EXACTKEYWORD , "Laparoscopic Surgery" ) OR EXCLUDE ( EXACTKEYWORD , "Adjuvant Chemotherapy" ) OR EXCLUDE ( EXACTKEYWORD , "Bladder Tumor" ) OR EXCLUDE ( EXACTKEYWORD , "Rat" ) OR EXCLUDE ( EXACTKEYWORD , "Surgery" ) OR EXCLUDE ( EXACTKEYWORD , "Animal Model" ) OR EXCLUDE ( EXACTKEYWORD , "Bladder Cancer" ) OR EXCLUDE ( EXACTKEYWORD , "Lymph Node Excision" ) OR EXCLUDE ( EXACTKEYWORD , "Adenocarcinoma" ) OR EXCLUDE ( EXACTKEYWORD , "Prostate" ) OR EXCLUDE ( EXACTKEYWORD , "Surgical Margin" ) OR EXCLUDE ( EXACTKEYWORD , "Cancer Hormone Therapy" ) OR EXCLUDE ( EXACTKEYWORD , "Rectum Tumor" ) OR EXCLUDE ( EXACTKEYWORD , "Tumor Invasion" ) OR EXCLUDE ( EXACTKEYWORD , "Cancer Grading" ) ) AND ( EXCLUDE ( SUBJAREA , "AGRI" ) ) | 1607 | 2022-2019 |
| ( ( TITLE-ABS-KEY ( prévent* OR réduct* OR anti* OR désembriga* OR désendoctrin* OR désengage* OR réinser* OR ré-inser* OR récidiv* OR déradicalis* OR dé-radicalis* OR contre* OR réintégr* OR ré-intégr* OR renonc* OR désist* OR réhab* ) ) OR ( TITLE-ABS-KEY ( verhinder* OR reduzier* OR minder* OR bekämpf* OR loslösen* OR rehab* OR resozialisier* OR reintegrat* OR wiedereinglieder* OR abstrandn* OR unterlass* OR rückfall* OR rückfäll* OR deradikal* OR aussteige* OR aussstieg* ) ) OR ( TITLE-ABS-KEY ( предупре* OR профилактик* OR предотвра* OR снижени* OR снизи* OR уменьш* OR противодейств* OR противостоя* OR реабorт* OR реинтегр* OR рецидив* OR дерадикализ* ) ) OR ( TITLE-ABS-KEY ( prevent* OR forebyg* OR reducere* OR formindske OR counter* OR bekæmp* OR disengage* OR afhop* OR rehab* OR reintegrat* OR gen-integrat* OR re-entry OR genindtræde OR return* OR desist* OR afstå* OR "tage afstand fra" OR recidivism OR deradikal* OR tilbagfald OR de-radikal* OR afradikalisering OR exit* ) ) OR ( TITLE-ABS-KEY ( prevent* OR forhindre* OR reducere* OR reduser* OR counter* OR kamp OR disengage* OR afhop* OR rehab* AND eler AND reintegrat* OR genintegrer* OR re-entry OR retur* OR desist* OR avstå* OR "avstand fra" OR recidivism OR deradikal* OR tilbakefall OR de-radikal* OR avradikalisering OR exit* ) ) OR ( TITLE-ABS-KEY ( prevent* OR förhindra* OR reducere* OR minska* OR counter* OR bekämpa OR disengage* OR afhop* OR rehab* AND eler AND reintegrat* OR återintegrera* OR re-entry OR återinträde OR returnera* OR desist* OR avstå* OR "avstånd från" OR recidivism OR deradikal* OR "falla tillbaka" OR de-radikal* OR avradikal* OR avradikalisering OR exit* OR utgång ) ) ) AND ( ( TITLE-ABS-KEY ( politique OR initiative* OR interven* OR progra* OR plan* OR traitement* OR approche* OR modèl* OR stratégi* OR méthod* OR projet* OR pratique* OR instrument* OR outil* OR cadre* OR protocole* OR guide* OR échelle* OR système* OR inventaire* OR métrique* OR gabarit* OR profile* OR critère* OR questionnaire* OR réfer* OR évalu* OR "plan d’intervention" OR "plan de traitement" OR "plan de gestion" OR "programme de gestion" OR "plan d’encadrement" OR "plan d’action" OR "programme de traitement" OR "plan de soins" OR "programme de soins" OR "projet de traitement" OR "plan thérapeutique" OR "programme thérapeutique" OR "plan de soutien" OR "plan d’accompagnement" OR "gestion de cas" OR "gestion de dossier" OR "gestion de risque*" OR "gestion des risques" OR progrès* OR évolution* OR progression* OR supervision* OR observation* OR surveill* OR suivi* OR mesure* ) ) OR ( TITLE-ABS-KEY ( initiative* OR interven* OR programm* OR schema* OR behand* OR ansatz* OR modell* OR strateg* OR method* OR projekt* OR praxis* OR instrument* OR werkzeug* OR rahmen* OR protokoll* OR anleit* OR handlungsempfehlung* OR ausmaß* OR maßstab* OR system* OR erfinder* OR metri* OR vorlage* OR profil* OR kriteri* OR umfrage* OR einliefer* OR einweis* OR beurteil* OR einschätz* OR "Fallplan*" OR "Fallgestaltung*" OR "Managementplan*" OR "Behandlungsplan*" OR "Unterstützungsplan*" OR "Förderplan*" OR "Fallmanagement*" OR "Fallbearbeitung*" OR "Risikomanagement*" OR "Risikobewältigung*" OR forschritt* OR entwicklung* OR beobacht* OR überwach* OR beaufsichtig* OR aufsicht* OR aufseher* OR maßnahme* OR messen* ) ) OR ( TITLE-ABS-KEY ( initiativ* OR interven* OR program* OR tilgang OR policy OR policies OR behandling OR model* OR strateg* OR metod* OR projekt OR instrument* OR tool* OR redskab OR framework OR ramme OR protokol OR analy* OR guide* OR vejledning OR skala OR system* OR indeks OR beholdning OR matrix OR format* OR retningslin* OR template* OR skabelon OR profil OR kriterie* OR spørgeskema OR refer* OR henvisning OR assess* OR vurde* OR måling OR screening OR profil* OR "case plan*" OR "case formulering" OR "management plan*" OR "treatment plan*" OR "behandlingsplan" OR "behandlingsforløb" OR "behandlingsprogram" OR "support plan*" OR "støtteforløb" OR "case manage*" OR "Sagsbehandling*" OR "plan" OR "risikohåndtering*" OR "sikkerhedsvurdering" OR "sikkerhedsanalyse" OR progress OR fremskridt OR forbedringer OR monitor* OR supervis* OR måle* OR måling ) ) OR ( TITLE-ABS-KEY ( initiativ* OR intervensjon OR program* OR adgang OR policy OR behandle OR modell OR strateg* OR metod* OR prosjekt OR instrument* OR tool* OR verktøy OR framework OR rammeverk OR protokoll OR analyse OR guide* OR veiledning OR skala OR system OR indeks OR inventar OR matrix OR metrisk OR format* OR retningslinjer OR template* OR mal OR profil* OR kriterie* OR spørreskjema OR refer* OR referanse OR assess* OR vurdere* OR måling OR screening OR profil OR "case plan*" OR "Saksplan*" OR "management plan*" OR "treatment plan*" OR "behandlingsplan*" OR "behandlingsforløp" OR "behandlingsprogram" OR "support plan*" OR "Støttekurs" OR "case manage*" OR "Saksbehandling *" OR "plan" OR "risikohåndtering*" OR "Sikkerhetsvurdering" OR "sikkerhetsanalyse" OR progress OR fremgang OR forbedringer OR monitor* OR tilsyn* OR måle* OR mål ) ) OR ( TITLE-ABS-KEY ( initiativ* OR intervensjon OR program* OR tillgång OR policy OR policies OR behandla* OR model* OR strateg* OR metod* OR projekt OR instrument* OR tool* OR verktyg OR framework* OR ram OR protokoll OR analy* OR guide* OR vägledning OR skala OR system* OR indeks OR inventering OR matrix OR metrisk OR format* OR riktlinj* OR template* OR mall OR profil* OR kriterie* OR frågeformulär OR refer* OR referens OR assess* OR bedöm* OR mätning OR screen* OR "case plan*" OR "Fallformulering" OR "management plan*" OR "treatment plan*" OR "behandlingsplan*" OR "behandlingsförlopp" OR "behandlingsprogram" OR "support plan*" OR "case manage*" OR "plan" OR "Planera" OR "Riskhantering*" OR "Säkerhetsbedömning" OR "Säkerhetsanalys" OR progress OR framsteg OR förbättringar OR monitor* OR övervaka* OR mäta* OR mått ) ) ) AND ( ( TITLE-ABS-KEY ( terroris* OR radical* OR extremis* OR djihadi* OR islamis* OR salafi* OR "droite extrémiste" OR "droite extrême" OR néo-nazi OR néonazisme OR "extrême droite" OR nationalis* OR "suprématis* blanc" OR "gauche extrémiste" OR "gauche extrême" OR "extrême gauche" OR anarch* ) ) OR ( TITLE-ABS-KEY ( radikalisier* OR extremis* OR terroris* OR jihadi* OR dschihad* OR gotteskriegertum* OR islamis* OR salafi* OR rechts* OR "rechter Flügel*" OR rechtsterroris* OR rechtsextrem* OR "extreme Rechte" OR neonazi* OR rechtsaußen* OR rechtsaußen* OR nationalist* OR linksaußen* OR "weiße Vorherrschaft" OR "Überlegenheit der Weißen" OR "linker Flügel*" OR linksterroris* OR linksextrem AND * OR "extreme Linke" OR anarch* ) ) OR ( TITLE-ABS-KEY ( радикализ* OR экстреми* OR террори* OR джихади* OR ислами* OR салафи* OR правое-крыло OR "правое крыло" OR крайне-правый OR "крайне правый" OR неонацист OR националист* OR превосходства AND бел* OR превосходства AND белой AND расы OR левое-крыло OR "левое крыло" OR крайне-левый OR "крайне левый" OR анарх* OR единичн* ) ) OR ( TITLE-ABS-KEY ( radikali* OR ekstremis* OR terroris* OR jihadi* OR islamis* OR salafi* OR højreorienteret OR "højreorienteret" OR "ekstrem højreorienteret" OR højreekstrem OR nynazis* OR nationalis* OR "hvidt overherredømme" OR venstreorienteret OR "ekstrem venstreorienteret" OR anark* OR enkeltsag ) ) OR ( TITLE-ABS-KEY ( radikali* OR ekstremis* OR terroris* OR jihadi* OR islamis* OR salafi* OR "høyre ving" OR høyreekstreme OR nynazis* OR nasjonalistis* OR "hvit overherredømme" OR venstreorientert OR "ekstrem venstreorientert" OR anark* OR enkelttilfelle ) ) OR ( TITLE-ABS-KEY ( radikali* OR ekstremis* OR terroris* OR jihadi* OR islamis* OR salafi* OR "högra vingen" OR extremhöger OR nynazis* OR nationalis* OR "vit överlägsenhet" OR vänsterorienterad OR vänster OR extremvänster OR anark* OR "enskilda fall" ) ) ) AND ( LIMIT-TO ( LANGUAGE , "Chinese" ) OR LIMIT-TO ( LANGUAGE , "Russian" ) OR LIMIT-TO ( LANGUAGE , "German" ) OR LIMIT-TO ( LANGUAGE , "French" ) OR LIMIT-TO ( LANGUAGE , "Japanese" ) OR LIMIT-TO ( LANGUAGE , "Spanish" ) OR LIMIT-TO ( LANGUAGE , "Korean" ) OR LIMIT-TO ( LANGUAGE , "Italian" ) OR LIMIT-TO ( LANGUAGE , "Polish" ) OR LIMIT-TO ( LANGUAGE , "Portuguese" ) OR LIMIT-TO ( LANGUAGE , "Czech" ) OR LIMIT-TO ( LANGUAGE , "Turkish" ) OR LIMIT-TO ( LANGUAGE , "Persian" ) OR LIMIT-TO ( LANGUAGE , "Hungarian" ) OR LIMIT-TO ( LANGUAGE , "Ukrainian" ) OR LIMIT-TO ( LANGUAGE , "Dutch" ) OR LIMIT-TO ( LANGUAGE , "Croatian" ) OR LIMIT-TO ( LANGUAGE , "Slovak" ) OR LIMIT-TO ( LANGUAGE , "Serbian" ) OR LIMIT-TO ( LANGUAGE , "Romanian" ) OR LIMIT-TO ( LANGUAGE , "Bulgarian" ) OR LIMIT-TO ( LANGUAGE , "Moldavian" ) OR LIMIT-TO ( LANGUAGE , "Moldovan" ) OR LIMIT-TO ( LANGUAGE , "Slovenian" ) OR LIMIT-TO ( LANGUAGE , "Danish" ) OR LIMIT-TO ( LANGUAGE , "Bosnian" ) OR LIMIT-TO ( LANGUAGE , "Lithuanian" ) OR LIMIT-TO ( LANGUAGE , "Norwegian" ) OR LIMIT-TO ( LANGUAGE , "Greek" ) OR LIMIT-TO ( LANGUAGE , "Swedish" ) OR LIMIT-TO ( LANGUAGE , "Afrikaans" ) OR LIMIT-TO ( LANGUAGE , "Arabic" ) OR LIMIT-TO ( LANGUAGE , "Hebrew" ) OR LIMIT-TO ( LANGUAGE , "Estonian" ) OR LIMIT-TO ( LANGUAGE , "Finnish" ) OR LIMIT-TO ( LANGUAGE , "Malay" ) OR LIMIT-TO ( LANGUAGE , "catalan" ) OR LIMIT-TO ( LANGUAGE , "Azerbaijani" ) OR LIMIT-TO ( LANGUAGE , "Indonesian" ) OR LIMIT-TO ( LANGUAGE , "Latvian" ) OR LIMIT-TO ( LANGUAGE , "Albanian" ) OR LIMIT-TO ( LANGUAGE , "Icelandic" ) OR LIMIT-TO ( LANGUAGE , "Thai" ) OR LIMIT-TO ( LANGUAGE , "Belarusian" ) OR LIMIT-TO ( LANGUAGE , "Undefined" ) ) AND ( LIMIT-TO ( PUBYEAR , 2018 ) OR LIMIT-TO ( PUBYEAR , 2017 ) OR LIMIT-TO ( PUBYEAR , 2016 ) OR LIMIT-TO ( PUBYEAR , 2015 ) ) AND ( EXCLUDE ( DOCTYPE , "ed" ) OR EXCLUDE ( DOCTYPE , "le" ) ) AND ( EXCLUDE ( SUBJAREA , "AGRI" ) OR EXCLUDE ( SUBJAREA , "CHEM" ) OR EXCLUDE ( SUBJAREA , "ENGI" ) OR EXCLUDE ( SUBJAREA , "CENG" ) OR EXCLUDE ( SUBJAREA , "ENVI" ) OR EXCLUDE ( SUBJAREA , "MATE" ) OR EXCLUDE ( SUBJAREA , "PHYS" ) OR EXCLUDE ( SUBJAREA , "EART" ) OR EXCLUDE ( SUBJAREA , "ENER" ) ) AND ( EXCLUDE ( EXACTKEYWORD , "Nonhuman" ) OR EXCLUDE ( EXACTKEYWORD , "Prostatectomy" ) OR EXCLUDE ( EXACTKEYWORD , "Prostatic Neoplasms" ) OR EXCLUDE ( EXACTKEYWORD , "Cancer Surgery" ) OR EXCLUDE ( EXACTKEYWORD , "Prostate Cancer" ) OR EXCLUDE ( EXACTKEYWORD , "Multimodality Cancer Therapy" ) OR EXCLUDE ( EXACTKEYWORD , "Prostate Specific Antigen" ) OR EXCLUDE ( EXACTKEYWORD , "Antineoplastic Combined Chemotherapy Protocols" ) OR EXCLUDE ( EXACTKEYWORD , "Neoplasm Recurrence, Local" ) OR EXCLUDE ( EXACTKEYWORD , "Neoplasm Staging" ) OR EXCLUDE ( EXACTKEYWORD , "Rat" ) OR EXCLUDE ( EXACTKEYWORD , "Lymph Node Metastasis" ) OR EXCLUDE ( EXACTKEYWORD , "Postoperative Complication" ) OR EXCLUDE ( EXACTKEYWORD , "Cancer Radiotherapy" ) OR EXCLUDE ( EXACTKEYWORD , "Postoperative Complications" ) OR EXCLUDE ( EXACTKEYWORD , "Lymph Node Dissection" ) OR EXCLUDE ( EXACTKEYWORD , "Prostate Tumor" ) OR EXCLUDE ( EXACTKEYWORD , "Prostate-Specific Antigen" ) OR EXCLUDE ( EXACTKEYWORD , "Animal Model" ) OR EXCLUDE ( EXACTKEYWORD , "Cancer Chemotherapy" ) OR EXCLUDE ( EXACTKEYWORD , "Lymphatic Metastasis" ) OR EXCLUDE ( EXACTKEYWORD , "Cancer Patient" ) OR EXCLUDE ( EXACTKEYWORD , "Cancer Recurrence" ) OR EXCLUDE ( EXACTKEYWORD , "Cancer Survival" ) OR EXCLUDE ( EXACTKEYWORD , "Stomach Neoplasms" ) OR EXCLUDE ( EXACTKEYWORD , "Cancer Prognosis" ) OR EXCLUDE ( EXACTKEYWORD , "Laparoscopy" ) OR EXCLUDE ( EXACTKEYWORD , "Postoperative Period" ) OR EXCLUDE ( EXACTKEYWORD , "Tumor Invasion" ) OR EXCLUDE ( EXACTKEYWORD , "Animal Tissue" ) OR EXCLUDE ( EXACTKEYWORD , "Chemoradiotherapy" ) OR EXCLUDE ( EXACTKEYWORD , "Radical Prostatectomy" ) OR EXCLUDE ( EXACTKEYWORD , "Radical Resection" ) OR EXCLUDE ( EXACTKEYWORD , "Gastrectomy" ) OR EXCLUDE ( EXACTKEYWORD , "Rats" ) OR EXCLUDE ( EXACTKEYWORD , "Rectal Neoplasms" ) OR EXCLUDE ( EXACTKEYWORD , "Chemotherapy, Adjuvant" ) OR EXCLUDE ( EXACTKEYWORD , "Cystectomy" ) OR EXCLUDE ( EXACTKEYWORD , "Adenocarcinoma" ) OR EXCLUDE ( EXACTKEYWORD , "Metastasis" ) OR EXCLUDE ( EXACTKEYWORD , "Neoplasm Invasiveness" ) OR EXCLUDE ( EXACTKEYWORD , "Tumor Marker" ) OR EXCLUDE ( EXACTKEYWORD , "Cancer Adjuvant Therapy" ) OR EXCLUDE ( EXACTKEYWORD , "Biopsy" ) OR EXCLUDE ( EXACTKEYWORD , "Breast Cancer" ) OR EXCLUDE ( EXACTKEYWORD , "Cancer Diagnosis" ) OR EXCLUDE ( EXACTKEYWORD , "Cancer Grading" ) OR EXCLUDE ( EXACTKEYWORD , "Urinary Bladder Neoplasms" ) OR EXCLUDE ( EXACTKEYWORD , "Biomarkers, Tumor" ) OR EXCLUDE ( EXACTKEYWORD , "Breast Neoplasms" ) OR EXCLUDE ( EXACTKEYWORD , "Chemotherapy" ) OR EXCLUDE ( EXACTKEYWORD , "Liver Neoplasms" ) ) | 1523 | 2018-2015 |
| ( ( TITLE-ABS-KEY ( prévent* OR réduct* OR anti* OR désembriga* OR désendoctrin* OR désengage* OR réinser* OR ré-inser* OR récidiv* OR déradicalis* OR dé-radicalis* OR contre* OR réintégr* OR ré-intégr* OR renonc* OR désist* OR réhab* ) ) OR ( TITLE-ABS-KEY ( verhinder* OR reduzier* OR minder* OR bekämpf* OR loslösen* OR rehab* OR resozialisier* OR reintegrat* OR wiedereinglieder* OR abstrandn* OR unterlass* OR rückfall* OR rückfäll* OR deradikal* OR aussteige* OR aussstieg* ) ) OR ( TITLE-ABS-KEY ( предупре* OR профилактик* OR предотвра* OR снижени* OR снизи* OR уменьш* OR противодейств* OR противостоя* OR реабorт* OR реинтегр* OR рецидив* OR дерадикализ* ) ) OR ( TITLE-ABS-KEY ( prevent* OR forebyg* OR reducere* OR formindske OR counter* OR bekæmp* OR disengage* OR afhop* OR rehab* OR reintegrat* OR gen-integrat* OR re-entry OR genindtræde OR return* OR desist* OR afstå* OR "tage afstand fra" OR recidivism OR deradikal* OR tilbagfald OR de-radikal* OR afradikalisering OR exit* ) ) OR ( TITLE-ABS-KEY ( prevent* OR forhindre* OR reducere* OR reduser* OR counter* OR kamp OR disengage* OR afhop* OR rehab* AND eler AND reintegrat* OR genintegrer* OR re-entry OR retur* OR desist* OR avstå* OR "avstand fra" OR recidivism OR deradikal* OR tilbakefall OR de-radikal* OR avradikalisering OR exit* ) ) OR ( TITLE-ABS-KEY ( prevent* OR förhindra* OR reducere* OR minska* OR counter* OR bekämpa OR disengage* OR afhop* OR rehab* AND eler AND reintegrat* OR återintegrera* OR re-entry OR återinträde OR returnera* OR desist* OR avstå* OR "avstånd från" OR recidivism OR deradikal* OR "falla tillbaka" OR de-radikal* OR avradikal* OR avradikalisering OR exit* OR utgång ) ) ) AND ( ( TITLE-ABS-KEY ( politique OR initiative* OR interven* OR progra* OR plan* OR traitement* OR approche* OR modèl* OR stratégi* OR méthod* OR projet* OR pratique* OR instrument* OR outil* OR cadre* OR protocole* OR guide* OR échelle* OR système* OR inventaire* OR métrique* OR gabarit* OR profile* OR critère* OR questionnaire* OR réfer* OR évalu* OR "plan d’intervention" OR "plan de traitement" OR "plan de gestion" OR "programme de gestion" OR "plan d’encadrement" OR "plan d’action" OR "programme de traitement" OR "plan de soins" OR "programme de soins" OR "projet de traitement" OR "plan thérapeutique" OR "programme thérapeutique" OR "plan de soutien" OR "plan d’accompagnement" OR "gestion de cas" OR "gestion de dossier" OR "gestion de risque*" OR "gestion des risques" OR progrès* OR évolution* OR progression* OR supervision* OR observation* OR surveill* OR suivi* OR mesure* ) ) OR ( TITLE-ABS-KEY ( initiative* OR interven* OR programm* OR schema* OR behand* OR ansatz* OR modell* OR strateg* OR method* OR projekt* OR praxis* OR instrument* OR werkzeug* OR rahmen* OR protokoll* OR anleit* OR handlungsempfehlung* OR ausmaß* OR maßstab* OR system* OR erfinder* OR metri* OR vorlage* OR profil* OR kriteri* OR umfrage* OR einliefer* OR einweis* OR beurteil* OR einschätz* OR "Fallplan*" OR "Fallgestaltung*" OR "Managementplan*" OR "Behandlungsplan*" OR "Unterstützungsplan*" OR "Förderplan*" OR "Fallmanagement*" OR "Fallbearbeitung*" OR "Risikomanagement*" OR "Risikobewältigung*" OR forschritt* OR entwicklung* OR beobacht* OR überwach* OR beaufsichtig* OR aufsicht* OR aufseher* OR maßnahme* OR messen* ) ) OR ( TITLE-ABS-KEY ( initiativ* OR interven* OR program* OR tilgang OR policy OR policies OR behandling OR model* OR strateg* OR metod* OR projekt OR instrument* OR tool* OR redskab OR framework OR ramme OR protokol OR analy* OR guide* OR vejledning OR skala OR system* OR indeks OR beholdning OR matrix OR format* OR retningslin* OR template* OR skabelon OR profil OR kriterie* OR spørgeskema OR refer* OR henvisning OR assess* OR vurde* OR måling OR screening OR profil* OR "case plan*" OR "case formulering" OR "management plan*" OR "treatment plan*" OR "behandlingsplan" OR "behandlingsforløb" OR "behandlingsprogram" OR "support plan*" OR "støtteforløb" OR "case manage*" OR "Sagsbehandling*" OR "plan" OR "risikohåndtering*" OR "sikkerhedsvurdering" OR "sikkerhedsanalyse" OR progress OR fremskridt OR forbedringer OR monitor* OR supervis* OR måle* OR måling ) ) OR ( TITLE-ABS-KEY ( initiativ* OR intervensjon OR program* OR adgang OR policy OR behandle OR modell OR strateg* OR metod* OR prosjekt OR instrument* OR tool* OR verktøy OR framework OR rammeverk OR protokoll OR analyse OR guide* OR veiledning OR skala OR system OR indeks OR inventar OR matrix OR metrisk OR format* OR retningslinjer OR template* OR mal OR profil* OR kriterie* OR spørreskjema OR refer* OR referanse OR assess* OR vurdere* OR måling OR screening OR profil OR "case plan*" OR "Saksplan*" OR "management plan*" OR "treatment plan*" OR "behandlingsplan*" OR "behandlingsforløp" OR "behandlingsprogram" OR "support plan*" OR "Støttekurs" OR "case manage*" OR "Saksbehandling *" OR "plan" OR "risikohåndtering*" OR "Sikkerhetsvurdering" OR "sikkerhetsanalyse" OR progress OR fremgang OR forbedringer OR monitor* OR tilsyn* OR måle* OR mål ) ) OR ( TITLE-ABS-KEY ( initiativ* OR intervensjon OR program* OR tillgång OR policy OR policies OR behandla* OR model* OR strateg* OR metod* OR projekt OR instrument* OR tool* OR verktyg OR framework* OR ram OR protokoll OR analy* OR guide* OR vägledning OR skala OR system* OR indeks OR inventering OR matrix OR metrisk OR format* OR riktlinj* OR template* OR mall OR profil* OR kriterie* OR frågeformulär OR refer* OR referens OR assess* OR bedöm* OR mätning OR screen* OR "case plan*" OR "Fallformulering" OR "management plan*" OR "treatment plan*" OR "behandlingsplan*" OR "behandlingsförlopp" OR "behandlingsprogram" OR "support plan*" OR "case manage*" OR "plan" OR "Planera" OR "Riskhantering*" OR "Säkerhetsbedömning" OR "Säkerhetsanalys" OR progress OR framsteg OR förbättringar OR monitor* OR övervaka* OR mäta* OR mått ) ) ) AND ( ( TITLE-ABS-KEY ( terroris* OR radical* OR extremis* OR djihadi* OR islamis* OR salafi* OR "droite extrémiste" OR "droite extrême" OR néo-nazi OR néonazisme OR "extrême droite" OR nationalis* OR "suprématis* blanc" OR "gauche extrémiste" OR "gauche extrême" OR "extrême gauche" OR anarch* ) ) OR ( TITLE-ABS-KEY ( radikalisier* OR extremis* OR terroris* OR jihadi* OR dschihad* OR gotteskriegertum* OR islamis* OR salafi* OR rechts* OR "rechter Flügel*" OR rechtsterroris* OR rechtsextrem* OR "extreme Rechte" OR neonazi* OR rechtsaußen* OR rechtsaußen* OR nationalist* OR linksaußen* OR "weiße Vorherrschaft" OR "Überlegenheit der Weißen" OR "linker Flügel*" OR linksterroris* OR linksextrem AND * OR "extreme Linke" OR anarch* ) ) OR ( TITLE-ABS-KEY ( радикализ* OR экстреми* OR террори* OR джихади* OR ислами* OR салафи* OR правое-крыло OR "правое крыло" OR крайне-правый OR "крайне правый" OR неонацист OR националист* OR превосходства AND бел* OR превосходства AND белой AND расы OR левое-крыло OR "левое крыло" OR крайне-левый OR "крайне левый" OR анарх* OR единичн* ) ) OR ( TITLE-ABS-KEY ( radikali* OR ekstremis* OR terroris* OR jihadi* OR islamis* OR salafi* OR højreorienteret OR "højreorienteret" OR "ekstrem højreorienteret" OR højreekstrem OR nynazis* OR nationalis* OR "hvidt overherredømme" OR venstreorienteret OR "ekstrem venstreorienteret" OR anark* OR enkeltsag ) ) OR ( TITLE-ABS-KEY ( radikali* OR ekstremis* OR terroris* OR jihadi* OR islamis* OR salafi* OR "høyre ving" OR høyreekstreme OR nynazis* OR nasjonalistis* OR "hvit overherredømme" OR venstreorientert OR "ekstrem venstreorientert" OR anark* OR enkelttilfelle ) ) OR ( TITLE-ABS-KEY ( radikali* OR ekstremis* OR terroris* OR jihadi* OR islamis* OR salafi* OR "högra vingen" OR extremhöger OR nynazis* OR nationalis* OR "vit överlägsenhet" OR vänsterorienterad OR vänster OR extremvänster OR anark* OR "enskilda fall" ) ) ) AND ( LIMIT-TO ( LANGUAGE , "Chinese" ) OR LIMIT-TO ( LANGUAGE , "Russian" ) OR LIMIT-TO ( LANGUAGE , "German" ) OR LIMIT-TO ( LANGUAGE , "French" ) OR LIMIT-TO ( LANGUAGE , "Japanese" ) OR LIMIT-TO ( LANGUAGE , "Spanish" ) OR LIMIT-TO ( LANGUAGE , "Korean" ) OR LIMIT-TO ( LANGUAGE , "Italian" ) OR LIMIT-TO ( LANGUAGE , "Polish" ) OR LIMIT-TO ( LANGUAGE , "Portuguese" ) OR LIMIT-TO ( LANGUAGE , "Czech" ) OR LIMIT-TO ( LANGUAGE , "Turkish" ) OR LIMIT-TO ( LANGUAGE , "Persian" ) OR LIMIT-TO ( LANGUAGE , "Hungarian" ) OR LIMIT-TO ( LANGUAGE , "Ukrainian" ) OR LIMIT-TO ( LANGUAGE , "Dutch" ) OR LIMIT-TO ( LANGUAGE , "Croatian" ) OR LIMIT-TO ( LANGUAGE , "Slovak" ) OR LIMIT-TO ( LANGUAGE , "Serbian" ) OR LIMIT-TO ( LANGUAGE , "Romanian" ) OR LIMIT-TO ( LANGUAGE , "Bulgarian" ) OR LIMIT-TO ( LANGUAGE , "Moldavian" ) OR LIMIT-TO ( LANGUAGE , "Moldovan" ) OR LIMIT-TO ( LANGUAGE , "Slovenian" ) OR LIMIT-TO ( LANGUAGE , "Danish" ) OR LIMIT-TO ( LANGUAGE , "Bosnian" ) OR LIMIT-TO ( LANGUAGE , "Lithuanian" ) OR LIMIT-TO ( LANGUAGE , "Norwegian" ) OR LIMIT-TO ( LANGUAGE , "Greek" ) OR LIMIT-TO ( LANGUAGE , "Swedish" ) OR LIMIT-TO ( LANGUAGE , "Afrikaans" ) OR LIMIT-TO ( LANGUAGE , "Arabic" ) OR LIMIT-TO ( LANGUAGE , "Hebrew" ) OR LIMIT-TO ( LANGUAGE , "Estonian" ) OR LIMIT-TO ( LANGUAGE , "Finnish" ) OR LIMIT-TO ( LANGUAGE , "Malay" ) OR LIMIT-TO ( LANGUAGE , "catalan" ) OR LIMIT-TO ( LANGUAGE , "Azerbaijani" ) OR LIMIT-TO ( LANGUAGE , "Indonesian" ) OR LIMIT-TO ( LANGUAGE , "Latvian" ) OR LIMIT-TO ( LANGUAGE , "Albanian" ) OR LIMIT-TO ( LANGUAGE , "Icelandic" ) OR LIMIT-TO ( LANGUAGE , "Thai" ) OR LIMIT-TO ( LANGUAGE , "Belarusian" ) OR LIMIT-TO ( LANGUAGE , "Undefined" ) ) AND ( LIMIT-TO ( PUBYEAR , 2014 ) OR LIMIT-TO ( PUBYEAR , 2013 ) OR LIMIT-TO ( PUBYEAR , 2012 ) OR LIMIT-TO ( PUBYEAR , 2011 ) OR LIMIT-TO ( PUBYEAR , 2010 ) ) AND ( EXCLUDE ( SUBJAREA , "AGRI" ) OR EXCLUDE ( SUBJAREA , "ENGI" ) OR EXCLUDE ( SUBJAREA , "CENG" ) OR EXCLUDE ( SUBJAREA , "ENVI" ) OR EXCLUDE ( SUBJAREA , "PHYS" ) OR EXCLUDE ( SUBJAREA , "EART" ) OR EXCLUDE ( SUBJAREA , "ENER" ) ) AND ( EXCLUDE ( DOCTYPE , "ed" ) OR EXCLUDE ( DOCTYPE , "le" ) ) AND ( EXCLUDE ( EXACTKEYWORD , "Rat" ) OR EXCLUDE ( EXACTKEYWORD , "Cancer Staging" ) OR EXCLUDE ( EXACTKEYWORD , "Prostatectomy" ) OR EXCLUDE ( EXACTKEYWORD , "Prostatic Neoplasms" ) OR EXCLUDE ( EXACTKEYWORD , "Prostate Cancer" ) OR EXCLUDE ( EXACTKEYWORD , "Rats" ) OR EXCLUDE ( EXACTKEYWORD , "Antineoplastic Combined Chemotherapy Protocols" ) OR EXCLUDE ( EXACTKEYWORD , "Cancer Chemotherapy" ) OR EXCLUDE ( EXACTKEYWORD , "Cancer Radiotherapy" ) OR EXCLUDE ( EXACTKEYWORD , "Animal Model" ) OR EXCLUDE ( EXACTKEYWORD , "Multimodality Cancer Therapy" ) OR EXCLUDE ( EXACTKEYWORD , "Prostate Tumor" ) OR EXCLUDE ( EXACTKEYWORD , "Cancer Survival" ) OR EXCLUDE ( EXACTKEYWORD , "Prostate Specific Antigen" ) OR EXCLUDE ( EXACTKEYWORD , "Lymph Node Metastasis" ) OR EXCLUDE ( EXACTKEYWORD , "Adjuvant Chemotherapy" ) OR EXCLUDE ( EXACTKEYWORD , "Animal Tissue" ) OR EXCLUDE ( EXACTKEYWORD , "Postoperative Complications" ) OR EXCLUDE ( EXACTKEYWORD , "Postoperative Complication" ) OR EXCLUDE ( EXACTKEYWORD , "Prostate-Specific Antigen" ) OR EXCLUDE ( EXACTKEYWORD , "Chemotherapy, Adjuvant" ) OR EXCLUDE ( EXACTKEYWORD , "Cancer Patient" ) OR EXCLUDE ( EXACTKEYWORD , "Breast Cancer" ) OR EXCLUDE ( EXACTKEYWORD , "Tumor Recurrence" ) OR EXCLUDE ( EXACTKEYWORD , "Radical Prostatectomy" ) OR EXCLUDE ( EXACTKEYWORD , "Mouse" ) OR EXCLUDE ( EXACTKEYWORD , "Cancer Recurrence" ) OR EXCLUDE ( EXACTKEYWORD , "Postoperative Period" ) OR EXCLUDE ( EXACTKEYWORD , "Cancer Prognosis" ) OR EXCLUDE ( EXACTKEYWORD , "Mastectomy" ) OR EXCLUDE ( EXACTKEYWORD , "Lymph Node Dissection" ) OR EXCLUDE ( EXACTKEYWORD , "Lymphatic Metastasis" ) OR EXCLUDE ( EXACTKEYWORD , "Cancer Adjuvant Therapy" ) OR EXCLUDE ( EXACTKEYWORD , "Laparoscopy" ) OR EXCLUDE ( EXACTKEYWORD , "Advanced Cancer" ) OR EXCLUDE ( EXACTKEYWORD , "Liver" ) OR EXCLUDE ( EXACTKEYWORD , "Tumor Marker" ) OR EXCLUDE ( EXACTKEYWORD , "Tumor Markers, Biological" ) OR EXCLUDE ( EXACTKEYWORD , "Adenocarcinoma" ) OR EXCLUDE ( EXACTKEYWORD , "Breast Neoplasms" ) OR EXCLUDE ( EXACTKEYWORD , "Chemotherapy" ) OR EXCLUDE ( EXACTKEYWORD , "Cystectomy" ) OR EXCLUDE ( EXACTKEYWORD , "Rats, Wistar" ) OR EXCLUDE ( EXACTKEYWORD , "Animal Cell" ) OR EXCLUDE ( EXACTKEYWORD , "Tumor Volume" ) OR EXCLUDE ( EXACTKEYWORD , "Stomach Neoplasms" ) OR EXCLUDE ( EXACTKEYWORD , "Wistar Rat" ) OR EXCLUDE ( EXACTKEYWORD , "Lymph Node Excision" ) OR EXCLUDE ( EXACTKEYWORD , "Mice" ) ) | 1672 | 2014-2010 |
| ( ( TITLE-ABS-KEY ( prévent* OR réduct* OR anti* OR désembriga* OR désendoctrin* OR désengage* OR réinser* OR ré-inser* OR récidiv* OR déradicalis* OR dé-radicalis* OR contre* OR réintégr* OR ré-intégr* OR renonc* OR désist* OR réhab* ) ) OR ( TITLE-ABS-KEY ( verhinder* OR reduzier* OR minder* OR bekämpf* OR loslösen* OR rehab* OR resozialisier* OR reintegrat* OR wiedereinglieder* OR abstrandn* OR unterlass* OR rückfall* OR rückfäll* OR deradikal* OR aussteige* OR aussstieg* ) ) OR ( TITLE-ABS-KEY ( предупре* OR профилактик* OR предотвра* OR снижени* OR снизи* OR уменьш* OR противодейств* OR противостоя* OR реабorт* OR реинтегр* OR рецидив* OR дерадикализ* ) ) OR ( TITLE-ABS-KEY ( prevent* OR forebyg* OR reducere* OR formindske OR counter* OR bekæmp* OR disengage* OR afhop* OR rehab* OR reintegrat* OR gen-integrat* OR re-entry OR genindtræde OR return* OR desist* OR afstå* OR "tage afstand fra" OR recidivism OR deradikal* OR tilbagfald OR de-radikal* OR afradikalisering OR exit* ) ) OR ( TITLE-ABS-KEY ( prevent* OR forhindre* OR reducere* OR reduser* OR counter* OR kamp OR disengage* OR afhop* OR rehab* AND eler AND reintegrat* OR genintegrer* OR re-entry OR retur* OR desist* OR avstå* OR "avstand fra" OR recidivism OR deradikal* OR tilbakefall OR de-radikal* OR avradikalisering OR exit* ) ) OR ( TITLE-ABS-KEY ( prevent* OR förhindra* OR reducere* OR minska* OR counter* OR bekämpa OR disengage* OR afhop* OR rehab* AND eler AND reintegrat* OR återintegrera* OR re-entry OR återinträde OR returnera* OR desist* OR avstå* OR "avstånd från" OR recidivism OR deradikal* OR "falla tillbaka" OR de-radikal* OR avradikal* OR avradikalisering OR exit* OR utgång ) ) ) AND ( ( TITLE-ABS-KEY ( politique OR initiative* OR interven* OR progra* OR plan* OR traitement* OR approche* OR modèl* OR stratégi* OR méthod* OR projet* OR pratique* OR instrument* OR outil* OR cadre* OR protocole* OR guide* OR échelle* OR système* OR inventaire* OR métrique* OR gabarit* OR profile* OR critère* OR questionnaire* OR réfer* OR évalu* OR "plan d’intervention" OR "plan de traitement" OR "plan de gestion" OR "programme de gestion" OR "plan d’encadrement" OR "plan d’action" OR "programme de traitement" OR "plan de soins" OR "programme de soins" OR "projet de traitement" OR "plan thérapeutique" OR "programme thérapeutique" OR "plan de soutien" OR "plan d’accompagnement" OR "gestion de cas" OR "gestion de dossier" OR "gestion de risque*" OR "gestion des risques" OR progrès* OR évolution* OR progression* OR supervision* OR observation* OR surveill* OR suivi* OR mesure* ) ) OR ( TITLE-ABS-KEY ( initiative* OR interven* OR programm* OR schema* OR behand* OR ansatz* OR modell* OR strateg* OR method* OR projekt* OR praxis* OR instrument* OR werkzeug* OR rahmen* OR protokoll* OR anleit* OR handlungsempfehlung* OR ausmaß* OR maßstab* OR system* OR erfinder* OR metri* OR vorlage* OR profil* OR kriteri* OR umfrage* OR einliefer* OR einweis* OR beurteil* OR einschätz* OR "Fallplan*" OR "Fallgestaltung*" OR "Managementplan*" OR "Behandlungsplan*" OR "Unterstützungsplan*" OR "Förderplan*" OR "Fallmanagement*" OR "Fallbearbeitung*" OR "Risikomanagement*" OR "Risikobewältigung*" OR forschritt* OR entwicklung* OR beobacht* OR überwach* OR beaufsichtig* OR aufsicht* OR aufseher* OR maßnahme* OR messen* ) ) OR ( TITLE-ABS-KEY ( initiativ* OR interven* OR program* OR tilgang OR policy OR policies OR behandling OR model* OR strateg* OR metod* OR projekt OR instrument* OR tool* OR redskab OR framework OR ramme OR protokol OR analy* OR guide* OR vejledning OR skala OR system* OR indeks OR beholdning OR matrix OR format* OR retningslin* OR template* OR skabelon OR profil OR kriterie* OR spørgeskema OR refer* OR henvisning OR assess* OR vurde* OR måling OR screening OR profil* OR "case plan*" OR "case formulering" OR "management plan*" OR "treatment plan*" OR "behandlingsplan" OR "behandlingsforløb" OR "behandlingsprogram" OR "support plan*" OR "støtteforløb" OR "case manage*" OR "Sagsbehandling*" OR "plan" OR "risikohåndtering*" OR "sikkerhedsvurdering" OR "sikkerhedsanalyse" OR progress OR fremskridt OR forbedringer OR monitor* OR supervis* OR måle* OR måling ) ) OR ( TITLE-ABS-KEY ( initiativ* OR intervensjon OR program* OR adgang OR policy OR behandle OR modell OR strateg* OR metod* OR prosjekt OR instrument* OR tool* OR verktøy OR framework OR rammeverk OR protokoll OR analyse OR guide* OR veiledning OR skala OR system OR indeks OR inventar OR matrix OR metrisk OR format* OR retningslinjer OR template* OR mal OR profil* OR kriterie* OR spørreskjema OR refer* OR referanse OR assess* OR vurdere* OR måling OR screening OR profil OR "case plan*" OR "Saksplan*" OR "management plan*" OR "treatment plan*" OR "behandlingsplan*" OR "behandlingsforløp" OR "behandlingsprogram" OR "support plan*" OR "Støttekurs" OR "case manage*" OR "Saksbehandling *" OR "plan" OR "risikohåndtering*" OR "Sikkerhetsvurdering" OR "sikkerhetsanalyse" OR progress OR fremgang OR forbedringer OR monitor* OR tilsyn* OR måle* OR mål ) ) OR ( TITLE-ABS-KEY ( initiativ* OR intervensjon OR program* OR tillgång OR policy OR policies OR behandla* OR model* OR strateg* OR metod* OR projekt OR instrument* OR tool* OR verktyg OR framework* OR ram OR protokoll OR analy* OR guide* OR vägledning OR skala OR system* OR indeks OR inventering OR matrix OR metrisk OR format* OR riktlinj* OR template* OR mall OR profil* OR kriterie* OR frågeformulär OR refer* OR referens OR assess* OR bedöm* OR mätning OR screen* OR "case plan*" OR "Fallformulering" OR "management plan*" OR "treatment plan*" OR "behandlingsplan*" OR "behandlingsförlopp" OR "behandlingsprogram" OR "support plan*" OR "case manage*" OR "plan" OR "Planera" OR "Riskhantering*" OR "Säkerhetsbedömning" OR "Säkerhetsanalys" OR progress OR framsteg OR förbättringar OR monitor* OR övervaka* OR mäta* OR mått ) ) ) AND ( ( TITLE-ABS-KEY ( terroris* OR radical* OR extremis* OR djihadi* OR islamis* OR salafi* OR "droite extrémiste" OR "droite extrême" OR néo-nazi OR néonazisme OR "extrême droite" OR nationalis* OR "suprématis* blanc" OR "gauche extrémiste" OR "gauche extrême" OR "extrême gauche" OR anarch* ) ) OR ( TITLE-ABS-KEY ( radikalisier* OR extremis* OR terroris* OR jihadi* OR dschihad* OR gotteskriegertum* OR islamis* OR salafi* OR rechts* OR "rechter Flügel*" OR rechtsterroris* OR rechtsextrem* OR "extreme Rechte" OR neonazi* OR rechtsaußen* OR rechtsaußen* OR nationalist* OR linksaußen* OR "weiße Vorherrschaft" OR "Überlegenheit der Weißen" OR "linker Flügel*" OR linksterroris* OR linksextrem AND * OR "extreme Linke" OR anarch* ) ) OR ( TITLE-ABS-KEY ( радикализ* OR экстреми* OR террори* OR джихади* OR ислами* OR салафи* OR правое-крыло OR "правое крыло" OR крайне-правый OR "крайне правый" OR неонацист OR националист* OR превосходства AND бел* OR превосходства AND белой AND расы OR левое-крыло OR "левое крыло" OR крайне-левый OR "крайне левый" OR анарх* OR единичн* ) ) OR ( TITLE-ABS-KEY ( radikali* OR ekstremis* OR terroris* OR jihadi* OR islamis* OR salafi* OR højreorienteret OR "højreorienteret" OR "ekstrem højreorienteret" OR højreekstrem OR nynazis* OR nationalis* OR "hvidt overherredømme" OR venstreorienteret OR "ekstrem venstreorienteret" OR anark* OR enkeltsag ) ) OR ( TITLE-ABS-KEY ( radikali* OR ekstremis* OR terroris* OR jihadi* OR islamis* OR salafi* OR "høyre ving" OR høyreekstreme OR nynazis* OR nasjonalistis* OR "hvit overherredømme" OR venstreorientert OR "ekstrem venstreorientert" OR anark* OR enkelttilfelle ) ) OR ( TITLE-ABS-KEY ( radikali* OR ekstremis* OR terroris* OR jihadi* OR islamis* OR salafi* OR "högra vingen" OR extremhöger OR nynazis* OR nationalis* OR "vit överlägsenhet" OR vänsterorienterad OR vänster OR extremvänster OR anark* OR "enskilda fall" ) ) ) AND ( LIMIT-TO ( LANGUAGE , "Chinese" ) OR LIMIT-TO ( LANGUAGE , "Russian" ) OR LIMIT-TO ( LANGUAGE , "German" ) OR LIMIT-TO ( LANGUAGE , "French" ) OR LIMIT-TO ( LANGUAGE , "Japanese" ) OR LIMIT-TO ( LANGUAGE , "Spanish" ) OR LIMIT-TO ( LANGUAGE , "Korean" ) OR LIMIT-TO ( LANGUAGE , "Italian" ) OR LIMIT-TO ( LANGUAGE , "Polish" ) OR LIMIT-TO ( LANGUAGE , "Portuguese" ) OR LIMIT-TO ( LANGUAGE , "Czech" ) OR LIMIT-TO ( LANGUAGE , "Turkish" ) OR LIMIT-TO ( LANGUAGE , "Persian" ) OR LIMIT-TO ( LANGUAGE , "Hungarian" ) OR LIMIT-TO ( LANGUAGE , "Ukrainian" ) OR LIMIT-TO ( LANGUAGE , "Dutch" ) OR LIMIT-TO ( LANGUAGE , "Croatian" ) OR LIMIT-TO ( LANGUAGE , "Slovak" ) OR LIMIT-TO ( LANGUAGE , "Serbian" ) OR LIMIT-TO ( LANGUAGE , "Romanian" ) OR LIMIT-TO ( LANGUAGE , "Bulgarian" ) OR LIMIT-TO ( LANGUAGE , "Moldavian" ) OR LIMIT-TO ( LANGUAGE , "Moldovan" ) OR LIMIT-TO ( LANGUAGE , "Slovenian" ) OR LIMIT-TO ( LANGUAGE , "Danish" ) OR LIMIT-TO ( LANGUAGE , "Bosnian" ) OR LIMIT-TO ( LANGUAGE , "Lithuanian" ) OR LIMIT-TO ( LANGUAGE , "Norwegian" ) OR LIMIT-TO ( LANGUAGE , "Greek" ) OR LIMIT-TO ( LANGUAGE , "Swedish" ) OR LIMIT-TO ( LANGUAGE , "Afrikaans" ) OR LIMIT-TO ( LANGUAGE , "Arabic" ) OR LIMIT-TO ( LANGUAGE , "Hebrew" ) OR LIMIT-TO ( LANGUAGE , "Estonian" ) OR LIMIT-TO ( LANGUAGE , "Finnish" ) OR LIMIT-TO ( LANGUAGE , "Malay" ) OR LIMIT-TO ( LANGUAGE , "catalan" ) OR LIMIT-TO ( LANGUAGE , "Azerbaijani" ) OR LIMIT-TO ( LANGUAGE , "Indonesian" ) OR LIMIT-TO ( LANGUAGE , "Latvian" ) OR LIMIT-TO ( LANGUAGE , "Albanian" ) OR LIMIT-TO ( LANGUAGE , "Icelandic" ) OR LIMIT-TO ( LANGUAGE , "Thai" ) OR LIMIT-TO ( LANGUAGE , "Belarusian" ) OR LIMIT-TO ( LANGUAGE , "Undefined" ) ) AND ( LIMIT-TO ( PUBYEAR , 2009 ) OR LIMIT-TO ( PUBYEAR , 2008 ) OR LIMIT-TO ( PUBYEAR , 2007 ) OR LIMIT-TO ( PUBYEAR , 2006 ) OR LIMIT-TO ( PUBYEAR , 2005 ) ) AND ( EXCLUDE ( SUBJAREA , "AGRI" ) OR EXCLUDE ( SUBJAREA , "ENGI" ) OR EXCLUDE ( SUBJAREA , "ENVI" ) OR EXCLUDE ( SUBJAREA , "CENG" ) OR EXCLUDE ( SUBJAREA , "MATE" ) OR EXCLUDE ( SUBJAREA , "PHYS" ) OR EXCLUDE ( SUBJAREA , "ENER" ) OR EXCLUDE ( SUBJAREA , "EART" ) ) AND ( EXCLUDE ( DOCTYPE , "ed" ) OR EXCLUDE ( DOCTYPE , "le" ) ) AND ( EXCLUDE ( EXACTKEYWORD , "Rat" ) OR EXCLUDE ( EXACTKEYWORD , "Cancer Staging" ) OR EXCLUDE ( EXACTKEYWORD , "Prostatectomy" ) OR EXCLUDE ( EXACTKEYWORD , "Prostate Cancer" ) OR EXCLUDE ( EXACTKEYWORD , "Animal Model" ) OR EXCLUDE ( EXACTKEYWORD , "Prostate Specific Antigen" ) OR EXCLUDE ( EXACTKEYWORD , "Prostatic Neoplasms" ) OR EXCLUDE ( EXACTKEYWORD , "Cancer Survival" ) OR EXCLUDE ( EXACTKEYWORD , "Cancer Radiotherapy" ) OR EXCLUDE ( EXACTKEYWORD , "Rats" ) OR EXCLUDE ( EXACTKEYWORD , "Cancer Surgery" ) OR EXCLUDE ( EXACTKEYWORD , "Metastasis" ) OR EXCLUDE ( EXACTKEYWORD , "Animal Tissue" ) OR EXCLUDE ( EXACTKEYWORD , "Antineoplastic Combined Chemotherapy Protocols" ) OR EXCLUDE ( EXACTKEYWORD , "Cancer Chemotherapy" ) OR EXCLUDE ( EXACTKEYWORD , "Lymph Node Metastasis" ) OR EXCLUDE ( EXACTKEYWORD , "Multimodality Cancer Therapy" ) OR EXCLUDE ( EXACTKEYWORD , "Prostate Tumor" ) OR EXCLUDE ( EXACTKEYWORD , "Surgical Technique" ) OR EXCLUDE ( EXACTKEYWORD , "Chemotherapy, Adjuvant" ) OR EXCLUDE ( EXACTKEYWORD , "Postoperative Period" ) OR EXCLUDE ( EXACTKEYWORD , "Postoperative Complication" ) OR EXCLUDE ( EXACTKEYWORD , "Adjuvant Chemotherapy" ) OR EXCLUDE ( EXACTKEYWORD , "Cancer Recurrence" ) OR EXCLUDE ( EXACTKEYWORD , "Mouse" ) OR EXCLUDE ( EXACTKEYWORD , "Radical Prostatectomy" ) OR EXCLUDE ( EXACTKEYWORD , "Cancer Invasion" ) OR EXCLUDE ( EXACTKEYWORD , "Prostate-Specific Antigen" ) OR EXCLUDE ( EXACTKEYWORD , "Tumor Recurrence" ) OR EXCLUDE ( EXACTKEYWORD , "Laparoscopy" ) OR EXCLUDE ( EXACTKEYWORD , "Adenocarcinoma" ) OR EXCLUDE ( EXACTKEYWORD , "Cystectomy" ) OR EXCLUDE ( EXACTKEYWORD , "Cancer Adjuvant Therapy" ) OR EXCLUDE ( EXACTKEYWORD , "Radiotherapy, Adjuvant" ) OR EXCLUDE ( EXACTKEYWORD , "Postoperative Complications" ) OR EXCLUDE ( EXACTKEYWORD , "Radiotherapy" ) OR EXCLUDE ( EXACTKEYWORD , "Lymphatic Metastasis" ) OR EXCLUDE ( EXACTKEYWORD , "Preoperative Evaluation" ) OR EXCLUDE ( EXACTKEYWORD , "Mastectomy" ) OR EXCLUDE ( EXACTKEYWORD , "Radiation Dose" ) OR EXCLUDE ( EXACTKEYWORD , "Animal Cell" ) OR EXCLUDE ( EXACTKEYWORD , "Postoperative Care" ) OR EXCLUDE ( EXACTKEYWORD , "Prostate Biopsy" ) OR EXCLUDE ( EXACTKEYWORD , "Tumor Volume" ) OR EXCLUDE ( EXACTKEYWORD , "Wistar Rat" ) OR EXCLUDE ( EXACTKEYWORD , "Lymph Node Excision" ) OR EXCLUDE ( EXACTKEYWORD , "Prostate" ) OR EXCLUDE ( EXACTKEYWORD , "Rats, Wistar" ) ) | 1718 | 2009-2005 |
| ( ( TITLE-ABS-KEY ( prévent* OR réduct* OR anti* OR désembriga* OR désendoctrin* OR désengage* OR réinser* OR ré-inser* OR récidiv* OR déradicalis* OR dé-radicalis* OR contre* OR réintégr* OR ré-intégr* OR renonc* OR désist* OR réhab* ) ) OR ( TITLE-ABS-KEY ( verhinder* OR reduzier* OR minder* OR bekämpf* OR loslösen* OR rehab* OR resozialisier* OR reintegrat* OR wiedereinglieder* OR abstrandn* OR unterlass* OR rückfall* OR rückfäll* OR deradikal* OR aussteige* OR aussstieg* ) ) OR ( TITLE-ABS-KEY ( предупре* OR профилактик* OR предотвра* OR снижени* OR снизи* OR уменьш* OR противодейств* OR противостоя* OR реабorт* OR реинтегр* OR рецидив* OR дерадикализ* ) ) OR ( TITLE-ABS-KEY ( prevent* OR forebyg* OR reducere* OR formindske OR counter* OR bekæmp* OR disengage* OR afhop* OR rehab* OR reintegrat* OR gen-integrat* OR re-entry OR genindtræde OR return* OR desist* OR afstå* OR "tage afstand fra" OR recidivism OR deradikal* OR tilbagfald OR de-radikal* OR afradikalisering OR exit* ) ) OR ( TITLE-ABS-KEY ( prevent* OR forhindre* OR reducere* OR reduser* OR counter* OR kamp OR disengage* OR afhop* OR rehab* AND eler AND reintegrat* OR genintegrer* OR re-entry OR retur* OR desist* OR avstå* OR "avstand fra" OR recidivism OR deradikal* OR tilbakefall OR de-radikal* OR avradikalisering OR exit* ) ) OR ( TITLE-ABS-KEY ( prevent* OR förhindra* OR reducere* OR minska* OR counter* OR bekämpa OR disengage* OR afhop* OR rehab* AND eler AND reintegrat* OR återintegrera* OR re-entry OR återinträde OR returnera* OR desist* OR avstå* OR "avstånd från" OR recidivism OR deradikal* OR "falla tillbaka" OR de-radikal* OR avradikal* OR avradikalisering OR exit* OR utgång ) ) ) AND ( ( TITLE-ABS-KEY ( politique OR initiative* OR interven* OR progra* OR plan* OR traitement* OR approche* OR modèl* OR stratégi* OR méthod* OR projet* OR pratique* OR instrument* OR outil* OR cadre* OR protocole* OR guide* OR échelle* OR système* OR inventaire* OR métrique* OR gabarit* OR profile* OR critère* OR questionnaire* OR réfer* OR évalu* OR "plan d’intervention" OR "plan de traitement" OR "plan de gestion" OR "programme de gestion" OR "plan d’encadrement" OR "plan d’action" OR "programme de traitement" OR "plan de soins" OR "programme de soins" OR "projet de traitement" OR "plan thérapeutique" OR "programme thérapeutique" OR "plan de soutien" OR "plan d’accompagnement" OR "gestion de cas" OR "gestion de dossier" OR "gestion de risque*" OR "gestion des risques" OR progrès* OR évolution* OR progression* OR supervision* OR observation* OR surveill* OR suivi* OR mesure* ) ) OR ( TITLE-ABS-KEY ( initiative* OR interven* OR programm* OR schema* OR behand* OR ansatz* OR modell* OR strateg* OR method* OR projekt* OR praxis* OR instrument* OR werkzeug* OR rahmen* OR protokoll* OR anleit* OR handlungsempfehlung* OR ausmaß* OR maßstab* OR system* OR erfinder* OR metri* OR vorlage* OR profil* OR kriteri* OR umfrage* OR einliefer* OR einweis* OR beurteil* OR einschätz* OR "Fallplan*" OR "Fallgestaltung*" OR "Managementplan*" OR "Behandlungsplan*" OR "Unterstützungsplan*" OR "Förderplan*" OR "Fallmanagement*" OR "Fallbearbeitung*" OR "Risikomanagement*" OR "Risikobewältigung*" OR forschritt* OR entwicklung* OR beobacht* OR überwach* OR beaufsichtig* OR aufsicht* OR aufseher* OR maßnahme* OR messen* ) ) OR ( TITLE-ABS-KEY ( initiativ* OR interven* OR program* OR tilgang OR policy OR policies OR behandling OR model* OR strateg* OR metod* OR projekt OR instrument* OR tool* OR redskab OR framework OR ramme OR protokol OR analy* OR guide* OR vejledning OR skala OR system* OR indeks OR beholdning OR matrix OR format* OR retningslin* OR template* OR skabelon OR profil OR kriterie* OR spørgeskema OR refer* OR henvisning OR assess* OR vurde* OR måling OR screening OR profil* OR "case plan*" OR "case formulering" OR "management plan*" OR "treatment plan*" OR "behandlingsplan" OR "behandlingsforløb" OR "behandlingsprogram" OR "support plan*" OR "støtteforløb" OR "case manage*" OR "Sagsbehandling*" OR "plan" OR "risikohåndtering*" OR "sikkerhedsvurdering" OR "sikkerhedsanalyse" OR progress OR fremskridt OR forbedringer OR monitor* OR supervis* OR måle* OR måling ) ) OR ( TITLE-ABS-KEY ( initiativ* OR intervensjon OR program* OR adgang OR policy OR behandle OR modell OR strateg* OR metod* OR prosjekt OR instrument* OR tool* OR verktøy OR framework OR rammeverk OR protokoll OR analyse OR guide* OR veiledning OR skala OR system OR indeks OR inventar OR matrix OR metrisk OR format* OR retningslinjer OR template* OR mal OR profil* OR kriterie* OR spørreskjema OR refer* OR referanse OR assess* OR vurdere* OR måling OR screening OR profil OR "case plan*" OR "Saksplan*" OR "management plan*" OR "treatment plan*" OR "behandlingsplan*" OR "behandlingsforløp" OR "behandlingsprogram" OR "support plan*" OR "Støttekurs" OR "case manage*" OR "Saksbehandling *" OR "plan" OR "risikohåndtering*" OR "Sikkerhetsvurdering" OR "sikkerhetsanalyse" OR progress OR fremgang OR forbedringer OR monitor* OR tilsyn* OR måle* OR mål ) ) OR ( TITLE-ABS-KEY ( initiativ* OR intervensjon OR program* OR tillgång OR policy OR policies OR behandla* OR model* OR strateg* OR metod* OR projekt OR instrument* OR tool* OR verktyg OR framework* OR ram OR protokoll OR analy* OR guide* OR vägledning OR skala OR system* OR indeks OR inventering OR matrix OR metrisk OR format* OR riktlinj* OR template* OR mall OR profil* OR kriterie* OR frågeformulär OR refer* OR referens OR assess* OR bedöm* OR mätning OR screen* OR "case plan*" OR "Fallformulering" OR "management plan*" OR "treatment plan*" OR "behandlingsplan*" OR "behandlingsförlopp" OR "behandlingsprogram" OR "support plan*" OR "case manage*" OR "plan" OR "Planera" OR "Riskhantering*" OR "Säkerhetsbedömning" OR "Säkerhetsanalys" OR progress OR framsteg OR förbättringar OR monitor* OR övervaka* OR mäta* OR mått ) ) ) AND ( ( TITLE-ABS-KEY ( terroris* OR radical* OR extremis* OR djihadi* OR islamis* OR salafi* OR "droite extrémiste" OR "droite extrême" OR néo-nazi OR néonazisme OR "extrême droite" OR nationalis* OR "suprématis* blanc" OR "gauche extrémiste" OR "gauche extrême" OR "extrême gauche" OR anarch* ) ) OR ( TITLE-ABS-KEY ( radikalisier* OR extremis* OR terroris* OR jihadi* OR dschihad* OR gotteskriegertum* OR islamis* OR salafi* OR rechts* OR "rechter Flügel*" OR rechtsterroris* OR rechtsextrem* OR "extreme Rechte" OR neonazi* OR rechtsaußen* OR rechtsaußen* OR nationalist* OR linksaußen* OR "weiße Vorherrschaft" OR "Überlegenheit der Weißen" OR "linker Flügel*" OR linksterroris* OR linksextrem AND * OR "extreme Linke" OR anarch* ) ) OR ( TITLE-ABS-KEY ( радикализ* OR экстреми* OR террори* OR джихади* OR ислами* OR салафи* OR правое-крыло OR "правое крыло" OR крайне-правый OR "крайне правый" OR неонацист OR националист* OR превосходства AND бел* OR превосходства AND белой AND расы OR левое-крыло OR "левое крыло" OR крайне-левый OR "крайне левый" OR анарх* OR единичн* ) ) OR ( TITLE-ABS-KEY ( radikali* OR ekstremis* OR terroris* OR jihadi* OR islamis* OR salafi* OR højreorienteret OR "højreorienteret" OR "ekstrem højreorienteret" OR højreekstrem OR nynazis* OR nationalis* OR "hvidt overherredømme" OR venstreorienteret OR "ekstrem venstreorienteret" OR anark* OR enkeltsag ) ) OR ( TITLE-ABS-KEY ( radikali* OR ekstremis* OR terroris* OR jihadi* OR islamis* OR salafi* OR "høyre ving" OR høyreekstreme OR nynazis* OR nasjonalistis* OR "hvit overherredømme" OR venstreorientert OR "ekstrem venstreorientert" OR anark* OR enkelttilfelle ) ) OR ( TITLE-ABS-KEY ( radikali* OR ekstremis* OR terroris* OR jihadi* OR islamis* OR salafi* OR "högra vingen" OR extremhöger OR nynazis* OR nationalis* OR "vit överlägsenhet" OR vänsterorienterad OR vänster OR extremvänster OR anark* OR "enskilda fall" ) ) ) AND ( LIMIT-TO ( LANGUAGE , "Chinese" ) OR LIMIT-TO ( LANGUAGE , "Russian" ) OR LIMIT-TO ( LANGUAGE , "German" ) OR LIMIT-TO ( LANGUAGE , "French" ) OR LIMIT-TO ( LANGUAGE , "Japanese" ) OR LIMIT-TO ( LANGUAGE , "Spanish" ) OR LIMIT-TO ( LANGUAGE , "Korean" ) OR LIMIT-TO ( LANGUAGE , "Italian" ) OR LIMIT-TO ( LANGUAGE , "Polish" ) OR LIMIT-TO ( LANGUAGE , "Portuguese" ) OR LIMIT-TO ( LANGUAGE , "Czech" ) OR LIMIT-TO ( LANGUAGE , "Turkish" ) OR LIMIT-TO ( LANGUAGE , "Persian" ) OR LIMIT-TO ( LANGUAGE , "Hungarian" ) OR LIMIT-TO ( LANGUAGE , "Ukrainian" ) OR LIMIT-TO ( LANGUAGE , "Dutch" ) OR LIMIT-TO ( LANGUAGE , "Croatian" ) OR LIMIT-TO ( LANGUAGE , "Slovak" ) OR LIMIT-TO ( LANGUAGE , "Serbian" ) OR LIMIT-TO ( LANGUAGE , "Romanian" ) OR LIMIT-TO ( LANGUAGE , "Bulgarian" ) OR LIMIT-TO ( LANGUAGE , "Moldavian" ) OR LIMIT-TO ( LANGUAGE , "Moldovan" ) OR LIMIT-TO ( LANGUAGE , "Slovenian" ) OR LIMIT-TO ( LANGUAGE , "Danish" ) OR LIMIT-TO ( LANGUAGE , "Bosnian" ) OR LIMIT-TO ( LANGUAGE , "Lithuanian" ) OR LIMIT-TO ( LANGUAGE , "Norwegian" ) OR LIMIT-TO ( LANGUAGE , "Greek" ) OR LIMIT-TO ( LANGUAGE , "Swedish" ) OR LIMIT-TO ( LANGUAGE , "Afrikaans" ) OR LIMIT-TO ( LANGUAGE , "Arabic" ) OR LIMIT-TO ( LANGUAGE , "Hebrew" ) OR LIMIT-TO ( LANGUAGE , "Estonian" ) OR LIMIT-TO ( LANGUAGE , "Finnish" ) OR LIMIT-TO ( LANGUAGE , "Malay" ) OR LIMIT-TO ( LANGUAGE , "catalan" ) OR LIMIT-TO ( LANGUAGE , "Azerbaijani" ) OR LIMIT-TO ( LANGUAGE , "Indonesian" ) OR LIMIT-TO ( LANGUAGE , "Latvian" ) OR LIMIT-TO ( LANGUAGE , "Albanian" ) OR LIMIT-TO ( LANGUAGE , "Icelandic" ) OR LIMIT-TO ( LANGUAGE , "Thai" ) OR LIMIT-TO ( LANGUAGE , "Belarusian" ) OR LIMIT-TO ( LANGUAGE , "Undefined" ) ) AND ( LIMIT-TO ( PUBYEAR , 2004 ) OR LIMIT-TO ( PUBYEAR , 2003 ) OR LIMIT-TO ( PUBYEAR , 2002 ) OR LIMIT-TO ( PUBYEAR , 2001 ) OR LIMIT-TO ( PUBYEAR , 2000 ) ) AND ( EXCLUDE ( SUBJAREA , "AGRI" ) OR EXCLUDE ( SUBJAREA , "ENGI" ) OR EXCLUDE ( SUBJAREA , "CENG" ) OR EXCLUDE ( SUBJAREA , "MATE" ) OR EXCLUDE ( SUBJAREA , "EART" ) OR EXCLUDE ( SUBJAREA , "ENVI" ) OR EXCLUDE ( SUBJAREA , "PHYS" ) OR EXCLUDE ( SUBJAREA , "ENER" ) ) AND ( EXCLUDE ( DOCTYPE , "ed" ) OR EXCLUDE ( DOCTYPE , "le" ) ) AND ( EXCLUDE ( EXACTKEYWORD , "Prostatectomy" ) OR EXCLUDE ( EXACTKEYWORD , "Rat" ) OR EXCLUDE ( EXACTKEYWORD , "Prostate Specific Antigen" ) OR EXCLUDE ( EXACTKEYWORD , "Cancer Radiotherapy" ) OR EXCLUDE ( EXACTKEYWORD , "Prostate Cancer" ) OR EXCLUDE ( EXACTKEYWORD , "Neoplasm Staging" ) OR EXCLUDE ( EXACTKEYWORD , "Cancer Survival" ) OR EXCLUDE ( EXACTKEYWORD , "Antineoplastic Combined Chemotherapy Protocols" ) OR EXCLUDE ( EXACTKEYWORD , "Multimodality Cancer Therapy" ) OR EXCLUDE ( EXACTKEYWORD , "Rats" ) OR EXCLUDE ( EXACTKEYWORD , "Metastasis" ) OR EXCLUDE ( EXACTKEYWORD , "Cancer Surgery" ) OR EXCLUDE ( EXACTKEYWORD , "Lymph Node Metastasis" ) OR EXCLUDE ( EXACTKEYWORD , "Surgical Technique" ) OR EXCLUDE ( EXACTKEYWORD , "Animal Model" ) OR EXCLUDE ( EXACTKEYWORD , "Cancer Recurrence" ) OR EXCLUDE ( EXACTKEYWORD , "Cancer Chemotherapy" ) OR EXCLUDE ( EXACTKEYWORD , "Postoperative Complication" ) OR EXCLUDE ( EXACTKEYWORD , "Prostate Tumor" ) OR EXCLUDE ( EXACTKEYWORD , "Chemotherapy, Adjuvant" ) OR EXCLUDE ( EXACTKEYWORD , "Neoplasm Recurrence, Local" ) OR EXCLUDE ( EXACTKEYWORD , "Lymphadenectomy" ) OR EXCLUDE ( EXACTKEYWORD , "Lymphatic Metastasis" ) OR EXCLUDE ( EXACTKEYWORD , "Tumor Recurrence" ) OR EXCLUDE ( EXACTKEYWORD , "Adenocarcinoma" ) OR EXCLUDE ( EXACTKEYWORD , "Mouse" ) OR EXCLUDE ( EXACTKEYWORD , "Adjuvant Chemotherapy" ) OR EXCLUDE ( EXACTKEYWORD , "Prostate-Specific Antigen" ) OR EXCLUDE ( EXACTKEYWORD , "Rats, Wistar" ) OR EXCLUDE ( EXACTKEYWORD , "Prostate Carcinoma" ) OR EXCLUDE ( EXACTKEYWORD , "Wistar Rat" ) OR EXCLUDE ( EXACTKEYWORD , "Cancer Adjuvant Therapy" ) OR EXCLUDE ( EXACTKEYWORD , "Animal Tissue" ) OR EXCLUDE ( EXACTKEYWORD , "Cancer Diagnosis" ) OR EXCLUDE ( EXACTKEYWORD , "Radical Prostatectomy" ) OR EXCLUDE ( EXACTKEYWORD , "Postoperative Period" ) OR EXCLUDE ( EXACTKEYWORD , "Cystectomy" ) OR EXCLUDE ( EXACTKEYWORD , "Lymph Node Excision" ) OR EXCLUDE ( EXACTKEYWORD , "Mastectomy" ) OR EXCLUDE ( EXACTKEYWORD , "Squamous Cell Carcinoma" ) OR EXCLUDE ( EXACTKEYWORD , "Postoperative Complications" ) OR EXCLUDE ( EXACTKEYWORD , "Cancer Invasion" ) OR EXCLUDE ( EXACTKEYWORD , "Tumor Marker" ) OR EXCLUDE ( EXACTKEYWORD , "Breast Neoplasms" ) OR EXCLUDE ( EXACTKEYWORD , "Carcinoma, Squamous Cell" ) OR EXCLUDE ( EXACTKEYWORD , "Mice" ) ) | 1270 | 2004-2000 |

**Table A1.4(b). Medline Search Records (English Key Words Only) – Part I (LOE)**

| **Ovid MEDLINE(R) ALL <1946 to September 26, 2022>** | | |
| --- | --- | --- |
| 1 | (radicali* or extremis* or terroris* or jihadi* or islamis* or salafi* or right-wing or "right wing" or extreme-right or "extreme right" or neo-nazi or far-right or "far right" or Nationalist* or "white supremacis*" or left-wing or "left wing" or extreme-left or "extreme left" or anarch* or "single issue" or single-issue).ab,hw,kf,kw,ot,sh,sy,ti,fx. | 16825 |
| 2 | (initiative* or interven* or program* or policy or policies or scheme* or treat* or approach* or model* or strateg* or method* or project* or practice* or instrument* or tool* or framework* or protocol* or guid* or scale* or system* or inventor* or metric* or template* or profile* or criteria or questionnaire* or refer* or assess* or "case plan*" or "case formulat*" or "management plan*" or "treatment plan*" or "support plan*" or "case manage*" or "risk manage*" or progress* or monitor* or supervis* or measur*).ab,hw,kf,ot,sh,sy,ti,fx. | 22640387 |
| 3 | (prevent* or reduc* or counter* or disengage* or rehab* or reintegrat* or re-integrat* or re-entry or reentry or desist* or recidivism or deradical* or de-radical* or exit*).ab,hw,kf,ot,sh,sy,ti,fx. | 6700903 |
| 4 | 1 and 2 and 3 | 4150 |
| 5 | limit 4 to (humans and yr="2000 - 2022" and (afrikaans or albanian or arabic or armenian or azerbaijani or belorussian or bengali or bosnian or bulgarian or burmese or catalan or chinese or croatian or czech or danish or dutch or esperanto or estonian or finnish or flemish or french or gaelic, scots or georgian or german or greek or hausa or hebrew or hindi or hungarian or icelandic or indonesian or interlingua or italian or japanese or kirghiz or korean or latin or latvian or lithuanian or macedonian or malay or marathi or masai or multilingual or norwegian or persian or polish or portuguese or pushto or rumanian or russian or serbian or slovak or slovene or spanish or swahili or swedish or tagalog or tamil or telugu or thai or turkish or ukrainian or undetermined or urdu or vietnamese or welsh)) | 283 |

**Table A1.5(b). PyscInfo Search Records (English Key Words Only) – Part II (LOE)**

| **Ovid MEDLINE(R) ALL <1946 to September 26, 2022>** | | |
| --- | --- | --- |
| 1 | (radicali* or extremis* or terroris* or jihadi* or islamis* or salafi* or right-wing or "right wing" or extreme-right or "extreme right" or neo-nazi or far-right or "far right" or Nationalist* or "white supremacis*" or left-wing or "left wing" or extreme-left or "extreme left" or anarch* or "single issue" or single-issue).ab,hw,id,mh,ot,ti. | 19323 |
| 2 | (initiative* or interven* or program* or policy or policies or scheme* or treat* or approach* or model* or strateg* or method* or project* or practice* or instrument* or tool* or framework* or protocol* or guid* or scale* or system* or inventor* or metric* or template* or profile* or criteria or questionnaire* or refer* or assess* or "case plan*" or "case formulat*" or "management plan*" or "treatment plan*" or "support plan*" or "case manage*" or "risk manage*" or progress* or monitor* or supervis* or measur*).ab,hw,id,mh,ot,ti. | 4164301 |
| 3 | (prevent* or reduc* or counter* or disengage* or rehab* or reintegrat* or re-integrat* or re-entry or reentry or desist* or recidivism or deradical* or de-radical* or exit*).ab,hw,id,mh,ot,ti. | 860475 |
| 4 | 1 and 2 and 3 | 3345 |
| 5 | limit 4 to (human and yr="2000 - 2022") | 3074 |
| 6 | limit 5 to (afrikaans or albanian or arabic or bulgarian or catalan or chinese or czech or danish or dutch or finnish or french or georgian or german or greek or hebrew or hindi or hungarian or iranian or italian or japanese or korean or lithuanian or malaysian or nonenglish or norwegian or polish or portuguese or romanian or russian or serbo croatian or slovak or slovene or spanish or swedish or turkish or ukrainian) | 113 |

**Table A1.6(b). Dissertation and Theses Global (English Key Words Only) – Part I (LOE)**

| **Set#** | **Searched for** | **Databases** | **Results** |
| --- | --- | --- | --- |
| S1 | ti(radicali* OR extremis* OR terroris* OR jihadi* OR islamis* OR salafi* OR right-wing OR "right wing" OR extreme-right OR "extreme right" OR neo-nazi OR far-right OR "far right" OR Nationalist* OR "white supremacis*" OR left-wing OR "left wing" OR extreme-left OR "extreme left" OR anarch* OR "single issue" OR single-issue) OR ab(radicali* OR extremis* OR terroris* OR jihadi* OR islamis* OR salafi* OR right-wing OR "right wing" OR extreme-right OR "extreme right" OR neo-nazi OR far-right OR "far right" OR Nationalist* OR "white supremacis*" OR left-wing OR "left wing" OR extreme-left OR "extreme left" OR anarch* OR "single issue" OR single-issue) OR mainsubject(radicali* OR extremis* OR terroris* OR jihadi* OR islamis* OR salafi* OR right-wing OR "right wing" OR extreme-right OR "extreme right" OR neo-nazi OR far-right OR "far right" OR Nationalist* OR "white supremacis*" OR left-wing OR "left wing" OR extreme-left OR "extreme left" OR anarch* OR "single issue" OR single-issue) OR diskw(radicali* OR extremis* OR terroris* OR jihadi* OR islamis* OR salafi* OR right-wing OR "right wing" OR extreme-right OR "extreme right" OR neo-nazi OR far-right OR "far right" OR Nationalist* OR "white supremacis*" OR left-wing OR "left wing" OR extreme-left OR "extreme left" OR anarch* OR "single issue" OR single-issue) AND pd(20000101-20221231) | ProQuest Dissertations & Theses Global | 21071 |
| S2 | ti(initiative* OR interven* OR program* OR policy OR policies OR scheme* OR treat* OR approach* OR model* OR strateg* OR method* OR project* OR practice* OR instrument* OR tool* OR framework* OR protocol* OR guid* OR scale* OR system* OR inventor* OR metric* OR template* OR profile* OR criteria OR questionnaire* OR refer* OR assess* OR "case plan*" OR "case formulat*" OR "management plan*" OR "treatment plan*" OR "support plan*" OR "case manage*" OR "risk manage*" OR progress* OR monitor* OR supervis* OR measur*) OR ab(initiative* OR interven* OR program* OR policy OR policies OR scheme* OR treat* OR approach* OR model* OR strateg* OR method* OR project* OR practice* OR instrument* OR tool* OR framework* OR protocol* OR guid* OR scale* OR system* OR inventor* OR metric* OR template* OR profile* OR criteria OR questionnaire* OR refer* OR assess* OR "case plan*" OR "case formulat*" OR "management plan*" OR "treatment plan*" OR "support plan*" OR "case manage*" OR "risk manage*" OR progress* OR monitor* OR supervis* OR measur*) OR mainsubject(initiative* OR interven* OR program* OR policy OR policies OR scheme* OR treat* OR approach* OR model* OR strateg* OR method* OR project* OR practice* OR instrument* OR tool* OR framework* OR protocol* OR guid* OR scale* OR system* OR inventor* OR metric* OR template* OR profile* OR criteria OR questionnaire* OR refer* OR assess* OR "case plan*" OR "case formulat*" OR "management plan*" OR "treatment plan*" OR "support plan*" OR "case manage*" OR "risk manage*" OR progress* OR monitor* OR supervis* OR measur*) OR diskw(initiative* OR interven* OR program* OR policy OR policies OR scheme* OR treat* OR approach* OR model* OR strateg* OR method* OR project* OR practice* OR instrument* OR tool* OR framework* OR protocol* OR guid* OR scale* OR system* OR inventor* OR metric* OR template* OR profile* OR criteria OR questionnaire* OR refer* OR assess* OR "case plan*" OR "case formulat*" OR "management plan*" OR "treatment plan*" OR "support plan*" OR "case manage*" OR "risk manage*" OR progress* OR monitor* OR supervis* OR measur*) AND pd(20000101-20221231) | ProQuest Dissertations & Theses Global | 2548800 |
| S3 | ti(prevent* OR reduc* OR counter* OR disengage* OR rehab* OR reintegrat* OR re-integrat* OR re-entry OR reentry OR desist* OR recidivism OR deradical* OR de-radical* OR exit*) OR ab(prevent* OR reduc* OR counter* OR disengage* OR rehab* OR reintegrat* OR re-integrat* OR re-entry OR reentry OR desist* OR recidivism OR deradical* OR de-radical* OR exit*) OR mainsubject(prevent* OR reduc* OR counter* OR disengage* OR rehab* OR reintegrat* OR re-integrat* OR re-entry OR reentry OR desist* OR recidivism OR deradical* OR de-radical* OR exit*) OR diskw(prevent* OR reduc* OR counter* OR disengage* OR rehab* OR reintegrat* OR re-integrat* OR re-entry OR reentry OR desist* OR recidivism OR deradical* OR de-radical* OR exit*) AND pd(20000101-20221231) | ProQuest Dissertations & Theses Global | 631885 |
| S4 | S1 AND S2 AND S3 | ProQuest Dissertations & Theses Global | 3670 |
| S5 | (S1 AND S2 AND S3) AND la.exact("CHI" OR "TUR" OR "POR" OR "SPA" OR "HEB" OR "FRE" OR "SWE" OR "GRE" OR "AFR" OR "FIN" OR "GER" OR "IND" OR "SRP" OR "CAT" OR "DUT" OR "HAW" OR "RUS" OR "SWA") | ProQuest Dissertations & Theses Global | 426 |

**Table A1.7(b). Sociological Abstracts (English Key Words Only) – Part I (LOE)**

| **Set#** | **Searched for** | **Databases** | **Results** |
| --- | --- | --- | --- |
| S1 | ti(radicali* OR extremis* OR terroris* OR jihadi* OR islamis* OR salafi* OR right-wing OR "right wing" OR extreme-right OR "extreme right" OR neo-nazi OR far-right OR "far right" OR Nationalist* OR "white supremacis*" OR left-wing OR "left wing" OR extreme-left OR "extreme left" OR anarch* OR "single issue" OR single-issue) OR ab(radicali* OR extremis* OR terroris* OR jihadi* OR islamis* OR salafi* OR right-wing OR "right wing" OR extreme-right OR "extreme right" OR neo-nazi OR far-right OR "far right" OR Nationalist* OR "white supremacis*" OR left-wing OR "left wing" OR extreme-left OR "extreme left" OR anarch* OR "single issue" OR single-issue) OR mainsubject(radicali* OR extremis* OR terroris* OR jihadi* OR islamis* OR salafi* OR right-wing OR "right wing" OR extreme-right OR "extreme right" OR neo-nazi OR far-right OR "far right" OR Nationalist* OR "white supremacis*" OR left-wing OR "left wing" OR extreme-left OR "extreme left" OR anarch* OR "single issue" OR single-issue) OR if(radicali* OR extremis* OR terroris* OR jihadi* OR islamis* OR salafi* OR right-wing OR "right wing" OR extreme-right OR "extreme right" OR neo-nazi OR far-right OR "far right" OR Nationalist* OR "white supremacis*" OR left-wing OR "left wing" OR extreme-left OR "extreme left" OR anarch* OR "single issue" OR single-issue) AND pd(20000101-20221231) | Sociological Abstracts | 37018 |
| S2 | ti(initiative* OR interven* OR program* OR policy OR policies OR scheme* OR treat* OR approach* OR model* OR strateg* OR method* OR project* OR practice* OR instrument* OR tool* OR framework* OR protocol* OR guid* OR scale* OR system* OR inventor* OR metric* OR template* OR profile* OR criteria OR questionnaire* OR refer* OR assess* OR "case plan*" OR "case formulat*" OR "management plan*" OR "treatment plan*" OR "support plan*" OR "case manage*" OR "risk manage*" OR progress* OR monitor* OR supervis* OR measur*) OR ab(initiative* OR interven* OR program* OR policy OR policies OR scheme* OR treat* OR approach* OR model* OR strateg* OR method* OR project* OR practice* OR instrument* OR tool* OR framework* OR protocol* OR guid* OR scale* OR system* OR inventor* OR metric* OR template* OR profile* OR criteria OR questionnaire* OR refer* OR assess* OR "case plan*" OR "case formulat*" OR "management plan*" OR "treatment plan*" OR "support plan*" OR "case manage*" OR "risk manage*" OR progress* OR monitor* OR supervis* OR measur*) OR mainsubject(initiative* OR interven* OR program* OR policy OR policies OR scheme* OR treat* OR approach* OR model* OR strateg* OR method* OR project* OR practice* OR instrument* OR tool* OR framework* OR protocol* OR guid* OR scale* OR system* OR inventor* OR metric* OR template* OR profile* OR criteria OR questionnaire* OR refer* OR assess* OR "case plan*" OR "case formulat*" OR "management plan*" OR "treatment plan*" OR "support plan*" OR "case manage*" OR "risk manage*" OR progress* OR monitor* OR supervis* OR measur*) OR if(initiative* OR interven* OR program* OR policy OR policies OR scheme* OR treat* OR approach* OR model* OR strateg* OR method* OR project* OR practice* OR instrument* OR tool* OR framework* OR protocol* OR guid* OR scale* OR system* OR inventor* OR metric* OR template* OR profile* OR criteria OR questionnaire* OR refer* OR assess* OR "case plan*" OR "case formulat*" OR "management plan*" OR "treatment plan*" OR "support plan*" OR "case manage*" OR "risk manage*" OR progress* OR monitor* OR supervis* OR measur*) AND pd(20000101-20221231) | Sociological Abstracts | 1022960 |
| S3 | ti(prevent* OR reduc* OR counter* OR disengage* OR rehab* OR reintegrat* OR re-integrat* OR re-entry OR reentry OR desist* OR recidivism OR deradical* OR de-radical* OR exit*) OR ab(prevent* OR reduc* OR counter* OR disengage* OR rehab* OR reintegrat* OR re-integrat* OR re-entry OR reentry OR desist* OR recidivism OR deradical* OR de-radical* OR exit*) OR mainsubject(prevent* OR reduc* OR counter* OR disengage* OR rehab* OR reintegrat* OR re-integrat* OR re-entry OR reentry OR desist* OR recidivism OR deradical* OR de-radical* OR exit*) OR if(prevent* OR reduc* OR counter* OR disengage* OR rehab* OR reintegrat* OR re-integrat* OR re-entry OR reentry OR desist* OR recidivism OR deradical* OR de-radical* OR exit*) AND pd(20000101-20221231) | Sociological Abstracts | 168992 |
| S4 | S1 AND S2 AND S3 | Sociological Abstracts | 3613 |
| S5 | (S1 AND S2 AND S3) AND la.exact("FRE" OR "GER" OR "SPA" OR "RUS" OR "SLA" OR "POR" OR "HRV" OR "TUR" OR "ITA" OR "CHI" OR "SRP" OR "POL" OR "SLV" OR "SLO" OR "ARA" OR "CZE" OR "DUT" OR "SWE" OR "BUL" OR "DAN" OR "HEB" OR "IND" OR "JPN" OR "LIT" OR "NOR") | Sociological Abstracts | 304 |

**Table A1.8(b). International Bibliography of the Social Sciences (English Key Words Only) – Part I (LOE)**

| **Set#** | **Searched for** | **Databases** | **Results** |
| --- | --- | --- | --- |
| S1 | ti(radicali* OR extremis* OR terroris* OR jihadi* OR islamis* OR salafi* OR right-wing OR "right wing" OR extreme-right OR "extreme right" OR neo-nazi OR far-right OR "far right" OR Nationalist* OR "white supremacis*" OR left-wing OR "left wing" OR extreme-left OR "extreme left" OR anarch* OR "single issue" OR single-issue) OR ab(radicali* OR extremis* OR terroris* OR jihadi* OR islamis* OR salafi* OR right-wing OR "right wing" OR extreme-right OR "extreme right" OR neo-nazi OR far-right OR "far right" OR Nationalist* OR "white supremacis*" OR left-wing OR "left wing" OR extreme-left OR "extreme left" OR anarch* OR "single issue" OR single-issue) OR mainsubject(radicali* OR extremis* OR terroris* OR jihadi* OR islamis* OR salafi* OR right-wing OR "right wing" OR extreme-right OR "extreme right" OR neo-nazi OR far-right OR "far right" OR Nationalist* OR "white supremacis*" OR left-wing OR "left wing" OR extreme-left OR "extreme left" OR anarch* OR "single issue" OR single-issue) AND pd(20000101-20221231) | International Bibliography of the Social Sciences (IBSS) | 89222 |
| S2 | ti(initiative* OR interven* OR program* OR policy OR policies OR scheme* OR treat* OR approach* OR model* OR strateg* OR method* OR project* OR practice* OR instrument* OR tool* OR framework* OR protocol* OR guid* OR scale* OR system* OR inventor* OR metric* OR template* OR profile* OR criteria OR questionnaire* OR refer* OR assess* OR "case plan*" OR "case formulat*" OR "management plan*" OR "treatment plan*" OR "support plan*" OR "case manage*" OR "risk manage*" OR progress* OR monitor* OR supervis* OR measur*) OR ab(initiative* OR interven* OR program* OR policy OR policies OR scheme* OR treat* OR approach* OR model* OR strateg* OR method* OR project* OR practice* OR instrument* OR tool* OR framework* OR protocol* OR guid* OR scale* OR system* OR inventor* OR metric* OR template* OR profile* OR criteria OR questionnaire* OR refer* OR assess* OR "case plan*" OR "case formulat*" OR "management plan*" OR "treatment plan*" OR "support plan*" OR "case manage*" OR "risk manage*" OR progress* OR monitor* OR supervis* OR measur*) OR mainsubject(initiative* OR interven* OR program* OR policy OR policies OR scheme* OR treat* OR approach* OR model* OR strateg* OR method* OR project* OR practice* OR instrument* OR tool* OR framework* OR protocol* OR guid* OR scale* OR system* OR inventor* OR metric* OR template* OR profile* OR criteria OR questionnaire* OR refer* OR assess* OR "case plan*" OR "case formulat*" OR "management plan*" OR "treatment plan*" OR "support plan*" OR "case manage*" OR "risk manage*" OR progress* OR monitor* OR supervis* OR measur*) AND pd(20000101-20221231) | International Bibliography of the Social Sciences (IBSS) | 2069494 |
| S3 | ti(prevent* OR reduc* OR counter* OR disengage* OR rehab* OR reintegrat* OR re-integrat* OR re-entry OR reentry OR desist* OR recidivism OR deradical* OR de-radical* OR exit*) OR ab(prevent* OR reduc* OR counter* OR disengage* OR rehab* OR reintegrat* OR re-integrat* OR re-entry OR reentry OR desist* OR recidivism OR deradical* OR de-radical* OR exit*) OR mainsubject(prevent* OR reduc* OR counter* OR disengage* OR rehab* OR reintegrat* OR re-integrat* OR re-entry OR reentry OR desist* OR recidivism OR deradical* OR de-radical* OR exit*) AND pd(20000101-20221231) | International Bibliography of the Social Sciences (IBSS) | 295819 |
| S4 | S1 AND S2 AND S3 | International Bibliography of the Social Sciences (IBSS) | 8240 |
| S5 | (S1 AND S2 AND S3) AND la.exact("FRE" OR "GER" OR "SPA" OR "RUS" OR "ITA" OR "TUR" OR "POL" OR "POR" OR "CZE" OR "HUN" OR "UKR" OR "FIN" OR "MAY" OR "SLO" OR "SLV" OR "CAT" OR "SRP" OR "HRV" OR "SWE" OR "CHI" OR "PER" OR "AFR" OR "ARA" OR "BOS" OR "DUT" OR "HEB" OR "KOR" OR "NOR" OR "SLA") | International Bibliography of the Social Sciences (IBSS) | 579 |
| S6 | (S1 AND S2 AND S3) NOT (at.exact("General Information" OR "Editorial") AND la.exact("FRE" OR "GER" OR "SPA" OR "RUS" OR "ITA" OR "TUR" OR "POL" OR "POR" OR "CZE" OR "HUN" OR "UKR" OR "FIN" OR "MAY" OR "SLO" OR "SLV" OR "CAT" OR "SRP" OR "HRV" OR "SWE" OR "CHI" OR "PER" OR "AFR" OR "ARA" OR "BOS" OR "DUT" OR "HEB" OR "KOR" OR "NOR" OR "SLA")) | International Bibliography of the Social Sciences (IBSS) | 569 |

**Table A1.9(b). Web of Science – Part I (LOE)**

English Key Words

| **Search string** | **Editions** | **Timespan** | **Results** |
| --- | --- | --- | --- |
| 6: #3 AND #2 AND #1 and Russian or Spanish or French or German or Turkish or Portuguese or Croatian or Italian or Chinese or Czech or Polish or Slovenian or Estonian or Ukrainian or Slovak or Malay or Afrikaans or Unspecified or Catalan or Norwegian or Lithuanian or Arabic or Dutch or Korean or Swedish or Bulgarian or Greek or Hungarian or Danish or Hebrew or Indonesian or Japanese (Languages) and Editorial Material or Book Review (Exclude – Document Types) | A&HCI , BKCI-SSH , ESCI , CPCI-SSH , SSCI | 4:24 PM | Exact search | 1,221 |
| 5: #3 AND #2 AND #1 and Russian or Spanish or French or German or Turkish or Portuguese or Croatian or Italian or Chinese or Czech or Polish or Slovenian or Estonian or Ukrainian or Slovak or Malay or Afrikaans or Unspecified or Catalan or Norwegian or Lithuanian or Arabic or Dutch or Korean or Swedish or Bulgarian or Greek or Hungarian or Danish or Hebrew or Indonesian or Japanese (Languages) | A&HCI , BKCI-SSH , ESCI , CPCI-SSH , SSCI | 4:24 PM | Exact search | 1,239 |
| 4: #3 AND #2 AND #1 | A&HCI , BKCI-SSH , ESCI , CPCI-SSH , SSCI | 4:23 PM | Exact search | 11,822 |
| 3: TS=(prevent* OR reduc* OR counter* OR disengage* OR rehab* OR reintegrat* OR re-integrat* OR re-entry OR reentry OR desist* OR recidivism OR deradical* OR de-radical* OR exit*) | A&HCI , BKCI-SSH , ESCI , CPCI-SSH , SSCI | 4:23 PM | Timespan: 2000-01-01 to 2022-12-31 (Publication Date) | Exact search | 1,637,308 |
| 2: TS=(initiative* OR interven* OR program* OR policy OR policies OR scheme* OR treat* OR approach* OR model* OR strateg* OR method* OR project* OR practice* OR instrument* OR tool* OR framework* OR protocol* OR guid* OR scale* OR system* OR inventor* OR metric* OR template* OR profile* OR criteria OR questionnaire* OR refer* OR assess* OR "case plan*" OR "case formulat*" OR "management plan*" OR "treatment plan*" OR "support plan*" OR "case manage*" OR "risk manage*" OR progress* OR monitor* OR supervis* OR measur*) | A&HCI , BKCI-SSH , ESCI , CPCI-SSH , SSCI | 4:23 PM | Timespan: 2000-01-01 to 2022-12-31 (Publication Date) | Exact search | 7,979,007 |
| 1: TS=(radicali* OR extremis* OR terroris* OR jihadi* OR islamis* OR salafi* OR right-wing OR "right wing" OR extreme-right OR "extreme right" OR neo-nazi OR far-right OR "far right" OR Nationalist* OR "white supremacis*" OR left-wing OR "left wing" OR extreme-left OR "extreme left" OR anarch* OR "single issue" OR single-issue) | A&HCI , BKCI-SSH , ESCI , CPCI-SSH , SSCI | 4:23 PM | Timespan: 2000-01-01 to 2022-12-31 (Publication Date) | Exact search | 85,108 |

LOE Keywords

| **Search string** | **Editions** | **Timespan** | **Results** |
| --- | --- | --- | --- |
| 1: TS=(terroris* OR radical* OR extremis* OR djihadi* OR islamis* OR salafi* OR "droite extrémiste" OR "droite extrême" OR néo-nazi OR néonazisme OR "extrême droite" OR nationalis* OR "suprématis* blanc" OR "gauche extrémiste" OR "gauche extrême" OR "extrême gauche" OR anarch*) | Editions: WOS.BHCI,WOS.SSCI,WOS.AHCI,WOS.ESCI,WOS.ISSHP | Timespan: 2000-01-01 to 2022-12-31 | Results: 181022 |
| 2: TS=(radikalisier* OR extremis* OR terroris* OR jihadi* OR dschihad* OR Gotteskriegertum* OR islamis* OR salafi* OR rechts* OR "rechter Flügel*" OR rechtsterroris* OR rechtsextrem* OR "extreme Rechte" OR neonazi* OR rechtsaußen* OR Rechtsaußen* OR Nationalist* OR linksaußen* OR "weiße Vorherrschaft" OR "Überlegenheit der Weißen" OR "linker Flügel*" OR linksterroris* OR linksextrem * OR "extreme Linke" OR anarch*) | Editions: WOS.BHCI,WOS.SSCI,WOS.AHCI,WOS.ESCI,WOS.ISSHP | Timespan: 2000-01-01 to 2022-12-31 | Results: 68213 |
| 3: TS=(радикализ* OR экстреми* OR террори* OR джихади* OR ислами* OR салафи* OR правое-крыло OR "правое крыло" OR крайне-правый OR "крайне правый" OR неонацист OR националист* OR превосходства бел* OR превосходства белой расы OR левое-крыло OR "левое крыло" OR крайне-левый OR "крайне левый" OR анарх* OR единичн*) | Editions: WOS.BHCI,WOS.SSCI,WOS.AHCI,WOS.ESCI,WOS.ISSHP | Timespan: 2000-01-01 to 2022-12-31 | Results: 0 |
| 4: TS=(radikali* OR ekstremis* OR terroris* OR jihadi* OR islamis* OR salafi* OR højreorienteret OR "højreorienteret" OR "ekstrem højreorienteret" OR højreekstrem OR nynazis* OR nationalis* OR "hvidt overherredømme" OR venstreorienteret OR "ekstrem venstreorienteret" OR anark* OR enkeltsag) | Editions: WOS.BHCI,WOS.SSCI,WOS.AHCI,WOS.ESCI,WOS.ISSHP | Timespan: 2000-01-01 to 2022-12-31 | Results: 84358 |
| 5: TS=(radikali* OR ekstremis* OR terroris* OR jihadi* OR islamis* OR salafi* OR "høyre ving" OR høyreekstreme OR nynazis* OR nasjonalistis* OR "hvit overherredømme" OR venstreorientert OR "ekstrem venstreorientert" OR anark* OR enkelttilfelle) | Editions: WOS.BHCI,WOS.SSCI,WOS.AHCI,WOS.ESCI,WOS.ISSHP | Timespan: 2000-01-01 to 2022-12-31 | Results: 41943 |
| 6: TS=(radikali* OR ekstremis* OR terroris* OR jihadi* OR islamis* OR salafi* OR "högra vingen" OR extremhöger OR nynazis* OR nationalis* OR "vit överlägsenhet" OR vänsterorienterad OR vänster OR extremvänster OR anark* OR "enskilda fall") | Editions: WOS.BHCI,WOS.SSCI,WOS.AHCI,WOS.ESCI,WOS.ISSHP | Timespan: 2000-01-01 to 2022-12-31 | Results: 84359 |
| 7: #6 OR #5 OR #4 OR #3 OR #2 OR #1 | Editions: WOS.BHCI,WOS.SSCI,WOS.AHCI,WOS.ESCI,WOS.ISSHP | Timespan: 2000-01-01 to 2022-12-31 | Results: 182001 |
| 8: TS=(politique OR initiative* OR interven* OR progra* OR plan* OR traitement* OR approche* OR modèl* OR stratégi* OR méthod* OR projet* OR pratique* OR instrument* OR outil* OR cadre* OR protocole* OR guide* OR échelle* OR système* OR inventaire* OR métrique* OR gabarit* OR profile* OR critère* OR questionnaire* OR réfer* OR évalu* OR "plan d’intervention" OR "plan de traitement" OR "plan de gestion" OR "programme de gestion" OR "plan d’encadrement" OR "plan d’action" OR "programme de traitement" OR "plan de soins" OR "programme de soins" OR "projet de traitement" OR "plan thérapeutique" OR "programme thérapeutique" OR "plan de soutien" OR "plan d’accompagnement" OR "gestion de cas" OR "gestion de dossier" OR "gestion de risque*" OR "gestion des risques" OR progrès* OR évolution* OR progression* OR supervision* OR observation* OR surveill* OR suivi* OR mesure*) | Editions: WOS.BHCI,WOS.SSCI,WOS.AHCI,WOS.ESCI,WOS.ISSHP | Timespan: 2000-01-01 to 2022-12-31 | Results: 6319347 |
| 9: TS=(initiative* OR interven* OR programm* OR schema* OR behand* OR Ansatz* OR modell* OR strateg* OR method* OR projekt* OR praxis* OR instrument* OR Werkzeug* OR Rahmen* OR protokoll* OR anleit* OR Handlungsempfehlung* OR Ausmaß* OR Maßstab* OR system* OR Erfinder* OR metri* OR Vorlage* OR Profil* OR Kriteri* OR Umfrage* OR Einliefer* OR Einweis* OR Beurteil* OR Einschätz* OR "Fallplan*" OR "Fallgestaltung*" OR "Managementplan*" OR "Behandlungsplan*" OR "Unterstützungsplan*" OR "Förderplan*" OR "Fallmanagement*" OR "Fallbearbeitung*" OR "Risikomanagement*" OR "Risikobewältigung*" OR Forschritt* OR Entwicklung* OR beobacht* OR überwach* OR beaufsichtig* OR Aufsicht* OR Aufseher* OR Maßnahme* OR Messen*) | Editions: WOS.BHCI,WOS.SSCI,WOS.AHCI,WOS.ESCI,WOS.ISSHP | Timespan: 2000-01-01 to 2022-12-31 | Results: 5055454 |
| 10: TS=(Инициатив* OR Вмешательств* OR программ* OR политик* OR схема* OR терап* OR подход* OR модель OR стратеги* метод* OR проект* OR практи* OR инструмент* OR концепци* OR структур* OR протокол* OR инструк* OR принцип* OR разработк* OR масштаб* OR системн* OR пример* OR обзор* OR критери* OR опросник* OR направлен* OR консульта* OR оцен* OR диагности* OR план* OR "план развития" OR мониторинг* OR "план ведения" OR "план* поддерж*" OR "управлени* риск*" OR развитие OR контрол* OR наблюд* OR оцен* OR измер*) | Editions: WOS.BHCI,WOS.SSCI,WOS.AHCI,WOS.ESCI,WOS.ISSHP | Timespan: 2000-01-01 to 2022-12-31 | Results: 0 |
| 11: TS=(initiativ* OR interven* OR program* OR tilgang OR policy OR policies OR behandling OR model* OR strateg* OR metod* OR projekt OR instrument* OR tool* OR redskab OR framework OR ramme OR protokol OR analy* OR guide* OR vejledning OR skala OR system* OR indeks OR beholdning OR matrix OR format* OR retningslin* OR template* OR skabelon OR profil OR kriterie* OR spørgeskema OR refer* OR henvisning OR assess* OR vurde* OR måling OR screening OR profil* OR "case plan*" OR "case formulering" OR "management plan*" OR "treatment plan*" OR "behandlingsplan" OR "behandlingsforløb" OR "behandlingsprogram"OR "support plan*" OR "støtteforløb" OR "case manage*" OR "Sagsbehandling*" OR "plan" OR "risikohåndtering*" OR "sikkerhedsvurdering" OR sikkerhedsanalyse OR progress OR fremskridt OR forbedringer OR monitor* OR supervis* OR måle* OR måling ) | Editions: WOS.BHCI,WOS.SSCI,WOS.AHCI,WOS.ESCI,WOS.ISSHP | Timespan: 2000-01-01 to 2022-12-31 | Results: 7387410 |
| 12: TS=(initiativ* OR intervensjon OR program* OR adgang OR policy OR behandle OR modell OR strateg* OR metod* OR prosjekt OR instrument* OR tool* OR verktøy OR framework OR rammeverk OR protokoll OR analyse OR guide* OR veiledning OR skala OR system OR indeks OR inventar OR matrix OR metrisk OR format* OR retningslinjer OR template* OR mal OR profil* OR kriterie* OR spørreskjema OR refer* OR referanse OR assess* OR vurdere* OR måling OR screening OR profil OR "case plan*" OR "Saksplan*" OR "management plan*" OR "treatment plan*" OR "behandlingsplan*" OR "behandlingsforløp" OR "behandlingsprogram"OR "support plan*" OR "Støttekurs" OR "case manage*" OR "Saksbehandling *" OR "plan" OR "risikohåndtering*" OR "Sikkerhetsvurdering" OR "sikkerhetsanalyse" OR progress OR fremgang OR forbedringer OR monitor* OR tilsyn* OR måle* OR mål ) | Editions: WOS.BHCI,WOS.SSCI,WOS.AHCI,WOS.ESCI,WOS.ISSHP | Timespan: 2000-01-01 to 2022-12-31 | Results: 5514319 |
| 13: TS=(initiativ* OR intervensjon OR program* OR tillgång OR policy OR policies OR behandla* OR model* OR strateg* OR metod* OR projekt OR instrument* OR tool* OR verktyg OR framework* OR ram OR protokoll OR analy* OR guide* OR vägledning OR skala OR system* OR indeks OR inventering OR matrix OR metrisk OR format* OR riktlinj* OR template* OR mall OR profil* OR kriterie* OR frågeformulär OR refer* OR referens OR assess* OR bedöm* OR mätning OR screen* OR "case plan*" OR "Fallformulering" OR "management plan*" OR "treatment plan*" OR "behandlingsplan*" OR "behandlingsförlopp" OR "behandlingsprogram"OR "support plan*" OR "case manage*" OR "plan" OR "Planera" OR "Riskhantering*" OR "Säkerhetsbedömning" OR "Säkerhetsanalys" OR progress OR framsteg OR förbättringar OR monitor* OR övervaka* OR mäta* OR mått) | Editions: WOS.BHCI,WOS.SSCI,WOS.AHCI,WOS.ESCI,WOS.ISSHP | Timespan: 2000-01-01 to 2022-12-31 | Results: 7201529 |
| 14: #8 OR #9 OR #10 OR #11 OR #12 OR #13 | Editions: WOS.BHCI,WOS.SSCI,WOS.AHCI,WOS.ESCI,WOS.ISSHP | Timespan: 2000-01-01 to 2022-12-31 | Results: 8054633 |
| 15: TS=(prévent* OR réduct* OR anti* OR désembriga* OR désendoctrin* OR désengage* OR réinser* OR ré-inser* OR récidiv* OR déradicalis* OR dé-radicalis* OR contre* OR réintégr* OR ré-intégr* OR renonc* OR désist* OR réhab*) | Editions: WOS.BHCI,WOS.SSCI,WOS.AHCI,WOS.ESCI,WOS.ISSHP | Timespan: 2000-01-01 to 2022-12-31 | Results: 1365067 |
| 16: TS=(verhinder* OR reduzier* OR minder* OR bekämpf* OR loslösen* OR rehab* OR resozialisier* OR reintegrat* OR wiedereinglieder* OR Abstrandn* OR unterlass* OR rückfall* OR Rückfäll* OR deradikal* OR aussteige* OR Aussstieg*) | Editions: WOS.BHCI,WOS.SSCI,WOS.AHCI,WOS.ESCI,WOS.ISSHP | Timespan: 2000-01-01 to 2022-12-31 | Results: 112145 |
| 17: TS=(предупре* OR профилактик* OR предотвра* OR снижени* OR снизи* OR уменьш* OR противодейств* OR противостоя* OR реабORт* OR реинтегр* OR рецидив* OR дерадикализ*) | Editions: WOS.BHCI,WOS.SSCI,WOS.AHCI,WOS.ESCI,WOS.ISSHP | Timespan: 2000-01-01 to 2022-12-31 | Results: 0 |
| 18: TS=(prevent* OR forebyg* OR reducere* OR formindske OR counter* OR bekæmp* OR disengage* OR afhop* OR rehab* OR reintegrat* OR gen-integrat* OR re-entry OR genindtræde OR return* OR desist* OR afstå* OR "tage afstand fra" OR recidivism OR deradikal* OR tilbagfald OR de-radikal* OR afradikalisering OR exit*) | Editions: WOS.BHCI,WOS.SSCI,WOS.AHCI,WOS.ESCI,WOS.ISSHP | Timespan: 2000-01-01 to 2022-12-31 | Results: 1004570 |
| 19: TS=(prevent* OR forhindre* OR reducere* OR reduser* OR counter* OR kamp OR disengage* OR afhop* OR rehab* ELER reintegrat* OR genintegrer* OR re-entry OR retur* OR desist* OR avstå* OR "avstand fra" OR recidivism OR deradikal* OR tilbakefall OR de-radikal* OR avradikalisering OR exit*) | Editions: WOS.BHCI,WOS.SSCI,WOS.AHCI,WOS.ESCI,WOS.ISSHP | Timespan: 2000-01-01 to 2022-12-31 | Results: 912707 |
| 20: TS=(prevent* OR förhindra* OR reducere* OR minska* OR counter* OR bekämpa OR disengage* OR afhop* OR rehab* ELER reintegrat* OR återintegrera* OR re-entry OR återinträde OR returnera* OR desist* OR avstå* OR "avstånd från" OR recidivism OR deradikal* OR "falla tillbaka" OR de-radikal* OR avradikal* OR avradikalisering OR exit* OR utgång) | Editions: WOS.BHCI,WOS.SSCI,WOS.AHCI,WOS.ESCI,WOS.ISSHP | Timespan: 2000-01-01 to 2022-12-31 | Results: 721778 |
| 21: #20 OR #19 OR #18 OR #17 OR #16 OR #15 | Editions: WOS.BHCI,WOS.SSCI,WOS.AHCI,WOS.ESCI,WOS.ISSHP | Timespan: 2000-01-01 to 2022-12-31 | Results: 1721877 |
| 22: #21 AND #14 AND #7 | Editions: WOS.BHCI,WOS.SSCI,WOS.AHCI,WOS.ESCI,WOS.ISSHP | Timespan: 2000-01-01 to 2022-12-31 | Results: 33847 |
| 23: #21 AND #14 AND #7 and Russian or Spanish or French or German or Portuguese or Turkish or Italian or Polish or Croatian or Chinese or Korean or Czech or Ukrainian or Slovenian or Slovak or Unspecified or Afrikaans or Indonesian or Dutch or Estonian or Lithuanian or Catalan or Greek or Malay or Swedish or Norwegian or Bulgarian or Arabic or Hungarian or Serbian or Japanese or Persian or Danish or Hebrew or Icelandic or Latvian or Welsh (Languages) | Editions: WOS.BHCI,WOS.SSCI,WOS.AHCI,WOS.ESCI,WOS.ISSHP | Timespan: 2000-01-01 to 2022-12-31 | Results: 4257 |
| 24: #21 AND #14 AND #7 and Russian or Spanish or French or German or Portuguese or Turkish or Italian or Polish or Croatian or Chinese or Korean or Czech or Ukrainian or Slovenian or Slovak or Unspecified or Afrikaans or Indonesian or Dutch or Estonian or Lithuanian or Catalan or Greek or Malay or Swedish or Norwegian or Bulgarian or Arabic or Hungarian or Serbian or Japanese or Persian or Danish or Hebrew or Icelandic or Latvian or Welsh (Languages) and Book Review or Editorial Material (Exclude – Document Types) | Editions: WOS.BHCI,WOS.SSCI,WOS.AHCI,WOS.ESCI,WOS.ISSHP | Timespan: 2000-01-01 to 2022-12-31 | Results: 4204 |
| 25: #21 AND #14 AND #7 and Russian or Spanish or French or German or Portuguese or Turkish or Italian or Polish or Croatian or Chinese or Korean or Czech or Ukrainian or Slovenian or Slovak or Unspecified or Afrikaans or Indonesian or Dutch or Estonian or Lithuanian or Catalan or Greek or Malay or Swedish or Norwegian or Bulgarian or Arabic or Hungarian or Serbian or Japanese or Persian or Danish or Hebrew or Icelandic or Latvian or Welsh (Languages) and Book Review or Editorial Material (Exclude – Document Types) and Oncology or Chemistry Multidisciplinary or Urology Nephrology or Geography or Engineering Chemical or Architecture or Surgery or Engineering Multidisciplinary or Food Science Technology or Environmental Sciences or Orthopedics or Meteorology Atmospheric Sciences or Water Resources or Tropical Medicine or Physics Multidisciplinary or Materials Science Composites or Materials Science Coatings Films or Materials Science Characterization Testing or Hematology or Geology or Forestry or Ergonomics or Engineering Petroleum or Engineering Marine or Crystallography or Construction Building Technology or Physics Nuclear or Microbiology or Otorhinolaryngology or Chemistry Analytical or Transplantation or Engineering Mechanical or Marine Freshwater Biology or Geosciences Multidisciplinary or Fisheries or Engineering Geological or Engineering Civil or Electrochemistry or Biochemistry Molecular Biology or Agricultural Economics Policy or Geography Physical or Peripheral Vascular Disease or Dermatology or Gastroenterology Hepatology or Nutrition Dietetics or Biotechnology Applied Microbiology or Engineering Electrical Electronic (Exclude – Web of Science Categories) | Editions: WOS.BHCI,WOS.SSCI,WOS.AHCI,WOS.ESCI,WOS.ISSHP | Timespan: 2000-01-01 to 2022-12-31 | Results: 3708 |
| 26: #21 AND #14 AND #7 and Russian or Spanish or French or German or Portuguese or Turkish or Italian or Polish or Croatian or Chinese or Korean or Czech or Ukrainian or Slovenian or Slovak or Unspecified or Afrikaans or Indonesian or Dutch or Estonian or Lithuanian or Catalan or Greek or Malay or Swedish or Norwegian or Bulgarian or Arabic or Hungarian or Serbian or Japanese or Persian or Danish or Hebrew or Icelandic or Latvian or Welsh (Languages) and Book Review or Editorial Material (Exclude – Document Types) and Oncology or Chemistry Multidisciplinary or Urology Nephrology or Geography or Engineering Chemical or Architecture or Surgery or Engineering Multidisciplinary or Food Science Technology or Environmental Sciences or Orthopedics or Meteorology Atmospheric Sciences or Water Resources or Tropical Medicine or Physics Multidisciplinary or Materials Science Composites or Materials Science Coatings Films or Materials Science Characterization Testing or Hematology or Geology or Forestry or Ergonomics or Engineering Petroleum or Engineering Marine or Crystallography or Construction Building Technology or Physics Nuclear or Microbiology or Otorhinolaryngology or Chemistry Analytical or Transplantation or Engineering Mechanical or Marine Freshwater Biology or Geosciences Multidisciplinary or Fisheries or Engineering Geological or Engineering Civil or Electrochemistry or Biochemistry Molecular Biology or Agricultural Economics Policy or Geography Physical or Peripheral Vascular Disease or Dermatology or Gastroenterology Hepatology or Nutrition Dietetics or Biotechnology Applied Microbiology or Engineering Electrical Electronic (Exclude – Web of Science Categories) and Agriculture or Respiratory System or Materials Science or Anesthesiology (Exclude – Research Areas) | Editions: WOS.BHCI,WOS.SSCI,WOS.AHCI,WOS.ESCI,WOS.ISSHP | Timespan: 2000-01-01 to 2022-12-31 | Results: 3642 |

**Appendix I (C) Part II Search Record (English Language)**

**Table A1.1(c) Academic Platform Search Record – Part II**

| **Search Source** | **Source** | **Search Date)** | **Date Coverage for Search** | **Results** |
| --- | --- | --- | --- | --- |
| Criminal Justice Abstracts | EBSCO | 25/09/2022 | 01/01/2000 - 31/08/2022 | 2428 |
| Scopus | Elsevier | 25/09/2022 | 01/01/2000 - 31/08/2022 | 23,558 |
| CINCH: Australian Criminology Database | Informit | 25/09/2022 | 01/01/2000 - 31/08/2022 | 591 |
| Medline | Ovid | 25/09/2022 | 01/01/2000 - 31/08/2022 | 11,538 |
| PsycInfo | Ovid | 25/09/2022 | 01/01/2000 - 31/08/2022 | 5,644 |
| Dissertations and Theses Global | ProQuest | 25/09/2022 | 01/01/2000 - 31/08/2022 | 1,677 |
| International Bibliography of the Social Sciences | ProQuest | 25/09/2022 | 01/01/2000 - 31/08/2022 | 1611 |
| Sociological Abstracts | ProQuest | 25/09/2022 | 01/01/2000 - 31/08/2022 | 2086 |
| Book Citation Index – Social Sciences & Humanities (BKCI-SSH)  Social Sciences Citation Index (SSCI)  Arts & Humanities Citation Index (A&HCI)  Emerging Sources Citation Index (ESCI)  Conference Proceedings Citation Index – Social Science & Humanities (CPCI-SSH) | Web of Science | 25/09/2022 | 01/01/2000 - 31/08/2022 | 6739 |

**Table A1.2(c). Criminal Justice Abstracts Search Record – Part II**

| # | **Query** | **Limiters/Expanders** | **Last Run Via** | **Results** |
| --- | --- | --- | --- | --- |
| S5 | S1 AND S2 AND S3 AND S4 | Limiters - Publication Date: 20000101-20221231 | Interface - EBSCOhost Research Databases | 2,428 |
|  |  | Search modes - Boolean/Phrase | Search Screen - Advanced Search |  |
|  |  |  | Database - Criminal Justice Abstracts |  |
| S4 | TI ( review* OR meta-analy* ) OR AB ( review* OR meta-analy* ) OR KW ( review* OR meta-analy* ) OR SU ( review* OR meta-analy* ) | Limiters - Publication Date: 20000101-20221231 | Interface - EBSCOhost Research Databases | 63,945 |
|  |  | Search modes - Boolean/Phrase | Search Screen - Advanced Search |  |
|  |  |  | Database - Criminal Justice Abstracts |  |
| S3 | TI ( prevent* OR reduc* OR counter* OR disengage* OR rehab* OR reintegrat* OR re-integrat* OR re-entry OR reentry OR desist* OR recidivism OR deradical* OR de-radical* OR exit* OR de-mobili* OR demobili* OR disarm* ) OR AB ( prevent* OR reduc* OR counter* OR disengage* OR rehab* OR reintegrat* OR re-integrat* OR re-entry OR reentry OR desist* OR recidivism OR deradical* OR de-radical* OR exit* OR de-mobili* OR demobili* OR disarm* ) OR KW ( prevent* OR reduc* OR counter* OR disengage* OR rehab* OR reintegrat* OR re-integrat* OR re-entry OR reentry OR desist* OR recidivism OR deradical* OR de-radical* OR exit* OR de-mobili* OR demobili* OR disarm* ) OR SU ( prevent* OR reduc* OR counter* OR disengage* OR rehab* OR reintegrat* OR re-integrat* OR re-entry OR reentry OR desist* OR recidivism OR deradical* OR de-radical* OR exit* OR de-mobili* OR demobili* OR disarm* ) | Limiters - Publication Date: 20000101-20221231 | Interface - EBSCOhost Research Databases | 111,675 |
|  |  | Search modes - Boolean/Phrase | Search Screen - Advanced Search |  |
|  |  |  | Database - Criminal Justice Abstracts |  |
| S2 | TI ( initiative* OR interven* OR program* OR policy OR policies OR scheme* OR treat* OR approach* OR model* OR strateg* OR method* OR project* OR practice* OR instrument* OR tool* OR framework* OR protocol* OR guid* OR scale* OR system* OR inventor* OR metric* OR template* OR profile* OR criteria OR questionnaire* OR refer* OR assess* OR "case plan*" OR "case formulat*" OR "management plan*" OR "treatment plan*" OR "support plan*" OR "case manage*" OR "risk manage*" OR progress* OR monitor* OR supervis* OR measur* ) OR AB ( initiative* OR interven* OR program* OR policy OR policies OR scheme* OR treat* OR approach* OR model* OR strateg* OR method* OR project* OR practice* OR instrument* OR tool* OR framework* OR protocol* OR guid* OR scale* OR system* OR inventor* OR metric* OR template* OR profile* OR criteria OR questionnaire* OR refer* OR assess* OR "case plan*" OR "case formulat*" OR "management plan*" OR "treatment plan*" OR "support plan*" OR "case manage*" OR "risk manage*" OR progress* OR monitor* OR supervis* OR measur* ) OR KW ( initiative* OR interven* OR program* OR policy OR policies OR scheme* OR treat* OR approach* OR model* OR strateg* OR method* OR project* OR practice* OR instrument* OR tool* OR framework* OR protocol* OR guid* OR scale* OR system* OR inventor* OR metric* OR template* OR profile* OR criteria OR questionnaire* OR refer* OR assess* OR "case plan*" OR "case formulat*" OR "management plan*" OR "treatment plan*" OR "support plan*" OR "case manage*" OR "risk manage*" OR progress* OR monitor* OR supervis* OR measur* ) OR SU ( initiative* OR interven* OR program* OR policy OR policies OR scheme* OR treat* OR approach* OR model* OR strateg* OR method* OR project* OR practice* OR instrument* OR tool* OR framework* OR protocol* OR guid* OR scale* OR system* OR inventor* OR metric* OR template* OR profile* OR criteria OR questionnaire* OR refer* OR assess* OR "case plan*" OR "case formulat*" OR "management plan*" OR "treatment plan*" OR "support plan*" OR "case manage*" OR "risk manage*" OR progress* OR monitor* OR supervis* OR measur* ) | Limiters - Publication Date: 20000101-20221231 | Interface - EBSCOhost Research Databases | 361,549 |
|  |  | Search modes - Boolean/Phrase | Search Screen - Advanced Search |  |
|  |  |  | Database - Criminal Justice Abstracts |  |
| S1 | TI ( violen* OR assault* OR "batter" OR batters OR battere* OR coerc* OR beat* OR stalk* OR rape OR homicid* OR murder* OR kill* OR "intimate terror*" OR "domestic abuse*" OR "sex* abuse*" OR "sex* offen*" OR gangs OR "gang" OR insurgent* OR rebel* OR fighter* OR combatant* OR guerrilla* OR militant* OR soldier* ) OR AB ( violen* OR assault* OR "batter" OR batters OR battere* OR coerc* OR beat* OR stalk* OR rape OR homicid* OR murder* OR kill* OR "intimate terror*" OR "domestic abuse*" OR "sex* abuse*" OR "sex* offen*" OR gangs OR "gang" OR insurgent* OR rebel* OR fighter* OR combatant* OR guerrilla* OR militant* OR soldier* ) OR KW ( violen* OR assault* OR "batter" OR batters OR battere* OR coerc* OR beat* OR stalk* OR rape OR homicid* OR murder* OR kill* OR "intimate terror*" OR "domestic abuse*" OR "sex* abuse*" OR "sex* offen*" OR gangs OR "gang" OR insurgent* OR rebel* OR fighter* OR combatant* OR guerrilla* OR militant* OR soldier* ) OR SU ( violen* OR assault* OR "batter" OR batters OR battere* OR coerc* OR beat* OR stalk* OR rape OR homicid* OR murder* OR kill* OR "intimate terror*" OR "domestic abuse*" OR "sex* abuse*" OR "sex* offen*" OR gangs OR "gang" OR insurgent* OR rebel* OR fighter* OR combatant* OR guerrilla* OR militant* OR soldier* ) | Limiters - Publication Date: 20000101-20221231 | Interface - EBSCOhost Research Databases | 79,320 |
|  |  | Search modes - Boolean/Phrase | Search Screen - Advanced Search |  |
|  |  |  | Database - Criminal Justice Abstracts |  |

**Table A1.3(c). Scopus Search Records – Part II**

| **Query** | **Records** |
| --- | --- |
| ( TITLE-ABS KEY ( violen* OR assault* OR "batter" OR batters OR battere* OR coerc* OR beat* OR stalk* OR rape OR homicid* OR murder* OR kill*OR "intimate terror*" OR "domestic abuse*" OR "sex* abuse*" OR "sex* offen*" OR gangs OR "gang" OR insurgent* OR rebel* OR fighter* ORcombatant* OR guerrilla* OR militant* OR soldier* ) ) AND ( TITLE-ABS-KEY ( initiative* OR interven* OR program* OR policy OR policies ORscheme* OR treat* OR approach* OR model* OR strateg* OR method* OR project* OR practice* OR instrument* OR tool* OR framework* ORprotocol* OR guid* OR scale* OR system* OR inventor* OR metric* OR template* OR profile* OR criteria OR questionnaire* OR refer* OR assess*OR "case plan*" OR "case formulat*" OR "management plan*" OR "treatment plan*" OR "support plan*" OR "case manage*" OR "risk manage*" ORprogress* OR monitor* OR supervis* OR measur* ) ) AND ( TITLE-ABS-KEY ( prevent* OR reduc* OR counter* OR disengage* OR rehab* ORreintegrat* OR re-integrat* OR re-entry OR reentry OR desist* OR recidivism OR deradical* OR de-radical* OR exit* OR de-mobili* OR demobili* ORdisarm* ) ) AND ( TITLE-ABS-KEY ( review* OR meta-analy* ) ) AND ( LIMIT-TO ( LANGUAGE , "english" ) ) AND ( LIMIT-TO ( PUBYEAR , 2022 ) ) | 1474 |
| ( TITLE-ABS-KEY ( violen* OR assault* OR "batter" OR batters OR battere* OR coerc* OR beat* OR stalk* OR rape OR homicid* OR murder* OR kill*OR "intimate terror*" OR "domestic abuse*" OR "sex* abuse*" OR "sex* offen*" OR gangs OR "gang" OR insurgent* OR rebel* OR fighter* ORcombatant* OR guerrilla* OR militant* OR soldier* ) ) AND ( TITLE-ABS-KEY ( initiative* OR interven* OR program* OR policy OR policies OR scheme*OR treat* OR approach* OR model* OR strateg* OR method* OR project* OR practice* OR instrument* OR tool* OR framework* OR protocol* ORguid* OR scale* OR system* OR inventor* OR metric* OR template* OR profile* OR criteria OR questionnaire* OR refer* OR assess* OR "case plan*"OR "case formulat*" OR "management plan*" OR "treatment plan*" OR "support plan*" OR "case manage*" OR "risk manage*" OR progress* ORmonitor* OR supervis* OR measur* ) ) AND ( TITLE-ABS-KEY ( prevent* OR reduc* OR counter* OR disengage* OR rehab* OR reintegrat* OR re-integrat* OR re-entry OR reentry OR desist* OR recidivism OR deradical* OR de-radical* OR exit* OR de-mobili* OR demobili* OR disarm* ) ) AND (TITLE-ABS-KEY ( review* OR meta-analy* ) ) AND ( LIMIT-TO ( LANGUAGE , "english" ) ) AND ( LIMIT-TO ( PUBYEAR , 2021 ) ) AND ( LIMIT-TO (EXACTSRCTITLE , "frontiers in immunology" ) OR LIMIT-TO ( EXACTSRCTITLE , "trauma violence and abuse" ) ) | 185 |
| ( TITLE-ABS-KEY ( violen* OR assault* OR "batter" OR batters OR battere* OR coerc* OR beat* OR stalk* OR rape OR homicid* OR murder* OR kill*OR "intimate terror*" OR "domestic abuse*" OR "sex* abuse*" OR "sex* offen*" OR gangs OR "gang" OR insurgent* OR rebel* OR fighter* ORcombatant* OR guerrilla* OR militant* OR soldier* ) ) AND ( TITLE-ABS-KEY ( initiative* OR interven* OR program* OR policy OR policies ORscheme* OR treat* OR approach* OR model* OR strateg* OR method* OR project* OR practice* OR instrument* OR tool* OR framework* ORprotocol* OR guid* OR scale* OR system* OR inventor* OR metric* OR template* OR profile* OR criteria OR questionnaire* OR refer* OR assess*OR "case plan*" OR "case formulat*" OR "management plan*" OR "treatment plan*" OR "support plan*" OR "case manage*" OR "risk manage*" ORprogress* OR monitor* OR supervis* OR measur* ) ) AND ( TITLE-ABS-KEY ( prevent* OR reduc* OR counter* OR disengage* OR rehab* ORreintegrat* OR re-integrat* OR re-entry OR reentry OR desist* OR recidivism OR deradical* OR de-radical* OR exit* OR de-mobili* OR demobili*OR disarm* ) ) AND ( TITLE-ABS-KEY ( review* OR meta-analy* ) ) AND ( LIMIT-TO ( LANGUAGE , "english" ) ) AND ( LIMIT-TO ( PUBYEAR , 2021 ) )AND ( EXCLUDE ( EXACTSRCTITLE , "frontiers in immunology" ) OR EXCLUDE ( EXACTSRCTITLE , "trauma violence and abuse" ) ) | 1989 |
| ( TITLE-ABS-KEY ( violen* OR assault* OR "batter" OR batters OR battere* OR coerc* OR beat* OR stalk* OR rape OR homicid* OR murder* OR kill*OR "intimate terror*" OR "domestic abuse*" OR "sex* abuse*" OR "sex* offen*" OR gangs OR "gang" OR insurgent* OR rebel* OR fighter* ORcombatant* OR guerrilla* OR militant* OR soldier* ) ) AND ( TITLE-ABS-KEY ( initiative* OR interven* OR program* OR policy OR policies ORscheme* OR treat* OR approach* OR model* OR strateg* OR method* OR project* OR practice* OR instrument* OR tool* OR framework* ORprotocol* OR guid* OR scale* OR system* OR inventor* OR metric* OR template* OR profile* OR criteria OR questionnaire* OR refer* OR assess*OR "case plan*" OR "case formulat*" OR "management plan*" OR "treatment plan*" OR "support plan*" OR "case manage*" OR "risk manage*" ORprogress* OR monitor* OR supervis* OR measur* ) ) AND ( TITLE-ABS-KEY ( prevent* OR reduc* OR counter* OR disengage* OR rehab* ORreintegrat* OR re-integrat* OR re-entry OR reentry OR desist* OR recidivism OR deradical* OR de-radical* OR exit* OR de-mobili* OR demobili*OR disarm* ) ) AND ( TITLE-ABS-KEY ( review* OR meta-analy* ) ) AND ( LIMIT-TO ( LANGUAGE , "english" ) ) AND ( LIMIT-TO ( PUBYEAR , 2021 ) ) | 2174 |
| ( TITLE-ABS-KEY ( violen* OR assault* OR "batter" OR batters OR battere* OR coerc* OR beat* OR stalk* OR rape OR homicid* OR murder* OR kill*OR "intimate terror*" OR "domestic abuse*" OR "sex* abuse*" OR "sex* offen*" OR gangs OR "gang" OR insurgent* OR rebel* OR fighter* ORcombatant* OR guerrilla* OR militant* OR soldier* ) ) AND ( TITLE-ABS-KEY ( initiative* OR interven* OR program* OR policy OR policies ORscheme* OR treat* OR approach* OR model* OR strateg* OR method* OR project* OR practice* OR instrument* OR tool* OR framework* ORprotocol* OR guid* OR scale* OR system* OR inventor* OR metric* OR template* OR profile* OR criteria OR questionnaire* OR refer* OR assess*OR "case plan*" OR "case formulat*" OR "management plan*" OR "treatment plan*" OR "support plan*" OR "case manage*" OR "risk manage*" ORprogress* OR monitor* OR supervis* OR measur* ) ) AND ( TITLE-ABS-KEY ( prevent* OR reduc* OR counter* OR disengage* OR rehab* ORreintegrat* OR re-integrat* OR re-entry OR reentry OR desist* OR recidivism OR deradical* OR de-radical* OR exit* OR de-mobili* OR demobili*OR disarm* ) ) AND ( TITLE-ABS-KEY ( review* OR meta-analy* ) ) AND ( LIMIT-TO ( LANGUAGE , "english" ) ) AND ( LIMIT-TO ( PUBYEAR , 2020 ) ) | 1707 |
| ( TITLE-ABS-KEY ( violen* OR assault* OR "batter" OR batters OR battere* OR coerc* OR beat* OR stalk* OR rape OR homicid* OR murder* OR kill*OR "intimate terror*" OR "domestic abuse*" OR "sex* abuse*" OR "sex* offen*" OR gangs OR "gang" OR insurgent* OR rebel* OR fighter* ORcombatant* OR guerrilla* OR militant* OR soldier* ) ) AND ( TITLE-ABS-KEY ( initiative* OR interven* OR program* OR policy OR policies ORscheme* OR treat* OR approach* OR model* OR strateg* OR method* OR project* OR practice* OR instrument* OR tool* OR framework* ORprotocol* OR guid* OR scale* OR system* OR inventor* OR metric* OR template* OR profile* OR criteria OR questionnaire* OR refer* OR assess*OR "case plan*" OR "case formulat*" OR "management plan*" OR "treatment plan*" OR "support plan*" OR "case manage*" OR "risk manage*" ORprogress* OR monitor* OR supervis* OR measur* ) ) AND ( TITLE-ABS-KEY ( prevent* OR reduc* OR counter* OR disengage* OR rehab* ORreintegrat* OR re-integrat* OR re-entry OR reentry OR desist* OR recidivism OR deradical* OR de-radical* OR exit* OR de-mobili* OR demobili*OR disarm* ) ) AND ( TITLE-ABS-KEY ( review* OR meta-analy* ) ) AND ( LIMIT-TO ( LANGUAGE , "english" ) ) AND ( LIMIT-TO ( PUBYEAR , 2019 ) ) | 1393 |
| ( TITLE-ABS-KEY ( violen* OR assault* OR "batter" OR batters OR battere* OR coerc* OR beat* OR stalk* OR rape OR homicid* OR murder* ORkill* OR "intimate terror*" OR "domestic abuse*" OR "sex* abuse*" OR "sex* offen*" OR gangs OR "gang" OR insurgent* OR rebel* OR fighter* ORcombatant* OR guerrilla* OR militant* OR soldier* ) ) AND ( TITLE-ABS-KEY ( initiative* OR interven* OR program* OR policy OR policies ORscheme* OR treat* OR approach* OR model* OR strateg* OR method* OR project* OR practice* OR instrument* OR tool* OR framework* ORprotocol* OR guid* OR scale* OR system* OR inventor* OR metric* OR template* OR profile* OR criteria OR questionnaire* OR refer* OR assess*OR "case plan*" OR "case formulat*" OR "management plan*" OR "treatment plan*" OR "support plan*" OR "case manage*" OR "risk manage*" ORprogress* OR monitor* OR supervis* OR measur* ) ) AND ( TITLE-ABS-KEY ( prevent* OR reduc* OR counter* OR disengage* OR rehab* ORreintegrat* OR re-integrat* OR re-entry OR reentry OR desist* OR recidivism OR deradical* OR de-radical* OR exit* OR de-mobili* OR demobili*OR disarm* ) ) AND ( TITLE-ABS-KEY ( review* OR meta-analy* ) ) AND ( LIMIT-TO ( LANGUAGE , "english" ) ) AND ( LIMIT-TO ( PUBYEAR , 2018 ) ) | 1332 |
| ( TITLE-ABS-KEY ( violen* OR assault* OR "batter" OR batters OR battere* OR coerc* OR beat* OR stalk* OR rape OR homicid* OR murder* ORkill* OR "intimate terror*" OR "domestic abuse*" OR "sex* abuse*" OR "sex* offen*" OR gangs OR "gang" OR insurgent* OR rebel* OR fighter* ORcombatant* OR guerrilla* OR militant* OR soldier* ) ) AND ( TITLE-ABS-KEY ( initiative* OR interven* OR program* OR policy OR policies ORscheme* OR treat* OR approach* OR model* OR strateg* OR method* OR project* OR practice* OR instrument* OR tool* OR framework* ORprotocol* OR guid* OR scale* OR system* OR inventor* OR metric* OR template* OR profile* OR criteria OR questionnaire* OR refer* OR assess*OR "case plan*" OR "case formulat*" OR "management plan*" OR "treatment plan*" OR "support plan*" OR "case manage*" OR "risk manage*" ORprogress* OR monitor* OR supervis* OR measur* ) ) AND ( TITLE-ABS-KEY ( prevent* OR reduc* OR counter* OR disengage* OR rehab* ORreintegrat* OR re-integrat* OR re-entry OR reentry OR desist* OR recidivism OR deradical* OR de-radical* OR exit* OR de-mobili* OR demobili*OR disarm* ) ) AND ( TITLE-ABS-KEY ( review* OR meta-analy* ) ) AND ( LIMIT-TO ( LANGUAGE , "english" ) ) AND ( LIMIT-TO ( PUBYEAR , 2017 ) ) | 1222 |
| ( TITLE-ABS-KEY ( violen* OR assault* OR "batter" OR batters OR battere* OR coerc* OR beat* OR stalk* OR rape OR homicid* OR murder* OR kill*OR "intimate terror*" OR "domestic abuse*" OR "sex* abuse*" OR "sex* offen*" OR gangs OR "gang" OR insurgent* OR rebel* OR fighter* ORcombatant* OR guerrilla* OR militant* OR soldier* ) ) AND ( TITLE-ABS-KEY ( initiative* OR interven* OR program* OR policy OR policies ORscheme* OR treat* OR approach* OR model* OR strateg* OR method* OR project* OR practice* OR instrument* OR tool* OR framework* ORprotocol* OR guid* OR scale* OR system* OR inventor* OR metric* OR template* OR profile* OR criteria OR questionnaire* OR refer* OR assess*OR "case plan*" OR "case formulat*" OR "management plan*" OR "treatment plan*" OR "support plan*" OR "case manage*" OR "risk manage*" ORprogress* OR monitor* OR supervis* OR measur* ) ) AND ( TITLE-ABS-KEY ( prevent* OR reduc* OR counter* OR disengage* OR rehab* ORreintegrat* OR re-integrat* OR re-entry OR reentry OR desist* OR recidivism OR deradical* OR de-radical* OR exit* OR de-mobili* OR demobili*OR disarm* ) ) AND ( TITLE-ABS-KEY ( review* OR meta-analy* ) ) AND ( LIMIT-TO ( LANGUAGE , "english" ) ) AND ( LIMIT-TO ( PUBYEAR , 2016 ) ) | 1200 |
| ( TITLE-ABS-KEY ( violen* OR assault* OR "batter" OR batters OR battere* OR coerc* OR beat* OR stalk* OR rape OR homicid* OR murder* OR kill*OR "intimate terror*" OR "domestic abuse*" OR "sex* abuse*" OR "sex* offen*" OR gangs OR "gang" OR insurgent* OR rebel* OR fighter* ORcombatant* OR guerrilla* OR militant* OR soldier* ) ) AND ( TITLE-ABS-KEY ( initiative* OR interven* OR program* OR policy OR policies ORscheme* OR treat* OR approach* OR model* OR strateg* OR method* OR project* OR practice* OR instrument* OR tool* OR framework* ORprotocol* OR guid* OR scale* OR system* OR inventor* OR metric* OR template* OR profile* OR criteria OR questionnaire* OR refer* OR assess*OR "case plan*" OR "case formulat*" OR "management plan*" OR "treatment plan*" OR "support plan*" OR "case manage*" OR "risk manage*" ORprogress* OR monitor* OR supervis* OR measur* ) ) AND ( TITLE-ABS-KEY ( prevent* OR reduc* OR counter* OR disengage* OR rehab* ORreintegrat* OR re-integrat* OR re-entry OR reentry OR desist* OR recidivism OR deradical* OR de-radical* OR exit* OR de-mobili* OR demobili*OR disarm* ) ) AND ( TITLE-ABS-KEY ( review* OR meta-analy* ) ) AND ( LIMIT-TO ( LANGUAGE , "english" ) ) AND ( LIMIT-TO ( PUBYEAR , 2015 ) ) | 1126 |
| ( TITLE-ABS-KEY ( violen* OR assault* OR "batter" OR batters OR battere* OR coerc* OR beat* OR stalk* OR rape OR homicid* OR murder* OR kill*OR "intimate terror*" OR "domestic abuse*" OR "sex* abuse*" OR "sex* offen*" OR gangs OR "gang" OR insurgent* OR rebel* OR fighter* ORcombatant* OR guerrilla* OR militant* OR soldier* ) ) AND ( TITLE-ABS-KEY ( initiative* OR interven* OR program* OR policy OR policies ORscheme* OR treat* OR approach* OR model* OR strateg* OR method* OR project* OR practice* OR instrument* OR tool* OR framework* ORprotocol* OR guid* OR scale* OR system* OR inventor* OR metric* OR template* OR profile* OR criteria OR questionnaire* OR refer* OR assess*OR "case plan*" OR "case formulat*" OR "management plan*" OR "treatment plan*" OR "support plan*" OR "case manage*" OR "risk manage*" ORprogress* OR monitor* OR supervis* OR measur* ) ) AND ( TITLE-ABS-KEY ( prevent* OR reduc* OR counter* OR disengage* OR rehab* ORreintegrat* OR re-integrat* OR re-entry OR reentry OR desist* OR recidivism OR deradical* OR de-radical* OR exit* OR de-mobili* OR demobili*OR disarm* ) ) AND ( TITLE-ABS-KEY ( review* OR meta-analy* ) ) AND ( LIMIT-TO ( LANGUAGE , "english" ) ) AND ( LIMIT-TO ( PUBYEAR , 2014 ) ) | 1188 |
| ( TITLE-ABS-KEY ( violen* OR assault* OR "batter" OR batters OR battere* OR coerc* OR beat* OR stalk* OR rape OR homicid* OR murder* OR kill*OR "intimate terror*" OR "domestic abuse*" OR "sex* abuse*" OR "sex* offen*" OR gangs OR "gang" OR insurgent* OR rebel* OR fighter* ORcombatant* OR guerrilla* OR militant* OR soldier* ) ) AND ( TITLE-ABS-KEY ( initiative* OR interven* OR program* OR policy OR policies ORscheme* OR treat* OR approach* OR model* OR strateg* OR method* OR project* OR practice* OR instrument* OR tool* OR framework* ORprotocol* OR guid* OR scale* OR system* OR inventor* OR metric* OR template* OR profile* OR criteria OR questionnaire* OR refer* OR assess*OR "case plan*" OR "case formulat*" OR "management plan*" OR "treatment plan*" OR "support plan*" OR "case manage*" OR "risk manage*" ORprogress* OR monitor* OR supervis* OR measur* ) ) AND ( TITLE-ABS-KEY ( prevent* OR reduc* OR counter* OR disengage* OR rehab* ORreintegrat* OR re-integrat* OR re-entry OR reentry OR desist* OR recidivism OR deradical* OR de-radical* OR exit* OR de-mobili* OR demobili*OR disarm* ) ) AND ( TITLE-ABS-KEY ( review* OR meta-analy* ) ) AND ( LIMIT-TO ( LANGUAGE , "english" ) ) AND ( LIMIT-TO ( PUBYEAR , 2013 ) ) | 1149 |
| ( TITLE-ABS-KEY ( violen* OR assault* OR "batter" OR batters OR battere* OR coerc* OR beat* OR stalk* OR rape OR homicid* OR murder* OR kill*OR "intimate terror*" OR "domestic abuse*" OR "sex* abuse*" OR "sex* offen*" OR gangs OR "gang" OR insurgent* OR rebel* OR fighter* ORcombatant* OR guerrilla* OR militant* OR soldier* ) ) AND ( TITLE-ABS-KEY ( initiative* OR interven* OR program* OR policy OR policies ORscheme* OR treat* OR approach* OR model* OR strateg* OR method* OR project* OR practice* OR instrument* OR tool* OR framework* ORprotocol* OR guid* OR scale* OR system* OR inventor* OR metric* OR template* OR profile* OR criteria OR questionnaire* OR refer* OR assess*OR "case plan*" OR "case formulat*" OR "management plan*" OR "treatment plan*" OR "support plan*" OR "case manage*" OR "risk manage*" ORprogress* OR monitor* OR supervis* OR measur* ) ) AND ( TITLE-ABS-KEY ( prevent* OR reduc* OR counter* OR disengage* OR rehab* ORreintegrat* OR re-integrat* OR re-entry OR reentry OR desist* OR recidivism OR deradical* OR de-radical* OR exit* OR de-mobili* OR demobili*OR disarm* ) ) AND ( TITLE-ABS-KEY ( review* OR meta-analy* ) ) AND ( LIMIT-TO ( LANGUAGE , "english" ) ) AND ( LIMIT-TO ( PUBYEAR , 2012 ) ) | 1076 |
| ( TITLE-ABS-KEY ( violen* OR assault* OR "batter" OR batters OR battere* OR coerc* OR beat* OR stalk* OR rape OR homicid* OR murder* OR kill*OR "intimate terror*" OR "domestic abuse*" OR "sex* abuse*" OR "sex* offen*" OR gangs OR "gang" OR insurgent* OR rebel* OR fighter* ORcombatant* OR guerrilla* OR militant* OR soldier* ) ) AND ( TITLE-ABS-KEY ( initiative* OR interven* OR program* OR policy OR policies ORscheme* OR treat* OR approach* OR model* OR strateg* OR method* OR project* OR practice* OR instrument* OR tool* OR framework* ORprotocol* OR guid* OR scale* OR system* OR inventor* OR metric* OR template* OR profile* OR criteria OR questionnaire* OR refer* OR assess*OR "case plan*" OR "case formulat*" OR "management plan*" OR "treatment plan*" OR "support plan*" OR "case manage*" OR "risk manage*" ORprogress* OR monitor* OR supervis* OR measur* ) ) AND ( TITLE-ABS-KEY ( prevent* OR reduc* OR counter* OR disengage* OR rehab* ORreintegrat* OR re-integrat* OR re-entry OR reentry OR desist* OR recidivism OR deradical* OR de-radical* OR exit* OR de-mobili* OR demobili* ORdisarm* ) ) AND ( TITLE-ABS-KEY ( review* OR meta-analy* ) ) AND ( LIMIT-TO ( LANGUAGE , "english" ) ) AND ( LIMIT-TO ( PUBYEAR , 2011 ) ) | 991 |
| ( TITLE-ABS-KEY ( violen* OR assault* OR "batter" OR batters OR battere* OR coerc* OR beat* OR stalk* OR rape OR homicid* OR murder* OR kill*OR "intimate terror*" OR "domestic abuse*" OR "sex* abuse*" OR "sex* offen*" OR gangs OR "gang" OR insurgent* OR rebel* OR fighter* ORcombatant* OR guerrilla* OR militant* OR soldier* ) ) AND ( TITLE-ABS-KEY ( initiative* OR interven* OR program* OR policy OR policies ORscheme* OR treat* OR approach* OR model* OR strateg* OR method* OR project* OR practice* OR instrument* OR tool* OR framework* ORprotocol* OR guid* OR scale* OR system* OR inventor* OR metric* OR template* OR profile* OR criteria OR questionnaire* OR refer* OR assess*OR "case plan*" OR "case formulat*" OR "management plan*" OR "treatment plan*" OR "support plan*" OR "case manage*" OR "risk manage*" ORprogress* OR monitor* OR supervis* OR measur* ) ) AND ( TITLE-ABS-KEY ( prevent* OR reduc* OR counter* OR disengage* OR rehab* ORreintegrat* OR re-integrat* OR re-entry OR reentry OR desist* OR recidivism OR deradical* OR de-radical* OR exit* OR de-mobili* OR demobili*OR disarm* ) ) AND ( TITLE-ABS-KEY ( review* OR meta-analy* ) ) AND ( LIMIT-TO ( LANGUAGE , "english" ) ) AND ( LIMIT-TO ( PUBYEAR , 2010 ) ORLIMIT-TO ( PUBYEAR , 2009 ) ) | 1784 |
| ( TITLE-ABS-KEY ( violen* OR assault* OR "batter" OR batters OR battere* OR coerc* OR beat* OR stalk* OR rape OR homicid* OR murder* OR kill*OR "intimate terror*" OR "domestic abuse*" OR "sex* abuse*" OR "sex* offen*" OR gangs OR "gang" OR insurgent* OR rebel* OR fighter* ORcombatant* OR guerrilla* OR militant* OR soldier* ) ) AND ( TITLE-ABS-KEY ( initiative* OR interven* OR program* OR policy OR policies ORscheme* OR treat* OR approach* OR model* OR strateg* OR method* OR project* OR practice* OR instrument* OR tool* OR framework* ORprotocol* OR guid* OR scale* OR system* OR inventor* OR metric* OR template* OR profile* OR criteria OR questionnaire* OR refer* OR assess*OR "case plan*" OR "case formulat*" OR "management plan*" OR "treatment plan*" OR "support plan*" OR "case manage*" OR "risk manage*" ORprogress* OR monitor* OR supervis* OR measur* ) ) AND ( TITLE-ABS-KEY ( prevent* OR reduc* OR counter* OR disengage* OR rehab* ORreintegrat* OR re-integrat* OR re-entry OR reentry OR desist* OR recidivism OR deradical* OR de-radical* OR exit* OR de-mobili* OR demobili*OR disarm* ) ) AND ( TITLE-ABS-KEY ( review* OR meta-analy* ) ) AND ( LIMIT-TO ( LANGUAGE , "english" ) ) AND ( LIMIT-TO ( PUBYEAR , 2008 ) ORLIMIT-TO ( PUBYEAR , 2007 ) ) | 1544 |
| ( TITLE-ABS-KEY ( violen* OR assault* OR "batter" OR batters OR battere* OR coerc* OR beat* OR stalk* OR rape OR homicid* OR murder* OR kill*OR "intimate terror*" OR "domestic abuse*" OR "sex* abuse*" OR "sex* offen*" OR gangs OR "gang" OR insurgent* OR rebel* OR fighter* ORcombatant* OR guerrilla* OR militant* OR soldier* ) ) AND ( TITLE-ABS-KEY ( initiative* OR interven* OR program* OR policy OR policies ORscheme* OR treat* OR approach* OR model* OR strateg* OR method* OR project* OR practice* OR instrument* OR tool* OR framework* ORprotocol* OR guid* OR scale* OR system* OR inventor* OR metric* OR template* OR profile* OR criteria OR questionnaire* OR refer* OR assess*OR "case plan*" OR "case formulat*" OR "management plan*" OR "treatment plan*" OR "support plan*" OR "case manage*" OR "risk manage*" ORprogress* OR monitor* OR supervis* OR measur* ) ) AND ( TITLE-ABS-KEY ( prevent* OR reduc* OR counter* OR disengage* OR rehab* ORreintegrat* OR re-integrat* OR re-entry OR reentry OR desist* OR recidivism OR deradical* OR de-radical* OR exit* OR de-mobili* OR demobili*OR disarm* ) ) AND ( TITLE-ABS-KEY ( review* OR meta-analy* ) ) AND ( LIMIT-TO ( LANGUAGE , "english" ) ) AND ( LIMIT-TO ( PUBYEAR , 2006 )OR LIMIT-TO ( PUBYEAR , 2005 ) ) | 1489 |
| ( TITLE-ABS-KEY ( violen* OR assault* OR "batter" OR batters OR battere* OR coerc* OR beat* OR stalk* OR rape OR homicid* OR murder* OR kill*OR "intimate terror*" OR "domestic abuse*" OR "sex* abuse*" OR "sex* offen*" OR gangs OR "gang" OR insurgent* OR rebel* OR fighter* ORcombatant* OR guerrilla* OR militant* OR soldier* ) ) AND ( TITLE-ABS-KEY ( initiative* OR interven* OR program* OR policy OR policies ORscheme* OR treat* OR approach* OR model* OR strateg* OR method* OR project* OR practice* OR instrument* OR tool* OR framework* ORprotocol* OR guid* OR scale* OR system* OR inventor* OR metric* OR template* OR profile* OR criteria OR questionnaire* OR refer* OR assess*OR "case plan*" OR "case formulat*" OR "management plan*" OR "treatment plan*" OR "support plan*" OR "case manage*" OR "risk manage*" ORprogress* OR monitor* OR supervis* OR measur* ) ) AND ( TITLE-ABS-KEY ( prevent* OR reduc* OR counter* OR disengage* OR rehab* ORreintegrat* OR re-integrat* OR re-entry OR reentry OR desist* OR recidivism OR deradical* OR de-radical* OR exit* OR de-mobili* OR demobili*OR disarm* ) ) AND ( TITLE-ABS-KEY ( review* OR meta-analy* ) ) AND ( LIMIT-TO ( LANGUAGE , "english" ) ) AND ( LIMIT-TO ( PUBYEAR , 2004 ) ORLIMIT-TO ( PUBYEAR , 2003 ) ) | 1315 |
| ( TITLE-ABS-KEY ( violen* OR assault* OR "batter" OR batters OR battere* OR coerc* OR beat* OR stalk* OR rape OR homicid* OR murder* OR kill*OR "intimate terror*" OR "domestic abuse*" OR "sex* abuse*" OR "sex* offen*" OR gangs OR "gang" OR insurgent* OR rebel* OR fighter* ORcombatant* OR guerrilla* OR militant* OR soldier* ) ) AND ( TITLE-ABS-KEY ( initiative* OR interven* OR program* OR policy OR policies ORscheme* OR treat* OR approach* OR model* OR strateg* OR method* OR project* OR practice* OR instrument* OR tool* OR framework* ORprotocol* OR guid* OR scale* OR system* OR inventor* OR metric* OR template* OR profile* OR criteria OR questionnaire* OR refer* OR assess*OR "case plan*" OR "case formulat*" OR "management plan*" OR "treatment plan*" OR "support plan*" OR "case manage*" OR "risk manage*" ORprogress* OR monitor* OR supervis* OR measur* ) ) AND ( TITLE-ABS-KEY ( prevent* OR reduc* OR counter* OR disengage* OR rehab* ORreintegrat* OR re-integrat* OR re-entry OR reentry OR desist* OR recidivism OR deradical* OR de-radical* OR exit* OR de-mobili* OR demobili*OR disarm* ) ) AND ( TITLE-ABS-KEY ( review* OR meta-analy* ) ) AND ( LIMIT-TO ( LANGUAGE , "english" ) ) AND ( LIMIT-TO ( PUBYEAR , 2002 )OR LIMIT-TO ( PUBYEAR , 2001 ) OR LIMIT-TO ( PUBYEAR , 2000 ) ) | 1394 |
| ( TITLE-ABS-KEY ( violen* OR assault* OR "batter" OR batters OR battere* OR coerc* OR beat* OR stalk* OR rape OR homicid* OR murder* ORkill* OR "intimate terror*" OR "domestic abuse*" OR "sex* abuse*" OR "sex* offen*" OR gangs OR "gang" OR insurgent* OR rebel* OR fighter* ORcombatant* OR guerrilla* OR militant* OR soldier* ) ) AND ( TITLE-ABS-KEY ( initiative* OR interven* OR program* OR policy OR policies ORscheme* OR treat* OR approach* OR model* OR strateg* OR method* OR project* OR practice* OR instrument* OR tool* OR framework* ORprotocol* OR guid* OR scale* OR system* OR inventor* OR metric* OR template* OR profile* OR criteria OR questionnaire* OR refer* ORassess* OR "case plan*" OR "case formulat*" OR "management plan*" OR "treatment plan*" OR "support plan*" OR "case manage*" OR "risk manage*" OR progress* OR monitor* OR supervis* OR measur* ) ) AND ( TITLE-ABS-KEY ( prevent* OR reduc* OR counter* OR disengage* ORrehab* OR reintegrat* OR re-integrat* OR re-entry OR reentry OR desist* OR recidivism OR deradical* OR de-radical* OR exit* OR de-mobili* ORdemobili* OR disarm* ) ) AND ( TITLE-ABS-KEY ( review* OR meta-analy* ) ) AND ( LIMIT-TO ( LANGUAGE , "english" ) ) AND ( EXCLUDE (DOCTYPE , "no" ) OR EXCLUDE ( DOCTYPE , "ed" ) OR EXCLUDE ( DOCTYPE , "le" ) ) AND ( LIMIT-TO ( PUBYEAR , 2022 ) OR LIMIT-TO ( PUBYEAR, 2021 ) OR LIMIT-TO ( PUBYEAR , 2020 ) OR LIMIT-TO ( PUBYEAR , 2019 ) OR LIMIT-TO ( PUBYEAR , 2018 ) OR LIMIT-TO ( PUBYEAR , 2017 ) ORLIMIT-TO ( PUBYEAR , 2016 ) OR LIMIT-TO ( PUBYEAR , 2015 ) OR LIMIT-TO ( PUBYEAR , 2014 ) OR LIMIT-TO ( PUBYEAR , 2013 ) OR LIMIT-TO (PUBYEAR , 2012 ) OR LIMIT-TO ( PUBYEAR , 2011 ) OR LIMIT-TO ( PUBYEAR , 2010 ) OR LIMIT-TO ( PUBYEAR , 2009 ) OR LIMIT-TO ( PUBYEAR ,2008 ) OR LIMIT-TO ( PUBYEAR , 2007 ) OR LIMIT-TO ( PUBYEAR , 2006 ) OR LIMIT-TO ( PUBYEAR , 2005 ) OR LIMIT-TO ( PUBYEAR , 2004 ) ORLIMIT-TO ( PUBYEAR , 2003 ) OR LIMIT-TO ( PUBYEAR , 2002 ) OR LIMIT-TO ( PUBYEAR , 2001 ) OR LIMIT-TO ( PUBYEAR , 2000 ) ) | 23350 |
| ( TITLE-ABS-KEY ( violen* OR assault* OR "batter" OR batters OR battere* OR coerc* OR beat* OR stalk* OR rape OR homicid* OR murder* ORkill* OR "intimate terror*" OR "domestic abuse*" OR "sex* abuse*" OR "sex* offen*" OR gangs OR "gang" OR insurgent* OR rebel* OR fighter* ORcombatant* OR guerrilla* OR militant* OR soldier* ) ) AND ( TITLE-ABS-KEY ( initiative* OR interven* OR program* OR policy OR policies ORscheme* OR treat* OR approach* OR model* OR strateg* OR method* OR project* OR practice* OR instrument* OR tool* OR framework* ORprotocol* OR guid* OR scale* OR system* OR inventor* OR metric* OR template* OR profile* OR criteria OR questionnaire* OR refer* ORassess* OR "case plan*" OR "case formulat*" OR "management plan*" OR "treatment plan*" OR "support plan*" OR "case manage*" OR "risk manage*" OR progress* OR monitor* OR supervis* OR measur* ) ) AND ( TITLE-ABS-KEY ( prevent* OR reduc* OR counter* OR disengage* ORrehab* OR reintegrat* OR re-integrat* OR re-entry OR reentry OR desist* OR recidivism OR deradical* OR de-radical* OR exit* OR de-mobili* ORdemobili* OR disarm* ) ) AND ( TITLE-ABS-KEY ( review* OR meta-analy* ) ) AND ( LIMIT-TO ( LANGUAGE , "english" ) ) AND ( EXCLUDE (DOCTYPE , "no" ) OR EXCLUDE ( DOCTYPE , "ed" ) OR EXCLUDE ( DOCTYPE , "le" ) ) AND ( LIMIT-TO ( PUBYEAR , 2022 ) OR LIMIT-TO ( PUBYEAR, 2021 ) OR LIMIT-TO ( PUBYEAR , 2020 ) OR LIMIT-TO ( PUBYEAR , 2019 ) OR LIMIT-TO ( PUBYEAR , 2018 ) OR LIMIT-TO ( PUBYEAR , 2017 ) ORLIMIT-TO ( PUBYEAR , 2016 ) OR LIMIT-TO ( PUBYEAR , 2015 ) OR LIMIT-TO ( PUBYEAR , 2014 ) OR LIMIT-TO ( PUBYEAR , 2013 ) OR LIMIT-TO (PUBYEAR , 2012 ) OR LIMIT-TO ( PUBYEAR , 2011 ) OR LIMIT-TO ( PUBYEAR , 2010 ) OR LIMIT-TO ( PUBYEAR , 2009 ) OR LIMIT-TO ( PUBYEAR ,2008 ) OR LIMIT-TO ( PUBYEAR , 2007 ) OR LIMIT-TO ( PUBYEAR , 2006 ) OR LIMIT-TO ( PUBYEAR , 2005 ) OR LIMIT-TO ( PUBYEAR , 2004 ) ORLIMIT-TO ( PUBYEAR , 2003 ) OR LIMIT-TO ( PUBYEAR , 2002 ) OR LIMIT-TO ( PUBYEAR , 2001 ) OR LIMIT-TO ( PUBYEAR , 2000 ) ) | 23350 |
| ( TITLE-ABS-KEY ( violen* OR assault* OR "batter" OR batters OR battere* OR coerc* OR beat* OR stalk* OR rape OR homicid* OR murder* ORkill* OR "intimate terror*" OR "domestic abuse*" OR "sex* abuse*" OR "sex* offen*" OR gangs OR "gang" OR insurgent* OR rebel* OR fighter* ORcombatant* OR guerrilla* OR militant* OR soldier* ) ) AND ( TITLE-ABS-KEY ( initiative* OR interven* OR program* OR policy OR policies ORscheme* OR treat* OR approach* OR model* OR strateg* OR method* OR project* OR practice* OR instrument* OR tool* OR framework* ORprotocol* OR guid* OR scale* OR system* OR inventor* OR metric* OR template* OR profile* OR criteria OR questionnaire* OR refer* OR assess*OR "case plan*" OR "case formulat*" OR "management plan*" OR "treatment plan*" OR "support plan*" OR "case manage*" OR "risk manage*" ORprogress* OR monitor* OR supervis* OR measur* ) ) AND ( TITLE-ABS-KEY ( prevent* OR reduc* OR counter* OR disengage* OR rehab* ORreintegrat* OR re-integrat* OR re-entry OR reentry OR desist* OR recidivism OR deradical* OR de-radical* OR exit* OR de-mobili* OR demobili*OR disarm* ) ) AND ( TITLE-ABS-KEY ( review* OR meta-analy* ) ) AND ( LIMIT-TO ( LANGUAGE , "english" ) ) AND ( EXCLUDE ( DOCTYPE , "no" )OR EXCLUDE ( DOCTYPE , "ed" ) OR EXCLUDE ( DOCTYPE , "le" ) ) | 26493 |
| ( TITLE-ABS-KEY ( violen* OR assault* OR "batter" OR batters OR battere* OR coerc* OR beat* OR stalk* OR rape OR homicid* OR murder* ORkill* OR "intimate terror*" OR "domestic abuse*" OR "sex* abuse*" OR "sex* offen*" OR gangs OR "gang" OR insurgent* OR rebel* OR fighter* ORcombatant* OR guerrilla* OR militant* OR soldier* ) ) AND ( TITLE-ABS-KEY ( initiative* OR interven* OR program* OR policy OR policies ORscheme* OR treat* OR approach* OR model* OR strateg* OR method* OR project* OR practice* OR instrument* OR tool* OR framework* ORprotocol* OR guid* OR scale* OR system* OR inventor* OR metric* OR template* OR profile* OR criteria OR questionnaire* OR refer* OR assess*OR "case plan*" OR "case formulat*" OR "management plan*" OR "treatment plan*" OR "support plan*" OR "case manage*" OR "risk manage*" ORprogress* OR monitor* OR supervis* OR measur* ) ) AND ( TITLE-ABS-KEY ( prevent* OR reduc* OR counter* OR disengage* OR rehab* ORreintegrat* OR re-integrat* OR re-entry OR reentry OR desist* OR recidivism OR deradical* OR de-radical* OR exit* OR de-mobili* OR demobili*OR disarm* ) ) AND ( TITLE-ABS-KEY ( review* OR meta-analy* ) ) AND ( LIMIT-TO ( LANGUAGE , "english" ) ) | 26726 |
| ( TITLE-ABS-KEY ( violen* OR assault* OR "batter" OR batters OR battere* OR coerc* OR beat* OR stalk* OR rape OR homicid* OR murder* ORkill* OR "intimate terror*" OR "domestic abuse*" OR "sex* abuse*" OR "sex* offen*" OR gangs OR "gang" OR insurgent* OR rebel* OR fighter* ORcombatant* OR guerrilla* OR militant* OR soldier* ) ) AND ( TITLE-ABS-KEY ( initiative* OR interven* OR program* OR policy OR policies ORscheme* OR treat* OR approach* OR model* OR strateg* OR method* OR project* OR practice* OR instrument* OR tool* OR framework* ORprotocol* OR guid* OR scale* OR system* OR inventor* OR metric* OR template* OR profile* OR criteria OR questionnaire* OR refer* OR assess*OR "case plan*" OR "case formulat*" OR "management plan*" OR "treatment plan*" OR "support plan*" OR "case manage*" OR "risk manage*" ORprogress* OR monitor* OR supervis* OR measur* ) ) AND ( TITLE-ABS-KEY ( prevent* OR reduc* OR counter* OR disengage* OR rehab* ORreintegrat* OR re-integrat* OR re-entry OR reentry OR desist* OR recidivism OR deradical* OR de-radical* OR exit* OR de-mobili* OR demobili*OR disarm* ) ) AND ( TITLE-ABS-KEY ( review* OR meta-analy* ) ) | 28696 |
| TITLE-ABS-KEY ( review* OR meta-analy* ) | 6415221 |
| TITLE-ABS-KEY ( prevent* OR reduc* OR counter* OR disengage* OR rehab* OR reintegrat* OR re-integrat* OR re-entry OR reentry ORdesist* OR recidivism OR deradical* OR de-radical* OR exit* OR de-mobili* OR demobili* OR disarm* ) | 13419817 |
| TITLE-ABS-KEY ( initiative* OR interven* OR program* OR policy OR policies OR scheme* OR treat* OR approach* OR model* OR strateg*OR method* OR project* OR practice* OR instrument* OR tool* OR framework* OR protocol* OR guid* OR scale* OR system* OR inventor*OR metric* OR template* OR profile* OR criteria OR questionnaire* OR refer* OR assess* OR "case plan*" OR "case formulat*" OR"management plan*" OR "treatment plan*" OR "support plan*" OR "case manage*" OR "risk manage*" OR progress* OR monitor* ORsupervis* OR measur* ) | 59607244 |
| TITLE-ABS-KEY ( violen* OR assault* OR "batter" OR batters OR battere* OR coerc* OR beat* OR stalk* OR rape OR homicid* OR murder* ORkill* OR "intimate terror*" OR "domestic abuse*" OR "sex* abuse*" OR "sex* offen*" OR gangs OR "gang" OR insurgent* OR rebel* OR fighter*OR combatant* OR guerrilla* OR militant* OR soldier* ) | 1142223 |

**Table A1.4(c). CINCH: Australian Criminology Database Search Records – Part II**

| [All Fields: violen* OR All Fields: assault* OR All Fields: 'batter' OR All Fields: batters OR All Fields: battere* OR All Fields: coerc* OR All Fields: beat* OR All Fields: stalk* OR All Fields: rape OR All Fields: homicid* OR All Fields: murder* OR All Fields: kill* OR All Fields: 'intimate terror*' OR All Fields: 'domestic abuse*' OR All Fields: 'sex* abuse*' OR All Fields: 'sex* offen*' OR All Fields: gangs OR All Fields: 'gang' OR All Fields: insurgent* OR All Fields: rebel* OR All Fields: fighter* OR All Fields: combatant* OR All Fields: guerrilla* OR All Fields: militant* OR All Fields: soldier*] AND [All Fields: initiative* OR All Fields: interven* OR All Fields: program* OR All Fields: policy OR All Fields: policies OR All Fields: scheme* OR All Fields: treat* OR All Fields: approach* OR All Fields: model* OR All Fields: strateg* OR All Fields: method* OR All Fields: project* OR All Fields: practice* OR All Fields: instrument* OR All Fields: tool* OR All Fields: framework* OR All Fields: protocol* OR All Fields: guid* OR All Fields: scale* OR All Fields: system* OR All Fields: inventor* OR All Fields: metric* OR All Fields: template* OR All Fields: profile* OR All Fields: criteria OR All Fields: questionnaire* OR All Fields: refer* OR All Fields: assess* OR All Fields: 'case plan*' OR All Fields: 'case formulat*' OR All Fields: 'management plan*' OR All Fields: 'treatment plan*' OR All Fields: 'support plan*' OR All Fields: 'case manage*' OR All Fields: 'risk manage*' OR All Fields: progress* OR All Fields: monitor* OR All Fields: supervis* OR All Fields: measur*] AND [All Fields: prevent* OR All Fields: reduc* OR All Fields: counter* OR All Fields: disengage* OR All Fields: rehab* OR All Fields: reintegrat* OR All Fields: re-integrat* OR All Fields: re-entry OR All Fields: reentry OR All Fields: desist* OR All Fields: recidivism OR All Fields: deradical* OR All Fields: de-radical* OR All Fields: exit* OR All Fields: de-mobili* OR All Fields: demobili* OR All Fields: disarm*] AND [All Fields: review* OR All Fields: meta-analy*] AND Publication Date: (01/01/2000 TO 31/12/2022) | 591 |
| --- | --- |

**Table A1.5(c). Medline Search Records – Part II**

| 1 | (violen* or assault* or "batter" or batters or battere* or coerc* or beat* or stalk* or rape or homicid* or murder* or kill* or "intimate terror*" or "domestic abuse*" or "sex* abuse*" or "sex* offen*" or gangs or "gang" or insurgent* or rebel* or fighter* or combatant* or guerrilla* or militant* or soldier*).ab,hw,kf,ot,sh,sy,ti,fx. | 484303 |
| --- | --- | --- |
| 2 | (initiative* or interven* or program* or policy or scheme* or treat* or approach* or model* or strateg* or method* or project* or practice* or instrument* or tool* or framework* or protocol* or guid* or scale* or system* or inventor* or metric* or template* or profile* or criteria or questionnaire* or refer* or assess* or "case plan*" or "case formulat*" or "management plan*" or "treatment plan*" or "support plan*" or "case manage*" or "risk manage*" or progress* or monitor* or supervis* or measur*).ab,hw,kf,ot,sh,sy,ti,fx. | 22624605 |
| 3 | (prevent* or reduc* or counter* or disengage* or rehab* or reintegrat* or re-integrat* or re-entry or reentry or desist* or recidivism or deradical* or de-radical* or exit* or de-mobili* or demobili* or disarm*).ab,hw,kf,ot,sh,sy,ti,fx. | 6700350 |
| 4 | (review* or meta-analy*).ab,hw,kf,ot,sh,sy,ti,fx. | 4324246 |
| 5 | 1 and 2 and 3 and 4 | 18036 |
| 6 | limit 5 to (english language and humans and yr="2000 - 2022") | 11538 |

**Table A1.6(c). PsycInfo Search Records – Part II**

| 1 | (violen* or assault* or "batter" or batters or battere* or coerc* or beat* or stalk* or rape or homicid* or murder* or kill* or "intimate terror*" or "domestic abuse*" or "sex* abuse*" or "sex* offen*" or gangs or "gang" or insurgent* or rebel* or fighter* or combatant* or guerrilla* or militant* or soldier*).ab,hw,id,mh,ot,sh,ti. | 203778 |
| --- | --- | --- |
| 2 | (initiative* or interven* or program* or policy or scheme* or treat* or approach* or model* or strateg* or method* or project* or practice* or instrument* or tool* or framework* or protocol* or guid* or scale* or system* or inventor* or metric* or template* or profile* or criteria or questionnaire* or refer* or assess* or "case plan*" or "case formulat*" or "management plan*" or "treatment plan*" or "support plan*" or "case manage*" or "risk manage*" or progress* or monitor* or supervis* or measur*).ab,hw,id,mh,ot,sh,ti. | 4160657 |
| 3 | (prevent* or reduc* or counter* or disengage* or rehab* or reintegrat* or re-integrat* or re-entry or reentry or desist* or recidivism or deradical* or de-radical* or exit* or de-mobili* or demobili* or disarm*).ab,hw,id,mh,ot,sh,ti. | 861007 |
| 4 | (review* or meta-analy*).ab,hw,id,mh,ot,sh,ti. | 609538 |
| 5 | 1 and 2 and 3 and 4 | 7198 |
| 6 | limit 5 to (human and english language and yr="2000 - 2022") | 5644 |

**Table A1.7(c). Dissertations and Theses Global – Part II**

| Set# | Searched for | Databases | Results |
| --- | --- | --- | --- |
| S1 | ti(violen* OR assault* OR "batter" OR batters OR battere* OR coerc* OR beat* OR stalk* OR rape OR homicid* OR murder* OR kill* OR "intimate terror*" OR "domestic abuse*" OR "sex* abuse*" OR "sex* offen*" OR gangs OR "gang" OR insurgent* OR rebel* OR fighter* OR combatant* OR guerrilla* OR militant* OR soldier*) OR ab(violen* OR assault* OR "batter" OR batters OR battere* OR coerc* OR beat* OR stalk* OR rape OR homicid* OR murder* OR kill* OR "intimate terror*" OR "domestic abuse*" OR "sex* abuse*" OR "sex* offen*" OR gangs OR "gang" OR insurgent* OR rebel* OR fighter* OR combatant* OR guerrilla* OR militant* OR soldier*) OR mainsubject(violen* OR assault* OR "batter" OR batters OR battere* OR coerc* OR beat* OR stalk* OR rape OR homicid* OR murder* OR kill* OR "intimate terror*" OR "domestic abuse*" OR "sex* abuse*" OR "sex* offen*" OR gangs OR "gang" OR insurgent* OR rebel* OR fighter* OR combatant* OR guerrilla* OR militant* OR soldier*) OR diskw(violen* OR assault* OR "batter" OR batters OR battere* OR coerc* OR beat* OR stalk* OR rape OR homicid* OR murder* OR kill* OR "intimate terror*" OR "domestic abuse*" OR "sex* abuse*" OR "sex* offen*" OR gangs OR "gang" OR insurgent* OR rebel* OR fighter* OR combatant* OR guerrilla* OR militant* OR soldier*) AND pd(20000101-20221231) | ProQuest Dissertations & Theses Global | 96605 |
| S2 | ti(initiative* OR interven* OR program* OR policy OR policies OR scheme* OR treat* OR approach* OR model* OR strateg* OR method* OR project* OR practice* OR instrument* OR tool* OR framework* OR protocol* OR guid* OR scale* OR system* OR inventor* OR metric* OR template* OR profile* OR criteria OR questionnaire* OR refer* OR assess* OR "case plan*" OR "case formulat*" OR "management plan*" OR "treatment plan*" OR "support plan*" OR "case manage*" OR "risk manage*" OR progress* OR monitor* OR supervis* OR measur*) OR ab(initiative* OR interven* OR program* OR policy OR policies OR scheme* OR treat* OR approach* OR model* OR strateg* OR method* OR project* OR practice* OR instrument* OR tool* OR framework* OR protocol* OR guid* OR scale* OR system* OR inventor* OR metric* OR template* OR profile* OR criteria OR questionnaire* OR refer* OR assess* OR "case plan*" OR "case formulat*" OR "management plan*" OR "treatment plan*" OR "support plan*" OR "case manage*" OR "risk manage*" OR progress* OR monitor* OR supervis* OR measur*) OR mainsubject(initiative* OR interven* OR program* OR policy OR policies OR scheme* OR treat* OR approach* OR model* OR strateg* OR method* OR project* OR practice* OR instrument* OR tool* OR framework* OR protocol* OR guid* OR scale* OR system* OR inventor* OR metric* OR template* OR profile* OR criteria OR questionnaire* OR refer* OR assess* OR "case plan*" OR "case formulat*" OR "management plan*" OR "treatment plan*" OR "support plan*" OR "case manage*" OR "risk manage*" OR progress* OR monitor* OR supervis* OR measur*) OR diskw(initiative* OR interven* OR program* OR policy OR policies OR scheme* OR treat* OR approach* OR model* OR strateg* OR method* OR project* OR practice* OR instrument* OR tool* OR framework* OR protocol* OR guid* OR scale* OR system* OR inventor* OR metric* OR template* OR profile* OR criteria OR questionnaire* OR refer* OR assess* OR "case plan*" OR "case formulat*" OR "management plan*" OR "treatment plan*" OR "support plan*" OR "case manage*" OR "risk manage*" OR progress* OR monitor* OR supervis* OR measur*) AND pd(20000101-20221231) | ProQuest Dissertations & Theses Global | 2548316 |
| S3 | ti(prevent* OR reduc* OR counter* OR disengage* OR rehab* OR reintegrat* OR re-integrat* OR re-entry OR reentry OR desist* OR recidivism OR deradical* OR de-radical* OR exit* OR de-mobili* OR demobili* OR disarm*) OR ab(prevent* OR reduc* OR counter* OR disengage* OR rehab* OR reintegrat* OR re-integrat* OR re-entry OR reentry OR desist* OR recidivism OR deradical* OR de-radical* OR exit* OR de-mobili* OR demobili* OR disarm*) OR mainsubject(prevent* OR reduc* OR counter* OR disengage* OR rehab* OR reintegrat* OR re-integrat* OR re-entry OR reentry OR desist* OR recidivism OR deradical* OR de-radical* OR exit* OR de-mobili* OR demobili* OR disarm*) OR diskw(prevent* OR reduc* OR counter* OR disengage* OR rehab* OR reintegrat* OR re-integrat* OR re-entry OR reentry OR desist* OR recidivism OR deradical* OR de-radical* OR exit* OR de-mobili* OR demobili* OR disarm*) AND pd(20000101-20221231) | ProQuest Dissertations & Theses Global | 632764 |
| S4 | ti(review* OR meta-analy*) OR ab(review* OR meta-analy*) OR mainsubject(review* OR meta-analy*) OR diskw(review* OR meta-analy*) AND pd(20000101-20221231) | ProQuest Dissertations & Theses Global | 187704 |
| S5 | S1 AND S2 AND S3 AND S4 | ProQuest Dissertations & Theses Global | 1867 |
| S6 | (S1 AND S2 AND S3 AND S4) AND la.exact("ENG") | ProQuest Dissertations & Theses Global | 1677 |

**Table A1.8(c). International Bibliography of the Social Sciences – Part II**

| Set# | Searched for | Databases | Results |
| --- | --- | --- | --- |
| S1 | ti(violen* OR assault* OR "batter" OR batters OR battere* OR coerc* OR beat* OR stalk* OR rape OR homicid* OR murder* OR kill* OR "intimate terror*" OR "domestic abuse*" OR "sex* abuse*" OR "sex* offen*" OR gangs OR "gang" OR insurgent* OR rebel* OR fighter* OR combatant* OR guerrilla* OR militant* OR soldier*) OR ab(violen* OR assault* OR "batter" OR batters OR battere* OR coerc* OR beat* OR stalk* OR rape OR homicid* OR murder* OR kill* OR "intimate terror*" OR "domestic abuse*" OR "sex* abuse*" OR "sex* offen*" OR gangs OR "gang" OR insurgent* OR rebel* OR fighter* OR combatant* OR guerrilla* OR militant* OR soldier*) OR mainsubject(violen* OR assault* OR "batter" OR batters OR battere* OR coerc* OR beat* OR stalk* OR rape OR homicid* OR murder* OR kill* OR "intimate terror*" OR "domestic abuse*" OR "sex* abuse*" OR "sex* offen*" OR gangs OR "gang" OR insurgent* OR rebel* OR fighter* OR combatant* OR guerrilla* OR militant* OR soldier*) AND pd(20000101-20221231) | International Bibliography of the Social Sciences (IBSS) | 160443 |
| S2 | ti(initiative* OR interven* OR program* OR policy OR policies OR scheme* OR treat* OR approach* OR model* OR strateg* OR method* OR project* OR practice* OR instrument* OR tool* OR framework* OR protocol* OR guid* OR scale* OR system* OR inventor* OR metric* OR template* OR profile* OR criteria OR questionnaire* OR refer* OR assess* OR "case plan*" OR "case formulat*" OR "management plan*" OR "treatment plan*" OR "support plan*" OR "case manage*" OR "risk manage*" OR progress* OR monitor* OR supervis* OR measur*) OR ab(initiative* OR interven* OR program* OR policy OR policies OR scheme* OR treat* OR approach* OR model* OR strateg* OR method* OR project* OR practice* OR instrument* OR tool* OR framework* OR protocol* OR guid* OR scale* OR system* OR inventor* OR metric* OR template* OR profile* OR criteria OR questionnaire* OR refer* OR assess* OR "case plan*" OR "case formulat*" OR "management plan*" OR "treatment plan*" OR "support plan*" OR "case manage*" OR "risk manage*" OR progress* OR monitor* OR supervis* OR measur*) OR mainsubject(initiative* OR interven* OR program* OR policy OR policies OR scheme* OR treat* OR approach* OR model* OR strateg* OR method* OR project* OR practice* OR instrument* OR tool* OR framework* OR protocol* OR guid* OR scale* OR system* OR inventor* OR metric* OR template* OR profile* OR criteria OR questionnaire* OR refer* OR assess* OR "case plan*" OR "case formulat*" OR "management plan*" OR "treatment plan*" OR "support plan*" OR "case manage*" OR "risk manage*" OR progress* OR monitor* OR supervis* OR measur*) AND pd(20000101-20221231) | International Bibliography of the Social Sciences (IBSS) | 2069233 |
| S3 | ti(prevent* OR reduc* OR counter* OR disengage* OR rehab* OR reintegrat* OR re-integrat* OR re-entry OR reentry OR desist* OR recidivism OR deradical* OR de-radical* OR exit* OR de-mobili* OR demobili* OR disarm*) OR ab(prevent* OR reduc* OR counter* OR disengage* OR rehab* OR reintegrat* OR re-integrat* OR re-entry OR reentry OR desist* OR recidivism OR deradical* OR de-radical* OR exit* OR de-mobili* OR demobili* OR disarm*) OR mainsubject(prevent* OR reduc* OR counter* OR disengage* OR rehab* OR reintegrat* OR re-integrat* OR re-entry OR reentry OR desist* OR recidivism OR deradical* OR de-radical* OR exit* OR de-mobili* OR demobili* OR disarm*) AND pd(20000101-20221231) | International Bibliography of the Social Sciences (IBSS) | 299758 |
| S4 | ti(review* OR meta-analy*) OR ab(review* OR meta-analy*) OR mainsubject(review* OR meta-analy*) AND pd(20000101-20221231) | International Bibliography of the Social Sciences (IBSS) | 371777 |
| S5 | S1 AND S2 AND S3 AND S4 | International Bibliography of the Social Sciences (IBSS) | 1725 |
| S6 | (S1 AND S2 AND S3 AND S4) AND la.exact("ENG") | International Bibliography of the Social Sciences (IBSS) | 1653 |
| S7 | (S1 AND S2 AND S3 AND S4) AND (la.exact("ENG") NOT stype.exact("Newspapers")) | International Bibliography of the Social Sciences (IBSS) | 1647 |
| S8 | (S1 AND S2 AND S3 AND S4) NOT (at.exact("General Information" OR "Editorial" OR "News" OR "Letter to the Editor") AND la.exact("ENG") NOT stype.exact("Newspapers")) | International Bibliography of the Social Sciences (IBSS) | 1611 |

**Table A1.9(c). Sociological Abstracts – Part II**

| Set# | Searched for | Databases | Results |
| --- | --- | --- | --- |
| S1 | ti(violen* OR assault* OR "batter" OR batters OR battere* OR coerc* OR beat* OR stalk* OR rape OR homicid* OR murder* OR kill* OR "intimate terror*" OR "domestic abuse*" OR "sex* abuse*" OR "sex* offen*" OR gangs OR "gang" OR insurgent* OR rebel* OR fighter* OR combatant* OR guerrilla* OR militant* OR soldier*) OR ab(violen* OR assault* OR "batter" OR batters OR battere* OR coerc* OR beat* OR stalk* OR rape OR homicid* OR murder* OR kill* OR "intimate terror*" OR "domestic abuse*" OR "sex* abuse*" OR "sex* offen*" OR gangs OR "gang" OR insurgent* OR rebel* OR fighter* OR combatant* OR guerrilla* OR militant* OR soldier*) OR mainsubject(violen* OR assault* OR "batter" OR batters OR battere* OR coerc* OR beat* OR stalk* OR rape OR homicid* OR murder* OR kill* OR "intimate terror*" OR "domestic abuse*" OR "sex* abuse*" OR "sex* offen*" OR gangs OR "gang" OR insurgent* OR rebel* OR fighter* OR combatant* OR guerrilla* OR militant* OR soldier*) AND pd(20000101-20221231) | Sociological Abstracts | 128182 |
| S2 | ti(initiative* OR interven* OR program* OR policy OR policies OR scheme* OR treat* OR approach* OR model* OR strateg* OR method* OR project* OR practice* OR instrument* OR tool* OR framework* OR protocol* OR guid* OR scale* OR system* OR inventor* OR metric* OR template* OR profile* OR criteria OR questionnaire* OR refer* OR assess* OR "case plan*" OR "case formulat*" OR "management plan*" OR "treatment plan*" OR "support plan*" OR "case manage*" OR "risk manage*" OR progress* OR monitor* OR supervis* OR measur*) OR ab(initiative* OR interven* OR program* OR policy OR policies OR scheme* OR treat* OR approach* OR model* OR strateg* OR method* OR project* OR practice* OR instrument* OR tool* OR framework* OR protocol* OR guid* OR scale* OR system* OR inventor* OR metric* OR template* OR profile* OR criteria OR questionnaire* OR refer* OR assess* OR "case plan*" OR "case formulat*" OR "management plan*" OR "treatment plan*" OR "support plan*" OR "case manage*" OR "risk manage*" OR progress* OR monitor* OR supervis* OR measur*) OR mainsubject(initiative* OR interven* OR program* OR policy OR policies OR scheme* OR treat* OR approach* OR model* OR strateg* OR method* OR project* OR practice* OR instrument* OR tool* OR framework* OR protocol* OR guid* OR scale* OR system* OR inventor* OR metric* OR template* OR profile* OR criteria OR questionnaire* OR refer* OR assess* OR "case plan*" OR "case formulat*" OR "management plan*" OR "treatment plan*" OR "support plan*" OR "case manage*" OR "risk manage*" OR progress* OR monitor* OR supervis* OR measur*) AND pd(20000101-20221231) | Sociological Abstracts | 1019203 |
| S3 | ti(prevent* OR reduc* OR counter* OR disengage* OR rehab* OR reintegrat* OR re-integrat* OR re-entry OR reentry OR desist* OR recidivism OR deradical* OR de-radical* OR exit* OR de-mobili* OR demobili* OR disarm*) OR ab(prevent* OR reduc* OR counter* OR disengage* OR rehab* OR reintegrat* OR re-integrat* OR re-entry OR reentry OR desist* OR recidivism OR deradical* OR de-radical* OR exit* OR de-mobili* OR demobili* OR disarm*) OR mainsubject(prevent* OR reduc* OR counter* OR disengage* OR rehab* OR reintegrat* OR re-integrat* OR re-entry OR reentry OR desist* OR recidivism OR deradical* OR de-radical* OR exit* OR de-mobili* OR demobili* OR disarm*) AND pd(20000101-20221231) | Sociological Abstracts | 168548 |
| S4 | ti(review* OR meta-analy*) OR ab(review* OR meta-analy*) OR mainsubject(review* OR meta-analy*) AND pd(20000101-20221231) | Sociological Abstracts | 158864 |
| S5 | S1 AND S2 AND S3 AND S4 | Sociological Abstracts | 2156 |
| S6 | (S1 AND S2 AND S3 AND S4) NOT at.exact("General Information" OR "Editorial" OR "Letter to the Editor" OR "News") | Sociological Abstracts | 2145 |
| S7 | (S1 AND S2 AND S3 AND S4) NOT (at.exact("General Information" OR "Editorial" OR "Letter to the Editor" OR "News") AND pd(20000101-20221231)) | Sociological Abstracts | 2145 |
| S8 | (S1 AND S2 AND S3 AND S4) NOT (at.exact("General Information" OR "Editorial" OR "Letter to the Editor" OR "News") AND la.exact("ENG") AND pd(20000101-20221231)) | Sociological Abstracts | 2086 |

**Table A1.10(c). Web of Science Searches – Part II**

| #8 | (#4 AND #3 AND #2 AND #1) AND ((LA==("ENGLISH")) NOT (DT==("BOOK REVIEW" OR "EDITORIAL MATERIAL" OR "LETTER") OR SJ==("POLYMER SCIENCE" OR "METALLURGY METALLURGICAL ENGINEERING" OR "MARINE FRESHWATER BIOLOGY" OR "HISTORY PHILOSOPHY OF SCIENCE" OR "TRANSPLANTATION" OR "PLANT SCIENCES" OR "PHYSICAL GEOGRAPHY" OR "MECHANICS" OR "HEMATOLOGY" OR "EVOLUTIONARY BIOLOGY" OR "ENTOMOLOGY" OR "CONSTRUCTION BUILDING TECHNOLOGY" OR "ARCHITECTURE" OR "PHYSICS" OR "ENERGY FUELS" OR "ALLERGY" OR "OTORHINOLARYNGOLOGY" OR "TOXICOLOGY" OR "BIOTECHNOLOGY APPLIED MICROBIOLOGY" OR "REPRODUCTIVE BIOLOGY" OR "FORESTRY" OR "BIOPHYSICS" OR "ZOOLOGY" OR "BIODIVERSITY CONSERVATION" OR "PARASITOLOGY" OR "GEOLOGY" OR "DERMATOLOGY" OR "WATER RESOURCES" OR "VIROLOGY" OR "MATERIALS SCIENCE" OR "GASTROENTEROLOGY HEPATOLOGY" OR "CELL BIOLOGY" OR "METEOROLOGY ATMOSPHERIC SCIENCES" OR "UROLOGY NEPHROLOGY" OR "TROPICAL MEDICINE" OR "ANESTHESIOLOGY" OR "GEOGRAPHY" OR "ORTHOPEDICS" OR "ENDOCRINOLOGY METABOLISM" OR "AGRICULTURE" OR "RESPIRATORY SYSTEM" OR "BIOCHEMISTRY MOLECULAR BIOLOGY" OR "FOOD SCIENCE TECHNOLOGY" OR "TRANSPORTATION" OR "ONCOLOGY" OR "CARDIOVASCULAR SYSTEM CARDIOLOGY" OR "ENGINEERING" OR "IMMUNOLOGY" OR "INFECTIOUS DISEASES" OR "OBSTETRICS GYNECOLOGY" OR "ENVIRONMENTAL SCIENCES ECOLOGY")))  5:51 PM | Timespan: 2000-01-01 to 2022-12-31 (Index Date)  A&HCI , BKCI-SSH , ESCI , CPCI-SSH , SSCI | 6,739 |
| --- | --- | --- |
| #7 | (#4 AND #3 AND #2 AND #1) AND ((LA==("ENGLISH")) NOT (DT==("BOOK REVIEW" OR "EDITORIAL MATERIAL" OR "LETTER")))  5:48 PM | Timespan: 2000-01-01 to 2022-12-31 (Index Date)  A&HCI , BKCI-SSH , ESCI , CPCI-SSH , SSCI | 7,532 |
| #6 | (#4 AND #3 AND #2 AND #1) NOT (DT==("BOOK REVIEW" OR "EDITORIAL MATERIAL" OR "LETTER"))  5:48 PM | Timespan: 2000-01-01 to 2022-12-31 (Index Date)  A&HCI , BKCI-SSH , ESCI , CPCI-SSH , SSCI | 8,074 |
| #5 | #4 AND #3 AND #2 AND #1  3:48 PM | Timespan: 2000-12-31 to 2022-08-31 (Index Date)  A&HCI , BKCI-SSH , ESCI , CPCI-SSH , SSCI | 8,031 |
| #4 | TS=(review* OR meta-analy*)  3:47 PM | Timespan: 2000-01-01 to 2022-12-31 (Index Date) | Exact search  A&HCI , BKCI-SSH , ESCI , CPCI-SSH , SSCI | 869,432 |
| #3 | TS=(prevent* OR reduc* OR counter* OR disengage* OR rehab* OR reintegrat* OR re-integrat* OR re-entry OR reentry OR desist* OR recidivism OR deradical* OR de-radical* OR exit* OR de-mobili* OR demobili* OR disarm*)  3:47 PM | Timespan: 2000-01-01 to 2022-12-31 (Index Date) | Exact search  A&HCI , BKCI-SSH , ESCI , CPCI-SSH , SSCI | 1,639,684 |
| #2 | TS=(initiative* OR interven* OR program* OR policy OR scheme* OR treat* OR approach* OR model* OR strateg* OR method* OR project* OR practice* OR instrument* OR tool* OR framework* OR protocol* OR guid* OR scale* OR system* OR inventor* OR metric* OR template* OR profile* OR criteria OR questionnaire* OR refer* OR assess* OR "case plan*" OR "case formulat*" OR "management plan*" OR "treatment plan*" OR "support plan*" OR "case manage*" OR "risk manage*" OR progress* OR monitor* OR supervis* OR measur*)  3:46 PM | Timespan: 2000-01-01 to 2022-12-31 (Index Date) | Exact search  A&HCI , BKCI-SSH , ESCI , CPCI-SSH , SSCI | 7,949,100 |
| #1 | TS=(violen* OR assault* OR "batter" OR batters OR battere* OR coerc* OR beat* OR stalk* OR rape OR homicid* OR murder* OR kill* OR "intimate terror*" OR "domestic abuse*" OR "sex* abuse*" OR "sex* offen*" OR gangs OR "gang" OR insurgent* OR rebel* OR fighter* OR combatant* OR guerrilla* OR militant* OR soldier*)  3:46 PM | Timespan: 2000-01-01 to 2022-12-31 (Index Date) | Exact search  A&HCI , BKCI-SSH , ESCI , CPCI-SSH , SSCI | 346,731 |

**Appendix I (D) Part II Search Record (LOE)**

**Table A1.1(d) Academic Platform Search Record – Part II**

| **Search Source** | **Source** | **Search Date)** | **Date Coverage for Search** | **Results** |
| --- | --- | --- | --- | --- |
| Criminal Justice Abstracts | EBSCO | 13/10/2022 | 01/01/2000 - 13/10/2022 | 54 |
| Scopus | Elsevier | 13/10/2022 | 01/01/2000 - 13/10/2022 | 1,993 |
| Medline | Ovid | 13/10/2022 | 01/01/2000 - 13/10/2022 | 883* |
| PsycInfo | Ovid | 13/10/2022 | 01/01/2000 - 13/10/2022 | 244* |
| Dissertations and Theses Global | ProQuest | 13/10/2022 | 01/01/2000 - 13/10/2022 | 191* |
| International Bibliography of the Social Sciences | ProQuest | 13/10/2022 | 01/01/2000 - 13/10/2022 | 80* |
| Sociological Abstracts | ProQuest | 13/10/2022 | 01/01/2000 - 13/10/2022 | 61* |
| Book Citation Index – Social Sciences & Humanities (BKCI-SSH)  Social Sciences Citation Index (SSCI)  Arts & Humanities Citation Index (A&HCI)  Emerging Sources Citation Index (ESCI)  Conference Proceedings Citation Index – Social Science & Humanities (CPCI-SSH) | Web of Science | 13/10/2022 | 01/01/2000 - 12/10/2022 | 598 |

* Unable to search LOE keywords due to unsupported characters.

**Table A1.2(d). Criminal Justice Abstracts Search Records – Part II (LOE)**

English Key Words

| **#** | **Query** | **Limiters/Expanders** | **Last Run Via** | **Results** |
| --- | --- | --- | --- | --- |
| S26 | S22 AND S23 AND S24 AND S25 | Limiters - Publication Date: 20000101-20221231; Language: Croatian, Czech, Danish, Dutch/Flemish, French, German, Italian, Lithuanian, Norwegian, Portuguese, Slovak, Slovenian, Spanish, Swedish | Interface - EBSCOhost Research Databases | 38 |
| S25 | TI ( review* OR meta-analy* ) OR AB ( review or meta-analysis ) OR KW ( review* OR meta-analy* ) OR SU ( review* OR meta-analy* ) | Search modes - Boolean/Phrase  Limiters - Publication Date: 20000101-20221231 | Search Screen - Advanced Search | 59,974 |
|  |  |  | Database - Criminal Justice Abstracts |  |
|  |  |  | Interface - EBSCOhost Research Databases |  |
| S24 | TI ( prevent* OR reduc* OR counter* OR disengage* OR rehab* OR reintegrat* OR re-integrat* OR re-entry OR reentry OR desist* OR recidivism OR deradical* OR de-radical* OR exit* OR de-mobili* OR demobili* OR disarm* ) OR AB ( prevent* OR reduc* OR counter* OR disengage* OR rehab* OR reintegrat* OR re-integrat* OR re-entry OR reentry OR desist* OR recidivism OR deradical* OR de-radical* OR exit* OR de-mobili* OR demobili* OR disarm* ) OR KW ( prevent* OR reduc* OR counter* OR disengage* OR rehab* OR reintegrat* OR re-integrat* OR re-entry OR reentry OR desist* OR recidivism OR deradical* OR de-radical* OR exit* OR de-mobili* OR demobili* OR disarm* ) OR SU ( prevent* OR reduc* OR counter* OR disengage* OR rehab* OR reintegrat* OR re-integrat* OR re-entry OR reentry OR desist* OR recidivism OR deradical* OR de-radical* OR exit* OR de-mobili* OR demobili* OR disarm* ) | Search modes - Boolean/Phrase | Search Screen - Advanced Search | 112,473 |
|  |  |  | Database - Criminal Justice Abstracts |  |
|  |  | Limiters - Publication Date: 20000101-20221231 | Interface - EBSCOhost Research Databases |  |
| S23 | TI ( initiative* OR interven* OR program* OR policy OR policies OR scheme* OR treat* OR approach* OR model* OR strateg* OR method* OR project* OR practice* OR instrument* OR tool* OR framework* OR protocol* OR guid* OR scale* OR system* OR inventor* OR metric* OR template* OR profile* OR criteria OR questionnaire* OR refer* OR assess* OR "case plan*" OR "case formulat*" OR "management plan*" OR "treatment plan*" OR "support plan*" OR "case manage*" OR "risk manage*" OR progress* OR monitor* OR supervis* OR measur* ) OR AB ( initiative* OR interven* OR program* OR policy OR policies OR scheme* OR treat* OR approach* OR model* OR strateg* OR method* OR project* OR practice* OR instrument* OR tool* OR framework* OR protocol* OR guid* OR scale* OR system* OR inventor* OR metric* OR template* OR profile* OR criteria OR questionnaire* OR refer* OR assess* OR "case plan*" OR "case formulat*" OR "management plan*" OR "treatment plan*" OR "support plan*" OR "case manage*" OR "risk manage*" OR progress* OR monitor* OR supervis* OR measur* ) OR KW ( initiative* OR interven* OR program* OR policy OR policies OR scheme* OR treat* OR approach* OR model* OR strateg* OR method* OR project* OR practice* OR instrument* OR tool* OR framework* OR protocol* OR guid* OR scale* OR system* OR inventor* OR metric* OR template* OR profile* OR criteria OR questionnaire* OR refer* OR assess* OR "case plan*" OR "case formulat*" OR "management plan*" OR "treatment plan*" OR "support plan*" OR "case manage*" OR "risk manage*" OR progress* OR monitor* OR supervis* OR measur* ) OR SU ( initiative* OR interven* OR program* OR policy OR policies OR scheme* OR treat* OR approach* OR model* OR strateg* OR method* OR project* OR practice* OR instrument* OR tool* OR framework* OR protocol* OR guid* OR scale* OR system* OR inventor* OR metric* OR template* OR profile* OR criteria OR questionnaire* OR refer* OR assess* OR "case plan*" OR "case formulat*" OR "management plan*" OR "treatment plan*" OR "support plan*" OR "case manage*" OR "risk manage*" OR progress* OR monitor* OR supervis* OR measur* ) | Search modes - Boolean/Phrase | Search Screen - Advanced Search | 364,019 |
|  |  |  | Database - Criminal Justice Abstracts |  |
|  |  | Limiters - Publication Date: 20000101-20221231 | Interface - EBSCOhost Research Databases |  |
| S22 | TI ( violen* OR assault* OR "batter" OR batters OR battere* OR coerc* OR beat* OR stalk* OR rape OR homicid* OR murder* OR kill* OR "intimate terror*" OR "domestic abuse*" OR "sex* abuse*" OR "sex* offen*" OR gangs OR "gang" OR insurgent* OR rebel* OR fighter* OR combatant* OR guerrilla* OR militant* OR soldier* ) OR AB ( violen* OR assault* OR "batter" OR batters OR battere* OR coerc* OR beat* OR stalk* OR rape OR homicid* OR murder* OR kill* OR "intimate terror*" OR "domestic abuse*" OR "sex* abuse*" OR "sex* offen*" OR gangs OR "gang" OR insurgent* OR rebel* OR fighter* OR combatant* OR guerrilla* OR militant* OR soldier* ) OR KW ( violen* OR assault* OR "batter" OR batters OR battere* OR coerc* OR beat* OR stalk* OR rape OR homicid* OR murder* OR kill* OR "intimate terror*" OR "domestic abuse*" OR "sex* abuse*" OR "sex* offen*" OR gangs OR "gang" OR insurgent* OR rebel* OR fighter* OR combatant* OR guerrilla* OR militant* OR soldier* ) OR SU ( violen* OR assault* OR "batter" OR batters OR battere* OR coerc* OR beat* OR stalk* OR rape OR homicid* OR murder* OR kill* OR "intimate terror*" OR "domestic abuse*" OR "sex* abuse*" OR "sex* offen*" OR gangs OR "gang" OR insurgent* OR rebel* OR fighter* OR combatant* OR guerrilla* OR militant* OR soldier* ) | Limiters - Publication Date: 20000101-20221231 | Interface - EBSCOhost Research Databases | 81421 |
|  |  | Search modes - Boolean/Phrase | Search Screen - Advanced Search |  |
|  |  |  | Database - Criminal Justice Abstracts |  |

LOE Key Words

| **#** | **Query** | **Limiters/Expanders** | **Last Run Via** | **Results** |
| --- | --- | --- | --- | --- |
| S21 | S5 AND S10 AND S15 AND S20 | Limiters - Publication Date: 20000101-20221231; Language: Croatian, Czech, Danish, Dutch/Flemish, French, German, Italian, Lithuanian, Norwegian, Portuguese, Slovak, Slovenian, Spanish, Swedish | Interface - EBSCOhost Research Databases | 16 |
|  |  | Search modes - Boolean/Phrase | Search Screen - Advanced Search |  |
|  |  |  | Database - Criminal Justice Abstracts |  |
| S20 | S16 OR S17 OR S18 OR S19 | Expanders - Apply equivalent subjects | Interface - EBSCOhost Research Databases | 1,339 |
|  |  | Search modes - Boolean/Phrase | Search Screen - Advanced Search |  |
|  |  |  | Database - Criminal Justice Abstracts |  |
| S19 | SU ( systématique OR méta-analyse* OR revue* OR recension ) OR SU ( meta-Analyse* OR Rezens* OR Bewert* ) OR SU ( метаанали* OR мета-анали* OR обзор* ) OR SU ( meta-analytisk OR gennemgang ) OR SU ( meta-analyse OR anmeldelse ) OR SU ( meta-analys OR recension ) | Expanders - Apply equivalent subjects | Interface - EBSCOhost Research Databases | 5 |
|  |  | Search modes - Boolean/Phrase | Search Screen - Advanced Search |  |
|  |  |  | Database - Criminal Justice Abstracts |  |
| S18 | KW ( systématique OR méta-analyse* OR revue* OR recension ) OR KW ( meta-Analyse* OR Rezens* OR Bewert* ) OR KW ( метаанали* OR мета-анали* OR обзор* ) OR KW ( meta-analytisk OR gennemgang ) OR KW ( meta-analyse OR anmeldelse ) OR KW ( meta-analys OR recension ) | Expanders - Apply equivalent subjects | Interface - EBSCOhost Research Databases | 28 |
|  |  | Search modes - Boolean/Phrase | Search Screen - Advanced Search |  |
|  |  |  | Database - Criminal Justice Abstracts |  |
| S17 | AB ( systématique OR méta-analyse* OR revue* OR recension ) OR AB ( meta-Analyse* OR Rezens* OR Bewert* ) OR AB ( метаанали* OR мета-анали* OR обзор* ) OR AB ( meta-analytisk OR gennemgang ) OR AB ( meta-analyse OR anmeldelse ) OR AB ( meta-analys OR recension ) | Expanders - Apply equivalent subjects | Interface - EBSCOhost Research Databases | 1,152 |
|  |  | Search modes - Boolean/Phrase | Search Screen - Advanced Search |  |
|  |  |  | Database - Criminal Justice Abstracts |  |
| S16 | TI ( systématique OR méta-analyse* OR revue* OR recension ) OR TI ( meta-Analyse* OR Rezens* OR Bewert* ) OR TI ( метаанали* OR мета-анали* OR обзор* ) OR TI ( meta-analytisk OR gennemgang ) OR TI ( meta-analyse OR anmeldelse ) OR TI ( meta-analys OR recension ) | Expanders - Apply equivalent subjects | Interface - EBSCOhost Research Databases | 227 |
|  |  | Search modes - Boolean/Phrase | Search Screen - Advanced Search |  |
|  |  |  | Database - Criminal Justice Abstracts |  |
| S15 | S11 OR S12 OR S13 OR S14 | Expanders - Apply equivalent subjects | Interface - EBSCOhost Research Databases | 120,745 |
|  |  | Search modes - Boolean/Phrase | Search Screen - Advanced Search |  |
|  |  |  | Database - Criminal Justice Abstracts |  |
| S14 | SU ( prévent* OR réduct* OR anti* OR contre* OR désengage* OR réhab* OR désendoctrin* OR désembriga* OR réinser* OR ré-inser* OR récidiv* OR réintégr* OR ré-intégr* OR renonc* OR désist* OR démobilis* OR désarm* ) OR SU ( verhinder* OR reduzier* OR minder* OR bekämpf* OR loslösen* OR rehab* OR resozialisier* OR reintegrat* OR wiedereinglieder* OR Abstrandn* OR unterlass* OR rückfall* OR Rückfäll* OR deradikal* OR aussteige* Aussstieg* demobilisier* OR entwaffn* ) OR SU ( предупре* OR профилактик* OR предотвра* OR снижени* OR снизи* OR уменьш* OR противодейств* OR противостоя* OR реабORт* OR реинтегр* OR рецидив* OR демобOR* OR разоруж* ) OR SU ( prevent* OR forebyg* OR reducere* OR formindske OR counter* OR bekæmp* OR disengage* OR afhop* OR rehab* OR reintegrat* OR gen-integrat* OR re-entry OR genindtræde OR return* OR desist* OR afstå* OR "tage afstand fra" OR recidivism OR tilbagfald OR exit* OR demobili* OR afvæbne ) OR SU ( prevent* OR forhindre* OR reducere* OR reduser* OR counter* OR kamp OR disengage* OR afhop* OR rehab* OR reintegrat* OR genintegrer* OR re-entry OR retur* OR desist* OR avstå* OR "avstand fra" OR recidivism OR tilbakefall OR exit* OR demobili* OR avvæpne ) OR SU ( prevent* OR förhindra* OR reducere* OR minska* OR counter* OR bekämpa OR disengage* OR afhop* OR rehab* OR reintegrat* OR återintegrera* OR re-entry OR återinträde OR returnera* OR desist* OR avstå* OR "avstånd från" OR recidivism OR deradikal * OR "falla tillbaka" OR exit* OR utgång* OR demobili* OR avväpna ) | Expanders - Apply equivalent subjects | Interface - EBSCOhost Research Databases | 49,266 |
|  |  | Search modes - Boolean/Phrase | Search Screen - Advanced Search |  |
|  |  |  | Database - Criminal Justice Abstracts |  |
| S13 | KW ( prévent* OR réduct* OR anti* OR contre* OR désengage* OR réhab* OR désendoctrin* OR désembriga* OR réinser* OR ré-inser* OR récidiv* OR réintégr* OR ré-intégr* OR renonc* OR désist* OR démobilis* OR désarm* ) OR KW ( verhinder* OR reduzier* OR minder* OR bekämpf* OR loslösen* OR rehab* OR resozialisier* OR reintegrat* OR wiedereinglieder* OR Abstrandn* OR unterlass* OR rückfall* OR Rückfäll* OR deradikal* OR aussteige* Aussstieg* demobilisier* OR entwaffn* ) OR KW ( предупре* OR профилактик* OR предотвра* OR снижени* OR снизи* OR уменьш* OR противодейств* OR противостоя* OR реабORт* OR реинтегр* OR рецидив* OR демобOR* OR разоруж* ) OR KW ( prevent* OR forebyg* OR reducere* OR formindske OR counter* OR bekæmp* OR disengage* OR afhop* OR rehab* OR reintegrat* OR gen-integrat* OR re-entry OR genindtræde OR return* OR desist* OR afstå* OR "tage afstand fra" OR recidivism OR tilbagfald OR exit* OR demobili* OR afvæbne ) OR KW ( prevent* OR forhindre* OR reducere* OR reduser* OR counter* OR kamp OR disengage* OR afhop* OR rehab* OR reintegrat* OR genintegrer* OR re-entry OR retur* OR desist* OR avstå* OR "avstand fra" OR recidivism OR tilbakefall OR exit* OR demobili* OR avvæpne ) OR KW ( prevent* OR förhindra* OR reducere* OR minska* OR counter* OR bekämpa OR disengage* OR afhop* OR rehab* OR reintegrat* OR återintegrera* OR re-entry OR återinträde OR returnera* OR desist* OR avstå* OR "avstånd från" OR recidivism OR deradikal * OR "falla tillbaka" OR exit* OR utgång* OR demobili* OR avväpna ) | Expanders - Apply equivalent subjects | Interface - EBSCOhost Research Databases | 15,451 |
|  |  | Search modes - Boolean/Phrase | Search Screen - Advanced Search |  |
|  |  |  | Database - Criminal Justice Abstracts |  |
| S12 | AB ( prévent* OR réduct* OR anti* OR contre* OR désengage* OR réhab* OR désendoctrin* OR désembriga* OR réinser* OR ré-inser* OR récidiv* OR réintégr* OR ré-intégr* OR renonc* OR désist* OR démobilis* OR désarm* ) OR AB ( verhinder* OR reduzier* OR minder* OR bekämpf* OR loslösen* OR rehab* OR resozialisier* OR reintegrat* OR wiedereinglieder* OR Abstrandn* OR unterlass* OR rückfall* OR Rückfäll* OR deradikal* OR aussteige* Aussstieg* demobilisier* OR entwaffn* ) OR AB ( предупре* OR профилактик* OR предотвра* OR снижени* OR снизи* OR уменьш* OR противодейств* OR противостоя* OR реабORт* OR реинтегр* OR рецидив* OR демобOR* OR разоруж* ) OR AB ( prevent* OR forebyg* OR reducere* OR formindske OR counter* OR bekæmp* OR disengage* OR afhop* OR rehab* OR reintegrat* OR gen-integrat* OR re-entry OR genindtræde OR return* OR desist* OR afstå* OR "tage afstand fra" OR recidivism OR tilbagfald OR exit* OR demobili* OR afvæbne ) OR AB ( prevent* OR forhindre* OR reducere* OR reduser* OR counter* OR kamp OR disengage* OR afhop* OR rehab* OR reintegrat* OR genintegrer* OR re-entry OR retur* OR desist* OR avstå* OR "avstand fra" OR recidivism OR tilbakefall OR exit* OR demobili* OR avvæpne ) OR AB ( prevent* OR förhindra* OR reducere* OR minska* OR counter* OR bekämpa OR disengage* OR afhop* OR rehab* OR reintegrat* OR återintegrera* OR re-entry OR återinträde OR returnera* OR desist* OR avstå* OR "avstånd från" OR recidivism OR deradikal * OR "falla tillbaka" OR exit* OR utgång* OR demobili* OR avväpna ) | Expanders - Apply equivalent subjects | Interface - EBSCOhost Research Databases | 96,717 |
|  |  | Search modes - Boolean/Phrase | Search Screen - Advanced Search |  |
|  |  |  | Database - Criminal Justice Abstracts |  |
| S11 | TI ( prévent* OR réduct* OR anti* OR contre* OR désengage* OR réhab* OR désendoctrin* OR désembriga* OR réinser* OR ré-inser* OR récidiv* OR réintégr* OR ré-intégr* OR renonc* OR désist* OR démobilis* OR désarm* ) OR TI ( verhinder* OR reduzier* OR minder* OR bekämpf* OR loslösen* OR rehab* OR resozialisier* OR reintegrat* OR wiedereinglieder* OR Abstrandn* OR unterlass* OR rückfall* OR Rückfäll* OR deradikal* OR aussteige* Aussstieg* demobilisier* OR entwaffn* ) OR TI ( предупре* OR профилактик* OR предотвра* OR снижени* OR снизи* OR уменьш* OR противодейств* OR противостоя* OR реабORт* OR реинтегр* OR рецидив* OR демобOR* OR разоруж* ) OR TI ( prevent* OR forebyg* OR reducere* OR formindske OR counter* OR bekæmp* OR disengage* OR afhop* OR rehab* OR reintegrat* OR gen-integrat* OR re-entry OR genindtræde OR return* OR desist* OR afstå* OR "tage afstand fra" OR recidivism OR tilbagfald OR exit* OR demobili* OR afvæbne ) OR TI ( prevent* OR forhindre* OR reducere* OR reduser* OR counter* OR kamp OR disengage* OR afhop* OR rehab* OR reintegrat* OR genintegrer* OR re-entry OR retur* OR desist* OR avstå* OR "avstand fra" OR recidivism OR tilbakefall OR exit* OR demobili* OR avvæpne ) OR TI ( prevent* OR förhindra* OR reducere* OR minska* OR counter* OR bekämpa OR disengage* OR afhop* OR rehab* OR reintegrat* OR återintegrera* OR re-entry OR återinträde OR returnera* OR desist* OR avstå* OR "avstånd från" OR recidivism OR deradikal * OR "falla tillbaka" OR exit* OR utgång* OR demobili* OR avväpna ) | Expanders - Apply equivalent subjects | Interface - EBSCOhost Research Databases | 24,875 |
|  |  | Search modes - Boolean/Phrase | Search Screen - Advanced Search |  |
|  |  |  | Database - Criminal Justice Abstracts |  |
| S10 | S6 OR S7 OR S8 OR S9 | Expanders - Apply equivalent subjects | Interface - EBSCOhost Research Databases | 408,614 |
|  |  | Search modes - Boolean/Phrase | Search Screen - Advanced Search |  |
|  |  |  | Database - Criminal Justice Abstracts |  |
| S9 | SU ( politique OR initiative* OR interven* OR progra* OR plan* OR traitement* OR approche* OR modèl* OR stratégi* OR méthod* OR projet* OR pratique* OR instrument* OR outil* OR cadre* OR protocole* OR guide* OR échelle* OR système* OR inventaire* OR métrique* gabarit* OR profile* OR critère* OR questionnaire* OR réfer* OR évalu* OR "plan d’intervention" OR "plan de traitement" OR "plan de gestion" OR "programme de gestion" OR "plan d’encadrement" OR "plan d’action" OR "programme de traitement" OR "plan de soins" OR "programme de soins" OR "projet de traitement" OR "plan thérapeutique" OR "programme thérapeutique" OR "plan de soutien" OR "plan d’accompagnement" OR "gestion de cas" OR "gestion de dossier" OR "gestion de risque*" OR "gestion des risques" OR progrès* OR évolution* OR progression* OR supervision* OR observation* OR surveill* OR suivi* OR mesure* ) OR SU ( initiative* OR interven* OR programm* OR Schema* OR behand* OR Ansatz* OR modell* OR strateg* OR Method* OR Projekt* OR praxis* OR instrument* OR Werkzeug* OR Rahmen* OR protokoll* OR anleit* OR Handlungsempfehlung* OR Ausmaß* OR Maßstab* OR system* OR Erfinder* OR metri* OR Vorlage* OR Profil* OR Kriteri* OR Umfrage* OR Einliefer* OR Einweis* OR Beurteil* OR Einschätz* OR "Fallplan*" OR "Fallgestaltung*" OR "Managementplan*" OR "Behandlungsplan*" OR "Unterstützungsplan*" OR "Förderplan*" OR "Fallmanagement*" OR "Fallbearbeitung*" OR "Risikomanagement*" OR "Risikobewältigung*" OR Forschritt* OR Entwicklung* OR beobacht* OR überwach* OR beaufsichtig* OR Aufsicht* OR Aufseher* OR Maßnahme* OR Messen* ) OR SU ( Инициатив* OR Вмешательств* OR программ* OR политик* OR схема* OR терап* OR подход* OR модель OR стратеги* метод* OR проект* OR практи* OR инструмент* OR концепци* OR структур* OR протокол* OR инструк* OR принцип* OR разработк* OR масштаб* OR системн* OR пример* OR обзор* OR критери* OR опросник* OR направлен* OR консульта* OR оцен* OR диагности* OR план* OR "план развития" OR мониторинг* OR "план ведения" OR "план* поддерж*" OR "управлени* риск*" OR развитие OR контрол* OR наблюд* OR оцен* OR измер* ) OR SU ( initiativ* OR interven* OR program* OR tilgang OR policy OR behandling OR model* OR strategi* OR metod* OR projekt OR instrument* OR tool* OR redskab OR framework* OR ramme OR protokol* OR analyse OR guide* OR vejledning OR skala OR system* OR indeks OR beholdning OR matrix OR format OR retningslin* OR template* OR skabelon OR profil* OR kriterie* OR spørgeskema OR refer* OR henvisning OR assess* OR vurde* OR måling OR screen* OR "case plan*" OR "case formulering" OR "management plan*" OR "treatment plan*" OR "behandlingsplan" OR "behandlingsforløb" OR "behandlingsprogram" OR "support plan*" OR "støtteforløb" OR "case manage*" OR "Sagsbehandling*" OR plan* OR "risikohåndtering*" OR "sikkerhedsvurdering" OR "sikkerhedsanalyse" OR progress OR fremskridt OR forbedringer OR monitor* OR supervis* OR måle* OR måling ) OR SU ( initiativ* OR intervensjon OR program* OR adgang OR policy OR behandle OR modell OR strategi* OR metod* OR prosjekt OR instrument OR tool* OR verktøy OR framework* OR rammeverk OR protokoll OR analyse OR guide* OR veiledning OR skala OR system* OR indeks OR inventar OR matrix OR metrisk OR format OR retningslinjer OR template* OR mal OR profil* OR kriterie* OR spørreskjema OR refer* OR referanse OR assess* OR vurdere* OR måling OR screen* OR "case plan*" OR "Saksplan*" OR "management plan*" OR "treatment plan*" OR "behandlingsplan*" OR "behandlingsforløp" OR "behandlingsprogram" OR "support plan*" OR "Støttekurs" OR "case manage*" OR "Saksbehandling*" OR "plan" OR "risikohåndtering*" OR "Sikkerhetsvurdering" OR "sikkerhetsanalyse" OR progress OR fremgang OR forbedringer OR monitor* OR tilsyn* OR måle* OR mål ) OR SU ( initiativ* OR intervensjon OR program* OR tillgång OR policy OR behandla* OR model* OR strateg* OR metod* OR projekt OR instrument* OR tool* OR verktyg OR framework* OR ram OR protokoll OR analyse OR guide* OR vägledning OR skala OR system* OR indeks OR inventering OR matrix OR metrisk OR format* OR riktlinj* OR template* OR mall OR profil* OR kriterie* OR frågeformulär OR refer* OR referens OR assess* OR bedöm* OR mätning OR screen* OR "case plan*" OR "Fallformulering" OR "management plan*" OR "treatment plan*" OR "behandlingsplan*" OR "behandlingsförlopp" OR "behandlingsprogram"OR "support plan*" OR "case manage*" OR "plan" OR "Planera" OR "Riskhantering*" OR "Säkerhetsbedömning" OR "Säkerhetsanalys" OR progress OR framsteg OR förbättringar OR monitor* OR övervaka* OR mäta* OR mått ) | Expanders - Apply equivalent subjects | Interface - EBSCOhost Research Databases | 137,808 |
|  |  | Search modes - Boolean/Phrase | Search Screen - Advanced Search |  |
|  |  |  | Database - Criminal Justice Abstracts |  |
| S8 | KW ( politique OR initiative* OR interven* OR progra* OR plan* OR traitement* OR approche* OR modèl* OR stratégi* OR méthod* OR projet* OR pratique* OR instrument* OR outil* OR cadre* OR protocole* OR guide* OR échelle* OR système* OR inventaire* OR métrique* gabarit* OR profile* OR critère* OR questionnaire* OR réfer* OR évalu* OR "plan d’intervention" OR "plan de traitement" OR "plan de gestion" OR "programme de gestion" OR "plan d’encadrement" OR "plan d’action" OR "programme de traitement" OR "plan de soins" OR "programme de soins" OR "projet de traitement" OR "plan thérapeutique" OR "programme thérapeutique" OR "plan de soutien" OR "plan d’accompagnement" OR "gestion de cas" OR "gestion de dossier" OR "gestion de risque*" OR "gestion des risques" OR progrès* OR évolution* OR progression* OR supervision* OR observation* OR surveill* OR suivi* OR mesure* ) OR KW ( initiative* OR interven* OR programm* OR Schema* OR behand* OR Ansatz* OR modell* OR strateg* OR Method* OR Projekt* OR praxis* OR instrument* OR Werkzeug* OR Rahmen* OR protokoll* OR anleit* OR Handlungsempfehlung* OR Ausmaß* OR Maßstab* OR system* OR Erfinder* OR metri* OR Vorlage* OR Profil* OR Kriteri* OR Umfrage* OR Einliefer* OR Einweis* OR Beurteil* OR Einschätz* OR "Fallplan*" OR "Fallgestaltung*" OR "Managementplan*" OR "Behandlungsplan*" OR "Unterstützungsplan*" OR "Förderplan*" OR "Fallmanagement*" OR "Fallbearbeitung*" OR "Risikomanagement*" OR "Risikobewältigung*" OR Forschritt* OR Entwicklung* OR beobacht* OR überwach* OR beaufsichtig* OR Aufsicht* OR Aufseher* OR Maßnahme* OR Messen* ) OR KW ( Инициатив* OR Вмешательств* OR программ* OR политик* OR схема* OR терап* OR подход* OR модель OR стратеги* метод* OR проект* OR практи* OR инструмент* OR концепци* OR структур* OR протокол* OR инструк* OR принцип* OR разработк* OR масштаб* OR системн* OR пример* OR обзор* OR критери* OR опросник* OR направлен* OR консульта* OR оцен* OR диагности* OR план* OR "план развития" OR мониторинг* OR "план ведения" OR "план* поддерж*" OR "управлени* риск*" OR развитие OR контрол* OR наблюд* OR оцен* OR измер* ) OR KW ( initiativ* OR interven* OR program* OR tilgang OR policy OR behandling OR model* OR strategi* OR metod* OR projekt OR instrument* OR tool* OR redskab OR framework* OR ramme OR protokol* OR analyse OR guide* OR vejledning OR skala OR system* OR indeks OR beholdning OR matrix OR format OR retningslin* OR template* OR skabelon OR profil* OR kriterie* OR spørgeskema OR refer* OR henvisning OR assess* OR vurde* OR måling OR screen* OR "case plan*" OR "case formulering" OR "management plan*" OR "treatment plan*" OR "behandlingsplan" OR "behandlingsforløb" OR "behandlingsprogram" OR "support plan*" OR "støtteforløb" OR "case manage*" OR "Sagsbehandling*" OR plan* OR "risikohåndtering*" OR "sikkerhedsvurdering" OR "sikkerhedsanalyse" OR progress OR fremskridt OR forbedringer OR monitor* OR supervis* OR måle* OR måling ) OR KW ( initiativ* OR intervensjon OR program* OR adgang OR policy OR behandle OR modell OR strategi* OR metod* OR prosjekt OR instrument OR tool* OR verktøy OR framework* OR rammeverk OR protokoll OR analyse OR guide* OR veiledning OR skala OR system* OR indeks OR inventar OR matrix OR metrisk OR format OR retningslinjer OR template* OR mal OR profil* OR kriterie* OR spørreskjema OR refer* OR referanse OR assess* OR vurdere* OR måling OR screen* OR "case plan*" OR "Saksplan*" OR "management plan*" OR "treatment plan*" OR "behandlingsplan*" OR "behandlingsforløp" OR "behandlingsprogram" OR "support plan*" OR "Støttekurs" OR "case manage*" OR "Saksbehandling*" OR "plan" OR "risikohåndtering*" OR "Sikkerhetsvurdering" OR "sikkerhetsanalyse" OR progress OR fremgang OR forbedringer OR monitor* OR tilsyn* OR måle* OR mål ) OR KW ( initiativ* OR intervensjon OR program* OR tillgång OR policy OR behandla* OR model* OR strateg* OR metod* OR projekt OR instrument* OR tool* OR verktyg OR framework* OR ram OR protokoll OR analyse OR guide* OR vägledning OR skala OR system* OR indeks OR inventering OR matrix OR metrisk OR format* OR riktlinj* OR template* OR mall OR profil* OR kriterie* OR frågeformulär OR refer* OR referens OR assess* OR bedöm* OR mätning OR screen* OR "case plan*" OR "Fallformulering" OR "management plan*" OR "treatment plan*" OR "behandlingsplan*" OR "behandlingsförlopp" OR "behandlingsprogram"OR "support plan*" OR "case manage*" OR "plan" OR "Planera" OR "Riskhantering*" OR "Säkerhetsbedömning" OR "Säkerhetsanalys" OR progress OR framsteg OR förbättringar OR monitor* OR övervaka* OR mäta* OR mått ) | Expanders - Apply equivalent subjects | Interface - EBSCOhost Research Databases | 47,673 |
|  |  | Search modes - Boolean/Phrase | Search Screen - Advanced Search |  |
|  |  |  | Database - Criminal Justice Abstracts |  |
| S7 | AB ( politique OR initiative* OR interven* OR progra* OR plan* OR traitement* OR approche* OR modèl* OR stratégi* OR méthod* OR projet* OR pratique* OR instrument* OR outil* OR cadre* OR protocole* OR guide* OR échelle* OR système* OR inventaire* OR métrique* gabarit* OR profile* OR critère* OR questionnaire* OR réfer* OR évalu* OR "plan d’intervention" OR "plan de traitement" OR "plan de gestion" OR "programme de gestion" OR "plan d’encadrement" OR "plan d’action" OR "programme de traitement" OR "plan de soins" OR "programme de soins" OR "projet de traitement" OR "plan thérapeutique" OR "programme thérapeutique" OR "plan de soutien" OR "plan d’accompagnement" OR "gestion de cas" OR "gestion de dossier" OR "gestion de risque*" OR "gestion des risques" OR progrès* OR évolution* OR progression* OR supervision* OR observation* OR surveill* OR suivi* OR mesure* ) OR AB ( initiative* OR interven* OR programm* OR Schema* OR behand* OR Ansatz* OR modell* OR strateg* OR Method* OR Projekt* OR praxis* OR instrument* OR Werkzeug* OR Rahmen* OR protokoll* OR anleit* OR Handlungsempfehlung* OR Ausmaß* OR Maßstab* OR system* OR Erfinder* OR metri* OR Vorlage* OR Profil* OR Kriteri* OR Umfrage* OR Einliefer* OR Einweis* OR Beurteil* OR Einschätz* OR "Fallplan*" OR "Fallgestaltung*" OR "Managementplan*" OR "Behandlungsplan*" OR "Unterstützungsplan*" OR "Förderplan*" OR "Fallmanagement*" OR "Fallbearbeitung*" OR "Risikomanagement*" OR "Risikobewältigung*" OR Forschritt* OR Entwicklung* OR beobacht* OR überwach* OR beaufsichtig* OR Aufsicht* OR Aufseher* OR Maßnahme* OR Messen* ) OR AB ( Инициатив* OR Вмешательств* OR программ* OR политик* OR схема* OR терап* OR подход* OR модель OR стратеги* метод* OR проект* OR практи* OR инструмент* OR концепци* OR структур* OR протокол* OR инструк* OR принцип* OR разработк* OR масштаб* OR системн* OR пример* OR обзор* OR критери* OR опросник* OR направлен* OR консульта* OR оцен* OR диагности* OR план* OR "план развития" OR мониторинг* OR "план ведения" OR "план* поддерж*" OR "управлени* риск*" OR развитие OR контрол* OR наблюд* OR оцен* OR измер* ) OR AB ( initiativ* OR interven* OR program* OR tilgang OR policy OR behandling OR model* OR strategi* OR metod* OR projekt OR instrument* OR tool* OR redskab OR framework* OR ramme OR protokol* OR analyse OR guide* OR vejledning OR skala OR system* OR indeks OR beholdning OR matrix OR format OR retningslin* OR template* OR skabelon OR profil* OR kriterie* OR spørgeskema OR refer* OR henvisning OR assess* OR vurde* OR måling OR screen* OR "case plan*" OR "case formulering" OR "management plan*" OR "treatment plan*" OR "behandlingsplan" OR "behandlingsforløb" OR "behandlingsprogram" OR "support plan*" OR "støtteforløb" OR "case manage*" OR "Sagsbehandling*" OR plan* OR "risikohåndtering*" OR "sikkerhedsvurdering" OR "sikkerhedsanalyse" OR progress OR fremskridt OR forbedringer OR monitor* OR supervis* OR måle* OR måling ) OR AB ( initiativ* OR intervensjon OR program* OR adgang OR policy OR behandle OR modell OR strategi* OR metod* OR prosjekt OR instrument OR tool* OR verktøy OR framework* OR rammeverk OR protokoll OR analyse OR guide* OR veiledning OR skala OR system* OR indeks OR inventar OR matrix OR metrisk OR format OR retningslinjer OR template* OR mal OR profil* OR kriterie* OR spørreskjema OR refer* OR referanse OR assess* OR vurdere* OR måling OR screen* OR "case plan*" OR "Saksplan*" OR "management plan*" OR "treatment plan*" OR "behandlingsplan*" OR "behandlingsforløp" OR "behandlingsprogram" OR "support plan*" OR "Støttekurs" OR "case manage*" OR "Saksbehandling*" OR "plan" OR "risikohåndtering*" OR "Sikkerhetsvurdering" OR "sikkerhetsanalyse" OR progress OR fremgang OR forbedringer OR monitor* OR tilsyn* OR måle* OR mål ) OR AB ( initiativ* OR intervensjon OR program* OR tillgång OR policy OR behandla* OR model* OR strateg* OR metod* OR projekt OR instrument* OR tool* OR verktyg OR framework* OR ram OR protokoll OR analyse OR guide* OR vägledning OR skala OR system* OR indeks OR inventering OR matrix OR metrisk OR format* OR riktlinj* OR template* OR mall OR profil* OR kriterie* OR frågeformulär OR refer* OR referens OR assess* OR bedöm* OR mätning OR screen* OR "case plan*" OR "Fallformulering" OR "management plan*" OR "treatment plan*" OR "behandlingsplan*" OR "behandlingsförlopp" OR "behandlingsprogram"OR "support plan*" OR "case manage*" OR "plan" OR "Planera" OR "Riskhantering*" OR "Säkerhetsbedömning" OR "Säkerhetsanalys" OR progress OR framsteg OR förbättringar OR monitor* OR övervaka* OR mäta* OR mått ) | Expanders - Apply equivalent subjects | Interface - EBSCOhost Research Databases | 366,561 |
|  |  | Search modes - Boolean/Phrase | Search Screen - Advanced Search |  |
|  |  |  | Database - Criminal Justice Abstracts |  |
| S6 | TI ( politique OR initiative* OR interven* OR progra* OR plan* OR traitement* OR approche* OR modèl* OR stratégi* OR méthod* OR projet* OR pratique* OR instrument* OR outil* OR cadre* OR protocole* OR guide* OR échelle* OR système* OR inventaire* OR métrique* gabarit* OR profile* OR critère* OR questionnaire* OR réfer* OR évalu* OR "plan d’intervention" OR "plan de traitement" OR "plan de gestion" OR "programme de gestion" OR "plan d’encadrement" OR "plan d’action" OR "programme de traitement" OR "plan de soins" OR "programme de soins" OR "projet de traitement" OR "plan thérapeutique" OR "programme thérapeutique" OR "plan de soutien" OR "plan d’accompagnement" OR "gestion de cas" OR "gestion de dossier" OR "gestion de risque*" OR "gestion des risques" OR progrès* OR évolution* OR progression* OR supervision* OR observation* OR surveill* OR suivi* OR mesure* ) OR TI ( initiative* OR interven* OR programm* OR Schema* OR behand* OR Ansatz* OR modell* OR strateg* OR Method* OR Projekt* OR praxis* OR instrument* OR Werkzeug* OR Rahmen* OR protokoll* OR anleit* OR Handlungsempfehlung* OR Ausmaß* OR Maßstab* OR system* OR Erfinder* OR metri* OR Vorlage* OR Profil* OR Kriteri* OR Umfrage* OR Einliefer* OR Einweis* OR Beurteil* OR Einschätz* OR "Fallplan*" OR "Fallgestaltung*" OR "Managementplan*" OR "Behandlungsplan*" OR "Unterstützungsplan*" OR "Förderplan*" OR "Fallmanagement*" OR "Fallbearbeitung*" OR "Risikomanagement*" OR "Risikobewältigung*" OR Forschritt* OR Entwicklung* OR beobacht* OR überwach* OR beaufsichtig* OR Aufsicht* OR Aufseher* OR Maßnahme* OR Messen* ) OR TI ( Инициатив* OR Вмешательств* OR программ* OR политик* OR схема* OR терап* OR подход* OR модель OR стратеги* метод* OR проект* OR практи* OR инструмент* OR концепци* OR структур* OR протокол* OR инструк* OR принцип* OR разработк* OR масштаб* OR системн* OR пример* OR обзор* OR критери* OR опросник* OR направлен* OR консульта* OR оцен* OR диагности* OR план* OR "план развития" OR мониторинг* OR "план ведения" OR "план* поддерж*" OR "управлени* риск*" OR развитие OR контрол* OR наблюд* OR оцен* OR измер* ) OR TI ( initiativ* OR interven* OR program* OR tilgang OR policy OR behandling OR model* OR strategi* OR metod* OR projekt OR instrument* OR tool* OR redskab OR framework* OR ramme OR protokol* OR analyse OR guide* OR vejledning OR skala OR system* OR indeks OR beholdning OR matrix OR format OR retningslin* OR template* OR skabelon OR profil* OR kriterie* OR spørgeskema OR refer* OR henvisning OR assess* OR vurde* OR måling OR screen* OR "case plan*" OR "case formulering" OR "management plan*" OR "treatment plan*" OR "behandlingsplan" OR "behandlingsforløb" OR "behandlingsprogram" OR "support plan*" OR "støtteforløb" OR "case manage*" OR "Sagsbehandling*" OR plan* OR "risikohåndtering*" OR "sikkerhedsvurdering" OR "sikkerhedsanalyse" OR progress OR fremskridt OR forbedringer OR monitor* OR supervis* OR måle* OR måling ) OR TI ( initiativ* OR intervensjon OR program* OR adgang OR policy OR behandle OR modell OR strategi* OR metod* OR prosjekt OR instrument OR tool* OR verktøy OR framework* OR rammeverk OR protokoll OR analyse OR guide* OR veiledning OR skala OR system* OR indeks OR inventar OR matrix OR metrisk OR format OR retningslinjer OR template* OR mal OR profil* OR kriterie* OR spørreskjema OR refer* OR referanse OR assess* OR vurdere* OR måling OR screen* OR "case plan*" OR "Saksplan*" OR "management plan*" OR "treatment plan*" OR "behandlingsplan*" OR "behandlingsforløp" OR "behandlingsprogram" OR "support plan*" OR "Støttekurs" OR "case manage*" OR "Saksbehandling*" OR "plan" OR "risikohåndtering*" OR "Sikkerhetsvurdering" OR "sikkerhetsanalyse" OR progress OR fremgang OR forbedringer OR monitor* OR tilsyn* OR måle* OR mål ) OR TI ( initiativ* OR intervensjon OR program* OR tillgång OR policy OR behandla* OR model* OR strateg* OR metod* OR projekt OR instrument* OR tool* OR verktyg OR framework* OR ram OR protokoll OR analyse OR guide* OR vägledning OR skala OR system* OR indeks OR inventering OR matrix OR metrisk OR format* OR riktlinj* OR template* OR mall OR profil* OR kriterie* OR frågeformulär OR refer* OR referens OR assess* OR bedöm* OR mätning OR screen* OR "case plan*" OR "Fallformulering" OR "management plan*" OR "treatment plan*" OR "behandlingsplan*" OR "behandlingsförlopp" OR "behandlingsprogram"OR "support plan*" OR "case manage*" OR "plan" OR "Planera" OR "Riskhantering*" OR "Säkerhetsbedömning" OR "Säkerhetsanalys" OR progress OR framsteg OR förbättringar OR monitor* OR övervaka* OR mäta* OR mått ) | Expanders - Apply equivalent subjects | Interface - EBSCOhost Research Databases | 104,764 |
|  |  | Search modes - Boolean/Phrase | Search Screen - Advanced Search |  |
|  |  |  | Database - Criminal Justice Abstracts |  |
| S5 | S1 OR S2 OR S3 OR S4 | Expanders - Apply equivalent subjects | Interface - EBSCOhost Research Databases | 81,419 |
|  |  | Search modes - Boolean/Phrase | Search Screen - Advanced Search |  |
|  |  |  | Database - Criminal Justice Abstracts |  |
| S4 | SU ( violen* OR agress* OR coerciti* OR contrain* OR batter* OR traque* OR harcel* OR viol* OR homicid* OR meurtr* OR tue* OR intimider OR "instiller la terreur" OR "violence domestique" OR "abus sexuel" OR "crime sexuel" OR gangs OR gang OR bandes OR bande OR rebelle OR rebelles OR insurgé* OR combattant* OR guérilla* OR militant* OR soldat* ) OR SU ( gewalt* OR angreif* OR Angriff* OR überfall* OR Anschlag* OR Körperverletzung* OR misshand* OR nötig*OR zwing* OR schlag* OR Schläg* OR stalk* OR nachstell* OR vergewaltig* OR mord* OR mörder* OR tödlich* OR totschl* OR töt* OR "persönlicher Terror*" OR "häusliche Gewalt*" OR "sexuelle Gewalt*" OR "sexueller Gewalt*" OR Sexualstraftäter* OR Sexualstraftat* OR gangs* OR Bande* OR rebel* OR aufständisch* OR aufrührer* OR kämpfer* OR Guerillakämpfer* OR Untergrundkämpfer* OR militant* OR soldat* ) OR SU ( насильствен* OR напад* OR бит* OR избиват* OR принужд* OR принудит* OR преследов* OR изнасилование OR убийств* OR убий* OR "домашнее насORе" OR "сексуальное насORе" OR "сексуальное преступ*" OR банды OR банда OR бунтарь OR бунтовщики OR повстанец OR повстанцы OR боец OR бойцы OR боевик OR боевики OR солдат* ) OR SU ( vold* OR angreb* OR overfald* OR slå OR tvang OR stalk* OR efterfølger OR rape OR voldtage OR voldtægt OR drab OR mord OR myrde OR morder OR dræbe OR "intim terror*" OR "misbrug i hjemmet" OR "vold i hjemmet" OR "overgreb i hjemmet" OR hustruvold OR "seksuelt misbrug" OR "seksuel vold" OR "seksuelle overgreb" OR "seksuel krænk*" OR seksualforbryd* OR gjeng OR rebel* OR oprør* OR oprører OR slagsbror OR fighter OR kombattant OR guerrilla* OR militant* OR soldat* ) OR SU ( vold* OR angrep* OR overfall* OR slå OR tvang OR stalk* OR etterfølger OR rape OR voldtekt OR voldta OR drap OR mord OR myrde OR morder OR drepe OR "intim terror*" OR "overgrep i hjemmet" OR "misbrug i hjemmet" OR "vold hjemmet" OR "konevold" OR "seksuell mishandling" OR "seksuell vold" OR "seksuelle overgrep" OR "seksuell krænk*" OR seksualforbryt* OR gäng OR rebel* OR opprør* OR opprører OR kampbror OR stridende OR stridende OR gerilja* OR militant* OR soldat* ) OR SU ( våld* OR "ge sig på*" OR överfall* OR slå OR tvång OR stalk* OR rape OR våldta OR våldtäkt OR raps OR dråp OR mord OR mörda OR mördare OR döda OR "intim terror*" OR "våld i hemmet" OR "misbrug i hemmat" OR "hustru våld" OR "sexuella övergrepp" OR "sexuellt våld" OR "sexuell krænk*" OR sexualförbryt OR bande OR rebel* OR uppror* OR rebell OR uprorsmann OR "strid bror" OR kämpe OR stridande OR kombatant OR gerilla* OR militant* OR soldat* ) | Search modes - Boolean/Phrase | Interface - EBSCOhost Research Databases | 44,228 |
|  |  |  | Search Screen - Advanced Search |  |
|  |  |  | Database - Criminal Justice Abstracts |  |
| S3 | KW ( violen* OR agress* OR coerciti* OR contrain* OR batter* OR traque* OR harcel* OR viol* OR homicid* OR meurtr* OR tue* OR intimider OR "instiller la terreur" OR "violence domestique" OR "abus sexuel" OR "crime sexuel" OR gangs OR gang OR bandes OR bande OR rebelle OR rebelles OR insurgé* OR combattant* OR guérilla* OR militant* OR soldat* ) OR KW ( gewalt* OR angreif* OR Angriff* OR überfall* OR Anschlag* OR Körperverletzung* OR misshand* OR nötig*OR zwing* OR schlag* OR Schläg* OR stalk* OR nachstell* OR vergewaltig* OR mord* OR mörder* OR tödlich* OR totschl* OR töt* OR "persönlicher Terror*" OR "häusliche Gewalt*" OR "sexuelle Gewalt*" OR "sexueller Gewalt*" OR Sexualstraftäter* OR Sexualstraftat* OR gangs* OR Bande* OR rebel* OR aufständisch* OR aufrührer* OR kämpfer* OR Guerillakämpfer* OR Untergrundkämpfer* OR militant* OR soldat* ) OR KW ( насильствен* OR напад* OR бит* OR избиват* OR принужд* OR принудит* OR преследов* OR изнасилование OR убийств* OR убий* OR "домашнее насORе" OR "сексуальное насORе" OR "сексуальное преступ*" OR банды OR банда OR бунтарь OR бунтовщики OR повстанец OR повстанцы OR боец OR бойцы OR боевик OR боевики OR солдат* ) OR KW ( vold* OR angreb* OR overfald* OR slå OR tvang OR stalk* OR efterfølger OR rape OR voldtage OR voldtægt OR drab OR mord OR myrde OR morder OR dræbe OR "intim terror*" OR "misbrug i hjemmet" OR "vold i hjemmet" OR "overgreb i hjemmet" OR hustruvold OR "seksuelt misbrug" OR "seksuel vold" OR "seksuelle overgreb" OR "seksuel krænk*" OR seksualforbryd* OR gjeng OR rebel* OR oprør* OR oprører OR slagsbror OR fighter OR kombattant OR guerrilla* OR militant* OR soldat* ) OR KW ( vold* OR angrep* OR overfall* OR slå OR tvang OR stalk* OR etterfølger OR rape OR voldtekt OR voldta OR drap OR mord OR myrde OR morder OR drepe OR "intim terror*" OR "overgrep i hjemmet" OR "misbrug i hjemmet" OR "vold hjemmet" OR "konevold" OR "seksuell mishandling" OR "seksuell vold" OR "seksuelle overgrep" OR "seksuell krænk*" OR seksualforbryt* OR gäng OR rebel* OR opprør* OR opprører OR kampbror OR stridende OR stridende OR gerilja* OR militant* OR soldat* ) OR KW ( våld* OR "ge sig på*" OR överfall* OR slå OR tvång OR stalk* OR rape OR våldta OR våldtäkt OR raps OR dråp OR mord OR mörda OR mördare OR döda OR "intim terror*" OR "våld i hemmet" OR "misbrug i hemmat" OR "hustru våld" OR "sexuella övergrepp" OR "sexuellt våld" OR "sexuell krænk*" OR sexualförbryt OR bande OR rebel* OR uppror* OR rebell OR uprorsmann OR "strid bror" OR kämpe OR stridande OR kombatant OR gerilla* OR militant* OR soldat* ) | Search modes - Boolean/Phrase | Interface - EBSCOhost Research Databases | 19,881 |
|  |  |  | Search Screen - Advanced Search |  |
|  |  |  | Database - Criminal Justice Abstracts |  |
| S2 | AB ( violen* OR agress* OR coerciti* OR contrain* OR batter* OR traque* OR harcel* OR viol* OR homicid* OR meurtr* OR tue* OR intimider OR "instiller la terreur" OR "violence domestique" OR "abus sexuel" OR "crime sexuel" OR gangs OR gang OR bandes OR bande OR rebelle OR rebelles OR insurgé* OR combattant* OR guérilla* OR militant* OR soldat* ) OR AB ( gewalt* OR angreif* OR Angriff* OR überfall* OR Anschlag* OR Körperverletzung* OR misshand* OR nötig*OR zwing* OR schlag* OR Schläg* OR stalk* OR nachstell* OR vergewaltig* OR mord* OR mörder* OR tödlich* OR totschl* OR töt* OR "persönlicher Terror*" OR "häusliche Gewalt*" OR "sexuelle Gewalt*" OR "sexueller Gewalt*" OR Sexualstraftäter* OR Sexualstraftat* OR gangs* OR Bande* OR rebel* OR aufständisch* OR aufrührer* OR kämpfer* OR Guerillakämpfer* OR Untergrundkämpfer* OR militant* OR soldat* ) OR AB ( насильствен* OR напад* OR бит* OR избиват* OR принужд* OR принудит* OR преследов* OR изнасилование OR убийств* OR убий* OR "домашнее насORе" OR "сексуальное насORе" OR "сексуальное преступ*" OR банды OR банда OR бунтарь OR бунтовщики OR повстанец OR повстанцы OR боец OR бойцы OR боевик OR боевики OR солдат* ) OR AB ( vold* OR angreb* OR overfald* OR slå OR tvang OR stalk* OR efterfølger OR rape OR voldtage OR voldtægt OR drab OR mord OR myrde OR morder OR dræbe OR "intim terror*" OR "misbrug i hjemmet" OR "vold i hjemmet" OR "overgreb i hjemmet" OR hustruvold OR "seksuelt misbrug" OR "seksuel vold" OR "seksuelle overgreb" OR "seksuel krænk*" OR seksualforbryd* OR gjeng OR rebel* OR oprør* OR oprører OR slagsbror OR fighter OR kombattant OR guerrilla* OR militant* OR soldat* ) OR AB ( vold* OR angrep* OR overfall* OR slå OR tvang OR stalk* OR etterfølger OR rape OR voldtekt OR voldta OR drap OR mord OR myrde OR morder OR drepe OR "intim terror*" OR "overgrep i hjemmet" OR "misbrug i hjemmet" OR "vold hjemmet" OR "konevold" OR "seksuell mishandling" OR "seksuell vold" OR "seksuelle overgrep" OR "seksuell krænk*" OR seksualforbryt* OR gäng OR rebel* OR opprør* OR opprører OR kampbror OR stridende OR stridende OR gerilja* OR militant* OR soldat* ) OR AB ( våld* OR "ge sig på*" OR överfall* OR slå OR tvång OR stalk* OR rape OR våldta OR våldtäkt OR raps OR dråp OR mord OR mörda OR mördare OR döda OR "intim terror*" OR "våld i hemmet" OR "misbrug i hemmat" OR "hustru våld" OR "sexuella övergrepp" OR "sexuellt våld" OR "sexuell krænk*" OR sexualförbryt OR bande OR rebel* OR uppror* OR rebell OR uprorsmann OR "strid bror" OR kämpe OR stridande OR kombatant OR gerilla* OR militant* OR soldat* ) | Search modes - Boolean/Phrase | Interface - EBSCOhost Research Databases | 68,862 |
|  |  |  | Search Screen - Advanced Search |  |
|  |  |  | Database - Criminal Justice Abstracts |  |
| S1 | TI ( violen* OR agress* OR coerciti* OR contrain* OR batter* OR traque* OR harcel* OR viol* OR homicid* OR meurtr* OR tue* OR intimider OR "instiller la terreur" OR "violence domestique" OR "abus sexuel" OR "crime sexuel" OR gangs OR gang OR bandes OR bande OR rebelle OR rebelles OR insurgé* OR combattant* OR guérilla* OR militant* OR soldat* ) OR TI ( gewalt* OR angreif* OR Angriff* OR überfall* OR Anschlag* OR Körperverletzung* OR misshand* OR nötig*OR zwing* OR schlag* OR Schläg* OR stalk* OR nachstell* OR vergewaltig* OR mord* OR mörder* OR tödlich* OR totschl* OR töt* OR "persönlicher Terror*" OR "häusliche Gewalt*" OR "sexuelle Gewalt*" OR "sexueller Gewalt*" OR Sexualstraftäter* OR Sexualstraftat* OR gangs* OR Bande* OR rebel* OR aufständisch* OR aufrührer* OR kämpfer* OR Guerillakämpfer* OR Untergrundkämpfer* OR militant* OR soldat* ) OR TI ( насильствен* OR напад* OR бит* OR избиват* OR принужд* OR принудит* OR преследов* OR изнасилование OR убийств* OR убий* OR "домашнее насORе" OR "сексуальное насORе" OR "сексуальное преступ*" OR банды OR банда OR бунтарь OR бунтовщики OR повстанец OR повстанцы OR боец OR бойцы OR боевик OR боевики OR солдат* ) OR TI ( vold* OR angreb* OR overfald* OR slå OR tvang OR stalk* OR efterfølger OR rape OR voldtage OR voldtægt OR drab OR mord OR myrde OR morder OR dræbe OR "intim terror*" OR "misbrug i hjemmet" OR "vold i hjemmet" OR "overgreb i hjemmet" OR hustruvold OR "seksuelt misbrug" OR "seksuel vold" OR "seksuelle overgreb" OR "seksuel krænk*" OR seksualforbryd* OR gjeng OR rebel* OR oprør* OR oprører OR slagsbror OR fighter OR kombattant OR guerrilla* OR militant* OR soldat* ) OR TI ( vold* OR angrep* OR overfall* OR slå OR tvang OR stalk* OR etterfølger OR rape OR voldtekt OR voldta OR drap OR mord OR myrde OR morder OR drepe OR "intim terror*" OR "overgrep i hjemmet" OR "misbrug i hjemmet" OR "vold hjemmet" OR "konevold" OR "seksuell mishandling" OR "seksuell vold" OR "seksuelle overgrep" OR "seksuell krænk*" OR seksualforbryt* OR gäng OR rebel* OR opprør* OR opprører OR kampbror OR stridende OR stridende OR gerilja* OR militant* OR soldat* ) OR TI ( våld* OR "ge sig på*" OR överfall* OR slå OR tvång OR stalk* OR rape OR våldta OR våldtäkt OR raps OR dråp OR mord OR mörda OR mördare OR döda OR "intim terror*" OR "våld i hemmet" OR "misbrug i hemmat" OR "hustru våld" OR "sexuella övergrepp" OR "sexuellt våld" OR "sexuell krænk*" OR sexualförbryt OR bande OR rebel* OR uppror* OR rebell OR uprorsmann OR "strid bror" OR kämpe OR stridande OR kombatant OR gerilla* OR militant* OR soldat* ) | Search modes - Boolean/Phrase | Interface - EBSCOhost Research Databases | 32,023 |
|  |  |  | Search Screen - Advanced Search |  |
|  |  |  | Database - Criminal Justice Abstracts |  |

**Table A1.3(d). Scopus Search Records – Part II (LOE)**

English Keywords

| **Query** | **Records** |
| --- | --- |
| ( TITLE-ABS-KEY ( violen* OR assault* OR "batter" OR batters OR battere* OR coerc* OR beat* OR stalk* OR rape OR homicid* OR murder* OR kill* OR "intimate terror*" OR "domestic abuse*" OR "sex* abuse*" OR "sex* offen*" OR gangs OR "gang" OR insurgent* OR rebel* OR fighter* OR combatant* OR guerrilla* OR militant* OR soldier* ) AND PUBYEAR > 1999 AND PUBYEAR < 2023 AND PUBYEAR > 1999 AND PUBYEAR < 2023 ) AND ( TITLE-ABS-KEY ( initiative* OR interven* OR program* OR policy OR policies OR scheme* OR treat* OR approach* OR model* OR strateg* OR method* OR project* OR practice* OR instrument* OR tool* OR framework* OR protocol* OR guid* OR scale* OR system* OR inventor* OR metric* OR template* OR profile* OR criteria OR questionnaire* OR refer* OR assess* OR "case plan*" OR "case formulat*" OR "management plan*" OR "treatment plan*" OR "support plan*" OR "case manage*" OR "risk manage*" OR progress* OR monitor* OR supervis* OR measur* ) AND PUBYEAR > 1999 AND PUBYEAR < 2023 AND PUBYEAR > 1999 AND PUBYEAR < 2023 ) AND ( TITLE-ABS-KEY ( prevent* OR reduc* OR counter* OR disengage* OR rehab* OR reintegrat* OR re-integrat* OR re-entry OR reentry OR desist* OR recidivism OR deradical* OR de-radical* OR exit* OR de-mobili* OR demobili* OR disarm* ) AND PUBYEAR > 1999 AND PUBYEAR < 2023 AND PUBYEAR > 1999 AND PUBYEAR < 2023 ) AND ( TITLE-ABS-KEY ( review* OR meta-analy* ) AND PUBYEAR > 1999 AND PUBYEAR < 2023 AND PUBYEAR > 1999 AND PUBYEAR < 2023 ) AND ( LIMIT-TO ( LANGUAGE , "german" ) OR LIMIT-TO ( LANGUAGE , "spanish" ) OR LIMIT-TO ( LANGUAGE , "french" ) OR LIMIT-TO ( LANGUAGE , "chinese" ) OR LIMIT-TO ( LANGUAGE , "portuguese" ) OR LIMIT-TO ( LANGUAGE , "italian" ) OR LIMIT-TO ( LANGUAGE , "russian" ) OR LIMIT-TO ( LANGUAGE , "japanese" ) OR LIMIT-TO ( LANGUAGE , "turkish" ) OR LIMIT-TO ( LANGUAGE , "polish" ) OR LIMIT-TO ( LANGUAGE , "persian" ) OR LIMIT-TO ( LANGUAGE , "croatian" ) OR LIMIT-TO ( LANGUAGE , "czech" ) OR LIMIT-TO ( LANGUAGE , "dutch" ) OR LIMIT-TO ( LANGUAGE , "hungarian" ) OR LIMIT-TO ( LANGUAGE , "hebrew" ) OR LIMIT-TO ( LANGUAGE , "danish" ) OR LIMIT-TO ( LANGUAGE , "greek" ) OR LIMIT-TO ( LANGUAGE , "slovenian" ) OR LIMIT-TO ( LANGUAGE , "korean" ) OR LIMIT-TO ( LANGUAGE , "serbian" ) OR LIMIT-TO ( LANGUAGE , "slovak" ) OR LIMIT-TO ( LANGUAGE , "swedish" ) OR LIMIT-TO ( LANGUAGE , "finnish" ) OR LIMIT-TO ( LANGUAGE , "norwegian" ) OR LIMIT-TO ( LANGUAGE , "romanian" ) OR LIMIT-TO ( LANGUAGE , "afrikaans" ) OR LIMIT-TO ( LANGUAGE , "bosnian" ) OR LIMIT-TO ( LANGUAGE , "bulgarian" ) OR LIMIT-TO ( LANGUAGE , "lithuanian" ) OR LIMIT-TO ( LANGUAGE , "moldavian" ) OR LIMIT-TO ( LANGUAGE , "moldovan" ) OR LIMIT-TO ( LANGUAGE , "latvian" ) OR LIMIT-TO ( LANGUAGE , "macedonian" ) OR LIMIT-TO ( LANGUAGE , "catalan" ) OR LIMIT-TO ( LANGUAGE , "undefined" ) ) | 1,820 results |
| ( TITLE-ABS-KEY ( violen* OR assault* OR "batter" OR batters OR battere* OR coerc* OR beat* OR stalk* OR rape OR homicid* OR murder* OR kill* OR "intimate terror*" OR "domestic abuse*" OR "sex* abuse*" OR "sex* offen*" OR gangs OR "gang" OR insurgent* OR rebel* OR fighter* OR combatant* OR guerrilla* OR militant* OR soldier* ) AND PUBYEAR > 1999 AND PUBYEAR < 2023 AND PUBYEAR > 1999 AND PUBYEAR < 2023 ) AND ( TITLE-ABS-KEY ( initiative* OR interven* OR program* OR policy OR policies OR scheme* OR treat* OR approach* OR model* OR strateg* OR method* OR project* OR practice* OR instrument* OR tool* OR framework* OR protocol* OR guid* OR scale* OR system* OR inventor* OR metric* OR template* OR profile* OR criteria OR questionnaire* OR refer* OR assess* OR "case plan*" OR "case formulat*" OR "management plan*" OR "treatment plan*" OR "support plan*" OR "case manage*" OR "risk manage*" OR progress* OR monitor* OR supervis* OR measur* ) AND PUBYEAR > 1999 AND PUBYEAR < 2023 AND PUBYEAR > 1999 AND PUBYEAR < 2023 ) AND ( TITLE-ABS-KEY ( prevent* OR reduc* OR counter* OR disengage* OR rehab* OR reintegrat* OR re-integrat* OR re-entry OR reentry OR desist* OR recidivism OR deradical* OR de-radical* OR exit* OR de-mobili* OR demobili* OR disarm* ) AND PUBYEAR > 1999 AND PUBYEAR < 2023 AND PUBYEAR > 1999 AND PUBYEAR < 2023 ) AND ( TITLE-ABS-KEY ( review* OR meta-analy* ) AND PUBYEAR > 1999 AND PUBYEAR < 2023 AND PUBYEAR > 1999 AND PUBYEAR < 2023 ) | 25,305 results |
| TITLE-ABS-KEY ( review* OR meta-analy* ) AND PUBYEAR > 1999 AND PUBYEAR < 2023 AND PUBYEAR > 1999 AND PUBYEAR < 2023 | 4,927,200 results |
| TITLE-ABS-KEY ( prevent* OR reduc* OR counter* OR disengage* OR rehab* OR reintegrat* OR re-integrat* OR re-entry OR reentry OR desist* OR recidivism OR deradical* OR de-radical* OR exit* OR de-mobili* OR demobili* OR disarm* ) AND PUBYEAR > 1999 AND PUBYEAR < 2023 AND PUBYEAR > 1999 AND PUBYEAR < 2023 | 10,623,744 results |
| TITLE-ABS-KEY ( initiative* OR interven* OR program* OR policy OR policies OR scheme* OR treat* OR approach* OR model* OR strateg* OR method* OR project* OR practice* OR instrument* OR tool* OR framework* OR protocol* OR guid* OR scale* OR system* OR inventor* OR metric* OR template* OR profile* OR criteria OR questionnaire* OR refer* OR assess* OR "case plan*" OR "case formulat*" OR "management plan*" OR "treatment plan*" OR "support plan*" OR "case manage*" OR "risk manage*" OR progress* OR monitor* OR supervis* OR measur* ) AND PUBYEAR > 1999 AND PUBYEAR < 2023 AND PUBYEAR > 1999 AND PUBYEAR < 2023 | 44,498,094 results |
| TITLE-ABS-KEY ( violen* OR assault* OR "batter" OR batters OR battere* OR coerc* OR beat* OR stalk* OR rape OR homicid* OR murder* OR kill* OR "intimate terror*" OR "domestic abuse*" OR "sex* abuse*" OR "sex* offen*" OR gangs OR "gang" OR insurgent* OR rebel* OR fighter* OR combatant* OR guerrilla* OR militant* OR soldier* ) AND PUBYEAR > 1999 AND PUBYEAR < 2023 AND PUBYEAR > 1999 AND PUBYEAR < 2023 | 867,433 results |

LOE Keywords

| **Query** | **Records** |
| --- | --- |
| ( ( TITLE-ABS-KEY ( violen* OR agress* OR coerciti* OR contrain* OR batter* OR traque* OR harcel* OR viol* OR homicid* OR meurtr* OR tue* OR intimider OR "instiller la terreur" OR "violence domestique" OR "abus sexuel" OR "crime sexuel" OR gangs OR gang OR bandes OR bande OR rebelle OR rebelles OR insurgé* OR combattant* OR guérilla* OR militant* OR soldat* ) OR TITLE-ABS-KEY ( gewalt* OR angreif* OR angriff* OR überfall* OR anschlag* OR körperverletzung* OR misshand* OR nötig*or AND zwing* OR schlag* OR schläg* OR stalk* OR nachstell* OR vergewaltig* OR mord* OR mörder* OR tödlich* OR totschl* OR töt* OR "persönlicher terror*" OR "häusliche gewalt*" OR "sexuelle gewalt*" OR "sexueller gewalt*" OR sexualstraftäter* OR sexualstraftat* OR gangs* OR bande* OR rebel* OR aufständisch* OR aufrührer* OR kämpfer* OR guerillakämpfer* OR untergrundkämpfer* OR militant* OR soldat* ) OR TITLE-ABS-KEY ( насильствен* OR напад* OR бит* OR избиват* OR принужд* OR принудит* OR преследов* OR изнасилование OR убийств* OR убий* OR "домашнее насorе" OR "сексуальное насorе" OR "сексуальное преступ*" OR банды OR банда OR бунтарь OR бунтовщики OR повстанец OR повстанцы OR боец OR бойцы OR боевик OR боевики OR солдат* ) OR TITLE-ABS-KEY ( vold* OR angreb* OR overfald* OR slå OR tvang OR stalk* OR efterfølger OR rape OR voldtage OR voldtægt OR drab OR mord OR myrde OR morder OR dræbe OR "intim terror*" OR "misbrug i hjemmet" OR "vold i hjemmet" OR "overgreb i hjemmet" OR hustruvold OR "seksuelt misbrug" OR "seksuel vold" OR "seksuelle overgreb" OR "seksuel krænk*" OR seksualforbryd* OR gjeng OR rebel* OR oprør* OR oprører OR slagsbror OR fighter OR kombattant OR guerrilla* OR militant* OR soldat* ) OR TITLE-ABS-KEY ( vold* OR angrep* OR overfall* OR slå OR tvang OR stalk* OR etterfølger OR rape OR voldtekt OR voldta OR drap OR mord OR myrde OR morder OR drepe OR "intim terror*" OR "overgrep i hjemmet" OR "misbrug i hjemmet" OR "vold hjemmet" OR "konevold" OR "seksuell mishandling" OR "seksuell vold" OR "seksuelle overgrep" OR "seksuell krænk*" OR seksualforbryt* OR gäng OR rebel* OR opprør* OR opprører OR kampbror OR stridende OR stridende OR gerilja* OR militant* OR soldat* ) OR TITLE-ABS-KEY ( våld* OR "ge sig på*" OR överfall* OR slå OR tvång OR stalk* OR rape OR våldta OR våldtäkt OR raps OR dråp OR mord OR mörda OR mördare OR döda OR "intim terror*" OR "våld i hemmet" OR "misbrug i hemmat" OR "hustru våld" OR "sexuella övergrepp" OR "sexuellt våld" OR "sexuell krænk*" OR sexualförbryt OR bande OR rebel* OR uppror* OR rebell OR uprorsmann OR "strid bror" OR kämpe OR stridande OR kombatant OR gerilla* OR militant* OR soldat* ) ) ) AND ( ( TITLE-ABS-KEY ( politique OR initiative* OR interven* OR progra* OR plan* OR traitement* OR approche* OR modèl* OR stratégi* OR méthod* OR projet* OR pratique* OR instrument* OR outil* OR cadre* OR protocole* OR guide* OR échelle* OR système* OR inventaire* OR métrique* AND gabarit* OR profile* OR critère* OR questionnaire* OR réfer* OR évalu* OR "plan d’intervention" OR "plan de traitement" OR "plan de gestion" OR "programme de gestion" OR "plan d’encadrement" OR "plan d’action" OR "programme de traitement" OR "plan de soins" OR "programme de soins" OR "projet de traitement" OR "plan thérapeutique" OR "programme thérapeutique" OR "plan de soutien" OR "plan d’accompagnement" OR "gestion de cas" OR "gestion de dossier" OR "gestion de risque*" OR "gestion des risques" OR progrès* OR évolution* OR progression* OR supervision* OR observation* OR surveill* OR suivi* OR mesure* ) OR TITLE-ABS-KEY ( initiative* OR interven* OR programm* OR schema* OR behand* OR ansatz* OR modell* OR strateg* OR method* OR projekt* OR praxis* OR instrument* OR werkzeug* OR rahmen* OR protokoll* OR anleit* OR handlungsempfehlung* OR ausmaß* OR maßstab* OR system* OR erfinder* OR metri* OR vorlage* OR profil* OR kriteri* OR umfrage* OR einliefer* OR einweis* OR beurteil* OR einschätz* OR "fallplan*" OR "fallgestaltung*" OR "managementplan*" OR "behandlungsplan*" OR "unterstützungsplan*" OR "förderplan*" OR "fallmanagement*" OR "fallbearbeitung*" OR "risikomanagement*" OR "risikobewältigung*" OR forschritt* OR entwicklung* OR beobacht* OR überwach* OR beaufsichtig* OR aufsicht* OR aufseher* OR maßnahme* OR messen* ) OR TITLE-ABS-KEY ( инициатив* OR вмешательств* OR программ* OR политик* OR схема* OR терап* OR подход* OR модель OR стратеги* AND метод* OR проект* OR практи* OR инструмент* OR концепци* OR структур* OR протокол* OR инструк* OR принцип* OR разработк* OR масштаб* OR системн* OR пример* OR обзор* OR критери* OR опросник* OR направлен* OR консульта* OR оцен* OR диагности* OR план* OR "план развития" OR мониторинг* OR "план ведения" OR "план* поддерж*" OR "управлени* риск*" OR развитие OR контрол* OR наблюд* OR оцен* OR измер* ) OR TITLE-ABS-KEY ( initiativ* OR interven* OR program* OR tilgang OR policy OR behandling OR model* OR strategi* OR metod* OR projekt OR instrument* OR tool* OR redskab OR framework* OR ramme OR protokol* OR analyse OR guide* OR vejledning OR skala OR system* OR indeks OR beholdning OR matrix OR format OR retningslin* OR template* OR skabelon OR profil* OR kriterie* OR spørgeskema OR refer* OR henvisning OR assess* OR vurde* OR måling OR screen* OR "case plan*" OR "case formulering" OR "management plan*" OR "treatment plan*" OR "behandlingsplan" OR "behandlingsforløb" OR "behandlingsprogram" OR "support plan*" OR "støtteforløb" OR "case manage*" OR "sagsbehandling*" OR plan* OR "risikohåndtering*" OR "sikkerhedsvurdering" OR "sikkerhedsanalyse" OR progress OR fremskridt OR forbedringer OR monitor* OR supervis* OR måle* OR måling ) OR TITLE-ABS-KEY ( initiativ* OR intervensjon OR program* OR adgang OR policy OR behandle OR modell OR strategi* OR metod* OR prosjekt OR instrument OR tool* OR verktøy OR framework* OR rammeverk OR protokoll OR analyse OR guide* OR veiledning OR skala OR system* OR indeks OR inventar OR matrix OR metrisk OR format OR retningslinjer OR template* OR mal OR profil* OR kriterie* OR spørreskjema OR refer* OR referanse OR assess* OR vurdere* OR måling OR screen* OR "case plan*" OR "saksplan*" OR "management plan*" OR "treatment plan*" OR "behandlingsplan*" OR "behandlingsforløp" OR "behandlingsprogram" OR "support plan*" OR "støttekurs" OR "case manage*" OR "saksbehandling*" OR "plan" OR "risikohåndtering*" OR "sikkerhetsvurdering" OR "sikkerhetsanalyse" OR progress OR fremgang OR forbedringer OR monitor* OR tilsyn* OR måle* OR mål ) OR TITLE-ABS-KEY ( initiativ* OR intervensjon OR program* OR tillgång OR policy OR behandla* OR model* OR strateg* OR metod* OR projekt OR instrument* OR tool* OR verktyg OR framework* OR ram OR protokoll OR analyse OR guide* OR vägledning OR skala OR system* OR indeks OR inventering OR matrix OR metrisk OR format* OR riktlinj* OR template* OR mall OR profil* OR kriterie* OR frågeformulär OR refer* OR referens OR assess* OR bedöm* OR mätning OR screen* OR "case plan*" OR "fallformulering" OR "management plan*" OR "treatment plan*" OR "behandlingsplan*" OR "behandlingsförlopp" OR "behandlingsprogram" OR "support plan*" OR "case manage*" OR "plan" OR "planera" OR "riskhantering*" OR "säkerhetsbedömning" OR "säkerhetsanalys" OR progress OR framsteg OR förbättringar OR monitor* OR övervaka* OR mäta* OR mått ) ) ) AND ( ( TITLE-ABS-KEY ( prévent* OR réduct* OR anti* OR contre* OR désengage* OR réhab* OR désendoctrin* OR désembriga* OR réinser* OR ré-inser* OR récidiv* OR réintégr* OR ré-intégr* OR renonc* OR désist* OR démobilis* OR désarm* ) OR TITLE-ABS-KEY ( verhinder* OR reduzier* OR minder* OR bekämpf* OR loslösen* OR rehab* OR resozialisier* OR reintegrat* OR wiedereinglieder* OR abstrandn* OR unterlass* OR rückfall* OR rückfäll* OR deradikal* OR aussteige* AND aussstieg* AND demobilisier* OR entwaffn* ) OR TITLE-ABS-KEY ( предупре* OR профилактик* OR предотвра* OR снижени* OR снизи* OR уменьш* OR противодейств* OR противостоя* OR реабorт* OR реинтегр* OR рецидив* OR демобor* OR разоруж* ) OR TITLE-ABS-KEY ( prevent* OR forebyg* OR reducere* OR formindske OR counter* OR bekæmp* OR disengage* OR afhop* OR rehab* OR reintegrat* OR gen-integrat* OR re-entry OR genindtræde OR return* OR desist* OR afstå* OR "tage afstand fra" OR recidivism OR tilbagfald OR exit* OR demobili* OR afvæbne ) OR TITLE-ABS-KEY ( prevent* OR forhindre* OR reducere* OR reduser* OR counter* OR kamp OR disengage* OR afhop* OR rehab* OR reintegrat* OR genintegrer* OR re-entry OR retur* OR desist* OR avstå* OR "avstand fra" OR recidivism OR tilbakefall OR exit* OR demobili* OR avvæpne ) OR TITLE-ABS-KEY ( prevent* OR förhindra* OR reducere* OR minska* OR counter* OR bekämpa OR disengage* OR afhop* OR rehab* OR reintegrat* OR återintegrera* OR re-entry OR återinträde OR returnera* OR desist* OR avstå* OR "avstånd från" OR recidivism OR deradikal AND * OR "falla tillbaka" OR exit* OR utgång* OR demobili* OR avväpna ) ) ) AND ( ( TITLE-ABS-KEY ( systématique OR méta-analyse* OR revue* OR recension ) OR TITLE-ABS-KEY ( meta-analyse* OR rezens* OR bewert* ) OR TITLE-ABS-KEY ( метаанали* OR мета-анали* OR обзор* ) OR TITLE-ABS-KEY ( meta-analytisk OR gennemgang ) OR TITLE-ABS-KEY ( meta-analyse OR anmeldelse ) OR TITLE-ABS-KEY ( meta-analys OR recension ) ) AND PUBYEAR > 1999 AND PUBYEAR < 2023 AND PUBYEAR > 1999 AND PUBYEAR < 2023 ) AND ( LIMIT-TO ( LANGUAGE , "french" ) OR LIMIT-TO ( LANGUAGE , "german" ) OR LIMIT-TO ( LANGUAGE , "russian" ) OR LIMIT-TO ( LANGUAGE , "spanish" ) OR LIMIT-TO ( LANGUAGE , "dutch" ) OR LIMIT-TO ( LANGUAGE , "italian" ) OR LIMIT-TO ( LANGUAGE , "croatian" ) OR LIMIT-TO ( LANGUAGE , "danish" ) OR LIMIT-TO ( LANGUAGE , "polish" ) OR LIMIT-TO ( LANGUAGE , "portuguese" ) ) | 173 results |
| ( ( TITLE-ABS-KEY ( violen* OR agress* OR coerciti* OR contrain* OR batter* OR traque* OR harcel* OR viol* OR homicid* OR meurtr* OR tue* OR intimider OR "instiller la terreur" OR "violence domestique" OR "abus sexuel" OR "crime sexuel" OR gangs OR gang OR bandes OR bande OR rebelle OR rebelles OR insurgé* OR combattant* OR guérilla* OR militant* OR soldat* ) OR TITLE-ABS-KEY ( gewalt* OR angreif* OR angriff* OR überfall* OR anschlag* OR körperverletzung* OR misshand* OR nötig*or AND zwing* OR schlag* OR schläg* OR stalk* OR nachstell* OR vergewaltig* OR mord* OR mörder* OR tödlich* OR totschl* OR töt* OR "persönlicher terror*" OR "häusliche gewalt*" OR "sexuelle gewalt*" OR "sexueller gewalt*" OR sexualstraftäter* OR sexualstraftat* OR gangs* OR bande* OR rebel* OR aufständisch* OR aufrührer* OR kämpfer* OR guerillakämpfer* OR untergrundkämpfer* OR militant* OR soldat* ) OR TITLE-ABS-KEY ( насильствен* OR напад* OR бит* OR избиват* OR принужд* OR принудит* OR преследов* OR изнасилование OR убийств* OR убий* OR "домашнее насorе" OR "сексуальное насorе" OR "сексуальное преступ*" OR банды OR банда OR бунтарь OR бунтовщики OR повстанец OR повстанцы OR боец OR бойцы OR боевик OR боевики OR солдат* ) OR TITLE-ABS-KEY ( vold* OR angreb* OR overfald* OR slå OR tvang OR stalk* OR efterfølger OR rape OR voldtage OR voldtægt OR drab OR mord OR myrde OR morder OR dræbe OR "intim terror*" OR "misbrug i hjemmet" OR "vold i hjemmet" OR "overgreb i hjemmet" OR hustruvold OR "seksuelt misbrug" OR "seksuel vold" OR "seksuelle overgreb" OR "seksuel krænk*" OR seksualforbryd* OR gjeng OR rebel* OR oprør* OR oprører OR slagsbror OR fighter OR kombattant OR guerrilla* OR militant* OR soldat* ) OR TITLE-ABS-KEY ( vold* OR angrep* OR overfall* OR slå OR tvang OR stalk* OR etterfølger OR rape OR voldtekt OR voldta OR drap OR mord OR myrde OR morder OR drepe OR "intim terror*" OR "overgrep i hjemmet" OR "misbrug i hjemmet" OR "vold hjemmet" OR "konevold" OR "seksuell mishandling" OR "seksuell vold" OR "seksuelle overgrep" OR "seksuell krænk*" OR seksualforbryt* OR gäng OR rebel* OR opprør* OR opprører OR kampbror OR stridende OR stridende OR gerilja* OR militant* OR soldat* ) OR TITLE-ABS-KEY ( våld* OR "ge sig på*" OR överfall* OR slå OR tvång OR stalk* OR rape OR våldta OR våldtäkt OR raps OR dråp OR mord OR mörda OR mördare OR döda OR "intim terror*" OR "våld i hemmet" OR "misbrug i hemmat" OR "hustru våld" OR "sexuella övergrepp" OR "sexuellt våld" OR "sexuell krænk*" OR sexualförbryt OR bande OR rebel* OR uppror* OR rebell OR uprorsmann OR "strid bror" OR kämpe OR stridande OR kombatant OR gerilla* OR militant* OR soldat* ) ) ) AND ( ( TITLE-ABS-KEY ( politique OR initiative* OR interven* OR progra* OR plan* OR traitement* OR approche* OR modèl* OR stratégi* OR méthod* OR projet* OR pratique* OR instrument* OR outil* OR cadre* OR protocole* OR guide* OR échelle* OR système* OR inventaire* OR métrique* AND gabarit* OR profile* OR critère* OR questionnaire* OR réfer* OR évalu* OR "plan d’intervention" OR "plan de traitement" OR "plan de gestion" OR "programme de gestion" OR "plan d’encadrement" OR "plan d’action" OR "programme de traitement" OR "plan de soins" OR "programme de soins" OR "projet de traitement" OR "plan thérapeutique" OR "programme thérapeutique" OR "plan de soutien" OR "plan d’accompagnement" OR "gestion de cas" OR "gestion de dossier" OR "gestion de risque*" OR "gestion des risques" OR progrès* OR évolution* OR progression* OR supervision* OR observation* OR surveill* OR suivi* OR mesure* ) OR TITLE-ABS-KEY ( initiative* OR interven* OR programm* OR schema* OR behand* OR ansatz* OR modell* OR strateg* OR method* OR projekt* OR praxis* OR instrument* OR werkzeug* OR rahmen* OR protokoll* OR anleit* OR handlungsempfehlung* OR ausmaß* OR maßstab* OR system* OR erfinder* OR metri* OR vorlage* OR profil* OR kriteri* OR umfrage* OR einliefer* OR einweis* OR beurteil* OR einschätz* OR "fallplan*" OR "fallgestaltung*" OR "managementplan*" OR "behandlungsplan*" OR "unterstützungsplan*" OR "förderplan*" OR "fallmanagement*" OR "fallbearbeitung*" OR "risikomanagement*" OR "risikobewältigung*" OR forschritt* OR entwicklung* OR beobacht* OR überwach* OR beaufsichtig* OR aufsicht* OR aufseher* OR maßnahme* OR messen* ) OR TITLE-ABS-KEY ( инициатив* OR вмешательств* OR программ* OR политик* OR схема* OR терап* OR подход* OR модель OR стратеги* AND метод* OR проект* OR практи* OR инструмент* OR концепци* OR структур* OR протокол* OR инструк* OR принцип* OR разработк* OR масштаб* OR системн* OR пример* OR обзор* OR критери* OR опросник* OR направлен* OR консульта* OR оцен* OR диагности* OR план* OR "план развития" OR мониторинг* OR "план ведения" OR "план* поддерж*" OR "управлени* риск*" OR развитие OR контрол* OR наблюд* OR оцен* OR измер* ) OR TITLE-ABS-KEY ( initiativ* OR interven* OR program* OR tilgang OR policy OR behandling OR model* OR strategi* OR metod* OR projekt OR instrument* OR tool* OR redskab OR framework* OR ramme OR protokol* OR analyse OR guide* OR vejledning OR skala OR system* OR indeks OR beholdning OR matrix OR format OR retningslin* OR template* OR skabelon OR profil* OR kriterie* OR spørgeskema OR refer* OR henvisning OR assess* OR vurde* OR måling OR screen* OR "case plan*" OR "case formulering" OR "management plan*" OR "treatment plan*" OR "behandlingsplan" OR "behandlingsforløb" OR "behandlingsprogram" OR "support plan*" OR "støtteforløb" OR "case manage*" OR "sagsbehandling*" OR plan* OR "risikohåndtering*" OR "sikkerhedsvurdering" OR "sikkerhedsanalyse" OR progress OR fremskridt OR forbedringer OR monitor* OR supervis* OR måle* OR måling ) OR TITLE-ABS-KEY ( initiativ* OR intervensjon OR program* OR adgang OR policy OR behandle OR modell OR strategi* OR metod* OR prosjekt OR instrument OR tool* OR verktøy OR framework* OR rammeverk OR protokoll OR analyse OR guide* OR veiledning OR skala OR system* OR indeks OR inventar OR matrix OR metrisk OR format OR retningslinjer OR template* OR mal OR profil* OR kriterie* OR spørreskjema OR refer* OR referanse OR assess* OR vurdere* OR måling OR screen* OR "case plan*" OR "saksplan*" OR "management plan*" OR "treatment plan*" OR "behandlingsplan*" OR "behandlingsforløp" OR "behandlingsprogram" OR "support plan*" OR "støttekurs" OR "case manage*" OR "saksbehandling*" OR "plan" OR "risikohåndtering*" OR "sikkerhetsvurdering" OR "sikkerhetsanalyse" OR progress OR fremgang OR forbedringer OR monitor* OR tilsyn* OR måle* OR mål ) OR TITLE-ABS-KEY ( initiativ* OR intervensjon OR program* OR tillgång OR policy OR behandla* OR model* OR strateg* OR metod* OR projekt OR instrument* OR tool* OR verktyg OR framework* OR ram OR protokoll OR analyse OR guide* OR vägledning OR skala OR system* OR indeks OR inventering OR matrix OR metrisk OR format* OR riktlinj* OR template* OR mall OR profil* OR kriterie* OR frågeformulär OR refer* OR referens OR assess* OR bedöm* OR mätning OR screen* OR "case plan*" OR "fallformulering" OR "management plan*" OR "treatment plan*" OR "behandlingsplan*" OR "behandlingsförlopp" OR "behandlingsprogram" OR "support plan*" OR "case manage*" OR "plan" OR "planera" OR "riskhantering*" OR "säkerhetsbedömning" OR "säkerhetsanalys" OR progress OR framsteg OR förbättringar OR monitor* OR övervaka* OR mäta* OR mått ) ) ) AND ( ( TITLE-ABS-KEY ( prévent* OR réduct* OR anti* OR contre* OR désengage* OR réhab* OR désendoctrin* OR désembriga* OR réinser* OR ré-inser* OR récidiv* OR réintégr* OR ré-intégr* OR renonc* OR désist* OR démobilis* OR désarm* ) OR TITLE-ABS-KEY ( verhinder* OR reduzier* OR minder* OR bekämpf* OR loslösen* OR rehab* OR resozialisier* OR reintegrat* OR wiedereinglieder* OR abstrandn* OR unterlass* OR rückfall* OR rückfäll* OR deradikal* OR aussteige* AND aussstieg* AND demobilisier* OR entwaffn* ) OR TITLE-ABS-KEY ( предупре* OR профилактик* OR предотвра* OR снижени* OR снизи* OR уменьш* OR противодейств* OR противостоя* OR реабorт* OR реинтегр* OR рецидив* OR демобor* OR разоруж* ) OR TITLE-ABS-KEY ( prevent* OR forebyg* OR reducere* OR formindske OR counter* OR bekæmp* OR disengage* OR afhop* OR rehab* OR reintegrat* OR gen-integrat* OR re-entry OR genindtræde OR return* OR desist* OR afstå* OR "tage afstand fra" OR recidivism OR tilbagfald OR exit* OR demobili* OR afvæbne ) OR TITLE-ABS-KEY ( prevent* OR forhindre* OR reducere* OR reduser* OR counter* OR kamp OR disengage* OR afhop* OR rehab* OR reintegrat* OR genintegrer* OR re-entry OR retur* OR desist* OR avstå* OR "avstand fra" OR recidivism OR tilbakefall OR exit* OR demobili* OR avvæpne ) OR TITLE-ABS-KEY ( prevent* OR förhindra* OR reducere* OR minska* OR counter* OR bekämpa OR disengage* OR afhop* OR rehab* OR reintegrat* OR återintegrera* OR re-entry OR återinträde OR returnera* OR desist* OR avstå* OR "avstånd från" OR recidivism OR deradikal AND * OR "falla tillbaka" OR exit* OR utgång* OR demobili* OR avväpna ) ) ) AND ( ( TITLE-ABS-KEY ( systématique OR méta-analyse* OR revue* OR recension ) OR TITLE-ABS-KEY ( meta-analyse* OR rezens* OR bewert* ) OR TITLE-ABS-KEY ( метаанали* OR мета-анали* OR обзор* ) OR TITLE-ABS-KEY ( meta-analytisk OR gennemgang ) OR TITLE-ABS-KEY ( meta-analyse OR anmeldelse ) OR TITLE-ABS-KEY ( meta-analys OR recension ) ) AND PUBYEAR > 1999 AND PUBYEAR < 2023 AND PUBYEAR > 1999 AND PUBYEAR < 2023 ) | 828 results |
| ( TITLE-ABS-KEY ( systématique OR méta-analyse* OR revue* OR recension ) OR TITLE-ABS-KEY ( meta-analyse* OR rezens* OR bewert* ) OR TITLE-ABS-KEY ( метаанали* OR мета-анали* OR обзор* ) OR TITLE-ABS-KEY ( meta-analytisk OR gennemgang ) OR TITLE-ABS-KEY ( meta-analyse OR anmeldelse ) OR TITLE-ABS-KEY ( meta-analys OR recension ) ) AND PUBYEAR > 1999 AND PUBYEAR < 2023 AND PUBYEAR > 1999 AND PUBYEAR < 2023 | 85,044 results |
| ( TITLE-ABS-KEY ( prévent* OR réduct* OR anti* OR contre* OR désengage* OR réhab* OR désendoctrin* OR désembriga* OR réinser* OR ré-inser* OR récidiv* OR réintégr* OR ré-intégr* OR renonc* OR désist* OR démobilis* OR désarm* ) OR TITLE-ABS-KEY ( verhinder* OR reduzier* OR minder* OR bekämpf* OR loslösen* OR rehab* OR resozialisier* OR reintegrat* OR wiedereinglieder* OR abstrandn* OR unterlass* OR rückfall* OR rückfäll* OR deradikal* OR aussteige* AND aussstieg* AND demobilisier* OR entwaffn* ) OR TITLE-ABS-KEY ( предупре* OR профилактик* OR предотвра* OR снижени* OR снизи* OR уменьш* OR противодейств* OR противостоя* OR реабorт* OR реинтегр* OR рецидив* OR демобor* OR разоруж* ) OR TITLE-ABS-KEY ( prevent* OR forebyg* OR reducere* OR formindske OR counter* OR bekæmp* OR disengage* OR afhop* OR rehab* OR reintegrat* OR gen-integrat* OR re-entry OR genindtræde OR return* OR desist* OR afstå* OR "tage afstand fra" OR recidivism OR tilbagfald OR exit* OR demobili* OR afvæbne ) OR TITLE-ABS-KEY ( prevent* OR forhindre* OR reducere* OR reduser* OR counter* OR kamp OR disengage* OR afhop* OR rehab* OR reintegrat* OR genintegrer* OR re-entry OR retur* OR desist* OR avstå* OR "avstand fra" OR recidivism OR tilbakefall OR exit* OR demobili* OR avvæpne ) OR TITLE-ABS-KEY ( prevent* OR förhindra* OR reducere* OR minska* OR counter* OR bekämpa OR disengage* OR afhop* OR rehab* OR reintegrat* OR återintegrera* OR re-entry OR återinträde OR returnera* OR desist* OR avstå* OR "avstånd från" OR recidivism OR deradikal AND * OR "falla tillbaka" OR exit* OR utgång* OR demobili* OR avväpna ) ) | 15,867,112 results |
| ( TITLE-ABS-KEY ( politique OR initiative* OR interven* OR progra* OR plan* OR traitement* OR approche* OR modèl* OR stratégi* OR méthod* OR projet* OR pratique* OR instrument* OR outil* OR cadre* OR protocole* OR guide* OR échelle* OR système* OR inventaire* OR métrique* AND gabarit* OR profile* OR critère* OR questionnaire* OR réfer* OR évalu* OR "plan d’intervention" OR "plan de traitement" OR "plan de gestion" OR "programme de gestion" OR "plan d’encadrement" OR "plan d’action" OR "programme de traitement" OR "plan de soins" OR "programme de soins" OR "projet de traitement" OR "plan thérapeutique" OR "programme thérapeutique" OR "plan de soutien" OR "plan d’accompagnement" OR "gestion de cas" OR "gestion de dossier" OR "gestion de risque*" OR "gestion des risques" OR progrès* OR évolution* OR progression* OR supervision* OR observation* OR surveill* OR suivi* OR mesure* ) OR TITLE-ABS-KEY ( initiative* OR interven* OR programm* OR schema* OR behand* OR ansatz* OR modell* OR strateg* OR method* OR projekt* OR praxis* OR instrument* OR werkzeug* OR rahmen* OR protokoll* OR anleit* OR handlungsempfehlung* OR ausmaß* OR maßstab* OR system* OR erfinder* OR metri* OR vorlage* OR profil* OR kriteri* OR umfrage* OR einliefer* OR einweis* OR beurteil* OR einschätz* OR "fallplan*" OR "fallgestaltung*" OR "managementplan*" OR "behandlungsplan*" OR "unterstützungsplan*" OR "förderplan*" OR "fallmanagement*" OR "fallbearbeitung*" OR "risikomanagement*" OR "risikobewältigung*" OR forschritt* OR entwicklung* OR beobacht* OR überwach* OR beaufsichtig* OR aufsicht* OR aufseher* OR maßnahme* OR messen* ) OR TITLE-ABS-KEY ( инициатив* OR вмешательств* OR программ* OR политик* OR схема* OR терап* OR подход* OR модель OR стратеги* AND метод* OR проект* OR практи* OR инструмент* OR концепци* OR структур* OR протокол* OR инструк* OR принцип* OR разработк* OR масштаб* OR системн* OR пример* OR обзор* OR критери* OR опросник* OR направлен* OR консульта* OR оцен* OR диагности* OR план* OR "план развития" OR мониторинг* OR "план ведения" OR "план* поддерж*" OR "управлени* риск*" OR развитие OR контрол* OR наблюд* OR оцен* OR измер* ) OR TITLE-ABS-KEY ( initiativ* OR interven* OR program* OR tilgang OR policy OR behandling OR model* OR strategi* OR metod* OR projekt OR instrument* OR tool* OR redskab OR framework* OR ramme OR protokol* OR analyse OR guide* OR vejledning OR skala OR system* OR indeks OR beholdning OR matrix OR format OR retningslin* OR template* OR skabelon OR profil* OR kriterie* OR spørgeskema OR refer* OR henvisning OR assess* OR vurde* OR måling OR screen* OR "case plan*" OR "case formulering" OR "management plan*" OR "treatment plan*" OR "behandlingsplan" OR "behandlingsforløb" OR "behandlingsprogram" OR "support plan*" OR "støtteforløb" OR "case manage*" OR "sagsbehandling*" OR plan* OR "risikohåndtering*" OR "sikkerhedsvurdering" OR "sikkerhedsanalyse" OR progress OR fremskridt OR forbedringer OR monitor* OR supervis* OR måle* OR måling ) OR TITLE-ABS-KEY ( initiativ* OR intervensjon OR program* OR adgang OR policy OR behandle OR modell OR strategi* OR metod* OR prosjekt OR instrument OR tool* OR verktøy OR framework* OR rammeverk OR protokoll OR analyse OR guide* OR veiledning OR skala OR system* OR indeks OR inventar OR matrix OR metrisk OR format OR retningslinjer OR template* OR mal OR profil* OR kriterie* OR spørreskjema OR refer* OR referanse OR assess* OR vurdere* OR måling OR screen* OR "case plan*" OR "saksplan*" OR "management plan*" OR "treatment plan*" OR "behandlingsplan*" OR "behandlingsforløp" OR "behandlingsprogram" OR "support plan*" OR "støttekurs" OR "case manage*" OR "saksbehandling*" OR "plan" OR "risikohåndtering*" OR "sikkerhetsvurdering" OR "sikkerhetsanalyse" OR progress OR fremgang OR forbedringer OR monitor* OR tilsyn* OR måle* OR mål ) OR TITLE-ABS-KEY ( initiativ* OR intervensjon OR program* OR tillgång OR policy OR behandla* OR model* OR strateg* OR metod* OR projekt OR instrument* OR tool* OR verktyg OR framework* OR ram OR protokoll OR analyse OR guide* OR vägledning OR skala OR system* OR indeks OR inventering OR matrix OR metrisk OR format* OR riktlinj* OR template* OR mall OR profil* OR kriterie* OR frågeformulär OR refer* OR referens OR assess* OR bedöm* OR mätning OR screen* OR "case plan*" OR "fallformulering" OR "management plan*" OR "treatment plan*" OR "behandlingsplan*" OR "behandlingsförlopp" OR "behandlingsprogram" OR "support plan*" OR "case manage*" OR "plan" OR "planera" OR "riskhantering*" OR "säkerhetsbedömning" OR "säkerhetsanalys" OR progress OR framsteg OR förbättringar OR monitor* OR övervaka* OR mäta* OR mått ) ) | 59,137,875 results |
| ( TITLE-ABS-KEY ( violen* OR agress* OR coerciti* OR contrain* OR batter* OR traque* OR harcel* OR viol* OR homicid* OR meurtr* OR tue* OR intimider OR "instiller la terreur" OR "violence domestique" OR "abus sexuel" OR "crime sexuel" OR gangs OR gang OR bandes OR bande OR rebelle OR rebelles OR insurgé* OR combattant* OR guérilla* OR militant* OR soldat* ) OR TITLE-ABS-KEY ( gewalt* OR angreif* OR angriff* OR überfall* OR anschlag* OR körperverletzung* OR misshand* OR nötig*or AND zwing* OR schlag* OR schläg* OR stalk* OR nachstell* OR vergewaltig* OR mord* OR mörder* OR tödlich* OR totschl* OR töt* OR "persönlicher terror*" OR "häusliche gewalt*" OR "sexuelle gewalt*" OR "sexueller gewalt*" OR sexualstraftäter* OR sexualstraftat* OR gangs* OR bande* OR rebel* OR aufständisch* OR aufrührer* OR kämpfer* OR guerillakämpfer* OR untergrundkämpfer* OR militant* OR soldat* ) OR TITLE-ABS-KEY ( насильствен* OR напад* OR бит* OR избиват* OR принужд* OR принудит* OR преследов* OR изнасилование OR убийств* OR убий* OR "домашнее насorе" OR "сексуальное насorе" OR "сексуальное преступ*" OR банды OR банда OR бунтарь OR бунтовщики OR повстанец OR повстанцы OR боец OR бойцы OR боевик OR боевики OR солдат* ) OR TITLE-ABS-KEY ( vold* OR angreb* OR overfald* OR slå OR tvang OR stalk* OR efterfølger OR rape OR voldtage OR voldtægt OR drab OR mord OR myrde OR morder OR dræbe OR "intim terror*" OR "misbrug i hjemmet" OR "vold i hjemmet" OR "overgreb i hjemmet" OR hustruvold OR "seksuelt misbrug" OR "seksuel vold" OR "seksuelle overgreb" OR "seksuel krænk*" OR seksualforbryd* OR gjeng OR rebel* OR oprør* OR oprører OR slagsbror OR fighter OR kombattant OR guerrilla* OR militant* OR soldat* ) OR TITLE-ABS-KEY ( vold* OR angrep* OR overfall* OR slå OR tvang OR stalk* OR etterfølger OR rape OR voldtekt OR voldta OR drap OR mord OR myrde OR morder OR drepe OR "intim terror*" OR "overgrep i hjemmet" OR "misbrug i hjemmet" OR "vold hjemmet" OR "konevold" OR "seksuell mishandling" OR "seksuell vold" OR "seksuelle overgrep" OR "seksuell krænk*" OR seksualforbryt* OR gäng OR rebel* OR opprør* OR opprører OR kampbror OR stridende OR stridende OR gerilja* OR militant* OR soldat* ) OR TITLE-ABS-KEY ( våld* OR "ge sig på*" OR överfall* OR slå OR tvång OR stalk* OR rape OR våldta OR våldtäkt OR raps OR dråp OR mord OR mörda OR mördare OR döda OR "intim terror*" OR "våld i hemmet" OR "misbrug i hemmat" OR "hustru våld" OR "sexuella övergrepp" OR "sexuellt våld" OR "sexuell krænk*" OR sexualförbryt OR bande OR rebel* OR uppror* OR rebell OR uprorsmann OR "strid bror" OR kämpe OR stridande OR kombatant OR gerilla* OR militant* OR soldat* ) ) | 1,304,954 results |

**Table A1.4(d). Medline Search Records (English Key Words Only) – Part II (LOE)**

| **Ovid MEDLINE(R) ALL <1946 to October 11, 2022>** | | |
| --- | --- | --- |
| 1 | (violen* or assault* or "batter" or batters or battere* or coerc* or beat* or stalk* or rape or homicid* or murder* or kill* or "intimate terror*" or "domestic abuse*" or "sex* abuse*" or "sex* offen*" or gangs or "gang" or insurgent* or rebel* or fighter* or combatant* or guerrilla* or militant* or soldier*).ab,hw,kf,ot,sh,sy,ti,fx. | 485202 |
| 2 | (initiative* or interven* or program* or policy or scheme* or treat* or approach* or model* or strateg* or method* or project* or practice* or instrument* or tool* or framework* or protocol* or guid* or scale* or system* or inventor* or metric* or template* or profile* or criteria or questionnaire* or refer* or assess* or "case plan*" or "case formulat*" or "management plan*" or "treatment plan*" or "support plan*" or "case manage*" or "risk manage*" or progress* or monitor* or supervis* or measur*).ab,hw,kf,ot,sh,sy,ti,fx. | 22674764 |
| 3 | (prevent* or reduc* or counter* or disengage* or rehab* or reintegrat* or re-integrat* or re-entry or reentry or desist* or recidivism or deradical* or de-radical* or exit* or de-mobili* or demobili* or disarm*).ab,hw,kf,ot,sh,sy,ti,fx. | 6716175 |
| 4 | (review* or meta-analy*).ab,hw,kf,ot,sh,sy,ti,fx. | 4334254 |
| 5 | 1 and 2 and 3 and 4 | 18066 |
| 6 | limit 5 to (humans and yr="2000 - 2022" and (afrikaans or albanian or arabic or armenian or azerbaijani or belorussian or bengali or bosnian or bulgarian or burmese or catalan or chinese or croatian or czech or danish or dutch or esperanto or estonian or finnish or flemish or french or gaelic, scots or georgian or german or greek or hausa or hebrew or hindi or hungarian or icelandic or indonesian or interlingua or italian or japanese or kirghiz or korean or latin or latvian or lithuanian or macedonian or malay or marathi or masai or multilingual or norwegian or persian or polish or portuguese or pushto or rumanian or russian or serbian or slovak or slovene or spanish or swahili or swedish or tagalog or tamil or telugu or thai or turkish or ukrainian or undetermined or urdu or vietnamese or welsh)) | 883 |

**Table A1.5(d). PyscInfo Search Records (English Key Words Only) – Part II (LOE)**

| **APA PsycInfo <1806 to October Week 1 2022>** | | |
| --- | --- | --- |
| 1 | (violen* or assault* or "batter" or batters or battere* or coerc* or beat* or stalk* or rape or homicid* or murder* or kill* or "intimate terror*" or "domestic abuse*" or "sex* abuse*" or "sex* offen*" or gangs or "gang" or insurgent* or rebel* or fighter* or combatant* or guerrilla* or militant* or soldier*).ab,hw,id,mh,ot,sh,ti. | 204444 |
| 2 | (initiative* or interven* or program* or policy or scheme* or treat* or approach* or model* or strateg* or method* or project* or practice* or instrument* or tool* or framework* or protocol* or guid* or scale* or system* or inventor* or metric* or template* or profile* or criteria or questionnaire* or refer* or assess* or "case plan*" or "case formulat*" or "management plan*" or "treatment plan*" or "support plan*" or "case manage*" or "risk manage*" or progress* or monitor* or supervis* or measur*).ab,hw,id,mh,ot,sh,ti. | 4174424 |
| 3 | (prevent* or reduc* or counter* or disengage* or rehab* or reintegrat* or re-integrat* or re-entry or reentry or desist* or recidivism or deradical* or de-radical* or exit* or de-mobili* or demobili* or disarm*).ab,hw,id,mh,ot,sh,ti. | 864581 |
| 4 | (review* or meta-analy*).ab,hw,id,mh,ot,sh,ti. | 611429 |
| 5 | 1 and 2 and 3 and 4 | 7233 |
| 6 | limit 5 to (human and yr="2000 - 2022") | 5922 |
| 7 | limit 6 to (afrikaans or albanian or arabic or bulgarian or catalan or chinese or czech or danish or dutch or finnish or french or georgian or german or greek or hebrew or hindi or hungarian or iranian or italian or japanese or korean or lithuanian or malaysian or nonenglish or norwegian or polish or portuguese or romanian or russian or serbo croatian or slovak or slovene or spanish or swedish or turkish or ukrainian) | 244 |

**Table A1.6(d). Dissertations and Theses Global (English Key Words Only) – Part II (LOE)**

| **Set#** | **Searched for** | **Databases** | **Results** |
| --- | --- | --- | --- |
| S1 | ti(violen* OR assault* OR "batter" OR batters OR battere* OR coerc* OR beat* OR stalk* OR rape OR homicid* OR murder* OR kill* OR "intimate terror*" OR "domestic abuse*" OR "sex* abuse*" OR "sex* offen*" OR gangs OR "gang" OR insurgent* OR rebel* OR fighter* OR combatant* OR guerrilla* OR militant* OR soldier*) OR ab(violen* OR assault* OR "batter" OR batters OR battere* OR coerc* OR beat* OR stalk* OR rape OR homicid* OR murder* OR kill* OR "intimate terror*" OR "domestic abuse*" OR "sex* abuse*" OR "sex* offen*" OR gangs OR "gang" OR insurgent* OR rebel* OR fighter* OR combatant* OR guerrilla* OR militant* OR soldier*) OR mainsubject(violen* OR assault* OR "batter" OR batters OR battere* OR coerc* OR beat* OR stalk* OR rape OR homicid* OR murder* OR kill* OR "intimate terror*" OR "domestic abuse*" OR "sex* abuse*" OR "sex* offen*" OR gangs OR "gang" OR insurgent* OR rebel* OR fighter* OR combatant* OR guerrilla* OR militant* OR soldier*) OR diskw(violen* OR assault* OR "batter" OR batters OR battere* OR coerc* OR beat* OR stalk* OR rape OR homicid* OR murder* OR kill* OR "intimate terror*" OR "domestic abuse*" OR "sex* abuse*" OR "sex* offen*" OR gangs OR "gang" OR insurgent* OR rebel* OR fighter* OR combatant* OR guerrilla* OR militant* OR soldier*) | ProQuest Dissertations & Theses Global | 134241 |
| S2 | ti(initiative* OR interven* OR program* OR policy OR policies OR scheme* OR treat* OR approach* OR model* OR strateg* OR method* OR project* OR practice* OR instrument* OR tool* OR framework* OR protocol* OR guid* OR scale* OR system* OR inventor* OR metric* OR template* OR profile* OR criteria OR questionnaire* OR refer* OR assess* OR "case plan*" OR "case formulat*" OR "management plan*" OR "treatment plan*" OR "support plan*" OR "case manage*" OR "risk manage*" OR progress* OR monitor* OR supervis* OR measur*) OR ab(initiative* OR interven* OR program* OR policy OR policies OR scheme* OR treat* OR approach* OR model* OR strateg* OR method* OR project* OR practice* OR instrument* OR tool* OR framework* OR protocol* OR guid* OR scale* OR system* OR inventor* OR metric* OR template* OR profile* OR criteria OR questionnaire* OR refer* OR assess* OR "case plan*" OR "case formulat*" OR "management plan*" OR "treatment plan*" OR "support plan*" OR "case manage*" OR "risk manage*" OR progress* OR monitor* OR supervis* OR measur*) OR mainsubject(initiative* OR interven* OR program* OR policy OR policies OR scheme* OR treat* OR approach* OR model* OR strateg* OR method* OR project* OR practice* OR instrument* OR tool* OR framework* OR protocol* OR guid* OR scale* OR system* OR inventor* OR metric* OR template* OR profile* OR criteria OR questionnaire* OR refer* OR assess* OR "case plan*" OR "case formulat*" OR "management plan*" OR "treatment plan*" OR "support plan*" OR "case manage*" OR "risk manage*" OR progress* OR monitor* OR supervis* OR measur*) OR diskw(initiative* OR interven* OR program* OR policy OR policies OR scheme* OR treat* OR approach* OR model* OR strateg* OR method* OR project* OR practice* OR instrument* OR tool* OR framework* OR protocol* OR guid* OR scale* OR system* OR inventor* OR metric* OR template* OR profile* OR criteria OR questionnaire* OR refer* OR assess* OR "case plan*" OR "case formulat*" OR "management plan*" OR "treatment plan*" OR "support plan*" OR "case manage*" OR "risk manage*" OR progress* OR monitor* OR supervis* OR measur*) | ProQuest Dissertations & Theses Global | 3817910 |
| S3 | ti(prevent* OR reduc* OR counter* OR disengage* OR rehab* OR reintegrat* OR re-integrat* OR re-entry OR reentry OR desist* OR recidivism OR deradical* OR de-radical* OR exit* OR de-mobili* OR demobili* OR disarm*) OR ab(prevent* OR reduc* OR counter* OR disengage* OR rehab* OR reintegrat* OR re-integrat* OR re-entry OR reentry OR desist* OR recidivism OR deradical* OR de-radical* OR exit* OR de-mobili* OR demobili* OR disarm*) OR mainsubject(prevent* OR reduc* OR counter* OR disengage* OR rehab* OR reintegrat* OR re-integrat* OR re-entry OR reentry OR desist* OR recidivism OR deradical* OR de-radical* OR exit* OR de-mobili* OR demobili* OR disarm*) OR diskw(prevent* OR reduc* OR counter* OR disengage* OR rehab* OR reintegrat* OR re-integrat* OR re-entry OR reentry OR desist* OR recidivism OR deradical* OR de-radical* OR exit* OR de-mobili* OR demobili* OR disarm*) | ProQuest Dissertations & Theses Global | 860735 |
| S4 | ti(review* OR meta-analy*) OR ab(review* OR meta-analy*) OR mainsubject(review* OR meta-analy*) OR diskw(review* OR meta-analy*) | ProQuest Dissertations & Theses Global | 268864 |
| S5 | S1 AND S2 AND S3 AND S4 | ProQuest Dissertations & Theses Global | 2275 |
| S6 | (S1 AND S2 AND S3 AND S4) AND pd(20000101-20221231) | ProQuest Dissertations & Theses Global | 1878 |
| S7 | (S1 AND S2 AND S3 AND S4) AND (la.exact("CHI" OR "POR" OR "SPA" OR "HEB" OR "FRE" OR "TUR" OR "AFR" OR "DUT" OR "GER" OR "GRE" OR "SRP" OR "SWE") AND pd(20000101-20221231)) | ProQuest Dissertations & Theses Global | 191 |

**Table A1.7(d). International Bibliography of the Social Sciences (English Key Words Only) – Part II (LOE)**

| **Set#** | **Searched for** | **Databases** | **Results** |
| --- | --- | --- | --- |
| S9 | ti(violen* OR assault* OR "batter" OR batters OR battere* OR coerc* OR beat* OR stalk* OR rape OR homicid* OR murder* OR kill* OR "intimate terror*" OR "domestic abuse*" OR "sex* abuse*" OR "sex* offen*" OR gangs OR "gang" OR insurgent* OR rebel* OR fighter* OR combatant* OR guerrilla* OR militant* OR soldier*) OR ab(violen* OR assault* OR "batter" OR batters OR battere* OR coerc* OR beat* OR stalk* OR rape OR homicid* OR murder* OR kill* OR "intimate terror*" OR "domestic abuse*" OR "sex* abuse*" OR "sex* offen*" OR gangs OR "gang" OR insurgent* OR rebel* OR fighter* OR combatant* OR guerrilla* OR militant* OR soldier*) OR mainsubject(violen* OR assault* OR "batter" OR batters OR battere* OR coerc* OR beat* OR stalk* OR rape OR homicid* OR murder* OR kill* OR "intimate terror*" OR "domestic abuse*" OR "sex* abuse*" OR "sex* offen*" OR gangs OR "gang" OR insurgent* OR rebel* OR fighter* OR combatant* OR guerrilla* OR militant* OR soldier*) | International Bibliography of the Social Sciences (IBSS) | 188621 |
| S10 | ti(initiative* OR interven* OR program* OR policy OR policies OR scheme* OR treat* OR approach* OR model* OR strateg* OR method* OR project* OR practice* OR instrument* OR tool* OR framework* OR protocol* OR guid* OR scale* OR system* OR inventor* OR metric* OR template* OR profile* OR criteria OR questionnaire* OR refer* OR assess* OR "case plan*" OR "case formulat*" OR "management plan*" OR "treatment plan*" OR "support plan*" OR "case manage*" OR "risk manage*" OR progress* OR monitor* OR supervis* OR measur*) OR ab(initiative* OR interven* OR program* OR policy OR policies OR scheme* OR treat* OR approach* OR model* OR strateg* OR method* OR project* OR practice* OR instrument* OR tool* OR framework* OR protocol* OR guid* OR scale* OR system* OR inventor* OR metric* OR template* OR profile* OR criteria OR questionnaire* OR refer* OR assess* OR "case plan*" OR "case formulat*" OR "management plan*" OR "treatment plan*" OR "support plan*" OR "case manage*" OR "risk manage*" OR progress* OR monitor* OR supervis* OR measur*) OR mainsubject(initiative* OR interven* OR program* OR policy OR policies OR scheme* OR treat* OR approach* OR model* OR strateg* OR method* OR project* OR practice* OR instrument* OR tool* OR framework* OR protocol* OR guid* OR scale* OR system* OR inventor* OR metric* OR template* OR profile* OR criteria OR questionnaire* OR refer* OR assess* OR "case plan*" OR "case formulat*" OR "management plan*" OR "treatment plan*" OR "support plan*" OR "case manage*" OR "risk manage*" OR progress* OR monitor* OR supervis* OR measur*) | International Bibliography of the Social Sciences (IBSS) | 2725402 |
| S11 | ti(prevent* OR reduc* OR counter* OR disengage* OR rehab* OR reintegrat* OR re-integrat* OR re-entry OR reentry OR desist* OR recidivism OR deradical* OR de-radical* OR exit* OR de-mobili* OR demobili* OR disarm*) OR ab(prevent* OR reduc* OR counter* OR disengage* OR rehab* OR reintegrat* OR re-integrat* OR re-entry OR reentry OR desist* OR recidivism OR deradical* OR de-radical* OR exit* OR de-mobili* OR demobili* OR disarm*) OR mainsubject(prevent* OR reduc* OR counter* OR disengage* OR rehab* OR reintegrat* OR re-integrat* OR re-entry OR reentry OR desist* OR recidivism OR deradical* OR de-radical* OR exit* OR de-mobili* OR demobili* OR disarm*) | International Bibliography of the Social Sciences (IBSS) | 340499 |
| S12 | ti(review* OR meta-analy*) OR ab(review* OR meta-analy*) OR mainsubject(review* OR meta-analy*) | International Bibliography of the Social Sciences (IBSS) | 600750 |
| S13 | S9 AND S10 AND S11 AND S12 | International Bibliography of the Social Sciences (IBSS) | 1833 |
| S14 | (S9 AND S10 AND S11 AND S12) AND pd(20000101-20221231) | International Bibliography of the Social Sciences (IBSS) | 1727 |
| S15 | (S9 AND S10 AND S11 AND S12) NOT (at.exact("General Information" OR "Editorial" OR "News" OR "Correspondence" OR "Credit/Acknowledgement" OR "Letter to the Editor") AND pd(20000101-20221231)) | International Bibliography of the Social Sciences (IBSS) | 1682 |
| S16 | (S9 AND S10 AND S11 AND S12) NOT (at.exact("General Information" OR "Editorial" OR "News" OR "Correspondence" OR "Credit/Acknowledgement" OR "Letter to the Editor") AND la.exact("SPA" OR "FRE" OR "GER" OR "POR" OR "POL" OR "RUM" OR "RUS" OR "AFR" OR "HEB" OR "HUN" OR "ITA" OR "SLA" OR "TUR" OR "UKR" OR "URD") AND pd(20000101-20221231)) | International Bibliography of the Social Sciences (IBSS) | 80 |

**Table A1.8(d). Sociological Abstracts (English Key Words Only) – Part II (LOE)**

| **Set#** | **Searched for** | **Databases** | **Results** |
| --- | --- | --- | --- |
| S1 | ti(violen* OR assault* OR "batter" OR batters OR battere* OR coerc* OR beat* OR stalk* OR rape OR homicid* OR murder* OR kill* OR "intimate terror*" OR "domestic abuse*" OR "sex* abuse*" OR "sex* offen*" OR gangs OR "gang" OR insurgent* OR rebel* OR fighter* OR combatant* OR guerrilla* OR militant* OR soldier*) OR ab(violen* OR assault* OR "batter" OR batters OR battere* OR coerc* OR beat* OR stalk* OR rape OR homicid* OR murder* OR kill* OR "intimate terror*" OR "domestic abuse*" OR "sex* abuse*" OR "sex* offen*" OR gangs OR "gang" OR insurgent* OR rebel* OR fighter* OR combatant* OR guerrilla* OR militant* OR soldier*) OR mainsubject(violen* OR assault* OR "batter" OR batters OR battere* OR coerc* OR beat* OR stalk* OR rape OR homicid* OR murder* OR kill* OR "intimate terror*" OR "domestic abuse*" OR "sex* abuse*" OR "sex* offen*" OR gangs OR "gang" OR insurgent* OR rebel* OR fighter* OR combatant* OR guerrilla* OR militant* OR soldier*) | Sociological Abstracts | 172979 |
| S2 | ti(initiative* OR interven* OR program* OR policy OR policies OR scheme* OR treat* OR approach* OR model* OR strateg* OR method* OR project* OR practice* OR instrument* OR tool* OR framework* OR protocol* OR guid* OR scale* OR system* OR inventor* OR metric* OR template* OR profile* OR criteria OR questionnaire* OR refer* OR assess* OR "case plan*" OR "case formulat*" OR "management plan*" OR "treatment plan*" OR "support plan*" OR "case manage*" OR "risk manage*" OR progress* OR monitor* OR supervis* OR measur*) OR ab(initiative* OR interven* OR program* OR policy OR policies OR scheme* OR treat* OR approach* OR model* OR strateg* OR method* OR project* OR practice* OR instrument* OR tool* OR framework* OR protocol* OR guid* OR scale* OR system* OR inventor* OR metric* OR template* OR profile* OR criteria OR questionnaire* OR refer* OR assess* OR "case plan*" OR "case formulat*" OR "management plan*" OR "treatment plan*" OR "support plan*" OR "case manage*" OR "risk manage*" OR progress* OR monitor* OR supervis* OR measur*) OR mainsubject(initiative* OR interven* OR program* OR policy OR policies OR scheme* OR treat* OR approach* OR model* OR strateg* OR method* OR project* OR practice* OR instrument* OR tool* OR framework* OR protocol* OR guid* OR scale* OR system* OR inventor* OR metric* OR template* OR profile* OR criteria OR questionnaire* OR refer* OR assess* OR "case plan*" OR "case formulat*" OR "management plan*" OR "treatment plan*" OR "support plan*" OR "case manage*" OR "risk manage*" OR progress* OR monitor* OR supervis* OR measur*) | Sociological Abstracts | 1555030 |
| S3 | ti(prevent* OR reduc* OR counter* OR disengage* OR rehab* OR reintegrat* OR re-integrat* OR re-entry OR reentry OR desist* OR recidivism OR deradical* OR de-radical* OR exit* OR de-mobili* OR demobili* OR disarm*) OR ab(prevent* OR reduc* OR counter* OR disengage* OR rehab* OR reintegrat* OR re-integrat* OR re-entry OR reentry OR desist* OR recidivism OR deradical* OR de-radical* OR exit* OR de-mobili* OR demobili* OR disarm*) OR mainsubject(prevent* OR reduc* OR counter* OR disengage* OR rehab* OR reintegrat* OR re-integrat* OR re-entry OR reentry OR desist* OR recidivism OR deradical* OR de-radical* OR exit* OR de-mobili* OR demobili* OR disarm*) | Sociological Abstracts | 231322 |
| S4 | ti(review* OR meta-analy*) OR ab(review* OR meta-analy*) OR mainsubject(review* OR meta-analy*) | Sociological Abstracts | 323943 |
| S5 | S1 AND S2 AND S3 AND S4 | Sociological Abstracts | 2768 |
| S6 | (S1 AND S2 AND S3 AND S4) AND pd(20000101-20221231) | Sociological Abstracts | 2163 |
| S7 | (S1 AND S2 AND S3 AND S4) NOT (at.exact("General Information" OR "Editorial" OR "Correspondence" OR "Letter to the Editor" OR "News") AND pd(20000101-20221231)) | Sociological Abstracts | 2152 |
| S8 | (S1 AND S2 AND S3 AND S4) NOT (at.exact("General Information" OR "Editorial" OR "Correspondence" OR "Letter to the Editor" OR "News") AND la.exact("SPA" OR "POR" OR "FRE" OR "GER" OR "SLA" OR "DAN" OR "HRV" OR "ITA" OR "RUS" OR "SRP" OR "TUR") AND pd(20000101-20221231)) | Sociological Abstracts | 61 |

**Table A1.9(d). Web of Science – Part II (LOE)**

English Key Words

| **Search string** | **Editions** | **Timespan** | **Results** |
| --- | --- | --- | --- |
| 7: #4 AND #3 AND #2 AND #1 and Book Review or Editorial Material or Letter (Exclude – Document Types) and Spanish or German or Portuguese or French or Russian or Italian or Turkish or Chinese or Croatian or Greek or Polish or Korean or Slovak or Arabic or Czech or Estonian or Danish or Afrikaans or Dutch or Icelandic or Indonesian or Japanese or Slovenian or Unspecified (Languages) | A&HCI , BKCI-SSH , ESCI , CPCI-SSH , SSCI | 9:27 AM | Exact search | 544 |
| 6: #4 AND #3 AND #2 AND #1 and Book Review or Editorial Material or Letter (Exclude – Document Types) | A&HCI , BKCI-SSH , ESCI , CPCI-SSH , SSCI | 9:26 AM | Exact search | 8101 |
| 5: #4 AND #3 AND #2 AND #1 | A&HCI , BKCI-SSH , ESCI , CPCI-SSH , SSCI | 9:24 AM | Exact search | 8197 |
| 4: TS=(review* OR meta-analy*) | A&HCI , BKCI-SSH , ESCI , CPCI-SSH , SSCI | 9:24 AM | Timespan: 2000-01-01 to 2022-12-31 (Publication Date) | Exact search | 872360 |
| 3: TS=(prevent* OR reduc* OR counter* OR disengage* OR rehab* OR reintegrat* OR re-integrat* OR re-entry OR reentry OR desist* OR recidivism OR deradical* OR de-radical* OR exit* OR de-mobili* OR demobili* OR disarm*) | A&HCI , BKCI-SSH , ESCI , CPCI-SSH , SSCI | 9:24 AM | Timespan: 2000-01-01 to 2022-12-31 (Publication Date) | Exact search | 1645301 |
| 2: TS=(initiative* OR interven* OR program* OR policy OR scheme* OR treat* OR approach* OR model* OR strateg* OR method* OR project* OR practice* OR instrument* OR tool* OR framework* OR protocol* OR guid* OR scale* OR system* OR inventor* OR metric* OR template* OR profile* OR criteria OR questionnaire* OR refer* OR assess* OR "case plan*" OR "case formulat*" OR "management plan*" OR "treatment plan*" OR "support plan*" OR "case manage*" OR "risk manage*" OR progress* OR monitor* OR supervis* OR measur*) | A&HCI , BKCI-SSH , ESCI , CPCI-SSH , SSCI | 9:24 AM | Timespan: 2000-01-01 to 2022-12-31 (Publication Date) | Exact search | 7971696 |
| 1: TS=(violen* OR assault* OR "batter" OR batters OR battere* OR coerc* OR beat* OR stalk* OR rape OR homicid* OR murder* OR kill* OR "intimate terror*" OR "domestic abuse*" OR "sex* abuse*" OR "sex* offen*" OR gangs OR "gang" OR insurgent* OR rebel* OR fighter* OR combatant* OR guerrilla* OR militant* OR soldier*) | A&HCI , BKCI-SSH , ESCI , CPCI-SSH , SSCI | 9:23 AM | Timespan: 2000-01-01 to 2022-12-31 (Publication Date) | Exact search | 347637 |

LOE Key Words

| **Search string** | **Editions** | **Timespan** | **Results** |
| --- | --- | --- | --- |
| 1: TS=(violen* OR agress* OR coerciti* OR contrain* OR batter* OR traque* OR harcel* OR viol* OR homicid* OR meurtr* OR tue* OR intimider OR "instiller la terreur" OR "violence domestique" OR "abus sexuel" OR "crime sexuel" OR gangs OR gang OR bandes OR bande OR rebelle OR rebelles OR insurgé* OR combattant* OR guérilla* OR militant* OR soldat* ) | Editions: WOS.SSCI,WOS.AHCI,WOS.ISSHP,WOS.BHCI,WOS.ESCI | Timespan: 2000-01-01 to 2022-12-31 | Results: 316322 |
| 2: TS=(gewalt* OR angreif* OR Angriff* OR überfall* OR Anschlag* OR Körperverletzung* OR misshand* OR nötig*OR zwing* OR schlag* OR Schläg* OR stalk* OR nachstell* OR vergewaltig* OR mord* OR mörder* OR tödlich* OR totschl* OR töt* OR "persönlicher Terror*" OR "häusliche Gewalt*" OR "sexuelle Gewalt*" OR "sexueller Gewalt*" OR Sexualstraftäter* OR Sexualstraftat* OR gangs* OR Bande* OR rebel* OR aufständisch* OR aufrührer* OR kämpfer* OR Guerillakämpfer* OR Untergrundkämpfer* OR militant* OR soldat* ) | Editions: WOS.SSCI,WOS.AHCI,WOS.ISSHP,WOS.BHCI,WOS.ESCI | Timespan: 2000-01-01 to 2022-12-31 | Results: 690942 |
| 3: TS=(насильствен* OR напад* OR бит* OR избиват* OR принужд* OR принудит* OR преследов* OR изнасилование OR убийств* OR убий* OR "домашнее насORе" OR "сексуальное насORе" OR "сексуальное преступ*" OR банды OR банда OR бунтарь OR бунтовщики OR повстанец OR повстанцы OR боец OR бойцы OR боевик OR боевики OR солдат*) | Editions: WOS.SSCI,WOS.AHCI,WOS.ISSHP,WOS.BHCI,WOS.ESCI | Timespan: 2000-01-01 to 2022-12-31 | Results: 0 |
| 4: TS=(vold* OR angreb* OR overfald* OR slå OR tvang OR stalk* OR efterfølger OR rape OR voldtage OR voldtægt OR drab OR mord OR myrde OR morder OR dræbe OR "intim terror*" OR "misbrug i hjemmet" OR "vold i hjemmet" OR "overgreb i hjemmet" OR hustruvold OR "seksuelt misbrug" OR "seksuel vold" OR "seksuelle overgreb" OR "seksuel krænk*" OR seksualforbryd* OR gjeng OR rebel* OR oprør* OR oprører OR slagsbror OR fighter OR kombattant OR guerrilla* OR militant* OR soldat*) | Editions: WOS.SSCI,WOS.AHCI,WOS.ISSHP,WOS.BHCI,WOS.ESCI | Timespan: 2000-01-01 to 2022-12-31 | Results: 44194 |
| 5: TS=(vold* OR angrep* OR overfall* OR slå OR tvang OR stalk* OR etterfølger OR rape OR voldtekt OR voldta OR drap OR mord OR myrde OR morder OR drepe OR "intim terror*" OR "overgrep i hjemmet" OR "misbrug i hjemmet" OR "vold hjemmet" OR "konevold" OR "seksuell mishandling" OR "seksuell vold" OR "seksuelle overgrep" OR "seksuell krænk*" OR seksualforbryt* OR gäng OR rebel* OR opprør* OR opprører OR kampbror OR stridende OR stridende OR gerilja* OR militant* OR soldat*) | Editions: WOS.SSCI,WOS.AHCI,WOS.ISSHP,WOS.BHCI,WOS.ESCI | Timespan: 2000-01-01 to 2022-12-31 | Results: 43880 |
| 6: TS=(våld* OR "ge sig på*" OR överfall* OR slå OR tvång OR stalk* OR rape OR våldta OR våldtäkt OR raps OR dråp OR mord OR mörda OR mördare OR döda OR "intim terror*" OR "våld i hemmet" OR "misbrug i hemmat" OR "hustru våld" OR "sexuella övergrepp" OR "sexuellt våld" OR "sexuell krænk*" OR sexualförbryt OR bande OR rebel* OR uppror* OR rebell OR uprorsmann OR "strid bror" OR kämpe OR stridande OR kombatant OR gerilla* OR militant* OR soldat*) | Editions: WOS.SSCI,WOS.AHCI,WOS.ISSHP,WOS.BHCI,WOS.ESCI | Timespan: 2000-01-01 to 2022-12-31 | Results: 42663 |
| 7: #6 OR #5 OR #4 OR #3 OR #2 OR #1 | Editions: WOS.SSCI,WOS.AHCI,WOS.ISSHP,WOS.BHCI,WOS.ESCI | Timespan: 2000-01-01 to 2022-12-31 | Results: 991644 |
| 8: TS=(politique OR initiative* OR interven* OR progra* OR plan* OR traitement* OR approche* OR modèl* OR stratégi* OR méthod* OR projet* OR pratique* OR instrument* OR outil* OR cadre* OR protocole* OR guide* OR échelle* OR système* OR inventaire* OR métrique* gabarit* OR profile* OR critère* OR questionnaire* OR réfer* OR évalu* OR "plan d’intervention" OR "plan de traitement" OR "plan de gestion" OR "programme de gestion" OR "plan d’encadrement" OR "plan d’action" OR "programme de traitement" OR "plan de soins" OR "programme de soins" OR "projet de traitement" OR "plan thérapeutique" OR "programme thérapeutique" OR "plan de soutien" OR "plan d’accompagnement" OR "gestion de cas" OR "gestion de dossier" OR "gestion de risque*" OR "gestion des risques" OR progrès* OR évolution* OR progression* OR supervision* OR observation* OR surveill* OR suivi* OR mesure*) | Editions: WOS.SSCI,WOS.AHCI,WOS.ISSHP,WOS.BHCI,WOS.ESCI | Timespan: 2000-01-01 to 2022-12-31 | Results: 6337509 |
| 9: TS=(initiative* OR interven* OR programm* OR Schema* OR behand* OR Ansatz* OR modell* OR strateg* OR Method* OR Projekt* OR praxis* OR instrument* OR Werkzeug* OR Rahmen* OR protokoll* OR anleit* OR Handlungsempfehlung* OR Ausmaß* OR Maßstab* OR system* OR Erfinder* OR metri* OR Vorlage* OR Profil* OR Kriteri* OR Umfrage* OR Einliefer* OR Einweis* OR Beurteil* OR Einschätz* OR "Fallplan*" OR "Fallgestaltung*" OR "Managementplan*" OR "Behandlungsplan*" OR "Unterstützungsplan*" OR "Förderplan*" OR "Fallmanagement*" OR "Fallbearbeitung*" OR "Risikomanagement*" OR "Risikobewältigung*" OR Forschritt* OR Entwicklung* OR beobacht* OR überwach* OR beaufsichtig* OR Aufsicht* OR Aufseher* OR Maßnahme* OR Messen*) | Editions: WOS.SSCI,WOS.AHCI,WOS.ISSHP,WOS.BHCI,WOS.ESCI | Timespan: 2000-01-01 to 2022-12-31 | Results: 5070459 |
| 10: TS=(Инициатив* OR Вмешательств* OR программ* OR политик* OR схема* OR терап* OR подход* OR модель OR стратеги* метод* OR проект* OR практи* OR инструмент* OR концепци* OR структур* OR протокол* OR инструк* OR принцип* OR разработк* OR масштаб* OR системн* OR пример* OR обзор* OR критери* OR опросник* OR направлен* OR консульта* OR оцен* OR диагности* OR план* OR "план развития" OR мониторинг* OR "план ведения" OR "план* поддерж*" OR "управлени* риск*" OR развитие OR контрол* OR наблюд* OR оцен* OR измер*) | Editions: WOS.SSCI,WOS.AHCI,WOS.ISSHP,WOS.BHCI,WOS.ESCI | Timespan: 2000-01-01 to 2022-12-31 | Results: 0 |
| 11: TS=(initiativ* OR interven* OR program* OR tilgang OR policy OR behandling OR model* OR strategi* OR metod* OR projekt OR instrument* OR tool* OR redskab OR framework* OR ramme OR protokol* OR analyse OR guide* OR vejledning OR skala OR system* OR indeks OR beholdning OR matrix OR format OR retningslin* OR template* OR skabelon OR profil* OR kriterie* OR spørgeskema OR refer* OR henvisning OR assess* OR vurde* OR måling OR screen* OR "case plan*" OR "case formulering" OR "management plan*" OR "treatment plan*" OR "behandlingsplan" OR "behandlingsforløb" OR "behandlingsprogram" OR "support plan*" OR "støtteforløb" OR "case manage*" OR "Sagsbehandling*" OR plan* OR "risikohåndtering*" OR "sikkerhedsvurdering" OR "sikkerhedsanalyse" OR progress OR fremskridt OR forbedringer OR monitor* OR supervis* OR måle* OR måling ) | Editions: WOS.SSCI,WOS.AHCI,WOS.ISSHP,WOS.BHCI,WOS.ESCI | Timespan: 2000-01-01 to 2022-12-31 | Results: 6618761 |
| 12: TS=(initiativ* OR intervensjon OR program* OR adgang OR policy OR behandle OR modell OR strategi* OR metod* OR prosjekt OR instrument OR tool* OR verktøy OR framework* OR rammeverk OR protokoll OR analyse OR guide* OR veiledning OR skala OR system* OR indeks OR inventar OR matrix OR metrisk OR format OR retningslinjer OR template* OR mal OR profil* OR kriterie* OR spørreskjema OR refer* OR referanse OR assess* OR vurdere* OR måling OR screen* OR "case plan*" OR "Saksplan*" OR "management plan*" OR "treatment plan*" OR "behandlingsplan*" OR "behandlingsforløp" OR "behandlingsprogram" OR "support plan*" OR "Støttekurs" OR "case manage*" OR "Saksbehandling*" OR "plan" OR "risikohåndtering*" OR "Sikkerhetsvurdering" OR "sikkerhetsanalyse" OR progress OR fremgang OR forbedringer OR monitor* OR tilsyn* OR måle* OR mål) | Editions: WOS.SSCI,WOS.AHCI,WOS.ISSHP,WOS.BHCI,WOS.ESCI | Timespan: 2000-01-01 to 2022-12-31 | Results: 5587106 |
| 13: TS=(initiativ* OR intervensjon OR program* OR tillgång OR policy OR behandla* OR model* OR strateg* OR metod* OR projekt OR instrument* OR tool* OR verktyg OR framework* OR ram OR protokoll OR analyse OR guide* OR vägledning OR skala OR system* OR indeks OR inventering OR matrix OR metrisk OR format* OR riktlinj* OR template* OR mall OR profil* OR kriterie* OR frågeformulär OR refer* OR referens OR assess* OR bedöm* OR mätning OR screen* OR "case plan*" OR "Fallformulering" OR "management plan*" OR "treatment plan*" OR "behandlingsplan*" OR "behandlingsförlopp" OR "behandlingsprogram"OR "support plan*" OR "case manage*" OR "plan" OR "Planera" OR "Riskhantering*" OR "Säkerhetsbedömning" OR "Säkerhetsanalys" OR progress OR framsteg OR förbättringar OR monitor* OR övervaka* OR mäta* OR mått ) | Editions: WOS.SSCI,WOS.AHCI,WOS.ISSHP,WOS.BHCI,WOS.ESCI | Timespan: 2000-01-01 to 2022-12-31 | Results: 6333562 |
| 14: #8 OR #9 OR #10 OR #11 OR #12 OR #13 | Editions: WOS.SSCI,WOS.AHCI,WOS.ISSHP,WOS.BHCI,WOS.ESCI | Timespan: 2000-01-01 to 2022-12-31 | Results: 7602786 |
| 15: TS=(prévent* OR réduct* OR anti* OR contre* OR désengage* OR réhab* OR désendoctrin* OR désembriga* OR réinser* OR ré-inser* OR récidiv* OR réintégr* OR ré-intégr* OR renonc* OR désist* OR démobilis* OR désarm*) | Editions: WOS.SSCI,WOS.AHCI,WOS.ISSHP,WOS.BHCI,WOS.ESCI | Timespan: 2000-01-01 to 2022-12-31 | Results: 1369443 |
| 16: TS=(verhinder* OR reduzier* OR minder* OR bekämpf* OR loslösen* OR rehab* OR resozialisier* OR reintegrat* OR wiedereinglieder* OR Abstrandn* OR unterlass* OR rückfall* OR Rückfäll* OR deradikal* OR aussteige* Aussstieg* demobilisier* OR entwaffn*) | Editions: WOS.SSCI,WOS.AHCI,WOS.ISSHP,WOS.BHCI,WOS.ESCI | Timespan: 2000-01-01 to 2022-12-31 | Results: 112417 |
| 17: TS=(предупре* OR профилактик* OR предотвра* OR снижени* OR снизи* OR уменьш* OR противодейств* OR противостоя* OR реабORт* OR реинтегр* OR рецидив* OR демобOR* OR разоруж*) | Editions: WOS.SSCI,WOS.AHCI,WOS.ISSHP,WOS.BHCI,WOS.ESCI | Timespan: 2000-01-01 to 2022-12-31 | Results: 0 |
| 18: TS=(prevent* OR forebyg* OR reducere* OR formindske OR counter* OR bekæmp* OR disengage* OR afhop* OR rehab* OR reintegrat* OR gen-integrat* OR re-entry OR genindtræde OR return* OR desist* OR afstå* OR "tage afstand fra" OR recidivism OR tilbagfald OR exit* OR demobili* OR afvæbne) | Editions: WOS.SSCI,WOS.AHCI,WOS.ISSHP,WOS.BHCI,WOS.ESCI | Timespan: 2000-01-01 to 2022-12-31 | Results: 1008447 |
| 19: TS=(prevent* OR forhindre* OR reducere* OR reduser* OR counter* OR kamp OR disengage* OR afhop* OR rehab* OR reintegrat* OR genintegrer* OR re-entry OR retur* OR desist* OR avstå* OR "avstand fra" OR recidivism OR tilbakefall OR exit* OR demobili* OR avvæpne) | Editions: WOS.SSCI,WOS.AHCI,WOS.ISSHP,WOS.BHCI,WOS.ESCI | Timespan: 2000-01-01 to 2022-12-31 | Results: 1008602 |
| 20: TS=(prevent* OR förhindra* OR reducere* OR minska* OR counter* OR bekämpa OR disengage* OR afhop* OR rehab* OR reintegrat* OR återintegrera* OR re-entry OR återinträde OR returnera* OR desist* OR avstå* OR "avstånd från" OR recidivism OR deradikal * OR "falla tillbaka" OR exit* OR utgång* OR demobili* OR avväpna) | Editions: WOS.SSCI,WOS.AHCI,WOS.ISSHP,WOS.BHCI,WOS.ESCI | Timespan: 2000-01-01 to 2022-12-31 | Results: 823279 |
| 21: #20 OR #19 OR #18 OR #17 OR #16 OR #15 | Editions: WOS.SSCI,WOS.AHCI,WOS.ISSHP,WOS.BHCI,WOS.ESCI | Timespan: 2000-01-01 to 2022-12-31 | Results: 1727935 |
| 22: TS=(systématique OR méta-analyse* OR revue* OR recension) | Editions: WOS.SSCI,WOS.AHCI,WOS.ISSHP,WOS.BHCI,WOS.ESCI | Timespan: 2000-01-01 to 2022-12-31 | Results: 20900 |
| 23: TS=(meta-Analyse* OR Rezens* OR Bewert*) | Editions: WOS.SSCI,WOS.AHCI,WOS.ISSHP,WOS.BHCI,WOS.ESCI | Timespan: 2000-01-01 to 2022-12-31 | Results: 19622 |
| 24: TS=(метаанали* OR мета-анали* OR обзор*) | Editions: WOS.SSCI,WOS.AHCI,WOS.ISSHP,WOS.BHCI,WOS.ESCI | Timespan: 2000-01-01 to 2022-12-31 | Results: 0 |
| 25: TS=(meta-analytisk OR gennemgang) | Editions: WOS.SSCI,WOS.AHCI,WOS.ISSHP,WOS.BHCI,WOS.ESCI | Timespan: 2000-01-01 to 2022-12-31 | Results: 0 |
| 26: TS=(meta-analyse OR anmeldelse) | Editions: WOS.SSCI,WOS.AHCI,WOS.ISSHP,WOS.BHCI,WOS.ESCI | Timespan: 2000-01-01 to 2022-12-31 | Results: 139 |
| 27: TS=(meta-analys OR recension) | Editions: WOS.SSCI,WOS.AHCI,WOS.ISSHP,WOS.BHCI,WOS.ESCI | Timespan: 2000-01-01 to 2022-12-31 | Results: 239 |
| 28: #27 OR #26 OR #25 OR #24 OR #23 OR #22 | Editions: WOS.SSCI,WOS.AHCI,WOS.ISSHP,WOS.BHCI,WOS.ESCI | Timespan: 2000-01-01 to 2022-12-31 | Results: 21034 |
| 29: #28 AND #21 AND #14 AND #7 | Editions: WOS.SSCI,WOS.AHCI,WOS.ISSHP,WOS.BHCI,WOS.ESCI | Timespan: 2000-01-01 to 2022-12-31 | Results: 1703 |
| 30: #28 AND #21 AND #14 AND #7 and Editorial Material (Exclude – Document Types) | Editions: WOS.SSCI,WOS.AHCI,WOS.ISSHP,WOS.BHCI,WOS.ESCI | Timespan: 2000-01-01 to 2022-12-31 | Results: 1699 |
| 31: #28 AND #21 AND #14 AND #7 and Editorial Material (Exclude – Document Types) and German or Italian or Spanish or French or Portuguese or Chinese or Dutch or Greek or Korean or Polish or Russian or Turkish (Languages) | Editions: WOS.SSCI,WOS.AHCI,WOS.ISSHP,WOS.BHCI,WOS.ESCI | Timespan: 2000-01-01 to 2022-12-31 | Results: 54 |

**Appendix II. Screening Tool**

Part I: Countering radicalisation to violence

| **Topic** | **Inclusion criteria Research Question 1** | **Inclusion criteria Research Question 2** | **Exclusion criteria** |
| --- | --- | --- | --- |
| Duplicate | N/A | | Duplicate title, author and year to be excluded. |
| Research design | Quantitative study using an experimental research design to report on primary or secondary research data.  OR  Quantitative study using a stronger quasi-experimental research design to report on primary or secondary research data. | Quantitative study using an experimental, quasi-experimental or non-experimental research design to report on primary or secondary data.  OR  Qualitative study using any research design to report on primary or secondary data. | Study does not meet inclusion criteria for either Q1 or Q2. |
| Comparator condition | Treatment as usual OR Alternative treatment OR  No treatment | N/A | Question 1  Study does not use an eligible comparator condition.  No exclusion criteria for Question 2. |
| Individual focus | Intervention delivered to individual or tool that is individually-focused. | | Intervention delivered to collective or group. |
| Case management intervention, tool or approach. | **Intervention:** Examination of case management intervention or a stage in the case management process  OR  **Tool:** Examination of tool(s) used within case management process.  OR  **Approach:** Examination of approach(es) underpinning the case management process. | | Absence of case management intervention, tool or approach. |
| Radicalisation to violence | Intervention seeking to tackle cognitive or behavioural radicalisation/ deliver relevant primary or secondary outcomes:  **Primary:** preventing engagement in violent extremism or promoting disengagement from / preventing re-engagement in violent extremism.  **Secondary:** reduction in risk factors seen to contribute to radicalisation/ strengthening of strengths and/ or protective factors seen to contribute to resilience. | | Not explicitly focused on tackling cognitive or behavioural radicalisation |
| Effectiveness/ Implementation | Study examines the effectiveness of an intervention, tool or approach in delivering intervention outcomes | Study examines the process of implementing an intervention, tool or approach. | No discussion of either element. |

Part II: Countering other forms of violence

| **Topic** | **Inclusion criteria Research Question 3** | **Inclusion criteria Research Question 4** | | **Inclusion criteria Research Question 5** | | **Exclusion criteria** |
| --- | --- | --- | --- | --- | --- | --- |
| Duplicate | N/A | | | | | Duplicate title, author and year to be excluded. |
| Research design | Systematic reviews or meta-analyses | | | | | Not a systematic review or a meta-analysis |
| Individual focus | Intervention delivered to individual or tool that is individually-focused. | | | | | Intervention delivered to collective or group. |
| Case management intervention, tool or approach. | **Intervention:** Examination of case management intervention or stage in the case management process  OR  **Tool:** Examination of tool(s) used within case management process.  OR  **Approach:** Examination of approach(es) underpinning the case management process. | | | | | No discussion of case management intervention, tool or approach. |
| Violence | Intervention seeking to tackle individual involvement in violent behaviour of any type (e.g. domestic violence, gang-related violence, sexual violence)/ deliver relevant primary or secondary outcomes:  **Primary:** preventing individuals’ engagement in violence or promoting disengagement from violence/ preventing violent recidivism.  **Secondary:** reduction in risk factors seen to contribute violent behaviour/ strengthening of protective factors seen to contribute to resilience. | | | | | Interventions that do not specifically target violence. |
| Effectiveness/ Implementation | Study examines effectiveness of an intervention, tool or approach in delivering intervention outcomes | | Study examines the process of implementing intervention, tool or approach | | Study examines effectiveness of intervention, tool or approach in delivering intervention outcomes  OR  Study examines the process of implementing intervention, tool or approach. | No discussion of either effectiveness/ implementation. |
| Methods  (Full-text screening only) | The methodology used meets the following criteria:  - Authors must specify clear inclusion and exclusion criteria, and offer some justification.  - Authors must use explicit search strategy, specifying:  The different strategies used to identify research (e.g., keyword searches of academic databases; citation searching; reviewing grey literature, etc.).  The specific sources used to identify literature.  The process of screening studies.  The number of records identified in initial searches.  The number of unique studies included in the review.  - Authors must have conducted a systematic coding and analysis of included studies.  The methods of coding and analysis should be clearly outlined, and justified.  Where relevant, methods used to conduct meta-analysis must be specified. | | | | | Does not meet the specified criteria |

**Appendix III. Quality Assessment Tools**

Part I: Countering radicalisation to violence

| **Risk of Bias: EPHPP Quality Assessment Tool^[2]^** | | |
| --- | --- | --- |
| **A Selection Bias** | Are the individuals selected to participate in the study likely to be representative of the target population? | 1 Very likely  2 Somewhat likely  3 Not likely  4 Can’t tell |
|  | What percentage of selected individuals agreed to participate? | 1 80 - 100%  2 60 – 79%  3 less than 60%  4 Not applicable  5 Can’t tell |
|  | Overall rating for section | 1 Strong  2 Moderate  3 Weak |
| **B Study Design** | Indicate the study design | 1 Randomized controlled trial  2 Controlled clinical trial  3 Cohort analytic (two group pre + post)  4 Case-control  5 Cohort (one group pre + post (before & after))  6 Interrupted time series  7 Other (specify) |
|  | Was the study described as randomized? | 1 No (Go to component C)  2 Yes |
|  | If Yes  Was the method of randomization described? | 1 No  2 Yes |
|  | If Yes  Was the method appropriate? | 1 No  2 Yes |
|  | Overall rating for section | 1 Strong  2 Moderate  3 Weak |
| **C CONFOUNDERS** | Were there important differences between groups prior to the intervention?  Examples of confounders:  1 Race; 2 Sex; 3 Marital status/family; 4 Age;  5 SES (income or class); 6 Education; 7 Health status; 8 Pre-intervention score on outcome measure. | 1 Yes  2 No  3 Can’t tell |
|  | If Yes.  Indicate the percentage of relevant confounders that were controlled (either in the design (e.g. stratification, matching) or analysis)? | 1 80 – 100% (most)  2 60 – 79% (some)  3 Less than 60% (few or none)  4 Can’t Tell |
|  | Overall rating for section | 1 Strong  2 Moderate  3 Weak |
| **D BLINDING** | Was (were) the outcome assessor(s) aware of the intervention or exposure status of participants? | 1 Yes  2 No  3 Can’t tell |
|  | Were the study participants aware of the research question? | 1 Yes  2 No  3 Can’t tell |
|  | Overall rating for section | 1 Strong  2 Moderate  3 Weak |
| **E DATA COLLECTION METHODS** | Were data collection tools shown to be valid? | 1 Yes  2 No  3 Can’t tell |
|  | Were data collection tools shown to be reliable? | 1 Yes  2 No  3 Can’t tell |
|  | Overall rating for section | 1 Strong  2 Moderate  3 Weak |
| **F WITHDRAWALS AND DROP- OUTS** | Were withdrawals and drop-outs reported in terms of numbers and/or reasons per group? | 1 Yes  2 No  3 Can’t tell  4 Not Applicable (i.e. one time surveys or interviews) |
|  | Indicate the percentage of participants completing the study. (If the percentage differs by groups, record the lowest). | 1 80 -100%  2 60-79%  3 less than 60%  4 Can’t tell  5 Not Applicable (i.e. Retrospective case-control) |
|  | Overall rating for section | 1 Strong  2 Moderate  3 Weak  Not applicable |
| **G INTERVENTION INTEGRITY** | What percentage of participants received the allocated intervention or exposure of interest | 1 80 -100%  2 60-79%  3 less than 60%  4 Can’t tell |
|  | Was the consistency of the intervention measured? | 1 Yes  2 No  3 Can’t tell |
|  | Is it likely that the subjects received an unintended intervention (contamination or co-intervention) that may the results? | 1 Yes  2 No  3 Can’t tell |
| **H ANALYSES** | Indicate the unit of allocation (single code) | Community  Organization/institution  Practice/office  Individual |
|  | Indicate the unit of analysis (single code) | Community  Organization/institution  Practice/office  Individual |
|  | Are the statistical methods appropriate for the study design? | 1 Yes  2 No  3 Can’t tell |
|  | Is the analysis performed by intervention allocation status (i.e. intention to treat) rather than the actual intervention received? | 1 Yes  2 No  3 Can’t tell |
| **GLOBAL RATING** | Overall quality | 1 STRONG (No WEAK ratings)  2 MODERATE (One WEAK rating)  3 WEAK (Two or more WEAK ratings) |

| **Assessment of Qualitative Research CASP Checklist^[3]^** | | |
| --- | --- | --- |
| Question | Answer Options | Consider |
| Was there a clear statement of the aims of the research? | Yes  No  Can’t Tell | - What was the goal of the research; - Why it was thought important; - Its relevance. |
| Is a qualitative methodology appropriate? | Yes  No  Can’t Tell | - If the research seeks to interpret or illuminate the actions and/or subjective experiences of research participants; - Is qualitative research the right methodology for addressing the research goal. |
| Was the research design appropriate to address the aims of the research? | Yes  No  Can’t Tell | - If the researcher has justified the research design (e.g. have they discussed how they decided which method to use) |
| Was the recruitment strategy appropriate to the aims of the research? | Yes  No  Can’t Tell | - If the researcher has explained how the participants were selected; - If they explained why the participants they selected were the most appropriate to provide access to the type of knowledge sought by the study; - If there are any discussions around recruitment (e.g. why some people chose not to take part) |
| Was the data collected in a way that addressed the research issue? | Yes  No  Can’t Tell | - If the setting for the data collection was justified; - If it is clear how data were collected (e.g. focus group, semi-structured interview etc.); - If the researcher has justified the methods chosen; - If the researcher has made the methods explicit (e.g. for interview method, is there an indication of how interviews are conducted, or did they use a topic guide); - If methods were modified during the study. If so, has the researcher explained how and why; - If the form of data is clear (e.g. tape recordings, video material, notes etc.); - If the researcher has discussed saturation of data |
| Has the relationship between researcher and participants been adequately considered? | Yes  No  Can’t Tell | - If the researcher critically examined their own role, potential bias and influence during (a) formulation of the research questions (b) data collection, including sample recruitment and choice of location; - How the researcher responded to events during the study and whether they considered the implications of any changes in the research design. |
| Have ethical issues been taken into consideration? | Yes  No  Can’t Tell | - If there are sufficient details of how the research was explained to participants for the reader to assess whether ethical standards were maintained; - If the researcher has discussed issues raised by the study (e.g. issues around informed consent or confidentiality or how they have handled the effects of the study on the participants during and after the study); - If approval has been sought from the ethics committee. |
| Was the data analysis sufficiently rigorous? | Yes  No  Can’t Tell | - If there is an in-depth description of the analysis process; - If thematic analysis is used. If so, is it clear how the categories/themes were derived from the data; - Whether the researcher explains how the data presented were selected from the original sample to demonstrate the analysis process; - If sufficient data are presented to support the findings; - To what extent contradictory data are taken into account; - Whether the researcher critically examined their own role, potential bias and influence during analysis and selection of data for presentation. |
| Is there a clear statement of findings? | Yes  No  Can’t Tell | - If the findings are explicit; - If there is adequate discussion of the evidence both for and against the researcher’s arguments; - If the researcher has discussed the credibility of their findings (e.g. triangulation, respondent validation, more than one analyst); - If the findings are discussed in relation to the original research question |
| How valuable is the research? | Free text. | - If the researcher discusses the contribution the study makes to existing knowledge. - If they identify new areas where research is necessary - If the researchers have discussed whether or how the findings can be transferred to other populations or considered other ways the research may be used.   Note: For review purposes, we converted free text for this question into a yes/ no answer to the question of whether the research was valuable based on this criteria. |

Part II: Countering other forms of violence

| **Quality Assessment – AMSTAR II** | | |
| --- | --- | --- |
| **Quality –**  **AMSTAR 2^[4]^** | Did the research questions and inclusion criteria for the review include the components of PICO? | Yes  No |
|  | Did the report of the review contain an explicit statement that the review methods were established prior to conduct of the review and did the report justify any significant deviations from the protocol? | Yes  Partial Yes  No |
|  | Did the review authors explain their selection of the study designs for inclusion in the review? | Yes  No |
|  | Did the review authors use a comprehensive literature search strategy? | Yes  Partial Yes  No |
|  | Did the review authors perform study selection in duplicate? | Yes  No |
|  | Did the review authors perform data extraction in duplicate? | Yes  No |
|  | Did the review authors provide a list of excluded studies and justify the exclusions? | Yes  Partial Yes  No |
|  | Did the review authors describe the included studies in adequate detail? | Yes  Partial Yes  No |
|  | Did the review authors use a satisfactory technique for assessing the risk of bias (RoB) in individual studies that were included in the review?  Note: Separate criteria for Randomised Controlled Trials (RCTs) and Non-randomised studies of Interventions (NRSI) | **RCTs**  Yes  Partial Yes  No  Includes only NRSI  **NRSI**  Yes  Partial Yes  No  Includes only RCTs |
|  | Did the review authors report on the sources of funding for the studies included in the review? | Yes  No |
|  | If meta-analysis was performed did the review authors use appropriate methods for statistical combination of results?  Note: Separate criteria for Randomised Controlled Trials (RCTs) and Non-randomised studies of Interventions (NRSI) | **RCTs**  Yes  No  No meta-analysis conducted  **NRSI**  Yes  No  No meta-analysis conducted |
|  | If meta-analysis was performed did the review authors assess the potential impact of RoB in individual studies on the results of the meta-analysis or other evidence synthesis? | Yes  No  No meta-analysis conducted |
|  | Did the review authors account for RoB in individual studies when interpreting/ discussing the results of the review? | Yes  No |
|  | Did the review authors provide a satisfactory explanation for, and discussion of, any heterogeneity observed in the results of the review? | Yes  No |
|  | If they performed quantitative synthesis did the review authors carry out an adequate investigation of publication bias (small study bias) and discuss its likely impact on the results of the review? | Yes  No  No meta-analysis conducted |
|  | Did the review authors report any potential sources of conflict of interest, including any funding they received for conducting the review? | Yes  No |
| **Quality - Overall** | Overall assessment | Free text: discuss overall quality of review. |

**Appendix IV. Data Extraction Tools**

Part I: Countering radicalisation to violence

| **Category** | **Field** | | **Type** | |
| --- | --- | --- | --- | --- |
| **Study**  **Information** | Author and year | | Free text – format: Surname(s) (Year) | |
|  | Title | | Free text – study title | |
|  | Source type | | Pre-defined codes (single code)   - Journal article - Book - Book chapter - Think tank report - Government/ public agency report - Dissertation/ thesis - Other (specify) | |
|  | Geographical focus | | Free text – country where study conducted | |
|  | Language | | Free text – language | |
| **Methods** | Type of research | | Pre-defined codes (single code)   - Quantitative - Qualitative - Mixed methods | |
|  | Quantitative  Research design | | Pre-defined codes (single code)   - Experimental (specify) - Strong quasi-experimental (specify) - Weaker quasi experimental (specify) - Other (specify) - N/A | |
|  | Qualitative Research Design | | - Free text – research design. - N/A | |
|  | Comparator condition | | Pre-defined codes   - Treatment as usual - Alternative treatment - No treatment - Wait list - N/A | |
|  | Randomisation | | Pre-defined codes:   - Randomised - Quasi-randomised - Non-randomised - Other (specify) | |
|  | Description of data collection and analysis | | Free text – basic description of data collection and data analysis approach. | |
|  | Population of study | | Pre-defined codes (multi-code if needed)   - Practitioners - Clients - Other (specify) | |
|  | Sample | | Free text – description of sample. Specify:   - Sample demographics (if available) - Eligibility criteria - Any sub-groups analysed - Any other pertinent information. | |
|  | Evidence relevant to research objectives | | Pre-defined codes (multi-code if needed)   - Impact on radicalisation to violence - Process of implementation | |
| **Intervention Details^[5]^** | Focus | | Pre-defined codes (multi-code if needed):   - Intervention(s) as-a-whole - Specific tool(s) (e.g. risk assessment) - Specific Approach(es) (e.g., RNR, strengths-based, etc.) | |
|  | **Intervention Details I:**  Intervention name | | Free text: name of intervention  N/A | |
|  | **Intervention Details II:**  Intervention description | | Free text: basic description of intervention  N/A | |
|  | **Tool Details I:**  Tool name | | Free text: name of tool  N/A | |
|  | **Tool Details II:**  Tool description | | Free text: basic description of tool  N/A | |
|  | **Approach Details I:**  Description of approach | | Free text: implicit or explicit theory of change of intervention/ tools examined. | |
|  | **Approach Details II:**  Theory of change components | | Pre-defined codes.  Answer all (multi-code if needed):  Drivers (I): Type of Driver   - Risk Factors - Protective Factors - Don’t know   Drivers (II): Domains   - Socio-demographic - Attitudinal - Psychological/ Personality - Experiential - Criminogenic - Don’t know   Drivers (III) Level of analysis   - Micro - Meso - Exo - Macro - Don’t know   Mechanisms   - Strengths-based mechanisms - Risk-oriented mechanisms - Don’t know   Progress/ Outcome Measures (I)   - Attitudinal - Motivational - Behavioural - Don’t know   Progress/ Outcome Measures (II)   - Reduction in risk factors - Development of protective factors - Building of strengths or skills - Don’t know | |
|  | Stage of case management | | Pre-defined codes (multi-code if needed):   - Identification/ referral - Risk assessment - Case planning - Implementation - Monitoring/ evaluation - Transition/ exit - All (single code) - Not specified (single code) | |
| **Intervention Context** | Country of delivery | | Free text – country of delivery. | |
|  | Population | | Pre-defined codes (multi-code if needed)   - Secondary prevention - Tertiary prevention | |
|  | Context | | Free text - brief description of intervention context including any implementation and moderator factors as listed in Section 1.2.6. | |
|  | Delivery agents | | Free text – list the different types of practitioners who deliver intervention. | |
|  | Transferability across contexts | | Free text – qualitative assessment of extent to which findings would be relevant outside specific context examined in study. | |
|  | | | | |
| **Other Synthesis Research Question 2: Implementation** | | | | |
| **Q2a – Process of Implementation**  **(if relevant)** | | Measure of implementation | | Free text – list/ describe measures |
|  |  | Description of measure | | Free text – name and description of the progress/ outcome measure. |
|  |  | Source of data used in original analysis. | | Pre-defined codes   - Self-report - Observation - Official source - Interview   Other (specify) |
|  |  | Type of respondent. | | Free text – description of the type of respondent from which data was collected (e.g. client, practitioner etc.) |
|  |  | Implemented in way expected? | | Free text – description of extent to which implemented in way expected. |
|  |  | Evidence in support specific intervention, tool, or approach | | Free text – positive research findings (page number) |
|  |  | Evidence opposed to specific intervention, tool, or approach | | Free text – negative research findings (page number) |
|  |  | Study author(s)’ conclusions | | Free text – outline conclusions drawn in original study about process. |
|  |  | Coder’s conclusions | | Free text –coder conclusions about process of implementation. |
| **Q2b – Implementation Factors/ Moderators**  **(if relevant)** | | Implementation factors/ moderators. | | Free text – describe factors/ moderators discussed. |
|  |  | Source of data used in original analysis. | | Pre-defined codes   - Self-report - Observation - Official source - Interview   Other (specify) |
|  |  | Type of respondent. | | Free text – description of the type of respondent from which data was collected (e.g. client, practitioner etc.) |
|  |  | Evidence of implementation factors/ moderators having a positive effect on process. | | Free text – positive findings |
|  |  | Evidence of implementation factors/ moderators having a negative effect on process. | | Free text – negative findings |
|  | | Study author(s)’ conclusions | | Free text – outline conclusions drawn in original study about factor(s) |
|  |  | Coder’s conclusions | | Free text –coder conclusions about factor/ moderator. |

Part II: Countering other forms of violence - Review coding

| **Category** | **Field** | **Type** |
| --- | --- | --- |
| **Study**  **Information** | Author and year | Free text – format: Surname(s) (Year) |
|  | Title | Free text – study title |
|  | Source type | Pre-defined codes (single code)   - Journal article - Book - Book chapter - Think tank report - Government/ public agency report - Dissertation/ thesis - Other (specify) |
|  | Geographical focus | Free text – country where study conducted |
|  | Language | Free text – language |
| **Methods** | Type of study | Pre-defined codes (multi-code if needed)   - Systematic review - Meta-analysis - Other synthesis (specify) |
|  | Research designs included | Pre-defined codes (multi-code if needed)   - Experimental (specify) - Strong quasi-experimental (specify) - Weaker quasi experimental (specify) - Other (specify) |
|  | Comparator conditions | Pre-defined codes   - Treatment as usual - Alternative treatment - No treatment - Wait list - Other (specify) - N/A |
|  | Randomisation | Pre-defined codes:   - Only randomised studies included - Non-randomised studies included |
|  | Research designs included | Free text – specific research designs. |
|  | Description of data collection and analysis | Free text – basic description of data collection and data analysis approach. Include inclusion and exclusion criteria. |
|  | Population of study | Pre-defined codes (multi-code if needed)   - Practitioners - Clients - Other (specify) |
|  | Number of studies included | Number |
|  | Evidence relevant to research objectives | Pre-defined codes (multi-code if needed)   - Impact on violence - Implementation |
| **Intervention Details^[6]^** | Focus | Pre-defined codes (multi-code if needed)   - Intervention(s) as a whole - Specific Tool(s) (e.g. risk assessment) - Specific Approach(es) (e.g. RNR, strengths-based) |
|  | Type of violence (I) | Pre-defined codes (multi-code if needed)   - Interpersonal - Collective |
|  | Type of violence (II) | Free-text: focus of the intervention/ tool/ approach (e.g. gang-related; domestic violence; general violent offending etc.) |
|  | **Intervention Details I:**  Intervention name | Free text: name of intervention  N/A |
|  | **Intervention Details II:**  Intervention description | Free text: basic description of intervention  N/A |
|  | **Tool Details I:**  Tool name | Free text: name of tool  N/A |
|  | **Tool Details II:**  Tool description | Free text: basic description of tool  N/A |
|  | **Approach Details I:**  Intervention approach | Free text: implicit or explicit theory of change of intervention/ tools examined. |
|  | **Approach Details II:**  Theory of change components | Free-text  Answer all:  Drivers:  Describe drivers, considering:   - Does the intervention consider risk/ and or protective factors? - Does the intervention consider different types of driver (e.g. demographic, attitudinal etc.)? - Does the intervention consider factors at different levels of analysis?   Mechanisms:  Describe mechanisms, considering:   - Does the review explicitly examine a specific model of rehabilitation or some combination of models (e.g., RNR, strengths-based, GLM, etc.)? - Do assumptions (explicit or implicit) underpinning the intervention/ tool/ approach align with specific model(s) of rehabilitation?   Progress/ Outcome measures  Describe measures, considering:   - What types of measures are used (e.g. attitudinal, behavioural, etc.)? - How do they relate to intervention outcomes (e.g., do they relate to a reduction in risk, building protective factors, etc.)? |
|  | Stage of case management | Pre-defined codes (multi-code if needed)   - Identification/ referral - Risk assessment - Case planning - Implementation - Monitoring/ evaluation - Transition/ exit - All (single code) - Not specified (single code) |
| **Intervention Context** | Country of delivery | Free text – country of delivery. |
|  | Population | Pre-defined codes (multi-code if needed)   - Secondary prevention - Tertiary prevention |
|  | Context | Free text - brief description of intervention context including any implementation and moderator factors as listed in Section 1.2.6. |
|  | Delivery agents | Free text – list the different types of practitioners who deliver intervention. |
|  | Transferability across contexts | Free text – qualitative assessment of extent to which findings relevant outside of the specific context examined in study. |
| **Q 3. Progress and Outcome Measures** | Tools used to monitor client-level progress | Free text – description of how client-level progress are captured (if relevant) |
|  | Client-level progress measures | Free text – list of client-level progress measures (if relevant) |
|  | Tools used to measure client-level outcomes | Free text – description of how client-level outcome measures are captured (if relevant) |
|  | Client-level intervention outcomes  (Primary) | Free text – list/ describe primary outcomes (e.g. prevent radicalisation; promote disengagement/ deradicalisation etc.) |
|  | Client-level intervention outcomes  (Secondary) | Free text – list/ describe secondary outcomes (e.g. specific risk/ protective factors) |
| **Q4. Implementation Process**  **(if relevant)** | Qualitative data | Free text – description of key data presented on implementation. |
|  | Quantitative data. | Free text – present data from any quantitative analysis incl. meta-analysis.  Include: effect sizes, confidence intervals,  assessment of heterogeneity (if relevant). |
|  | Author Conclusions | Free text – author conclusions on process |
|  | Coder Conclusions | Free text – coder conclusions on process |
| **Q4.**  **Implementation Factors**  **(if relevant)** | Qualitative data | Free text – description of key data presented on implementation factors. |
|  | Quantitative data. | Free text – present data from any quantitative analysis incl. meta-analysis.  Include: effect sizes, confidence intervals,  assessment of heterogeneity (if relevant). |
|  | Author Conclusions | Free text – author conclusions on factors |
|  | Coder Conclusions | Free text – coder conclusions on factors |
| **Impact on violence**  **(if relevant)** | Qualitative synthesis of evidence | Free text – description of key data presented on effectiveness. |
|  | Results from quantitative analysis | Free text – present data from any quantitative analysis incl. meta-analysis.  Include: effect sizes, confidence intervals,  assessment of heterogeneity (if relevant). |
|  | Conclusions – are tools and approaches effective? | Free text – summary of conclusions. |
| **Transferability to countering radicalisation to violence** | Transferable lessons | Free text – qualitative assessment of transferability including discussion of key findings relevant to interventions seeking to counter radicalisation to violence. |

1. LOE refers to languages other than English throughout.
2. https://www.ephpp.ca/PDF/Quality%20Assessment%20Tool_2010_2.pdf
3. Available at https://casp-uk.net/casp-tools-checklists/. The original tool also includes a question relating to how the value of the research that was not used to assess studies.
4. From Shea et al. (2017). The criteria for coding each question as Yes, Partial Yes (where relevant), or No will be based on the more detailed criteria included in the full AMSTAR 2 tool, which is available from https://www.bmj.com/content/bmj/suppl/2017/09/21/bmj.j4008.DC1/sheb036104.wf1.pdf.
5. If more than one intervention, tool or approach is examined within an individual study, a separate row will be included to capture details of all each intervention, tools and approaches with empirical data to report.
6. If more than one intervention, tool or approach examined within an individual study, a separate row will be included to capture details of each intervention, tool or approach with empirical data to report.
